# Combining proteomic markers to construct a logistic regression model for polycystic ovary syndrome

**DOI:** 10.3389/fendo.2023.1227252

**Published:** 2023-10-03

**Authors:** Cheng Tong, Yue Wu, Zhenchao Zhuang, Zhejiong Wang, Ying Yu

**Affiliations:** ^1^ Department of Laboratory Medicine, The First Affiliated Hospital of Zhejiang Chinese Medical University (Zhejiang Provincial Hospital of Chinese Medicine), Hangzhou, China; ^2^ The First School of Clinical Medicine, Zhejiang Chinese Medical University, Hangzhou, Zhejiang, China

**Keywords:** polycystic ovary syndrome, proteomics, logistic regression, DIA mass spectrometry, histones

## Abstract

**Introduction:**

Proteomics technology has been used in various fields in recent years for the Q6 exploration of novel markers and the study of disease pathogenesis, and has become one of the most important tools for researchers to explore unknown areas. However, there are fewer studies related to the construction of clinical models using proteomics markers.

**Methods:**

In our previous study we used DIA proteomics to screen for proteins that were significant in 31 PCOS patients compared to women of normal reproductive age. In this study, we used logistic regression among these protein markers to screen out variables with diagnostic value and constructed logistic regression models.

**Results:**

We constructed a logistic model using these protein markers, where HIST1H4A (OR=1.037) was an independent risk factor for polycystic ovary syndrome and TREML1 (OR=0.976) were protective factors for the disease. The logistic regression model equation is: Logit (PCOS) =0.036*[HIST1H4A]-0.024*[TREML1]-16.368. The ROC curve analyzing the diagnostic value of the model has an AUC value of 0.977 and a Youden index of0.903, which gives a cutoff value of 0.518 at this point. The model has a sensitivity of 93.5% and a specificity of 96.8%. Calibration curves show fair consistency of the model.

**Discussion:**

Our study is the first to use proteomic results with clinical biochemical data to construct a logistic regression model, and the model is consistent. However, our study still needs a more complete sample to confirm our findings.

## Introduction

Polycystic ovary syndrome (PCOS) is one of the most common gynecological disorders in women of reproductive age and is thought to be a metabolic syndrome caused by a combination of environmental, endocrine and genetic factors. According to statistics, approximately 10% of women of reproductive age worldwide suffer from PCOS, which is also associated with insulin resistance, hyperandrogenemia, oligomenorrhoea and amenorrhea (1). Previous studies have shown that patients with PCOS have a higher incidence of pregnancy complications than healthy women, such as miscarriage, hypertensive disorders of pregnancy, preterm delivery and infertility (2–5). The most serious risks for patients with PCOS in their reproductive years are increased risk of infertility and miscarriage, which can be caused by ovulation disorders, abnormal endocrine metabolism and impaired egg development (6), and all are closely related to the inflammatory response (7). In addition to the serious risks to the physical health of the patient, PCOS can also have a negative impact on mental health. Some research studies have found that many PCOS patients suffer from varying degrees of anxiety, depression and other psychological problems (8). Therefore, timely diagnosis of PCOS and early intervention and prevention of the disease are crucial for women of childbearing age.

Due to the complexity of the causes of PCOS and the lack of clarity on its pathogenesis, there has been a long process of identifying guidelines for the treatment of PCOS, and many guidelines and expert consensuses have been proposed. The 2003 Rotterdam Consensus Workshop recognized PCOS as a syndrome of ovarian dysfunction (9), the diagnostic criteria require at least two of the following three features to be met (1): clinical presentation or biochemical indication of hyperandrogenemia; (2) Irregular or non-ovulatory menstruation; and (3) polycystic ovarian morphology (PCOM): that is, ultrasonography indicating ≥12 follicles with a maximum diameter of 2-9 mm or any ovarian volume >10 ml. Other conditions that may cause hyperandrogenemia or reduced ovulation should also be excluded. In addition, many international experts have put forward expert consensus on the treatment of PCOS, such as the NIH criteria developed by the National Institutes of Health in 1990 (10), AES standard developed by the American AE-PCOS Association in 2006 (11), and the Diagnostic Criteria for Polycystic Ovarian Syndrome issued by our Ministry of Health in 2011 (12). Such a variety of diagnostic criteria undoubtedly poses a challenge for the diagnosis of PCOS. A community sample study reported a 12% prevalence of PCOS when PCOM was not included in the required diagnostic criteria, a value that rose to 18% when PCOM was included in the required diagnostic criteria (13). However, PCOS is a heterogeneous disease, leading to a wide variety of phenotypes that can occur in PCOS. For example, elevated LH/FSH is often considered clinically in the diagnosis of PCOS, but 1/3 of patients with PCOS have normal levels of LH or FSH (14). Using modern ultrasound techniques, it will be found that approximately 80% of young women will develop PCOM (15). The diagnostic criteria for PCOS have been gradually harmonized in recent years, with the 2023 update of the “*International evidence-based guideline for the assessment and management of polycystic ovary syndrome”* (16). The guideline improves on past problems of inconsistent standards, lack of evidence-based processes, and inconsistency in assessment and management by implementing more rigorous development rules, while providing more evidence-based recommendations and clinical consensus. The guidelines recommend irregular menstruation and clinical hyperandrogenism as the first steps in diagnosing PCOS. Biochemical hyperandrogenism can be tested when the patient does not present with clinical hyperandrogenism. However, even though indicators such as total testosterone and free androgen index can help identify hyperandrogenemia, however, the quality of evidence for these is low; in other words, current conventional markers are difficult to meet clinical diagnostic needs, and more research is needed around the diagnosis of PCOS. Proteins are involved in a variety of reactions in the body and their biological properties determine the physiological processes in which they are involved, therefore quantitative and qualitative analysis of proteins can provide important and relevant information for research in a number of fields worldwide. In recent years, proteomics has been increasingly used in the study of the pathogenesis of various diseases. Liquid chromatography-tandem mass spectrometry (LC-MS/MS) based proteomics techniques provide a broad platform for protein quantification studies, and are more sensitive, selective and have better protein coverage than traditional western blotting (Wb) and enzyme-linked immunosorbent assay (ELISA) (17). Commonly used LC-MS/MS based proteomics techniques include two main targeted methods: selective/multi-response monitoring (SRM/MRM) and parallel reaction monitoring (PRM), and two non-targeted methods: data-dependent acquisition (DDA) and data-independent acquisition (DIA) (18). Non-targeted methods have a wider protein coverage compared to targeted methods, of which the DIA technique is a new technique developed in recent years, with the advantage that it not only has the high sensitivity and reproducibility of the PRM technique, but also covers the advantages of the high protein coverage of the DDA technique (17). In our previous study, we used this technique to screen for more than 80 differential proteins in 31 patients with PCOS (experimental group) and in 31 healthy women of the same age (control group) over the same period (19).

Although many studies in recent years and our study have jointly elaborated the proteomic expression of circulating levels in PCOS patients (20, 21), how to apply the results of these studies to the clinic is still a problem that needs to be solved. Therefore, we would like to construct a regression model and evaluate the model based on the results of proteomic screening in our previous study.

## Materials and methods

### Study subjects

31 women diagnosed with PCOS who visited the gynecology clinic of the First Affiliated Hospital of Zhejiang University of Traditional Chinese Medicine from December 2018 to April 2019 were included as the experimental group, whose diagnostic criteria met the Rotterdam diagnostic criteria (9): (1) having hyperandrogenemia or having clinical manifestations of hyperandrogenemia; (2) polycystic ovarian-like changes under ultrasound (Polycystic (2) Polycystic Ovarian Morphology (PCOM): ≥12 follicles of 2-9 mm in diameter and/or ovarian volume ≥10 ml in one or both ovaries on ultrasound; (3) Irregular uterine bleeding or irregular menstruation. A diagnosis of PCOS was considered to be made when two of the three criteria were met, but Cushing’s syndrome, hyperprolactinemia, congenital adrenocortical hyperplasia, thyroid disorders, androgenic tumors, and other conditions that can cause irregular menstruation or irregular uterine bleeding should also be excluded. 31 people who attended the First Hospital of Zhejiang University of Traditional Chinese Medicine for health check-ups during the same period were also included as the control group. Inclusion criteria for the control group: 1. having a normal menstrual cycle (25-35 days); 2. no hyperandrogenemia or clinical hyperandrogenemia manifested. All subjects were between 18-37 years of age and were excluded from taking hormonal drugs or drugs that may affect insulin sensitivity in the last 3 months, as well as those with severe liver and renal insufficiency, cardiac disease and autoimmune diseases. The study was approved by the Ethics Committee of The First Affiliated Hospital of Zhejiang Chinese Medical University (Zhejiang Provincial Hospital of Traditional Chinese Medicine). All participants signed a written informed consent.

### Sample and clinical data collection

Subjects were collected early morning fasting blood on the 3rd-5th day of their menstrual period. Biochemical and female hormonal markers such as follicle stimulating hormone (FSH), prolactin (PRL), luteinizing hormone (LH), total cholesterol (TC), total testosterone (T), triglycerides (TG), low density lipoprotein (LDL), high density lipoprotein (HDL) were collected from all PCOS patients and control participators by IMMULITE 2000 analyzer (Siemens Medical Diagnostic Products Ltd, UK), using a two-site chemiluminescent immunoassay.

### Removal of high-abundance proteins

All 62 plasma samples were taken in 10ul (approx. 600ug), added to a TOP14 High Abundance Protein Removal Centrifuge Column (Thermo) and incubated for 30min at room temperature. Centrifugation was carried out at 1000g for 2 min and the centrifuged solution was approximately 320μl in 10mM PBS, 0.15M NaCl, 0.02% azide, pH 7.4.

### Sample digestion

Samples with high protein abundance removed were added to a 10kD ultrafiltration membrane (Millipore) and centrifuged at 12,000g for 10min; 200μl of 8 M Urea was added, also centrifuged at 12,000g for 10min, and finally 50μl of 8M urea was added and the samples were added to a 96 well plate.

### Peptide desalination

100μl of methanol was added to a SOLA μ HRP plate and centrifuge at 600g for 1min; subsequently 100 μl of 80% ACN 0.1% TFA was added to the reaction system and centrifuged at 1000 g for 1 min.; added 200μl of 0.1% THA and centrifuge at 1000g for 1min; the samples were added to SoLAμ HRP plates, and centrifuge at 1000g for 2min; added 100μl of 80% ACN 0.1% TFA and centrifuge at 1000g for 1min; added 200μl of 0.1% THA and centrifuge at 1000g for 1min; The sample was repeated once; 200 μl of 0.1% THA was added, centrifuged at 1000g for 2 min; 100 μl of 80% ACN 0.1% TFA was added, centrifuged at 1000g for 3 min and the eluate was collected; the eluate was spun dry at 40°C in a centrifuge concentrator, dissolved to 0.5 μg/μl with 0.1% FA, and 1 μg of mass spectrometry was added for DIA quantitative analysis.

### Data statistics and analysis

The proteomic data were adjusted for Benjamin-Hocheberg FDR; unpaired two-tailed student t-tests were used for clinical biochemical and hormonal results and were considered statistically significant at a p-value <0.05 (prior studies were completed) (19). The proteomic data were further analyzed to screen for five markers that we felt had better diagnostic value and disease relevance for inclusion in the follow-up study. One-way binary logistic regression analysis of protein markers and clinical indicators was performed using SPSS 25.0 and considered significant at <0.05 to screen candidates for inclusion in model construction. In order to prevent multicollinearity between variables from interfering with the results of the study, we used Lasso regression to further screen the screened markers. Finally, multi-factor binary logistic regression (stepwise forward method) was used to analyze the correlation between the variables included in the model construction and disease occurrence and to screen for independent risk factors. The degree of model fit was assessed by the Hosmer-Leeshawn goodness-of-fit test. The effects of variables in the logistic regression model were described by ratio (OR) and 95% confidence interval. The ratio of each parameter was defined, with OR > 1 being considered to be associated with an increased risk of developing the disease (risk factor) and OR < 1 being associated with a reduced risk (protective factor). Subject operating characteristic curves (ROC curves) were plotted and the area under the curve was calculated to predict model efficacy. Nomograms were plotted using R4.2.2 software and associated packages to visualize the risk of disease, and internal validation was performed using Bootstrap and associated packages to randomize the sample, plotting the Calibration curve and calculating the C-index to assess model performance.

## Results

**Figure 1:**
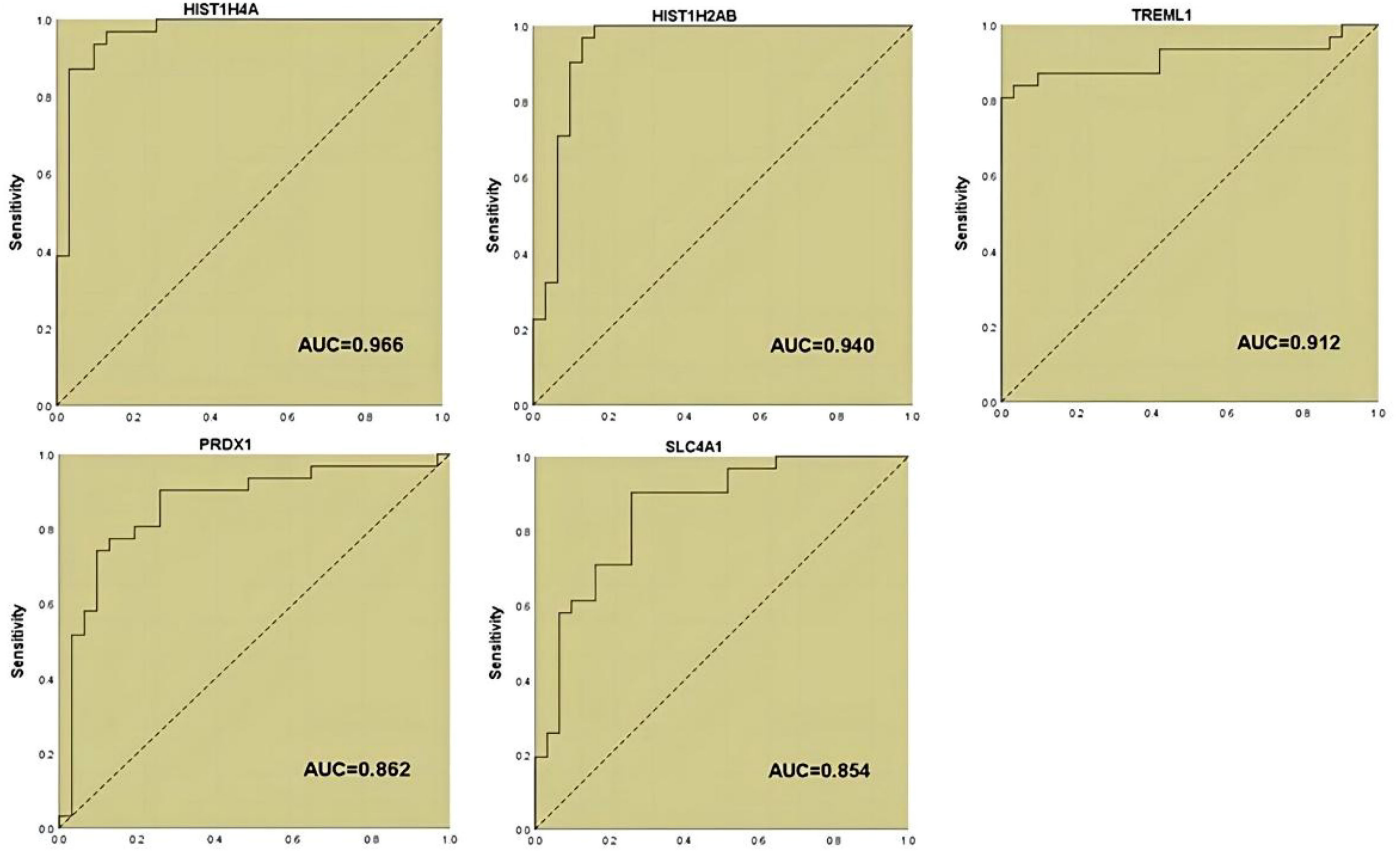
ROC analysis of the five proteins that were initially included in the model construction.

In our previous study (19), we applied DIA proteomics techniques to screen for proteins and clinical assays that were differentially associated in PCOS in 62 subjects (31 with PCOS versus 31 women of normal reproductive age. Here, we performed one-way logistic regression analysis on these assays and the five protein markers that we considered to be of most diagnostic value to further clarify the relationship with PCOS, p<0.05 was considered to be significant (**Table 1**; **Figure 1**).

**Table 1:** Univariate logistic regression analysis of potential factors of PCOS in the modelling data sets.

Variables	B	SE	Wald	P	OR	95% CI
LH (IU/L)	0.169	0.072	5.597	0.018	1.185	1.029-1.363
LH/FSH	1.098	0.412	7.100	0.008	2.998	1.337-6.724
PRL (IU/L)	-0.005	0.002	7.429	0.006	0.995	0.992-0.999
T (nmol/L)	1.797	0.568	10.020	0.002	6.031	1.982-18.348
HIST1H4A (ng/dL)	0.045	0.012	14.581	<0.001	1.046	1.022-1.070
HIST1H2AB (ng/dL)	0.028	0.007	17.678	<0.001	1.028	1.015-1.042
TREML1 (ng/dL)	-0.042	0.011	15.585	<0.001	0.959	0.939-0.979
PRDX1 (ng/dL)	0.022	0.006	14.635	<0.001	1.023	1.011-1.034
SLC4A1 (ng/dL)	0.014	0.003	15.367	<0.001	1.014	1.007-1.021
FBG (mmol/L)	1.759	0.759	5.368	0.021	5.804	1.311-25.689
TG (mmol/L)	2.675	0.999	7.168	0.007	14.506	2.047-102.772
HDL (mmol/L)	-2.576	1.058	5.924	0.015	0.076	0.010-0.606
LDL (mmol/L)	1.025	0.569	3.239	0.072	2.787	0.913-8.508

B, regression coefficient; SE, standard error; OR, odds ratio; CI, confidence interval; HIST1H4A, Histone H4; HIST1H2AB, Histone H2A; TREML1, Trem-like transcript 1 protein; PRDX1, Peroxiredoxin-1; SLC4A1, Band 3 anion transport protein.

**Figure 2:**
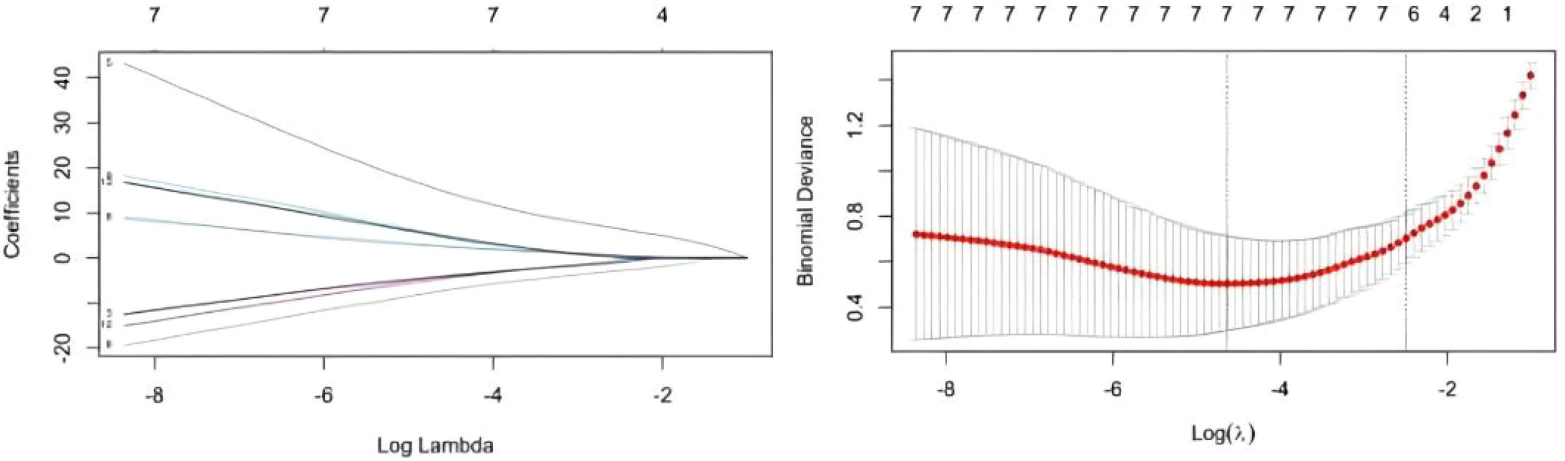
Results of Lasso regression analysis. A total of six most likely non-zero coefficient characteristic variables were screened based on LASSO regression according to the indicators for which the difference in univariate analysis was statistically significant, including PRL, HIST1H4A, TREML1, SLC4A1.

**Figure 3:**
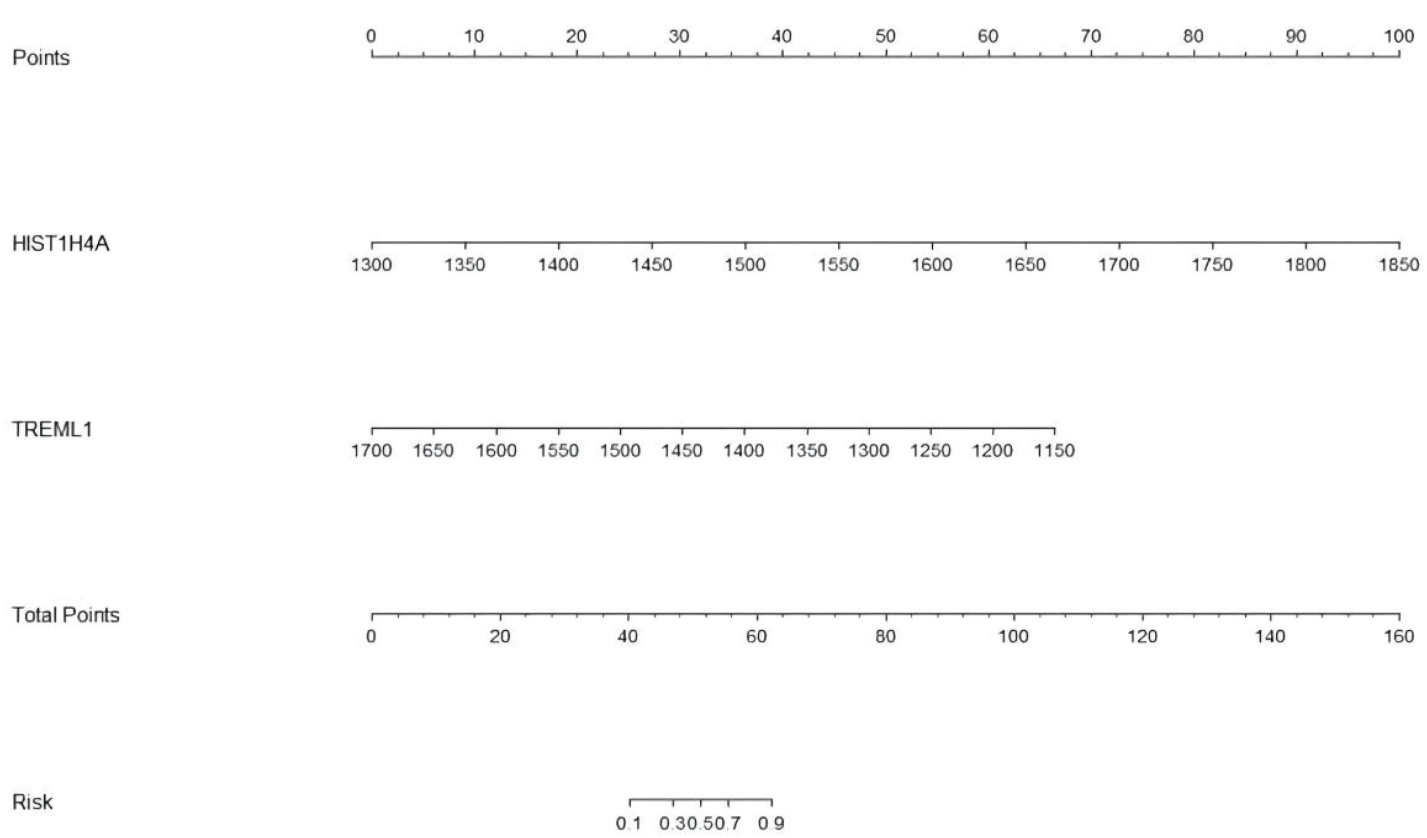
The Nomograms model of PCOS. The Nomograms model gives each patient a point based on their HIST1H4A and TREML1 (ng/dL) results, and the total points from the three results gives the patient's risk of disease.

**Figure 4:**
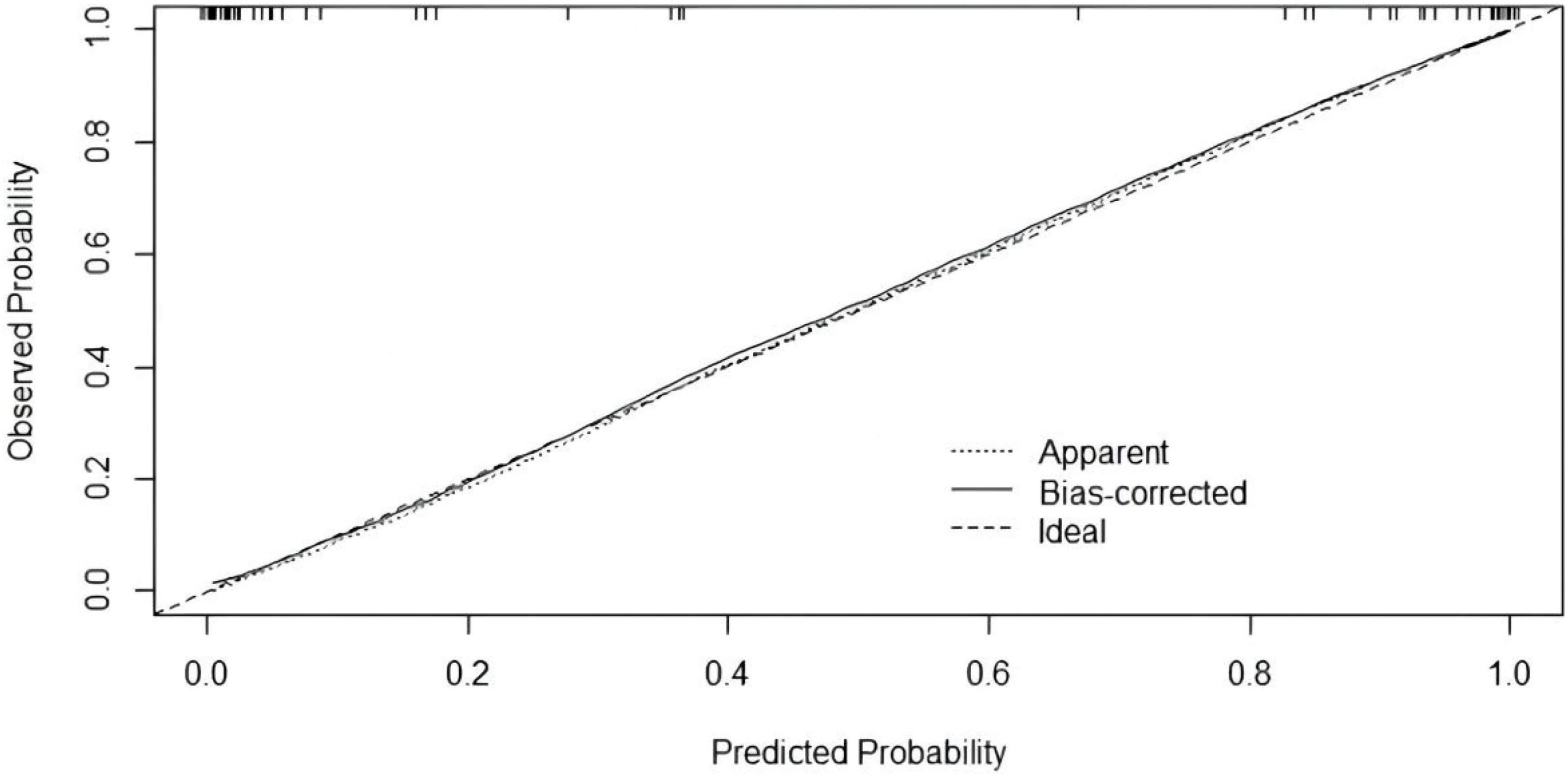
Logistic regression model for Calibration curve analysis.

**Figure 5:**
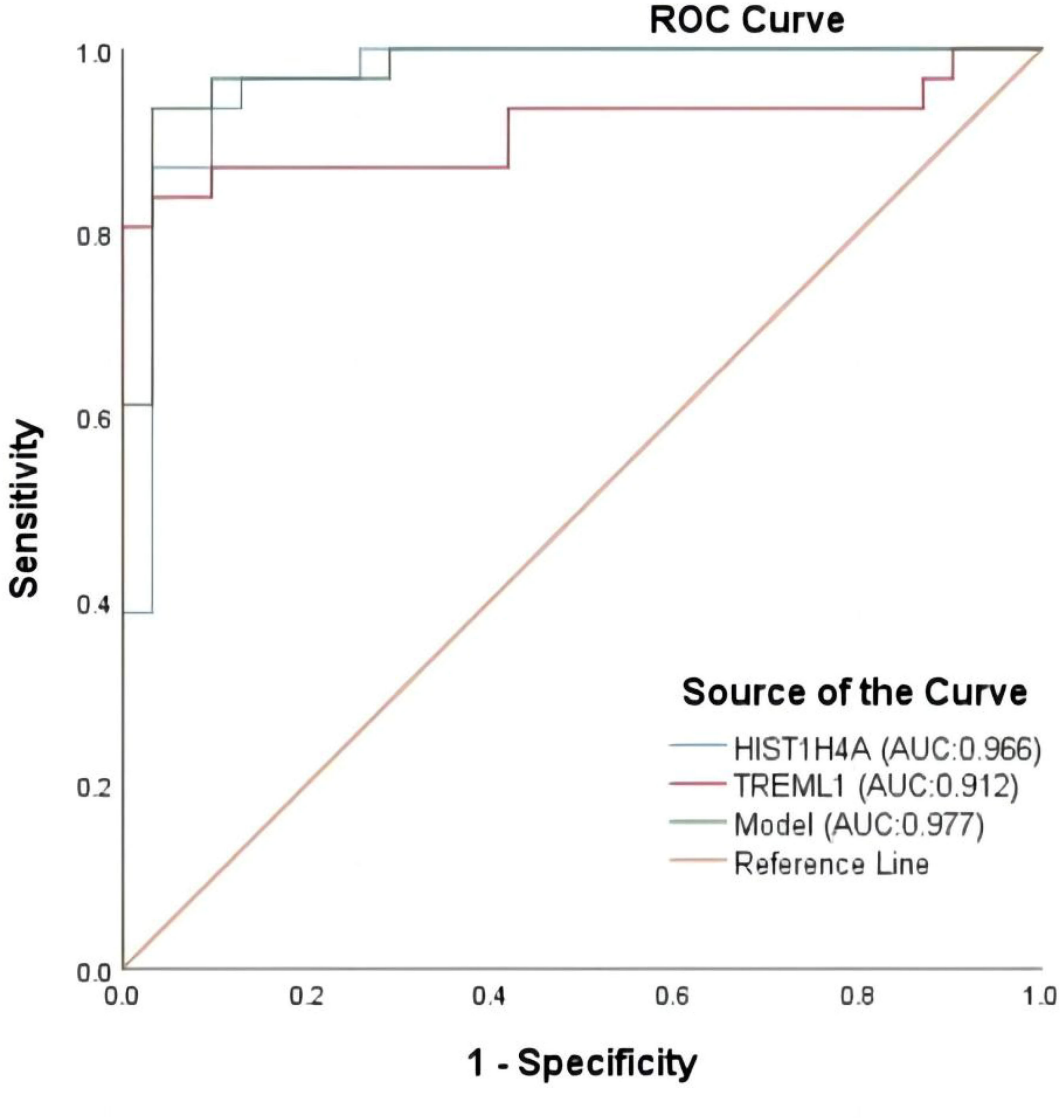
Comparison of ROC curves for model and differential variables.

Twelve variables were screened by univariate logistic regression analysis (**Table 1**). Lasso regression analysis and Twenty-fold cross-validation were used to identify factors associated with disease, and six variables were ultimately screened, including: PRL, HIST1H4A, TREML1, SLC4A1, HIST1H2AB and HDL (**Figure 2**). The screened variables were continued to be subjected to multi-factor logistic regression analysis (stepwise forward method), and according to the multi-factor logistic regression analysis, ultimately HIST1H4A and TREML1 were deemed ready for inclusion in the model construction. Of these, HIST1H4A was an independent risk factor for polycystic ovary syndrome, TREML1were protective factors for the disease (**Table 2**). The logistic regression model equation is Logit (p=PCOS) = 0.036*[HIST1H4A]-0.024*[TREML1]-16.368, where (p=PCOS) is the probability of PCOS based on this model, [TREML1] and [HIST1H4A] are the serum concentrations of TREML1 and HIST1H4A in ng/dL respectively. The ROC curve cutoff value was 0.518, which means that PCOS can be considered when the model results are greater than 0.518. To visualize the risk of prevalence, the variables screened from the results of the multifactorial logistic regression analysis were used as independent variables, and the presence or absence of PCOS was included as the dependent variable in the Nomograms model, which was constructed using R4.2.2 software (**Figure 3**). The final variables that were included in the model are shown in the column line graph model, the results for each variable are assigned specific Points, and the final Total Point obtained by summing all the Points corresponds to the risk of illness. In order to verify the efficacy of the model, the C-index was calculated and the actual and predicted values of the model were 0.977 and 0.973, respectively, and the Hosmer-Leeshawn test for the model was 0.98 > 0.05. The Bootstrap method (repetitive sampling) was also used for internal validation with 1,000 random times from the samples, and the results showed that the constructed model fitted the calibration curve well (**Figure 4**). The essence of the Bootstrap method is to resample the observed information and thus make statistical inferences about the distributional properties of the aggregate. Bootstrap, by resampling, avoids the sample reduction problem caused by Cross-Validation, and secondly, Bootstrap can also be used to create randomness in the data. We also compared the diagnostic performance of the constructed models with the variables in aggregate and plotted ROC curves for analysis. The logistic regression model had an AUC of 0.977, outperforming all the markers screened, suggesting that the model has good value (**Figure 5**).

**Table 2:** Multivariate logistic regression analysis of potential factors of PCOS in the modelling data sets.

Variables	B	Wald	OR	95%CI	P value
HIST1H4A (ng/dL)	0.036	8.358	1.037	1.012-1.063	0.004
TREML1 (ng/dL)	-0.024	0.444	0.976	0.957-0.995	0.015

## Discussion

Proteomics is an emerging tool for testing. It can be used to characterize dynamic changes in proteins, provide potential protein markers that can be used for disease diagnosis, and provide a rich source for the diagnosis of complex diseases (22). In this study, we used DIA proteomics techniques to screen for protein markers present in PCOS patients that differ from normal women of childbearing age and considered other variables to jointly construct a logistic regression model. A diagnostic model including HIST1H4A and TREML1 was constructed and the combined marker model achieved an AUC of 0.977 in differentiating PCOS from healthy controls.

At present, due to the continuous updating of mass spectrometry detection technology, the determination of many tiny components of the human body can be easily accomplished by mass spectrometry, which is of great significance for expanding the detection spectrum and discovering new disease markers. Moreover, the technology has the advantages of gradual maturity and increasing convenience, which has already been popularized in various clinical laboratories, and at the same time, it is being used in many fields such as drug metabolism, biomedicine, and genomics research (23, 24). Histones form the core of the eukaryotic ribosome and their N-terminal amino acid residues can be covalently modified to induce transcription (25). HIST1H4A, a member of the histone family, can be involved in apoptosis and tissue inflammatory responses through the regulation of downstream proteins (26). Previous studies on HIST1H4A or histone modifications have focused on the correlation between various malignancies and chronic inflammation, while recent reviews have described the pathogenesis of PCOS as being closely related to the inflammatory response and apoptosis (27). In addition, most studies have shown that post-translational modifications of histones (including acetylation and methylation) play an important role in follicle maturation and ovulation (28, 29). Eini et al. showed that histone hyperacetylation disrupts the chromatin structure of follicles during meiosis and delays follicular maturation, leading to polycystic ovaries (30). In combination with these studies, we consider that these histones are mainly involved in the formation of PCOM and apoptosis of ovarian follicular cells in PCOS, among others.

TREML1 is a member of the innate immune receptor family expressed on myeloid cells and is highly expressed on both monocytes and macrophages. TREML1 plays an important role in adaptation to inflammatory responses, metabolic homeostasis, and regulation of platelet function. It has been demonstrated that activation of TREML1 attenuates the induction of IL-12, which is essential for the adaptation of Th1 cells to the inflammatory response (31). A study constructed an animal model of insulin resistance by means of a high-fat diet and found that TREML1 was widely up-regulated in the subcutaneous adipose tissue of these animals, but the experimental group of mice did not show significant obesity (32). Although there are no studies that can directly confirm the correlation between TREML1 and PCOS, based on the above studies we speculate that there is some role for TREML1 in helping the body to adapt to the chronic inflammatory environment and stopping the onset of obesity, but this speculation still needs to be argued by further research. Proteomics has advantages in elucidating disease progression and pathogenesis. Alterations in proteins can reflect some changes in DNA, and thus in-depth proteomic analysis may provide valuable clinically relevant information in the progression of a number of complex diseases, such as various types of cancers, neurodegenerative pathologies, and endocrine metabolic diseases. Despite the great potential and advantages of proteomics in monitoring disease progression and elucidating the biological processes involved in disease development (33, 34), the application of proteomics to PCOS is still inadequate. This study uses DIA proteomics to explore the pathogenesis of PCOS and new targets for treatment at the protein level, providing a new dimension of understanding the disease progression of PCOS. Some scholars have also used proteomics techniques to identify the expression of several proteins related to inflammatory response, complement and coagulation in the follicular fluid of PCOS patients (20), which is similar to the results we found in the patients’ serum. Previously, a predictive model for the development of insulin resistance in patients with PCOS was constructed using variables related to reproductive hormones, BMI, skin folds and insulin (35). In contrast, the proteome is much larger and more complex, with relatively independent metabolic processes and the ability to respond to the internal and external environment of the body through active interactions, thus better reflecting the progression of the disease over time and space, and providing new directions for research into the pathophysiological mechanisms of PCOS through proteomic results.

In this study, when HIST1H4A alone was used for the diagnosis of PCOS, we found that its AUC was similar to that of our model, so we statistically analyzed the sensitivity and specificity of HIST1H4A for the diagnosis of PCOS, which were 87.1% and 90.3%, respectively, whereas the model’s sensitivity and specificity were superior (93.5% and 96.8%, respectively). Meanwhile, our study shows that the diagnostic efficacy of HIST1H4A is superior, which may be related to some pathogenesis of the disease, and we believe that further exploration can be conducted around this protein marker and the related path mechanisms of PCOS, and we can validate our conjecture through more animal and cellular experiments.

However, there are some limitations of this study that need to be addressed; PCOS is a heterogeneous disease with different subtypes, such as insulin-resistant and non-insulin-resistant patients, and obese and non-obese patients, which have not been taken into account in our study for the time being. Therefore, we still need to further expand the sample size and also should perform external validation of the constructed model with multicenter data to make the results of the model more convincing. Unfortunately, factors such as fasting insulin, fasting glucose, LH, and FSH, which are considered to be highly correlated with PCOS, were not included in our model in this study, which we took into consideration for the following reasons: even though these indicators would show variability between patients with PCOS and the normal population, they still do not have enough diagnostic value to diagnose the disease, or because they did not reflect enough significance based on our sample to be eliminated in the screening of the variables and were not included in our model, which we believe may also be a result of the insufficient evidence of insufficient sample size.

## Conclusion

In summary, in this study, we developed a logistic regression model for PCOS based on proteomic markers, which may help to provide further reference for the diagnosis of PCOS and also reveal that some proteins may have certain mechanisms in the pathogenesis of PCOS. Subsequently, we expect to validate the ability of the model using a larger sample size and continue to refine the model construction, and eventually hope to better inform the diagnosis of PCOS.

## Data availability statement

The data presented in the study are deposited in the iproX repository, accession number is PXD044121.

## Ethics statement

The studies involving humans were approved by the Ethics Committee of The First Affiliated Hospital of Zhejiang Chinese Medical University (Zhejiang Provincial Hospital of Traditional Chinese Medicine). The studies were conducted in accordance with the local legislation and institutional requirements. The participants provided their written informed consent to participate in this study.

## Author contributions

CT and YW came up with the idea and design the study. CT wrote the initial draft (including substantive translation), made the tables and the figures. YW contributed to the preparation of the published work. ZZ contributed to data analysis and language revision. YY and ZW ensured that the descriptions are accurate and agreed by all authors. All authors contributed to the article and approved the submitted version.

## References

[1] Moghetti P Tosi F. Insulin resistance and pcos: Chicken or egg? J Endocrinol Invest (2021) 44(2):233–44. doi: 10.1007/s40618-020-01351-0 32648001

[2] Mayrhofer D Hager M Walch K Ghobrial S Rogenhofer N Marculescu R. The prevalence and impact of polycystic ovary syndrome in recurrent miscarriage: A retrospective cohort study and meta-analysis. J Clin Med (2020) 9(9):2700. doi: 10.3390/jcm9092700 32825545 PMC7565166

[3] Zhou S Ji Y Wang H. The risk factors of gestational hypertension in patients with polycystic ovary syndrome: A retrospective analysis. BMC Pregnancy Childbirth (2021) 21(1):336. doi: 10.1186/s12884-021-03808-3 33906610 PMC8080329

[4] Joham AE Teede HJ Ranasinha S Zoungas S Boyle J. Prevalence of infertility and use of fertility treatment in women with polycystic ovary syndrome: data from a large community-based cohort study. J Womens Health (Larchmt) (2015) 24(4):299–307. doi: 10.1089/jwh.2014.5000 25654626

[5] Robinson SL Yeung EH. Polycystic ovary syndrome and preterm birth-what's going on? Fertil Steril (2021) 115(2):326–7. doi: 10.1016/j.fertnstert.2020.09.169 PMC7882017 33272639

[6] Fauser BC Tarlatzis BC Rebar RW Legro RS Balen AH Lobo R. Consensus on women's health aspects of polycystic ovary syndrome (Pcos): the amsterdam eshre/asrm-sponsored 3rd pcos consensus workshop group. Fertil Steril (2012) 97(1):28–38.e25. doi: 10.1016/j.fertnstert.2011.09.024 22153789

[7] Abraham Gnanadass S Divakar Prabhu Y Valsala Gopalakrishnan A. Association of metabolic and inflammatory markers with polycystic ovarian syndrome (Pcos): an update. Arch Gynecol Obstet (2021) 303(3):631–43. doi: 10.1007/s00404-020-05951-2 33439300

[8] Damone AL Joham AE Loxton D Earnest A Teede HJ Moran LJ. Depression, anxiety and perceived stress in women with and without pcos: A community-based study. Psychol Med (2019) 49(9):1510–20. doi: 10.1017/s0033291718002076 30131078

[9] Revised 2003 consensus on diagnostic criteria and long-term health risks related to polycystic ovary syndrome (Pcos). Hum Reprod (2004) 19(1):41–7. doi: 10.1093/humrep/deh098 14688154

[10] Mousa A Joham A Boyle J. Polycystic Ovary Syndrome. Semin Reprod Med (2021) 39(3-04):69–70. doi: 10.1055/s-0041-1735506 34530478

[11] Azziz R Carmina E Dewailly D Diamanti-Kandarakis E Escobar-Morreale HF Futterweit W. Positions statement: criteria for defining polycystic ovary syndrome as a predominantly hyperandrogenic syndrome: an androgen excess society guideline. J Clin Endocrinol Metab (2006) 91(11):4237–45. doi: 10.1210/jc.2006-0178 16940456

[12] Yang S Ruan J Huang W Tan J Zhang X Feng X. [a pilot study of serum anti-mullerian hormone in diagnosis of polycystic ovary syndrome based on 2012 chinese polycystic ovary syndrome diagnosis criteria]. Zhonghua Fu Chan Ke Za Zhi (2015) 50(11):819–24. 26887768

[13] March WA Moore VM Willson KJ Phillips DI Norman RJ Davies MJ. The prevalence of polycystic ovary syndrome in a community sample assessed under contrasting diagnostic criteria. Hum Reprod (2010) 25(2):544–51. doi: 10.1093/humrep/dep399 19910321

[14] Chang RJ. A practical approach to the diagnosis of polycystic ovary syndrome. Am J Obstet Gynecol (2004) 191(3):713–7. doi: 10.1016/j.ajog.2004.04.045 15467530

[15] Leonhardt H Gull B Stener-Victorin E Hellström M. Ovarian volume and antral follicle count assessed by mri and transvaginal ultrasonography: A methodological study. Acta Radiol (2014) 55(2):248–56. doi: 10.1177/0284185113495835 23926234

[16] Teede HJ Tay CT Laven J Dokras A Moran LJ Piltonen TT. Recommendations from the 2023 international evidence-based guideline for the assessment and management of polycystic ovary syndrome. Fertil Steril (2023) 189(12):G43–64. doi: 10.1016/j.fertnstert.2023.07.025 37580861

[17] Li J Smith LS Zhu HJ. Data-independent acquisition (Dia): an emerging proteomics technology for analysis of drug-metabolizing enzymes and transporters. Drug Discovery Today Technol (2021) 39:49–56. doi: 10.1016/j.ddtec.2021.06.006 PMC8674493 34906325

[18] Ma J Liu M Wang Y Xin C Zhang H Chen S. Quantitative proteomics analysis of young and elderly skin with dia mass spectrometry reveals new skin aging-related proteins. Aging (Albany NY) (2020) 12(13):13529–54. doi: 10.18632/aging.103461 PMC7377841 32602849

[19] Yu Y Tan P Zhuang Z Wang Z Zhu L Qiu R. Dia proteomics analysis through serum profiles reveals the significant proteins as candidate biomarkers in women with pcos. BMC Med Genomics (2021) 14(1):125. doi: 10.1186/s12920-021-00962-7 33964924 PMC8106864

[20] Wang W Jiang Q Niu Y Ding Q Yang X Zheng Y. Proteomics and bioinformatics analysis of follicular fluid from patients with polycystic ovary syndrome. Front Mol Biosci (2022) 9:956406. doi: 10.3389/fmolb.2022.956406 36072434 PMC9441494

[21] Zhang J Ding N Xin W Yang X Wang F. Quantitative proteomics reveals that a prognostic signature of the endometrium of the polycystic ovary syndrome women based on ferroptosis proteins. Front Endocrinol (Lausanne) (2022) 13:871945. doi: 10.3389/fendo.2022.871945 35909514 PMC9330063

[22] Insenser M Escobar-Morreale HF. Proteomics and polycystic ovary syndrome. Expert Rev Proteomics (2013) 10(5):435–47. doi: 10.1586/14789450.2013.837665 24087928

[23] Loos G Van Schepdael A Cabooter D. Quantitative mass spectrometry methods for pharmaceutical analysis. Philos Trans A Math Phys Eng Sci (2016) 374(2079):20150366. doi: 10.1098/rsta.2015.0366 27644982 PMC5031633

[24] Xu X Jiang X Shi M Yin L. Mass spectrometry-based techniques for single-cell analysis. Analyst (2023) 148(16):3690–707. doi: 10.1039/d3an00370a 37458146

[25] Bajbouj K Al-Ali A Ramakrishnan RK Saber-Ayad M Hamid Q. Histone modification in nsclc: molecular mechanisms and therapeutic targets. Int J Mol Sci (2021) 22(21):11701. doi: 10.3390/ijms222111701 34769131 PMC8584007

[26] Ni L Lin B Zhang Y Hu L Lin J Fu F. Histone modification landscape and the key significance of H3k27me3 in myocardial ischaemia/reperfusion injury. Sci China Life Sci (2023) 66(6):1264–79. doi: 10.1007/s11427-022-2257-9 36808292

[27] Tong C Wu Y Zhang L Yu Y. Insulin resistance, autophagy and apoptosis in patients with polycystic ovary syndrome: association with pi3k signaling pathway. Front Endocrinol (Lausanne) (2022) 13:1091147. doi: 10.3389/fendo.2022.1091147 36589825 PMC9800521

[28] Vazquez BN Blengini CS Hernandez Y Serrano L Schindler K. Sirt7 promotes chromosome synapsis during prophase I of female meiosis. Chromosoma (2019) 128(3):369–83. doi: 10.1007/s00412-019-00713-9 PMC8494110 31256246

[29] Arroyo A Kim B Yeh J. Luteinizing hormone action in human oocyte maturation and quality: signaling pathways, regulation, and clinical impact. Reprod Sci (2020) 27(6):1223–52. doi: 10.1007/s43032-019-00137-x PMC7190682 32046451

[30] Eini F Novin MG Joharchi K Hosseini A Nazarian H Piryaei A. Intracytoplasmic oxidative stress reverses epigenetic modifications in polycystic ovary syndrome. Reprod Fertil Dev (2017) 29(12):2313–23. doi: 10.1071/rd16428 28442024

[31] Dower K Ellis DK Saraf K Jelinsky SA Lin LL. Innate immune responses to trem-1 activation: overlap, divergence, and positive and negative cross-talk with bacterial lipopolysaccharide. J Immunol (2008) 180(5):3520–34. doi: 10.4049/jimmunol.180.5.3520 18292579

[32] Kim EJ Choi MR Park H Kim M Hong JE Lee JY. Dietary fat increases solid tumor growth and metastasis of 4t1 murine mammary carcinoma cells and mortality in obesity-resistant balb/C mice. Breast Cancer Res (2011) 13(4):R78. doi: 10.1186/bcr2927 21834963 PMC3236342

[33] Ding Z Wang N Ji N Chen ZS. Proteomics technologies for cancer liquid biopsies. Mol Cancer (2022) 21(1):53. doi: 10.1186/s12943-022-01526-8 35168611 PMC8845389

[34] Monti C Zilocchi M Colugnat I Alberio T. Proteomics turns functional. J Proteomics (2019) 198:36–44. doi: 10.1016/j.jprot.2018.12.012 30553948

[35] Gennarelli G Holte J Berglund L Berne C Massobrio M Lithell H. Prediction models for insulin resistance in the polycystic ovary syndrome. Hum Reprod (2000) 15(10):2098–102. doi: 10.1093/humrep/15.10.2098 11006180

