# Supplementary material for: Combining proteomic markers to construct a logistic regression model for polycystic ovary syndrome
Source: Front Endocrinol (Lausanne). 2023 Oct 3;14:1227252. doi: 10.3389/fendo.2023.1227252 (PMC10579946; doi:10.3389/fendo.2023.1227252)
Supplement: Supplementary file 1 [file DataSheet_1.pdf]

ProteinAccessions	Genes	ProteinDescriptions	MolecularWeight	Average Intensity	Con_1	Con_2	Con_3	Con_4	Con_5	Con_6	Con_7
P02768	ALB	Serum albumin	69366.68	1.18E+08	79957808	84716104	70466528	117885024	63286400	88281888	75276680
P02647	APOA1	Apolipoprotein A-I	30777.83	1.01E+08	121711480	122049936	111401896	104138760	80962544	96620976	104226288
P02790	HPX	Hemopexin	51676.37	3.19E+07	37069120	32072526	43862344	35318612	25985996	39360752	30934850
P02765	AHSG	Alpha-2-HS-glycoprot	39324.67	2.55E+07	21214592	23649384	33299814	25571360	17146968	23629876	29199138
P02774	GC	Vitamin D-binding pr	52963.65	2.00E+07	26825784	23187422	24325444	23902864	18593404	20736040	22942660
P04004	VTN	Vitronectin	54305.59	1.96E+07	18124512	13974569	23048888	21740996	14152054	21241618	19804262
P01024	C3	Complement C3	187148.06	1.71E+07	14137431	14205530	21083510	16939870	14631717	17767246	16560443
P02652	APOA2	Apolipoprotein A-II	11175.01	1.56E+07	16373083	16929670	19127870	18067224	12784090	13923255	16797496
P01011	SERPINA3	Alpha-1-antichymotry	47650.86	1.39E+07	14488383	13926978	17872274	15290806	10831234	12753978	13281332
P04217	A1BG	Alpha-1B-glycoprotei	54253.51	1.35E+07	15443014	12684926	17489844	18223320	11192193	15879364	12210324
Q13595	TRA2A	Transformer-2 protei	32688.62	1.08E+07	10993255	11241172	14348038	12752825	6912998.5	8964386	9578540
Q86UX2	ITIH5	Inter-alpha-trypsin	104576.41	1.06E+07	10492943	12056006	12518754	6117737.5	7790667.5	6919142	11192650
P01042	KNG1	Kininogen-1	71957.37	1.02E+07	13185338	10659745	12576008	12792298	8995657	11741196	10813451
P04003	C4BPA	C4b-binding protein	67033.18	9.33E+06	9689172	9130774	14142670	8776505	8303370	9549940	9336461
P0C0L4	C4A	Complement C4-A	192785.48	9.03E+06	7149540.5	7455286.5	12272386	5149004.5	9069657	10798348	8102474.5
P02766	TTR	Transthyretin	15887.02	9.02E+06	10861628	10635098	8889366	10926525	9596504	10996910	10386469
P19823	ITIH2	Inter-alpha-trypsin	106463.47	8.82E+06	9532312	9931188	9054136	10180781	7302500	10490519	10514532
P08603	CFH	Complement factor H	139096.31	8.52E+06	8724936	8632103	12306215	9111656	7255333.5	9333787	8556446
O95155	UBE4B	Ubiquitin conjugatio	146184.8	8.45E+06	8000144	8511411	13230606	8551815	7433780	8705271	7836317.5
C9JQL5;Q01628	;IFITM3	Putative dispanin su	14796.48;14632.09	8.30E+06	8184504.5	8892888	10334148	17188868	8190003.5	13143265	14140610
P04114	APOB	Apolipoprotein B-100	515604.72	7.35E+06	8535656	7060226	9108860	6851307	3617785.5	8374734	5713273
P00747	PLG	Plasminogen	90568.98	7.29E+06	7859164.5	9081335	9577786	10247970	6527036.5	8507359	8508558
Q8TF62	ATP8B4	Probable phospholipi	135868.18	7.05E+06	3421961	4914638	8637743	4051114.25	6978363	8728187	6221762
Q5T5C0	STXBP5	Syntaxin-binding pro	127572.56	6.80E+06	4797204	5863372	8205316.5	5775224.5	4852970.5	6125004.5	5444180.5
P05155	SERPING1	Plasma protease C1 i	55154.18	6.70E+06	9916344	3516391.5	3549598.75	7058813	6901140	6508012	7398796
Q14624	ITIH4	Inter-alpha-trypsin	103357.42	6.67E+06	7142331.5	6636002	8345138	7520836	6118830	6946622.5	6980320
P02749	APOH	Beta-2-glycoprotein	38298.15	6.22E+06	6792522	6187692	5782349	7126821.5	5360822	8525214	6666899
Q8IUB2	WFDC3	WAP four-disulfide c	24686.98	5.76E+06	6133734	5957154	7898975	5743546	4700113.5	6641174	5708637.5
P02751	FN1	Fibronectin	262624.55	5.69E+06	4768160	4280319	6410563.5	6691896	4026499	5810951.5	6253206
P06727	APOA4	Apolipoprotein A-IV	45399.06	5.13E+06	8110090.5	3554352	8178487.5	5233738	5049846	5501992	6672546
P02656	APOC3	Apolipoprotein C-III	10852.3	5.11E+06	6672787.5	5016306	5999818.5	4888729.5	1789822.625	5471448	3669850
P05546	SERPIND1	Heparin cofactor 2	57070.6	5.06E+06	4480433	5427110	6032254	4864996	4740363	4759558	4921569
P19827	ITIH1	Inter-alpha-trypsin	101389.12	4.89E+06	4818919	4895898	5924488.5	6097778.5	4146657.75	6251638.5	5443429
P02775	PPBP	Platelet basic prote	13894.21	4.68E+06	4018912.25	4640180.5	5777010.5	4405186	3980527.25	5189567	4951711
P04196	HRG	Histidine-rich glyco	59578.32	4.55E+06	9523663	8267555.5	5087873	6171376.5	4435332.5	4425607.5	5885581.5
P55786	NPEPPS	Puromycin-sensitive	103276.31	3.90E+06	2202156.5	2822425	2112516.25	3777405	1104437.5	1635933	2345068.75
P06396	GSN	Gelsolin	85697.51	3.84E+06	4683195	4295582	5141062	3460535.75	4720999	3470178.5	6005898.5
P02649	APOE	Apolipoprotein E	36154.08	3.83E+06	4512273	4575501	5254675	4515819.5	3241785.5	3055567	2609604.25
P00734	F2	Prothrombin	70036.86	3.65E+06	3631887	4004492.75	4978208.5	4674770	3417079.5	4246747	4054824.75
Q6P179	ERAP2	Endoplasmic reticulu	110461.69	3.58E+06	9934.717773	26808096	13365.49805	1743.328369	974.9887695	12092.93066	Filtered
Q86WI1	PKHD1L1	Fibrocystin-L	465734.36	3.51E+06	2798141.5	5324837.5	2886967.5	3170185	Filtered	1776214	5392047
P02654	APOC1	Apolipoprotein C-I	9331.92	3.45E+06	3702183.5	3854939.75	4953492	4746784	2372821.75	2967150	2526915
P00450	CP	Ceruloplasmin	122205.18	3.36E+06	2807883.25	3799397.5	5222159	3541635.5	3248826.5	3249552.25	3242365
Q03591	CFHR1	Complement factor H-	37650.56	3.35E+06	1790933.25	4260759.5	6186639	4613140.5	4076618.25	5071635.5	4315191
P10909	CLU	Clusterin	52494.57	3.04E+06	3775572.75	3174393.75	3265841.75	3731310.25	2501455.25	3458920.75	3209023.75
P15586	GNS	N-acetylglucosamine-	62082.09	3.02E+06	4199568.5	2331739.5	1005680.75	4132850.75	2747355.5	3595650.25	2997144.25
P01008	SERPINC1	Antithrombin-III	52602.43	2.95E+06	4670856.5	3154718.25	2348954	4186895.25	3112072.25	3498565.25	3491264.75
P01009	SERPINA1	Alpha-1-antitrypsin	46736.55	2.88E+06	3193098	5629100	8591792	5043423.5	1050649.75	1939886.125	1743049.375
P02776	PF4	Platelet factor 4	10844.94	2.78E+06	2645606.75	2353954.25	4358865.5	2457325.5	2741752	2882099	2709857.5
P00751	CFB	Complement factor B	85532.87	2.72E+06	2332944	1926124.5	3462217.75	3365304	2382891.75	2671606.75	2263460
P08697	SERPINF2	Alpha-2-antiplasmin	54565.76	2.64E+06	2760158.5	2706909.25	2344510.25	2939539.25	2254222	2934883.75	2719615.5
P35542	SAA4	Serum amyloid A-4 pr	14746.65	2.55E+06	2504241.25	2734239.25	3155341	2339878.5	1724016	2329764.5	2200094.25
P27169	PON1	Serum paraoxonase/ar	39731.31	2.50E+06	3455279.25	3073647.5	3484785.25	2946228	2201425	2704171.75	3064826.75
P01023	A2M	Alpha-2-macroglobuli	163290.99	2.41E+06	976288.5625	1492647.25	1970678.625	1054161	1101179.625	798750.25	1270219
P02747	C1QC	Complement C1q subco	25773.64	2.34E+06	2158478	2625286.25	3467717.25	2630140.25	2429164.75	3284394.5	2567267
P43652	AFM	Afamin	69069.11	2.20E+06	1960758.25	2424860	2104459.5	2566789.75	1596948.375	2444867.75	2908074.75
P01031	C5	Complement C5	188305.29	2.11E+06	2421510.75	2153972.75	2475678	2378854.5	1569968.125	2071031.125	1984513.375
P07225	PROS1	Vitamin K-dependent	75122.59	2.06E+06	2561074.25	2248176.5	3215358	2204552.5	1760328.25	2349166.5	2077936.25
Q96PD5	PGLYRP2	N-acetylmuramoyl-L-a	62217.01	2.06E+06	2121077.25	2220320	2007065.25	2838514	1934061.125	2789233.5	2553528.25
P02748	C9	Complement component	63173.37	1.99E+06	2221981.5	2176830	3017601.75	2570976	1744396	2077655.875	1820897
P43405	SYK	Tyrosine-protein kin	72066.2	1.99E+06	1828954.75	1973694.125	2460866.75	1731092.375	870934.75	2166794.25	1582774.75
P02787	TF	Serotransferrin	77063.89	1.99E+06	1779523.875	2435630.25	1909915.375	1883582.375	1446952.125	1258678.875	1059013.875
P09871	C1S	Complement C1s subco	76684.43	1.98E+06	2511024.75	2120507.5	2800888.5	2080621.125	1840237.875	2379721.25	2019818.75
P01871	IGHM	Ig mu chain C region	49306.59	1.93E+06	221749.4531	643809.875	374472.3125	1422229.625	571297.8125	402031.4688	623837.5625
O15195	VILL	Villin-like protein	95907.4	1.87E+06	Filtered	Filtered	Filtered	Filtered	Filtered	Filtered	1981810.75
P43629;Q14943	KIR3DL1;KIR3DS1	Killer cell immunogl	49097.96;42902.02	1.86E+06	931134.9375	936218.8125	2071307.875	774670.1875	2282259.75	1544552.125	2273888.75
Q9UPN4	CEP131	Centrosomal protein	122148.74	1.84E+06	1706298.625	1645825.5	2107359	2044197.625	1560630.125	1832302.5	1906906.25
P51884	LUM	Lumican	38429.01	1.83E+06	2839220.75	1988874.5	2291069	1760966.625	2571840.75	2547945	2346566.25
P08185	SERPINA6	Corticosteroid-bindi	45140.87	1.78E+06	2304728.25	2954976.75	829566.1875	1016188.25	1753548.75	711015.625	1994504.375
P02750	LRG1	Leucine-rich alpha-2	38177.91	1.72E+06	1989532.375	1497881.375	2661692.75	1943687.125	1651432.875	1765471.125	1475338.5
P01019	AGT	Angiotensinogen	53154.19	1.68E+06	1400136.5	1230304.25	1983700.375	1611109.25	1325234	2198421.25	2108604
P02753	RBP4	Retinol-binding prot	23010	1.68E+06	1404160.75	3130403.5	2437608.5	2328935.75	2204246	2335494.25	2732457.5
P13671	C6	Complement component	104786.06	1.64E+06	2599872.75	1959302.375	2331555.25	2203249.75	1641361.25	1638126	1934075.375

P02655	APOC2	Apolipoprotein C-II	11283.86	1.60E+06	1549984.125	1493807.25	1792265.25	1742024.25	761025.875	1325639.875	794269.25
P00738	HP	Haptoglobin	45205.3	1.58E+06	149828.7813	305590.7188	322332.625	262904.25	308119.25	260218.3438	333711.625
P03952	KLKB1	Plasma kallikrein	71369.69	1.54E+06	1841076.25	1737150.625	1794503.375	1536537.625	1355847.25	1689298	1468205.375
P00748	F12	Coagulation factor X	67792.09	1.54E+06	1023522.563	1172098.875	3415925.5	1285803.625	899980.375	1965044.75	2146639.5
P02746	C1QB	Complement C1q subco	26721.75	1.52E+06	1412226.75	1366042.75	1738770.75	1612549.625	1219067	1778118.75	1464293.25
P29590	PML	Protein PML	97550.58	1.47E+06	844276.3125	1226354.75	1100508.75	Filtered	76771.10938	1290938.5	1169316.25
P00736	C1R	Complement C1r subco	80118.54	1.43E+06	1734020.125	1539368.25	1951216.875	1557473.875	1163159.625	1811404	1403074.875
O60828	PQBP1	Polyglutamine-bindin	30472.25	1.39E+06	2009913.5	1008857	3339054	1477117.25	1191003	1478158	1591503.5
P05388;Q8NHW5	RPLP0;RPLP0P6	60S acidic ribosomal	34273.5;34364.48	1.37E+06	88810.94531	2053460.25	2101534	1564938.5	114863.8594	1281179.125	1523887.25
P22792	CPN2	Carboxypeptidase N s	60556.64	1.36E+06	1048666.75	1318095.625	1872799.5	1719856.375	1103221	1483238.875	1725035.75
O75636	FCN3	Ficolin-3	32902.98	1.34E+06	1800438	914396.75	1497933.75	1457775	1156462.5	1204116.25	1661422.375
P01877	IGHA2	Ig alpha-2 chain C r	36526.31	1.33E+06	682999.6875	746267.5	534510	587826.8125	462917.6563	155434.6719	1132246.5
P29622	SERPINA4	Kallistatin	48541.96	1.26E+06	1475844.75	1396909.375	1251565.125	1504552.25	1181226.75	1421873.375	1561603.125
P10643	C7	Complement component	93518.23	1.24E+06	1513237.125	1637624.625	1163577.5	2154861	1139536.5	1330008	1911033.875
O95445	APOM	Apolipoprotein M	21253.29	1.18E+06	1220821.75	1217403.5	1638056.375	1287407.625	879894.0625	1126516.25	1179813.625
Q9H3P2	NELFA	Negative elongation	57276.8	1.17E+06	1158672	1119089.875	1423708.125	950840.125	860124.3125	1093257.25	1184808.25
P36955	SERPINF1	Pigment epithelium-d	46312.2	1.15E+06	1061858.625	739945.125	1565896.25	1181579.5	976188	1626284.5	1444009.125
P05156	CFI	Complement factor I	65750.25	1.14E+06	700995.0625	1216438.625	1791554.375	1209421.125	1016460.813	1303296.5	1138811
P07477	PRSS1	Trypsin-1	26558.08	1.13E+06	844948.3125	1190245.125	568812.625	569611.1875	634303.875	312004.7813	378975.4375
P23435	CBLN1	Cerebellin-1	21097.17	1.13E+06	1695885.875	1600443.125	1783873.125	1196208.375	1177314.25	1690019.25	1325369.25
Q66K66	TMEM198	Transmembrane protei	39474.72	1.13E+06	241004.9844	1947734.375	17146.58594	2475419.5	817720.125	1706897.25	15946.63281
P35858	IGFALS	Insulin-like growth	66035	1.09E+06	1188744.5	1144220.125	968674.6875	1399063.375	800108.4375	1167887.25	1260176
P69905	HBA1	Hemoglobin subunit a	15257.54	1.05E+06	79405.42188	62338.05078	45604.33984	119620.5859	218431.625	67052.05469	98892.94531
Q06033	ITIH3	Inter-alpha-trypsin	99849.23	1.04E+06	1040328.188	919058.75	1661463.5	1147230.5	876530	1173807.125	1308496.25
P07360	C8G	Complement component	22277.46	1.02E+06	917370.125	841799.625	1253115.75	1282449.375	790716	916663.3125	1037788.313
P07358	C8B	Complement component	67046.87	9.35E+05	928456.5625	862205	1180950.875	1102082	661541.375	711624	923974.8125
Q9C0B1	FTO	Alpha-ketoglutarate-	58282.04	9.23E+05	394809.3125	494476.7188	440226.0313	878526.5625	1133069.875	695489.4375	529727.375
Q14118	DAG1	Dystroglycan	97441.49	9.09E+05	449952.1563	478543.6563	1125929.375	920146.375	800059	970429.1875	655994.25
P06681	C2	Complement C2	83267.83	9.02E+05	1210556.125	869149.5	1143665.5	937633.1875	820137.4375	961793.875	1013914.375
P01876	IGHA1	Ig alpha-1 chain C r	37654.64	8.73E+05	266591.4688	577923.4375	262547.5625	256579.1563	175774.125	88887.07813	758176.5
P05452	CLEC3B	Tetranectin	22536.81	8.41E+05	890443.25	1307011.75	836360.9375	787104.875	1048102.375	860222.5625	1515616
P23142	FBLN1	Fibulin-1	77214.05	8.10E+05	816201.125	823321.0625	905972.125	901786.875	865326	1078127.375	1290223.875
Q08188	TGM3	Protein-glutamine ga	76631.83	7.82E+05	Filtered	872064.125	968965.0625	8154.057617	Filtered	924284.0625	724287.3125
P69891	HBG1	Hemoglobin subunit g	16140.46	7.59E+05	239543.8125	152940.4219	104218.4531	304215.8125	870373.0625	190067.7031	305171.0938
P39059	COL15A1	Collagen alpha-1(XV)	141720.14	7.40E+05	963805.3125	768831.875	931950.625	1031697.063	831377.3125	838520.375	929636.75
P02745	C1QA	Complement C1q subco	26016.56	7.19E+05	1004960.5	981222.0625	1087341.625	734763.5	723734.75	940369.0625	831801.9375
Q96IY4	CPB2	Carboxypeptidase B2	48424.25	6.56E+05	940030.8125	729465.1875	741002.125	826316.4375	614321	705248.3125	775340.125
P68871	HBB	Hemoglobin subunit b	15998.4	6.46E+05	48488.91406	39227.625	30805.68359	61421.63281	51787.0625	40297.18359	49414.58203
P07357	C8A	Complement component	65163.2	6.24E+05	648436.25	618204.125	918383.3125	767682.375	488862.2813	521042.25	703300.375
Q8IVT5	KSR1	Kinase suppressor of	102159.96	6.16E+05	351568.75	449816.6563	669064.8125	530126.75	293939.0625	420912.9063	541078.3125
P20851	C4BPB	C4b-binding protein	28357.44	5.94E+05	597124.5	929063.625	943917.9375	553999.0625	643442.375	575184.5625	815368.25
Q14520	HABP2	Hyaluronan-binding p	62671.7	5.85E+05	578554.75	721351.375	779256.125	699412.9375	518599.75	632228.8125	695841.375
P17936	IGFBP3	Insulin-like growth	31674.2	5.84E+05	724705.5	697944.5625	514196.7813	698064.375	490127.375	630841.25	719196.125
P0CG05	IGLC2	Ig lambda-2 chain C	11293.56	5.68E+05	148324.7969	156653.4531	140931.7344	144039.9531	181316.9063	90018.17188	205491.6563
P04220		Ig mu heavy chain di	43057.31	5.67E+05	835071.3125	560529.625	59033.85156	704189.6875	575443	618108.1875	81714.95313
P06276	BCHE	Cholinesterase	68418.05	5.64E+05	553175.25	565743.125	654593.5625	613322.3125	523286.2813	531155.125	534768.125
P01859	IGHG2	Ig gamma-2 chain C r	35900.64	5.62E+05	271459.6563	142173.7656	165226.5625	109163.5625	185344.2656	128341.1094	313675.25
P01834	IGKC	Ig kappa chain C reg	11608.85	5.33E+05	327007.1875	250312.8125	161373.6406	108001.9688	124077.5391	124778.5781	310109.5313
P07996	THBS1	Thrombospondin-1	129382.66	5.27E+05	438267.9375	498903.2188	700337.4375	506865.1563	513997.9063	602677.9375	612221.0625
P01857	IGHG1	Ig gamma-1 chain C r	36105.91	5.08E+05	121540.5938	100023.9297	94793.19531	47291.69531	104826.8984	66220.04688	186020.6875
P05160	F13B	Coagulation factor X	75510.67	5.01E+05	644665.9375	613440.5625	703143.9375	471695.5625	427891.25	510360.8125	524143.375
P02760	AMBP	Protein AMBP	38999.47	4.99E+05	541002.375	426996.6875	629661.75	445902.25	367362.375	540082.1875	496043.125
Q99417	MYCBP	C-Myc-binding protei	11966.68	4.82E+05	589398.5625	616091.625	835509.8125	416096.75	Filtered	813051	600209.0625
P80108	GPLD1	Phosphatidylinositol	92336.44	4.79E+05	469428.4688	319001.7188	640980.5625	680307	353543.2188	335947.4375	465520.75
P07333	CSF1R	Macrophage colony-st	107983.75	4.73E+05	912079.75	60716.11719	61490.71875	616572.8125	42666.34375	46326.34375	51242.57813
P49750	YLPM1	YLP motif-containing	219985.15	4.68E+05	328203.9375	448747.0938	840115.375	357354.5	Filtered	751109.25	476508.0625
O43157	PLXNB1	Plexin-B1	232297.66	4.58E+05	384188.0938	423130.4063	353885.75	348704.8438	277776.5938	280511.0625	362281.3125
O75882	ATRN	Attractin	158536.87	4.55E+05	662845.375	565229	583531.25	572464.4375	466987.1563	541370.6875	505293.875
Q86XA9;Q9P2D3	HEATR5A;HEATR5B	HEAT repeat-containi	222003.95;224302.39	4.31E+05	425326.4063	420373	530098.8125	504739.5625	315968.1563	452047.4375	384363
Q9BYJ0	FGFBP2	Fibroblast growth fa	24581.03	4.30E+05	379280.6563	318116.8438	13181.64746	670463.125	415917.6875	11939.5625	15140.26465
P01344	IGF2	Insulin-like growth	20140.34	4.29E+05	424784.5313	796827.375	673355.6875	392586.5	324872.7188	368356.8125	775160
P02671	FGA	Fibrinogen alpha cha	94973.03	4.24E+05	296989.3125	465761.8438	462712.0625	389604.125	329100.5	444432.4375	626089.25
P51812	RPS6KA3	Ribosomal protein S6	83735.98	4.07E+05	360109.1563	285620.625	351918.125	316376.8438	112429.4844	208919.0313	231396.125
P49908	SEPP1	Selenoprotein P	43173.64	4.06E+05	567822.125	520080.8438	486001.8125	380364.4688	339681.9375	382941.625	468355.4063
P31025	LCN1	Lipocalin-1	19250.01	4.04E+05	184688.7813	1540.429199	28158.60547	2496.479492	Filtered	Filtered	Filtered
Q9UGM5	FETUB	Fetuin-B	42054.94	3.75E+05	413664.5625	343480.9375	485569.5313	439369.2813	330808.6875	409410.75	463217.2813
P26927	MST1	Hepatocyte growth fa	80319.87	3.73E+05	490607.5625	294505.4375	512313.9063	488122.7813	371363.75	445215.9688	437262.4375
P02788	LTF	Lactotransferrin	78181.95	3.69E+05	205748.8438	119052.2578	88185.66406	142901.9063	8262571.5	187426	201079.0938
P05090	APOD	Apolipoprotein D	21275.55	3.56E+05	1999425.875	1033798.5	603121.4375	533524.4375	379392.125	373734.8438	533495.5
O00187	MASP2	Mannan-binding lecti	75702.36	3.55E+05	408427.375	294353.875	439660.75	377071.125	255882.5469	318718.6563	398569.3125
P0C0L5	C4B	Complement C4-B	192751.45	3.50E+05	165188.7656	253760.625	388037.625	242180.9375	512659.9375	493655.0313	390384.2188
P51693	APLP1	Amyloid-like protein	72176.39	3.40E+05	377436.1875	354172.8125	326480.6563	500398.0938	313897.0313	475633.4688	432419.0313
Q96KN2	CNDP1	Beta-Ala-His dipepti	56706.08	3.34E+05	497219.1563	355545.4375	658425.5	373522.8438	365037.8438	363346.0625	392389.5313
Q04695	KRT17	Keratin, type I cyto	48105.66	3.33E+05	293514.5938	Filtered	Filtered	Filtered	314904.3438	686668.9375	Filtered
P15169	CPN1	Carboxypeptidase N c	52286.23	3.28E+05	283777.5625	303271.9063	380209.3125	400145.1875	247699.9844	313257.5938	408483.9688

P20742	PZP	Pregnancy zone prote	163862.95	3.21E+05	324372.5313	46994.23828	388256.3438	20480.17773	921690.5625	464711.5938	36053.90234
O43920	NDUFS5	NADH dehydrogenase [	12517.5	3.19E+05	Filtered	457768.5313	89285.4375	55031.51953	357158.9063	140593	Filtered
P14151	SELL	L-selectin	42187.08	3.15E+05	274668.7188	403645.75	245159.25	357058.5	302675.4063	392293.9063	396866.5625
O43866	CD5L	CD5 antigen-like	38087.79	3.14E+05	189291.9844	273821.6563	161375.9688	288838.375	162869.125	249384.375	267366.9375
P04259	KRT6B	Keratin, type II cyt	60066.95	3.10E+05	290470.9375	132874.5156	542574.0625	205357.1875	54448.80469	335626.8125	169124.0313
O14960	LECT2	Leukocyte cell-deriv	16389.96	2.95E+05	130636.0391	108834.0547	13878.78027	9495.101563	Filtered	80008.21875	95124.30469
P01621		Ig kappa chain V-III	10728.87	2.83E+05	183018.6406	141192.1875	92458.60156	74466.05469	108972.0781	54622.76172	222015.125
P06576	ATP5B	ATP synthase subunit	56559.9	2.82E+05	Filtered	5689.15625	4701.683105	Filtered	5094.459473	6044.71582	12028.04688
P48637	GSS	Glutathione syntheta	52384.82	2.80E+05	11417.46094	Filtered	Filtered	Filtered	Filtered	Filtered	Filtered
P04070	PROC	Vitamin K-dependent	52071.27	2.74E+05	373360.6875	290146.0625	331499.4375	358739.2813	212024.2656	290096.9688	274411.0625
Q16610	ECM1	Extracellular matrix	60674.09	2.73E+05	361308.875	263236.625	281703.75	230191.5781	211796.4219	290519.5	365083.3438
P55056	APOC4	Apolipoprotein C-IV	14553.04	2.69E+05	426614.5625	335457.9688	255907.8594	202207.9063	95036.98438	234577.2031	180679.7188
P00740	F9	Coagulation factor I	51778.43	2.66E+05	267032.2188	257322.8594	160023.2813	307040.8438	273236.375	354288.5938	222049.3438
P47929	LGALS7	Galectin-7	15075.05	2.66E+05	262922.875	171156.5781	451369.0313	262289.75	298130.9063	203938.7813	542760.125
P01765		Ig heavy chain V-III	NaN	2.58E+05	198072.5313	190743.1094	241236.7813	230538.0938	114136.3281	303182.8438	142202.8594
P04207		Ig kappa chain V-III	14275.17	2.55E+05	183156.6875	141149.7344	91655.53906	74270.73438	108781.375	54542.86328	222015.125
P12259	F5	Coagulation factor V	251703.42	2.52E+05	297359.25	242187.75	268393.875	274834.375	243314.8438	223750.8438	297365.4688
P04264	KRT1	Keratin, type II cyt	66038.73	2.52E+05	247245.0469	127797.0703	429447.4375	887324.875	89244.80469	202794.0938	99619.97656
Q13790	APOF	Apolipoprotein F	35399.49	2.49E+05	303386.1875	330909	224794.6875	361532.75	138383.2656	209075.0625	235987.0938
Q9UK55	SERPINA10	Protein Z-dependent	50706.95	2.47E+05	285410	158899.7656	233032.0781	250352.375	171575	288605.0938	254376.6719
P27918	CFP	Properdin	51276.37	2.46E+05	195237.9375	345166.9063	346466.6875	235495	236045.0781	378647.4375	253032.5313
P01860	IGHG3	Ig gamma-3 chain C r	41286.95	2.41E+05	19573.54102	19654.33984	59139.87109	22045.24219	56770.14844	42342.08594	71518.73438
O14791	APOL1	Apolipoprotein L1	43974.2	2.39E+05	195986.5781	215079.6406	207737.3281	153980.625	77073.26563	197243.8281	315252.6875
P00742	F10	Coagulation factor X	54731.69	2.38E+05	205523.6094	204053.5625	257021.2344	311181.875	192966.5938	241084.2344	238366.4375
P03951	F11	Coagulation factor X	70109.12	2.37E+05	173444.7031	207022.7656	229739.4219	160385.4063	146722.8594	166308.5469	166883.4688
Q8N812	C12orf76	Uncharacterized prot	14959.12	2.37E+05	Filtered	Filtered	Filtered	Filtered	Filtered	Filtered	Filtered
P05543	SERPINA7	Thyroxine-binding gl	46324.5	2.34E+05	290516.0313	299032.125	205242.8438	299786.125	146852.0313	345559.1875	247842.7969
P11226	MBL2	Mannose-binding prot	26143.53	2.33E+05	38080.88281	408306.25	410978.5313	78663.20313	125350.625	370441.8438	209047.9219
Q9NZP8	C1RL	Complement C1r subco	53498.34	2.32E+05	280837.9375	222540.4531	264295.8125	271090.3125	191789.5469	259178.2813	256325.7969
Q9Y5C1	ANGPTL3	Angiopoietin-related	53637.46	2.32E+05	256947.8125	237446.6406	301609	199413.5	107518.3203	241263.4844	162564.375
P25311	AZGP1	Zinc-alpha-2-glycopr	34258.71	2.32E+05	1177424.75	347717.75	290579.7813	252114.5156	182767.3594	292852.5	223445.6406
P04433		Ig kappa chain V-III	12575.18	2.21E+05	129055.9063	127906.3359	47575.82031	53605.07031	49798.69141	38587.93359	120570.0156
P00746	CFD	Complement factor D	27032.93	2.16E+05	154577.3125	224718.7344	189303	213875.9063	197597.7344	265157.9375	221989.0781
Q01082	SPTBN1	Spectrin beta chain,	274609.22	2.15E+05	213619.7969	175879.0313	249300.5938	255597.0469	227072.3125	163156.5469	275082.9688
P01764	IGHV3-23	Ig heavy chain V-III	12582.31	2.09E+05	111040.0156	65960.36719	110963.5625	68492.36719	128693.0859	78829.51563	121921.2969
P16070	CD44	CD44 antigen	81537.62	2.09E+05	222124.7188	252300.1719	190451.9063	229240.1875	188004.375	219846.3281	220609.3438
Q04756	HGFAC	Hepatocyte growth fa	70681.83	2.05E+05	300703.0313	176774.6406	184692.9063	261180.5313	230887.9375	207832.0625	252303.7188
O76009;Q15323	KRT33A;KRT31	Keratin, type I cuti	45940.34;47237.05	2.04E+05	Filtered	578376.875	Filtered	Filtered	Filtered	Filtered	Filtered
P18135		Ig kappa chain V-III	14072.89	2.03E+05	108214.2188	72564.41406	65584.625	48308.89063	47866.78516	34960.88672	120618.875
P04180	LCAT	Phosphatidylcholine-	49577.92	1.97E+05	197138.375	199550.4688	273997.625	212156.1875	146075.1719	212453.6875	190546.9688
P22891	PROZ	Vitamin K-dependent	44743.89	1.97E+05	187221.0156	132387.4063	260527.1563	184183.7188	116054.7656	222650.9375	214413.8594
P12829	MYL4	Myosin light chain 4	21564.6	1.97E+05	Filtered	Filtered	Filtered	Filtered	Filtered	Filtered	Filtered
P43251	BTD	Biotinidase	61132.91	1.87E+05	210983.7344	195462.3281	179986.6406	220588.625	172537.4531	196381.3125	235279.4375
O15304	SIVA1	Apoptosis regulatory	18694.64	1.81E+05	Filtered	Filtered	117337.6797	47724.47266	Filtered	Filtered	Filtered
Q5VU43	PDE4DIP	Myomegalin	265079.92	1.75E+05	249108.7656	170479.4063	313728.375	118761.3438	194737.8594	205406.9688	161372.5
Q6UVK1	CSPG4	Chondroitin sulfate	250536.77	1.72E+05	2693.209717	71590.74219	219191.25	103049.0547	60414.92188	152992.1563	174535.375
P18428	LBP	Lipopolysaccharide-b	53383.62	1.72E+05	130434.9766	135826.4375	294454.125	121807.1328	143161.0313	182571.9531	124829.3516
Q12805	EFEMP1	EGF-containing fibul	54640.54	1.72E+05	199596.6563	182021.125	206201.9844	90604.53125	152927.7031	195204.1563	219132.8125
P04275	VWF	von Willebrand facto	309264.51	1.72E+05	184932.4688	143052.8125	191671.7813	197626.8594	83286.95313	202450.0469	130625.8984
Q7Z794	KRT77	Keratin, type II cyt	61901.41	1.70E+05	145961.4844	67043.41406	269425.3438	8946.048828	51416.71484	168446.75	84552.39844
P08571	CD14	Monocyte differentia	40076.2	1.65E+05	254712.5938	148849.2656	231554.3594	186295.1875	132139.4688	210737.3281	185432.1719
P06309		Ig kappa chain V-II	12664.16	1.65E+05	151242.125	91425.08594	102577.2813	69154.8125	15181.70508	67013.61719	124258.8125
Q8N1N4	KRT78	Keratin, type II cyt	56865.82	1.62E+05	150749.7031	56714.41406	317864.625	220336.4219	48905.63672	159277.625	41454.35938
Q60240	PLIN1	Perilipin-1	55990.36	1.61E+05	Filtered	Filtered	Filtered	Filtered	Filtered	Filtered	Filtered
P36980	CFHR2	Complement factor H-	30650.73	1.59E+05	137949.5625	245443.2969	132558.3438	94874.14063	101875.4922	156844.9531	120309.6328
P26358	DNMT1	DNA (cytosine-5)-met	183165.22	1.57E+05	178518.7656	170873.5781	211570.5156	175081.375	125269.8594	160244.4531	167259.8281
O95835	LATS1	Serine/threonine-pro	126869.65	1.57E+05	Filtered	198338.7188	425449.5313	94224.23438	Filtered	Filtered	77398.74219
P01769		Ig heavy chain V-III	NaN	1.56E+05	17307.01172	39812	Filtered	Filtered	46269.46875	Filtered	30393.38672
P12955	PEPD	Xaa-Pro dipeptidase	54548.17	1.52E+05	42391.95703	31908.61719	28832.48047	25306.04883	14282.78613	27284.51563	28113.10352
P01766		Ig heavy chain V-III	13226.75	1.51E+05	77482.33594	42261.14844	50962.33984	29349.04102	56355.35938	25658.95703	60608.10547
P0DJI8	SAA1	Serum amyloid A-1 pr	1.35E+04	1.50E+05	1.56E+05	6.36E+04	3.99E+05	2.36E+05	5.24E+04	9.48E+04	6.09E+04
P48740	MASP1	Mannan-binding lecti	79246.67	1.50E+05	184955.4063	162597.7188	233205.5156	165920.5	123346.0703	140662.2969	174253.8438
P01781		Ig heavy chain V-III	NaN	1.48E+05	106834.4297	25868.93359	97827.39063	92646.00781	154282.8125	75629.49219	139155.9375
P54802	NAGLU	Alpha-N-acetylglucos	82265.69	1.46E+05	317208.8125	250404.7969	7024.45752	121205.7344	56604.08594	131812.25	63018.0625
P02743	APCS	Serum amyloid P-comp	25387.12	1.44E+05	84909.00781	58538.33984	261576.1719	102745.5703	117449.2109	114480.2422	123517.375
Q8WZ42	TTN	Titin	3816030.05	1.40E+05	102837.9688	206882.7813	160462.3281	191874.5938	96907.23438	86385.25	180750.7656
Q08380	LGALS3BP	Galectin-3-binding p	65331	1.37E+05	173568.1719	211807.0938	277942.375	198027.7031	119345.2344	124794.6172	171553.9219
P19652	ORM2	Alpha-1-acid glycopr	23602.62	1.35E+05	221407.0313	106944.9141	165383.3438	102711.1797	211991.5313	106499.2578	129954.7578
P35555	FBN1	Fibrillin-1	312237.48	1.33E+05	285621.0313	245181.7188	45140.73828	222742.3125	428059.3125	21444.9082	316894.6875
Q8N0X4	CLYBL	Citrate lyase subuni	37359.4	1.33E+05	Filtered	Filtered	Filtered	56493.95703	Filtered	Filtered	Filtered
Q562R1	ACTBL2	Beta-actin-like prot	42003.17	1.32E+05	123756.2891	100652.1172	132283.8906	113241.8125	158419.1875	135228.9219	143631.2188
P01591	JCHAIN	Immunoglobulin J cha	18098.58	1.31E+05	162881.7344	45728.95313	25240.24414	79507.11719	61842.36719	25579.13281	48804.42969
A6NHR9	SMCHD1	Structural maintenan	226373.57	1.30E+05	133573.75	137135.1875	153330.5781	193812.2188	127021.0938	126164.7891	115340.4375
Q9UJV3	MID2	Probable E3 ubiquiti	83210.25	1.29E+05	180520.9219	216133.3125	103446.8516	106590.6953	78325.30469	115435.7031	35503.5
O43396	TXNL1	Thioredoxin-like pro	32251.3	1.28E+05	18219.95117	Filtered	6161.471191	4773.635742	4961.314453	Filtered	Filtered

P09486	SPARC	SPARC	34632.16	1.27E+05	100054	128772.8594	152081.2344	128673.0469	113659.875	150514.3438	124579.8125
P61626	LYZ	Lysozyme C	16537.01	1.26E+05	115217.4297	107312.0078	143495.75	115093.8203	124390.1953	158661.2188	111198.5469
P01743		Ig heavy chain V-I r	12945.68	1.25E+05	79737.10938	41614.77344	40160.91797	39321.68359	47131.57031	26140.83203	63984.89063
P33151	CDH5	Cadherin-5	87528.41	1.24E+05	118358.0234	157176.125	174624.1563	149459.1094	104001.5781	125746.1641	152893.7813
O95777	LSM8	U6 snRNA-associated	10402.64	1.23E+05	Filtered	138784.7344	156436.3281	137419.9375	98985.30469	132907.4375	125448.0234
P55072	VCP	Transitional endopla	89321.8	1.23E+05	Filtered	Filtered	Filtered	Filtered	Filtered	Filtered	Filtered
P05154	SERPINA5	Plasma serine protea	45674.67	1.21E+05	259314.8594	13657.55566	12393.12207	151910.3125	171802.4375	190762.6563	162055.1563
P04278	SHBG	Sex hormone-binding	43779.2	1.21E+05	232051.5625	91128.19531	58680.77344	143916.0625	89582.88281	76197.14063	153109.6406
P01034	CST3	Cystatin-C	15799.22	1.17E+05	139425.6563	103315.7188	113973.2344	103912.875	86116.6875	134255.2969	130931.2188
P02538	KRT6A	Keratin, type II cyt	60044.95	1.12E+05	Filtered	Filtered	Filtered	21956.28906	47391.97266	Filtered	Filtered
P12236	SLC25A6	ADP/ATP translocase	32866.26	1.09E+05	55331.16016	79030.02344	65300.75781	111720.4453	35883.29688	82127.67969	54003.60156
P05976	MYL1	Myosin light chain 1	21145.1	1.05E+05	Filtered	Filtered	Filtered	Filtered	Filtered	Filtered	Filtered
P13639	EEF2	Elongation factor 2	95338.14	1.04E+05	Filtered	Filtered	Filtered	Filtered	4912.869629	Filtered	Filtered
P32119	PRDX2	Peroxiredoxin-2	21891.92	1.02E+05	54439.16406	52543.21094	22848.01758	124243.4766	111002.8047	57597.84766	85136.8125
O00291	HIP1	Huntingtin-interacti	116221.14	1.02E+05	Filtered	Filtered	Filtered	83286.13281	105033.4766	Filtered	Filtered
P78386	KRT85	Keratin, type II cut	55802.11	9.86E+04	Filtered	136432.4219	Filtered	Filtered	Filtered	Filtered	Filtered
P07359	GP1BA	Platelet glycoprotei	71540.03	9.80E+04	85018.54688	103529.7734	120354.0781	96647.35938	71208.5625	125369.2109	104794.5391
P01861	IGHG4	Ig gamma-4 chain C r	35940.56	9.77E+04	24963.33594	31442.5332	7507.61377	4615.476563	12342.88379	15151.72461	40839.11328
P49368	CCT3	T-complex protein 1	60533.88	9.68E+04	Filtered	12508.36133	34256.87109	13563.55078	9955.730469	17930.89648	Filtered
Q6EMK4	VASN	Vasorin	71712.65	9.58E+04	100223.5625	90622.46875	98768.67188	85460.02344	77334.82031	116877.9297	106504.2969
Q6UXB8	PI16	Peptidase inhibitor	49471.05	9.52E+04	76458.11719	100070.5625	142734.5313	94214.53125	101218.0313	157886.9219	99969.21875
Q9NRM6	IL17RB	Interleukin-17 recep	55884.53	9.15E+04	22839.93945	26104.84375	23015.73242	29752.2832	15975.18652	17697.49609	37522.94531
P12081	HARS	Histidine--tRNA liga	57410.5	9.15E+04	41307.75	55430.04688	83733.02344	34684.61328	31015.16797	19165.81641	25620.32617
Q92954	PRG4	Proteoglycan 4	151077.11	8.98E+04	59348.24219	58484.26172	109937.4219	55895.83984	49835.75781	78312.69531	58592.92188
Q15485	FCN2	Ficolin-2	34001.14	8.94E+04	84318.64844	62538.31641	118816.3047	58365.98047	55498.98828	101983.1719	78065.23438
Q9ULV0	MYO5B	Unconventional myosi	213672.38	8.92E+04	Filtered	22846.66992	Filtered	Filtered	Filtered	4186.103027	53049.71875
P40197	GP5	Platelet glycoprotei	60959.19	8.78E+04	75878.33594	94405.625	119523.2422	91435.67188	76041.52344	97268.14844	114223.5469
Q15293	RCN1	Reticulocalbin-1	38889.99	8.65E+04	51125.18359	43314.26953	40377.70313	122592.2344	35783.54688	129931.4922	35965.48438
P35908	KRT2	Keratin, type II cyt	65432.88	8.57E+04	41355.20313	56093.41406	128522.1406	162516.9063	9896.998047	41860.45313	39570.57031
P60709;P63261	ACTB;ACTG1	Actin, cytoplasmic 1	41736.73;41792.84	8.49E+04	78519.08594	67031.39844	82016.34375	65746.11719	98085.01563	85005.15625	91834.125
P07451	CA3	Carbonic anhydrase 3	29557.42	8.31E+04	Filtered	Filtered	Filtered	6961.578613	Filtered	Filtered	Filtered
P35030	PRSS3	Trypsin-3	32528.83	8.18E+04	75408.17188	82939.77344	59443.86719	67788.64063	68447.13281	61202.0625	74257.64063
P01617		Ig kappa chain V-II	NaN	7.79E+04	Filtered	Filtered	Filtered	Filtered	Filtered	Filtered	Filtered
P13645	KRT10	Keratin, type I cyto	58827.09	7.73E+04	72985.0625	47376.92969	103985.5781	91380.88281	32642.32813	36984.17969	40790.74609
P11277	SPTB	Spectrin beta chain,	246468.1	7.57E+04	14055.16895	32688.40039	52718.4375	Filtered	Filtered	31085.4043	29245.66406
P10124	SRGN	Serglycin	17651.79	7.52E+04	81710.74219	60720.85938	78383.96094	46338.26953	41070.375	54944.58594	95213.375
P55058	PLTP	Phospholipid transfe	54739.43	7.51E+04	90883.17969	115681.9531	88077.5	85963.72656	85212.82813	84011.15625	96491.23438
Q5SRE5	NUP188	Nucleoporin NUP188 h	196042.98	7.40E+04	Filtered	Filtered	Filtered	Filtered	Filtered	Filtered	Filtered
P02763	ORM1	Alpha-1-acid glycopr	23511.56	7.32E+04	127749.8906	64947.625	45957.97656	39280.57031	31882.23047	38121.90234	31558.29492
P11216	PYGB	Glycogen phosphoryla	96695.95	7.11E+04	Filtered	Filtered	Filtered	Filtered	Filtered	Filtered	Filtered
P49747	COMP	Cartilage oligomeric	82860.48	7.05E+04	70265.57031	76652.83594	74157.13281	56822.19531	63432.42969	98267.38281	72314.72656
P02786	TFRC	Transferrin receptor	84871.37	7.02E+04	112363.4766	157492.7188	95024.82813	160473.0469	67066.73438	76335.42188	80483.08594
P80748		Ig lambda chain V-II	11935.15	7.00E+04	17417.80469	20986.69922	9424.571289	21890.66406	29360.25195	10826.99023	30649.35352
Q9Y4B5	MTCL1	Microtubule cross-li	209525.99	6.96E+04	95649.75781	Filtered	Filtered	39371.83203	Filtered	Filtered	Filtered
P08519	LPA	Apolipoprotein(a)	501319.13	6.96E+04	18119.25586	68365.04688	84082.26563	236299.7969	154265.875	76219.22656	201531.3438
Q9NSB4	KRT82	Keratin, type II cut	56652.69	6.95E+04	Filtered	135265.9375	Filtered	Filtered	Filtered	Filtered	Filtered
Q06830	PRDX1	Peroxiredoxin-1	22110.35	6.92E+04	41504.58203	36498.88281	27656.49023	55260.84375	41863.48047	34957.82031	38145.55469
Q9Y5Y7	LYVE1	Lymphatic vessel end	35213.43	6.92E+04	46035.36719	74434.95313	58893.23828	54370.93359	63341.94922	78796.32813	97668.02344
O60462	NRP2	Neuropilin-2	104858.67	6.88E+04	53286.08984	77350.75781	106955.5156	68799.26563	6987.645508	62387.24609	62484.375
P04206		Ig kappa chain V-III	11830.3	6.60E+04	37769.49609	23347.73242	26402.48242	22077.16016	23283.57227	21842.38477	82034.5
P35527	KRT9	Keratin, type I cyto	62064.32	6.53E+04	55701.65625	21269.1582	150551.8281	64126.64063	23411.79492	96908.65625	20581.28711
Q9P2P1	NYNRIN	Protein NYNRIN	208366.33	6.47E+04	33208.39063	33516.32813	34994.95703	15730.82813	46398.59766	46741.125	38423.8125
O75144	ICOSLG	ICOS ligand	33348.83	6.43E+04	46073.19922	44220.21484	64081.17969	74744.92188	81158.65625	76239.28906	73547.60938
Q02818	NUCB1	Nucleobindin-1	53879.3	6.36E+04	Filtered	167491.8125	686858.25	63537.23047	2456.541748	22758.15234	Filtered
Q8IVU3	HERC6	Probable E3 ubiquiti	115126.27	6.30E+04	71530.94531	121379.1406	159193.5156	107196	51443.42969	40094.09375	39019.93359
P08637	FCGR3A	Low affinity immunog	29089.13	6.18E+04	51838.18359	36946.75391	61009.95313	48272.55859	91234.85938	93716.03125	60230.70313
P68431	HIST1H3A	Histone H3.1	15404.08	6.17E+04	Filtered	Filtered	Filtered	Filtered	44352.01172	Filtered	Filtered
P61769	B2M	Beta-2-microglobulin	13714.56	6.12E+04	25769.20508	61675.13281	23063.75977	4439.604004	66743.63281	27272.3125	52830.89063
P62068	USP46	Ubiquitin carboxyl-t	42442.39	6.00E+04	Filtered	Filtered	Filtered	Filtered	Filtered	Filtered	Filtered
P04430		Ig kappa chain V-I r	11840.13	5.91E+04	Filtered	24899.19531	29334.09375	Filtered	18207.92773	14082.14551	47847.54688
P03950	ANG	Angiogenin	16550.07	5.90E+04	44649.83594	66584.67188	46537.75781	50308.91406	66602.24219	81791.39844	63552.375
Q92496	CFHR4	Complement factor H-	65350.76	5.85E+04	69599.63281	149313.4844	79442.6875	29323.54883	42383.43359	66394.49219	43663.95703
P01779		Ig heavy chain V-III	NaN	5.83E+04	19618.46289	16576.94922	26112.51758	18803.29102	23865.67383	19342.91797	31380.77734
P09493	TPM1	Tropomyosin alpha-1	32708.56	5.79E+04	Filtered	Filtered	Filtered	Filtered	Filtered	Filtered	Filtered
P14735	IDE	Insulin-degrading en	117968.49	5.74E+04	70338	71686.45313	Filtered	76798.84375	Filtered	55208.06641	61726.33984
Q9BWP8	COLEC11	Collectin-11	28665.37	5.73E+04	65474.63672	77617.35938	56495.93359	66153.26563	48543.49219	61503.15234	67033.41406
Q01973	ROR1	Inactive tyrosine-pr	104282.61	5.72E+04	69674.40625	53890.42578	65940.94531	52111.98438	39661.98438	45454.58984	66772.15625
Q8TEX9	IPO4	Importin-4	118715.32	5.65E+04	38779.55078	39620.85156	62471.35547	36653.41406	20119.33398	38137.21094	48554.20313
Q9Y6Z7	COLEC10	Collectin-10	30705.02	5.65E+04	65022.39844	44853.125	78420.28125	46787	37963.82422	42413.45313	41686.78516
P01717		Ig lambda chain V-IV	11516.76	5.59E+04	Filtered	Filtered	Filtered	Filtered	Filtered	Filtered	Filtered
Q9UHG3	PCYOX1	Prenylcysteine oxida	56640.13	5.49E+04	94852.85938	63706.31641	51618.97656	72461.5	20808.7793	37937.28906	51847.53125
Q9NQ79	CRTAC1	Cartilage acidic pro	71420.7	5.41E+04	109783.7969	67813.21875	54632.25	56649.36328	40622.94141	78875.77344	62195.48828
P24592	IGFBP6	Insulin-like growth	25322.43	5.38E+04	46676.21875	48949.30469	59157.39063	49213.83984	41212.53125	80383.19531	60508.00391
Q13103	SPP2	Secreted phosphoprot	24337.65	5.36E+04	64838.71484	45020.97266	60540.49609	95288.25	32086.25195	60902.32031	84780.6875
P01625		Ig kappa chain V-IV	12640.07	5.30E+04	69490.80469	43186.69922	24183.59961	37683.47656	23702.375	26912.54688	39139.80859

Q14571	ITPR2	Inositol 1,4,5-trisp	308063.65	5.29E+04	Filtered	47286.85938	72209.59375	32244.27734	45908.86328	65496.01563	41525.26953
P01700;P04208		Ig lambda chain V-I	11896.17;11724.96	5.27E+04	13954.57324	14882.69238	8626.592773	10914.51758	11925.79688	7336.414063	26716.60352
P48668	KRT6C	Keratin, type II cyt	60024.95	5.17E+04	41717.03125	20200.57422	64960.19531	38932.74609	42575.48438	24490.48828	16915.32813
Q96C86	DCPS	m7GpppX diphosphatas	38608.8	5.12E+04	Filtered	Filtered	Filtered	Filtered	Filtered	Filtered	Filtered
Q9Y2H5	PLEKHA6	Pleckstrin homology	117127.98	5.05E+04	99583.67969	73572	84090.94531	84292.1875	64176.14844	81391.32031	63432.98047
Q9UNN8	PROCR	Endothelial protein	26671.43	5.01E+04	42132.51953	51015.64844	43820.52734	89850.97656	45179.48828	48127.31641	55502.5625
Q9BXR6	CFHR5	Complement factor H-	64419.4	5.01E+04	72351.95313	69345.625	83962.52344	49079.11328	44672.87109	58427.76563	47038.3125
Q99497	PARK7	Protein deglycase DJ	19891.05	4.97E+04	Filtered	38486.76172	24213.10938	18859.88477	27958.16016	25399.79883	Filtered
P60174	TPI1	Triosephosphate isom	30790.99	4.96E+04	7854.639648	11191.5459	119510.2891	1637.485107	7840.879883	99635.10938	4708.613281
P23083		Ig heavy chain V-I r	13008.71	4.96E+04	31256.78516	36727.73438	13640.12305	22293.98047	Filtered	16859.51367	17745.23633
O75150	RNF40	E3 ubiquitin-protein	113650.39	4.94E+04	16066.54492	14595.91895	11576.57129	9311.175781	9342.314453	6886.192871	13647.4668
Q15582	TGFBI	Transforming growth	74680.9	4.84E+04	49736.51172	45436.33594	40434.26172	40838.62109	43418.73438	56135.35156	65499.375
P09172	DBH	Dopamine beta-hydrox	69064.98	4.82E+04	51374.96094	85512.59375	13850.76367	48823.91016	14352.14941	13320.22363	4243.065918
Q4LDE5	SVEP1	Sushi, von Willebran	390170	4.81E+04	17112.25781	13600.07813	2959.478027	Filtered	Filtered	3866.207275	3248.617188
Q9HDC9	APMAP	Adipocyte plasma mem	46480.36	4.79E+04	59449.36719	64765.96484	69443.54688	63659.61719	74439.23438	45928.21094	18586.24609
Q86YW5	TREML1	Trem-like transcript	32678.55	4.79E+04	63904.24219	55377.25	49317.71094	66082.1875	51663.69922	51789.16406	77614.82813
P22692	IGFBP4	Insulin-like growth	27933.97	4.79E+04	40534.21875	41281.02734	55077.80469	51013.32813	47544.01563	47160.05859	48687.52344
Q9Y6R7	FCGBP	IgGFc-binding protei	572016.67	4.70E+04	25605.55273	27429.8418	23836.50391	20003.1543	21185.73633	16486.6875	20211.0625
P01033	TIMP1	Metalloproteinase in	23170.86	4.70E+04	45392.23047	46354.07031	59486.84375	54386.51172	39982.13672	46748.21484	59431.19531
P13598	ICAM2	Intercellular adhesi	30654.31	4.70E+04	35088.13672	57235.47266	47418.61328	61715.4375	39859.29297	42844.40234	71879.41406
P01772		Ig heavy chain V-III	13718.2	4.65E+04	7595.99707	Filtered	32572.5293	40481.6875	Filtered	24840.11328	41855.21094
P02549	SPTA1	Spectrin alpha chain	280014.47	4.61E+04	124253.4844	Filtered	Filtered	Filtered	3664.705078	98745.64063	Filtered
B9A064	IGLL5	Immunoglobulin lambd	23063.24	4.57E+04	14526.51855	1619.186157	8983.763672	8111.423828	39320.82422	2356.187012	25062.88867
P08709	F7	Coagulation factor V	51593.85	4.52E+04	55445.62109	37103.25	50359.16797	46931.42969	44962.83984	58272.04688	52234.80859
P18206	VCL	Vinculin	123799.3	4.50E+04	603738.3125	4664.573242	3564.655273	6491.510254	4680.964355	4412.576172	4659.510742
P12882;Q9UKX2	MYH1;MYH2	Myosin-1;Myosin-2	223145.43;223044.45	4.49E+04	Filtered	Filtered	Filtered	Filtered	Filtered	Filtered	Filtered
P14314	PRKCSH	Glucosidase 2 subuni	59425.41	4.37E+04	3278.973145	Filtered	475063.75	4483.864258	Filtered	4410.836914	4030.817871
P01609		Ig kappa chain V-I r	11764.01	4.35E+04	22049.83984	13950.77051	29195.01172	15607.77148	18000.56055	13985.72461	48701.12891
P07355	ANXA2	Annexin A2	38604.04	4.32E+04	Filtered	Filtered	Filtered	Filtered	Filtered	Filtered	25777.51563
Q9UKV8	AGO2	Protein argonaute-2	97208.11	4.30E+04	Filtered	Filtered	Filtered	Filtered	Filtered	29163.125	Filtered
P01833	PIGR	Polymeric immunoglob	83283.57	4.18E+04	Filtered	Filtered	Filtered	Filtered	71164.15625	Filtered	Filtered
P62805	HIST1H4A	Histone H4	11367.34	4.08E+04	18384.47852	23715.39063	14342.37109	14147.25098	57644.83984	11060.89941	14470.2373
P00739	HPR	Haptoglobin-related	39029.56	4.08E+04	6638.007324	19453.23633	15285.32422	24759.38672	25865.71875	11974.75293	18888.41211
Q9NPH3	IL1RAP	Interleukin-1 recept	65418.44	4.06E+04	65712.16406	65167.84375	42215.57031	64891.89453	41633.42188	40707.53125	49125.32422
Q9BZE9	ASPSCR1	Tether containing UB	60183.07	4.05E+04	33032.50781	40479.03125	50522.53516	38022.39063	35477.99609	38787.55859	54388.23828
P61158	ACTR3	Actin-related protei	47371.17	4.05E+04	28287.38477	865.5178223	68679.14063	81400.0625	2238.996826	54974.53125	63812.375
P68032;P68133	ACTC1;ACTA1	Actin, alpha cardiac	42018.97;42051.03	3.99E+04	25323.01367	25747.42383	40857.29688	25444.58984	37512.54688	31581.70703	32409.55078
Q9H8L6	MMRN2	Multimerin-2	104408.55	3.95E+04	32278.43555	28779.83203	27987.62109	37677.73828	42565.20313	59811.35938	59102.75391
P04908;P0C0S5;P0C0S8	HIST1H2AB;H2AFZ;HIST	Histone H2A type 1-B	14135.49;13552.73;14	3.91E+04	14598.26465	32335.14453	13233.18066	8996.429688	60365.3125	7994.931641	13684.56836
P22352	GPX3	Glutathione peroxida	25552.33	3.87E+04	60691.43359	28410.86719	28286.49609	34612.17578	33421.5625	22777.35352	34084.26172
P62328	TMSB4X	Thymosin beta-4	5052.65	3.87E+04	23893.41406	21415.94922	50915.79688	37863.38281	29766.30469	35041.98828	59473.75391
Q6UX71	PLXDC2	Plexin domain-contai	59583.16	3.85E+04	43287.23828	37343.69141	44972.24219	42772.14844	43114.88672	43945.16016	47972.44531
P13796	LCP1	Plastin-2	70288.39	3.79E+04	44135.42969	39494.90625	49886.12891	41015.48438	37235.96875	47541.98828	42049.42578
P41222	PTGDS	Prostaglandin-H2 D-i	21028.82	3.79E+04	17707.70898	28360.95313	47951.84375	20255.65039	24185.45898	27947.46484	26166.79297
P05787	KRT8	Keratin, type II cyt	53704.24	3.78E+04	1890.97998	Filtered	4742.465332	Filtered	37098.14063	Filtered	Filtered
P49914	MTHFS	5-formyltetrahydrofo	23255.65	3.77E+04	9518.109375	35944.83594	56569.80469	33078.30859	24592.02539	32489.32422	Filtered
P05019	IGF1	Insulin-like growth	21841.19	3.71E+04	66680.17188	70674.24219	13645.84375	21251.39063	43195.76563	15978.73438	75959.38281
P08779	KRT16	Keratin, type I cyto	51267.82	3.68E+04	Filtered	Filtered	3317.55249	Filtered	7533.291992	Filtered	Filtered
O75083	WDR1	WD repeat-containing	66193.52	3.68E+04	Filtered	Filtered	Filtered	Filtered	Filtered	2216.394043	Filtered
P06310		Ig kappa chain V-II	14706.7	3.66E+04	20489.12109	Filtered	23265.90234	17113.10547	15087.30176	9463.612305	39161.80469
Q99969	RARRES2	Retinoic acid recept	18617.58	3.64E+04	26486.48828	32867.26953	42170.78906	28888.64453	32672.68945	36338.44922	31207.53125
Q01459	CTBS	Di-N-acetylchitobias	43759.83	3.62E+04	48183.24609	34668.26953	45262.39844	51556.68359	31687.42383	53934.17578	57928.75781
Q12955	ANK3	Ankyrin-3	480410.01	3.62E+04	35475.42969	34837.94531	38389.25781	30972.81836	28898.13672	60794.75	56496.99609
P08294	SOD3	Extracellular supero	25850.91	3.60E+04	36152.94141	371072.75	41401.07422	35743.98438	32627.92969	46863.91406	47993.18359
P31946	YWHAB	14-3-3 protein beta/	28082.39	3.60E+04	21124.19727	7326.72168	100893.2344	9956.560547	10703.7959	31034.48047	26636.11523
P20908	COL5A1	Collagen alpha-1(V)	183559.87	3.53E+04	Filtered	Filtered	Filtered	Filtered	7268.692383	Filtered	Filtered
P15085	CPA1	Carboxypeptidase A1	47140.27	3.53E+04	Filtered	Filtered	Filtered	Filtered	35283.24609	Filtered	Filtered
P13647	KRT5	Keratin, type II cyt	62378.34	3.50E+04	20010.45703	19122.01953	21994.48633	85098.96875	17843.27148	11308.4248	12625.79395
P01702;P06316		Ig lambda chain V-I	11453.61;13564.22	3.45E+04	Filtered	Filtered	Filtered	Filtered	Filtered	Filtered	Filtered
P13727	PRG2	Bone marrow proteogl	25205.55	3.45E+04	45720.03906	34216.5625	28240.36523	47372.38672	20675.7168	30523.70508	40692.48828
Q9BTY2	FUCA2	Plasma alpha-L-fucos	54066.68	3.43E+04	29373.02148	23137.14453	42757.74219	32854.07031	10183.52637	19941.55469	23373.99805
Q92823	NRCAM	Neuronal cell adhesi	143889.73	3.42E+04	21839.0332	62274.82813	20902.60938	10478.31348	22233.54492	26052.19336	59127.87109
P30043	BLVRB	Flavin reductase (NA	22119.34	3.42E+04	10574.83691	14542.0166	8835.157227	47323.58984	30748.30859	22008.12305	28791.00391
O94819	KBTBD11	Kelch repeat and BTB	65719.85	3.36E+04	Filtered	Filtered	Filtered	Filtered	Filtered	Filtered	Filtered
P02042	HBD	Hemoglobin subunit d	16055.47	3.30E+04	Filtered	Filtered	Filtered	Filtered	Filtered	Filtered	Filtered
P08238	HSP90AB1	Heat shock protein H	83264.19	3.24E+04	3585.8396	4539.079102	Filtered	4638.75	8383.673828	33761.22656	27569.75977
P24593	IGFBP5	Insulin-like growth	30570.24	3.20E+04	24367.73438	25338.91016	31404.43555	24607.24023	21600.28906	205167.1094	32272.67578
P33908	MAN1A1	Mannosyl-oligosaccha	72968.53	3.17E+04	60597.76172	41894.01953	53574.5625	40197.92969	43251.34766	43267.15625	41313.69922
P16233	PNLIP	Pancreatic triacylgl	51156.83	3.16E+04	Filtered	Filtered	Filtered	Filtered	31577.97266	Filtered	Filtered
P30530	AXL	Tyrosine-protein kin	98335.73	3.14E+04	12093.1748	42590.60938	58727.42188	46146.35547	38841.17578	34180.55469	54140.87109
P01880	IGHD	Ig delta chain C reg	42253.7	3.14E+04	Filtered	Filtered	8448.695313	Filtered	115260.9219	Filtered	8728.450195
P08174	CD55	Complement decay-acc	41400.13	3.13E+04	67688.67188	Filtered	6285.911133	Filtered	Filtered	4198.874023	3895.42334
Q6VUC0	TFAP2E	Transcription factor	46212.09	3.05E+04	15926.36914	15038.29199	Filtered	Filtered	16720.92383	Filtered	Filtered
Q86UD1	OAF	Out at first protein	30688.28	3.02E+04	31197.99219	24903.18164	33674.85938	29190.97266	32164.27148	37882.88281	27573.81641
P06737	PYGL	Glycogen phosphoryla	97148.6	3.00E+04	Filtered	Filtered	Filtered	Filtered	Filtered	Filtered	Filtered

P00915	CA1	Carbonic anhydrase 1	28870.2	3.00E+04	171805.0469	48208.78125	4927.420898	63171.36719	36308.42969	18656.32031	39158.61719
P00488	F13A1	Coagulation factor X	83267.29	2.97E+04	36801.78516	24202.25195	30486.46875	24532.04688	22671.00391	28247.40234	24771.71484
P42766	RPL35	60S ribosomal protei	14551.47	2.95E+04	Filtered	37247.66797	38152.51563	39304.42578	Filtered	31044.99805	34633.9375
P00918	CA2	Carbonic anhydrase 2	29246.05	2.93E+04	15266.38086	19061.64258	6765.918945	45069.625	30720.56836	14500.5625	22611.7168
P01608		Ig kappa chain V-I r	11782.19	2.92E+04	7257.071289	Filtered	Filtered	Filtered	Filtered	Filtered	Filtered
Q76LX8	ADAMTS13	A disintegrin and me	153604.44	2.92E+04	31794.54492	33877.0625	28734.68359	33224.49219	19800.35547	32748.32031	37246.57031
Q12884	FAP	Prolyl endopeptidase	87712.55	2.91E+04	4998.463867	Filtered	3343.766846	Filtered	Filtered	4864.012207	Filtered
P13473	LAMP2	Lysosome-associated	44960.73	2.90E+04	81881.75781	41147.98047	56760.97656	48894.78906	34034.98828	39812.34375	39586.44922
P00491	PNP	Purine nucleoside ph	32117.86	2.87E+04	4277.252441	3322.968994	Filtered	12017.23242	7799.987793	3581.084229	26710.85156
P42785	PRCP	Lysosomal Pro-X carb	55799.79	2.86E+04	41135.03906	90430.42969	Filtered	Filtered	2549.50708	17767.43164	6264.122559
P22105	TNXB	Tenascin-X	458220.29	2.85E+04	25279.34766	29851.66992	26901.28711	25070.91602	25768.32422	25919.0918	36766.16797
O00391	QSOX1	Sulfhydryl oxidase 1	82577.73	2.82E+04	47520.46484	39734.70703	31516.51172	31226.79102	22860.50586	32912.10156	33685.24219
P14625	HSP90B1	Endoplasmin	92468.86	2.81E+04	67618.04688	34100.78125	28004.67383	28420.07813	22104.7207	64577.08203	26643.17188
P17066	HSPA6	Heat shock 70 kDa pr	71028.14	2.81E+04	32579.10547	26711.95117	30004.43359	30759.88086	29954.50391	24912.10156	30429.66797
P13591	NCAM1	Neural cell adhesion	94574.16	2.80E+04	29108.32031	27385.66602	29601.60938	35823.43359	27189.54492	29134.17383	39609.60547
Q6UY14	ADAMTSL4	ADAMTS-like protein	116545.08	2.78E+04	21355.30859	19150.14258	36170.66797	53011.33203	34510.30078	19212.32813	20113.23828
P10646	TFPI	Tissue factor pathwa	35015.1	2.76E+04	31393.55664	26673.68555	30384.74023	Filtered	Filtered	28516.42969	20830.77734
O95497	VNN1	Pantetheinase	57011.67	2.75E+04	Filtered	15074.4873	495931.0313	2278.270752	25466.23828	8467.942383	Filtered
P02533	KRT14	Keratin, type I cyto	51561.46	2.75E+04	11190.61523	49221.25	21311.38672	16489.92773	7440.725586	11432.62793	33268.12891
P13646	KRT13	Keratin, type I cyto	49588.21	2.70E+04	Filtered	41973.80078	Filtered	Filtered	Filtered	Filtered	Filtered
A6NKQ9;P0DN86;P0DN87	CGB1;CGB3;CGB7;CGB2	Choriogonadotropin s	20467.97;17738.72;17	2.70E+04	14816.55762	12555.95898	17461.2207	26956.22852	18886.12109	21105.2793	15132.18848
P09211	GSTP1	Glutathione S-transf	23355.83	2.66E+04	35115.27344	Filtered	Filtered	6057.452637	Filtered	5675.255859	Filtered
P05067	APP	Amyloid beta A4 prot	86943.24	2.58E+04	15060.3623	25299.9668	38965.58203	24819.79883	31142.18359	29646.38281	27493.83789
Q9NPY3	CD93	Complement component	68560.02	2.58E+04	28588.02539	26622.90234	30671.88672	24997.95117	28516.97461	37221.16797	31917.11719
Q9NPR2	SEMA4B	Semaphorin-4B	92193.23	2.56E+04	22144.55078	21590.12305	24296.66406	23225.25781	7913.096191	22799.20508	21824.83594
P15151	PVR	Poliovirus receptor	45302.6	2.55E+04	27830.88672	28635.92578	17906.1543	26407.58984	20058.50977	31690.30469	31449.17578
O75116	ROCK2	Rho-associated prote	160899.82	2.55E+04	Filtered	Filtered	32035.20508	33052.70313	9976.71582	11809.89063	Filtered
P01613		Ig kappa chain V-I r	NaN	2.51E+04	11652.54199	8583.37207	Filtered	Filtered	Filtered	Filtered	9281.943359
O00533	CHL1	Neural cell adhesion	135071.47	2.51E+04	27543.42578	27818.73828	23035.6582	28038.60352	27756.40234	24120.51367	30663.67578
P08195	SLC3A2	4F2 cell-surface ant	67994	2.50E+04	20207.17188	30091.08789	23104.50391	34130.07813	21974.91016	26343.82617	29577.875
P15144	ANPEP	Aminopeptidase N	109539.6	2.47E+04	36854.58984	43295	31180.45313	31298.67578	35896.04688	39042.60156	39032.64063
Q96CX2	KCTD12	BTB/POZ domain-conta	35700.78	2.44E+04	Filtered	Filtered	Filtered	1993.848755	Filtered	1490.429932	Filtered
P07998	RNASE1	Ribonuclease pancrea	17644.24	2.42E+04	20056.60742	22934.61914	34917.29688	28959.54688	23165.14648	30360.28516	27575.23828
P31944	CASP14	Caspase-14	27679.51	2.42E+04	Filtered	Filtered	Filtered	38274.34375	Filtered	Filtered	Filtered
P08253	MMP2	72 kDa type IV colla	73882.3	2.41E+04	25388.89063	31009.59961	25081.35156	27132.72852	29968.54883	33439.33984	36069.31641
Q7Z7G0	ABI3BP	Target of Nesh-SH3	118641.97	2.39E+04	29131.74609	23694.72656	23684.19922	24594.58398	27679.21875	24956.10156	31394.50586
Q9BY67	CADM1	Cell adhesion molecu	48509.09	2.39E+04	23216.46875	31201.9375	49058.21094	27008.59766	15558.61523	16019.4502	21904.16016
P54108	CRISP3	Cysteine-rich secret	27630.28	2.38E+04	51840.42969	63007.64844	40290.24219	37068.00781	26397.51953	43856.32813	37666.80469
Q14165	MLEC	Malectin	32233.87	2.37E+04	Filtered	Filtered	Filtered	Filtered	Filtered	28748.79688	Filtered
P35443	THBS4	Thrombospondin-4	105869.3	2.35E+04	16129.87207	19415.71484	19774.68555	13725.6123	37028.16016	15148.52051	24468.99805
P53634	CTSC	Dipeptidyl peptidase	51853.82	2.34E+04	8000.589844	7479.147949	158249.4844	10172.44629	Filtered	9593.666016	Filtered
P68104;Q5VTE0	EEF1A1;EEF1A1P5	Elongation factor 1-	50140.86;50185.02	2.33E+04	19905.70313	32910.375	14815.38477	16609.54297	19637.37109	15820.81836	53398.77344
P23470	PTPRG	Receptor-type tyrosi	162003.47	2.32E+04	26413.37891	22162.15039	22198.8457	24041.53906	18233.70508	28767.79492	27911.56641
P01598		Ig kappa chain V-I r	11788.23	2.32E+04	14551.42871	7194.480957	8393.941406	8892.896484	6902.917969	5772.249512	15554.2666
P06753;P07951;P09493	TPM3;TPM2;TPM1;TPM4	Tropomyosin alpha-3	32949.97;32850.73;32	2.32E+04	Filtered	Filtered	10675.92773	9127.411133	9317.307617	8692.430664	11637.08301
P14780	MMP9	Matrix metalloprotei	78458.23	2.31E+04	63652.45313	28208.92969	50754.55469	13970.77344	14851.61621	33446.30859	18910.51758
Q12860	CNTN1	Contactin-1	113320.41	2.31E+04	26447.94141	30986.80469	25172.375	32398.75586	18214.75586	26396.42383	31600.56445
Q15113	PCOLCE	Procollagen C-endope	47972.49	2.30E+04	30375.9707	18842.14844	21857.74219	22908.36914	24095.58984	30357.15234	30719.00586
P59665;P59666	DEFA1;DEFA3	Neutrophil defensin	10200.94;10244.95	2.30E+04	28255.1875	24332.85938	31242.9707	20303.33984	22917.62695	38855.96484	17658.88086
P55103	INHBC	Inhibin beta C chain	38237.86	2.30E+04	9782.183594	13839.51172	25461.42969	27750.19727	16357.75977	33273.96484	20959.53711
Q14126	DSG2	Desmoglein-2	122293.92	2.29E+04	32256.70508	28054.81641	23499.01563	31720.47852	17053.65625	28671.02344	28597.33008
O60814;P06899;P23527	HIST1H2BK;HIST1H2BJ;	Histone H2B type 1-K	13890.13;13904.16;13	2.27E+04	7316.113281	17122.60156	Filtered	Filtered	39275.34766	Filtered	Filtered
P11021	HSPA5	78 kDa glucose-regul	72332.96	2.27E+04	25240.50586	21871.5918	29290.29102	31519.9375	24197.49609	23113.04102	21214.83594
P36871	PGM1	Phosphoglucomutase-1	61449.12	2.24E+04	Filtered	Filtered	Filtered	Filtered	Filtered	Filtered	Filtered
Q9Y490	TLN1	Talin-1	269767.05	2.22E+04	10567.09961	6832.747559	7298.154297	8878.826172	12365.08301	9935.442383	8002.290039
P00338	LDHA	L-lactate dehydrogen	36688.72	2.18E+04	19411.14063	16798.83008	17786.35352	18501.39648	31048.75781	24552.99219	19981.23828
O43790;P78385;Q14533	KRT86;KRT83;KRT81	Keratin, type II cut	53500.62;54195.4;549	2.18E+04	Filtered	37629.21094	Filtered	Filtered	Filtered	Filtered	Filtered
P10721	KIT	Mast/stem cell growt	109864.56	2.16E+04	28183.71875	14955.77051	15184.52832	17217.84961	11201.69434	17569.75977	22023.69336
Q9H9E3	COG4	Conserved oligomeric	89083.05	2.15E+04	3744.646729	29302.00586	33633.95313	28753.21484	8764.963867	31415.4043	13923.62891
Q9BUN1	MENT	Protein MENT	36769.39	2.15E+04	20019.48633	33816.09375	19836.47266	20988.87305	14554.00488	16783.54102	19300.42969
P13489	RNH1	Ribonuclease inhibit	49973.46	2.15E+04	Filtered	Filtered	Filtered	Filtered	Filtered	Filtered	Filtered
Q86VB7	CD163	Scavenger receptor c	125450.64	2.12E+04	14737.32813	25634.14258	25697.13477	16717.75586	16734.1582	27167.85938	18557.58398
P01611		Ig kappa chain V-I r	11607.91	2.12E+04	Filtered	4341.795898	Filtered	Filtered	Filtered	Filtered	6839.26709
Q13201	MMRN1	Multimerin-1	138110.14	2.08E+04	18590.44531	23515.37305	26459.10547	20261.63672	15967.5752	21535.89453	24811.1582
P98160	HSPG2	Basement membrane-sp	468829.98	2.08E+04	22609.00195	18607.70117	20752.44336	28316.40039	22969.05859	31352.06641	32668.5332
Q8IX30	SCUBE3	Signal peptide, CUB	109282.5	2.08E+04	29916.29883	35519.17188	Filtered	Filtered	12972.47559	6409.575684	7023.567383
P07858	CTSB	Cathepsin B	37821.58	2.06E+04	84009.35156	15173.34766	31426.20703	12043.15039	24590.81445	28394.58789	5549.382813
Q9Y4L1	HYOU1	Hypoxia up-regulated	111335.38	2.06E+04	23139.9082	17815.08594	26796.99609	16680.08594	16432.82422	24093.78516	22936.45508
Q16627	CCL14	C-C motif chemokine	10678.3	2.04E+04	18539.1875	20996.3125	17882.19141	19624.42773	22829.07617	18179.08398	21998.04492
P02452;P02458	COL1A1;COL2A1	Collagen alpha-1(I)	138941.49;141785.37	2.04E+04	Filtered	8299.008789	Filtered	Filtered	14686.02637	21850.79297	Filtered
P01721;P06317		Ig lambda chain V-VI	11917.87;12247.41	2.01E+04	Filtered	Filtered	Filtered	2193.501221	Filtered	Filtered	5194.533203
O14786	NRP1	Neuropilin-1	103134.34	2.00E+04	16408.98047	24648.95313	22036.93359	25793.62305	22983.35547	24598.49414	26354.83203
P01824		Ig heavy chain V-II	14116.8	1.99E+04	36045.07031	6808.769531	Filtered	4710.144043	9700.651367	1714.959106	5714.371582
Q02487	DSC2	Desmocollin-2	99961.68	1.98E+04	Filtered	41517.39063	5225.792969	Filtered	Filtered	35439.10938	Filtered
P32455	GBP1	Guanylate-binding pr	67930.73	1.98E+04	Filtered	Filtered	Filtered	Filtered	Filtered	17019.04688	Filtered

Q99784	OLFM1	Noelin	55342.64	1.98E+04	22766.4668	29169.00195	19772.4082	22452.96094	19514.46484	21326.89258	25935.42773
Q14152;Q96T17	EIF3A;MAP7D2	Eukaryotic translati	166569.37;81965.39	1.97E+04	33931.75391	9579.865234	Filtered	22466.73828	27126.5918	30754.62305	Filtered
P04075	ALDOA	Fructose-bisphosphat	39420.01	1.97E+04	16844.7832	14153.37012	13542.63867	16255.08301	18322.1582	17834.33789	15413.19141
P07195	LDHB	L-lactate dehydrogen	36638.48	1.96E+04	32835.47266	22018.57813	21411.67773	27123.22656	30362.59766	29065.07617	24952.25195
P05362	ICAM1	Intercellular adhesi	57825.22	1.93E+04	19516.2168	20947.37109	13633.97168	19511.25195	16167.29395	15639.71289	22944.64063
Q7Z460	CLASP1	CLIP-associating pro	169450.58	1.93E+04	13948.72656	19234.8125	18329.7168	13416.21191	12528.73535	14386.9209	13697.35254
P02675	FGB	Fibrinogen beta chai	55928.15	1.91E+04	25453.69141	15645.07617	16615.89258	10900.26172	15002.64258	10357.85156	20562.94531
Q9UBQ6	EXTL2	Exostosin-like 2	37465.65	1.91E+04	76413.875	60632.9375	11112.32324	7617.522461	8764.925781	8852.079102	35476.91797
P81605	DCD	Dermcidin	11283.86	1.91E+04	3010.643066	934.5760498	Filtered	Filtered	Filtered	Filtered	Filtered
P0CG47;P0CG48;P62979	UBB;UBC;RPS27A;UBA52	Polyubiquitin-B;Poly	25761.64;77038.61;17	1.90E+04	15515.65527	16393.21094	13521.09668	13466.75684	15299.56738	11302.81934	14109.97754
Q9UNW1	MINPP1	Multiple inositol po	55051.19	1.90E+04	21652.96094	20621.73828	21294.79883	24245.33789	18423.51953	22238.70703	19371.9707
Q12907	LMAN2	Vesicular integral-m	40228.66	1.88E+04	5647.113281	Filtered	Filtered	2711.476563	Filtered	3219.171387	Filtered
Q9UKF6	CPSF3	Cleavage and polyade	77486	1.88E+04	9807.443359	5314.981934	6676.940918	31337.20508	4400.03418	6099.391113	6417.088867
P04745;P04746;P19961	AMY1A;AMY2A;AMY2B	Alpha-amylase 1;Panc	57767.82;57706.85;57	1.87E+04	20160.19922	5806.428223	7909.32666	7660.963379	701284.875	15526.62207	14418.32813
P11597	CETP	Cholesteryl ester tr	54756.21	1.87E+04	21567.54102	20357.60352	14624.12402	13728.33984	14408.77246	15674.65039	7031.267578
Q13093	PLA2G7	Platelet-activating	50077.42	1.86E+04	16672.4707	8678.513672	20964.99023	19529.67969	13292.50195	23607.48438	14893.12891
Q14766	LTBP1	Latent-transforming	186796.1	1.85E+04	17038.70703	19679.29102	21439.8125	16198.29883	16006.24023	18293.7793	20174.50977
P07339	CTSD	Cathepsin D	44552.22	1.84E+04	12574.72461	12084.87207	15446.01563	14258.3457	16659.50977	14059.72754	13549.9668
P01597		Ig kappa chain V-I r	NaN	1.82E+04	Filtered	Filtered	7387.831543	Filtered	Filtered	Filtered	21588.01953
Q15063	POSTN	Periostin	93313.94	1.81E+04	18571.42188	20301.32813	22308.77148	18926.53125	30245.71875	23930.60547	35955.46875
P34096	RNASE4	Ribonuclease 4	16840.47	1.80E+04	23979.38086	17211.53125	16397.95898	18784.79297	18965.57422	18809.66211	16408.77344
P43121	MCAM	Cell surface glycopr	71607.4	1.79E+04	20804.46484	18397.03711	17702.42578	21083.43164	11168.16699	19165.03516	25524.72852
Q14515	SPARCL1	SPARC-like protein 1	75207.74	1.79E+04	16930.28125	24160.31641	22250.21094	19185.56445	16100.10254	17277.64648	22977.15234
P01714		Ig lambda chain V-II	11392.52	1.77E+04	Filtered	Filtered	Filtered	Filtered	Filtered	Filtered	Filtered
P01771		Ig heavy chain V-III	NaN	1.77E+04	10478.46777	Filtered	7033.448242	3184.792236	Filtered	Filtered	6550.715332
Q16853	AOC3	Membrane primary ami	84621.9	1.77E+04	50904.74219	30906.38086	23335.69922	23566.31836	31015.90625	22853.98242	25327.14258
Q9NY97	B3GNT2	N-acetyllactosaminid	46021.99	1.76E+04	15352.32617	28296.33789	17499.05078	15383.15527	13807.4375	17130.66992	18504.96289
Q6ZRP7	QSOX2	Sulfhydryl oxidase 2	77528.74	1.75E+04	16901.62695	Filtered	15894.89453	18490.54883	8873.955078	1828.868164	22287.7793
Q10588	BST1	ADP-ribosyl cyclase/	35723.98	1.75E+04	29566.92188	25801.04492	24951.00391	28287.25781	12118.82324	12946.10352	16913.5625
Q96I34	PPP1R16A	Protein phosphatase	57811.38	1.75E+04	19282.54102	Filtered	Filtered	Filtered	14408.99414	Filtered	Filtered
P09466	PAEP	Glycodelin	20624.22	1.74E+04	Filtered	Filtered	Filtered	Filtered	Filtered	Filtered	32923.61328
O75460	ERN1	Serine/threonine-pro	109735.02	1.72E+04	1029.545776	2982.251953	4565.722168	2362.465088	1720.238892	2496.084473	3303.750244
Q9UJJ9	GNPTG	N-acetylglucosamine-	33973.55	1.72E+04	16983.55469	16824.87109	18833.61328	16646.98242	12176.54297	20373.83789	18656.46484
P01780		Ig heavy chain V-III	NaN	1.70E+04	17866.0957	21858.78516	1860.173218	Filtered	19946.55469	Filtered	6520.03418
P07237	P4HB	Protein disulfide-is	57116.36	1.69E+04	10148.84766	11346.47656	11860.08496	10915.07129	9803.770508	10365.5752	10986.19922
P26038	MSN	Moesin	67820.04	1.68E+04	17504.27539	14672.30566	13593.01074	19170.5918	23446.19727	16171.70508	17300.75977
P15924	DSP	Desmoplakin	331773.89	1.67E+04	Filtered	Filtered	2584.640137	12318.64648	125895.5547	6302.042969	Filtered
Q13822	ENPP2	Ectonucleotide pyrop	98993.58	1.67E+04	20894.5918	19593.14258	15406.33789	25717.11523	26013.17578	12350.5127	17603.90625
P49913	CAMP	Cathelicidin antimic	19301.38	1.66E+04	18944.20703	18696.37109	20644.86719	13941.14453	Filtered	19872.18945	5052.292969
Q16706	MAN2A1	Alpha-mannosidase 2	131140.57	1.66E+04	15981.97656	15422.49609	18008.93945	15433.29883	15023.31152	14608.37109	17268.85742
P35579	MYH9	Myosin-9	226532.24	1.65E+04	72702.1875	Filtered	2807.321533	Filtered	3133.797119	2872.307373	3772.716309
Q6YHK3	CD109	CD109 antigen	161689.08	1.64E+04	12782.38086	12082.84473	17778.9668	16254.07813	13070.65332	12315.99512	17294.5957
P18669	PGAM1	Phosphoglycerate mut	28803.93	1.64E+04	Filtered	8070.812012	Filtered	Filtered	7530.359863	Filtered	Filtered
Q16270	IGFBP7	Insulin-like growth	29130.31	1.63E+04	11740.84766	13539.11426	12482.72461	10043.80566	12910.64258	10419.6123	27769.84375
P21333	FLNA	Filamin-A	280739	1.63E+04	Filtered	Filtered	6258.185547	3588.295898	2025.097412	6423.040527	11851.72852
Q9UM47	NOTCH3	Neurogenic locus not	243631.29	1.63E+04	11583.26074	11934.63574	12358.2959	12574.67871	11381.83398	14074.09668	11615.36328
P12318	FCGR2A	Low affinity immunog	35000.68	1.63E+04	22615.36914	16991.31055	12864.75293	Filtered	Filtered	17448.32422	Filtered
Q13616	CUL1	Cullin-1	89678.52	1.62E+04	Filtered	9191.904297	3269.676025	Filtered	Filtered	Filtered	Filtered
P19320	VCAM1	Vascular cell adhesi	81276.17	1.61E+04	12654.50391	21317.66406	16354.63867	16333.49707	13821.8916	22782.9043	18204.53516
P02679	FGG	Fibrinogen gamma cha	51511.65	1.60E+04	21840.27148	11774.26855	16902.33008	13123.64355	9949.579102	11538.41016	19000.93359
Q96S96	PEBP4	Phosphatidylethanola	25733.38	1.59E+04	16592.15625	14681.47266	21811.02344	12664.34863	11303.5166	13133.26367	9660.138672
P07737	PFN1	Profilin-1	15054.23	1.59E+04	9832.060547	11178.35156	13795.45313	9783.939453	10072.29297	14162.49805	22231.65039
P12830	CDH1	Cadherin-1	97456.15	1.59E+04	16885.46484	15782.9375	18410.98242	15274.66016	10922.97656	14713.25684	17209.66992
P13497	BMP1	Bone morphogenetic p	111248.7	1.58E+04	68360.60938	Filtered	3153.858398	4374.908203	2610.387939	3113.240479	Filtered
P78417	GSTO1	Glutathione S-transf	27565.85	1.57E+04	17861.08008	13401.99902	15594.85059	15351.86328	14747.01758	12420.91602	12869.77734
P07437	TUBB	Tubulin beta chain	49670.81	1.57E+04	12994.81348	11900.64746	13045.5459	11820.08789	19468.58984	10832	15378.19043
P17813	ENG	Endoglin	70578.07	1.56E+04	14425.23828	13081.45508	10169.10156	13553.4502	19307.85742	11421.31152	17632.18164
P04209		Ig lambda chain V-II	11580.65	1.56E+04	4618.333008	3851.117188	Filtered	Filtered	4529.855957	Filtered	Filtered
P20774	OGN	Mimecan	33922.22	1.56E+04	16093.4873	14318.99512	18083.68945	14370.06641	22543.625	16239.18848	21103.56445
P01708		Ig lambda chain V-II	11505.78	1.55E+04	3383.084229	Filtered	Filtered	Filtered	Filtered	Filtered	Filtered
P01596		Ig kappa chain V-I r	NaN	1.55E+04	Filtered	Filtered	Filtered	Filtered	Filtered	Filtered	Filtered
P04406	GAPDH	Glyceraldehyde-3-pho	36053.21	1.54E+04	9491.899414	10236.37207	7925.657227	36317.0625	21999.19922	7046.647461	9446.317383
P26373	RPL13	60S ribosomal protei	24261.47	1.54E+04	23309.98828	11452.69238	9829.93457	17039.99414	15871.71387	16542.59961	19160.56055
Q15166	PON3	Serum paraoxonase/la	39607.48	1.54E+04	27220.48828	19306.37305	13684.02441	16366.19336	16951.42383	16084.25195	18987.10547
Q12841	FSTL1	Follistatin-related	34985.51	1.54E+04	16366.79297	15777.69629	18367.73047	18202.69336	12961.2832	19253.68359	16634.75586
P01619		Ig kappa chain V-III	NaN	1.52E+04	Filtered	Filtered	Filtered	Filtered	Filtered	Filtered	Filtered
Q9UJ41	RABGEF1	Rab5 GDP/GTP exchang	79371.22	1.51E+04	Filtered	Filtered	Filtered	Filtered	Filtered	Filtered	Filtered
P01854	IGHE	Ig epsilon chain C r	47019.03	1.51E+04	Filtered	Filtered	Filtered	Filtered	Filtered	Filtered	Filtered
P01604		Ig kappa chain V-I r	12126.61	1.51E+04	Filtered	Filtered	Filtered	26361.43164	Filtered	Filtered	Filtered
P0DJI9	SAA2	Serum amyloid A-2 pr	13527.09	1.47E+04	12654.08984	Filtered	19240.86328	4066.064697	Filtered	Filtered	Filtered
Q8WVN6	SECTM1	Secreted and transme	27039.01	1.45E+04	20406.15234	Filtered	Filtered	13395.37305	Filtered	14090.87109	Filtered
P12111	COL6A3	Collagen alpha-3(VI)	343669.37	1.45E+04	18110.47656	13187.5625	15683.05859	14385.57617	13536.85938	18021.10156	18918.84766
Q15942	ZYX	Zyxin	61277.34	1.43E+04	Filtered	Filtered	23022.26172	11629.98828	Filtered	10627.85449	29713.4082
P12109	COL6A1	Collagen alpha-1(VI)	108529.39	1.43E+04	14129.62305	12496.85742	16703.49414	12774.69531	35913.22656	18114.60938	18013.68359
Q96EE4	CCDC126	Coiled-coil domain-c	15668.18	1.42E+04	20913.07813	18842.13477	11585.65723	Filtered	19200.89648	2110.637207	Filtered

P00451	F8	Coagulation factor V	267009.38	1.41E+04	3334.830811	3953.304199	3878.087646	4483.37793	Filtered	4071.594971	166848.7656
P14923	JUP	Junction plakoglobin	81744.74	1.41E+04	3969.606201	Filtered	Filtered	Filtered	5118.794434	Filtered	Filtered
Q02224	CENPE	Centromere-associate	316415.19	1.40E+04	9400.354492	10189.3877	29948.02148	11208.53125	12853.21094	19206.58203	17984.34375
P14543	NID1	Nidogen-1	136377.03	1.40E+04	17403.94141	16790.52344	18643.08789	14133.60547	11800.77148	22103.45508	19836.37891
P13611	VCAN	Versican core protei	372820.05	1.39E+04	9691.745586	Filtered	6747.705078	6708.557617	6211.391113	55551.75391	Filtered
P01825;P06331		Ig heavy chain V-II	12790.32;16228.46	1.38E+04	5540.852539	5241.987305	3089.753418	5654.660645	Filtered	Filtered	7150.625977
O43505	B4GAT1	Beta-1,4-glucuronylt	47119.06	1.38E+04	6199.458496	13159.46094	15635.77832	12800.74414	16220.20508	8870.624023	20312.01953
P27797	CALR	Calreticulin	48141.56	1.37E+04	16063.52148	12689.00586	10089.99902	14239.89648	12286.64941	13177.96094	17618.45703
P55290	CDH13	Cadherin-13	78286.85	1.37E+04	10802.08203	13880.59375	14659.82617	10840.18652	10859.86816	13620.33691	16494.60547
Q8NBP7	PCSK9	Proprotein convertas	74286.21	1.36E+04	11271.38965	14232.94727	13261.01758	12435.6582	12554.28223	13170.9502	13162.3584
P35590	TIE1	Tyrosine-protein kin	125089.7	1.35E+04	7472.321289	6219.549805	7738.658203	17350.18164	13469.13281	27139.71875	14273.89258
P02741	CRP	C-reactive protein	2.50E+04	1.35E+04	Filtered	Filtered	1.93E+04	Filtered	Filtered	Filtered	Filtered
Q07954	LRP1	Prolow-density lipop	504606.32	1.35E+04	15820.73828	11888.29492	16360.9043	14690.38086	18805.48242	16149.82227	17103.59766
P30613	PKLR	Pyruvate kinase PKLR	61830.16	1.34E+04	Filtered	Filtered	Filtered	Filtered	Filtered	Filtered	Filtered
Q8IXL6	FAM20C	Extracellular serine	66234.49	1.34E+04	15843.02051	13417.66016	14472.50586	18072.72656	11047.69922	14822.68555	15706.12207
Q8NE86	MCU	Calcium uniporter pr	39866.85	1.32E+04	Filtered	13282.9541	Filtered	Filtered	13159.29102	Filtered	Filtered
P23526	AHCY	Adenosylhomocysteina	47716.07	1.31E+04	Filtered	Filtered	Filtered	Filtered	7660.690918	Filtered	Filtered
Q9H4A9	DPEP2	Dipeptidase 2	53305.96	1.31E+04	6472.265625	6621.056641	7844.099121	6010.666504	7442.505371	5175.182617	7304.899902
P35916	FLT4	Vascular endothelial	152757.34	1.30E+04	13218.70996	12336.33008	18239.03906	11243.59375	10913.29297	17744.4082	17047.15234
P01773		Ig heavy chain V-III	NaN	1.30E+04	Filtered	Filtered	Filtered	Filtered	Filtered	Filtered	4196.98291
P63104	YWHAZ	14-3-3 protein zeta/	27745.1	1.30E+04	Filtered	Filtered	5987.15625	Filtered	Filtered	7853.909668	10985.26367
Q8IWV2	CNTN4	Contactin-4	113453.64	1.28E+04	5734.057129	4723.348633	4318.54541	5101.841797	4854.933594	4824.158203	5535.462891
P04792	HSPB1	Heat shock protein b	22782.52	1.28E+04	Filtered	Filtered	Filtered	Filtered	Filtered	Filtered	Filtered
P07911	UMOD	Uromodulin	69760.85	1.27E+04	19139.36523	23360.44531	7789.785645	22910.07227	7030.586426	12238.62695	16288.4502
O43852	CALU	Calumenin	37106.78	1.26E+04	Filtered	Filtered	Filtered	9940.520508	11742.27832	2047.536743	Filtered
Q9H9T3	ELP3	Elongator complex pr	62258.9	1.26E+04	Filtered	Filtered	Filtered	Filtered	Filtered	Filtered	Filtered
P23528	CFL1	Cofilin-1	18502.49	1.25E+04	4637.205078	7657.496582	4814.872559	4441.617676	6825.507324	3709.765869	9508.608398
Q15223	NECTIN1	Nectin-1	57158.17	1.25E+04	13761.80664	16972.97852	9015.556641	11731.52051	14375.22754	14858.55078	15147.91504
Q8IZF2	ADGRF5	Adhesion G protein-c	149457.07	1.25E+04	12511.9873	14988.57422	17147.73633	15529.05566	11551.22168	10355.39453	18232.19727
P11166	SLC2A1	Solute carrier famil	54083.77	1.24E+04	Filtered	Filtered	Filtered	Filtered	7498.661133	Filtered	Filtered
P62937	PPIA	Peptidyl-prolyl cis-	18012.49	1.24E+04	7666.64209	10157.50879	8161.991211	12018.40918	10800.79297	9670.844727	14437.3291
P08727	KRT19	Keratin, type I cyto	44105.99	1.23E+04	Filtered	Filtered	Filtered	3145.659424	47785.44922	1694.810669	Filtered
P18065	IGFBP2	Insulin-like growth	34814.08	1.22E+04	30459.71289	22633.68945	12553.23047	21402.34375	24607.24023	15017.88477	12647.73438
P05451	REG1A	Lithostathine-1-alph	18730.98	1.22E+04	40569.36719	8892.109375	11491.95996	14005.21484	10472.32813	17953.83203	9878.430664
P04066	FUCA1	Tissue alpha-L-fucos	53688.85	1.22E+04	11957.29688	12946.38086	16604.91797	14098.04102	11446.50684	14585.71875	17147.82031
Q13477	MADCAM1	Mucosal addressin ce	40155.27	1.21E+04	Filtered	11525.45996	17005.58984	12665.37012	7741.170898	11154.22656	11551.58691
P50395	GDI2	Rab GDP dissociation	50663.24	1.20E+04	41724.10156	39742.38281	Filtered	2834.321777	6880.196289	1210.757813	10869.40625
Q99650	OSMR	Oncostatin-M-specifi	110508.51	1.20E+04	6064.393555	4056.933105	2954.93042	2852.395508	4475.022949	3683.53125	3399.279297
P12821	ACE	Angiotensin-converti	149714.85	1.20E+04	6929.628418	7194.074219	7425.654297	10230.75391	6745.709473	4985.442871	7347.974121
P00558	PGK1	Phosphoglycerate kin	44614.68	1.19E+04	9042.532227	4669.262695	6079.961914	10300.29883	12218.90332	10478.44336	11512.32031
Q06481	APLP2	Amyloid-like protein	86955.77	1.19E+04	Filtered	Filtered	Filtered	Filtered	Filtered	Filtered	Filtered
P06702	S100A9	Protein S100-A9	13241.99	1.19E+04	Filtered	Filtered	Filtered	Filtered	15769.99805	3609.386475	Filtered
P01762		Ig heavy chain V-III	NaN	1.19E+04	Filtered	Filtered	Filtered	Filtered	Filtered	Filtered	Filtered
Q15828	CST6	Cystatin-M	16511.08	1.18E+04	10647.38867	10405.2207	11730.37988	Filtered	8932.939453	8913.40332	11189.25879
P19013	KRT4	Keratin, type II cyt	57285.23	1.18E+04	Filtered	Filtered	Filtered	Filtered	Filtered	Filtered	Filtered
O15394	NCAM2	Neural cell adhesion	93046.26	1.17E+04	11141.86719	11952.59961	11574.02148	8781.982422	7081.65918	6782.17334	17742.62305
Q9Y6N7	ROBO1	Roundabout homolog 1	180929.65	1.17E+04	12062.76563	9398.762695	Filtered	Filtered	11379.59863	14569.05859	Filtered
P02730	SLC4A1	Band 3 anion transpo	101792.32	1.16E+04	Filtered	Filtered	Filtered	1946.761353	7678.509277	1402.158203	Filtered
P30041	PRDX6	Peroxiredoxin-6	25034.98	1.15E+04	7522.428223	8333.423828	3341.105713	10435.00195	12242.80859	5882.185547	9677.264648
Q9Y646	CPQ	Carboxypeptidase Q	51887.56	1.14E+04	11166.17188	9326.380859	10264.08008	13327.625	14558.73242	14365.3457	11866.75
O14498	ISLR	Immunoglobulin super	45997.31	1.12E+04	11101.83398	9892.258789	19756.22656	10381.67188	9096.402344	14850.40039	14107.34766
Q86U17	SERPINA11	Serpin A11	46989.15	1.12E+04	8438.071289	6633.461914	12724.50977	7815.134277	8600.050781	9157.556641	15805.51172
P54289	CACNA2D1	Voltage-dependent ca	124568.03	1.10E+04	13618.36328	10470.32129	8552.229492	16665.06445	9539.854492	11638.03223	13486.74609
A1L4H1	SSC5D	Soluble scavenger re	165742.73	1.09E+04	12319.49512	7494.603516	8520.232422	6007.332031	11238.76367	7632.680176	16252.17871
P16157	ANK1	Ankyrin-1	206264.77	1.08E+04	Filtered	Filtered	Filtered	Filtered	Filtered	Filtered	Filtered
Q12913	PTPRJ	Receptor-type tyrosi	145941.49	1.07E+04	16366.98145	15333.12695	12541.16406	12112.87891	8353.436523	12050	8369.498047
P05106	ITGB3	Integrin beta-3	87057.81	1.07E+04	13356.95898	5815.894531	9419.554688	5137.217773	5988.785645	7876.436523	7989.878418
Q6Q788	APOA5	Apolipoprotein A-V	41212.59	1.06E+04	11249.25684	8050.726563	9572.248047	10898.40918	7230.067871	7229.465332	7037.586914
P80188	LCN2	Neutrophil gelatinas	22588.07	1.06E+04	22404.95313	10790.94336	17819.73828	14071.01563	Filtered	15166.03418	16572.45313
P04040	CAT	Catalase	59756.17	1.06E+04	5079.064453	7396.727539	Filtered	19073.08398	6713.916504	6975.773438	12039.90723
P06732	CKM	Creatine kinase M-ty	43101.11	1.06E+04	Filtered	Filtered	4755.034668	5372.542969	3157.841797	8233.543945	5170.533203
P19022	CDH2	Cadherin-2	99809.33	1.06E+04	Filtered	Filtered	8324.782227	6013.04541	5110.478027	6839.443359	104666.3594
Q7Z7M0	MEGF8	Multiple epidermal g	303100.31	1.03E+04	11178.35059	12381.98926	12119.68848	10667.98047	8683.3125	10540.13477	12220.22852
P07900	HSP90AA1	Heat shock protein H	84659.71	1.02E+04	2332.540771	2426.951172	22420.33789	1981.485352	7489.054688	48383.71875	2415.731201
O95479	H6PD	GDH/6PGL endoplasmic	88892.68	1.02E+04	12062.24121	10221.74219	10416.87402	11383.23047	12044.43457	12185.08496	15885.19434
O15031	PLXNB2	Plexin-B2	205127.21	1.01E+04	2771.144531	3896.120117	11148.19043	31223.28125	7294.958496	3282.74707	3620.246338
P37802	TAGLN2	Transgelin-2	22391.44	1.01E+04	7991.254395	5449.448242	16606.52344	6812.364258	12533.50879	10147.6416	20553.07813
P24821	TNC	Tenascin	240853.41	9.94E+03	9955.869141	10672.16406	14648.45215	8096.352051	7562.856445	9382.554688	10030.93555
P28827	PTPRM	Receptor-type tyrosi	163682.4	9.93E+03	12716.48438	14977.81348	14076.9248	12174.76758	7797.528809	8365.716797	7998.771484
P26447	S100A4	Protein S100-A4	11728.51	9.93E+03	Filtered	Filtered	2651.608154	3851.797363	11936.95605	4035.28418	14064.09863
P11717	IGF2R	Cation-independent m	274374.82	9.85E+03	6639.830078	6302.419434	5507.147949	6802.316406	4879.828125	5033.369141	5747.536621
P05062	ALDOB	Fructose-bisphosphat	39473.02	9.84E+03	22483.44336	4390.40918	7271.240723	8091.513672	10374.71582	7628.493164	4895.819336
P01040	CSTA	Cystatin-A	11006.49	9.68E+03	11902.99805	Filtered	Filtered	7455.731445	Filtered	Filtered	Filtered
Q2T9J0	TYSND1	Peroxisomal leader p	59308.99	9.68E+03	Filtered	Filtered	Filtered	Filtered	Filtered	Filtered	Filtered
P29508	SERPINB3	Serpin B3	44564.59	9.67E+03	Filtered	Filtered	Filtered	4662.918945	14681.44922	Filtered	Filtered

P25774	CTSS	Cathepsin S	37495.67	9.64E+03	16634.10938	12583.49414	11296.70703	11396.76855	11920.2168	9836.073242	6575.489746
Q9HCU0	CD248	Endosialin	80859.24	9.63E+03	9315.736328	8033.094727	13750.78516	11540.44727	12980.77637	12650.11426	10783.65918
P10319;P30491;P30685	HLA-B	HLA class I histocom	40337.14;40495.32;40	9.61E+03	7963.556152	Filtered	11009.10254	7877.750488	Filtered	Filtered	9814.563477
Q02413	DSG1	Desmoglein-1	113747.59	9.60E+03	Filtered	1848.618652	9647.378906	8137.035156	Filtered	6040.012207	Filtered
P62826	RAN	GTP-binding nuclear	24423.1	9.57E+03	Filtered	Filtered	Filtered	Filtered	Filtered	Filtered	10503.41309
Q7Z7M8	B3GNT8	UDP-GlcNAc:betaGal b	43396	9.55E+03	8478.610352	6870.114746	7012.484863	2711.979004	Filtered	11062.62891	Filtered
P07602	PSAP	Prosaposin	58112.64	9.48E+03	11484.9873	Filtered	11069.20508	Filtered	Filtered	3396.868164	Filtered
Q8NHL6	LILRB1	Leukocyte immunoglob	70819.38	9.44E+03	Filtered	Filtered	Filtered	Filtered	Filtered	Filtered	Filtered
P11142	HSPA8	Heat shock cognate 7	70898.09	9.43E+03	6229.828125	6060.378418	4969.808105	6457.097168	10543.70801	6030.628418	7992.536133
P40189	IL6ST	Interleukin-6 recept	103536.6	9.41E+03	10277.39844	12293.00586	9442.017578	11090.80762	7695.743652	9776.230469	10765.38867
Q9UBX5	FBLN5	Fibulin-5	50180.48	9.37E+03	Filtered	7901.69043	8068.923828	10796.44434	9714.332031	8167.177246	7886.125
P19021	PAM	Peptidyl-glycine alp	108332.06	9.17E+03	42318.37891	Filtered	8933.966797	9831.331055	8893.388672	12846.89258	10686.31641
P56199	ITGA1	Integrin alpha-1	130847.78	9.15E+03	Filtered	Filtered	Filtered	Filtered	Filtered	Filtered	Filtered
Q8WZ75	ROBO4	Roundabout homolog 4	107457.42	9.10E+03	8498.732422	11675.04199	11731.22754	10538.43945	6989.483398	7762.195313	12851.74609
P14618	PKM	Pyruvate kinase PKM	57936.88	9.10E+03	6147.921387	5844.077148	6631.266602	5970.817383	11268.55273	8880.192383	8714.994141
O15511	ARPC5	Actin-related protei	16320.42	9.05E+03	7266.169922	7613.207031	13890.23828	8887.688477	Filtered	7034.619141	7577.779297
Q12794	HYAL1	Hyaluronidase-1	48368.13	9.02E+03	11147.90137	Filtered	9449.092773	Filtered	Filtered	10356.78613	Filtered
P22897	MRC1	Macrophage mannose r	166011.54	8.98E+03	7268.729004	7775.696289	9309.883789	9593.850586	8269.427734	10180.29199	10921.92188
P32004	L1CAM	Neural cell adhesion	140002.79	8.94E+03	2824.331055	25244.61719	4173.925781	3800.399902	18265.63086	2721.976563	2879.548096
P16112	ACAN	Aggrecan core protei	250193.19	8.80E+03	7712.639648	8545.546875	8688.770508	9982.628906	10637.44922	Filtered	9710.124023
P61224;P62834	RAP1B;RAP1A	Ras-related protein	20824.78;20987.18	8.80E+03	Filtered	7422.295898	Filtered	Filtered	8592.339844	8520.269531	Filtered
P12035	KRT3	Keratin, type II cyt	64416.99	8.76E+03	Filtered	Filtered	Filtered	Filtered	Filtered	Filtered	Filtered
Q96RD9	FCRL5	Fc receptor-like pro	106437.23	8.75E+03	3437.202881	3411.179688	6159.738281	4298.402832	2871.868652	Filtered	7393.453125
P11684	SCGB1A1	Uteroglobin	9993.74	8.71E+03	11068.60938	Filtered	9253.070313	9519.206055	Filtered	9241.913086	8956.900391
P62258	YWHAE	14-3-3 protein epsil	29173.89	8.66E+03	Filtered	10812.99805	Filtered	5729.549316	17972.0957	5033.538086	14354.53906
O15204	ADAMDEC1	ADAM DEC1	52775.42	8.61E+03	12997.72363	9611.145508	7797.502441	8342.764648	6670.486328	7321.0625	8845.395508
P08123	COL1A2	Collagen alpha-2(I)	129313.89	8.60E+03	Filtered	Filtered	Filtered	Filtered	15248.70117	4432.822266	Filtered
P00568	AK1	Adenylate kinase iso	21634.84	8.57E+03	4958.284668	2204.182129	Filtered	9393.130859	6579.239746	3064.155273	8303.725586
P08567	PLEK	Pleckstrin	40124.85	8.40E+03	4542.209473	Filtered	4662.805664	4337.518066	Filtered	5827.942383	7410.131836
P0DMV8;P0DMV9;P34931	HSPA1A;HSPA1B;HSPA1L	Heat shock 70 kDa pr	70052.23;70052.23;70	8.37E+03	7603.074219	7431.466797	Filtered	8188.646973	8645.558594	6738.355957	Filtered
P07942	LAMB1	Laminin subunit beta	198038.36	8.34E+03	15309.26465	11014.95996	7179.848633	9333.384766	12074.37988	8994.802734	10895.76855
Q9Y274	ST3GAL6	Type 2 lactosamine a	38213.62	8.31E+03	Filtered	Filtered	6631.040039	Filtered	10656.2041	10475.21875	Filtered
P80511	S100A12	Protein S100-A12	10575.04	8.28E+03	Filtered	Filtered	Filtered	Filtered	Filtered	3558.824463	Filtered
O94985	CLSTN1	Calsyntenin-1	109792.75	8.23E+03	7036.65918	8701.504883	7677.745117	9759.830078	8480.794922	6256.964844	7903.146484
P39060	COL18A1	Collagen alpha-1(XVI	178187.66	8.21E+03	12714.34766	10119.3125	10118.16406	8575.287109	7687.07373	9374.493164	7441.09668
P17931	LGALS3	Galectin-3	26152.33	8.20E+03	9857.642578	11926.13574	9572.644531	8737.890625	9134.81543	9136.325195	8135.525391
P07307	ASGR2	Asialoglycoprotein r	35092.21	8.19E+03	7474.842773	9003.623047	7739.14502	10922.41211	6007.569336	9317.431641	10207.44922
Q92859	NEO1	Neogenin	160016.54	8.18E+03	13053.52344	6044.114746	12029.68848	9301.620117	9343.855469	12200.09961	13005.1084
P21926	CD9	CD9 antigen	25416.05	8.18E+03	Filtered	3981.986572	6597.369141	3982.872314	Filtered	5394.29248	10358.44727
P13987	CD59	CD59 glycoprotein	14177.28	8.16E+03	9100.476563	9738.232422	10105.04297	7526.339844	9805.09375	10936.78125	9494.013672
P05534	HLA-A	HLA class I histocom	40688.55	8.15E+03	Filtered	Filtered	Filtered	Filtered	Filtered	Filtered	Filtered
Q8IUL8	CILP2	Cartilage intermedia	126291.36	8.12E+03	Filtered	6129.753418	11180.48145	7522.408203	9825.910156	10045.50781	10489.83887
P68363;Q71U36;Q9BQE3	TUBA1B;TUBA1A;TUBA1C	Tubulin alpha-1B cha	50151.63;50135.63;49	8.12E+03	6360.069824	8308.036133	7900.248047	4747.871582	15138.24805	7383.262695	11207.44141
P15291	B4GALT1	Beta-1,4-galactosylt	43920.27	8.10E+03	8682.320313	10228.61328	11162.06836	8286.583984	Filtered	11824.63184	9905.825195
P08833	IGFBP1	Insulin-like growth	27903.66	8.07E+03	17426.33398	Filtered	Filtered	Filtered	10261.78516	Filtered	2999.108643
P09105	HBQ1	Hemoglobin subunit t	15507.78	8.06E+03	Filtered	Filtered	Filtered	Filtered	Filtered	Filtered	Filtered
P05121	SERPINE1	Plasminogen activato	45059.98	8.06E+03	5762.347168	6607.533203	9653.525391	6981.239258	9533.018555	8149.162109	8948.705078
P01600;P01610;P80362		Ig kappa chain V-I r	11670.97;11840.2;117	7.99E+03	Filtered	Filtered	Filtered	Filtered	Filtered	Filtered	Filtered
O00592	PODXL	Podocalyxin	58635.25	7.99E+03	7398.281738	9886.263672	8383.368164	Filtered	Filtered	6603.609375	9307.776367
Q13332	PTPRS	Receptor-type tyrosi	217041.35	7.99E+03	2975.069824	19160.25781	3651.516846	4694.963867	Filtered	5196.381348	5263.669434
P22392	NME2	Nucleoside diphospha	17298.03	7.96E+03	6572.272949	Filtered	Filtered	8552.154297	10008.59473	4952.133789	6312.678223
Q14574	DSC3	Desmocollin-3	99968.98	7.93E+03	9461.790039	6949.37207	7670.666016	4861.601563	6277.685059	7864.338379	10747.79102
A2RTY3	HEATR9	Protein HEATR9	65680.89	7.87E+03	Filtered	Filtered	2155.177002	Filtered	Filtered	Filtered	3304.489502
P05164	MPO	Myeloperoxidase	83868.57	7.84E+03	18612.63086	7898.668945	12689.91797	6367.704102	Filtered	10442.2373	6745.381348
Q15848	ADIPOQ	Adiponectin	26413.64	7.82E+03	46372.65234	18985.30664	8065.810547	11731.11328	8766.342773	7134.025391	13141.71094
P01137	TGFB1	Transforming growth	44341.15	7.82E+03	7094.959961	4922.810547	10399.36035	8217.897461	4872.262207	10460.80762	11140.72363
P55774	CCL18	C-C motif chemokine	9848.77	7.80E+03	8755.882813	Filtered	6646.128418	14320.1123	1637.282104	10397.32129	7018.762695
Q8NG11	TSPAN14	Tetraspanin-14	30690.83	7.75E+03	3686.511475	Filtered	2501.213379	Filtered	Filtered	Filtered	Filtered
Q9UBR2	CTSZ	Cathepsin Z	33867.93	7.74E+03	7743.458496	8074.614746	9658.666016	5002.70752	15856.08301	9509.401367	10688.6748
P01742;P01761;P06326		Ig heavy chain V-I r	12472;13732.48;13579	7.68E+03	Filtered	Filtered	Filtered	Filtered	Filtered	Filtered	Filtered
P05556	ITGB1	Integrin beta-1	88415.27	7.58E+03	6047.93457	5613.246094	6884.557129	5348.062012	9118.802734	5659.785156	5996.05127
P02461	COL3A1	Collagen alpha-1(III	138564.28	7.57E+03	Filtered	Filtered	Filtered	Filtered	14573.07813	Filtered	Filtered
P12814	ACTN1	Alpha-actinin-1	103057.55	7.57E+03	3847.122559	Filtered	Filtered	Filtered	8570.608398	3586.246582	7154.526855
Q13740	ALCAM	CD166 antigen	65102.28	7.49E+03	12865.97754	9245.740234	10285.91602	10097.5	7065.789063	9591.666016	10837.05469
P08311	CTSG	Cathepsin G	28837.21	7.28E+03	10398.95605	Filtered	5478.557129	Filtered	Filtered	7272.291992	Filtered
P51153;P59190;P61006	RAB13;RAB15;RAB8A;RA	Ras-related protein	22774.12;24390.62;23	7.26E+03	Filtered	Filtered	Filtered	Filtered	Filtered	Filtered	11179.8125
P01605		Ig kappa chain V-I r	11834.15	7.24E+03	Filtered	Filtered	Filtered	Filtered	Filtered	Filtered	Filtered
P23284	PPIB	Peptidyl-prolyl cis-	23742.53	7.17E+03	4211.347656	6855.199707	8758.205078	7151.472656	7417.899902	7162.397461	8491.203125
P14174	MIF	Macrophage migration	12476.29	7.17E+03	Filtered	Filtered	Filtered	3335.17334	Filtered	Filtered	2994.640137
Q5T749	KPRP	Keratinocyte proline	64135.73	7.16E+03	2601.874023	3058.121094	9371.841797	2630.350586	Filtered	4834.115723	Filtered
P20023	CR2	Complement receptor	112915.83	7.12E+03	8478.870117	7683.318848	9259.413086	3973.493652	5741.008301	10354.96484	5375.762207
Q9UIB8	CD84	SLAM family member 5	38782.1	7.11E+03	6996.453613	7285.664551	5429.194336	5792.704102	6452.604492	5960.493164	8440.886719
P12110	COL6A2	Collagen alpha-2(VI)	108579.43	7.10E+03	7274.184082	8728.955078	9163.952148	5331.261719	7495.787109	6724.666016	9709.813477
Q8TDL5	BPIFB1	BPI fold-containing	52441.54	7.00E+03	Filtered	Filtered	Filtered	Filtered	Filtered	Filtered	Filtered
Q92854	SEMA4D	Semaphorin-4D	96149.9	6.98E+03	6167.716309	Filtered	7113.836426	7419.353027	Filtered	6307.655273	5345.61377

P58335	ANTXR2	Anthrax toxin recept	53666.17	6.97E+03	8167.143555	6190.773438	13531.83105	7862.04248	Filtered	8120.09082	8125.245117
Q13228	SELENBP1	Selenium-binding pro	52390.97	6.76E+03	Filtered	6221.632324	Filtered	6725.214355	2283.395996	4603.290527	Filtered
Q8WWZ8	OIT3	Oncoprotein-induced	60021.83	6.74E+03	6556.52832	7679.225098	6910.476074	8543.103516	7336.188965	6318.452637	6610.604004
Q86TH1	ADAMTSL2	ADAMTS-like protein	104620.89	6.68E+03	4355.374023	4749.754395	5827.455078	5606.302734	4397.691895	4852.472656	9076.458008
Q9HCN6	GP6	Platelet glycoprotei	36866.01	6.66E+03	Filtered	Filtered	Filtered	Filtered	Filtered	Filtered	9046.163086
Q9H4G4	GLIPR2	Golgi-associated pla	17218.3	6.65E+03	4430.294434	5155.74707	6779.724609	6223.398438	9794.413086	6420.027344	8410.773438
Q92820	GGH	Gamma-glutamyl hydro	35964.37	6.64E+03	10378.95703	5382.330078	5472.695801	8021.114746	5510.834473	6593.942383	4646.839844
P10768	ESD	S-formylglutathione	31462.79	6.54E+03	Filtered	Filtered	Filtered	5614.206055	Filtered	Filtered	Filtered
P08514	ITGA2B	Integrin alpha-IIb	113376.87	6.47E+03	4529.566406	Filtered	Filtered	Filtered	Filtered	5299.624023	Filtered
P01775		Ig heavy chain V-III	NaN	6.46E+03	Filtered	Filtered	Filtered	Filtered	Filtered	Filtered	Filtered
Q8NBJ4	GOLM1	Golgi membrane prote	45333.24	6.30E+03	4061.833008	Filtered	4997.230469	3367.024902	Filtered	4363.707031	Filtered
Q12864	CDH17	Cadherin-17	92219.33	6.21E+03	Filtered	2984.087158	Filtered	Filtered	Filtered	Filtered	Filtered
P04211		Ig lambda chain V re	12379.94	6.21E+03	Filtered	Filtered	Filtered	2217.080322	1074.834961	Filtered	Filtered
Q9HD89	RETN	Resistin	11419.34	6.19E+03	3759.103271	5054.844238	3016.155762	Filtered	Filtered	Filtered	Filtered
P10912	GHR	Growth hormone recep	71499.5	6.13E+03	8949.232422	12810.43359	8865.172852	3474.327881	4280.21875	7078.145996	5476.950684
Q9NZ08	ERAP1	Endoplasmic reticulu	107234.8	6.12E+03	Filtered	Filtered	6892.161133	5637.078125	Filtered	5165.353027	8611.029297
P37837	TALDO1	Transaldolase	37540.12	6.11E+03	Filtered	3924.012451	Filtered	5844.9375	8142.684082	5723.167969	11602.72266
P24387	CRHBP	Corticotropin-releas	36144.35	6.07E+03	6978.78418	Filtered	Filtered	5487.582031	Filtered	8110.99707	Filtered
Q96NZ9	PRAP1	Proline-rich acidic	17207.68	6.03E+03	Filtered	5241.858887	5521.38623	5272.377441	Filtered	5902.402832	5504.010254
P52209	PGD	6-phosphogluconate d	53139.98	6.03E+03	Filtered	Filtered	Filtered	Filtered	Filtered	Filtered	Filtered
A0M8Q6	IGLC7	Ig lambda-7 chain C	11302.67	5.98E+03	Filtered	Filtered	Filtered	Filtered	Filtered	Filtered	Filtered
Q8IZP9	ADGRG2	Adhesion G-protein c	111592.54	5.90E+03	8537.716797	5866.723633	Filtered	7239.223633	Filtered	6240.060059	6422.780762
Q86SQ4	GPR126	G-protein coupled re	136694.61	5.85E+03	10324.90332	7563.395996	7108.796875	5853.501465	5969.617188	6524.321289	Filtered
Q8N149	LILRA2	Leukocyte immunoglob	52991.72	5.82E+03	Filtered	6123.73584	Filtered	Filtered	Filtered	Filtered	5392.76123
O75015	FCGR3B	Low affinity immunog	26215.85	5.77E+03	Filtered	Filtered	Filtered	Filtered	Filtered	4320.411621	2109.922119
Q93063	EXT2	Exostosin-2	82254.53	5.76E+03	51164.42578	5337.918945	4844.969727	6295.537109	5130.800781	5652.080078	6430.239746
P08493	MGP	Matrix Gla protein	12353.19	5.76E+03	Filtered	3652.771729	Filtered	Filtered	Filtered	6866.817383	3635.659424
P50454	SERPINH1	Serpin H1	46440.54	5.74E+03	Filtered	Filtered	Filtered	Filtered	5743.78418	Filtered	Filtered
Q5VY43	PEAR1	Platelet endothelial	110666.2	5.72E+03	7076.947266	4443.873047	8472.501953	4696.533203	Filtered	3804.748047	4976.852539
Q6GTS8	PM20D1	Probable carboxypept	55741.27	5.66E+03	7388.120605	Filtered	5450.461914	Filtered	Filtered	Filtered	6956.194336
Q14623	IHH	Indian hedgehog prot	45250.67	5.64E+03	Filtered	6056.553223	5134.310547	6543.98877	Filtered	Filtered	7988.105957
Q08554	DSC1	Desmocollin-1	99986.83	5.63E+03	5175.0625	4570.73584	5187.512695	1742.962891	Filtered	3742.147705	5706.995117
P09619	PDGFRB	Platelet-derived gro	123968.03	5.63E+03	8541.931641	7530.662109	8280.761719	2921.753174	Filtered	4054.915771	5574.246094
Q86UX7	FERMT3	Fermitin family homo	75952.8	5.61E+03	5139.92041	Filtered	Filtered	4095.141113	Filtered	5605.345703	5615.507324
P40925	MDH1	Malate dehydrogenase	36426.12	5.58E+03	1965.825317	Filtered	Filtered	2786.869873	6067.511719	Filtered	Filtered
P63241;Q6IS14;Q9GZV4	EIF5A;EIF5AL1;EIF5A2	Eukaryotic translati	16832.25;16773.17;16	5.54E+03	3703.380615	4186.338867	3499.685547	6117.963867	8732.12207	5191.649902	7255.95459
Q9UBX1	CTSF	Cathepsin F	53365.9	5.52E+03	6219.097656	Filtered	Filtered	6107.633789	3500.756104	8078.344727	Filtered
Q96J42	TXNDC15	Thioredoxin domain-c	39885.2	5.50E+03	Filtered	3386.520996	Filtered	6502.824707	Filtered	Filtered	7313.682129
P09382	LGALS1	Galectin-1	14715.7	5.46E+03	4105.95459	5379.369629	5737.275879	4701.333984	3719.449951	5160.503906	6402.585449
Q86UN3	RTN4RL2	Reticulon-4 receptor	46105.67	5.45E+03	Filtered	5219.709961	4727.529297	6157.638184	Filtered	Filtered	Filtered
P11150	LIPC	Hepatic triacylglyce	55914.49	5.44E+03	Filtered	Filtered	9061.53125	5705.415039	Filtered	Filtered	5973.676758
O00151	PDLIM1	PDZ and LIM domain p	36071.71	5.44E+03	Filtered	Filtered	7020.134277	Filtered	2793.227051	3245.76709	9256.977539
Q8IYS5	OSCAR	Osteoclast-associate	30480.96	5.44E+03	4857.827637	6346.209961	4981.978516	Filtered	Filtered	8061.558105	Filtered
Q9HBR0	SLC38A10	Putative sodium-coup	119762.23	5.39E+03	Filtered	4424.849609	4861.233887	3155.019531	4955.387695	6339.979492	Filtered
Q9NZD4	AHSP	Alpha-hemoglobin-sta	11840.41	5.36E+03	Filtered	Filtered	Filtered	Filtered	Filtered	Filtered	Filtered
P26641	EEF1G	Elongation factor 1-	50118.81	5.35E+03	Filtered	Filtered	Filtered	Filtered	5352.654297	Filtered	Filtered
Q14956	GPNMB	Transmembrane glycop	63922.54	5.34E+03	7337.23584	6684.766602	9712.584961	3298.859619	Filtered	4984.63916	5506.505859
P07738	BPGM	Bisphosphoglycerate	30005.21	5.25E+03	2797.940918	Filtered	Filtered	4140.572266	2662.348145	Filtered	Filtered
O00299	CLIC1	Chloride intracellul	26922.72	5.25E+03	Filtered	Filtered	Filtered	Filtered	Filtered	Filtered	Filtered
P06733	ENO1	Alpha-enolase	47168.96	5.23E+03	Filtered	Filtered	Filtered	1992.11084	Filtered	3030.562012	1671.779053
P10586	PTPRF	Receptor-type tyrosi	212878.88	5.23E+03	5411.926758	4872.82666	5733.174805	5350.254395	6406.614258	6368.358398	5302.469727
O00462	MANBA	Beta-mannosidase	100895.17	5.20E+03	2556.37793	2537.699951	Filtered	3884.886963	Filtered	4477.858398	3248.106201
P10599	TXN	Thioredoxin	11737.49	5.16E+03	3766.053955	4663.132813	3821.900879	4773.874023	6183.686523	3171.592285	6192.854492
P16109	SELP	P-selectin	90833.88	5.14E+03	2768.814697	6852.585938	7211.075195	4084.836914	5532.676758	3443.40625	8576.429688
Q8TER0	SNED1	Sushi, nidogen and E	152203.74	5.14E+03	6875.743164	6698.768555	4475.446777	5530.098633	4845.535156	5302.503418	6316.054688
P16035	TIMP2	Metalloproteinase in	24399.25	5.09E+03	3064.817139	Filtered	Filtered	Filtered	8074.957031	Filtered	Filtered
P06318;P06319		Ig lambda chain V-VI	11965.96;14146.72	5.09E+03	Filtered	Filtered	Filtered	Filtered	Filtered	Filtered	Filtered
Q9H6X2	ANTXR1	Anthrax toxin recept	62788.97	5.01E+03	2010.930176	3937.626221	9108.916016	4302.30957	3131.915527	4096.138672	3586.69751
P32942	ICAM3	Intercellular adhesi	59540.78	4.99E+03	3747.241455	Filtered	Filtered	4519.495117	4220.294434	4517.80957	8350.6875
P09960	LTA4H	Leukotriene A-4 hydr	69285.27	4.97E+03	5287.876465	Filtered	4065.961426	5278.825684	Filtered	6685.245117	6312.383789
P29279	CTGF	Connective tissue gr	38091.43	4.96E+03	3259.939697	5389.055176	Filtered	4073.332764	Filtered	Filtered	4088.100342
P00441	SOD1	Superoxide dismutase	15935.74	4.92E+03	3201.504395	Filtered	Filtered	4812.805176	18230.9082	2603.034668	Filtered
Q9C0K7	STRADB	STE20-related kinase	47026.39	4.89E+03	Filtered	2500.45752	4094.975098	5061.681641	Filtered	5142.982422	3251.606201
Q2TV78	MST1L	Putative macrophage	79694.14	4.83E+03	18345.05664	7474.893555	4209.359863	3430.936523	Filtered	5495.057129	4611.931641
P55899	FCGRT	IgG receptor FcRn la	39743.35	4.82E+03	Filtered	Filtered	Filtered	Filtered	Filtered	Filtered	6684.462891
O75594	PGLYRP1	Peptidoglycan recogn	21730.78	4.81E+03	8753.291016	5489.292969	8743.595703	2825.401367	1956.564941	6721.366211	5189.728027
P13716	ALAD	Delta-aminolevulinic	36294.87	4.75E+03	1953.009766	Filtered	Filtered	5175.660156	3933.543457	2604.0896	2047.619019
P05089	ARG1	Arginase-1	34734.94	4.66E+03	Filtered	Filtered	Filtered	3590.017334	Filtered	Filtered	Filtered
Q8N6C8	LILRA3	Leukocyte immunoglob	47471.78	4.57E+03	Filtered	6782.961426	Filtered	2781.091064	Filtered	Filtered	Filtered
P26572	MGAT1	Alpha-1,3-mannosyl-g	50878.36	4.55E+03	4681.114746	3961.275879	5680.432617	Filtered	Filtered	6629.610352	3947.270752
Q9NPG4	PCDH12	Protocadherin-12	128994.59	4.52E+03	4068.899902	5906.995117	5897.934082	4050.145752	7184.019531	4421.625977	4283.790039
P10619	CTSA	Lysosomal protective	54466.07	4.51E+03	2936.481934	5762.034668	5150.404785	4242.508789	3635.916504	3923.55249	5527.698242
P80419		Ig heavy chain V-III	13087.41	4.50E+03	Filtered	Filtered	Filtered	Filtered	Filtered	Filtered	Filtered
Q07075	ENPEP	Glutamyl aminopeptid	109244.43	4.49E+03	4724.478027	4408.353516	4181.794922	5311.313965	Filtered	3224.882324	4193.429688
P60022	DEFB1	Beta-defensin 1	7419.7	4.46E+03	4298.208984	5825.37793	Filtered	6252.490234	Filtered	3596.162598	3610.613525

P38571	LIPA	Lysosomal acid lipas	45419.01	4.43E+03	Filtered	Filtered	Filtered	Filtered	Filtered	Filtered	3086.144043
Q13404	UBE2V1	Ubiquitin-conjugatin	16494.95	4.35E+03	Filtered	Filtered	Filtered	Filtered	Filtered	Filtered	Filtered
Q9NQ38	SPINK5	Serine protease inhi	120714.46	4.34E+03	2715.794434	Filtered	Filtered	4789.896484	5527.950195	3289.798828	Filtered
P30101	PDIA3	Protein disulfide-is	56782.38	4.26E+03	Filtered	Filtered	Filtered	5077.088379	Filtered	2100.307617	4568.246582
P08246	ELANE	Neutrophil elastase	28518.06	4.19E+03	Filtered	Filtered	Filtered	Filtered	Filtered	1842.988403	Filtered
O75356	ENTPD5	Ectonucleoside triph	47517.29	4.19E+03	Filtered	Filtered	5342.314453	Filtered	Filtered	Filtered	Filtered
O75223	GGCT	Gamma-glutamylcyclot	21007.72	4.13E+03	Filtered	Filtered	Filtered	Filtered	Filtered	Filtered	Filtered
P14209	CD99	CD99 antigen	18847.87	4.12E+03	Filtered	6373.587891	7393.197266	Filtered	Filtered	Filtered	4416.796875
Q6E0U4	DMKN	Dermokine	47082.42	4.11E+03	Filtered	4616.952148	3818.165771	4680.129883	1725.769897	3454.733398	3290.107666
O95236	APOL3	Apolipoprotein L3	44278.2	4.09E+03	6396.374512	Filtered	Filtered	Filtered	3692.457031	Filtered	Filtered
O14773	TPP1	Tripeptidyl-peptidas	61247.93	4.08E+03	Filtered	Filtered	Filtered	Filtered	Filtered	Filtered	Filtered
P09603	CSF1	Macrophage colony-st	60179.46	4.06E+03	Filtered	Filtered	4317.405273	Filtered	Filtered	Filtered	Filtered
Q13361	MFAP5	Microfibrillar-assoc	19611.53	4.05E+03	3102.800049	3722.256592	Filtered	3938.057861	Filtered	4839.856445	4297.250488
P26992	CNTFR	Ciliary neurotrophic	40633.17	4.02E+03	3494.614746	2236.532715	Filtered	5096.468262	6571.456543	6653.047852	3554.241943
Q00341	HDLBP	Vigilin	141455.64	4.01E+03	3413.093262	Filtered	Filtered	Filtered	Filtered	Filtered	Filtered
Q8IW75	SERPINA12	Serpin A12	47175	3.95E+03	Filtered	Filtered	Filtered	Filtered	Filtered	Filtered	Filtered
Q9BS26	ERP44	Endoplasmic reticulu	46971.19	3.91E+03	4915.139648	3388.531494	3628.416504	3384.415771	2594.304688	5924.822266	2421.716064
Q6UXH0	ANGPTL8	Angiopoietin-like pr	22105.39	3.85E+03	Filtered	Filtered	Filtered	Filtered	Filtered	Filtered	Filtered
O00602	FCN1	Ficolin-1	35078.4	3.83E+03	Filtered	Filtered	Filtered	Filtered	Filtered	Filtered	Filtered
P23141	CES1	Liver carboxylestera	62521.07	3.80E+03	Filtered	Filtered	3002.473633	Filtered	Filtered	3543.470703	Filtered
P60660	MYL6	Myosin light polypep	16930.05	3.78E+03	1482.561035	Filtered	5140.306152	Filtered	3917.920654	3185.443848	4293.169434
Q04721	NOTCH2	Neurogenic locus not	265404.78	3.76E+03	4396.445801	6272.037109	3671.213623	4392.131348	6125.821289	3497.271973	4273.827148
Q9NY15	STAB1	Stabilin-1	275482.19	3.71E+03	3151.937744	5034.487793	3312.227295	3746.25708	3364.833496	4235.304199	4087.825684
Q14112	NID2	Nidogen-2	151253.79	3.63E+03	Filtered	Filtered	Filtered	Filtered	Filtered	Filtered	Filtered
Q8NBS9	TXNDC5	Thioredoxin domain-c	47628.86	3.62E+03	Filtered	Filtered	Filtered	Filtered	Filtered	Filtered	Filtered
P18850	ATF6	Cyclic AMP-dependent	74584.64	3.62E+03	3856.169434	4135.105957	4319.090332	5438.08252	3296.591553	3102.244873	4527.304199
Q99941	ATF6B	Cyclic AMP-dependent	76709.18	3.61E+03	2685.750244	3182.068359	Filtered	5895.992676	Filtered	1908.828003	3300.581787
P16671	CD36	Platelet glycoprotei	53053.36	3.54E+03	3537.760986	Filtered	Filtered	Filtered	Filtered	Filtered	Filtered
Q99435	NELL2	Protein kinase C-bin	91346.37	3.48E+03	Filtered	Filtered	Filtered	Filtered	3481.050537	Filtered	Filtered
Q9UEW3	MARCO	Macrophage receptor	52657.96	3.46E+03	1792.210083	2129.03833	3307.460205	1620.253296	2684.814941	3308.568604	Filtered
P09668	CTSH	Pro-cathepsin H	37393.63	3.37E+03	4515.874512	4739.751953	4499.374512	1657.471802	2402.885498	3654.580322	3615.6604
P54764	EPHA4	Ephrin type-A recept	109859.97	3.33E+03	2825.012695	3037.25708	3600.112061	3569.934326	2082.448975	3337.298096	3008.828369
P19256	CD58	Lymphocyte function-	28147.38	3.28E+03	4779.40625	4189.62207	2405.396484	3085.266357	Filtered	3613.787109	4609.835449
Q9UBP4	DKK3	Dickkopf-related pro	38390.24	3.19E+03	3767.715088	Filtered	Filtered	Filtered	Filtered	4083.333984	Filtered
P00533	EGFR	Epidermal growth fac	134277.39	3.10E+03	6819.369141	4473.461914	4383.416504	4183.925781	5987.761719	2998.818115	3908.575439
Q06828	FMOD	Fibromodulin	43178.79	3.04E+03	Filtered	Filtered	Filtered	Filtered	11702.29883	986.453064	Filtered
P15090	FABP4	Fatty acid-binding p	14718.88	3.03E+03	Filtered	Filtered	Filtered	Filtered	Filtered	Filtered	Filtered
P17301	ITGA2	Integrin alpha-2	129295.44	3.03E+03	3033.112305	3974.381836	4365.299805	2233.743164	1686.256348	3050.354736	Filtered
P15531	NME1	Nucleoside diphospha	17148.72	3.02E+03	Filtered	Filtered	Filtered	Filtered	5674.449707	Filtered	Filtered
P07108	DBI	Acyl-CoA-binding pro	10044.42	2.99E+03	Filtered	Filtered	Filtered	Filtered	Filtered	1811.248657	2407.212402
P30740	SERPINB1	Leukocyte elastase i	42741.8	2.98E+03	Filtered	Filtered	Filtered	Filtered	Filtered	2525.992188	Filtered
O15143	ARPC1B	Actin-related protei	40949.78	2.94E+03	Filtered	Filtered	1142.09729	2367.316162	2576.494141	4084.083008	Filtered
P01236	PRL	Prolactin	25875.83	2.90E+03	2984.468994	Filtered	Filtered	Filtered	2451.671387	Filtered	Filtered
P14550	AKR1A1	Alcohol dehydrogenas	36573.04	2.79E+03	Filtered	Filtered	Filtered	Filtered	2789.479492	Filtered	Filtered
Q03167	TGFBR3	Transforming growth	93499.36	2.79E+03	Filtered	Filtered	Filtered	Filtered	2975.120361	2602.791016	Filtered
Q14697	GANAB	Neutral alpha-glucos	106873.98	2.74E+03	Filtered	Filtered	Filtered	3549.697266	3918.420898	Filtered	Filtered
Q9Y240	CLEC11A	C-type lectin domain	35694.63	2.57E+03	Filtered	Filtered	2603.618652	Filtered	8294.459961	Filtered	Filtered
Q13057	COASY	Bifunctional coenzym	62328.84	2.54E+03	Filtered	1176.804077	Filtered	Filtered	Filtered	Filtered	Filtered
Q9P232	CNTN3	Contactin-3	112882.72	2.54E+03	1138.134521	2763.1875	2913.195313	3212.920654	Filtered	Filtered	Filtered
Q9UKU6	TRHDE	Thyrotropin-releasin	116999.63	2.50E+03	Filtered	Filtered	Filtered	Filtered	Filtered	3293.267578	Filtered
P01719		Ig lambda chain V-V	11342.51	2.45E+03	Filtered	Filtered	Filtered	Filtered	Filtered	Filtered	Filtered
O43493	TGOLN2	Trans-Golgi network	51112.8	2.44E+03	Filtered	Filtered	2823.099854	2712.43042	835.668335	3265.343262	2533.25
Q9H095	IQCG	IQ domain-containing	51917.64	2.32E+03	2548.396729	Filtered	Filtered	1485.96875	Filtered	2977.646484	Filtered
P31146	CORO1A	Coronin-1A	51026.25	2.20E+03	1785.969727	Filtered	846.4710693	1574.225708	2061.981934	1883.432739	961.8251953
Q6ZVX7	NCCRP1	F-box only protein 5	30847.05	2.15E+03	Filtered	Filtered	Filtered	3156.779053	Filtered	Filtered	Filtered
P12273	PIP	Prolactin-inducible	16572.47	2.12E+03	Filtered	Filtered	Filtered	Filtered	Filtered	Filtered	Filtered
P24158	PRTN3	Myeloblastin	27807.03	2.10E+03	2213.84668	Filtered	Filtered	Filtered	Filtered	1122.507813	Filtered
P52565	ARHGDIA	Rho GDP-dissociation	23207.1	2.08E+03	Filtered	Filtered	Filtered	Filtered	Filtered	Filtered	Filtered
Q13449	LSAMP	Limbic system-associ	37393.49	1.86E+03	Filtered	Filtered	Filtered	1503.658813	Filtered	1738.5	2391.658203
Q9Y279	VSIG4	V-set and immunoglob	43987.06	1.41E+03	Filtered	Filtered	Filtered	Filtered	Filtered	2793.430908	Filtered
Q9BQ51	PDCD1LG2	Programmed cell deat	30956.88	1.08E+03	Filtered	Filtered	Filtered	Filtered	Filtered	Filtered	Filtered

Con_8	Con_9	Con_10	Con_11	Con_12	Con_13	Con_14	Con_15	Con_16	Con_17	Con_18	Con_19
115241904	92435912	138445264	75366320	185736656	73749976	84610336	110191128	63211268	68991632	69293880	109049392
135639680	112423992	125916608	116044448	70000576	110338192	116926720	102243904	95731288	111010984	111310344	112286752
32062430	28474504	40456624	37490588	33910560	44686920	35140024	33670040	35789628	31115348	31699700	35107880
30195726	28514298	34130464	35275376	23251872	25842028	18866578	28795798	28067146	32219910	25316408	22786146
21996540	19819438	27765400	21993830	17962848	21853040	19437962	19464912	22523538	23109088	20318116	26317040
17922056	18679976	24080544	23962224	16953992	21787082	17525238	18824196	19502500	19696658	20430468	21280342
14640489	14190620	15408199	16510176	15076940	21112288	17228782	19403368	18542200	14801221	15990362	19420602
18970968	17027166	17831232	15236226	17543112	17231406	18229436	18991784	15931509	17439254	16973054	17593190
14387127	13369300	14911647	15502330	12429472	14860590	12775209	11944194	12329960	15024846	15166545	16351624
12112078	12511740	16565346	14324474	13875837	16933952	15139847	11451715	13874456	13270435	13525678	15467538
10437887	9447852	12308811	10811600	8715909	12130160	8318409.5	8436905	8693272	11596729	11377218	12085920
7925873.5	11166011	11617378	15499124	5382356	9738512	11675808	8789174	11215576	16551500	10102714	8191337.5
9503302	10354962	9574925	11034350	9538055	11374008	9651245	9857884	11361296	10718508	9492505	10625977
8674529	9733607	10210438	9578576	10050075	10189606	8685463	7987708.5	9632197	11027511	8982931	12235516
6264965.5	9214009	11695375	6643541	7404764.5	7727538	8156222	10399184	10858614	9571643	7712732.5	5982149
10757863	8940630	12686349	10967508	5579385	7890580.5	9792255	8014037	7766242.5	5683684.5	5163997.5	11640576
9493447	8570692	11278147	8935534	7977380.5	9762457	9428327	7122880	9089429	8993034	9093044	8914364
8070900.5	8046647	9991042	7788137	7250585.5	12328571	8379390	8770122	7761057.5	7917790.5	8073655	9302716
8662066	8648917	9720452	8437307	9674052	9917811	7169161.5	7607158	8076579	10367519	8151470.5	10487066
18883142	13532719	59472.94141	28356.9668	18788096	28629.57422	14987100	21820774	31736.55273	14454219	38759.43359	23503644
7187016	7237248.5	9477139	6137908.5	6591254	7076374	8448155	8934450	6928633	5588451	7208385.5	7625874
7777836.5	7546790.5	8915409	9209330	8001637.5	9133360	8094110.5	7974306	7476631.5	7001553.5	7038112	7571048
4775941.5	7461106	9603248	4561204.5	5795707	5453404.5	5855471	8817083	8322543	6769357.5	6484926	4685361
10093103	10080216	5290188.5	5901742	6603976.5	7583956.5	5889197	6084297	4557560.5	4585756	5470736	6024198.5
6698417.5	6883712.5	8820166	9293890	7232554	5857124.5	6454979.5	5119192.5	7775033.5	7697213	6092933	7997735
6633646	6480631.5	7869143.5	7564994	6137169	8232470	6580684	6980851	7253900.5	6155366.5	6790317.5	8983602
6712091.5	6389285	8359686.5	6398653.5	5851578	6887098.5	6088987	6430980.5	7265484.5	6800934	6596428	7100033
5283305.5	5019047.5	7511319	6267922.5	5535016.5	7422619.5	5640978	5596999	5470459.5	5988521.5	5957037	6656623
6239504	4353974.5	6040461.5	7453785	3186666	4733373.5	5154459	5640872.5	3426241.25	3796591	4465884.5	5119911.5
5907077.5	6901110.5	6061715.5	5235239.5	3396986	5126036	5913841	7208994.5	5529279	6216284	4308641	4904765
5193187.5	5469512	10176029	3718594.75	5111343.5	4369628.5	5575726	8195663.5	3970523.5	4956829	4322211	5753959
5140213	5350252.5	5845919.5	5126871.5	2560067	6119252.5	5419506.5	4557795.5	4728283	5040139.5	4615505	6525534
4819478	4829214	6273072.5	4899008	4399633	5695896.5	4636567.5	3491683.75	4833760	4280014	4722296.5	5259332.5
5726548	4294949	6263722.5	5739597.5	3627733	4578312	5018162	6367891	5557871	4167624.5	5249940.5	6880591.5
6941706.5	4099509.5	6346516	5926394	3745456.25	5105853	4369692	3727255.25	4724216.5	6339456	3142596.75	4855781.5
3034070.25	2163487.5	3917521.25	2315439.75	5307882.5	2010259.75	1727455.125	2580574.5	1216693.625	2290631.75	2722076	3056565.25
3981189.25	5198184	5281578	5450316	4873819.5	4386521	3862875	4124632	5977083.5	6044374	4958428.5	2866416
4343199.5	4609784	5089062.5	3151918.5	3504985.5	3170454	3040756.5	5326375.5	1995941.875	3355745.25	3075545.5	4492130
4001113	3596068.25	5383715	3790635.5	3815546	4611923.5	4076347.25	3792207	3897419.75	3471891.75	3865093.5	4678751
8499.640625	1989.708984	2944.720703	18364614	Filtered	Filtered	3054.815918	1449.42334	Filtered	13169.09961	9694.986328	3034.1521
1963291.625	4448079	3638.05957	2479349.75	1315145	2710166.25	1250563.375	4928451.5	3666774.5	2312955.5	5624.338867	2903616.25
5455014.5	4173835.25	5904105.5	2650163	3314003	2695522.75	4080256.5	5554346.5	3600086.25	4196078	3363703.25	3726707.25
2604192.25	3481496	2945033.5	3544605.5	2848030	4036197	3628407	3213951	4043942	3881572.5	3283344.75	3164866
3795139.5	4531884.5	4738626	4648785	4062785.75	4735415	3411147.25	4271237.5	3800470.75	5006670	4549274	1013890.25
2964797.75	2355850.25	3809191	3616359	3075202.5	3088589.75	2775200.75	3070236	3264041	3045469.75	3113767.75	3591295.25
4373662	2742257	3373369.75	4178094	2509874.25	2730360.5	3074669.25	2527266.25	2623624.5	2984895.25	2849059	3613551
3682776	2902544.75	3159318.5	4277939	2204436.25	2745090	3181770	2461212.75	2971887	2724469.25	2735631	3562642.75
1623008.25	1901903	1555197.875	5203150	3219010.5	6654000.5	1586596.375	4054729.75	1935105.5	3162570	4203809.5	2509823.25
4269083	2604256	4335890	4271663.5	2273538.75	2752746.25	2808241.25	3865024.5	3817001.25	3284338	3185472	4809512.5
2229035.5	2331073.25	2272646	1718739.75	2591087.5	3152737	2922762.75	2683942.75	3287434.5	2358826.5	2619486.25	3338804
2911657.25	2355095.75	3294886.5	2959053	2166951.5	2559277	2427466	2552727	2241587	2322748.5	2728900	2759522.25
2641999.25	2741358.5	2140880.75	2300395.75	2540930.5	2962768.25	2388581.5	2902060.5	2821802.75	2288757.75	2783444.5	3135758.5
3006268.5	2639677	2939283.5	3446736.5	2253820.5	2406474	3255127	2730466.5	2335363.75	2197847.25	2812855	2461542.5
1579705.625	2010103.625	1321976.625	989537	5624302	1381705.5	1321095.625	3277687.5	1456376.5	1749072.5	1278121.75	2188979.75
2526646.75	2158626.5	3210164.5	2709737	2271472.5	2095349.625	2625767.75	2321553.5	2031823.625	2703422.5	2758301	2424510.25
2461714.5	2338395.5	2629843.25	2550349.25	1513059.75	2697047.5	2412903	2071488.625	1924522.375	2267312.5	1856386.625	2547731
1901850.625	1885197.875	2284914	1861906	1914882	2090743.5	2185611	2210447.25	2179411.75	2184509.5	1964494.125	2391966.75
1948295.125	2011720.875	2284619	2348872	1877558.75	2405987.5	2066660.125	1740435.625	2130261.5	2181370.75	1770733.125	2335711.5
2842592	2316809.5	2529939.75	2653079.5	1582624.5	2050693.625	1917510.5	1868505.625	2213328.25	2429577	2231607.5	2365281.25
1555248.875	1370892.875	1771364.75	1899882.875	1655662.5	2157542.25	943311.5625	2381486	2147143	2533333	2234943.75	2093111.875
1760478.5	2080866.75	2599116	1580056.125	1789094.125	1773836.875	2197163.5	2464890.5	1997970.25	1400253.25	1890604.375	1959140.125
1496423.125	2060101.125	1156499.875	1611247	3994665.25	1625922.625	1402379.625	2774434.25	1075945.875	2059763.25	2014029.625	1035740.75
1852464.625	1851698.75	2540182.25	2038928.5	1949178.125	1850513.375	2087463	1855644.125	1607869.125	1884726.375	2168881.5	2355188.25
1520984.75	1525687	1488987.5	383160.2188	5488730	966460.5625	1018227.5	2136520.25	744760.25	1189577.75	1016453.5	2850785.5
Filtered	1915131.25	Filtered	1756846.625	1778776	Filtered	Filtered	10063.84082	Filtered	Filtered	Filtered	1532979.25
1655558	2393672.5	1654027.25	1104900.875	1454856.5	966802.9375	2458980	2507905.75	2064294.25	3050211.5	1768553	1215808.25
1632526.75	1555210.375	1596665.125	1720820.5	1462822.75	1813050.75	Filtered	1573970.25	2094692	2190291.25	2149957	1999375.375
1921492.125	2151600.75	2587447.75	2388536.25	1707958.375	1845918	2237160.5	1453957.75	2145424	1708405.625	2201216.75	1791203
2330159.5	2080436.125	2251788.5	1206223.5	613285.6875	983265.5625	852797.1875	803403.625	933874.5	767334.3125	744217.75	896042.1875
1462450	1688629.25	2264851	1591342.25	978836.4375	2447909.5	1301800.5	1340593.125	1684933	1711467.5	1634375.875	1975043
2157616	1690353	2066797.25	1844147.375	1596713.125	1694740.125	2070550.5	1661270.75	1686028.625	1470852.25	1484679.125	2515807.5
1650790.875	2550465	3202531.25	2907087.25	2600059.25	2094928.375	2125766.5	2186069.5	1995229.5	1820241.125	1561711.375	744555
1809702.25	1807718.375	2070442.125	1913654.25	1699777.5	2265367.5	1712577.875	1615049.25	1423410.875	1686899.875	1376938.5	1830262.75

1953484.875	1753267.875	3760021.25	867208.9375	1847210.125	833538.9375	2104885.5	2200282	1243832.25	1994599.5	1320714.375	1325954.625
482709.8438	1340933.875	528530.0625	249858.4375	115383.9141	542425.75	522145.3125	2040029.625	536979.75	1009833.688	662709.1875	1569662.125
1220402.75	1669006.75	1929028.375	1637566.75	1437200.5	1806912.125	1459382.75	1701771.625	1562065.75	1713966.5	1419140.5	1608428.875
1976663.25	982393.375	2329157.5	1113131.75	1813031.875	2045340	2107077	1901946.625	1732307.5	1034580.938	1790431.375	1251942.5
1537620.125	1171566.25	1790318.75	1464861.5	1304271.5	1310124.875	1646082.5	1558847.25	1379074.875	1751124.625	1630008.125	1766355.625
1833680.25	1376447.875	2003897.5	1291207.25	Filtered	858146.375	969523.8125	1725862.875	Filtered	1346953.625	Filtered	1920121.75
1207079	1362348.625	1758239.25	1399170.25	1353566.625	1233172.25	1506726.25	1464872.75	1217071.875	1446799.75	1639166.75	1756959.875
1566331.875	1773792.25	923231.125	1351023.75	1023343.938	1393348.25	1364229.375	1904573.75	1368625.125	1737170.5	1334991	1385912.375
68253.22656	51780.52734	1726413.125	1674198.875	827008.0625	84150.74219	1550528.125	133619.4063	1261956.5	1588050.875	319380.5313	2099733.25
1554819.875	1294390.25	1682662.25	1596982.25	1096559.25	1806256	1357044.875	1763334.375	1131972.625	1444530.625	1187394.5	1580581
1191053.5	944645.625	1760012.375	1606189	1318290.875	765194	1233788	548475.1875	1097743.75	747534.3125	1317448.75	2207978.5
836986.5	2307890	477443.5625	616035.75	5561179.5	1485818	420743.0625	842446.4375	539446.875	793853.6875	457742.6563	624316.75
1524179	1294109	1785651	813160.6875	1130146.125	1121790.25	1338332.875	1331626.625	1331583.375	1335329.875	1290060.75	1435410.5
1524201.625	1462502	1659736.875	1754554.75	1208105	1119810	1646730	1634941.5	981425.1875	1644094.125	1714390.75	1447341.5
1392343.125	1540579.875	1544011.125	1138868.75	1308128.125	1210264.625	1215511	1334949.25	846078.75	1193195.5	1333167.25	1337462.25
967130.3125	976426.75	1019965.313	1162002.5	1170680.25	1511004.625	1005818.625	1136072	1007784.688	866031.9375	1207745.625	1110093.75
1119011.25	1213923.75	1564452.875	1313188.125	1024226.5	1313013.125	1063884.375	1467229.125	1379072.625	1305577.125	1017398.375	1132468.125
849353.5625	1212442.375	1397707.75	1104791.25	791062	1354183	1187646.75	1000119.938	1183399	1130737.75	1105529.75	1012392.938
685180.5	539827.0625	338775.375	4554092.5	734808.375	303595.625	3492492.75	884715.5	564957.125	853141.9375	616361	409768.5938
1205901.625	1041508.188	1524907.125	1324994.125	906253.9375	1211116.625	1165436.75	825067.9375	848486.0625	1198231.75	1093581.375	1257664.75
2377629.75	2219852.25	139318.2188	1589437.375	66283.96094	1625908.125	1511215.625	2223100.25	1167557.875	1337977.875	1381637.75	2148025.5
991288.75	883559.1875	1228655.125	1106176.375	958120.375	1150766.5	1031377.688	1089657.375	1177321.75	1051210.25	915064.8125	1550540.625
117196.6953	215062.7813	197028.2813	118716.4688	49498340	131412.2969	285011.3438	207621.4688	147502.0625	83608.60938	141989.4375	137724.5938
735903	1090513	791422.1875	1361137.5	767432.1875	1568181.75	973316.5625	827813.5625	1308135	1255764.875	1228153.75	724219.3125
947981.375	897713.9375	981637.8125	765942	1020544.938	971508.1875	1025889.938	1110384.875	1008891.313	979261	1149631.5	1582254.375
846261.5	881093.4375	919322.625	744803.0625	984173.5625	894011.5625	888208.6875	1060424.75	927563.875	766178.3125	913265.4375	1107774
1009570.938	741024.1875	1403888.25	420597.7188	1126197.25	555756.6875	523259.1563	906368.0625	457251.2813	537796	359763.4375	739317.125
784693.6875	508454.2188	648653.25	500098.2188	749367.9375	1700946.5	593121.0625	624843.75	575873.625	662301.3125	506285.25	2541654
830786.625	937207.0625	944575.8125	1173266.125	971610.9375	1151180.75	924140.375	768007.25	984533.5	973818.0625	902797	1065980.625
384205.125	1868218.625	297392.5938	346203.4063	3816048	786306.8125	201138.7344	723389.1875	349454.9688	639864.375	330328.3125	338459.6875
862484.625	1260903.125	824026.0625	1324745.75	1150269.75	1123668.5	866259.1875	887745.875	1498407.625	1393929.875	1387261.5	692128.5
986380.375	778370.0625	940205.0625	968649.0625	901147.3125	817349.875	945132.875	718768.1875	1058060.625	999213.1875	897328.5	732184.8125
585178.125	758269.9375	Filtered	865429.9375	361165.4063	851272.6875	946067.9375	Filtered	856519.125	613772.375	742057.6875	Filtered
408968.0313	322770.7188	271835.7188	332402.75	21877682	568468.0625	453284.5938	396667.7188	462990.375	299857.4063	413343.0625	247674.7656
613133.0625	Filtered	1025405.563	859660.25	278478.9063	165375.3125	755509.8125	90828.17969	1009011.063	903105.875	667104	1073626.25
783038.75	675956	895261.25	873743.9375	635418.75	715914.875	779458.875	567484.9375	503983.4375	857105.6875	719370.25	779362.1875
703244.5625	660468.4375	693547.875	790485.0625	649070.9375	792258.5	614665.5	607018.9375	643786.0625	595770.3125	631511.8125	919047.875
75827.10156	111442.9063	97522.41406	61811.85938	30987576	65705.14063	151942.6875	113511.8594	82396.74219	45027.57422	73421.27344	84105.39844
573619.1875	635306.0625	542855.3125	556838	662760.5625	707930.25	626289.5	769531.6875	646258.75	593623.125	644018.0625	866568.5
651500.625	583734.25	791455.375	760330.8125	533992.125	667708.6875	Filtered	694712.25	589583.3125	778320.125	583560	619265.0625
521800.7813	813435.6875	568494.3125	793835.875	900906.625	940709.25	500090.2813	395413.6563	635449.375	832214.0625	688724	647953.9375
576677.875	565384.5625	630966.3125	709478.5625	537333.3125	588414.8125	560057	579735	475392.125	623620	468356.6875	685076.5625
461174.625	549886.625	676943.6875	687350.6875	449755.9688	557376.625	597578.375	685299.375	674703.625	662090.125	486522.6875	791543.9375
270208.8438	380104.75	173465.0938	149759.7188	2844238.25	259847.3281	239377.7344	652141.75	232137.75	365455.5	193850.0625	284338.5313
634553.5	215649.2344	638404.25	75974.75781	1233937.875	159191.9688	644207.5625	331973.3125	134781.7031	630590.3125	641774	399252.6875
510745.9688	547577.125	664435.3125	637979.625	444665.75	621822.375	475542.75	381143.75	612304.625	547935.0625	536328.5	671077.375
311696.125	229081.0625	209256.25	321493.3125	3483408	205413.25	299786.4688	638843.3125	133543.5313	209192.9063	166225.2031	294044.125
308367.2813	501289.8438	331815.4063	446750.9688	2281298	121504.6094	158379.2031	407071.7813	243629.0156	131421.4844	277983.0313	519369.1875
616496.8125	486611.5	807043.0625	615764.5625	534624.5	432504.6875	544147	681662.3125	685908.625	1020896.75	625137.5	711488.5625
129561.8594	327261.5	150660.6406	134501.4063	1897093.625	115300.1797	311673.1563	677650.8125	183797	381457.75	209755.375	270684.7813
552709.0625	547739.6875	730733.4375	554928.125	540013.9375	530182.8125	495904.125	573870.125	698638.625	605878.375	472028.2188	589740.6875
407877.6875	416946.625	924687.3125	876917.375	526109.875	549612.875	424339.8125	465222.2188	561852.5	418413.3125	379950.8125	400976.4688
276945.6875	713766.875	438373.4688	696633.25	567597.0625	423367.8438	318835.25	622164.1875	429469.625	966462.6875	654817.4375	556473.125
455926.0313	515260	467288.9688	493125.9688	483517.25	429395.2188	609303.875	683330.6875	491778.7188	459300.8125	397916.9375	574902.5625
563614.8125	48959.21875	567944.3125	61777.08203	719553.1875	59983.42969	45048.58594	58893.82813	57097.02734	54749.55469	54549.21094	639328.5625
135828.0156	537651.8125	806308.75	362421.4375	478660	508972	491387.75	686455.1875	556712.4375	424435.8125	423855.9375	327339.3438
312850.875	395058.8438	360588.0313	442435.6875	312514.8438	324930.4688	333315.4375	269249.0313	392177.25	372625.5625	321937.7813	333474.2188
488653.875	488693.2188	591484.8125	595171.125	653148.125	662904.125	513444.5938	410618.8125	455713.4375	435125.1563	413341.8125	531105.375
388902.125	373018.0625	464401	485297.1875	416666.0625	521011.9375	406599.875	401254.625	383268.4375	412977.75	434844.75	450353.2188
447973.1875	403513.3125	437325.25	294856.125	332155.875	1943.295288	548373.375	389875.4375	459374.5938	386231.375	424949.2813	460982.0625
274505.0313	352488.8125	793838.8125	793236.375	297737.1875	664759.1875	633204.5	334751.0313	679110.5625	374611.1875	296814.9375	397009.9375
482072.0625	529390.6875	620064.5625	515634.125	332467.8438	428906.6563	559876.125	430328.125	620330.625	418639.8125	493416.9063	556407.125
300484.5	262660.5625	162879.125	348244.625	264090.5938	357476.375	304760.0313	289503.5313	329374.625	163575.8438	321887.3438	265491.2813
455923.4375	382961.875	488142.125	535281.3125	352811.1875	412551.9375	440654.4688	459801.8125	444273.375	497498.7188	440448.1875	542805.8125
Filtered	487991	Filtered	Filtered	Filtered	Filtered	Filtered	Filtered	Filtered	Filtered	Filtered	Filtered
362877.2813	366206.6875	401378.7813	444181.0938	259563.9375	363508.2188	361693.1875	405204.9688	450320.4063	418699.625	320113.4688	409644.625
268821.8438	422442.4063	458315.4688	361884.0938	403703.875	432217.9688	394520	404897.9688	431256.1875	377023.75	227026.3438	433591.375
189147.5156	278733.4688	275007.1563	308356.2188	104939.9063	164562.1563	223265.1563	184190.8125	236192.3281	186603.375	253393.8281	149407.625
496823.5	450089.2813	477348.1563	649285.8125	1453306.5	642672.1875	447127	362297.1563	252804.5313	246920.7031	240169.625	214465.7656
327630.875	248698.0313	415333.0625	401383.75	307939.7188	355122.3125	337484.5625	301920.4375	329009.4688	343999.5938	328652.125	515419.75
218174.375	352536.0625	330894.1563	261250.8906	281679.8438	340011.125	265692.8438	468917.2188	362648.75	486454.9375	412435.375	264641.9375
509939.2188	395605.8125	488221.8438	404028.5313	307251.6875	283834.125	288118.9688	314712.2188	331645.4688	359124.1875	321765.8125	402845.9375
156555.6094	309701.7188	391374.3125	317163.3438	284674.75	242884.625	432395.1875	583338.625	324560.875	325541.2813	177796.875	428265.5938
Filtered	Filtered	Filtered	210228.2344	199145.4063	Filtered	Filtered	Filtered	Filtered	Filtered	Filtered	Filtered
373003.3125	291746.25	408931.5625	384774.875	304876.1875	437502.75	345438.75	379833.5938	278412.1875	343202.5313	282016.25	387489.125

141962.5781	88653.82813	20370.74805	188277.7813	162807.5313	662143.9375	435641.3438	56658.45313	513054.4688	144415.7188	314789	106707.3984
447396.875	305990.1875	Filtered	142315.9688	Filtered	190860.6875	229554.1563	Filtered	203726.2969	Filtered	Filtered	Filtered
332272.75	362079.9375	394762.125	385282.875	346123.1875	368748	344162.1563	403862.9063	503589.5938	403775.125	415847	324615.9063
306372.0313	348255	299540.7813	156395.875	670485.625	214578.4375	283276.4688	357790.5938	218585.8594	307654.5938	258484.1875	330350.7188
314553.0313	329578.3438	178589.3125	506577.5313	545912.75	188199.6406	113878.2422	97095.33594	189691.75	203715.5313	344588.8438	165819.5469
129940.9453	94428.1875	Filtered	Filtered	530974.8125	52161.51563	98778.69531	86864.42969	39310.32813	39537.17188	Filtered	105289.3516
162648.6719	392489.3125	167545.0625	162535.375	765663.4375	170862.75	204378.7031	160229.8594	162770.6563	171226.1875	118583.5781	196557.375
Filtered	3272.609131	Filtered	Filtered	Filtered	Filtered	Filtered	6357.669434	10670.39551	Filtered	Filtered	36316.88281
Filtered	Filtered	Filtered	Filtered	182761.4844	Filtered	Filtered	Filtered	Filtered	444530.3125	Filtered	Filtered
320328	268488.0938	268455.1875	261011.1563	270271.9063	269596.5313	231775.4219	300873.8125	222105.25	296329.25	286670.6875	356369.6875
320321	205369.2188	346843.6875	377094.5938	222380.4688	247113.3906	282340.3438	234610.3125	240284.3438	219257.1875	297584.5625	250311.9219
180763.8906	255346.3281	946238.6875	164056.1094	263899.75	183519.1094	241830.3594	551654.375	183802.2813	179538	252001.8281	165153.6719
185650.1875	274556.6563	205902.9375	266766.5938	145133.2344	172686.2969	235302.1875	331009.25	263764.125	297215.6875	232563.5938	379629.9063
209159.5625	Filtered	333474.7188	485220.9375	8065.946289	202381.7031	185479.4844	Filtered	Filtered	424680.6875	388920.625	Filtered
102575.3203	163231.7188	193422.5469	192541.7656	290628.5938	133042.5625	161112.3594	244459.6563	139071.3906	181630.7813	170633.0469	310763.4375
164616.5156	392723.9063	167513.4531	160819.75	775199.9375	170964.6406	204368.3438	306822.25	162704.9531	170420.2656	118007.7813	193991.375
229190.7813	219012.75	308893.2813	209870.1406	214007.3125	288854.5	260872.9375	258559.7656	227308.4063	228994.9844	225581.9844	317303.9375
157804.5313	148959.9688	87979.76563	256653.8906	2040764.25	119746.0156	99842.54688	81830.24219	184990.125	183192.5938	291370.9063	106389.0234
277215	298091.25	313464.125	349687.7188	231808.1563	317685.5	363042.5625	251674.0313	343619.5938	317847.9063	303747.8125	303441
204911.7031	242922.8281	227646.0313	234427.5625	226687.875	301305.4063	288257.1563	231042.5781	247092.2813	256710.0156	265590.125	257661.5625
213542	207274.6563	250315	325738.125	308194.9375	336645.4688	311237.25	374953.4063	270267.625	349889.0313	294300.6563	168955.0781
54010.03906	44920.67969	62349.15234	76000.82813	711477.625	31058.23047	154301.2031	316592.2188	102439.2656	94646.86719	167396.25	181309
222747.4531	215249.5313	141725.5781	290850.5625	152332.8125	256857.5625	297081.625	190272.9063	305978.4688	222503.7813	200381.1094	245993.9688
237696.2813	239043.75	241105.5	246213.2188	191491.4844	251062.6875	277515.4063	221637.3281	266841.6875	279320.8438	227554.4531	296134.8125
153620.1563	257258.2656	189991.7188	171516.6094	144752.6875	214969.5625	152434.9531	304773.1875	204420.0469	250946.1719	175609.0938	232246.6875
Filtered	Filtered	Filtered	Filtered	Filtered	Filtered	156256.25	315185.8125	Filtered	321450.0313	Filtered	293070.5
273839.5938	171664.625	163751.2813	200205.8438	136011.2188	212457.625	201146.9219	198522.9375	360942.75	273086.4063	342423	257843
118154.4922	166185.1875	338548.4375	155555.1563	194501.2344	371066.2813	301777.9375	291656.9063	128860.1406	417053.4688	95904.54688	317890.9063
216800.4219	220601.6094	253850.75	215822.9531	192639.7813	244839.1094	236248.0313	256545.2188	272423.1875	259985.7813	246008.1875	258541.4063
273752.0313	238891.4375	28901.9082	197206.7031	204642.8594	15406.44922	273628	292936.3438	227127.5	130507.9375	14192.52441	237718.1875
230837.8594	204608.5938	270224.4688	336138.375	674369.8125	390809.1875	229627.1406	181702.5625	160062.3281	198293.0313	153228.25	190299.5156
163926.4375	282358.125	133415.2344	92071.57031	1122873	104266.7656	104435.1484	274691.4375	82462.45313	103557.625	85569.33594	151040.7813
241893.1875	271512.3438	271807.5	255310.4219	199811.4844	146657.0313	229495.9844	237165.8281	251379.9531	224465.6406	257337.0781	153180.4219
218326.0313	154781.2969	264152.7813	317412.2813	113245.0625	164640.125	298290.6875	219505.25	250522.8594	249924.3281	234864.3438	253404.8906
138414.8906	292918.7188	98981.61719	108148.3594	468130.8438	125349.1406	105240.9531	288184.75	196784.25	119889.4297	95937.5	262666.6875
210563.875	200728.7813	237193.5	233257.6719	202684.5313	203700.0156	231968.9531	212865.7188	232111.2344	215636.3906	230472.9844	203489.5625
250561.5	219268.2813	224856.5313	372662.0938	164510.8438	179870.9063	242747.3125	169124.1563	253611.2188	199333.5156	216016.2656	156927.6094
Filtered	23077.11719	Filtered	Filtered	Filtered	10739.92383	Filtered	Filtered	Filtered	Filtered	Filtered	Filtered
104361	220619.0625	96821.9375	76168.51563	1068464.5	85922.6875	110590.5469	200440.5781	75557.64844	87601.875	58866.21094	111867.5313
209508.625	212941.4531	235754.3438	231159.375	159743.9063	196422.1094	188120.5781	199988.0781	172817.8281	188189.0938	179019.7188	216033.7188
216184.4688	193133.5938	121768.6016	169017.9063	244749.2969	319955.0313	322062.8125	236911.5	172733.4375	211630.8906	210350.125	139872.7969
Filtered	Filtered	Filtered	Filtered	Filtered	Filtered	Filtered	Filtered	Filtered	Filtered	Filtered	Filtered
163983.6563	175015.6719	201588.5625	235697.125	168638.8906	184350.0625	194765.4531	152353.0313	174444	171304.9219	190104.6875	205410.875
Filtered	Filtered	Filtered	Filtered	Filtered	Filtered	Filtered	Filtered	Filtered	191384.9688	Filtered	131474.9219
163198.4531	188715.7344	127810.1094	202120.0781	76734.75	321058.9063	92405.35156	96571.28906	207381.5156	226252.3125	180238.7344	211307.1094
171247.7188	Filtered	Filtered	88056.17969	131278.7344	140695.3125	184266.0781	171737.5156	149819.6875	87344.50781	150283.8594	97331.9375
92304.28125	70847.03906	157739.6563	80406.58594	80575.17188	176550.3438	132890.1406	202890.3906	166759.25	169230.1406	145980.8438	209301.9688
168743.9063	189619.1875	210646.9219	234050.3906	175757.7188	194110.4688	148507.6094	196950.1094	205764.9375	198949.75	181732.8125	140987.0313
120991.2344	75304.80469	99419.78125	195387.9063	146522.4531	155511.4375	164536.0156	233745.8906	167484.4531	143949.125	84808.78906	97824.78906
157888.8438	165492.25	88857.25	249497.7813	781278.8125	95126.51563	56986.42188	48642.54688	95345.5625	103175.25	170286.4375	81200.00781
139606.9844	175338	183677.6875	203871.3438	158346.4375	172487.9063	165189.5938	190169.5469	189596.9688	182610.4375	147232.375	226208.4063
148576.7813	226314.2656	89308.34375	95968.26563	694845.875	153293.1406	75044.76563	244358.75	60588.64063	84472.9375	74515.70313	120275.7891
154869.0313	116011.9609	69044.61719	242288.125	893857.9375	200123.7188	77618.19531	37454.44531	96166.51563	118705.75	155530.2813	80615.82031
Filtered	Filtered	Filtered	Filtered	7746.484863	Filtered	Filtered	Filtered	Filtered	Filtered	Filtered	Filtered
105129	268962.9063	244033.8438	136424.9844	155321.4844	125329.1953	103723.7031	108565.8516	245538.4688	287240.1563	129267.2344	122429.2266
159679	166959.1719	182180.2813	166669.8594	111695.5313	145189.2344	150052.0781	156612.5625	117400.5156	181031.8438	153342.375	178652.2031
Filtered	Filtered	Filtered	138982.6719	Filtered	63376.01953	Filtered	318752.9063	Filtered	89873.27344	Filtered	Filtered
19997.11328	196980.125	18865.35547	61068.67578	592972.875	18952.81641	72599.33594	43202.66797	53812.55859	62584.97266	60203.25391	16269.6377
24072.52539	19811.7832	28824.4668	25032.29492	35690.88672	18516.4668	30818.08008	51370.88281	64788.12891	40276.53906	49210.27344	63958.19531
93747.35938	176882.6875	61223.31641	63762.08203	592038.375	71228.5	65497.24219	146112.0938	53719.30469	62563.32813	62840.66016	85944.82813
1.19E+05	5.90E+04	5.75E+04	1.20E+05	1.26E+05	1.11E+05	9.02E+04	2.02E+05	5.99E+04	1.73E+05	5.94E+04	2.68E+05
161683.4219	123799.125	216346.8125	188299.1875	147883.1875	163247.125	173937.1719	131500.3906	118393.5156	156280.6563	115717.4375	185492.9688
248780.2188	118934.0313	146666.2031	92171.27344	250250.0313	133703.75	125440.7422	159192.8906	106423.5313	137933.0938	119323.875	209414.8438
83472.35938	357545.1563	417590.5625	43140.26563	225566.625	62205.72656	78010.64844	124758.5781	45814.52734	63580.66406	59070.25	110130.1094
173604.25	156422.4375	81531.22656	75314.64063	80231.53125	84081.84375	226202.0781	188024.2969	67298.55469	260187.4375	70428.83594	145774.8438
168432.5313	199542.3125	159287.0625	169166.9375	177886.9531	167467.3125	162797.0781	186158.7344	167044.6406	67861.14063	124287.6094	199768.1875
83014.625	163525.8438	172658.2188	209706.7031	255866.6875	245456.3594	72346.08594	102079.6406	101646.5469	128979.75	107618.7031	119349.9922
101342.4766	111598.9766	125529.9297	111573.4219	749799.875	189542.6563	68778.13281	56798.91797	49753.63281	53351.41797	44888.48438	109697.0156
25799.21094	637794.4375	22307.64453	512632.9375	389103.1563	495061.0625	25546.24805	31644.86133	532633.375	289379.375	564727.5625	21459.19922
Filtered	Filtered	Filtered	Filtered	43220.08203	70842.42188	Filtered	117635.0781	84295.6875	30802.01758	71730.67969	135423.6406
113590.4688	158229.4063	216389.375	169239.1563	141065.75	89144.73438	175836	126661.5313	143234.3281	108199.5859	153001.0156	159607.7656
111454.8047	112447.0781	93035.34375	23831.01172	344406.1875	58438.92188	68553.15625	135208.1719	43715.80078	79707.26563	68841.54688	163215.6719
192720.6406	132074.9844	142926.25	124167.875	121380.2188	131122.3281	121800.7031	214960.4063	96229.875	100048.7578	91534.45313	141092.0625
125670.6484	118522.2891	162575.5781	117996.7734	63952.85547	107271.4609	139262.25	108873.3125	145048.8125	183432.7969	158975.75	392441.625
Filtered	Filtered	5624.683105	Filtered	1553.357544	5140.963379	5420.530273	3478.766357	13465.44336	Filtered	Filtered	19492.37305

164044.8438	110685.9375	154894.5313	167496.1563	131284.3594	145665.2031	162163.2813	156235.3281	147110.4375	115645.0156	162990.6719	152676.7813
134538.4375	107432.4609	155843.6719	180276.125	107534.1563	135474.3281	126302.875	85930.47656	147966.5938	117829.4141	138607	117752.4453
94634.82031	45487.78125	63273.09766	62336.33594	570143.375	59716.08203	71213.07813	141996.0781	54726.41016	62480.54688	60356.05469	78437.46875
145356.8125	114275.5391	133113.25	152999.2031	120256.2031	111296.3203	163548.9063	138610.4375	175816.5781	120976.3047	111786.9609	134892.25
Filtered	109829.6484	122143.1484	Filtered	95640.75	118473.0781	101415.7266	111350.4219	90307	133845.2188	132610.1563	155734.3281
Filtered	Filtered	Filtered	Filtered	6844.341309	Filtered	3525.921387	Filtered	Filtered	Filtered	Filtered	Filtered
126788.6016	199225.8438	161527.5938	213989.5156	101628.5859	21361.32422	155310.1563	17866.94336	208861.7344	177025.7813	109411.9844	261339.2344
215795.7969	99869.17188	135129.9063	241346.5313	41755.28125	158086.9688	80519	81897.29688	362414.2813	141932.9063	117130.6563	135334.3594
118242.8906	149480.9375	105796.3359	145477.3906	98349.83594	101745.1406	126810.3125	139428.5938	147339.8125	123913.5313	129818.5313	137384.5156
Filtered	Filtered	Filtered	Filtered	160124.7656	Filtered	Filtered	Filtered	Filtered	Filtered	Filtered	Filtered
105427.4063	90207.34375	144564.9531	57983.96875	199066.7969	30113.97461	64105.89063	108001	54348.20313	60153.29297	60207.03516	100369.0781
Filtered	Filtered	Filtered	Filtered	Filtered	Filtered	Filtered	Filtered	Filtered	Filtered	Filtered	Filtered
Filtered	Filtered	Filtered	Filtered	Filtered	Filtered	Filtered	Filtered	3418.723877	Filtered	Filtered	Filtered
85223.375	96671.92188	129301.9141	58380.19141	315895.5938	53979.75391	206549.2188	65366.91016	83637.26563	36667.85156	67752.08594	65904.17188
Filtered	193306.3438	Filtered	Filtered	5012.977539	117538.6875	Filtered	Filtered	Filtered	Filtered	304613.2813	4717.471191
Filtered	Filtered	Filtered	Filtered	Filtered	Filtered	Filtered	Filtered	Filtered	Filtered	Filtered	Filtered
87807.60938	121169.6797	181861.5625	147438.7344	107080.4766	103668.7344	101251.5156	110635.0781	165999.4531	112067.3281	145879.875	99943.42188
31336.23828	55704.21875	10996.5293	12473.41699	227231.4844	8746.878906	40222.10156	39814.89844	10995.15918	48958.53125	24786.03711	99692.0625
49163.60156	62394.48047	33123.65625	Filtered	9838.179688	24841.72852	Filtered	16990.87305	57302.73828	45704.19922	17931.48438	7363.769043
95086.17188	91985.49219	127498.3047	115973.9219	97180.65625	97061.23438	94436.74219	96345.09375	114786.4141	90827.32031	117401.9063	100954.8672
108995.4844	77871.01563	182955.5781	116026.3984	130314.2813	74018.24219	125390.8906	98779.23438	133715.3906	96411.20313	146546.6719	122929.3594
41425.26953	71078.17188	33567.22656	25710.51367	255610.8125	35570.49219	45570.9375	134384.4219	50022.47656	69293.75781	49955.90625	83450.32031
32571.5918	40458.26172	25965.30664	33288.84766	26779.21094	37869.6875	31515.50195	31850.10547	45714.01953	24483.97461	27062.54688	26822.92188
69710.57031	68165.35938	74064.63281	77481.66406	48127.57813	122916.3047	75312.92188	103687.0156	70948.42969	73578.64844	58151.98438	104260.4688
111406.1719	133579.4219	141066.1563	58930.01953	77760.4375	116926.4063	160904.3906	99069.76563	69923.17188	92304.71094	119405.6563	91505.57031
Filtered	Filtered	34795.60938	7475.312012	Filtered	Filtered	36441.82031	95331.35938	92486.61719	151782.4531	128177.3672	144556.9688
112572.1875	107783.2969	161281.5938	102635.2734	85468.50781	90558.35938	114878.875	113333.8203	117223.2891	102394.4219	122038.8125	130815.625
22779.90625	38020.62109	157808.0625	49771.57031	82897.71875	72814.86719	41439.24219	39887.24219	50310.23828	141976.1563	40840.72656	51889.84766
100159.6406	87305.9375	41971.48438	100756.3906	158168.1563	36403.59375	31018.41992	35118.78125	112948.5781	36793.75	37897.03125	29328.84375
64015.26953	99941.21875	127721.7969	101349.0703	76310.28125	56746.67969	99568.98438	74697.75	84235.74219	72406.70313	90336.57031	84013.01563
Filtered	Filtered	6788.703613	Filtered	10942.82813	585366.625	16180.4082	Filtered	Filtered	Filtered	Filtered	Filtered
66492.07031	80407.375	66888.28125	67055.28906	45304.49609	51666.75781	59116.22266	48236.58984	62270.45703	60868.19922	62431.55859	63062.32422
Filtered	Filtered	Filtered	Filtered	Filtered	Filtered	Filtered	Filtered	Filtered	Filtered	Filtered	Filtered
64984.74219	90986.98438	48885.1875	96739.03906	482688.8438	48439.64844	24231.94727	19682.08398	20767.76563	30596.76758	35626.04688	34239.25
34296.5	30646.05078	32839.08984	34437.28906	3401.694336	40329.63281	36648.79688	38739.26563	35319.34375	35193.64063	39378.07813	Filtered
45616.83203	76310.82031	111728.4766	67153.95313	82059.58594	50429.09375	93931.69531	70169.09375	72888.39844	56547.05469	69962.60938	63845.58594
79882.28125	72976.40625	78627.8125	70972.89063	58550.10938	87850.03125	91529.45313	109963.0156	94376.08594	92740.84375	85979.71875	83766.69531
Filtered	Filtered	94218.75781	Filtered	Filtered	Filtered	Filtered	Filtered	Filtered	Filtered	Filtered	Filtered
52666.47656	47975.82813	47984.83594	44868.17188	693301.8125	60201.12891	28156.63086	54893.23047	26565.44531	33364	26434.94727	36069.99219
Filtered	Filtered	Filtered	Filtered	Filtered	Filtered	Filtered	Filtered	Filtered	Filtered	Filtered	Filtered
59609.90625	65401.14844	98277.61719	89199.61719	71935.71094	49760.86328	100377.2578	98769.02344	86921.42188	82151.70313	90482.78125	64249.57031
65526.78906	105072.1094	73122.23438	111517.25	94683.89063	68001.75781	89712.72656	62968.01563	56545.30469	78324.05469	177761.5938	69769.73438
31043.87891	48188.34766	24826.67188	18564.76953	340827.8438	24227.80078	23159.79297	47380.71484	21046.23633	38898.09766	22052.67969	27528.23047
73118.72656	Filtered	Filtered	80871.64844	Filtered	Filtered	39424.51172	Filtered	Filtered	49831.48828	Filtered	Filtered
9511.90625	72260.90625	43950.55469	100777.5625	59641.27734	71272.4375	72267.85938	97995.34375	60879.85547	74034.00781	43326.10547	30777.86328
Filtered	3788.775879	Filtered	Filtered	Filtered	Filtered	Filtered	Filtered	Filtered	Filtered	Filtered	Filtered
49386.31641	46979.03516	58170.05469	37517.85156	155266.2031	28551.53516	81487.08594	41231.875	54655.05078	44324.19922	30437.58789	30479.29297
64235.39063	79300.6875	91852.63281	95418.1875	65603.53125	78888.75	61596.93359	98881.53125	79463.5	59125.85547	99525.91406	57861.35547
65651.10938	66334.88281	105455.8906	61599.00391	31184.35938	109121.7656	6758.831055	5258.033203	60453.71484	84391.92969	51554.25	115694.0859
40716.04297	75753.34375	32274.60547	30441.90625	148781.5313	29332.61719	36959.63672	84331.50781	51578.98828	40734.19531	21870.01953	41181.28125
90407.07813	39950.76563	39911.49219	90236.02344	482136	41092.97656	16731.11719	21914.96094	42835	54783.32813	80828.73438	30638.50781
34976.40234	48438.84375	61570.72266	30144.10156	Filtered	17471.99805	29495.32227	7954.684082	39672.21875	19981.43359	37246.46875	28560.43359
71100.24219	61678.74219	77824.27344	92213.5	62345.04688	72819.57813	59335.67578	71950.23438	84589.05469	65816.79688	90679.50781	72457.39063
58075.80078	57904.05859	2542.482422	4817.592773	10381.56152	888.8859253	Filtered	2285.981934	Filtered	Filtered	Filtered	67993.15625
38070.60547	37369.85156	33216.96094	114927.6094	76892.16406	129826.7266	33013.77344	86082.60156	39345.46484	68742.24219	103270.25	50742.9375
45579.90625	51825.96875	92590.14844	91812.83594	59916.46094	73233.36719	104275.5234	56788.78906	101454.9063	124624.6328	83032.07813	70125.21094
Filtered	Filtered	Filtered	Filtered	Filtered	Filtered	Filtered	Filtered	Filtered	Filtered	Filtered	Filtered
71755.53906	79855.27344	60706.67969	28344.32813	20280.57031	9332.306641	42139.0625	7344.365234	34069.53906	37182.83594	21694.40625	25690.35352
Filtered	Filtered	Filtered	Filtered	Filtered	Filtered	Filtered	Filtered	Filtered	Filtered	Filtered	Filtered
40234.92969	63433.89844	36155.37109	43162.69141	563290.4375	42936.96094	27234.64258	Filtered	21215.56445	37505.82031	24467.24414	55763.24219
54255.83984	45667.02734	51801.88672	49858.76563	79448.07813	44232.51563	52430.59766	55709.54297	49453.26953	73594.82031	38727.43359	90602.60938
37119.54688	89344.82813	97224.14063	49860.72266	58940.01172	83043.26563	24883.94922	33381.67578	48007.54297	89856.96094	40079.9375	65559.09375
29010.32031	45308.92188	32835.31641	29248.1875	252402.6094	32967.64453	17369.4375	47818.63672	24202.16992	26161.47656	24318.86133	28674.00586
Filtered	Filtered	Filtered	Filtered	Filtered	Filtered	Filtered	Filtered	Filtered	Filtered	Filtered	Filtered
49862.94141	55894.32813	64395.05859	73677.6875	43921.05859	Filtered	46849.3125	Filtered	41870.70313	Filtered	48363.13672	51390.72266
54846.30469	52737.1875	50020.37891	59389.27734	43885.87891	45815.76563	47522.09375	74522.78125	73136.92188	58367.40625	75050.96875	60601.46484
55674.75391	61653.48828	67875.91406	48950.51953	76736.64844	49358.02734	55236.41406	77406.6875	52233.23438	41757.47266	49601.73828	77857.5
34787.02734	35550.79297	172470.8281	32091.24609	37677.07422	49088.40234	37305.20313	58903.25391	45843.26563	47283.30859	46151.83594	76778.625
96045.57813	42778.10156	85798.11719	47803.26953	32301.78906	50054.75	32095.58203	44952.14844	36655.20313	39383.63672	37853.87891	38930.96484
Filtered	30391.73047	Filtered	Filtered	194702.7656	Filtered	Filtered	Filtered	Filtered	Filtered	Filtered	Filtered
77545.17188	50154.65625	51198.64453	68052.07031	33576.26172	29991.86719	49666.64453	66299.02344	63589.18359	57239.72266	66246.85938	60716.46875
53221.58594	68704.38281	79105.17969	63971.92969	57605.92188	46425.82422	58442.31641	56247.75781	60027.60547	67841.46875	62701.54688	50723.10938
61578.64453	73356.60156	82929.72656	59443.13281	53599.65625	38432.46875	62807.80078	59074.70703	63838.21875	48758.28516	63863.62891	52514.48438
54145.65234	46641.74219	86453.36719	56090.07031	42213.12109	65971.28125	61332.35156	61503.96484	41501.16016	53299.95313	42944.69922	58161.625
33487.82813	52197.42969	32191.01758	27939.56836	319857.2188	34647.9375	21899.49219	61597.33594	25413.87891	17613.9043	19010.77734	26415.15234

34124.99219	50310.21875	76405.42188	37832.41016	54809.50391	49807.41797	45109.5625	63763.40625	68432.24219	49745.11328	48613.48828	Filtered
25645.14063	24945.69531	20337.80859	18392.43945	183107.625	25571.14258	25540.56641	67021.99219	16183.09375	32174.56445	24980.74414	24787.43164
41941.37109	55409.73438	29618.61328	53646.95313	479472.7813	25920.81055	17302.05469	16546.54297	18740.14844	20283.66992	28098.70508	16969.80469
56297.20313	Filtered	Filtered	Filtered	Filtered	Filtered	30744.85156	Filtered	Filtered	Filtered	Filtered	Filtered
51427.08203	55676.74219	67377.19531	73126.74219	57270.27344	70675.92969	52597.21875	40292.41797	32799.40234	61435.42188	42313.02344	72814.36719
75356.82031	39353.35938	49718.62891	69966.92969	91489.07813	39172.86719	40643.89453	45554.89063	50777.67969	96509.21875	80311.03906	44313.62891
48083.14063	55598.03516	37765.5625	57911.10938	46369.25781	52153.09375	49439.67188	56159.21094	56446.41016	69505.74219	38276.71484	59342.91406
27476.87695	20952.98633	33648.75781	Filtered	9464.724609	31224.72656	32405.84375	74613.55469	54398.86719	54615.08984	43145.05859	39019.41016
Filtered	Filtered	Filtered	65690.72656	9637.087891	Filtered	Filtered	Filtered	Filtered	Filtered	Filtered	4717.387695
27815.32031	87982.07813	9121.458008	32950.875	86243.84375	15329.02051	14544.40039	46972.30859	31279.24219	13499.60059	27947.43359	56273.07031
18698.92578	32424.38672	18361.39648	16107.9502	251624.125	23666.84961	23082.32031	36820.85156	18442.58789	20367.7207	20847.91602	24641.11523
49017.97656	50990.69531	62068.69922	50443.70313	41729.10547	54268.02734	52716.69531	38010.61719	43984.86328	35634.19922	48025.91406	43105.97266
72279.75	55845.44141	33180.66406	30625.24023	52759.78906	59773.71875	49335.65625	3023.855957	45924.38672	76745.95313	94751.03125	35824.10156
2496.782227	Filtered	Filtered	4323.91748	353108.7813	2998.395752	Filtered	12394.91797	Filtered	Filtered	Filtered	Filtered
49109.58594	47259.26172	52925.60547	39330.74219	39294.46484	53516.0625	74760.24219	65854.19531	56488.66406	48037.25781	39742.08203	45721.90234
65413.32813	80134.36719	61658.91406	104328.4766	53843.1875	44518.58984	80070.03906	61164.78125	65677.60938	54912.99219	62040.10156	77092.5625
57441.78906	49820.89063	77354.21875	51766.11719	52129.57813	62817.5625	37590.00781	50278.01953	49317.40625	43267.85938	45955.39844	46024.58203
16802.52539	16205.39941	21142.46875	32152.38867	22574.13086	20821.54492	15103.50293	24957.87695	19065.72656	24001.25195	24578.34375	16418.30664
56408.69922	43493.73047	44628.31641	63252.38281	42173.70703	51023.04688	62297.42188	61825.24219	59132.15234	49757.59766	53386.94531	60121.6875
40215.44141	49622.92969	67003.0625	62626.74609	39168.94531	40395.78125	55690.30469	46371.65625	46568.25391	40853.62891	99651.21094	61975.04688
82269.17969	25168.71484	60780.45703	38135.78516	64537.375	45120.40234	35286.36719	171139.2188	35579.23438	72125.25	8803.205078	17804.95313
Filtered	Filtered	Filtered	Filtered	6766.321289	Filtered	16764.35938	Filtered	86293.29688	Filtered	Filtered	Filtered
26891.25391	62430.8125	13574.1416	1590.111084	127412.3359	1586.831421	20884.32813	37364.21094	24460.35547	26922.87695	16059.46582	22445.86328
43607.95313	48041.38281	53740.76563	54861.79297	43122.73828	48766.33984	45409.05078	58117.17969	47553.13281	55892.125	38706.97266	51352.35156
1506.991211	3574.169922	9918.460938	5259.258301	5685.013184	346665.7813	5489.146484	5703.505371	3632.312012	4617.575195	6495.430176	5105.45752
Filtered	Filtered	Filtered	Filtered	Filtered	Filtered	Filtered	Filtered	Filtered	Filtered	Filtered	Filtered
Filtered	Filtered	Filtered	5269.179688	Filtered	4158.172852	Filtered	Filtered	4056.667969	Filtered	Filtered	Filtered
40227.83203	62703.80078	35811.05078	24352	108086.2188	43036.69922	27472.6543	72057.94531	Filtered	37888.23828	23531.29102	6106.049316
Filtered	Filtered	Filtered	Filtered	31162.59375	Filtered	Filtered	Filtered	Filtered	Filtered	38060.45703	55280.19141
24379.38477	4553.479492	Filtered	Filtered	Filtered	Filtered	Filtered	38308.11719	Filtered	7344.564941	Filtered	28507.28906
Filtered	Filtered	Filtered	Filtered	9457.212891	Filtered	Filtered	Filtered	80481.82031	Filtered	Filtered	Filtered
13200.41016	20147.25	19456.6582	18600.25	13685.36328	10387.50391	23149.19727	16251.69043	14156.94629	29233.62305	12465.74512	22834.11328
18438.50781	38755.66797	33948.16406	10330.85449	71318.07813	15089.2959	25893.8418	69066.65625	6401.920898	26959.02148	19280.91406	47762.58203
46859.29297	51991.96094	52112.57813	50166.16016	37816.58594	36271.95313	43762.50391	49831.53125	46457.33984	52920.85156	46155.03125	40373.19141
42265.54297	34466.18359	46877.71484	56809.75781	64828.41016	40569.53906	37542.875	47292.02734	46641.61719	45930.30078	49512.20703	66927.28125
Filtered	1892.717896	2723.718506	1332.442383	38002.55078	1757.556274	Filtered	32996.76953	30928.45313	2007.390503	2504.272705	Filtered
30255.02344	32734.43555	54434.42578	37382.82422	34104.30078	27953.23047	40921.9375	35383.41016	34980.27734	29661.52344	38981.96875	35584.85156
56132.66016	26623.2168	58207.17969	30480.49219	17810.74609	29913.49414	36168.64063	34972.49219	44833.66406	34737.14063	39530.32031	31588.54297
Filtered	19144.22266	10366.67383	17590.2207	22516.80273	10838.01563	13323.15234	10818.88281	16260.37695	14953.72656	14902.72949	19492.19531
46629.36719	41453.93359	24768.77734	32495.99414	41712.84766	39046.21875	28963.16016	46628.51953	30988.74805	58009.30469	28145.38867	50133.59375
31272.68945	64059.31641	75782.08594	63465.625	39109.83984	34406.73828	57465.70313	37564.60156	48396.29688	31427.52344	44425.77344	57757.73438
41498.20703	40228.08594	44032.46094	53621.53516	33015.80469	39285.79688	45331.31641	42477.83984	55558.82813	41790.33984	45568.39844	38677.87109
33242.21094	41634.32813	42195.95313	35475.79688	37111.03125	35695.10156	46760.16406	27735.3418	41023.80078	36173.73047	35905.89063	35289.71484
33227.42188	28776.80859	52745.30859	42776.44141	41465.51172	45193.63281	36686.34766	52255.38281	51281.51563	27158.88477	69273.875	39048.29688
Filtered	Filtered	Filtered	4496.042969	204138.1719	2213.141846	Filtered	Filtered	Filtered	Filtered	Filtered	Filtered
Filtered	47506.5625	44365.94531	62990.11719	Filtered	43254.00391	33294.75391	46502.73047	46541.59766	Filtered	42264.31641	16049.58594
13665.26074	13846.86035	20528.97461	60022.16406	15793.64355	24911.44141	38323.61719	59532.25	18416.54297	50067.23438	46819.18359	80050.39063
Filtered	4618.269531	Filtered	4635.760742	558309.4375	4777.327148	Filtered	Filtered	2791.456299	2478.228027	7328.768066	Filtered
Filtered	Filtered	2902.754883	Filtered	305887.375	Filtered	2147.400635	Filtered	Filtered	Filtered	2568.569336	1970.971313
20744.35547	58619.28516	22739.25781	21954.29297	104489.1953	35596.67188	23238.9043	31401.82422	19947.75781	14745.90527	25167.06641	20049.54688
37614.96484	42056.55469	40835.27734	38565.71094	25205.69531	31622.41602	22956.20898	40736.15234	34180.42969	47260.01953	28242.91602	44123.74609
42533.73438	40560.33594	59646.73438	45683.51563	40897.68359	27174.89648	54950.125	41192.9375	36064.63281	34220.50391	30345.84375	41915.66406
42375.99219	32552.54688	26808.09375	36737.36328	24767.7168	20921.42578	36004.18359	28053.27344	32768.18359	33292.79688	24278.41797	30466.77539
43328.91406	36655.40625	37011.33984	35435.97266	28579.41211	25364.93945	33587.58984	33149.38672	43735.31641	31574.70508	28486.32031	26404.96875
10937.66113	8906.250977	70309.78906	29939.10156	31066.74609	73292.57813	Filtered	63531.42969	18650.22266	28134.88867	48635.04688	14815.78516
Filtered	Filtered	Filtered	Filtered	Filtered	Filtered	Filtered	Filtered	Filtered	Filtered	Filtered	Filtered
Filtered	Filtered	Filtered	Filtered	Filtered	Filtered	Filtered	Filtered	Filtered	Filtered	Filtered	Filtered
24426.81445	17745.18555	10318.01953	39914.22656	164136.9063	8305.734375	21961.375	25311.92188	24522.53125	16728.33594	19831.31836	11832.17773
Filtered	Filtered	Filtered	897.5794067	92665.67188	Filtered	Filtered	Filtered	Filtered	Filtered	Filtered	Filtered
37853.20703	23613.2793	50497.01172	22788.31641	17136.98047	22843.54883	33152.64844	38928.41797	98387.82813	57563.92969	78841.77344	24733.0918
28436.2168	41350.21094	23902.56445	44808.08594	171289.625	29526.57227	16064.41016	13820.68066	8363.555664	18095.39258	17366.96289	6777.578613
25986.32422	Filtered	63569.08594	25595.20117	25321.50391	26924.00586	22533.01367	30883.07813	8874.74707	27500.91602	31524.93359	28292.01172
25458.98242	32482.29688	46932.39063	16915.79688	100149.8594	18698.60742	75573.0625	21226.78516	32390.54102	13411.52832	24087.48047	19997.37695
Filtered	Filtered	Filtered	Filtered	59527.59766	Filtered	Filtered	Filtered	Filtered	Filtered	Filtered	Filtered
Filtered	4992.780762	4790.15332	Filtered	760048.25	Filtered	5545.256836	Filtered	Filtered	Filtered	Filtered	2709.836426
Filtered	Filtered	5666.141602	54740.74219	2304.164551	36169.22266	3451.729492	Filtered	39175.83984	18967.66602	1881.50354	2849.453613
17937.56641	25679.96875	32607.78906	24802.3457	28962.99219	32989.71094	16939.88281	34770.12109	24968.82813	30482.68164	23557.62305	41366.89453
34604.95313	39371.69141	48334.33203	44056.17969	32583.11719	49987.32813	34993.27734	32558.44141	30123.90625	24735.26563	24864.28516	24223.37305
Filtered	Filtered	Filtered	Filtered	Filtered	Filtered	Filtered	Filtered	Filtered	Filtered	Filtered	Filtered
42406.76953	37483.78125	41149.75781	52392.46094	46630.46875	65624.32031	48599.27344	57016.73828	53543.84375	9667.286133	8285.652344	48206.89844
Filtered	Filtered	Filtered	3991.640137	Filtered	13824.05664	5921.95459	Filtered	Filtered	Filtered	Filtered	Filtered
Filtered	Filtered	Filtered	10116.19336	Filtered	8907.521484	Filtered	2772.203369	Filtered	Filtered	Filtered	Filtered
Filtered	Filtered	Filtered	Filtered	47427.94141	Filtered	Filtered	Filtered	Filtered	Filtered	Filtered	Filtered
23340.37305	25548.37109	25080.78516	36138.375	23438.10156	34871.54688	29414.24023	29747.81445	27151.72461	40804.29688	29184.54297	27292.55859
Filtered	Filtered	Filtered	15588.91211	Filtered	Filtered	Filtered	Filtered	Filtered	Filtered	Filtered	1262.494263

24684.13477	27174.61719	51380.5	21170.32617	455340.0938	36134.16016	60056.5	8573.863281	5508.505859	Filtered	7844.134766	3123.06543
33494.08984	22097.20703	36956.9375	25313.61523	28632.86328	21861.91211	14438.42773	24597.21094	38287.40234	26082.375	25864.55469	32062.52344
34163.44531	33551.60938	31644.87891	34264.00781	33050.03906	32668.91016	39335.92969	35576.73047	48766.51172	32930.80469	35885.19531	31473.22461
19665.20703	28307.36719	48046.51563	15739.68848	83849.75	14866.54492	65700.41406	25841.60938	29935.17578	11384.86426	18189.40625	23741.11523
Filtered	12448.90137	Filtered	Filtered	74975.94531	Filtered	5161.713867	Filtered	Filtered	Filtered	Filtered	Filtered
30057.11523	26599.77344	29963.61719	29915.36719	28657.96484	29248.18945	33733.03125	34078.32031	26289.28516	26114.71875	37465.47656	36502.33594
Filtered	Filtered	Filtered	2454.916504	Filtered	6337.511719	4787.72998	Filtered	Filtered	Filtered	25919.04688	Filtered
36438.20313	41336.81641	41089.58984	50073.24219	108064.8203	40276.57813	32390.32227	26216.20117	24251.15625	22940.37891	14896.1377	20058.6582
4298.594727	4061.915283	10832.9375	4246.625977	19859.33789	3574.232178	16611.375	1426.763672	4694.937012	92985.01563	3293.137695	3060.190186
3567.263184	28308.50195	5354.789063	67131.53906	Filtered	79594.14063	4966.380371	Filtered	4246.465332	Filtered	Filtered	33645.98438
29743.2207	23705.63672	32030.99219	43553.03516	42222.78516	22906.79883	23301.55273	29617.23242	32183	29785.20313	30352.55078	39546.05859
26859.05078	28609.5957	36579.61328	38818.39453	36527.02344	33370.01953	28032.2832	21972.83594	25366.4375	25077.87695	29371.13086	23046.83594
19369.46484	27745.50391	31034.83984	26358.59375	21128.10156	28367.35547	24518.02539	19859.62109	26077.83008	39224.71094	38491.41797	21021.78125
26772.16602	31131.50391	35097.23828	27103.31055	33160.57813	24539.48438	31700.94141	26323.64648	29854.29688	25783.97461	31381.24023	31989.60352
29967.87891	30554.53711	24141.56445	38465.46094	22728.51563	28127.67578	32717.15039	30027.95898	35729.85547	32388.95703	41149.95703	26028.74805
20048.79492	31412.07227	30187.25781	40130.09766	19712.80078	24361.07617	39851.02344	35526.06641	33579.51172	30144.28516	26864.13086	13614.32715
29317.52344	28558.47461	Filtered	22578.12305	22997.9668	24236.87109	41796.89453	Filtered	35011.33594	28915.86719	Filtered	Filtered
Filtered	12560.30078	5400.374023	11696.9541	Filtered	Filtered	7552.897461	4132.47168	5814.491211	5740.157227	3937.327148	10126.67676
14140.01172	12231.78906	41929.42969	22779.58008	437480.9688	11785.5332	13086.03711	12546.35742	12112.4375	12131.625	19947.27734	3982.443115
Filtered	Filtered	Filtered	Filtered	31288.34766	Filtered	Filtered	Filtered	Filtered	Filtered	Filtered	Filtered
18189.37891	12522.21973	17471.45703	Filtered	13776.55957	17433.19727	16324.70898	15096.06445	15239.57715	Filtered	11613.66602	18017.79297
Filtered	Filtered	7691.242188	Filtered	101983.9688	68093.32813	59678.11328	109161.5781	55028.33594	56009.01953	89014.16406	5857.414551
40031.35938	31829.57227	37097.57422	31995.38477	19894.38867	34418.79688	31179.60352	32374.19922	36908.92969	30954.59375	34854.45703	35417.53125
29847.92773	28282.13672	38723.83984	31773.82617	23975.35156	26043.18945	26641.12695	25625.73047	31455.60156	23761.56055	25922.49609	22154.18945
29361.30273	25366.75	27398.32813	26596.40039	21962.4375	27850.72852	26362.92578	21829.52539	21729.17383	24702.92969	27056.35742	27900.74023
25343.39063	27316.51172	24447.11328	34688.42969	19298.14648	26414.35352	26018.86133	21112.85156	22684.91016	26664.85156	32622.33008	24543.97852
22180.51758	19353.3125	26399.00586	12111.5	20786.5	17606.55859	32664.12891	46246.58594	22165.16211	33554.41797	16002.70605	19755.91211
9518.417969	15453.12891	Filtered	13952.18262	92651.95313	10725.38281	Filtered	20707.60547	Filtered	8910.03125	4206.258301	Filtered
28829.13086	34348.08203	53244.57813	31878.31055	22445.16602	22912.65234	25499.39453	27473.71484	30318.0918	28846.46289	39517.85547	32674.62891
31085.06836	30131.10938	20620.60742	29680.67969	23058.04688	25169.0293	29180.98047	29165.52344	26290.50781	31190.54688	31401.97852	28085.82617
33334.77734	35356.77734	30938.53125	39514.50391	32203.2832	28833.59961	27091.2207	25405.54297	26081.17383	20745.08398	18180.29688	20287.48633
Filtered	Filtered	Filtered	Filtered	Filtered	Filtered	Filtered	Filtered	Filtered	28077.03906	Filtered	Filtered
26505.85742	36188.49609	21374.13477	25023.20898	26180.74805	36444.22266	25553.96289	34001.91797	30314.61328	30317.39063	24360.2207	25429.87109
Filtered	Filtered	Filtered	Filtered	2170.862305	Filtered	Filtered	Filtered	Filtered	Filtered	Filtered	Filtered
28803.5	20865.86133	29426.95508	35169.57813	19691.60938	25708.22266	27966.43359	28256.44531	31706.45703	31932.2168	30655.70703	20809.53711
23558.75391	23658.90625	26199.08398	27540.46484	22694.37891	21447.66406	26840.63281	30009.5	26344.67578	30165.41016	27238.47266	35002.60547
32027.04688	14353.85938	34185.77734	26938.10156	22151.63477	18005.81641	17361.22266	42388.46094	31558.87891	28443.10156	32843.60938	23691.26563
44465.91406	35844.46875	45894.47656	30427.16992	45720.0625	43009.07813	27795.50781	21294.44922	31048.50781	21950.30469	26643.94922	13962.14648
Filtered	9144.40918	Filtered	Filtered	Filtered	13525.4668	Filtered	Filtered	12354.07324	15556.54395	Filtered	Filtered
19128.11328	14295.4707	25046.31836	27847.11523	17258.77148	17095.59961	25177.11133	23572.72266	23653.29297	37199.03516	31434.93945	13289.63184
12436.80859	Filtered	13939.05273	10232	7640.003906	7088.17627	7789.41748	10356.72656	8044.364258	8145.556641	7031.458984	80345.5
18084.48633	18558.81445	24795.79883	33376.22656	11787.5498	32976.53516	19257.66211	17021.59766	21613.33203	20462.75586	34190.58203	19160.60352
27035.80664	22285.03711	27443.24023	29046.72461	21624.19922	21474.9375	23350.88867	25182.28125	31025.31641	29217.67383	23987.17578	25524.69922
17563.07227	38049.12891	14020.2002	19192.85547	57709.64063	12646.94727	14193.37988	16640.35938	8838.375	8435.354492	9694.341797	16777.09961
Filtered	12544.60742	18026.58203	12309.5332	12697.48633	6744.59082	9704.580078	Filtered	6785.233398	Filtered	10846.16504	13335.69141
8233.421875	20304.83789	27277.10352	63783.1875	24847.0957	9104.265625	36422.32422	10903.41895	21103.24609	19233.96875	14769.94043	33794.93359
28536.04297	29448.26563	26511.10742	34053.54688	29259.06836	28864.66016	30079.69531	22712.02734	32426.53516	27595.55078	29920.70703	20484.54297
25672.52148	23650.56055	24106.15039	23951.74414	21862.12109	21109.12695	25792.24609	26764.49023	33716.39844	25437.12891	21657.26953	18117.12109
14681.82715	23508.66797	23078.90625	24804.29688	20017.95703	12360.0498	44536.90625	19515.31836	19654.51953	20974.27734	13597.24219	17160.50781
20633.92578	18786.22461	40515.97266	16927.45117	17850.86523	29560.79297	20834.48047	18083.34766	18299.66211	16692.48047	20534.54297	27315.39063
25012.82031	26031.80078	26790.78125	31528.70508	24907.68359	22349.44531	28470.05664	29056.44531	32691.07031	28184.76758	39112.79297	23380.68945
Filtered	9067.410156	Filtered	10762.4707	Filtered	7780.438477	Filtered	Filtered	8444.556641	11912.36719	Filtered	11790.37402
24511.92773	22761.61719	25925.21094	21164.20313	18181.47852	21843.0293	21917.00586	29041.57617	23865.61328	22759.9668	23776.26563	23747.18164
Filtered	Filtered	Filtered	Filtered	Filtered	Filtered	Filtered	Filtered	Filtered	Filtered	Filtered	Filtered
8796.59082	10922.95898	13637.35938	14268.43164	8091.325195	6505.488281	10828.20313	9871.25293	8168.794434	8716.881836	14306.7959	10941.76074
17467.46875	28528.66797	24586.13867	18796.95508	26358.74609	14857.3291	23981.57813	14236.3916	22599.64453	15103.81445	22371.49219	13641.62793
Filtered	Filtered	Filtered	Filtered	Filtered	5904.812012	Filtered	Filtered	Filtered	Filtered	Filtered	Filtered
18509.85742	18370.125	17850.52344	21945.87109	29241.03125	24958.42383	13816.05762	19853.07813	19914.38281	30801.6543	19102.79883	19804.22656
22839.98633	20546.32617	31405.00586	19258.4707	19108.00391	17137.93359	30507.93555	33410.16406	25135.44922	13605.14551	24335.68945	2070.133301
30013.83203	18376.83984	28041.37695	30229.63867	17235.23633	29705.21289	34703.64453	19158.86719	23988.50781	17491.0332	19377.60547	26531.66797
Filtered	Filtered	5824.27002	Filtered	20665.32422	Filtered	95740.67969	Filtered	Filtered	Filtered	Filtered	Filtered
14035.27344	19733.50391	21094.16406	33777.875	17882.09375	24567.17188	15133.10547	25282.76758	27819.23633	20696.01172	33421.32422	22532.51172
Filtered	12505.8252	Filtered	Filtered	45048.21094	14162.55566	Filtered	10849.22363	Filtered	Filtered	Filtered	Filtered
27584.45508	19196.03906	24071.07422	32160.14648	14590.5625	24157.52148	24698.08203	30575.08984	28159.85156	18767.54297	22616.43359	24998.88867
17756.83398	18340.57227	24301.41797	27283.94922	23182.01172	25807.28711	26531.55664	27075.75586	27395.48633	19418.94141	24870.16211	18968.51367
Filtered	Filtered	Filtered	45854.48047	21184.16016	29810.07813	Filtered	Filtered	3117.658936	3290.653076	Filtered	Filtered
30495.77148	7746.032715	28569.51367	16558.29297	22633.95703	12930.04297	15608.69336	28774.93555	10501.86719	8579.746094	25235.17188	17511.32422
16195.54102	18677.06641	26908.07617	24355.39844	19960.43164	20710.13672	16084.75684	19518.8418	25703.7793	19713.07422	21279.35156	24191.58203
25515.99805	22009.09766	22029.51563	16713.74219	23060.82422	18654.09766	17734.49414	18591.69727	23233.48828	22931.02734	16424.34766	24074.27734
Filtered	10105.45605	Filtered	16388.29492	29207.32031	Filtered	Filtered	Filtered	10495.75977	Filtered	Filtered	Filtered
5064.64209	8378.347656	Filtered	4949.675293	80747.28125	6075.846191	Filtered	11482.375	Filtered	10244.72656	8772.432617	5652.399414
19116.16602	22725.27344	23022.24219	19001.20313	21308.60742	18421.19141	22040.98828	19497.58984	21216.08008	28038.73828	26357.95117	18564.25977
Filtered	9349.861328	4251.179688	Filtered	64802.35938	9312.985352	10919.38477	27992.33008	8127.586914	10445.05566	6167.031738	10089.22656
Filtered	18951.41406	Filtered	8919.666016	59481.40234	7257.272949	Filtered	54870.96484	11845.02832	Filtered	13278.80762	38414.91016
19177.14258	30484.86719	Filtered	Filtered	10522.875	37924.94531	Filtered	22793.41406	21514.7832	30765.38281	Filtered	Filtered

20424.60156	21701.1582	26601.21289	23166.70508	19056.01758	20790.28516	16081.76953	21736.58789	25473.92578	28386.95117	29892.57813	23083.33984
Filtered	29418.45703	Filtered	26808.66016	Filtered	29777.44141	21944.54297	25503.57617	16310.24805	15318.14746	21569.44922	16709.20508
18302.55273	16302.94141	19638.70703	16834.01758	21478.46289	13083.60742	21699.75195	20176.03125	16775.57813	16633.56836	20398.89844	15074.15723
21949.81836	25006.66797	28545.88281	21948.74609	35466.42969	19384.57813	28048.58008	18249.0918	19505.55273	15887.9668	19535.96484	13501.87695
19302.19727	22191.29297	18598.19141	24477.69336	22507.43359	20415.41797	23527.70117	21160.75586	21429.50586	20844.83984	21782.50781	18625.34375
17014.12305	35980.58203	14139.34863	17425.09766	12830.22559	25392.40039	15576.02832	19769.72852	16371.76074	11637.55566	11221.83691	40891.10938
16964.27734	21045.66211	9770.092773	20509.58008	13549.41211	56607.00391	11568.03418	9318.322266	17374.93359	13754.43652	9070.553711	11905.9873
9401.404297	8352.157227	5806.975098	37137.67969	19817.56836	14504.35449	53371.82031	11843.58008	10322.24023	27622.47266	8011.855957	10957.80664
Filtered	13296.16406	Filtered	22371.48828	8577.242188	7483.025391	Filtered	8495.504883	Filtered	Filtered	Filtered	Filtered
12079.81934	14686.63477	11142.78027	Filtered	14440.83301	16716.59961	16511.45313	23357.86523	29408.61719	16726.12891	19197.59375	16522.38867
19161.9375	18634.95313	22518.81641	21616.31055	20983.45117	19581.41016	19970.64844	21031.48242	15901.04688	18842.2793	24505.77344	20438.86719
Filtered	Filtered	3356.450684	Filtered	6496.328613	3332.656982	Filtered	Filtered	Filtered	Filtered	Filtered	Filtered
22167.65234	6644.007813	37504.71094	6618.242676	3682.556641	5032.630859	2164.532959	3910.407715	5069.206543	4377.654297	4401.691895	4658.439941
12923.69336	9348.331055	8939.246094	6292.293457	7081.441406	6125.609375	7694.217773	9026.12793	6384.955078	8132.722656	13041.46973	5744.273438
13118.13281	11678.24121	12703.92871	19589.81641	5645.424805	14256.96875	15677.65527	33419.47656	17733.03516	17543.67578	14540.67383	27610.41211
14581.62207	15452.29785	15781.42383	11880.15234	17604.07813	17509.68359	15421.42188	16937.89063	17021.28906	19515.01953	18964.7168	12631.96484
23230.2207	15645.375	26623.83008	21452.89063	14425.81836	10726.81934	17610.85938	21894.82813	17399.5	16136.42773	19067.85352	24984.23242
18463.56836	17150.65234	18056.69531	17493.05859	17371.16016	14010.25488	18288.35547	19343.12891	13922.22949	16159.97461	31441.0625	22782.32813
Filtered	5160.113281	9654.244141	14073.19043	14876.77734	Filtered	Filtered	Filtered	Filtered	4917.025879	Filtered	Filtered
18243.47852	14740.70996	23244.23242	29382.8418	12736.66895	22690.4082	16735.37695	16256.80371	28361.49023	23624.43945	13343.51563	14372.59766
19681.89844	17539.37109	18995.66211	18603.56641	14383.3125	14563.38086	24528.79102	17614.49414	19028.16016	16811.80859	15294.88867	21150.67578
20892.11523	18516.83203	15706.60254	22026.36328	18994.13672	17556.43164	22053.46484	18795.70703	25027.61523	21617.94727	23223.25391	19085.14453
23058.88867	18940.69531	16856.82227	20535.48438	18164.37305	19574.02148	20541.67188	20131.10156	20699.7793	20128.42578	23436.59375	18265.5625
Filtered	Filtered	Filtered	Filtered	33174.37109	Filtered	Filtered	7581.344238	5171.291504	Filtered	Filtered	Filtered
9807.600586	12716.21973	5859.609375	Filtered	49751.77344	7225.041016	8884.151367	16707.2168	4543.85498	5206.716797	Filtered	18758.16016
22091.86719	24929.71289	20963.66016	32911.89844	29310.33789	35259.85156	21987.58008	15598.04395	17032.60547	13752.53418	18168.53906	10321.87109
14766.85449	24242.11719	31505.70313	17825.88672	19817.31836	10298.32324	16387.625	10804.29883	18118.81836	15218.87598	16006.53809	19779.83398
Filtered	18698.90625	23936.86133	20675.43555	8500.509766	8419.174805	20042.69922	18450.54297	Filtered	Filtered	9735.950195	Filtered
14835.68359	25370.9707	18586.56055	28187.19336	31767.04102	26698.50391	26636.99023	42475.90625	19124.17383	33601.79688	26375.6875	26831.67969
9556.029297	20271.83203	20222.51367	Filtered	9563.630859	18200.82031	Filtered	21874	Filtered	20620.02734	20827.43359	15563.13574
Filtered	Filtered	Filtered	3113	Filtered	21297.59766	Filtered	Filtered	Filtered	Filtered	Filtered	Filtered
3724.351807	13385.53711	3320.267578	3100.142822	Filtered	6836.605469	5048.769531	21819.16406	5083.661621	10655.27148	6199.701172	22444.73242
21799.67773	15297.64063	17754.91016	20327.20898	12254.44531	14832.70215	16418.625	20353.52344	16841.83398	16513.11719	21626.51953	14830.97363
Filtered	9859.758789	3638.58374	27700.47852	50794.35156	38696.08984	25137.16797	5237.619141	4771.018555	25706.80859	7440.790039	26349.26367
10391.51465	9226.244141	10316.19922	9372.586914	6914.050781	8248.291016	14119.98828	13716.81836	11589.01855	21816.72266	29545.05469	10330.31836
18896.85156	14274.79199	15806.02246	17343.96875	13457.13281	13019.30566	17195.33008	18723.67188	18464.54492	12475.27246	17500.13086	17946.34961
3001.765137	3000.216309	Filtered	Filtered	16262.72949	Filtered	Filtered	Filtered	Filtered	Filtered	Filtered	Filtered
15072.0791	11574.75195	15244.55176	17290.16797	9275.611328	14603.25684	19332.16602	11766.81055	17184.25	15116.97559	21276.71875	12280.53125
12896.95117	15526.17383	18383.28516	8385.040039	106160.9531	9604.630859	15312.5918	15276.42773	14288.68848	17404.7168	Filtered	15601.94727
17173.65234	17076.33203	20017.81836	20184.74023	13583.54492	17808.18555	16576.66406	38529.92578	15781.71973	18300.79102	18839.8125	19182.70508
1769.61377	2504.499023	5130.390625	2345.685547	75161.49219	Filtered	Filtered	Filtered	3171.391113	Filtered	2968.39502	2792.588379
14935.61328	15892.15332	18362.49219	18981.70117	14517.36816	12541.85938	16258.94141	12569.19629	21980.86523	12652.27344	19309.0332	13137.31055
2928.84375	Filtered	Filtered	Filtered	Filtered	Filtered	Filtered	Filtered	Filtered	Filtered	Filtered	Filtered
28953.62109	17086.03125	11716.88281	12917.79492	10385.37695	9635.042969	25295.84375	3768.147217	12782.3877	25058.38477	22836.04297	13046.08594
3043.634766	5391.939453	11876.36621	11630.77539	9168.433594	4504.249512	5763.979492	5954.164063	6675.787109	10528.03418	9136.048828	9022.186523
11250.19629	9666.220703	11205.54395	12769.1123	11114.34863	11446.28711	13602.58691	8910.679688	19665.50977	18161.92578	12604.98535	8251.038086
Filtered	Filtered	8349.148438	Filtered	17747.37109	19417.2207	Filtered	Filtered	Filtered	Filtered	Filtered	Filtered
Filtered	Filtered	52903.09766	Filtered	Filtered	Filtered	Filtered	Filtered	Filtered	Filtered	Filtered	Filtered
15912.36133	18552.23242	17565.07617	19922.79492	13926.88477	13913.52344	14435.03906	14670.58789	16771.33789	23276.44141	18503.28125	12227.18945
8928.674805	12827.2002	13282.91699	15420.5752	16978.59961	46267.08984	9792.208984	11084.49609	17644.10547	11997.00195	13217.38672	11140.94629
17690.38086	16348.04395	15802.13184	18804.57813	17298.80078	16682.76172	16621.72656	20059.5957	24193.55078	16128.62207	14639.36426	19167.22461
10715.60352	21470.00586	25324.50391	20301.73242	10656.76855	9804.388672	19315.00586	8531.996094	13172.10645	10959.08496	15831.57715	14150.9834
11505.48828	19042.12109	15158.35547	16233.44629	18540.67773	16811.4668	24358.08008	21606.25391	24897.43359	19032.15625	16211.56836	17296.93555
Filtered	Filtered	Filtered	Filtered	3092.404541	3714.027588	Filtered	Filtered	Filtered	47557.69922	2096.645508	1423.478882
14307.93457	15493.56348	15359.62988	15084.40137	28291.99609	9490.453125	27569.82031	14080.58203	18476.72852	11200.7832	13719.33008	15334.10449
14572.99219	13017.05371	11730.08398	16751.5625	13973.27344	11488.90039	17683.49609	15599.1123	15936.09961	17977.91016	16332.95898	16589.35938
21819.7168	13083.94238	16248.33398	12091.15039	9183.216797	10948.86816	14251.73242	20054.27344	16553.0293	22135.33008	12288.79199	12860.33984
Filtered	12688.17871	Filtered	Filtered	32467.94922	Filtered	Filtered	13401.89453	Filtered	Filtered	Filtered	3015.275635
16745.44336	17181.36523	19342.94336	21153.3125	12495.6416	15985.42773	17224.71875	14981.25488	16752.01172	14401.83398	19602.82617	17066.88477
5188.837891	Filtered	Filtered	Filtered	22621.5293	Filtered	Filtered	18458.32422	Filtered	2326.08252	Filtered	Filtered
Filtered	14078.42383	Filtered	Filtered	34745.66016	6045.332031	Filtered	12152.60938	Filtered	8443.349609	Filtered	7598.875
6786.946289	4882.375977	14463.59961	15425.45215	41102.27344	4698.860352	19046.67383	9553.543945	11142.59277	7653.336914	9296.048828	6612.726563
18139.65039	14259.70215	16077.35059	21686.56641	10313.91406	13915.00879	14893.52246	9716.009766	15412.16992	12606.5625	13464.68164	19992.23242
21007.50195	18587.58203	17899.21875	15799.6543	12131.55664	18139.34961	17989.23633	15383.28516	18716.875	19940.58398	15248.15918	18136.5957
18859.82031	17540.92188	19223.07422	18726.7207	17384.62305	12177.2998	13538.7793	22731.13672	21044.64844	16040.45215	15453.51563	12851.13086
Filtered	1730.235107	Filtered	Filtered	28069.09375	Filtered	Filtered	Filtered	Filtered	Filtered	Filtered	Filtered
Filtered	Filtered	Filtered	Filtered	Filtered	Filtered	Filtered	14296.55078	Filtered	Filtered	Filtered	10161.69629
Filtered	Filtered	Filtered	Filtered	Filtered	Filtered	Filtered	Filtered	Filtered	Filtered	Filtered	Filtered
Filtered	Filtered	Filtered	26633.30664	20309.11719	Filtered	Filtered	4419.226074	Filtered	Filtered	Filtered	Filtered
Filtered	Filtered	Filtered	Filtered	Filtered	8381.8125	Filtered	16112.19336	3401.315186	Filtered	Filtered	18386.19531
Filtered	Filtered	Filtered	Filtered	Filtered	Filtered	Filtered	11577.95996	14491.64941	Filtered	Filtered	Filtered
15077.29102	15093.25098	20864.00391	16770.73438	11507.40234	12200.9375	13865.42383	19262.33594	18861.92578	17061.0293	14500.85449	12365.64063
4395.942871	20886.20703	28208.85156	30321.91602	9995.999023	17795.73047	15795.7207	4664.708008	13854.91699	5679.8125	8142.923828	13397.65625
12364.12793	13891.17578	15511.01563	17423.15625	14141.44922	13501.07031	15626.6416	14861.43457	13046.21191	13697.59375	14630.87402	11116.80859
Filtered	Filtered	2021.674927	21895.96484	2792.441406	12656.47852	Filtered	Filtered	27138.53906	14155.94141	22596.03711	Filtered

Filtered	Filtered	Filtered	Filtered	2632.814453	2875.474121	3926.204102	4239.307617	3065.031738	190489.2969	Filtered	2120.420898
10122.68359	Filtered	Filtered	Filtered	48355.85938	Filtered	Filtered	Filtered	Filtered	Filtered	Filtered	Filtered
19275.39648	9982.313477	11013.53906	10744.00488	10997.4043	15226.45313	16122.20117	21482.91602	11560.19531	16758.07617	10024.50586	11118.06348
18861.92773	13721.91602	17958.85742	19342.75586	15922.40625	14842.41211	9542.636719	15926.56836	10935.08398	13083.41699	13704.78613	15863.26367
Filtered	7903.133301	7868.255371	10030.90039	Filtered	Filtered	Filtered	8733.040039	5561.69043	10953.42773	6318.068359	6997.843262
5096.350586	17237.75195	6362.741211	Filtered	29953.0957	11039.40234	5911.724121	12384.84961	7615.066895	6653.756836	Filtered	7861.984863
17990.33984	15276.76758	16092.31445	13840.31445	16447.40234	16639.35156	Filtered	12145.33008	14600.18945	16720.06055	13175.41211	8505.920898
16471.30078	15489.34375	Filtered	16678.85156	10047.02734	14325.66992	17643.4082	19447.0293	13671.3877	16458.39063	12771.7002	15542.4209
16292.00098	15865.74023	17163.39648	14615.35352	12088.45313	11060.63281	15936.50586	13083.52539	15413.22754	15733.70703	10229.47949	15792.53906
13686.36426	13951.38379	10696.41504	14147.5332	18418.20898	15918.17871	15184.43652	14153.87891	20309.94922	14139.86719	10792.74023	8123.625
11651.75098	6760.080078	7929.22168	3518.447021	15658.37891	6626.588867	13504.74023	11608.32617	10964.34375	16328.65234	9440.384766	11512.22656
Filtered	Filtered	Filtered	Filtered	Filtered	7.23E+03	Filtered	7.43E+03	Filtered	Filtered	Filtered	Filtered
15662.45996	12917.58789	13974.31348	19604.1875	13123.66406	13848.08691	14541.11035	15576.08398	18101.25586	11473.43262	15768.71875	14883.07422
Filtered	Filtered	6917.849121	Filtered	Filtered	Filtered	Filtered	Filtered	Filtered	Filtered	Filtered	Filtered
17165.73047	13287.9375	17961.16797	12904.10449	13630.02148	16879.86719	12583.83691	17041.45703	12799.56641	16413.31836	10808.06934	16933.42578
Filtered	Filtered	14217.12988	Filtered	Filtered	Filtered	Filtered	Filtered	16191.97461	Filtered	13452.9502	Filtered
Filtered	Filtered	Filtered	Filtered	3810.033936	15463.52246	Filtered	Filtered	14319.51563	Filtered	Filtered	Filtered
12670.81445	10045.45117	8184.996582	8002.851563	4915.213867	6205.893066	7258.642578	5065.831055	8413.049805	8273.568359	7606.23291	6434.186523
15428.50781	11576.57617	17971.91406	18981.94531	7838.300293	17380.14258	11370.99805	11585.63672	11751.44238	14600.45996	18147.52734	13569.21484
Filtered	26488.74023	Filtered	Filtered	55411.04297	Filtered	Filtered	Filtered	Filtered	3140.086914	Filtered	Filtered
Filtered	6057.542969	8641.605469	Filtered	3784.275635	Filtered	18686.95313	Filtered	2368.5896	Filtered	1998.97998	9202.452148
Filtered	4417.871582	Filtered	7407.14502	4496.118164	5364.275879	6868.003906	6486.140625	7790.07666	5001.114258	4876.674805	4364.338867
Filtered	Filtered	Filtered	Filtered	14400.98633	Filtered	Filtered	Filtered	Filtered	Filtered	Filtered	Filtered
10525.84863	8568.998047	17982.64063	18149.05469	8395.504883	25609.51367	10840.37109	19180.22656	16450.97852	14234.64258	17410.5	7231.654297
13542.54883	10022.40039	Filtered	11621.41699	11565.60156	Filtered	Filtered	17871.48242	Filtered	15643.04297	18632.56836	18967.82031
Filtered	Filtered	Filtered	Filtered	17072.92773	Filtered	Filtered	Filtered	Filtered	Filtered	Filtered	Filtered
7802.593262	4963.062988	9447.875	8745.935547	7354.087891	Filtered	7685.249023	9108.09668	6780.401367	7509.271973	4906.480957	4812.056641
13863.61523	15484.14258	13469.63379	15113.82227	12594.07129	17509.21484	14005.33984	10731.05859	14001.21484	12276.20996	15148.22949	12500.27051
16006.10449	11917.95313	11028.80176	16647.38672	9572.238281	12363.03125	16932.58789	14804.25195	14178.62305	11768.42969	15572.50195	15241.95215
Filtered	Filtered	Filtered	Filtered	10417.74609	Filtered	Filtered	Filtered	Filtered	Filtered	Filtered	7945.378418
8702.524414	12758.10938	12681.74316	14221.14746	18762.78516	10989.25195	14887.74023	9369.634766	9555.43457	8089.265137	8906.463867	9148.694336
8441.245117	4533.40625	Filtered	4299.20459	47275.18359	Filtered	Filtered	1928.700562	Filtered	Filtered	Filtered	Filtered
21430.44141	17286.01953	13232.52539	26464.9707	8087.753906	10707.95117	12095.23242	10375.7627	19585.69141	19768.29102	16822	11759.77344
9059.786133	16334.56543	18964.63672	17412.16797	18272.21484	14422.67969	15741.2002	10177.75098	31133.93164	12361.2168	8961.007813	13130.5791
12451.05664	16906.74805	19063.28516	17464.79688	14859.75977	23484.38867	11843.75391	20197.81641	10606.63281	13322.94336	10798.37207	16203.80078
Filtered	15607.19922	17944.48438	11347.04199	10279.41113	7293.350098	13537.39648	17122.27148	20456.42773	Filtered	19098.95703	Filtered
7534.855469	18560.75977	4678.024902	Filtered	6991.617676	7731.205078	12835.59375	12152.94531	16155.34375	18258.10156	13642.50879	20155.5957
6705.219727	970.0785522	2221.623047	4891.032715	Filtered	Filtered	3906.556885	2704.554688	2745.675781	6455.535645	6671.29834	Filtered
26591.51758	5042.008301	8556.714844	6945.916016	4828.149902	5876.875977	7054.70166	2844.187744	7429.466309	5615.089844	4960.433105	6139.985352
7437.749023	9576.206055	16316.63672	10884.73535	18719.04102	5786.091797	18393.36914	8015.812988	10854.05469	5206.833496	8936.24707	11796.60547
Filtered	Filtered	Filtered	16656.44336	Filtered	Filtered	Filtered	Filtered	Filtered	Filtered	Filtered	5691.015625
Filtered	3600.16333	9366.287109	6807.358398	Filtered	Filtered	Filtered	Filtered	Filtered	Filtered	1340.293213	Filtered
Filtered	Filtered	Filtered	Filtered	20719.78125	Filtered	Filtered	Filtered	Filtered	Filtered	Filtered	Filtered
12775.58105	15746.50977	9900.901367	2722.557129	Filtered	5433.73291	11825.72754	13424.50977	15924.16895	12402.37402	17787.15625	Filtered
Filtered	Filtered	Filtered	Filtered	11759.79102	Filtered	Filtered	Filtered	Filtered	Filtered	Filtered	Filtered
9358.591797	10532.53906	12515.69629	11712.0752	13026.8418	5818.208008	7947.804688	14781.09863	10276.5293	Filtered	12429.94434	8276.970703
Filtered	Filtered	Filtered	Filtered	Filtered	Filtered	2410.591553	Filtered	2893.915283	Filtered	Filtered	8539.97168
3117.38501	2907.13208	Filtered	2577.143311	17353.83008	Filtered	7748.932129	3745.120605	4619.272461	Filtered	6536.600586	9296.456055
9008.314453	7652.291992	18387.45508	Filtered	20369.20313	Filtered	23670.91211	10121.38965	13351.25586	3390.013672	10253.37305	8553.777344
6631.741211	11500.52344	16798.87695	15658.27734	10627.80371	14411.88867	11453.04297	12690.25	10501.85059	9406.787109	15901.30176	10447.85254
9810.52832	5017.039063	15464.75098	13718.4043	12350.24023	11955.08789	11617.57422	12024.18262	10741.72559	18540.51758	9430.323242	26598.19727
13748.41992	11200.72949	10646.38281	12257.56445	7190.421875	13326.52344	10125.85059	10118.36816	12340.45703	12427.5625	7990.422852	13010.85645
12686.44336	13702.44141	11302.26855	12173.13965	11491.92285	8766.945313	12803.76367	14761.93359	16821.36719	11121.11523	12646.04395	14636.9209
8517.50293	8852.983398	11896.08398	13099.85938	10723.64551	7708.797852	12240.21973	11549.82715	9229.258789	13916.13477	10873.2998	6717.182617
Filtered	Filtered	Filtered	Filtered	4367.796875	Filtered	Filtered	Filtered	Filtered	13430.66602	Filtered	Filtered
11559.9668	9455.376953	11860.84082	12435.4043	14384.34375	12723.9248	11253.03711	11148.09766	10551.09961	8855.191406	16482.95117	12553.17773
10008.34473	9886.350586	13550.79492	11572.91016	3502.151367	7177.544434	23440.14258	8334.438477	10403.55078	10466.52539	15461.44531	12618.81055
9311.834961	8274.103516	8832.432617	Filtered	Filtered	8918.874023	19636.43164	8615.869141	5757.98584	13391.1709	13223.99805	9259.710938
Filtered	8139.649414	14701.68262	14725.61133	21998.26953	Filtered	12256.24512	14424.5	16186.23926	15184.4541	17518.23242	2531.962402
5533.413086	15215.96094	12158.72852	Filtered	27825.32617	7764.924805	13934.52539	4402.925781	8994.738281	Filtered	Filtered	5938.785156
Filtered	Filtered	Filtered	Filtered	5018.063477	Filtered	6683.353027	3167.214844	Filtered	7577.875488	3207.516846	Filtered
4280.518066	2021.294189	10206.77832	2184.132813	4335.32959	6545.763672	3514.851807	Filtered	Filtered	Filtered	Filtered	Filtered
11086.58008	9955.914063	12052.58691	13479.61621	10801.46191	12215.3916	12058.94727	10401.27148	10282.35742	9825.248047	10628.75195	13189.79297
1147.965454	2693.128662	Filtered	Filtered	4113.787598	1965.842407	4203.870605	Filtered	2644.90918	1318.622192	27245.05859	2221.832275
8603.013672	13811.80371	8248.286133	10919.68066	11748.0791	13082.5293	9225.996094	8107.19873	9473.842773	7150.554199	7099.259277	10091.43164
13605.85547	3001.522705	4455.449707	10340.11621	2103.750488	3211.436279	12703.12402	2670.597168	2110.565918	2929.668945	5180.163086	2936.30249
8495.368164	18045.10352	16328.05664	17122.02344	13830.34961	16099.9873	15922.80664	13789.22852	14015.38477	10319.95703	10314.48535	15981.13574
8916.360352	7866.032715	7809.753906	15819.37109	9261.955078	11482.71582	10564.81348	17686.60547	15436.76465	10587.29395	11365.36914	7245.945801
14615.74219	6653.500977	Filtered	9912.195313	7586.102051	5677.944336	10310.93848	11381.17285	13831.2959	Filtered	Filtered	6802.447266
9062.080078	12151.30664	13640.73242	Filtered	24026.51563	5198.188477	20308.38281	5234.026367	9479.9375	Filtered	5184.191406	Filtered
5251.371094	5482.918945	7370.155273	7972.669434	7700.536133	5877.375488	5647.064453	4515.695313	5603.542969	5242.92334	5653.671387	64369.66406
6938.181641	5451.026367	14794.38281	7643.93457	4538.15918	Filtered	13624.40234	5372.923828	6557.661133	4300.468262	3207.901855	5465.412598
Filtered	Filtered	Filtered	Filtered	Filtered	Filtered	Filtered	Filtered	Filtered	Filtered	Filtered	Filtered
Filtered	Filtered	15150.93262	Filtered	Filtered	Filtered	Filtered	Filtered	Filtered	Filtered	Filtered	Filtered
Filtered	Filtered	Filtered	Filtered	Filtered	Filtered	Filtered	Filtered	Filtered	Filtered	Filtered	Filtered

11110.00488	7451.758789	14903.44922	Filtered	14862.40234	13422.4082	7643.297852	Filtered	3384.722412	6783.641113	4354.430664	9883.848633
Filtered	Filtered	9441.527344	12256.00781	8120.116211	10267.7373	15281.58203	13517.78809	7736.102539	8817.588867	14050.34961	4870.032715
6265.131348	3521.521729	12675.72266	8932.390625	9765.380859	5106.254395	18406.61914	9835.201172	5270.624512	5236.52832	23679.38672	8060.410645
Filtered	6126.744141	Filtered	8351.000977	27216.41406	Filtered	Filtered	Filtered	Filtered	Filtered	Filtered	Filtered
Filtered	Filtered	Filtered	Filtered	10603.73145	Filtered	10112.60059	Filtered	5807.117676	Filtered	Filtered	Filtered
Filtered	9236.086914	6920.019531	8796.823242	8726.652344	8529.134766	16736.18555	7055.787598	10555.32715	11121.32324	11048.83789	12909.68066
Filtered	10391.72852	Filtered	Filtered	12580.28906	4598.566406	Filtered	Filtered	Filtered	Filtered	10628.62695	12543.06348
Filtered	Filtered	Filtered	Filtered	Filtered	Filtered	Filtered	Filtered	Filtered	Filtered	Filtered	Filtered
5091.693848	6946.250977	11805.36621	7155.934082	15027.86426	6378.875488	12641.21289	6835.052246	8152.877441	5075.083984	7309.087402	7252.436523
9105.178711	9893.804688	10611.54688	12996.20215	9932.660156	9962.808594	15249.38867	17009.67969	14076.32422	10759.87402	17243.96875	11200.62305
12215.27344	7629.482422	8653.444336	15239.41797	11222.26367	5051.157227	9764.508789	12527.18164	5313.506836	10416.40918	7449.390137	8843.18457
7332.263672	8854.571289	6779.467773	11999.25488	7279.883301	1653.934082	10756.44141	12230.66992	10834.85156	9786.482422	10451.91797	Filtered
Filtered	Filtered	Filtered	Filtered	12541.80371	Filtered	Filtered	Filtered	10983.1416	Filtered	Filtered	Filtered
9373.850586	10621.42871	8027.571289	12410.07227	7006.705566	9194.868164	13297.22168	10936.46875	11366.60645	5984.092773	10898.3457	10427.37012
6574.033691	5482.852051	11515.51758	6282.746094	8528.610352	5570.76709	10766.84082	6762.97998	8500.515625	5065.246094	10084.25098	7996.379883
Filtered	5956.910645	5826.170898	4798.129883	7536.293457	9006.836914	9158.990234	11658.11816	Filtered	Filtered	5001.130371	11341.45703
Filtered	7310.271484	9418.070313	10479.54297	Filtered	9169.839844	11121.02441	10900.75098	14456.8916	10605.60938	Filtered	11959.61426
8387.738281	8556.712891	11062.13477	10685.1582	6105.149902	7159.905762	10267.10938	7532.845703	8067.109375	6863.40625	8175.510742	5666.519531
2949.611084	3731.344727	3481.989746	24711.84766	Filtered	2233.821777	20570.67578	4151.160156	3501.345459	20616.98047	24232.85156	Filtered
9261.854492	12261.83789	14295.40527	10341.46777	15081.59473	4366.491699	9836.217773	Filtered	10883.16992	9749.345703	11336.99121	Filtered
7050.071289	10242.36328	9300.332031	9170.607422	6736.098633	Filtered	9457.260742	11892.55957	5098.621094	6104.583008	10863.9668	9307.398438
Filtered	Filtered	Filtered	Filtered	14133.98047	Filtered	Filtered	4952.398926	Filtered	Filtered	Filtered	Filtered
Filtered	Filtered	5453.492676	12803.68066	5959.128418	Filtered	8152.672363	Filtered	2135.999756	3972.689697	4353.432617	4966.354492
7887.405762	12045.15918	Filtered	Filtered	Filtered	6611.553711	8218.299805	10945.86914	Filtered	10199.40723	Filtered	13068.49902
Filtered	14667.36328	8664.3125	9482.878906	7082.003418	Filtered	12608.47949	3641.600342	6768.262695	Filtered	6450.924805	Filtered
7942.181641	7879.226074	10024.3291	12667.93848	9445.483398	7548.966309	10100.43945	12793.17188	11919.66992	8036.310547	8244.444336	9241.128906
Filtered	Filtered	Filtered	Filtered	Filtered	Filtered	Filtered	Filtered	Filtered	Filtered	Filtered	Filtered
Filtered	4806.971191	12013.15527	Filtered	14026.95313	Filtered	18206.35156	3644.179199	8163.980469	Filtered	Filtered	4924.94043
7832.279297	9321.517578	18888.82227	9613.930664	6675.689941	4799.593262	8584.486328	7613.226563	8443.887695	8245.854492	10906.93945	11971.71973
Filtered	8175.661133	7840.871582	7092.497559	10551.27441	5803.683594	14295.12109	Filtered	10378.78516	8240.548828	9202.022461	Filtered
16024.48535	Filtered	9287.797852	10129.73145	8629.513672	9227.52832	5150.373047	Filtered	13509.31934	5720.416992	6152.443848	2508.551758
9858.261719	Filtered	9881.854492	Filtered	Filtered	Filtered	Filtered	Filtered	6422.205566	Filtered	7965.397949	Filtered
Filtered	Filtered	Filtered	Filtered	Filtered	Filtered	Filtered	Filtered	Filtered	Filtered	Filtered	Filtered
9810.75	7289.64209	9128.953125	13098.61426	Filtered	7418.616211	4235.542969	9827.977539	10273.13086	9948.549805	9400.727539	8272.947266
4322.671387	7736.123535	7105.447266	9577.552734	8599.041016	8741.84668	7419.647461	4506.019531	5895.16748	7173.070313	Filtered	7906.942871
6429.103516	10330.61914	8809.073242	7870.555176	13393.1123	7069.421875	4536.571289	9691.287109	9702.049805	6535.469238	12090.29102	8678.604492
8707.198242	10212.83594	6558.746094	11984.32031	7122.192383	9739.615234	7638.638672	12123.99805	10487.0625	Filtered	8007.540527	7433.200684
11071.05664	6069.296875	12694.2793	11453.01855	10749.42285	9389.839844	7040.113281	6680.066895	6665.225586	3913.490723	7583.854492	6163.555664
7682.562012	6865.374023	10309.54492	13159.87012	8647.993164	7341.039551	8785.170898	7388.317383	10928.35645	22164.59375	9605.012695	9653.375977
7041.571289	12731.84961	8001.90332	12548.93164	11511.76563	8629.444336	8858.487305	7041.512695	11410.36328	9506.945313	11848.39648	6047.591309
Filtered	Filtered	Filtered	Filtered	Filtered	Filtered	9978.183594	Filtered	Filtered	Filtered	6287.103027	Filtered
7257.779785	6421.080566	13480.82617	12645.28613	9629.96582	9197.428711	8261.4375	8951.956055	10551.23242	8292.413086	7815.724609	11242.05273
4406.679688	7691.443848	12444.29688	15427.08008	9356.496094	4552.970703	8884.456055	8058.825195	8359.289063	10188.79883	12712.95313	12791.2666
Filtered	8193.194336	11582.99316	7898.264648	9797.203125	8076.4375	Filtered	11326.39258	8169.238281	8398.947266	8277.250977	Filtered
5318.227051	1478.300293	Filtered	14344.80664	Filtered	Filtered	Filtered	Filtered	Filtered	6445.976074	8667.891602	Filtered
Filtered	Filtered	Filtered	Filtered	8064.591797	Filtered	Filtered	Filtered	Filtered	Filtered	Filtered	Filtered
8432.924805	3123.125977	9316.887695	9161.375	9422.957031	10034.57715	7124.865234	11489.50098	4903.207031	5521.597656	11528.13086	8516.324219
Filtered	Filtered	Filtered	Filtered	8454.006836	Filtered	Filtered	Filtered	Filtered	Filtered	Filtered	Filtered
Filtered	9363.145508	8295.852539	Filtered	Filtered	5635.525391	8321.708984	Filtered	10460.68848	Filtered	12068.22949	Filtered
4703.044434	2694.453613	Filtered	Filtered	2273.601807	3513.105225	2832.01709	4308.84668	4854.547852	3560.580566	5365.876953	3641.765137
7678	13282.81641	7805.911621	Filtered	17446.70508	5600.224609	9461.837891	4833.297852	Filtered	Filtered	1787.088623	Filtered
8729.413086	12042.95996	8306.194336	8321.751953	6005.220703	5440.507813	8404.055664	8664.966797	8575.59668	7014.263672	6584.48584	7354.700195
Filtered	Filtered	1795.209839	Filtered	Filtered	Filtered	5872.563477	9860.402344	7523.794434	7781.638672	10238.77539	6596.24707
Filtered	8685.813477	10652.16211	9716.912109	8031.946289	2010.664917	11570.11719	Filtered	6202.007813	3889.524658	9539.641602	8526.178711
9043.175781	7590.991211	8548.379883	11424.80469	17362.25195	8583.049805	8325.380859	4554.981445	8913.199219	9303.663086	5881.354004	3621.550781
12178.03223	6867.377441	11600.20117	10902.49609	7709.610352	5629.331055	6981.124023	10151.10742	7270.273926	8256.845703	10602.94141	10198.33594
4949.519531	8122.911621	4326.47998	3304.305176	Filtered	16779.4375	8017.501465	19433.29492	7116.426758	7704.31543	3896.889893	3099.880859
1986.025757	Filtered	Filtered	Filtered	13158.76074	Filtered	Filtered	Filtered	Filtered	Filtered	Filtered	4324.467285
Filtered	Filtered	8474.603516	10111.99414	9170.085938	7858.872559	3773.48877	7064.97168	Filtered	9512.666992	Filtered	8599.576172
Filtered	Filtered	Filtered	Filtered	Filtered	Filtered	Filtered	Filtered	Filtered	Filtered	Filtered	Filtered
5373.334473	5528.586426	6938.399902	5847.128906	13632.69629	7331.960449	5097.870117	6362.936523	15668.29688	5358.116699	14185.05469	5562.834961
Filtered	Filtered	Filtered	Filtered	Filtered	Filtered	Filtered	Filtered	Filtered	Filtered	Filtered	Filtered
3559.97583	3388.366211	6189.539063	6845.895996	23611.33203	1960.960938	Filtered	Filtered	Filtered	Filtered	3542.071533	5899.795898
8625.483398	8758.657227	9283.605469	11747.42383	6893.592773	10268.85645	8582.224609	6586.070801	8759.374023	7712.505371	7246.975586	7287.654297
Filtered	7967.887207	4337.945313	Filtered	Filtered	Filtered	4582.674805	Filtered	Filtered	Filtered	Filtered	Filtered
5445.737793	Filtered	Filtered	Filtered	8788.219727	5597.57373	9478.125	Filtered	7779.470215	Filtered	Filtered	9178.632813
Filtered	Filtered	Filtered	Filtered	Filtered	Filtered	Filtered	Filtered	Filtered	Filtered	Filtered	Filtered
5206.677734	7658.68457	8397.679688	14864.5293	7519.394043	9169.236328	7887.248535	Filtered	7623.665039	6506.956055	7353.044434	8161.794434
Filtered	Filtered	4340.832031	6347.114746	6807.397949	Filtered	6270.838867	Filtered	Filtered	5894.833496	5484.794922	Filtered
Filtered	5355.241211	Filtered	7125.257324	27237.40234	4040.155029	Filtered	Filtered	Filtered	Filtered	4064.831787	1145.502441
3121.043945	6925.742188	7266.922852	9270.68457	2466.827637	5877.466309	4640.048828	14950.94336	15942.50391	6633.739746	8626.446289	8393.123047
Filtered	7828.731934	9323.978516	11010.87305	5663.022461	8918.467773	7717.644531	7485.083496	9469.293945	6014.566895	7175.760742	8160.572754
8959.857422	6325.31543	7798.253906	7285.286133	8025.744629	6628.443848	8036.57959	6485.343262	9244.539063	8759.44043	8932.960938	Filtered
Filtered	Filtered	Filtered	Filtered	Filtered	3008.722412	Filtered	8597.983398	Filtered	Filtered	12817.13184	Filtered
9228.022461	Filtered	7703.355957	Filtered	6701.588867	7071.681641	6961.04541	Filtered	Filtered	Filtered	Filtered	Filtered

8373.393555	6590.915039	8670.327148	4503.645508	6353.046875	5902.477539	9982.500977	11230.3877	8851.09375	7824.590332	7501.435547	7117.476074
Filtered	3761.331787	4195.393555	Filtered	16895.11328	Filtered	11886.74121	4968.46582	Filtered	Filtered	5188.404297	Filtered
8539.392578	4528.662109	3835.869629	6601.460938	5295.128418	7018.029785	6509.935547	8377.318359	6324.714355	7473.32373	7004.618164	4334.087891
6965.683594	5071.282227	7726.765137	6004.789063	5371.591797	5996.300781	6731.681641	7292.90332	6631.533691	6443.130859	6629.183105	5505.664551
Filtered	Filtered	6980.156738	9584.672852	6238.232422	3903.878906	5197.304199	Filtered	Filtered	Filtered	Filtered	5911.90625
5823.094727	8072.913574	5824.239258	5356.154297	8257.97168	5004.256836	7615.130371	8289.111328	8105.753418	6309.102539	6520.69873	4172.790527
7149.144043	Filtered	6985.852051	7257.421387	7104.216797	10477.40234	3016.729492	1918.821777	Filtered	3803.333984	Filtered	Filtered
Filtered	Filtered	9802.941406	Filtered	8076.313965	Filtered	10233.29688	Filtered	Filtered	Filtered	Filtered	Filtered
Filtered	7669.114746	4841.884766	10322.99902	8230.923828	1193.087646	Filtered	6490.626953	8828.947266	8077.804199	12188.21484	8336.203125
Filtered	Filtered	Filtered	5362.92334	17469.54297	Filtered	Filtered	Filtered	Filtered	Filtered	Filtered	Filtered
Filtered	Filtered	4322.017578	10104.72754	Filtered	10053.79785	Filtered	9939.120117	7422.663086	Filtered	12554.03809	7323.631836
Filtered	Filtered	Filtered	Filtered	Filtered	Filtered	Filtered	Filtered	Filtered	Filtered	Filtered	Filtered
Filtered	Filtered	Filtered	Filtered	11179.62793	1839.102905	Filtered	11434.33887	Filtered	Filtered	1342.981323	Filtered
Filtered	Filtered	Filtered	Filtered	Filtered	Filtered	Filtered	5273.732422	Filtered	Filtered	9642.457031	Filtered
4053.22583	Filtered	Filtered	Filtered	3660.117432	3714.412354	4944.348145	Filtered	Filtered	2715.585449	4824.163086	4646.402832
Filtered	Filtered	5794.300293	2538.908936	4402.866699	7205.308594	6847.547363	6698.211914	7216.063965	Filtered	Filtered	2109.658447
5088.496582	6196.358887	8192.271484	Filtered	10050.68848	Filtered	2211.163086	Filtered	7373.139648	4509.274414	6192.466797	6953.169922
8039.180176	5245.16748	3658.503174	1886.342163	1273.595581	5237.014648	Filtered	Filtered	5990.781738	Filtered	8516.178711	4738.042969
Filtered	2815.802002	Filtered	4881.345215	Filtered	1835.886475	5816.359863	Filtered	Filtered	Filtered	4841.898438	4447.297852
Filtered	Filtered	5495.433594	Filtered	5162.837891	Filtered	8592.567383	Filtered	6207.323242	Filtered	Filtered	Filtered
Filtered	Filtered	Filtered	Filtered	8369.164063	Filtered	Filtered	3463.932129	Filtered	Filtered	Filtered	Filtered
5004.872559	6099.107422	7673.697266	7511.091797	6948.063965	5016.359375	Filtered	6154.034668	8749.259766	Filtered	6270.898926	6739.461914
4675.628906	7233.114258	4137.72168	4581.6875	6901.344238	7496.38916	Filtered	5491.147461	3854.308838	4610.218262	4693.574219	2967.539551
6444.208008	Filtered	Filtered	Filtered	8835.021484	7673.708984	5163.291992	6756.483398	Filtered	3654.216797	Filtered	Filtered
Filtered	Filtered	8787.305664	Filtered	5638.948242	6998.98291	6666.127441	5628.710449	9451.010742	7117.099121	7519.924316	5345.150879
5110.375977	6166.374023	5566.746094	5385.128906	4281.490234	4134.017578	5096.695313	4055.895508	5949.563477	7016.972168	5921.390137	5229.3125
Filtered	7797.917969	Filtered	Filtered	2309.915527	4421.233887	Filtered	Filtered	4434.621094	7048.884277	Filtered	Filtered
Filtered	Filtered	Filtered	Filtered	Filtered	Filtered	Filtered	Filtered	Filtered	Filtered	Filtered	Filtered
7744.815918	5760.936523	8682.606445	9347.581055	6254.346191	7255.630859	6056.175781	4141.451172	6471.373535	4672.450195	8745.286133	8372.225586
Filtered	Filtered	Filtered	Filtered	7495.326172	Filtered	Filtered	Filtered	Filtered	Filtered	Filtered	Filtered
Filtered	Filtered	Filtered	Filtered	4883.746582	Filtered	6819.441406	Filtered	Filtered	Filtered	8397.484375	5085.828613
5237.257324	5141.15918	3760.712402	4312.885742	14835.16309	Filtered	2985.112793	4987.728516	4660.186035	5503.752441	30056.61133	Filtered
8610.856445	Filtered	5929.847168	8104.432617	8361.101563	4281.227539	8159.930176	9680.405273	5734.955566	8386.770508	3931.434326	5759.286621
4893.978027	5233.658691	8164.077148	5363.460449	5399.165527	Filtered	4355.494141	Filtered	4042.383301	5716.544434	11352.68164	4891.044922
10468.63184	Filtered	Filtered	Filtered	9455.822266	Filtered	4277.78125	Filtered	Filtered	Filtered	Filtered	Filtered
4199.458496	5716.913086	7819.844727	6791.601074	8697.791016	4569.82959	9193.78125	4296.418945	7432.628418	5746.066895	5853.365723	5881.81543
2985.781006	4350.607422	5674.125	4297.884766	4323.230957	5951.543945	Filtered	Filtered	4891.180664	Filtered	Filtered	7358.660156
Filtered	Filtered	Filtered	Filtered	5739.104492	Filtered	Filtered	Filtered	Filtered	Filtered	Filtered	Filtered
5196.65918	5724.667969	5941.039551	5784.791992	4325.835938	4692.748047	5448.011719	4857.136719	5761.924805	6178.029297	5011.509277	6120.477051
Filtered	5390.862305	6636.655762	6436.118164	4602.85791	5122.854492	2107.823242	Filtered	4464.530273	Filtered	4710.296875	Filtered
5739.623535	5280.411621	Filtered	4458.469727	Filtered	Filtered	Filtered	Filtered	3416.958008	Filtered	5991.6875	6905.719238
Filtered	6610.278809	9134.629883	13539.91602	766.3966675	4931.133301	3289.661865	Filtered	4840.076172	Filtered	Filtered	6508.479004
Filtered	Filtered	Filtered	Filtered	Filtered	Filtered	Filtered	Filtered	6881.447754	Filtered	Filtered	Filtered
Filtered	3262.767334	3904.133301	Filtered	4525.321777	3346.29126	4607.274902	5393.399414	5222.514648	Filtered	4164.053711	Filtered
Filtered	Filtered	Filtered	Filtered	3549.224365	Filtered	Filtered	Filtered	Filtered	Filtered	Filtered	Filtered
Filtered	Filtered	Filtered	Filtered	Filtered	Filtered	Filtered	Filtered	Filtered	Filtered	Filtered	Filtered
Filtered	Filtered	4457.230469	Filtered	4045.366455	Filtered	Filtered	Filtered	5364.085449	Filtered	Filtered	5027.701172
Filtered	Filtered	4112.709473	Filtered	19026.68359	Filtered	6086.733887	Filtered	Filtered	Filtered	Filtered	Filtered
Filtered	Filtered	5496.161133	Filtered	5723.208496	4053.477783	7644.329102	Filtered	3199.286377	Filtered	Filtered	Filtered
2549.836426	5806.113281	3915.687012	Filtered	5978.01416	Filtered	8637.820313	Filtered	3404.55249	Filtered	Filtered	Filtered
5753.25293	5856.007813	6678.281738	7171.214844	5480.160645	6804.549316	6989.649414	5668.713867	6658.724609	3924.875732	5329.133301	6360.620605
Filtered	Filtered	4038.827148	Filtered	Filtered	2470.891113	Filtered	11966.38281	Filtered	Filtered	11550.86914	4074.884766
4452.163086	4415.202148	6722.02002	6094.66748	15542.13379	3374.247803	6729.347656	Filtered	4289.750977	Filtered	5111.913086	Filtered
4691.963867	5139.522461	13710.5957	5146.36084	5581.894531	4388.26123	4157.628418	5435.261719	5717.589844	4385.266602	7142.426758	5297.912109
5441.900391	5076.711914	4522.458984	5188.751465	4279.821289	3857.202881	7175.927734	6556.672363	4186.54541	5340.132813	3889.962158	7560.396973
5861.333984	Filtered	Filtered	Filtered	Filtered	Filtered	Filtered	Filtered	Filtered	Filtered	Filtered	Filtered
Filtered	Filtered	Filtered	Filtered	8223.905273	Filtered	Filtered	Filtered	Filtered	Filtered	Filtered	Filtered
4842.327148	5275.428223	7428.640625	Filtered	4098.53125	5897.059082	5289.091797	5471.060547	4325.930176	3425.907959	4103.04541	4609.149414
6550.133301	Filtered	5254.267578	Filtered	5283.97168	7281.273438	Filtered	Filtered	7394.612793	5974.552734	Filtered	Filtered
2509.275635	4056.969482	7502.977539	3537.790039	8618.115234	5012.084961	6922.538086	4974.618652	1705.268188	Filtered	3266.588379	4684.291992
6402.145508	Filtered	7080.455078	8274.746094	3827.964844	3428.499023	Filtered	5683.739258	6240.967773	3987.189697	4194.077637	6102.237793
4696.317871	3513.453125	4749.504395	Filtered	11911.13477	3141.891846	8266.322266	3796.767334	4100.717773	Filtered	Filtered	2748.410645
3839.032959	Filtered	3681.997559	5183.763672	2946.256104	4288.298828	1905.484009	Filtered	Filtered	Filtered	Filtered	Filtered
Filtered	Filtered	Filtered	Filtered	Filtered	913.0365601	Filtered	Filtered	Filtered	Filtered	Filtered	Filtered
Filtered	Filtered	Filtered	Filtered	Filtered	Filtered	6279.249512	Filtered	Filtered	Filtered	Filtered	Filtered
3418.144043	5153.492676	6039.662109	6385.098633	4216.848633	1719.834351	7834.679688	5128.048828	7741.555176	6472.847656	3518.940918	6264.311523
4041.807129	4897.233398	6931.335449	Filtered	15756.22266	2161.466553	6308.501465	3650.163574	Filtered	Filtered	3554.204346	3187.262451
Filtered	Filtered	Filtered	11528.0332	4406.779785	Filtered	Filtered	Filtered	Filtered	Filtered	Filtered	Filtered
3781.560059	Filtered	Filtered	6114.395996	Filtered	Filtered	Filtered	Filtered	Filtered	Filtered	Filtered	Filtered
Filtered	3541.696289	5300.331543	2745.598877	3241.240967	5086.367676	4752.502441	6244.982422	4081.801025	5647.759766	5295.092285	Filtered
Filtered	3200.331055	5367.404785	4392.805176	2718.582764	3717.150391	Filtered	3642.053223	3446.373047	4166.642578	4040.591797	4592.431641
5581.995117	3925.365479	6743.803711	6521.259766	5173.004883	5012.209473	5985.652832	6154.737305	4572.162109	3292.639404	6079.543457	6128.545898
Filtered	Filtered	Filtered	Filtered	3990.135498	Filtered	Filtered	Filtered	Filtered	Filtered	Filtered	Filtered
4575.144531	1690.762939	3031.69043	5843.307617	7193.687012	3724.103516	7061.107422	4531.552734	6315.181641	4109.65625	Filtered	3078.373047
Filtered	Filtered	3475.657227	6017.779785	3540.224121	5934.557617	Filtered	5589.408691	3286.973145	Filtered	Filtered	4056.451172

Filtered	Filtered	Filtered	Filtered	Filtered	Filtered	Filtered	Filtered	6868.381836	Filtered	Filtered	Filtered
Filtered	Filtered	Filtered	Filtered	4157.572266	Filtered	1747.411865	Filtered	Filtered	Filtered	Filtered	Filtered
5760.49707	3912.143555	Filtered	4900.70459	3786.766113	3525.540527	Filtered	6643.204102	Filtered	Filtered	6247.729004	Filtered
Filtered	Filtered	1133.996216	19777.23828	Filtered	Filtered	Filtered	Filtered	3867.577393	Filtered	3765.621094	4179.095703
Filtered	Filtered	Filtered	Filtered	2874.619629	Filtered	Filtered	Filtered	Filtered	Filtered	Filtered	Filtered
Filtered	Filtered	Filtered	4483.067871	Filtered	4817.203125	Filtered	Filtered	Filtered	Filtered	Filtered	Filtered
Filtered	Filtered	Filtered	Filtered	5430.664063	Filtered	Filtered	Filtered	Filtered	Filtered	Filtered	Filtered
Filtered	2937.131592	Filtered	Filtered	Filtered	2485.817871	4517.588867	2776.595703	7814.132324	6174.888672	6693.263672	2330.074951
4251.779297	3664.686523	3937.165039	4390.750488	4319.765137	2893.896973	4124.291992	4664.460938	5550.184082	4043.57959	4028.249023	Filtered
Filtered	Filtered	5072.027344	Filtered	Filtered	Filtered	Filtered	Filtered	Filtered	Filtered	Filtered	Filtered
Filtered	Filtered	4939.611328	Filtered	3626.08374	Filtered	Filtered	Filtered	Filtered	Filtered	Filtered	3628.175781
Filtered	Filtered	3797.088623	Filtered	Filtered	Filtered	Filtered	Filtered	Filtered	Filtered	Filtered	Filtered
Filtered	Filtered	4512.066406	6941.937012	4096.608887	3996.671631	Filtered	5427.564941	4852.220703	4490.625977	Filtered	3667.554932
6279.353027	2793.104736	4790.771973	Filtered	8235.208008	2526.021484	5279.241211	6867.885254	2746.911377	Filtered	Filtered	2457.733643
Filtered	Filtered	Filtered	Filtered	Filtered	2787.919434	Filtered	Filtered	1356.372437	Filtered	3150.926758	Filtered
Filtered	Filtered	5235.567871	Filtered	Filtered	Filtered	Filtered	Filtered	Filtered	Filtered	Filtered	Filtered
2938.749023	3753.201904	5182.614258	3181.363037	Filtered	3567.765137	3594.241455	4446.008789	2299.927246	4070.209229	1265.561401	2498.190186
Filtered	Filtered	Filtered	Filtered	Filtered	Filtered	Filtered	Filtered	3258.367676	Filtered	Filtered	Filtered
Filtered	Filtered	Filtered	Filtered	Filtered	Filtered	Filtered	Filtered	Filtered	Filtered	Filtered	Filtered
Filtered	Filtered	Filtered	Filtered	951.0093994	Filtered	Filtered	Filtered	Filtered	Filtered	Filtered	911.6333618
1738.970337	4416.89502	6247.589844	3120.773926	4501.293945	Filtered	3355.243408	1882.164917	2898.975342	Filtered	4240.790039	3242.310303
2931.377441	4919.489746	5485.613281	4937.688477	5264.756348	3569.05127	3463.521729	3610.113037	3884.15625	3609.389893	4003.389648	4217.953125
3556.471191	3208.104492	4906.993164	4642.099121	3710.301758	3318.129395	3812.891113	4701.374023	3413.22998	3431.683838	5754.880859	3641.512207
Filtered	Filtered	Filtered	Filtered	Filtered	Filtered	Filtered	Filtered	Filtered	Filtered	Filtered	1204.635376
Filtered	Filtered	Filtered	Filtered	Filtered	666.4396362	Filtered	Filtered	Filtered	Filtered	Filtered	Filtered
3268.084229	3196.602295	1908.102539	Filtered	2869.754395	4616.40332	4930.882813	5374.487793	4901.757324	3782.438477	3458.138428	4171.987305
Filtered	Filtered	Filtered	2357.596924	Filtered	Filtered	4130.75	4364.149902	Filtered	2800.365723	2867.963623	Filtered
Filtered	Filtered	Filtered	Filtered	Filtered	Filtered	Filtered	Filtered	Filtered	Filtered	Filtered	Filtered
Filtered	Filtered	Filtered	Filtered	Filtered	Filtered	Filtered	Filtered	Filtered	Filtered	Filtered	Filtered
Filtered	1909.377075	2456.658203	6883.255859	Filtered	1899.108887	2869.121094	3982.424072	5503.671875	1520.635132	1749.953003	1461.717285
Filtered	2778.876221	4652.616211	4484.053711	3153.977051	4640.575684	2456.90332	Filtered	Filtered	2794.148682	2530.550293	Filtered
Filtered	Filtered	Filtered	Filtered	2872.901855	2158.365967	2714.001953	4310.116699	3998.366943	Filtered	3559.619141	Filtered
Filtered	4455.774414	2948.762207	4350.595215	1983.959473	Filtered	Filtered	2101.897705	3369.357666	2406.607422	2852.064697	2871.931396
Filtered	2983.515381	3272.356445	Filtered	4051.293701	Filtered	3680.155029	1494.749146	2757.753662	Filtered	Filtered	Filtered
3145.370605	4955.495605	2994.99292	4358.045898	4113.337402	4709.933594	4496.828125	3113.660156	3065.67334	3622.086426	2746.48584	2779.856201
Filtered	Filtered	Filtered	Filtered	Filtered	655.814209	Filtered	Filtered	1082.567993	Filtered	Filtered	Filtered
Filtered	Filtered	Filtered	Filtered	4562.106445	Filtered	Filtered	Filtered	Filtered	Filtered	Filtered	Filtered
3289.980713	3995.939941	3008.238037	Filtered	3531.134521	1004.271973	2969.981201	3667.308838	2672.615479	Filtered	3176.026367	2224.781006
Filtered	Filtered	1964.908813	Filtered	4892.442383	Filtered	2454.043701	Filtered	Filtered	Filtered	Filtered	Filtered
Filtered	Filtered	Filtered	Filtered	2763.857666	Filtered	Filtered	Filtered	Filtered	Filtered	Filtered	Filtered
Filtered	Filtered	Filtered	1261.750122	Filtered	Filtered	3826.22998	Filtered	Filtered	Filtered	4058.539307	Filtered
2469.29126	3357.9104	2942.939453	2830.38916	2652.210449	Filtered	3364.184326	3125.467529	2327.435547	Filtered	4527.229004	3921.781982
Filtered	Filtered	Filtered	Filtered	Filtered	Filtered	Filtered	Filtered	Filtered	Filtered	Filtered	Filtered
Filtered	Filtered	Filtered	Filtered	Filtered	Filtered	Filtered	Filtered	Filtered	Filtered	Filtered	Filtered
Filtered	Filtered	Filtered	Filtered	Filtered	Filtered	Filtered	Filtered	Filtered	Filtered	Filtered	Filtered
Filtered	Filtered	Filtered	Filtered	Filtered	Filtered	Filtered	Filtered	Filtered	Filtered	Filtered	Filtered
Filtered	Filtered	Filtered	1632.973022	Filtered	Filtered	Filtered	Filtered	Filtered	Filtered	Filtered	Filtered
Filtered	Filtered	Filtered	Filtered	Filtered	Filtered	Filtered	Filtered	Filtered	Filtered	Filtered	Filtered
2795.601074	Filtered	Filtered	3476.409912	1216.564941	2951.657471	2635.697266	Filtered	2196.891113	Filtered	Filtered	Filtered
Filtered	Filtered	Filtered	Filtered	Filtered	Filtered	Filtered	Filtered	Filtered	Filtered	Filtered	Filtered
Filtered	Filtered	Filtered	Filtered	2268.345703	Filtered	Filtered	Filtered	Filtered	Filtered	Filtered	Filtered
Filtered	Filtered	3910.506836	2749.010986	Filtered	Filtered	Filtered	Filtered	3078.741211	Filtered	Filtered	Filtered
Filtered	3407.912354	Filtered	2632.523438	Filtered	Filtered	Filtered	Filtered	2956.766113	2341.639648	1555.46167	3516.131836
Filtered	1434.011963	1885.534424	Filtered	1425.010376	Filtered	3060.132568	Filtered	10713.22949	Filtered	Filtered	1533.016602
Filtered	Filtered	Filtered	Filtered	1136.726807	Filtered	Filtered	Filtered	Filtered	Filtered	Filtered	Filtered
Filtered	Filtered	Filtered	2122.953613	Filtered	Filtered	Filtered	Filtered	Filtered	Filtered	Filtered	Filtered
Filtered	Filtered	Filtered	Filtered	Filtered	Filtered	Filtered	Filtered	Filtered	Filtered	Filtered	Filtered
Filtered	Filtered	Filtered	Filtered	2780.383057	Filtered	Filtered	Filtered	Filtered	Filtered	Filtered	Filtered
Filtered	Filtered	Filtered	Filtered	2562.918213	Filtered	Filtered	Filtered	Filtered	Filtered	777.9506836	Filtered
Filtered	Filtered	Filtered	Filtered	Filtered	891.8214111	Filtered	Filtered	Filtered	Filtered	Filtered	Filtered
Filtered	Filtered	Filtered	Filtered	Filtered	Filtered	Filtered	Filtered	Filtered	Filtered	Filtered	Filtered

Con_20	Con_21	Con_22	Con_23	Con_24	Con_25	Con_26	Con_27	Con_28	Con_29	Con_30	Con_31
63834908	71078544	67057952	81370288	68818360	66623412	99347568	67493464	222066000	182078448	200156480	111471520
108872376	104325208	106196400	77468880	91212856	92724920	89284456	104709504	82099152	119558200	96190728	100966880
27031638	33948552	32908206	34279396	31425744	32786184	26138536	32608100	26909238	31053472	24099576	28309038
30694294	27114380	27689168	31360432	30188710	21750702	20328298	28875292	16479433	19419948	20378514	29505026
22161362	20128524	17633400	17184802	24542516	24496192	17563822	20039266	13324271	16925566	14444476	19340156
13997598	17992394	21500312	20221194	9172632	19580488	16812190	20428606	13489257	21250800	17518284	18296500
12107033	16984778	18085774	15661062	19244664	20339060	14294840	16703180	13047455	17636424	15950701	17539072
19033092	16125715	16758023	13498376	15269540	13810858	13081746	14655148	11705761	16965866	14886619	17118376
11936757	14551651	14703844	13903115	15935530	21275210	14350545	12172781	10782581	12913007	11879805	12629171
14049484	15209864	12739359	14359925	14238304	14035698	12158098	9800375	10218106	13446733	10136072	11442868
8237274	10905692	10715062	8163560	13174778	20068008	10311172	10869107	9079294	9131039	8751480	9741203
11872105	13095604	9893393	5361845.5	8403383	1476380.75	12286159	14006371	6169131	11591404	8400836	11581746
10246290	12156826	11834822	10547558	10440411	10958806	9408530	10540126	7468237.5	9111957	8199004	8115146
9393316	9578196	9707971	8422893	11117069	13375489	7163100	8927523	6535195	9457787	8412630	8454488
6288077	9215594	9197722	14661064	10688523	5774465.5	6865222	7647202.5	7114225	10615829	7268411	6974814
8964358	8843332	9566982	8216383.5	6890219	6201546	5556427	9946549	9504853	7238554.5	7766568.5	9144384
9245549	9493018	9169155	9001186	8839666	7532313.5	8910557	9616415	7331462.5	8683519	6757519.5	9789580
4893618	7587189	8153992	8531325	10013225	9977240	6346835	6854903	5631439.5	8085045	6532942	6759041.5
8782803	8897751	9468456	8226671.5	10783253	13307322	6640650	7848961	5015394	7744772.5	7508460	7348459
27375.41211	39514.89063	12694030	29210.12891	11232769	9867021	17623244	11260707	98653.375	106148.1797	121994.7656	63225.25391
8713730	6965407	6215771	6344768	7883698.5	10114739	5827851.5	7734505	8139203	5395727	5583392.5	6895640.5
7386390	5803348	6912866.5	6915163	8279871.5	7407906.5	6306429.5	6645940.5	5283328	6241781.5	5665912.5	7020092.5
4844960	8007629	7704570	14738754	7740152	2923990.5	5611417.5	6151983.5	5959723.5	9006878	6896568	5885593.5
5375627	11388186	5324849.5	5501819	6486323	6887655	7559359	5303806	7294681	6307295.5	5015846.5	5665913
5431589	7023290.5	7231824	5764889	7594292.5	7034258	6140084.5	6882130	5879694.5	7168701	5844084	5958827
6158284.5	7582415	6288616.5	5429070	7036111.5	8799323	6036614.5	6571891	5588285	7365110.5	6198833.5	5748165.5
6134365	5879483.5	7230420.5	7326661.5	6022733	5171690	4942192	6318472	4347629	5793388	5377894.5	6322731
5184555	6697619.5	6491750.5	5816349	5634780	5656403	4754917	6740707	4649587.5	5735528.5	4395540.5	5085130.5
3714845.75	5542635.5	5215128	4353014	5756569.5	4642681	4593733.5	4606294	3440414.75	4624901	4096638	4537856
5619772	4887409.5	8302537.5	3555628.25	5233026.5	2717360.25	5816832	8099534	3298835.75	4434169.5	3720047.75	5343601
4794538.5	5115336	4701298	4075463	4798242.5	3363556.5	3970027.5	4836788.5	4148488.25	5457300.5	4419567	5348693.5
6098964	4075880	5867738.5	4466308.5	5247492	4842513.5	4527565	5127605	4169691.5	4234535.5	3858865.75	3881696
5235576	5269552.5	4746358	4663079	4683633.5	4830207.5	4416264.5	5698053	3689771.75	4601815	3812597.5	4485239
4595910.5	4190903.5	4257220	2898671.25	4403004	6752905.5	3094559.5	5136181	3833757.5	3950192	4726978.5	3524272.5
5415851	4036712.75	5064006	2326419	5459191.5	3275791	2185793.25	4024164.75	3379176.5	1758935.75	3404782.25	4382622
1233308.875	2165838.25	1759541.625	2590806.75	2265004.5	2067519.375	2878290.75	2240866.5	7180733	6638080.5	5868222	2295794.5
3076670.75	3899898.5	4922564	4405505	4090947	2299075	2904690.5	4496954	2229008.75	2951004.5	3218190.25	2869891.25
3511862	3111767	2593991.25	3523915.5	3853448	4604944.5	4144514.25	3318563.25	3466014	3760248	2780956	4326799
3825922.25	3694191.75	3716390	3514675.5	3773466.75	3715525.75	2962402.75	4109798.25	2981800	4076598	3444599.5	3985707.75
9973849	2455.099609	Filtered	Filtered	Filtered	10532.04395	23284.46875	Filtered	9390.988281	Filtered	12680642	30956738
2484071.75	3900277.5	3699914.75	2252841.5	3431723.5	5073558	3865986.75	1417517.375	2776913.25	3473977	3429630	2877562.5
3809093.25	3051654.75	2902374.25	3118640.25	3192446	2977194.75	2611695.5	3583025.75	2535912.25	3492251.75	2418935.25	3824125
3933622.5	3358273.75	3260670.5	3174593	3714328	2798424.5	3116397.75	3196717.25	2722099	2870807.25	2870162.75	2718542.5
2744170	1211386.875	4532277.5	1681078.5	4591116.5	1079778.5	3600805.25	4510028	3381499	4209286.5	859863.125	3741939
2854336.25	3328614.75	3350432.25	3685824	3216644.25	3094358.5	2905528.25	3108861.5	2769758.5	2616231.25	2344778.5	2803408.75
2983528.75	3156481	3629001.75	2044961.75	2852961.5	1866682.5	3103052.25	3480127.75	2183760.5	2502172.75	2247824.5	3240510.5
3011191.5	3077584.75	3132161.25	2412753.5	3012780.5	2044302.75	3011688.5	2903990.5	2113806.75	2212631.75	2002103.75	2498248.75
10456543	1524822.375	1761188.375	5498646	1420308.875	18199968	1698371.25	1260000.25	4251228.5	2081530	2479990.5	1850357.125
3396777	3015317	2536136.25	3366293.75	3100391.25	2571494.75	1431930.125	4117593.75	2111041.5	2626008	2315338.25	2387126.5
2153132.5	2657712.75	2065150.125	3775046	2396326.25	3970847.5	1991289.75	2570229.75	2052257.125	3438341	2562661	2540846.5
2654490.5	3074503.25	2628747	2518017.5	2406047.5	3164704.25	2340449.25	2815095.5	2261578.5	2380128	2319185.5	2269359.5
2515854.5	2513794.5	2467001.75	2074583.375	2275587.5	3354815.5	1803720.625	2373547.5	2308675.75	2397917.5	2184957.75	2300173.5
2471796.75	2536516.75	2908388.25	2206866.5	2189083.75	2005289.5	1616461.75	1832317.125	1933206.125	1961643.875	1552597	2557513.5
1419904.75	1532772.125	1488562.125	1837817.75	1767803.75	1475793.25	3183921.5	1474727.875	6695042	3650385.5	3370883.25	2567300.25
2124529.25	2514289.5	2660308.75	1716070.375	2766136.5	1928667.5	1894257	1874288.75	1904099	2064072.625	1712651.25	1937159.125
2309410	1882059.875	2335311.5	2029108.75	2535060	2014328.25	2122214.25	2194567	1054611.75	1933699.5	2161090.5	2463609.75
2100430	2107839.25	2126056.75	2231609.75	2294115	2334490	1915265.375	1909237.5	1571283.125	1941000.25	1683546.75	1789419.875
1792649.5	2036240.875	2269497	2016663.875	2536689	2507675.25	1813129.125	2039956.625	1493199.875	1921762.25	1719103.25	1880493
2667755.5	2070661.75	1848852.5	1753606.5	2188823.5	1879498.125	1739769.625	2216421.5	1372167.625	1742401.875	1844466.375	1912828.75
1716937.5	2015537.75	1920308.5	1924628.25	2448246.25	3513877.75	1892887.875	1612014	1415300.875	1043474.813	1669271.125	1704867.625
2118572.5	2010608.5	1810140.25	1635426.875	2064946	3017023.75	1716933.5	1887535.625	2150983.25	1569392.25	1558654	2238683.25
1467188.5	1772715.875	1483577.5	814495.25	1350805.375	1319010.75	1446914.875	1420249.375	6005818	6050518	2280762.75	1542059.125
1556443.5	2336722	2107975	1806939.625	2175924.5	1802726.125	1881831.875	2081116.5	1568471.375	1648300	1700814.375	1505641.625
1207849	1082064	685090.4375	1469755.25	2170412.75	2041597.25	2360515	981859.125	7607177	4021540.75	3187873.25	1545864.875
Filtered	Filtered	Filtered	Filtered	Filtered	178825.0938	Filtered	Filtered	Filtered	2247161.25	3480811.5	Filtered
1361664.5	3283116	2612087	2511801.5	2404289.75	1284905.75	2192137.25	1760036	1574299	2363208.75	2413742.75	2074934.625
1732536.25	2240870.25	1742727.125	2654280.75	2077243	3316459.75	1896633.75	1528056.625	1302469	1314201.375	1624762.125	1496061.25
1579532.625	1894703.875	1675990.5	1602383.75	1697167.75	1519848.5	2234703.5	1638658.75	1801140.125	1492606.625	1587673.125	1501674.875
867057.5	2446806.25	2046095.875	1299519.75	2126227.5	1913738.75	2347664	2382307.5	1662748.875	1916900.25	1956020.25	2349893.5
1452934	1819383.875	1543344.125	1598873.125	1705166.5	3573036	1588961	1488414.75	1355109.25	1814688	1607053.5	1682608.625
1886466.75	2268797.75	2037408.75	713609.4375	1909224.25	2147782.5	1492397.25	1475917.375	1337198.5	1331969	1180536.25	1637843
1718186	935541	2326877.5	904711.875	2679385	496376.4688	858624.375	1442285.75	2043300.375	986351.5625	590721.25	1273016.5
1689326	1564507.5	1496526.25	1542660.25	1972322.5	2284024.25	1442598.75	1440796.625	1012925.688	1250320.125	1213971.875	1334500.625

1314873.375	1578957	1167503.25	2020960.125	1498353.125	1500106.625	936098.0625	1304624.375	1718027	1665595.875	1550035.75	1356097.25
860539.1875	436242.2188	821781.375	1113309.875	701661.8125	1201995.375	1434296.375	1134597.875	8522339	3333238.5	2879641.25	837935
1426362.75	1632626.75	1749459.25	1777024.75	1746760	1271436.625	1090409.875	1386021.25	1087396	1334786.625	1223156.875	1300056.75
1136337.125	2027803.25	1243722.25	2217351.75	1182693.25	1056949.5	1546053	1093863.5	717247.75	1657844.875	844811.8125	2360677
1364487	2132417.25	1717135	1477397	1644557	1559440	1352054.875	1992623.5	1178611.375	1299324.375	1330012.375	1282447.625
Filtered	1375465.875	1260967.25	Filtered	1334203.375	876971.6875	1824685.875	1227569.25	4194869	Filtered	2420487	1450485.625
1207088.25	1735656.5	1524399.5	1362558.375	1538694.125	1286172	1380948.625	1651118	1186176	1175097.125	1200387	1119386.875
1553921	1230850.875	2214142.5	766501.25	1444554.25	741085.5625	1513137	2254620.25	879160.125	1318543.5	1098422.875	1380893.25
1596253.875	1391784.625	1750029.5	1353092.875	49408.62891	1634851.625	68586.82031	1753101.5	214660.3438	1481865.5	1267632.625	60870.6875
1276858.125	1582112.25	1531635.25	1613444.125	1797232.875	1612003.75	1211530.75	991249.3125	800824.125	1357039.75	1070827.75	1337937.625
1488188.75	942491.8125	1723568.25	701970	2265700.25	948510.5625	632948.6875	1741745.5	686742.25	1248018.375	1664125.375	1337252.25
679290.75	724066.75	654207.625	933259.6875	928392.75	685994.5625	1262635.5	539099.75	4416761.5	2326259.75	2683722.25	1316580.25
1309916	1317473.125	1335326.75	1211587.25	1261094.75	926089.5	1316043.25	1297831.75	1006749.313	1158606.25	1030975.813	1230554.875
1603654.25	1488200.5	1137601.25	1266345.875	1407343.125	1358768.5	1660092	1136013.875	805241.5625	1183167.125	801571.5625	644463
1264338	1179814.75	1076758.5	925051.875	1307984.875	1366918.875	872202.0625	1130863	733033.25	1094642.5	1017992.625	1208206.375
487676	1111519.125	1086608	1095724.625	1219148.75	1241867.625	981530.6875	1016382.688	843377.3125	1331294	1062141.375	1175689.25
990539.1875	1209840.5	1324943	1324027.625	1131228	1066932.625	1128042.25	1304658.125	796804.4375	962254.3125	780585.3125	994189.25
1156724.5	963011.1875	1381349.25	1269528.625	1420248.625	1374144.5	1062495	953447.5625	813238.375	1073669.25	607643.5	854373.125
2100687	258564.7813	1355454.125	615192.9375	765589.5625	533920.4375	584463.25	183286.7344	421358.0938	553640.0625	955094.125	1016428.188
901089.125	1452077	1272006.875	465427	1559084	1303474.125	1132070.5	1396756.375	909078.625	973666.75	893172.0625	939488.8125
1091901.75	1397680	1269143.5	1292153.75	1410593.25	1438686.375	100420.5625	60242.28906	Filtered	4066681.75	4164178	1044333.875
1234322.625	1166919.75	1238102	1169261.875	1276465	864783.75	914298.375	1010668.188	823882.6875	921978.3125	895768.0625	1151376.375
182648.875	72803.60156	85684.21094	110514.6563	106932.9297	62863.23047	156871.0313	146561.6563	565797.625	192836.6094	269929.2813	268850.75
1118266.875	1586400.375	865622.8125	636769.0625	1102313	1872259.375	1318393.375	979107.3125	722562.375	1116876.25	856429.5625	742787.625
1099444.875	874100.25	1065160.25	1000848.813	1071200	1112270.125	799369.5	964405.875	779118	1192082.875	744476.125	933860.75
923329.875	823963.5	862097.875	909434.25	1097283.125	1256742.25	727995.5625	897175.625	695789.8125	998450.5625	725353	820800.75
401716.3438	424712.9063	418483.7188	564134.4375	522683	612507.0625	682010.3125	516411.6563	1729114	1436998.875	1037626.875	679902.25
664278	748370.3125	570814.875	355371.8125	639548.0625	2286038.5	556942.1875	559631.1875	607263.5	2080429.625	886732.25	905836.125
975281.75	1010254.938	885099.4375	322753.375	892590.5625	1416412.875	808649.5625	932376.5	701974.6875	808221.625	680334.9375	774845.75
633340	501767.9063	359779.625	634306.625	432750.9688	383127.3438	1026262	368709.625	2864251	2024778.5	1568319.75	945547.375
782026.5625	785699.25	1402569	702636.5	815912.0625	452790.1563	703906	782757.9375	667442.75	635836.375	581926.0625	726104.25
817930.75	911134	625674.625	896172.375	1140454.125	565201.8125	1158650.625	757465.8125	455727.1563	712681.25	592579.4375	738689
482067.3125	Filtered	Filtered	Filtered	909121.8125	394960.2813	Filtered	1021640.25	1007569	Filtered	Filtered	Filtered
585559.3125	262174.7813	242933.4063	371257.5313	143180.8594	178376.6875	563738.9375	414025.7813	893112	314930.9688	224108.1406	378123.1563
580516.75	820704.5	714910.75	723370.5	744826.5	687991.9375	923812.1875	671864.3125	493430	558444.9375	583023.5625	541406.6875
670099.3125	974546.1875	786338.75	339441.0938	958194.125	778495.75	777796.25	1080668.875	530575.3125	619625.375	586352.4375	612822.25
675734.1875	730331	720521.5	663670.0625	678925.9375	732151.3125	479671.4688	774217.125	455467.6563	550580.6875	456381.4375	508811.7188
99927.3125	42448.65234	37900.71484	56288.05469	54843.44922	37637.42578	97939.10156	82083.02344	272133.3125	115366.4609	162533	146455.9219
614796.625	588034.875	629429.4375	597063.1875	751122.625	881697.375	512417.625	577006.625	436993.25	681053.375	492477.6875	576518.125
735917.8125	753415.9375	655324.375	837698.0625	778731.6875	624833.5	491608.1563	630855.5625	Filtered	403392.75	449761.75	733198
541692.125	536958.375	775805.3125	464672.875	659135.375	712995.375	450332.0938	492809.25	526041	505957.5938	473154.25	516910.4688
553031.5	540263.0625	612100.6875	538414.5	654379.25	635770.0625	547891.4375	574549.5625	393631.125	475106.1875	438458.875	464365.2813
667834.3125	727301.625	745281.375	646741.75	655989.75	351362.1563	550710.25	573065.5	397392.7188	496005.1563	519735.125	543616.625
249431.2969	341244.75	224144.2969	279377.2188	339641.1563	251730.2031	785005.9375	398958.7813	1842315.625	625477	1068133.125	475613.25
410091.1875	695501.3125	104873.6953	676210.625	829810.4375	308398.0625	761121.375	598337.9375	1395219.625	1120155.5	920667.9375	710342
674765.3125	554459.875	780621.3125	439765.5	709430.6875	437568.375	526089.8125	593309.875	408552.1875	439296.6563	454382.4688	549259.0625
136059.2813	331700.875	289708.5	327948.25	397383.1875	231734.2031	668534.125	543031.875	1379959.75	546616.5	918428.9375	221370.4063
368711.125	244241.4219	296958.7188	231449.9531	404764.5625	228238.9063	542651.4375	241310.8281	2370585	884032.1875	1125631.875	651022.1875
600974.875	504779.9375	587730.625	631031.875	585747	517471.9375	483376.0313	662100.75	479980.3438	435002.625	398024.9063	443965.9375
266393.5938	362608.4688	356734.25	273054.6875	168669.5625	253848.0313	572659.5	547513.75	1272632.75	911753.6875	864197.9375	618918.8125
464620.3438	488336.0313	540222.625	625431.125	633164.3125	502976.0625	478953.9688	526983.9375	287398.9063	391196.6875	337698.6875	345792.1563
541087.375	443439.1563	442114.9375	433063.4688	437038.3125	363265.25	369630	408375.625	441931.5313	368340.3438	340947.6875	416825.0625
452882.7813	386721.8438	715685.1875	470936.2188	466897.9688	527982.6875	505812.125	443296.8438	442377.8438	332166.9375	323580.7813	401187.1875
546159.0625	468707.125	641668.25	482663.2188	362661.3125	337268.6563	451716.6875	582887.25	315186.1875	411986.6563	291000.7188	560012.4375
50475.18359	35138.13672	65318.39844	321001.625	54440.44531	661754.1875	449057.3125	563683.0625	42243.51172	910664.9375	823923.5	591781.8125
343717.8125	422663.9688	557308.1875	339493.9688	673327.125	325036.5	268459.3125	5680.974121	476575.75	663564.25	340509.5313	424902.125
325109.6563	378124.2188	313991.1563	137614.9531	321292.3438	270512.3125	333068.75	302180.875	349102.7813	344346.5313	308535.4063	332831.125
426326.5	455086.3438	463156.4375	466953.6875	503226.4688	459084.9688	435898.9688	446830.7188	320874.5938	383858	312356.8438	323968.625
349484.3438	476174.4375	378822.0625	461768.2188	442351.8125	472554.9375	434895.1875	354403.4063	335248.5313	431756.5	313628.5625	302662.0938
406179.4375	1231692.125	320230.4375	520446.4375	9624.551758	855050.8125	478159.7188	12910.79395	232656.3281	452672.6875	991786.1875	331935.0625
324768.7188	370389.9375	371965.4063	207113.7656	391064.9375	218592.5313	581892.375	316583.5313	417487.3125	594409.8125	257979.5781	241885.4375
528506.3125	451186.4063	441370.1875	579205	760530	366201.6875	502121.9375	536428.25	408741	489570.0938	542410.125	434205.2188
257923.6875	158884.3594	141823.0313	212219.7656	233348.9531	257099.125	300310.4688	158296.5938	197993.875	139968.625	2835639.75	165128.5313
383568.625	442610.4063	366167.875	305495.2188	401537.75	337880.5	389570.2813	451318.4375	332288.8438	352049.4375	358096.875	430542.1875
Filtered	Filtered	Filtered	1705953.625	Filtered	Filtered	Filtered	Filtered	Filtered	Filtered	Filtered	Filtered
440893.2813	440738.6875	469638.4688	347334.125	428723.4688	322341	335416.8438	326051.375	254942.875	262127.0469	181054.2188	395550.5938
371518.9375	444111.0938	314064.4063	281256.3438	383579.3125	659488.375	415876.8125	398689.9063	247028.1563	346876.2188	339658.5313	335089.8125
211487.25	184889.0625	242369.9531	215203.4688	117187.5625	141229.9688	276870.6563	245517.9375	261180.4688	263204	305221.0625	317369.75
188348.4531	193394.125	215169.5938	198336.9219	554332.375	465741.9375	232447.5156	160856.8594	129031.6016	175349.2813	129438.9297	158313.0313
368394.5625	336448.4375	350064.3438	341346.8125	553956.9375	365001.6563	242778.1563	378982.3125	237570.4063	344865.8438	300476.4375	395624.5625
261753.9063	428623.8125	371723.3125	308115.5938	350879.9688	222855.2031	379497.6563	347064.8438	181094.7344	325295.0938	256547.3438	216577.3281
426414.0938	321323.6563	309294.25	381432.5313	330794.4063	290241.3125	246571.0469	334590.4688	202609.75	262300.4375	294822.1563	291074.875
303529.3125	373601.75	338521.125	330003.9063	375818.4063	287767.0625	316361.5	347952.5625	235448.7031	228147.8281	243229.2813	333421.4063
Filtered	Filtered	509029.5	Filtered	469496.6875	Filtered	Filtered	Filtered	Filtered	Filtered	Filtered	Filtered
307260.5	357977.875	351321.3438	391909.4375	434342.4688	415148.4375	314798.5	212926.6406	173925.0313	341488.7188	290038.9063	314383.1563

507170.9063	163651.2969	138331.2969	343970.6875	43574.62109	74654.58594	110077.8984	54171.74609	168638.25	129272.5234	303901.7188	266949.3125
Filtered	Filtered	473144.7813	Filtered	165982.4219	Filtered	Filtered	103562.9688	299186.4688	307262.625	Filtered	422809.5625
358437.9063	314247.2813	405752.4688	311101.0938	306034.25	324797.9688	350128.8438	262673.875	220072.75	261365.3125	226893.7344	260933.6875
292548.875	234265.7344	255674.0469	232960.7344	424354.5313	277963.875	352337.8438	234211.1094	900217.375	441602.8438	390655.1875	240250.0781
164805.9531	111089.1797	861980	92376.79688	174606.8594	210764.2031	72970.49219	68451.04688	315210.8125	298913.0625	1449790.75	891596.3125
Filtered	73255.13281	31923.4668	38912.07031	25162.5332	91348.32813	Filtered	5449.627441	419385.0313	316406.3125	273966.375	120778.5625
173722.4688	230898.8906	143320.125	205998.5313	260898.375	229445.6563	535866.5625	217558.9531	1149542.75	439415.125	474130.5	328264.5
22266.28906	Filtered	Filtered	Filtered	Filtered	Filtered	Filtered	2085.407959	Filtered	Filtered	Filtered	Filtered
Filtered	Filtered	Filtered	Filtered	Filtered	Filtered	413642.6563	729564.625	Filtered	Filtered	Filtered	559644.75
268033.2813	271835.625	275237.75	269366.4375	229855.8906	280500.25	234354.6875	300842.75	217428.7813	239343.0625	236180.2656	248437.9219
213900.7344	308886.4688	306165.5313	213278.7813	261347.8906	277758.3125	256158.75	171597.0781	190674.1719	212037.6094	259014.2344	241805.5938
139814.5156	362139.0938	261493.3594	285794.7188	280243.375	279453	131814.9844	252614.8125	256992.6875	360004	397558.2813	296463.4375
260983.3281	324585.625	292071.875	157581.9531	256451.9688	359015.3125	125868.5625	289788.375	217614.2969	227299.9531	238729.4375	126176.2344
Filtered	257180.0625	164135.4844	279881.5	289654.125	Filtered	231989.3594	Filtered	Filtered	255569.2344	347429.0313	236106.2969
129954.2734	334440.5938	292180.2188	101364.4141	135371.9844	116313.4375	231527.6563	175632.8906	273393.0625	180293.8906	236067.4375	129828.6797
173089.1406	228756.5938	142921.1563	205766.1094	260239.8281	229135.3906	538048.875	217508.5938	566254.4375	439390.2813	474130.5	328264.5
251000.75	260861.5469	305991	265824.1563	282503.3125	262331.8438	291065.4688	277519.2813	173378.2813	229754.9844	259209.375	197435.8438
52658.66797	93628.41406	640458.25	155587.5313	126115.6641	117545.7969	32440.10156	60165.27344	188816.125	228505.2031	1005888.813	455088.875
148730.5156	142981.2656	220418.8594	246912.8281	166269.5469	237391.5156	268709.7813	257743.0156	123261.0547	247298.5	201896.4531	289788.4375
188618.0469	252175.7031	296909.9688	224300.4219	256058.8594	207083.125	253590.4844	258898.3906	209186.6094	237796.1094	184099.6094	195260.7969
337963.7813	146128.1563	398174.9375	190244.9531	284753.2813	137249.0156	110830.1875	197867.9063	263883.375	145586.8125	155365.375	192207.5938
130601.0625	207845.1563	180171.9219	114767.5547	55270.26953	45905.12891	338221.9063	571466.9375	942747.4375	167914.7031	428594.125	83997.22656
247316.2344	319230.3438	226254.0313	209319.4688	139771.8281	112763.0781	212603.8438	182334.9219	245028.7969	253538.8438	227047.9844	287819.6875
158800.1875	295986.875	304563.625	239189.7344	243425.2344	279876.125	186879.2188	230431	212612.4063	237276.0313	216561.0625	237646.5
190655.7813	160217.375	185815.6875	215552.2656	175184.25	243412.0625	192527.25	207741.6719	589997.5	336081.4063	307107.125	175423.875
184050	Filtered	246034.9688	162916.875	Filtered	Filtered	Filtered	200917	160475.2969	190149.125	Filtered	310112.4375
340355.3125	190079.6875	154186.3125	163215.4375	256702.5781	238783.4844	374216.0938	154634.6719	187699.6875	121921.9766	140131.6094	141671.9844
264904.375	74970.02344	94701.46094	333999.375	151585.5156	106862.1563	97735	277825.8438	121454.625	151591	125846.2891	462311.3125
205102.5313	256773.7031	232926.3281	197159.1563	228450.1094	309532.8125	214213.1563	223528.5938	172214.875	195832.0469	203459.3125	195975.2344
310254.2813	240921.875	252096.8438	178599.2344	264266.7188	12427.07715	197442.4375	316331.9063	285339.7188	189353.3281	28777.60156	248564.0781
154737.25	166262.8906	182972.375	112408.2656	299606.2813	271615.75	1471838.75	178285.1719	111384.6094	126840.4141	111015.3203	156960.3906
124910.0469	115985.0938	90621.875	112615.5234	200975.2969	168099.3281	318035.9375	122422.1641	668127.4375	414647.1563	365225.375	267450.9375
253311.7188	241593.5781	220041.6875	286688.5625	235045.7969	164500.7344	224871.8438	245920.9063	136059.0625	169512.5313	175633.3281	207945.9375
226641.9063	251153.7031	198371.4688	105822.8906	206044.5313	255641.25	220402.0469	157229.8281	189700.9219	212123.1875	192246.0781	231399.4063
134406.7969	117461.2422	106042.6719	124606.6797	150769.7344	167412.1406	367526.75	128474.8047	301296.375	291157.4688	366385.25	274692.5
219830.8906	231477.5	224627.75	211026.3438	225661.4688	186280.8906	187039.5625	159288.6719	137054.9063	160749.3438	147609.2656	156300.0313
262265.3125	244919.4531	223540.1719	175637.8906	228694.7031	142137.1875	247525.3281	232457.875	177432.4063	229160.0938	187935.2031	197939.6406
Filtered	Filtered	Filtered	Filtered	Filtered	Filtered	Filtered	Filtered	Filtered	Filtered	Filtered	Filtered
97274.4375	143956.7344	86512.67188	124359.6172	128225.7969	112619.1563	231797	120987.6016	611189.5625	216683	313088.6563	206433.7344
184436.6563	213187.0625	211937.0625	178191.9531	209754.7969	193869.875	179404.9531	203447.2656	144688.4844	181213.2031	178325.0781	183522.75
127751.9375	210543.7188	340683.0625	202640.0313	204483.0469	93878.03906	224149.7656	279666.2188	178486.0938	198116.9531	99565.39063	157045.25
Filtered	Filtered	Filtered	Filtered	Filtered	Filtered	Filtered	Filtered	Filtered	Filtered	Filtered	Filtered
195521.3594	200910.0469	209242.25	151725.2031	179087.7031	158416.4844	183846.7031	176859.7656	142715.625	167026.5469	146266.1406	197245.8125
249001.4688	Filtered	Filtered	Filtered	Filtered	280186.9375	Filtered	Filtered	Filtered	Filtered	Filtered	Filtered
179395.1563	229145.7813	176501.8906	193768.1563	217121.7031	447348.8438	212012.9688	180377.7188	153442.6719	103361.9063	85081.77344	199696.9063
100304.5703	162796.6875	111385.375	204118.7969	160791.0156	87421.1875	Filtered	96390.1875	87479.73438	150399	76146.63281	203078.4531
196065.5781	169402.3594	158726	121771.7578	140907.6875	351343.3438	168534.5781	210440.875	129908.5	174269.2813	141002.5469	124142.6328
161007.9063	197539.9531	172997.4688	190540.6406	202607.5781	142336.8594	181519.4375	157255.3438	107295.1719	160658.6094	128061.6016	131401
144350.3125	190256.2344	146327.7188	172560.0156	263784.4375	173724.8438	130903.7109	133083.4688	125469.7656	142477.3281	93607.32813	145038.5781
82455.10938	56181.82422	428384.1563	45140.49609	87674.48438	106202.1953	35065.13281	33239.48828	159213.1094	148132.0938	727016.125	223237.9375
140155.6094	204165.7969	169486.8125	129230.0313	198814.3125	234167.9844	179969.5469	157752.5625	125231.4219	150019.1094	128669.8984	143193.8906
125758.6719	70846.45313	67346.15625	97396.92188	113939.1016	139004.875	191469.5	82532.9375	559111.9375	254660.2344	226460.1563	145810.4531
26410.59375	82113.23438	354345.5625	39639.11328	89743.65625	120434.25	40006.17578	37616.97266	141902.5938	144694.6406	575745.125	279581.7188
Filtered	Filtered	Filtered	Filtered	Filtered	5392.42627	Filtered	Filtered	Filtered	Filtered	Filtered	Filtered
204623.3125	116068.0313	139593.625	62005.38672	244331.9063	239147.4219	290399.5	99347.35156	80924.23438	312305.25	93334.39063	169269.2344
128628.4688	152550.375	146282.5	74034.11719	186740.8125	291486.125	192767.3281	171511.7188	117425.1172	134023	132372.6094	148874.0313
83295.5	Filtered	308124.125	Filtered	Filtered	145697.9844	42810.58984	353644.4688	Filtered	Filtered	Filtered	Filtered
69577.10156	90607.50781	19157.32813	31655.45703	114644.3672	126397.1797	229721.9844	Filtered	452421.1875	189128.3906	109070.0469	160607.2813
60243.89844	1066247.375	50180.53125	55604.30859	19572.06836	15932.78125	921398.1875	48193.6875	35603.85156	25726.36523	1624485.625	50723.36328
69805.91406	123337.5781	53325.07031	45460.01172	111766.3281	112280.2344	222327.4063	79347.28125	456086.0938	186349.375	264220.5	178317.6563
8.87E+04	1.87E+05	1.46E+05	1.84E+05	9.15E+04	1.42E+06	1.16E+05	2.35E+05	1.13E+05	1.51E+05	9.68E+04	1.08E+05
135799.1406	153559.4844	138493	125324.2266	243204.2656	148710.7969	108669.7813	132458.0625	86927.50781	115530.5703	96781.10938	143792.75
143217.0625	160969.8125	158993.2031	145896.6094	259698.5313	258788.4688	114140.3125	156345.125	211367.2969	90229.14844	119193.6797	339612.7188
76066.70313	84932.64063	25383.83008	32068.40039	83051.125	101312.4375	92125.8125	163644.4688	297140.5625	92188.07813	45200.6875	74134.90625
266989.6563	196716.375	189744.9688	28975.6582	262808.4375	232837.25	186486.1094	134193.7188	164992.6563	114112.7031	125250.0703	111403.5469
135808.75	101305.125	147020.0469	294991.2188	540754.875	337551.2813	153843.6563	63054.66797	130515.8438	178297.9688	120538.4531	138886.4531
271679	112266.8125	85218.78906	164952.0938	251689.7813	284467.0625	107522.2813	49978.54297	51100.57422	79552.42188	112892.2969	57708.33984
145360.4375	70804.39063	60148.0625	59536.39063	116316.5234	141587.3438	50893.08594	75319.91406	90333.59375	96796.89063	91174.6875	48227.78906
51611.05469	25202.41406	421524.6875	14231.48242	205792.375	12286.40918	25418.00586	31479.93164	40526.96875	38466.75781	22069.23242	27550.38281
54400.64063	Filtered	42077.99219	147720.9688	Filtered	Filtered	121732.8594	Filtered	Filtered	Filtered	Filtered	Filtered
117344.4609	106045.8828	119148.0781	179029.2969	100779.125	83260.21094	88360.59375	149324.7813	101425.3594	121301.5625	86441.28906	138081.9844
101521.5703	73203.94531	47407.83984	81043.60938	140217.3594	123080.0781	146090.4531	66785.30469	493818.3438	280457.625	196327.1719	100604.8047
102541.7578	92705.24219	81341.40625	111168.3516	162254.7656	194574.4531	194177.125	145403.1094	96473.55469	93540.05469	84979.98438	104537.3125
104971.5547	170263.2656	72645.875	73738.92969	109222.4141	124530.2969	165414.2031	130471.1406	7083.882324	137403.5625	113544.0547	86765.83594
Filtered	12030.16016	Filtered	896089.1875	4339.798828	Filtered	Filtered	Filtered	Filtered	Filtered	9459.119141	Filtered

143256.6406	115203.2188	111999.5391	128169.4141	111572.2578	154754.2031	91656.98438	133611.5469	134692.7813	99767.03906	102804.5469	88645.64844
121172.5938	166686.7813	135180.7188	101378.625	122658.9063	111930.7266	99650.15625	114170.7813	97527	126919.8594	92505.23438	132864.25
67160.67969	89160.46875	50095.87109	84620.72656	112715.8047	122163.0547	52721.94531	92949.85938	130979.6328	186943.0469	272987.5938	158396.6094
154586.625	124132.4141	123069.7109	151471.3125	139483.75	112414.1328	119637.5625	123958.1953	82022.71875	91750.4375	84426.95313	87553.03125
100104.5078	Filtered	132640.9844	Filtered	132797.5938	Filtered	106808.9063	Filtered	87802.27344	139301.5469	Filtered	111406.5
Filtered	Filtered	Filtered	Filtered	Filtered	Filtered	Filtered	Filtered	Filtered	Filtered	Filtered	Filtered
52354.30859	227621.8906	168874.625	22903.85156	153852.2188	154278.5781	173669.8438	180024.5781	157152.6719	127433.4766	131386.9844	123030.5703
92134.41406	69907.49219	64869.22656	82013.19531	82364.80469	142604.3906	140677.8438	122421.0703	44711.05469	55086.17969	42658.04688	57692.21094
104743.4141	140363.4531	110331.7422	131305.6406	123858.4141	90503.47656	115252.375	118403	86659.47656	86042.1875	93697.84375	113514.9063
Filtered	Filtered	2361.852051	Filtered	Filtered	Filtered	Filtered	Filtered	Filtered	Filtered	Filtered	Filtered
54746.42188	62648.20703	61400.44141	75697.13281	64327.15234	65440.51563	87032.78125	47822.5	182689.2813	166743.5781	175598.9219	102098.125
Filtered	Filtered	Filtered	Filtered	Filtered	Filtered	Filtered	Filtered	Filtered	Filtered	Filtered	Filtered
Filtered	Filtered	Filtered	Filtered	Filtered	Filtered	Filtered	Filtered	Filtered	Filtered	Filtered	Filtered
140834.625	41647.89844	48005.38672	55183.60938	58122.89844	47759.66797	56861.76172	49790.08594	40899.75	39316.30469	44448.51953	82679.90625
8857.511719	Filtered	11817.21191	5424.27002	118920.3672	Filtered	Filtered	Filtered	Filtered	69182.92188	Filtered	Filtered
Filtered	Filtered	Filtered	Filtered	Filtered	Filtered	Filtered	Filtered	Filtered	Filtered	Filtered	Filtered
131979.9375	107471.7813	101499.7891	108557.8047	109947.1406	93650.5	87535.05469	104689.5625	65194.90625	69926.92969	76660.96094	96235.39844
29933.02734	37483.30469	83082.3125	175275.9688	18538.38086	57662.61719	225627.4375	42662.86328	155464.7344	508374.4375	86443.09375	215768.125
Filtered	Filtered	20689.63086	13951.10156	37594.40625	8248.037109	63634.44531	66920.03125	496129.6563	303240.75	26924.66406	101334.3828
99088.8125	98095.01563	103149.9219	98278.39844	100135.125	77747.75	95977.74219	113684.75	75944.36719	81103.48438	73876.85938	89164.10938
72617.97656	98118.98438	89289.41406	100628	84001.01563	83343.77344	127127.2422	94230.90625	69388.73438	75047.48438	60383.39844	85568.89063
48373.8125	64258.19922	55039.48438	56005.62891	60690.90234	45755.73828	147190.125	69835.875	284488.375	158943.5313	171970.2188	113796.8516
35408.875	54722.0625	22238.85156	42507.39063	27438.75781	26090.64258	78864.72656	32876.39063	27443.70508	20099.23633	23438.3418	20325.33984
67332.5	65508.33984	110299.6016	72414.67188	83032.26563	122791.3516	51977.41406	80710.58594	75504.08594	80018.76563	66435.24219	55643.41797
107290.9453	49231.25	117638.9219	112866.3359	58711.14453	59307.46094	38873.01172	74637.17969	49999.08984	108835.1953	78968.39063	85201.60156
90932.46875	114811.0469	138297.9531	76710.65625	25738.86133	Filtered	88157.07031	85803.08594	62781.12109	77151.375	91576.0625	144444.2188
116703.6719	82432.69531	88766.25	104146.8516	88957.48438	108530.4219	55415.49609	127259.8125	72858.92188	89495.625	86416.89063	65241.44141
37583.19531	142361.3906	38355.86328	42768.46484	41558.22656	145202.7656	132529.9063	35778.33594	97894.39844	127254.3359	101885.1953	127081.0391
99550.88281	36587.34375	189544.2656	30287.31055	48551.01563	31108.64648	32067.06055	20980.78125	75968.71094	61545.12109	184641.6094	142930.2031
85756.625	60570.98438	74739.45313	51197.49219	71871.11719	46187.54688	52729.53906	94897.17969	59202.73828	72250.85938	54038.14844	83673.1875
Filtered	Filtered	Filtered	Filtered	Filtered	Filtered	Filtered	Filtered	Filtered	Filtered	Filtered	Filtered
66891.83594	79869.64063	65196.32813	27049.04688	68774.47656	55805.39063	79896.03125	64863.82422	70948.64844	67595.91406	64788.875	78457.42969
Filtered	Filtered	Filtered	Filtered	Filtered	Filtered	Filtered	Filtered	Filtered	Filtered	Filtered	Filtered
38551.57031	17783.3125	182872.0781	16462.12891	36273.71484	34564.49219	28075.1875	20376.98828	116779.875	75982.39844	165926.7188	144894.1563
14249.3418	35684.88672	44239.34375	127760.625	46371.27344	24014.75977	34107.73828	115956.8984	Filtered	Filtered	32521.02344	27058.55664
62547.57422	146036.9844	73111.42969	70124.29688	46516.63672	57388.27734	109965.7109	89278.48438	65456.25	118168.9453	124062.4688	111088.4766
91403.10156	80755.8125	80746.24219	47624.81641	87431.86719	87972.34375	62326.46875	64428.05078	74256.4375	77478.03906	45192.98047	80270.11719
Filtered	Filtered	Filtered	Filtered	Filtered	Filtered	Filtered	Filtered	Filtered	Filtered	Filtered	Filtered
40027.78906	28943.1875	20916.16406	27629.17969	49249.59375	104741.2344	46122.28516	26210.53125	155253.4375	76198.89063	72917.75	34885.23438
Filtered	Filtered	Filtered	Filtered	Filtered	Filtered	Filtered	Filtered	Filtered	Filtered	Filtered	Filtered
70829.60938	87368.17188	66717.75781	88683.75	73572.82031	33126.94531	76158.60938	50195.34375	74567.9375	75030.03125	49858.24609	71646.11719
164766.8125	96730.66406	74886.9375	41171.28516	53844.56641	168681.7969	71911.89844	44732.09766	28466.03516	68840.79688	34657.28906	38971.38281
30185.57813	26324.26953	27539.85547	43836.14063	42340.53125	19496.92773	109694.0781	46034.44141	224431.8281	73724.91406	141370.2188	55038.25781
Filtered	85947.04688	Filtered	Filtered	Filtered	Filtered	Filtered	51158.87109	65727.39844	56370.50781	Filtered	44562.70703
59947.05469	24982.22852	78392.41406	25560.70313	69528.14063	40418.4375	26139.60156	25976.35352	31790.50977	29501.10938	25366.12891	66206.86719
Filtered	Filtered	Filtered	Filtered	Filtered	Filtered	Filtered	Filtered	Filtered	Filtered	Filtered	Filtered
83112.29688	41835.41797	27085.60352	28492.10938	56809.69141	58970.39063	67716.60156	57282.80078	44793.44922	51184.64844	21991.04492	35462.41016
70916.82813	68413.26563	73747.40625	58872.64844	62338.1875	69845.64844	60739.12891	71981.65625	54678.71094	60285.19141	33823.26953	57701.62891
121300.2891	55321.58984	76567.89844	42464.06641	92046.375	6295.527344	6310.638672	215624.6563	Filtered	180496.0938	158892.2031	86294.67188
40746.15234	47248.05078	28067.30859	36263.80859	42318.07813	39991.62109	98395.9375	44784.95703	247008.0156	106289.6484	115452.6484	76631.91406
28559.86133	40374.45313	120146.6719	34809.55469	35051.4375	53615.67969	19299.48438	28479.16016	45674.92578	48321.26172	346915.2188	163101.2969
25221.87305	64967.22656	58045.33984	120939.7891	61320.53516	36093.67188	47392.23047	63387.97266	46147.36328	68980.23438	49994.0625	45319.70703
86869.5625	78008.35938	82249.71094	65147.67969	58972.78906	48827.24219	60956.63281	56538.29688	45128.17188	50033.89063	46124.12109	54312.49219
1337.747437	Filtered	7380.503418	Filtered	2955.639893	46364.98438	44735.25391	Filtered	50031.16016	Filtered	45830.37891	Filtered
165317.25	37914.32031	35262.10156	113298.1875	31572.63672	380630.5625	42208.41406	29638.47461	113067.9531	51522.27344	54511.65625	31869.95117
49654.02344	61410.4375	74967.125	133906.6563	55168.19531	27568.87695	59894.51563	58526.42969	29082.16992	24505.125	60392.39063	38778.91797
Filtered	Filtered	Filtered	Filtered	Filtered	Filtered	Filtered	Filtered	Filtered	Filtered	Filtered	Filtered
48036.17188	104437.875	64849.70313	63939.05469	61446.5625	19159.39063	94601.25781	95421.10156	49682.20703	62014.05859	62602.16016	60789.32031
Filtered	Filtered	Filtered	Filtered	Filtered	Filtered	59990.44141	Filtered	Filtered	Filtered	Filtered	Filtered
35635.44922	39624.67578	45084.07813	41267.28906	66336.83594	55397.28906	63246.64063	24278.32813	155364.875	129589.7578	119809.7813	66322.8125
76467.49219	50694.50391	55562.32031	42182.64453	52857.28906	50684.37891	62572.39063	80416.3125	48660.55859	78837.07813	48678.14844	64378.53125
33755.10156	37688.88672	44784.62891	89361.13281	130137.6953	104990.3984	83675.49219	34295.33594	50341.45313	76876.42188	25893.69531	30725.06445
30523.88086	30133.58008	26982.08984	Filtered	48365.37109	39243.57031	55997.63281	26667.25195	162173.2813	58232.53516	79761.64063	69865.53906
Filtered	Filtered	Filtered	Filtered	Filtered	Filtered	Filtered	Filtered	Filtered	Filtered	Filtered	Filtered
22787.29688	60149.17578	54493.09375	Filtered	65315.46875	60554.17969	46844.86719	65652.65625	35038.39844	65727.82813	49415.39453	116304.8125
62304.93359	61181.30078	66379.4375	76742.25	61925.51172	52916.03125	71896.28125	53788.40234	34769.25	48352.75781	39045.55078	62277.19141
56010.48438	59935.91406	72556.96094	26871.82227	70759.39844	62403.16016	44996.35547	48225.96094	23912.05469	58310.99219	46739.63672	42478.94531
33007.38672	54953.52344	64696.98438	42466.26953	46686.23438	44774.17188	48317.25	47926.83203	37353.69141	61196.22656	56877.69922	131004.3828
36574.34766	39247.22266	36854.66406	34342.57813	38528.61719	73129.50781	64952.49609	52924.17188	44469.21875	78190.3125	66746.67188	82718.58594
9622.629883	17269.19141	Filtered	19780.02148	15908.64746	Filtered	39859.85156	25254.30469	91414.01563	27800.65234	61141.11328	Filtered
53029.07031	76712.03906	72585.26563	23190.42383	45048.87891	62186.04688	55037.35938	54836.94531	60563.96094	40856.55469	31416.87891	70832.48438
60020.07422	56314.48828	66271.89063	62748.84375	41667.78125	36834.30078	49964.83203	48024.86328	27208.58008	46414.75391	35913.46094	52084.79688
53274.61328	55852.03125	64135.07813	68921.63281	56889.10938	32764.21484	58969.67188	78283.55469	36364.5625	46023.5	38813.14063	59494.38672
38774.82813	55686.60156	47696.63672	45939.80859	82112.42969	61045.01953	45814.47266	30914.17773	28357.375	59984.02344	54664.80859	42552.30859
21312.26172	27357.79688	25192.19922	46236.53906	41242.61719	51853.80078	56264.37891	44068.07031	52956.87109	69867.24219	62896.30469	56878.15234

32547.98242	10387.67285	57975.07813	65824.21875	53310.40234	Filtered	40920.11328	46653.02734	49704.97266	60163.09375	35237.80078	43925.67188
24446.10352	18227.30469	25899.78125	34067.93359	31305.9668	16219.78613	94418.33594	45695.22266	154131.9844	48033.27344	140604.4531	48720.125
30145.54102	57501.42578	103174.125	13295.77344	24723.58008	16878.90625	15849.24512	18728.71484	49904.61328	39468.19922	124539.7969	88729.14844
Filtered	Filtered	Filtered	Filtered	18982.18555	96266.38281	Filtered	97916.78906	59398.29688	Filtered	Filtered	81996.73438
55947.8125	48913.24609	49140.74219	44809.36719	62684.64063	69271.60938	50713.11328	37285.70703	Filtered	29832.48633	23602.08398	39714.08203
34860.67969	47459.82031	43678.39844	56358.29688	35609.07031	75509.96094	59407.40234	46851.73828	36325.51563	31498.25586	35401.69141	34268.48828
59101.02734	45900.8125	49921.76953	57218.75781	62774.00781	93359.80469	45252.72266	37308.26953	26674.08008	45441.66797	41515.59375	42370.50781
57036.70703	68012.60938	44184.17969	28024.06836	27064.77344	27393.34375	56099.60156	115491.8047	67470.10938	56624.42578	66503.96875	32895.20313
Filtered	Filtered	Filtered	89407.875	84965.8125	Filtered	Filtered	Filtered	Filtered	Filtered	75776.90625	76830.13281
50646.63281	53405.49609	43440.82422	91840.26563	37996.24609	58591.51953	95391.89063	29230.81445	42550.375	35462.40234	139702.5781	79953.05469
14992.62402	37126.50781	17211.84961	26046.14063	18713.24414	21553.76172	51809.91016	29068.92969	96992.5625	90736.82813	136784.2031	56222.47656
54350.11719	54238.54688	70562.96094	41194.38281	74922.76563	53517.19531	41613.04297	48551.27344	31914.85156	40624.00391	34952.375	49134.18359
54106.27344	96085.34375	102621.9219	39513.69141	133139.375	66397.00781	59613.64453	55367.85156	15223.75586	69051.5625	21433.31445	67880.99219
9231.647461	259334.0938	Filtered	Filtered	Filtered	Filtered	2386.103516	Filtered	Filtered	Filtered	Filtered	Filtered
59041.52734	23365.12109	47936.73438	29488.96094	50183.92188	70945.24219	29340.56641	34376.60156	44238.38281	27155.68945	18232.30273	29100.25
59714.85547	50654.40234	56118.83594	72632.35938	59849.71875	70840.89063	41344.5625	69322.88281	43217.06641	54204.18359	50486.92188	69184.92188
44294.99609	72509.23438	42611.71094	45563.16406	47307.25781	33713.16016	44533.30859	54187.79688	31297.91602	47192.94922	36150.15625	39459.85547
17609.05273	14857.8916	12456.74316	11241.77148	14809.12988	24416.37305	16087.55469	12620.52441	14912.04297	10804.44531	13702.91992	13532.2334
47349.09766	43268.65625	45835.95313	54560	42580.06641	78959.96875	39792.75781	40845.75	36015.91406	39241.89844	36508.47266	35582.09766
41862.8125	49488.78125	43604.51953	43314.53516	45461.375	49395.55469	37608.96484	34832.70313	43187.28906	36810.78516	50264.75	26488.71289
47812.35938	40257.46094	60218.44531	2723.000244	89350.33594	58697.875	129301.4688	14118.37988	34401.90625	29340.92578	21603.41797	20969.26367
Filtered	Filtered	Filtered	Filtered	Filtered	Filtered	74682.20313	Filtered	Filtered	87385.64844	Filtered	Filtered
27400.51953	31188.11328	20514.76758	21636.39258	20810.45703	17433.12305	38596.82031	30961.12305	125236.4688	61774.45313	100029.625	53638.61719
38556.09375	60427.17188	50285.41016	35586.33594	47982.00391	60242.31641	38956.85938	50685.625	30116.35352	41188.34766	38217.80078	41808.80469
6294.864258	2922.214844	3379.581055	2927.355225	4132.537109	7981.873535	Filtered	3668.249512	Filtered	2341.565186	Filtered	4093.225586
Filtered	Filtered	Filtered	Filtered	Filtered	Filtered	Filtered	Filtered	Filtered	Filtered	Filtered	Filtered
Filtered	3013.748291	2795.65625	Filtered	2933.786621	Filtered	2968.083984	1845.149414	Filtered	Filtered	Filtered	Filtered
35996.21484	39541.59375	44886.39844	40258.82422	66276.74219	55231.23438	63761.63672	23998.66992	60211.34375	129709.4531	120110.5625	66282.96094
Filtered	Filtered	4359.001465	Filtered	24323.06445	Filtered	5303.382324	Filtered	Filtered	Filtered	6710.736328	Filtered
1761.540039	Filtered	Filtered	18954.55273	Filtered	Filtered	Filtered	Filtered	Filtered	Filtered	Filtered	Filtered
Filtered	Filtered	Filtered	Filtered	Filtered	Filtered	Filtered	Filtered	Filtered	Filtered	Filtered	Filtered
16578.98633	22737.18164	23691.0918	19128.5332	28482.02539	11122.00195	24207.73828	23474.73047	17258.7207	14681.9707	16294.74316	17790.79102
39175.9375	9631.282227	30362.49023	32053.72266	26728.0918	16984.10547	40114.51172	25976.66211	146314.6094	56711.06641	57278.64844	33261.24219
46195.46484	55609.45313	30660.4043	36359.21484	35515.69141	37740.4375	41740.48047	33544.01172	29529.69531	33998.60156	35208.89844	45041.77734
41881.64063	44288.12109	38119.98828	56146.08984	39839.11719	47425.98438	35276.26172	43497.28125	30188.99023	38759.67578	30274.31836	37761.96094
55007.86328	27503.43945	34278.05859	30647.74414	38642.1875	71334.8125	Filtered	32241.13672	49640.70703	40595.54297	Filtered	30173.54297
35232.01563	25762.02148	37631.81641	42648.9375	46703.10938	44771.51953	31301.19141	45416.01172	56836.32422	42449.03906	30504.07227	36722.57422
32019.91406	66457.97656	44485.29297	35430.77344	50767.80078	54519.65625	50319.94141	54712.23828	33488.74219	32818.98438	30595.80469	39612.58203
17894.55078	Filtered	Filtered	53698.33594	13976.18848	9479.323242	14466.9209	12024.44531	13103.70117	11694.18359	16097.89648	13486.02539
56641.85156	31495.0918	28627.31055	31824.61133	36906.60547	37979.72266	64426.05078	30980.64453	31635.42578	27181.56641	22612.84766	22288.20703
51245.88281	27489.40039	27411.61523	72469.375	32866.60547	33627.83984	28086.38672	43880.72656	35490.11328	45823.10156	20258.6582	51450.68359
38274.42969	41527.76172	41305.02734	41876.63281	37163.52344	33997.03516	40608.38672	39164.76172	35382.51953	33249.62891	29426.97266	31877.75
40049.02344	36409.11719	40920.24219	29447.08984	43986.96484	37949.80469	34333.76953	31329.53906	24809.01563	30100.3125	28483.05859	34784.58203
30943.15625	34763.64063	41551.17188	37852.89063	44759.31641	41006.21875	31164.32422	23995.85938	22401.23828	55562.98828	23763.63281	29340.49023
Filtered	Filtered	10119.06934	Filtered	Filtered	Filtered	Filtered	Filtered	Filtered	Filtered	Filtered	Filtered
57532.52344	32607.81445	46227.74219	Filtered	52889.78125	27374.17383	Filtered	Filtered	26960.44336	Filtered	14189.50293	53683.55469
49037.82422	57936.875	72538.54688	105507.3672	66654.5	11327.68262	16952.95313	60643.35547	16989.05859	15841.44824	18113.42188	66135.96094
Filtered	Filtered	12292.41699	Filtered	Filtered	Filtered	Filtered	Filtered	Filtered	Filtered	6478.256836	4782.86377
Filtered	Filtered	Filtered	Filtered	Filtered	Filtered	Filtered	Filtered	Filtered	Filtered	8070.220703	Filtered
22165.21094	17401.12695	21809.8418	41146.96484	30516.71484	23976.59961	24644.91602	17840.93164	108531.1641	52907.87109	39164.79688	29158.42578
38545.40234	40359.87109	29150.75781	36346.40234	35247.81641	33726.87109	26488.05664	37897.64453	28289.11133	26700.66406	31824.74414	33123.69531
37326.33203	34588.93359	52946.92188	37874.46484	55114.66016	36068.08594	38983.78125	18816.74609	19835.17578	16788.91602	20500.7207	45032.55859
31701.30273	28007.54688	23756.11133	26425.72266	Filtered	27434.68359	19164.16406	40507.68359	36376.52344	31549.35742	81477.71875	28903.65625
24979.83008	25390.85156	23004.7207	14324.3252	35304.60156	22631.10742	25998.48242	28034.06445	6273.361328	21776.21875	19847.58203	31697.63477
39151.52734	57134.83984	26719.45898	10160.76465	30603.16211	132032.0625	13594.49805	32372.05664	Filtered	6263.615723	Filtered	9534.082031
Filtered	Filtered	Filtered	Filtered	Filtered	Filtered	Filtered	Filtered	Filtered	Filtered	Filtered	Filtered
Filtered	Filtered	Filtered	Filtered	Filtered	Filtered	Filtered	Filtered	Filtered	Filtered	Filtered	Filtered
13604.93945	30085.10352	55506.96094	14158.6084	12107.19531	9806.451172	10050.58398	19467.83984	22979.94922	29560.45508	92015.14063	43705.52734
Filtered	Filtered	Filtered	Filtered	Filtered	Filtered	25565.17383	4592.026367	35959.37891	Filtered	33471.14844	17627.14453
28346.21094	27731.59766	32032.85742	18740.39453	29967.51563	29560.02734	33214.35938	34556.5	27279.21289	25963.21484	28559.2168	31878.85742
22128.57031	7824.710449	64764.98828	4862.977539	22439.79688	9008.459961	6106.288086	13202.01563	49442.85938	45387.22266	50849.33594	50610.53125
Filtered	49341.66406	29773.12695	32952.07422	21258.56641	29170.44141	53290.86719	47852.28906	33512.46094	43380.16406	37502.03516	Filtered
51212.41797	13398.8252	18335.53516	16929.30078	16001.13477	13487.86914	19141.41211	14916.67578	16780.69141	17955.38086	20799.23047	24395.31641
Filtered	Filtered	Filtered	Filtered	Filtered	Filtered	18193.39063	Filtered	Filtered	Filtered	Filtered	Filtered
Filtered	Filtered	Filtered	1977.181763	2934.02832	Filtered	3301.235352	Filtered	14037.96777	5542.883301	6436.898438	8270.016602
2641.599854	Filtered	922.8887939	Filtered	3634.692383	Filtered	Filtered	4488.719727	Filtered	3419.463379	Filtered	3550.196533
35164.90234	23461.80078	26433.13672	25276.24023	30089.44141	24194.4375	24950.60352	25544.93555	19699.47656	26227.37891	27677.93555	22658.26367
15519.81055	25544.66406	25865.50195	25691.97266	41497.63281	41722.33203	27292.42969	21910.22656	18252.40234	20751.91602	17566.64063	19886.5625
Filtered	Filtered	Filtered	Filtered	Filtered	Filtered	Filtered	Filtered	Filtered	Filtered	Filtered	Filtered
Filtered	9593.84082	46704.84766	46018.19922	51142.35547	49801.33203	8464.404297	8028.514648	57685.93359	Filtered	4030.32373	7026.618652
4555.317383	12489.94238	Filtered	Filtered	Filtered	Filtered	13195.84375	7945.902344	25779.81836	Filtered	73554.10938	Filtered
Filtered	Filtered	Filtered	Filtered	6086.006836	Filtered	Filtered	Filtered	Filtered	Filtered	64317.25	Filtered
Filtered	Filtered	Filtered	Filtered	Filtered	Filtered	Filtered	24361.98242	52806.90625	23364.92383	Filtered	17724.31836
26862.33984	41001.67969	30916.19336	28737.94141	40681.64844	25950.52539	35051.89063	31570.98828	24983.08984	25911.71094	14749.05762	24676.29297
Filtered	Filtered	Filtered	Filtered	Filtered	Filtered	Filtered	Filtered	Filtered	Filtered	Filtered	Filtered

9425.349609	915.2052612	2230.256592	7915.42334	20719.90039	19651.18164	8128.200684	Filtered	Filtered	Filtered	Filtered	30959.34766
18318.875	33564.75	26516.33203	27744.25	32459.60938	20254.47656	20428.08203	23322.34375	23539.76367	25453.42969	17296.16016	28075.32813
30703.97656	Filtered	31195.42383	30870.76172	28348.19727	25749.375	30061.39258	30678.7207	22675.76172	29751.56836	Filtered	29103.2832
37492.40625	10881.80176	13954.90234	18309.8457	14882.2041	14918.29883	14582.3418	13576.1582	11469.21875	10521.05078	14550.38867	30523.20117
Filtered	Filtered	Filtered	Filtered	Filtered	Filtered	Filtered	Filtered	54619.52734	21460.01367	22128.61719	Filtered
37039.1875	41639.80469	29064.73047	33882.12891	37187.33984	29826.49219	33850.98047	27157.16016	15883.92676	27070.15234	16664.625	29368.82031
Filtered	Filtered	19280.50977	1791.650391	Filtered	Filtered	29543.02344	Filtered	Filtered	Filtered	Filtered	35553.1875
20165.29297	23062.70313	18500.40234	21070.49219	46172.80469	44154.25781	29617.92578	15764.36328	11683.41895	15330.29297	11349.02246	15290.23047
4915.191406	Filtered	Filtered	135381.3125	26393.21094	2011.013428	Filtered	Filtered	Filtered	Filtered	Filtered	Filtered
Filtered	2665.103027	4899.266602	Filtered	18999.55273	235462.7031	39663.82813	34860.60156	34648.67188	Filtered	44208.37891	32199.50195
27642.51563	30447.28125	23569.31836	26947.05664	24006.38672	26375.39063	25595.41797	31387.625	16285.05762	25580.83203	19804.09766	26458.85742
20216.58398	26286.41602	24344.15234	17481.02539	16362.17676	21159.55859	28750.26953	20440.0957	14070.90625	19846.49609	54053.54688	19389.05859
37675.90234	29319.28516	20536.49219	25788.53125	61101.48828	22919.6543	24149.49805	29910.23047	16062.6748	38146.70313	17267.99023	21995.12695
27288.64648	29676.24219	28377.25781	27219.32617	28588.93359	29817.05859	29964.26563	24641.51953	20859.33008	22050.27539	21239.36523	27947.23438
32920.23828	32759.92578	28519.4707	23418.96875	31441.00391	30854.30273	31659.46875	17245.26367	13856.11133	25999.29492	21741.80469	28340.4375
26249.03906	34606.26563	17881.39648	22744.21094	34362.49219	20653.37695	24443.18164	26907.19531	28550.02148	39328.10938	31703.01953	34386.91016
Filtered	42811.96484	Filtered	29104.29102	Filtered	30328.03711	27089.51367	43838.69922	26356.44141	Filtered	21942.81055	22193.13281
6329.117676	Filtered	Filtered	Filtered	9437.425781	Filtered	11139.68555	Filtered	Filtered	Filtered	Filtered	Filtered
7964.263672	7671.762695	37377.46094	1034.977905	10698.83105	3940.669434	5222.318359	9085.010742	11381.85352	9737.227539	53408.78516	22301.63086
Filtered	Filtered	Filtered	Filtered	Filtered	Filtered	Filtered	Filtered	Filtered	Filtered	Filtered	Filtered
10468.28906	Filtered	14594.68359	8716.525391	12828.71387	22865.62109	6573.181641	7452.830078	Filtered	26916.22461	20798.7207	Filtered
4551.516113	112178.4219	Filtered	9085.047852	3213.523438	Filtered	Filtered	Filtered	Filtered	Filtered	Filtered	47251.65234
33525.85156	26260.49219	29441.21484	26765.37695	21078.92969	25492.39258	16770.875	25736.62891	26517.69922	25169.9043	17274.67188	16006.14941
24409.76758	29147.74609	26965.96875	24776.46875	40274.52734	21174.89844	27191.73047	23534.19922	13297.6123	16987.49609	14883.27246	21449.6543
36251.69531	20613.3457	22163.02344	12797.95801	25071.79883	20533.81055	31381.84766	22448.03125	21967.97266	42854.4375	15233.17969	42430.92969
23804.05469	19738.86328	34152.64453	30952.56055	31389.70703	24472.88281	25114.28125	21708.66406	25607.58203	25533.33398	16640.33984	21470.11133
23641.74805	9355.347656	8057.73877	133252.3438	31816.36914	2937.886963	Filtered	38347.94922	Filtered	Filtered	Filtered	Filtered
Filtered	11663.10547	8145.5625	13347.88477	10997.20313	10099.83105	20908.77344	12786.18164	26504.88086	23796.37109	50452.55078	15331.66797
25453.5332	25506.33984	28345.72852	22148.42383	24468.89648	30059.03906	25422.84961	21154.9375	16606.79688	19637.07031	20130.32031	25663.40039
27451.99609	33534.16406	25826.45313	25830.28125	23875.6875	23729.50586	32942.07031	22993.49414	18072.07031	24739.5918	23112.73047	21744.30273
17365.88477	21583.10156	21770.33398	22051.69922	41555.42578	31612.22461	32538.78906	12803.05078	13474.33398	13294.43555	11576.15332	13240.83203
Filtered	23604.71484	Filtered	Filtered	Filtered	1265.492676	Filtered	Filtered	Filtered	Filtered	Filtered	Filtered
32328.0332	27042.0918	28032.43555	29681.82422	32012.02539	24696.28711	29503.28125	28441.58984	12769.98438	13920.60449	15220.69434	19859.71875
Filtered	Filtered	Filtered	Filtered	Filtered	Filtered	Filtered	Filtered	Filtered	Filtered	Filtered	Filtered
22414.25977	29443.13086	23249.55078	29743.40039	31263.16211	19724.52344	30540.16406	21371.94141	15422.19434	18354.12891	17591.64063	24334.32031
20966.03125	21722.19141	23972.86328	31517.79688	26168.25	20126.51758	29684.42383	19088.32031	22033.52344	18853.49023	20920.67578	19063.79492
15445.68848	29024.34961	9488.880859	39554.22656	31735.54492	23344.70508	28510.69922	23929.39258	16082.35156	19049.78711	17705.92188	13064.54492
19701.05859	7811.417969	14392.14648	13877.59668	39184.14844	51894.55859	25105.49219	12166.03223	8624.025391	7635.460938	10376.45898	7889.024414
Filtered	11952.79004	Filtered	Filtered	Filtered	21100.65625	Filtered	Filtered	Filtered	Filtered	Filtered	47099.17969
29123.63672	23529.2207	16676.72656	20663.57422	19321.39453	20809.91797	34449.53906	15320.56152	21770.67578	24172.91406	23174.67188	21717.15234
13796.62695	Filtered	10621.99023	13422.44043	10577.7793	10943.95313	Filtered	296720.4063	7660.712402	9217.556641	5835.011719	147473.9219
35871.00391	17428.31641	14896.87598	13705.69336	22967.39258	13547.65332	38897.16797	15556.70898	15991.91406	25732.01953	25565.42773	29886.07617
25782.53125	26217.64063	25659.80273	20932.17578	23639.88086	22982.46875	26649.39258	26849.70703	17344.05469	19720.04688	15641.05078	20194.98633
12442.17578	15303.52441	10185.10645	6862.605469	28492.66406	15107.1582	35065.09984	14309.25586	33254.10156	19981.23828	51882.57813	26699.88086
13123.02246	6416.71875	7853.231445	24501.57813	10069.39063	5584.473145	Filtered	14328.68359	Filtered	7418.162598	8422.621094	12774.1084
22785.125	5647.186035	25526.93359	13458.35742	32540.31055	33903.71094	6138.687012	13753.83496	13345.37402	9008.625977	7332.253418	7906.433594
27010.51367	23333.64258	24956.1543	28762.16992	25281.26367	21281.53516	31958.0625	20303.10938	13763.84277	21711.93164	17445.32031	22284.33594
24758.47656	25058.66992	21535.10547	20381.4082	29886.57227	24608.48828	21572.59766	28870.03125	13632.38086	17160.88281	18593.13867	18999.00391
23635.61328	7388.608398	21835.56445	10327.83301	20150.15039	12318.73828	11173.75391	20603.52148	22200.74609	12500.6875	7452.993164	17372.18945
23879.72461	23639.8125	29413.49219	22212.98047	35107.92578	20698.00781	15047.8916	20459.27344	17748.37109	17225.46484	16673.11523	16893.76953
26197.35742	24818.82227	23756.47656	21968.88672	18281.56445	22043.46484	27866.24805	19190.41797	17166.36328	16010.64941	14368.75684	16627.09766
Filtered	Filtered	7585.73877	22134.61719	4746.197754	Filtered	Filtered	8204.201172	Filtered	Filtered	Filtered	Filtered
22559.1875	22524.36719	24683.85938	28033.29492	23003.54102	26552.26172	20959.08203	24068.49414	17818.08789	20816.55078	18809.97461	21047.58594
Filtered	Filtered	Filtered	Filtered	Filtered	Filtered	Filtered	Filtered	Filtered	Filtered	Filtered	Filtered
12313.93945	7723.753906	9937.954102	10338.72461	6385.487793	9196.30957	9269.271484	15835.45996	7247.12207	8599.003906	9446.953125	10523.00586
17036.67188	15978.19336	20296.53516	15885.98633	20547.07617	12330.66992	16056.78809	16344.09375	12166.66211	12897.10742	12493.74023	15426.31543
Filtered	Filtered	Filtered	Filtered	Filtered	Filtered	Filtered	Filtered	Filtered	Filtered	Filtered	Filtered
20600.59961	58242.92969	13289.61133	14567.04004	14589.84766	15246.60449	14290.58789	18174.21484	15987.10547	19437.5957	18788.71875	9471.09375
27402.89648	23312.38672	19300.2207	5226.894043	26273.64844	4679.785645	22936.68555	37604.85547	26696.46875	15117.36914	20139.83203	24424.86719
27189.94727	14292.89648	16304.81055	28041.44531	14950.52441	13414.00977	25084.97461	27281.0625	20571.70898	26109.93164	19638.02148	26419.61914
Filtered	Filtered	Filtered	Filtered	Filtered	Filtered	Filtered	Filtered	Filtered	Filtered	Filtered	Filtered
21548.5625	12258.88184	36510.70313	16598.18945	23972.93555	16845.75391	21063.46875	12540.59375	24005.14063	15892.2627	8519.535156	13216.93945
Filtered	Filtered	6806.854492	Filtered	11327.53027	10068.37988	9572.555664	Filtered	25732.83398	19781.92383	20244.95313	9438.842773
17665.51563	19417.56055	24114.07422	19052.24609	21115.43164	25737.66406	13769.97266	14883.1875	15957.20508	12167.06934	15929.28516	14641.77148
16646.30859	23378.46875	18249.34766	19180.6543	20747.93164	17409.48438	20015.75781	18613.19336	13303.77246	18453.0957	16151.46484	13679.37891
Filtered	Filtered	30157.98828	Filtered	Filtered	Filtered	5862.119629	4497.786133	Filtered	Filtered	Filtered	Filtered
22020.53516	12572.21484	9508.351563	22477.78906	32115.08008	14199.58496	18388.43164	17120.95508	8327.796875	17322.93164	8058.911133	10435.24805
21944.50391	24570.3418	23630.86133	17566.69531	23283.23438	22089.50391	16266.08398	21214.43359	13430.56738	17495.12891	12791.55078	18961.91016
22570.98438	28643.77734	27632.39453	22263.38281	18592.79883	27371.02148	19940.38672	18097.51563	17410.76367	27561.40625	14529.81543	13755.95313
Filtered	Filtered	Filtered	Filtered	38678.44141	Filtered	Filtered	Filtered	Filtered	Filtered	Filtered	Filtered
7866.762207	8216.935547	6790.750488	12394.09766	9802.285156	4917.286621	25930.92969	8918.587891	46086.07813	18473.53125	30370.64648	10810.43359
23474.28906	21174.93164	20510.74609	20045.58594	19192.96484	20415.01367	21895.11523	16195.94238	16100.18066	22977.86914	14673.80078	18053.54297
10953.60352	9199.643555	14616.05078	8496.341797	15253.91211	10309.81543	42932.94922	14347.57129	46902.61328	16314.94336	30936.88281	16278.08594
9330.358398	Filtered	8956.728516	6022.409668	28751.33789	Filtered	9341.59375	Filtered	Filtered	Filtered	Filtered	15609.97461
30810.98633	Filtered	Filtered	Filtered	Filtered	Filtered	Filtered	Filtered	17502.82422	Filtered	Filtered	18887.04102

23216.48242	20997.64063	20947.62109	17616.52344	20005.21289	19300.17383	20541.36914	20765.33594	10717.98535	20146.40234	12605.19531	17928.49805
14700.56055	Filtered	33536.48828	Filtered	27021.24219	Filtered	24341.69336	25296.10352	Filtered	13231.47656	14310.13867	19451.24805
15194.68555	13881.36133	14253.74316	13862.0625	14468.0293	11361.8252	14711.16016	18022.31445	10463.68457	13313.45898	13325.64355	19039.33398
12025.10938	17787.48438	20169.82031	19604.28125	23828.69141	15939.01465	11240.34766	15537.06543	8546.137695	10615.70898	9401.422852	15666.93848
19522.98242	17448.79297	22090.99219	15705.49414	20257.08594	26405.47266	18471.90625	15191.01758	13039.24219	10828.50781	11082.71484	14486.64355
44798.90625	10513.07617	Filtered	5798.924316	13916.93652	11050.29199	10644.62012	19956.69727	12600.0791	15205.05664	16453.58984	13558.19629
13037.58105	13207.23242	89530.67188	11031.31445	44677.83203	23647.02734	8838.956055	9972.874023	15348.19434	14578.33203	37932.39063	12125.26465
6223.205078	149011.3906	13059.84668	5466.084473	65455.82422	2853.177979	16809.41992	7466.878418	Filtered	6892.630371	Filtered	11814.70313
Filtered	Filtered	38306.5625	Filtered	Filtered	Filtered	Filtered	1970.987427	Filtered	Filtered	17280.1543	19536.49023
16768.25977	20421.50391	9616.955078	6132.848633	6451.707031	10097.53809	21420.09961	29592.44531	19391.9707	18998.47266	19055.56445	14924.91309
25497.58594	19616.28125	18628.48633	16556.91602	18363.57422	21920.84961	14474.95117	18360.8125	19180.03906	17198.50586	14476.28809	14748.33789
Filtered	Filtered	Filtered	Filtered	2757.300781	Filtered	Filtered	Filtered	Filtered	20996.30469	Filtered	Filtered
2814.118164	3642.779785	2846.198975	95666.5625	36658.09766	6823.102051	3873.462158	20886.63281	2839.602051	Filtered	18318.79883	3189.66748
10360.21094	9924.439453	7800.411133	8652.404297	11646.66699	5319.497559	6352.421875	7828.619141	4858.469238	3963.769287	4920.366211	6810.344727
21691.13867	18256.60938	12422.03125	14638.5332	13420.75195	33223.49219	15059.31445	28973.45703	14214.96289	20339.62305	18722.67773	14680.01367
15548.35059	9228.957031	18848.07617	9135.623047	19459.62891	26287.57617	31242.98633	17536.52734	15434.79492	16487.47852	13523.75781	14448.17969
20056.19531	17713.23633	19196	17136.34375	17572.54492	19121.44727	14237.21777	19076.89063	15445.05859	16789.53516	19791.55469	13777.77051
17258.95508	16501.08398	15825.22852	27183.80273	17711.76953	18402.27344	13696.66992	18486.93555	10763.93359	27107.50781	18978.64844	16096.97852
8843.290039	Filtered	11046.37207	12185.33984	20185.73242	Filtered	Filtered	Filtered	24202.77734	Filtered	25050.83203	13296.41699
14145.96582	14767.04004	11786.84863	14877.89551	20538.24414	11175.08984	26591.50586	14728.73926	33019.21094	14029.47559	14382.52344	18685.97266
16158.49707	20379.25	19386.77734	17314.01563	14897.7207	22603.71094	18256.08594	18074.26953	16726.4375	13600.51172	16129.08398	12274.64648
18090.0957	17784.25195	18022.4043	14135.18359	18677.09766	13414.23535	16826.62305	15941.64063	11745.78906	14102.86133	13088.5918	16114.24316
17547.47656	17785.51172	17818.39258	18094.21875	17061.87305	12321.94531	18683.58789	18274.46484	14884.15918	18523.00781	12658.12402	14554.53125
Filtered	Filtered	Filtered	Filtered	Filtered	Filtered	18160.38672	Filtered	23936.95898	6773.952148	19412.05664	Filtered
7503.428711	Filtered	5662.794922	Filtered	13919.35254	8653.491211	15220.86816	10546.03027	37156.19141	16272.99902	26335.5293	14573.14844
11331.50195	13401.25684	12276.60059	12730.8457	25423.46289	20230.93164	17127.2207	7841.751953	9320.678711	9891.275391	8316.088867	8637.255859
19680.01758	17373.3418	20266.65039	12386.23438	11084.57031	15832.94922	14412.62012	16139.42188	18197.64063	12190.40234	16538.44141	23139.20117
Filtered	Filtered	11236.08496	Filtered	Filtered	18001.58789	20494.38086	20140.72461	Filtered	Filtered	11820.95508	12700.13574
18080.65625	8760.950195	11392.80859	16170.77734	11063.63379	22763.35938	10853.03809	16520.39648	5429.396484	10963.07324	21324.24805	8220.861328
21655.57422	13083.91992	22248.58984	Filtered	17601.64648	Filtered	23071.64063	27236.72852	Filtered	14638.2334	9635.976563	17041.10938
Filtered	Filtered	Filtered	Filtered	Filtered	Filtered	Filtered	Filtered	Filtered	Filtered	Filtered	Filtered
7477.664551	5634.592773	6718.430176	7052.027344	10320.11328	18401.3418	19917.80664	10675.2832	65852.79688	32657.52148	27943.67773	9407.34668
14909.15527	16451.53711	18750.31055	11120.40039	16506.88086	13998.64648	27534.04297	24171.16211	18957.19727	13640.17578	9073.141602	16973.27344
9107.799805	29200.73047	20516.52734	24100.16406	7239.864746	2767.431641	63808.46875	3236.843018	15511.16016	7597.780273	17958.41797	52177.37891
9130.261719	14852.63379	21129.15625	19690.43945	10542.2168	7286.753984	16541.26758	22908.07227	10246.55859	19884.30078	16862.00195	17612.51953
19871.92969	23703.30273	16160.3877	15428.19336	17783.82031	9276.411133	22597.79102	17194.89258	17770.8457	17760.94336	19072.54688	15786.81445
Filtered	Filtered	Filtered	Filtered	Filtered	Filtered	Filtered	Filtered	Filtered	Filtered	4012.198975	Filtered
17113.69531	13597.40332	13816.49609	8617.029297	17643.93555	17099.25195	14880.9082	22278.01953	13926.40039	20662.45313	12136.17578	15822.63574
14897.21582	Filtered	10821.44141	6851.291016	14260.33398	15396.98438	Filtered	3749.60083	11845.3916	Filtered	18642.82031	10462.15918
24406.66797	17410.78125	16507.12695	14941.60352	16513.31641	10453.57129	13799.15039	18085.02148	15875.17285	12058.2627	14283.40723	14293.40723
Filtered	Filtered	Filtered	3418.573242	1683.605103	Filtered	Filtered	4338.891602	Filtered	Filtered	5178.518555	37953.03125
14785.10938	13605.6582	17847.80078	16606.53516	16471.32617	9722.708984	10785.36133	15323.48926	9339.227539	9949.419922	8159.410645	9542.612305
Filtered	Filtered	1654.773193	Filtered	Filtered	Filtered	Filtered	2487.04126	Filtered	Filtered	5062.427734	108573.3672
20898.69922	27284.36133	22886.81641	13426.75586	18225.36133	12868.31738	17911.19531	22695.62891	Filtered	Filtered	17030.64844	18029.35742
11058.95215	3535.576172	4076.665283	9912.617188	3500.002686	2608.498047	Filtered	16382.8418	8948.366211	5688.610352	4813.092285	9199.338867
10953.75098	12267.10742	7458.469727	8653.835938	11885.48828	10235.6377	15488.25586	13049.74414	82974.64844	39514.89453	13003.61719	25630.36328
Filtered	Filtered	Filtered	Filtered	7870.446777	16324.87891	Filtered	Filtered	Filtered	Filtered	Filtered	Filtered
5494.810547	Filtered	Filtered	Filtered	Filtered	Filtered	Filtered	Filtered	Filtered	Filtered	Filtered	5134.934082
18753.56445	21658.38867	18358.74805	13556.9043	18814.42188	14265.4873	18419.18164	12441.87988	12093.9541	12059.03809	9792.316406	13699.30469
11501.04395	7633.422852	68461.74219	9826.441406	27538.13281	16974.81836	6022.330078	5913.077148	8821.290039	7286.066406	28444.50391	7167.926758
22335.96484	19440.41016	15346.01367	22168.24609	17215.21875	Filtered	18490.98828	11806.01953	13871.04492	13364.21484	17736.23633	11199.2666
15675.87305	14118.08789	9016.817383	26995.32031	10853.17188	8341.1875	8705.213867	19793.01367	10959.65039	14304.18359	7186.047852	13056.98828
20578.14648	17711.52734	14909.45703	11802.57715	8996.543945	11622.33984	15399.0498	13149.57227	9365.938477	19109.38672	13830.99316	14341.15723
Filtered	Filtered	Filtered	3378.82373	3068.46582	3642.104736	62200.99219	60969.84375	Filtered	Filtered	Filtered	Filtered
18155.28125	12387.45703	16658.10547	11642.8125	9450.238281	9570.90332	7747.494141	11282.63379	9961.395508	8576.703125	8513.328125	7291.541016
15515.14355	13237.83496	9256.195313	13910.60547	8886.058594	9277.693359	16238.88184	16599.71484	10768.52051	15053.4082	21557.01758	12540.84668
22992.71875	15057.77539	17610.82813	11431.36426	15272.53418	17929.8418	12606.72852	16322.71875	9898.939453	16542.58398	14407.04785	11955.57129
4739.291016	9191.897461	7713.722168	Filtered	6840.822266	9913.87793	16668.79297	Filtered	44495.24609	19885.17383	25248.13867	Filtered
12576.37207	16907.52734	13632.04688	13925.09082	15865.27441	14714.19434	16878.24805	14331.84961	9285.05957	12845.83203	11118.21484	14403.82715
Filtered	1488.333008	Filtered	Filtered	Filtered	3466.159424	8927.669922	Filtered	31291.38477	5471.787109	14895.52148	8258.251953
Filtered	Filtered	6449.651367	Filtered	Filtered	Filtered	14264.36133	8713.5	38614.36719	21437.19727	37955.54297	14971.90332
15300.53418	Filtered	11665.49121	13259.33594	6513.336914	5226.787109	6945.317871	6347.679688	Filtered	3677.29126	12838.02441	10441.18848
16377.83008	16432.07422	18611.61523	6102.770508	17591.78125	8574.324219	21066.98242	13282.02148	8936.237305	11430.57324	12345.64551	14904.97559
19131.74609	18345.45703	16038.45996	13802.77539	13958.31641	10291.81934	16214.72559	14283.62402	10788.71191	14573.12305	13707.23438	21396.375
15843.79102	12383.66211	11022.22852	10133.26367	13605.46777	19722.72266	16384.82031	16694.82031	14207.08008	15117.11328	14328.0752	13749.28027
Filtered	Filtered	Filtered	Filtered	Filtered	Filtered	5971.94043	Filtered	18252.24414	7317.612793	5360.608398	Filtered
Filtered	Filtered	Filtered	Filtered	Filtered	Filtered	Filtered	Filtered	22756.98438	Filtered	11569.98242	Filtered
Filtered	Filtered	Filtered	Filtered	Filtered	Filtered	Filtered	Filtered	Filtered	Filtered	Filtered	Filtered
Filtered	Filtered	Filtered	Filtered	Filtered	Filtered	Filtered	Filtered	15509.95996	Filtered	7441.438965	Filtered
Filtered	Filtered	7732.756348	Filtered	Filtered	87655	Filtered	Filtered	Filtered	6004.578125	Filtered	8808.725586
Filtered	13425.10645	Filtered	Filtered	Filtered	Filtered	Filtered	Filtered	Filtered	Filtered	Filtered	Filtered
11109.71582	15137.89355	15141.63867	13075.44922	15483.4541	10978.11719	16605.85547	12237.65625	11205.85645	9023.651367	11640.03125	13967.95215
14975.96094	8132.291016	5055.941406	22428.19141	14538.47559	9062.748047	Filtered	25048.71484	8208.255859	12705.88281	10524.37109	14167.4043
10401.46777	14045.93164	13109.39844	17890.80664	16729.07422	12918.41602	18261.86914	16564.5625	13993.61328	14550.61816	12062.69824	12062.69824
Filtered	12267.35547	8510.927734	20948.33008	2691.50293	Filtered	Filtered	18844.75195	Filtered	14511.62891	Filtered	12126.24121

Filtered	Filtered	4254.657715	7000.165527	5230.255371	5455.670898	Filtered	Filtered	Filtered	Filtered	Filtered	Filtered
Filtered	Filtered	Filtered	Filtered	Filtered	Filtered	Filtered	Filtered	Filtered	Filtered	46137.98438	2200.028564
6275.634766	13335.03516	16849.43359	7547.763672	17133.28516	7049.853027	11785.72852	18455.33203	Filtered	Filtered	Filtered	8485.282227
12050.91406	18156.93164	11973.79492	15377.31836	16148.49121	15822.89063	9844.419922	10555.08203	8983.032227	11794.71094	10475.43652	9310.349609
Filtered	6642.504395	Filtered	5650.222656	48806.83203	8268.258789	68865.60938	6006.264648	Filtered	Filtered	4269.168457	5843.97998
7293.538086	10767.36523	8227.259766	6263.48291	15460.37988	13589.87988	26635.86133	9677.09375	30378.72266	13916.16309	34473.375	18012.70313
Filtered	13390.83008	12936.57227	13909.33789	16287.7207	12444.69531	13716.96973	13996.27441	5708.461914	Filtered	13612.86328	15598.74805
17229.41406	16748.29883	11286.73535	15301.62207	15907.54492	10049.31348	16291.80762	17604.26758	9494.037109	11314.0293	8884.926758	13142.61328
12824.44141	14864.93262	13527.23047	15636.63867	10249.5498	8302.93457	15199.47754	10467.86523	12980.55664	9255.692383	20393.22656	11854.96387
17090.48242	16598.20703	14918.16211	11866.44336	17589.10352	13306	15737.60938	14235.90137	14450.0625	11813.04492	10996.68945	15013.55371
41970.83594	22097.71875	8838.185547	15191.80078	6342.595703	13913.03516	3189.84082	12357.13672	2981.332031	15281.48633	4549.519531	20625.32422
Filtered	Filtered	4.04E+03	Filtered	Filtered	6.83E+04	Filtered	Filtered	Filtered	7.65E+03	Filtered	Filtered
15469.10742	15477.4834	14109.93945	15853.36035	14404.5332	11810.81348	14470.25195	13786.74414	11816.16699	13151.87305	9534.62793	11226.58105
Filtered	Filtered	Filtered	Filtered	Filtered	Filtered	Filtered	Filtered	Filtered	Filtered	Filtered	Filtered
12603.32227	13766.08496	15142.47656	12918.04883	12913.78809	15409.66211	10322.01074	11669.59375	7619.736816	9326.84375	10586.42773	13163.62598
Filtered	Filtered	12929.90234	Filtered	Filtered	Filtered	Filtered	Filtered	Filtered	12922.53418	11976.97949	15083.93457
Filtered	Filtered	Filtered	Filtered	Filtered	Filtered	Filtered	Filtered	Filtered	Filtered	Filtered	Filtered
8023.245605	8703.24707	7026.873047	8195.574219	6538.528809	6464.134277	6270.128906	8200.222656	7970.049316	10377.09375	8346.336914	7231.072266
10614.78223	10282.35547	10721.48242	14683.31836	13194.22363	15295.05957	9338.383789	11963.00098	14773.67188	8140.117676	11431.70703	12539
Filtered	Filtered	Filtered	Filtered	4327.536133	Filtered	11931.55273	Filtered	22607.35352	8510.015625	11743.97656	9678.802734
13156.31055	7212.946289	Filtered	Filtered	Filtered	Filtered	Filtered	Filtered	Filtered	Filtered	Filtered	Filtered
4105.624512	3992.754395	4033.69043	Filtered	6031.456543	4822.907227	7772.036133	Filtered	44704.41797	2196.443359	1964.325317	Filtered
Filtered	Filtered	Filtered	Filtered	Filtered	Filtered	Filtered	Filtered	Filtered	Filtered	Filtered	Filtered
10448.48438	9054.06543	19965.80078	9189.248047	12896.97754	18656.25	13450.16309	7927.294434	7427.019043	13883.2207	20427.66602	9043.71875
Filtered	Filtered	Filtered	14601.67578	Filtered	10936.12305	Filtered	15847.87109	Filtered	Filtered	Filtered	Filtered
Filtered	Filtered	Filtered	Filtered	4134.234375	Filtered	Filtered	Filtered	23812.36328	9545.454102	10994.06836	Filtered
8751.592773	6903.720215	Filtered	7846.675293	4463.355469	6871.186523	5782.270996	7347.072266	Filtered	7134.917969	Filtered	11577.42383
15875.39453	12030.16113	15354.76465	13406.37891	15197.86621	19723.50195	14649.14453	10691.11914	8736.850586	Filtered	10384.2832	10712.29883
14926.63281	12149.41504	15709.15918	10418.78027	16957.02148	14912.14844	10116.93262	12842.91895	5701.599609	7231.189941	9635.90332	6720.927246
Filtered	Filtered	Filtered	Filtered	Filtered	Filtered	Filtered	14419.78418	Filtered	Filtered	Filtered	Filtered
12215.50098	7704.704102	6444.688965	8664.932617	8295.925781	8950.414063	17520.59375	11776.84766	8495.255859	8652.417969	13526.98047	10406.40918
Filtered	Filtered	Filtered	Filtered	Filtered	Filtered	Filtered	Filtered	6802.022461	Filtered	Filtered	Filtered
11768.86328	17524.39453	9940.316406	6205.388672	17408.70117	15253.85645	15924.21289	15547.0625	6665.219727	6727.883789	4658.646973	4806.11377
9153.041992	12039.82422	12981.27441	9189.454102	9090.861328	3350.967773	10368.51172	8925.628906	4956.984863	Filtered	Filtered	9992.075195
10568.81152	13356.13574	11766.47754	7104.12793	18783.97266	12956.9082	11584.09766	13801.13281	7818.610352	5333.464844	6673.225586	14986.73633
13768.83203	Filtered	13034.65039	2035.595459	8183.908203	Filtered	Filtered	Filtered	Filtered	Filtered	Filtered	Filtered
Filtered	18345.26367	6751.547363	1585.779785	Filtered	14197.99121	Filtered	Filtered	Filtered	14635.2207	15074.19141	7127.96875
5945.880859	3938.58252	1317.087646	6234.423828	4220.330078	3504.069092	342752.9375	Filtered	Filtered	Filtered	Filtered	Filtered
3308.573242	6760.699219	5730.703613	7438.720703	9134.050781	6043.459961	29021.86133	5897.743164	Filtered	65136.45703	10690.47461	105622.3359
10562.76758	5768.06543	8516.55957	11022.49805	8639.798828	3860.574707	6406.42627	10202.5752	8043.862793	7164.086914	6740.584473	9915.22168
Filtered	8241.947266	Filtered	Filtered	Filtered	Filtered	Filtered	Filtered	Filtered	Filtered	Filtered	Filtered
Filtered	Filtered	Filtered	28606.36914	Filtered	Filtered	Filtered	Filtered	Filtered	7222.819336	4361.228516	Filtered
Filtered	Filtered	Filtered	Filtered	Filtered	Filtered	Filtered	Filtered	11232.04004	Filtered	Filtered	Filtered
13985.20801	13338.00586	11512.46289	16502.18945	7711.20166	13225.83203	16428.55078	13998.10449	5160.150391	8094.57666	9935.849609	20550.43359
Filtered	Filtered	Filtered	Filtered	Filtered	Filtered	Filtered	Filtered	Filtered	Filtered	Filtered	Filtered
10375.09961	4968.956055	3886.899658	7629.175781	9109.916016	10575.99902	10600.7832	Filtered	Filtered	Filtered	Filtered	Filtered
Filtered	Filtered	Filtered	Filtered	Filtered	Filtered	10762.36816	Filtered	31144.18555	Filtered	11405.98047	Filtered
18834.53906	Filtered	3665.960693	3787.666748	Filtered	Filtered	4043.63623	6090.05127	2555.383545	3679.432617	4000.030762	6040.568848
10204.6123	4649.539551	6570.33252	Filtered	6056.456543	5553.228516	Filtered	7717.132813	Filtered	Filtered	Filtered	7797.183594
7596.494141	10929.71094	11452.14453	10409.85156	16242.54297	11210.49414	12649.00195	9259.207031	6972.713867	10183.90039	5264.133789	23214.74414
10190.85156	8580.588867	8660.095703	5749.914551	11304.28516	9179.111328	10331.25977	14313.13867	11230.5	7689.086426	11121.20898	10163.67773
9055.0625	10084.2373	8075.762695	9422.605469	11400.29883	9122.935547	14925.61523	12994.51953	7613.105469	11405.02441	11347.39453	14078.49219
14410.34375	16704.85156	10279.61914	15568.33105	10849.03613	5864.34082	10467.26074	11774.44043	12113.25098	9177.967773	6939.410645	11526.15039
7116.818359	8178.636719	10672.09863	13904.40723	9782.480469	8510.233398	9138.357422	11091.51074	18793.60547	11226.72266	7345.636719	5889.793457
Filtered	Filtered	Filtered	25399.30664	Filtered	Filtered	Filtered	Filtered	Filtered	Filtered	Filtered	Filtered
10719.1084	9388.228516	10587.11133	9217.020508	11960.95898	10039.94336	7020.723145	7965.289551	6036.237793	9101.301758	11695.2168	8372.554688
11846.42871	17997.90234	14333.11523	12155.2998	8205.552734	7501.65332	1981.91687	16721.04883	Filtered	12775.28613	13399.26758	12163.36621
8558.109375	11480.1377	14463.30371	13206.11914	13135.64063	16879.39258	12951.41797	10407.91602	8512.602539	14891.29688	9103.19043	9147.008789
2669.407959	Filtered	16239.84277	13551.79883	9228.635742	10536.67969	Filtered	Filtered	Filtered	Filtered	Filtered	Filtered
15557.29688	Filtered	Filtered	2535.536621	5243.775879	3523.169678	Filtered	5678.426758	Filtered	Filtered	Filtered	7176.105957
Filtered	Filtered	Filtered	Filtered	5802.429199	Filtered	Filtered	Filtered	Filtered	Filtered	Filtered	1751.494507
1361.99646	10120.85645	1506.19043	6309.425781	8432.757813	Filtered	86038.98438	Filtered	1196.99585	Filtered	Filtered	Filtered
12929.23828	8424.43457	10507.94922	10954.84766	9607.665039	9687.263672	9819.022461	9248.550781	6406.117188	6495.989746	8309.619141	9835.397461
3281.327881	965.3642578	2024.105957	1965.77002	2437.695801	775.9578247	38936.3125	Filtered	Filtered	Filtered	30443.02148	2613.264404
7275.295898	7447.374512	11215.23438	9497.049805	12639.96875	13184.54102	9455.59082	5167.094727	6432.327148	8875.253906	6461.386719	6691.865234
Filtered	10863.26367	10497.45605	25359.07227	1831.83728	28879.41406	Filtered	Filtered	43872.49219	Filtered	19398.59766	31891.70117
15609.35059	7067.410645	5906.931641	13612.97754	10353.9248	7373.345215	Filtered	13538.28711	12074.44531	6602.165527	Filtered	11272.15625
11905.80371	11432.49707	9032.427734	11156.88574	8844.192383	8807.24707	10609.56836	5726.464844	8362.224609	9597.793945	8049.11377	7700.48291
Filtered	Filtered	11980.50684	10709.16113	8410.380859	13591.94141	8651.929688	Filtered	Filtered	Filtered	Filtered	Filtered
15437.70508	4273.072754	3713.954102	5711.884766	4429.10498	Filtered	Filtered	7313.569824	Filtered	Filtered	Filtered	5479.185059
5933.886719	6313.497559	5294.87207	32233.75977	6981.255371	6140.409668	6398.395508	4175.969238	29028.07227	7269.068848	40667.76172	6308.948242
4971.817871	6418.661621	20987.0918	5556.393555	5309.809082	Filtered	16790.55078	3698.797119	7744.276855	6422.479492	5938.619141	5983.425293
Filtered	Filtered	Filtered	Filtered	Filtered	Filtered	Filtered	Filtered	Filtered	Filtered	Filtered	Filtered
Filtered	Filtered	Filtered	Filtered	Filtered	Filtered	Filtered	Filtered	Filtered	Filtered	Filtered	Filtered
Filtered	Filtered	Filtered	Filtered	Filtered	Filtered	Filtered	Filtered	Filtered	Filtered	Filtered	Filtered

12328.66211	6164.178711	11395.71289	6664.730469	5389.95752	Filtered	7467.057617	6900.40332	10064.25098	Filtered	Filtered	Filtered
Filtered	Filtered	9187.06543	6073.894043	9061.5625	10806.08887	11862.42578	11438.66797	Filtered	Filtered	Filtered	Filtered
6106.999512	9195.742188	7220.897949	Filtered	12922.95801	6364.444336	5349.177246	4141.813477	Filtered	Filtered	12158.27832	4427.345215
Filtered	Filtered	8709.376953	3484.924072	4401.635254	Filtered	Filtered	Filtered	Filtered	Filtered	14259.69531	11875.17383
Filtered	Filtered	Filtered	Filtered	Filtered	Filtered	Filtered	Filtered	Filtered	Filtered	Filtered	Filtered
12566.27539	16157.17578	11012.33301	9777.558594	12080.31738	5393.480469	10358.64551	Filtered	Filtered	Filtered	9890.489258	Filtered
Filtered	Filtered	9243.076172	Filtered	Filtered	6220.000977	Filtered	Filtered	Filtered	Filtered	Filtered	Filtered
Filtered	Filtered	Filtered	Filtered	Filtered	Filtered	Filtered	Filtered	Filtered	Filtered	Filtered	Filtered
8073.847656	5978.495605	7082.11377	8554.601563	5903.473633	3799.427246	6642.712891	6947.073242	5071.718262	5998.15332	5824.629883	6379.864258
11483.14355	13605.72363	11163.63184	9540.161133	8957.533203	9645.768555	6200.717285	9381.768555	11582.12402	4864.394531	4483.354004	6646.037598
Filtered	14086	9451.557617	9372.618164	7632.231445	10644.14746	13131.49609	5452.11084	Filtered	9261.492188	4290.241699	Filtered
9219.167969	9800.873047	Filtered	4891.043945	9077.506836	7325.407715	10702.8584	9977.609375	Filtered	Filtered	10594.42676	3889.657471
Filtered	Filtered	Filtered	Filtered	Filtered	7336.180664	Filtered	Filtered	Filtered	Filtered	Filtered	Filtered
11898.74316	8382.49707	10018.6084	10742.57422	10426.24805	6522.583496	6589.373047	11071.02637	Filtered	4183.225586	10132.60059	5917.354492
5623.494629	7356.074707	7530.638184	8146.697266	7249.615723	5145.788574	5888.208008	6274.741211	4392.739258	6389.395508	6889.421387	8168.391602
3857.726318	5587.658203	5918.241211	7240.922852	6599.035645	14781.00195	5423.370605	6449.632813	5116.811523	7679.26416	7382.835938	Filtered
Filtered	10883.79199	11061.39355	8672.19043	11442.18164	6550.203613	Filtered	Filtered	Filtered	8468.147461	5047.524902	6369.497559
8583.771484	4862.700195	8176.453613	5983.472656	11739.58984	6448.734863	7025.368652	7282.915527	Filtered	Filtered	11150.59375	5096.641602
47570.66016	2409.623535	3507.900146	3659.935059	Filtered	1554.471802	2776.258545	32165.52734	Filtered	Filtered	Filtered	Filtered
11060.37012	10758.53809	6337.970215	Filtered	9491.410156	9505.995117	8686.398438	Filtered	Filtered	10422.89063	5724.769531	6099.021973
13462.61035	9899.082031	8058.373047	8936.805664	7740.901367	Filtered	Filtered	11780.15918	7010.868164	8843.547852	7474.972656	14955.37402
Filtered	Filtered	2970.339844	31833.51172	Filtered	Filtered	Filtered	Filtered	Filtered	Filtered	2211.872559	Filtered
5841.56543	Filtered	6457.408203	4474.352051	4594.553223	5492.973633	8117.15625	10524.61621	Filtered	Filtered	Filtered	Filtered
11509.76465	10111.56836	Filtered	Filtered	8253.30957	7400.896973	Filtered	9430.774414	5843.461914	13528.80176	7872.227051	9489.353516
10605.36719	Filtered	10118.02539	Filtered	Filtered	Filtered	Filtered	4153.523438	8693.711914	Filtered	Filtered	Filtered
10194.14844	8002.68457	10255.5293	7819.325684	7899.844238	5638.770996	7486.825684	7658.527344	5709.714844	6327.689453	7687.505859	9930.646484
Filtered	Filtered	Filtered	Filtered	Filtered	Filtered	Filtered	Filtered	Filtered	Filtered	Filtered	Filtered
7455.63623	Filtered	Filtered	3832.761719	Filtered	Filtered	Filtered	Filtered	Filtered	Filtered	Filtered	4838.46875
11536.33008	8996.421875	7138.234863	12381.20313	4997.726563	4924.745117	Filtered	17193.84766	5759.124023	7195.660156	Filtered	9572.214844
Filtered	6535.597168	7520.266602	11060.55469	7906.17627	4638.148438	Filtered	Filtered	7582.050781	5853.835938	Filtered	7691.799805
11665.20898	3828.453613	6081.505371	4622.661133	8745.169922	7898.609375	3845.991943	Filtered	Filtered	Filtered	2925.9729	Filtered
Filtered	Filtered	8619.87793	Filtered	9626.572266	10222.1875	Filtered	Filtered	Filtered	Filtered	Filtered	Filtered
Filtered	Filtered	Filtered	Filtered	Filtered	Filtered	Filtered	Filtered	Filtered	Filtered	Filtered	Filtered
10700.16992	10707.61328	10975.18359	10072.14063	9327.412109	8455.035156	9000.751953	6912.614258	Filtered	Filtered	Filtered	8536.436523
13918.76074	7597.083008	6384.703613	6404.288086	8285.972656	6929.046875	11899.03418	13153.32422	Filtered	Filtered	Filtered	11592.43359
7810.874023	9159.179688	10558.80957	11090.8291	9358.071289	5235.158691	3345.908447	7570.517578	8354.03125	7044.779785	7780.626465	5356.776367
8876.667969	9346.008789	8064.463867	6583.947266	8697.007813	11311.35352	7471.374512	7981.075195	3916.012695	9901.507813	8460.587891	7378.120117
2505.640381	3013.427734	7541.09668	6463.947266	10757.63477	6731.175293	3414.633545	55676.53125	Filtered	Filtered	Filtered	4766.800781
10462.49707	4340.532715	5390.026367	9624.180664	4223.92627	Filtered	Filtered	11365.47461	Filtered	Filtered	Filtered	9077.96875
8149.953125	8175.53418	8078.639648	7073.17041	6956.791992	6019.368164	5811.63623	7011.861328	3016.56543	4357.253906	6426.341309	5896.99707
Filtered	Filtered	Filtered	Filtered	Filtered	Filtered	Filtered	Filtered	Filtered	Filtered	7104.032227	Filtered
6309.666992	6297.749512	7696.791504	10444.01953	8464.56543	5401.875	9230.037109	7476.120605	3744.043945	6233.978516	3761.82666	7274.173828
10179.9043	8101.728516	4267.238281	10093.00391	5503.495117	4605.108887	6382.930176	9807.771484	7057.439453	8228.702148	6250.600098	6621.923828
6725.947754	8206.948242	6377.908203	5752.605469	6532.229492	5907.118652	Filtered	7365.482422	Filtered	Filtered	Filtered	7141.282715
3807.0625	8568.227539	Filtered	Filtered	2350.405029	Filtered	6853.753906	7682.307617	Filtered	11154.16504	4000.674316	3705.957031
Filtered	Filtered	Filtered	Filtered	Filtered	Filtered	Filtered	Filtered	Filtered	Filtered	Filtered	Filtered
10960.70605	6330.454102	7579.748535	6441.425781	8590.539063	11642.16699	Filtered	9143.920898	Filtered	Filtered	8747.405273	3848.045898
Filtered	Filtered	Filtered	13312.97754	Filtered	Filtered	Filtered	Filtered	4422.318359	Filtered	Filtered	Filtered
Filtered	Filtered	9539.541016	9653.289063	11165.01172	8731.006836	Filtered	6649.319336	Filtered	7126.879883	6940.755859	5481.839844
3586.05127	3932.385254	3518.205322	3456.912354	3975.68335	3416.36499	3292.941162	4261.17041	Filtered	Filtered	2859.2229	3218.611816
5530.431641	Filtered	Filtered	Filtered	8312.882813	Filtered	Filtered	Filtered	Filtered	Filtered	Filtered	Filtered
7329.653809	9106.453125	8925.134766	10352.87109	7926.688477	7225.229492	8359.057617	9375.59082	5980.438477	6426.02832	7116.829102	8555.943359
9756.728516	6314.487793	12984.29492	6131.281738	Filtered	Filtered	9120.791016	8831.487305	Filtered	6062.495117	5815.458984	9712.788086
6730.841797	Filtered	8297.936523	8468.333984	9779.399414	9232.021484	Filtered	Filtered	Filtered	Filtered	Filtered	Filtered
6135.720703	7532.254883	5216.00293	3202.432373	9725.859375	6435.833008	8729.162109	8939.044922	2439.72876	1735.270874	2587.259033	2350.55957
7479.524414	7616.732422	8326.886719	8195.736328	9058.751953	10379.43848	5576.008301	7895.915039	Filtered	5821.666016	6693.119141	8180.198242
Filtered	11731.93359	6283.183594	5461.577637	3937.227539	11475.64941	5883.152832	10035.78223	6126.931641	7764.950195	3927.661133	4193.046875
Filtered	Filtered	Filtered	Filtered	Filtered	Filtered	Filtered	Filtered	Filtered	Filtered	9057.790039	Filtered
8998.896484	5461.083496	7033.58252	6438.530762	8907.556641	6604.861328	7693.112305	8331.634766	Filtered	Filtered	4464.310059	13573.57715
Filtered	Filtered	Filtered	Filtered	Filtered	Filtered	Filtered	Filtered	Filtered	Filtered	Filtered	Filtered
4508.762695	12117.71582	4727.241699	5185.945313	5608.500977	7034.078125	13213.62695	4175.474609	11148.47266	13968.88086	9841.003906	10885.3916
Filtered	Filtered	4404.364746	Filtered	3734.560791	Filtered	Filtered	Filtered	Filtered	Filtered	Filtered	Filtered
Filtered	Filtered	4940.491699	5606.422852	5622.086426	Filtered	Filtered	7135.123047	Filtered	4085.233398	Filtered	Filtered
7509.470215	7123.662598	7409.410156	4821.493164	8434.845703	9108.061523	5111.158203	4726.887695	6187.768066	6809.206055	3299.633789	5113.148438
Filtered	Filtered	Filtered	Filtered	8242.229492	8520.899414	Filtered	Filtered	Filtered	Filtered	Filtered	Filtered
7320.056152	3997.063965	5985.373535	6016.408691	6191.999512	Filtered	Filtered	8470.40332	Filtered	Filtered	Filtered	Filtered
Filtered	Filtered	Filtered	Filtered	Filtered	Filtered	Filtered	Filtered	Filtered	Filtered	Filtered	Filtered
7714.086426	9592.774414	6826.764648	9192.068359	7116.123535	7091.328125	4170.767578	Filtered	Filtered	Filtered	Filtered	Filtered
6109.790039	Filtered	Filtered	Filtered	Filtered	Filtered	Filtered	Filtered	Filtered	5968.048828	Filtered	Filtered
Filtered	Filtered	9911.924805	Filtered	Filtered	3440.361816	Filtered	Filtered	6063.249023	4057.752197	12944.75391	9746.441406
1805.852295	10854.78027	9748.788086	4592.749023	4219.286133	9231.00293	9037.798828	3737.073975	8589.727539	7492.370605	Filtered	4531.336426
11894.86621	8487.166016	6295.25	4995.193359	5401.002441	5948.84082	6690.298828	8987.479492	Filtered	7938.296875	Filtered	7751.987305
Filtered	6634.779297	5984.015625	4123.883789	5320.722656	6086.603516	7341.684082	11503.86719	7535.064453	Filtered	4664.98291	5189.409668
12081.50293	Filtered	Filtered	4787.558105	Filtered	Filtered	Filtered	Filtered	Filtered	Filtered	Filtered	Filtered
Filtered	Filtered	Filtered	5331.067871	Filtered	6247.372559	Filtered	Filtered	Filtered	Filtered	Filtered	Filtered

6816.960938	5734.82373	6826.496582	7767.583984	6910.138672	Filtered	Filtered	5105.195313	3455.080322	5763.89502	6322.914063	6799.400879
8350.572266	Filtered	Filtered	4673.780762	Filtered	Filtered	Filtered	Filtered	Filtered	Filtered	Filtered	Filtered
9030.444336	7679.486816	8469.449219	5505.326172	6707.316406	8484.75	Filtered	6460.063965	Filtered	6281.442871	Filtered	5076.386719
10359.94922	8900.927734	6357.484375	6706.25293	6318.124023	Filtered	10441.22559	10750.00977	Filtered	7191.851074	4788.739258	4048.594727
Filtered	Filtered	Filtered	Filtered	Filtered	Filtered	Filtered	Filtered	Filtered	Filtered	Filtered	Filtered
6794.529297	Filtered	5606.335938	9917.28418	6047.223633	4125.386719	Filtered	3780.786865	4193.73291	5457.04248	7501.30957	6014.438477
Filtered	Filtered	Filtered	Filtered	10729.08594	6264.230469	Filtered	Filtered	Filtered	Filtered	Filtered	Filtered
4928.246094	Filtered	Filtered	Filtered	Filtered	Filtered	Filtered	Filtered	Filtered	Filtered	Filtered	Filtered
Filtered	6922.496094	Filtered	7935.610352	3154.238281	Filtered	Filtered	9223.185547	Filtered	6230.346191	Filtered	6316.235352
Filtered	Filtered	11966.11816	Filtered	Filtered	Filtered	Filtered	Filtered	3027.85376	Filtered	11484.33691	Filtered
Filtered	Filtered	2408.483887	5211.916992	3503.305176	Filtered	Filtered	Filtered	6280.966309	Filtered	Filtered	Filtered
Filtered	Filtered	Filtered	Filtered	Filtered	Filtered	Filtered	Filtered	Filtered	Filtered	Filtered	Filtered
2085.476318	Filtered	Filtered	1587.172485	2347.307129	Filtered	6941.395508	3093.107422	11448.49707	5686.587891	4297.568848	4296.768555
Filtered	13939.5918	Filtered	Filtered	Filtered	Filtered	Filtered	Filtered	Filtered	Filtered	Filtered	12160.38184
Filtered	2890.626465	5958.708496	5606.597168	6852.916992	Filtered	Filtered	Filtered	Filtered	Filtered	4091.883301	Filtered
10146.85547	9926.172852	6750.154785	Filtered	5502.535156	Filtered	Filtered	4666.537598	Filtered	5114.608887	Filtered	8533.614258
Filtered	4570.487793	Filtered	5783.339355	4852.678711	2921.723389	Filtered	4713.723633	Filtered	3150.789063	3275.016602	3821.662109
Filtered	Filtered	8314.826172	5159.277832	5632.751465	5373.827148	Filtered	Filtered	Filtered	Filtered	Filtered	7190.98291
9167.869141	4808.149414	5770.052734	Filtered	Filtered	8467.746094	Filtered	6966.861328	Filtered	4166.605469	7083.22998	Filtered
Filtered	Filtered	Filtered	Filtered	Filtered	Filtered	Filtered	Filtered	Filtered	Filtered	Filtered	Filtered
Filtered	Filtered	Filtered	Filtered	Filtered	Filtered	Filtered	Filtered	9057.145508	Filtered	Filtered	Filtered
Filtered	5967.740723	Filtered	5290.211426	5923.491211	Filtered	4236.818848	Filtered	2935.729736	Filtered	Filtered	5468.629883
Filtered	5069.777344	4191.71582	Filtered	4261.671387	Filtered	Filtered	Filtered	Filtered	Filtered	Filtered	3584.237305
Filtered	Filtered	8031.436523	Filtered	Filtered	Filtered	6173.898926	Filtered	Filtered	Filtered	Filtered	Filtered
6230.214844	Filtered	4625.178711	7262.183105	Filtered	Filtered	Filtered	Filtered	Filtered	Filtered	Filtered	Filtered
5272.39502	4285.027832	4874.263672	4957.547363	5201.352539	Filtered	Filtered	2925.437256	Filtered	1287.779907	Filtered	1923.913574
5322.810059	Filtered	Filtered	Filtered	4813.185059	6353.804688	9243.425781	8797.000977	Filtered	6193.831055	Filtered	Filtered
Filtered	Filtered	Filtered	Filtered	Filtered	Filtered	Filtered	Filtered	Filtered	Filtered	Filtered	Filtered
7404.529785	7474.12793	7255.037109	7690.203613	6798.231445	Filtered	3115.308105	4151.596191	Filtered	Filtered	Filtered	Filtered
Filtered	6470.991699	Filtered	Filtered	Filtered	Filtered	Filtered	Filtered	Filtered	Filtered	Filtered	Filtered
Filtered	Filtered	6051.527344	Filtered	Filtered	Filtered	Filtered	Filtered	Filtered	Filtered	Filtered	Filtered
4128.458984	6560.669434	3693.829834	4548.50293	Filtered	Filtered	4843.31543	6619.904297	2132.86792	2192.734619	6489.875977	5414.615723
4321.042969	5510.263672	Filtered	4554.455566	5115.390137	3823.692871	Filtered	4662.602539	Filtered	2541.99292	Filtered	Filtered
Filtered	Filtered	4235.345703	4290.240723	Filtered	Filtered	Filtered	8080.590332	Filtered	Filtered	Filtered	7353.300781
1982.6698	Filtered	Filtered	Filtered	Filtered	Filtered	Filtered	Filtered	Filtered	Filtered	Filtered	Filtered
7845.470703	Filtered	3338.762695	5588.07959	3859.023438	3496.984375	Filtered	5793.850586	3397.542725	Filtered	Filtered	4644.425781
Filtered	6428.70166	4881.553223	3919.777832	7379.635254	3494.231445	Filtered	2550.960693	Filtered	9000.363281	Filtered	5876.86084
Filtered	Filtered	3448.159912	Filtered	Filtered	5203.959961	Filtered	Filtered	7181.26123	Filtered	Filtered	Filtered
5180.725586	6608.18457	6356.052734	6435.725586	5068.688477	3920.888428	5128.396484	6295.349609	4035.245117	6018.914063	4556.673828	5439.396484
6378.823242	Filtered	3481.70459	8304.789063	Filtered	Filtered	2913.827637	4848.822266	Filtered	Filtered	Filtered	2304.393555
Filtered	7507.081543	4439.217773	4393.279785	6204.254883	Filtered	1867.06665	Filtered	Filtered	Filtered	Filtered	Filtered
Filtered	Filtered	Filtered	5593.119629	1464.880127	3624.750244	Filtered	Filtered	Filtered	Filtered	Filtered	3224.409668
Filtered	Filtered	6532.561035	Filtered	5196.811035	5737.210938	Filtered	Filtered	Filtered	Filtered	Filtered	Filtered
Filtered	Filtered	4505.172363	Filtered	Filtered	Filtered	Filtered	Filtered	Filtered	Filtered	Filtered	Filtered
Filtered	Filtered	Filtered	Filtered	Filtered	Filtered	Filtered	Filtered	Filtered	Filtered	Filtered	Filtered
Filtered	Filtered	Filtered	Filtered	Filtered	Filtered	Filtered	Filtered	Filtered	Filtered	Filtered	Filtered
Filtered	5716.446777	5695.147461	Filtered	4183.255371	Filtered	Filtered	Filtered	Filtered	Filtered	Filtered	Filtered
Filtered	Filtered	Filtered	Filtered	Filtered	Filtered	Filtered	Filtered	Filtered	Filtered	Filtered	Filtered
2762.972656	Filtered	Filtered	Filtered	Filtered	Filtered	Filtered	4023.224609	Filtered	Filtered	Filtered	4095.287109
Filtered	2999.642578	Filtered	3765.945313	Filtered	Filtered	3919.332275	4140.064453	Filtered	Filtered	Filtered	Filtered
5301.99707	5619.685547	6670.46875	3918.228027	7022.193848	4193.191406	4479.273926	4334.902344	3104.293457	2539.915283	Filtered	5252.883789
9412.120117	11821.02051	2105.974121	Filtered	4926.202148	3271.114258	Filtered	Filtered	Filtered	Filtered	6891.11377	4151.335449
Filtered	Filtered	Filtered	3232.451172	2644.708984	2282.414551	Filtered	Filtered	Filtered	Filtered	Filtered	5218.574219
7758.270508	7527.886719	3543.637207	5206.581543	4833.237793	3289.733154	Filtered	6349.510742	2803.603271	5424.213379	Filtered	6503.590332
5182.449219	7860.480957	4836.353516	2192.71167	6751.359863	6331.287598	7874.416992	4186.635742	1990.752563	4827.110352	Filtered	3740.904785
Filtered	3446.133057	Filtered	Filtered	Filtered	Filtered	3608.585693	5369.095703	Filtered	Filtered	5293.396973	Filtered
Filtered	Filtered	Filtered	Filtered	Filtered	Filtered	Filtered	Filtered	2614.174316	Filtered	Filtered	Filtered
5796.418945	7613.170898	3843.708496	5939.848145	3370.579102	Filtered	Filtered	Filtered	2257.841797	5208.37207	Filtered	4171.960449
Filtered	Filtered	4731.019531	Filtered	5766.131348	5662.668457	Filtered	Filtered	Filtered	Filtered	Filtered	Filtered
6249.167969	4067.2146	5443.333984	4366.036133	4467.018555	5474.606934	3036.450684	Filtered	Filtered	Filtered	3836.669678	Filtered
Filtered	4680.378418	3800.512695	5963.600586	Filtered	3699.488281	Filtered	4206.17334	Filtered	3970.248779	Filtered	Filtered
2641.812256	Filtered	Filtered	Filtered	Filtered	Filtered	Filtered	Filtered	Filtered	Filtered	Filtered	3454.659668
Filtered	Filtered	Filtered	Filtered	3292.719238	2484.02002	Filtered	Filtered	Filtered	Filtered	Filtered	Filtered
Filtered	2522.026611	Filtered	Filtered	Filtered	5731.641113	Filtered	Filtered	Filtered	Filtered	Filtered	Filtered
Filtered	Filtered	Filtered	Filtered	Filtered	Filtered	Filtered	Filtered	Filtered	Filtered	Filtered	Filtered
6730.871094	2366.488525	3467.935791	6120.414063	3940.404785	7412.852539	Filtered	3724.730957	Filtered	Filtered	3109.798828	3117.5271
3903.235107	Filtered	1714.91272	Filtered	2094.477051	Filtered	Filtered	Filtered	Filtered	Filtered	Filtered	4075.314209
Filtered	Filtered	2599.912598	Filtered	Filtered	Filtered	Filtered	Filtered	Filtered	Filtered	2099.587402	Filtered
2865.316162	6696.605957	Filtered	Filtered	4399.007813	Filtered	Filtered	Filtered	Filtered	Filtered	Filtered	3585.200928
Filtered	Filtered	4795.791504	3851.581299	3811.714355	6031.461426	3721.409424	3793.352783	Filtered	Filtered	Filtered	Filtered
4102.104492	4491.935547	4255.036133	4691.875	5332.436523	3197.933838	Filtered	Filtered	Filtered	Filtered	Filtered	Filtered
6114.15625	2993.620117	4606.504883	6385.9375	4214.78418	7100.65918	Filtered	5144.026367	6909.772949	3026.121826	1902.301514	4822.285156
Filtered	Filtered	Filtered	Filtered	Filtered	Filtered	Filtered	Filtered	5284.289063	Filtered	Filtered	Filtered
3543.928711	3855.010254	2907.407471	3929.227295	2742.915771	3516.020996	Filtered	4123.807129	Filtered	Filtered	Filtered	Filtered
5131.739746	5017.528809	Filtered	Filtered	5156.331543	3384.902832	Filtered	Filtered	Filtered	Filtered	2693.204346	3246.154053

Filtered	Filtered	Filtered	3338.830811	Filtered	Filtered	Filtered	Filtered	Filtered	Filtered	Filtered	Filtered
Filtered	Filtered	Filtered	Filtered	Filtered	Filtered	Filtered	Filtered	Filtered	Filtered	Filtered	Filtered
Filtered	Filtered	Filtered	Filtered	6065.772461	Filtered	Filtered	Filtered	Filtered	Filtered	Filtered	Filtered
3751.628418	Filtered	1425.427124	5224.444336	3426.358643	Filtered	3129.116943	Filtered	Filtered	Filtered	Filtered	Filtered
Filtered	Filtered	Filtered	Filtered	Filtered	Filtered	Filtered	Filtered	4851.502441	Filtered	Filtered	Filtered
Filtered	Filtered	5065.260254	Filtered	Filtered	Filtered	Filtered	Filtered	Filtered	Filtered	Filtered	Filtered
Filtered	Filtered	Filtered	Filtered	Filtered	Filtered	Filtered	Filtered	Filtered	Filtered	Filtered	Filtered
Filtered	Filtered	Filtered	Filtered	3140.178955	1943.93335	Filtered	Filtered	Filtered	Filtered	Filtered	Filtered
5222.922363	Filtered	4572.285156	Filtered	3750.044922	Filtered	Filtered	3965.790527	Filtered	Filtered	Filtered	5046.995117
Filtered	3828.077637	Filtered	3025.359375	Filtered	Filtered	6134.428711	Filtered	Filtered	4121.463379	3419.805176	Filtered
Filtered	Filtered	1883.945801	24047.94727	2806.031006	Filtered	Filtered	Filtered	Filtered	Filtered	Filtered	Filtered
Filtered	Filtered	Filtered	Filtered	Filtered	Filtered	Filtered	Filtered	Filtered	Filtered	Filtered	Filtered
4104.79541	3646.539795	2971.833984	4431.61084	2495.900879	2154.840332	Filtered	5432.089355	Filtered	Filtered	Filtered	Filtered
Filtered	Filtered	3881.630859	3609.401367	Filtered	Filtered	Filtered	Filtered	Filtered	Filtered	1776.481934	Filtered
Filtered	3506.436279	Filtered	Filtered	Filtered	Filtered	Filtered	Filtered	Filtered	8341.208984	Filtered	Filtered
Filtered	Filtered	Filtered	Filtered	Filtered	Filtered	Filtered	Filtered	2659.598389	Filtered	Filtered	Filtered
4471.213867	3113.606445	4132.906738	4947.038574	4075.883057	4461.34082	Filtered	3210.919189	Filtered	Filtered	Filtered	Filtered
Filtered	Filtered	4445.414063	Filtered	Filtered	Filtered	Filtered	Filtered	Filtered	Filtered	Filtered	Filtered
Filtered	Filtered	Filtered	Filtered	Filtered	Filtered	Filtered	Filtered	Filtered	Filtered	Filtered	Filtered
Filtered	Filtered	3680.086426	Filtered	3867.970703	Filtered	Filtered	Filtered	Filtered	Filtered	Filtered	Filtered
Filtered	Filtered	1670.063232	5452.742188	Filtered	Filtered	Filtered	3748.430908	Filtered	1879.867676	Filtered	Filtered
4627.007324	3913.319824	4694.70166	3563.854492	4482.361816	3892.976563	1946.784302	2081.539063	Filtered	2859.576172	Filtered	Filtered
3722.292725	4272.49707	4881.952637	4349.794434	3962.115723	3735.210693	Filtered	3619.728271	Filtered	2895.102295	2713.333252	3091.167969
Filtered	Filtered	Filtered	Filtered	Filtered	Filtered	Filtered	Filtered	Filtered	Filtered	Filtered	Filtered
Filtered	Filtered	Filtered	Filtered	Filtered	Filtered	Filtered	Filtered	Filtered	Filtered	Filtered	Filtered
2309.610107	3856.223877	4372.242676	3912.327881	4533.446777	4260.040039	Filtered	Filtered	Filtered	3888.672607	Filtered	2453.664063
Filtered	Filtered	Filtered	3080.276855	3484.069336	5657.300781	Filtered	Filtered	Filtered	Filtered	Filtered	Filtered
Filtered	Filtered	Filtered	Filtered	Filtered	Filtered	Filtered	Filtered	Filtered	Filtered	Filtered	Filtered
Filtered	Filtered	Filtered	Filtered	Filtered	Filtered	Filtered	Filtered	Filtered	Filtered	Filtered	Filtered
3894.075195	3543.303955	6160.002441	1945.235474	3708.319824	4567.275391	2436.314697	2887.445068	3492.872314	1835.554077	Filtered	Filtered
Filtered	Filtered	2562.708496	Filtered	4545.415039	Filtered	Filtered	Filtered	Filtered	Filtered	Filtered	Filtered
4086.489014	Filtered	3227.11377	4099.129883	3289.678955	3291.826416	Filtered	Filtered	Filtered	Filtered	Filtered	Filtered
Filtered	3398.966064	3466.181885	Filtered	3410.812256	2349.288574	3827.477783	Filtered	Filtered	Filtered	2064.056152	2188.521729
Filtered	Filtered	2117.238037	Filtered	2821.694824	Filtered	Filtered	Filtered	Filtered	Filtered	Filtered	Filtered
2161.524414	1408.069092	2012.09375	2084.596436	3683.570801	2945.974854	1252.267944	Filtered	Filtered	Filtered	Filtered	1236.340454
Filtered	Filtered	Filtered	Filtered	Filtered	Filtered	Filtered	Filtered	Filtered	Filtered	Filtered	Filtered
Filtered	Filtered	Filtered	Filtered	Filtered	Filtered	Filtered	Filtered	Filtered	Filtered	Filtered	Filtered
Filtered	Filtered	Filtered	Filtered	2973.790283	Filtered	Filtered	Filtered	Filtered	Filtered	Filtered	Filtered
Filtered	Filtered	Filtered	Filtered	Filtered	Filtered	Filtered	Filtered	Filtered	Filtered	Filtered	Filtered
Filtered	Filtered	Filtered	3312.137207	Filtered	Filtered	Filtered	Filtered	Filtered	Filtered	Filtered	Filtered
Filtered	Filtered	Filtered	4342.017578	Filtered	Filtered	Filtered	Filtered	Filtered	Filtered	Filtered	Filtered
2627.855225	Filtered	1840.489868	3864.489258	3366.149902	Filtered	Filtered	4555.258301	Filtered	Filtered	Filtered	Filtered
Filtered	Filtered	Filtered	Filtered	3263.265381	Filtered	Filtered	Filtered	Filtered	Filtered	Filtered	Filtered
Filtered	Filtered	Filtered	Filtered	Filtered	Filtered	Filtered	Filtered	Filtered	Filtered	Filtered	Filtered
Filtered	Filtered	Filtered	Filtered	Filtered	Filtered	Filtered	Filtered	Filtered	Filtered	Filtered	Filtered
Filtered	Filtered	1270.981079	Filtered	Filtered	Filtered	Filtered	Filtered	Filtered	Filtered	Filtered	Filtered
3059.559326	Filtered	Filtered	Filtered	Filtered	Filtered	Filtered	Filtered	1306.702881	Filtered	Filtered	Filtered
Filtered	Filtered	Filtered	Filtered	Filtered	Filtered	Filtered	Filtered	Filtered	Filtered	Filtered	Filtered
1105.374512	Filtered	Filtered	Filtered	Filtered	Filtered	Filtered	Filtered	Filtered	Filtered	Filtered	Filtered
Filtered	Filtered	Filtered	3432.788818	Filtered	599.0800781	Filtered	Filtered	Filtered	Filtered	Filtered	Filtered
Filtered	Filtered	Filtered	Filtered	Filtered	Filtered	Filtered	Filtered	Filtered	Filtered	4122.853516	Filtered
Filtered	Filtered	2708.743164	1802.691772	Filtered	Filtered	1390.475098	1940.153198	Filtered	Filtered	Filtered	Filtered
Filtered	2813.07666	2464.496338	Filtered	1633.360107	1400.636963	Filtered	Filtered	Filtered	Filtered	Filtered	Filtered
Filtered	Filtered	1512.1427	3219.440918	899.2863159	Filtered	Filtered	1023.541443	Filtered	Filtered	Filtered	Filtered
Filtered	Filtered	Filtered	Filtered	Filtered	Filtered	Filtered	Filtered	Filtered	Filtered	Filtered	Filtered
Filtered	Filtered	Filtered	Filtered	Filtered	Filtered	Filtered	Filtered	Filtered	Filtered	Filtered	Filtered
Filtered	Filtered	Filtered	Filtered	Filtered	Filtered	Filtered	Filtered	Filtered	Filtered	Filtered	Filtered
Filtered	Filtered	Filtered	Filtered	Filtered	Filtered	Filtered	Filtered	Filtered	Filtered	Filtered	Filtered
Filtered	Filtered	2740.714111	Filtered	Filtered	Filtered	Filtered	Filtered	Filtered	Filtered	Filtered	Filtered
Filtered	Filtered	Filtered	Filtered	Filtered	Filtered	Filtered	Filtered	Filtered	Filtered	Filtered	Filtered
Filtered	Filtered	Filtered	Filtered	Filtered	Filtered	Filtered	Filtered	Filtered	Filtered	Filtered	Filtered

PCOS_1	PCOS_2	PCOS_3	PCOS_4	PCOS_5	PCOS_6	PCOS_7	PCOS_8	PCOS_9	PCOS_10	PCOS_11	PCOS_12
73644264	156787600	65054816	84853568	128435856	132461864	224841536	51715556	58874312	113018584	83225360	66592364
104650792	117463336	61991852	65888736	100583216	89082256	106856272	91545136	102340552	98428776	68254184	119451936
26656140	29553752	29767256	25874978	29553406	39090380	26641470	34995884	32700074	21069532	25252960	25233810
25423922	23860366	18966322	28788604	26024682	33350860	24711516	21310952	25454012	15784453	18378988	18669494
19801154	22311562	18294300	16128967	17493190	23820532	17477254	19769276	20087308	13497461	12508105	19923954
22467210	18515332	20145054	18637702	19785758	21313476	17464530	23692136	24426694	17652934	17704442	24799166
16950804	15561743	22794116	16624835	16918984	20290606	16841124	22001398	20549022	8904154	17788408	18842052
13520557	17280356	14875904	11254067	15924502	13361698	14778464	12574393	14945699	11899001	11933409	18757636
13805350	14901358	15075664	10369368	13776324	14224258	12992652	16123715	13469966	9964142	11389718	13011137
14840703	14414446	14163142	14395538	15219183	13118936	11715799	13343169	14458857	11044524	11794563	13790655
10875533	12812063	10442465	8829392	10828580	10764312	10420650	13473688	10737163	9730754	8307864. 5	10098661
13035660	7547138	8711136	8039653	9819277	6067733. 5	3657285. 5	15757323	12926073	33437686	12446476	14672011
9467692	11029941	9651976	10211515	9944227	10476468	7625702	9552404	10714696	7336483. 5	7997652. 5	11045605
9035036	7075207. 5	10822206	8365484. 5	8968340	11888713	7972280. 5	9936985	10621409	6290436	7288934	8914773
9065511	13123916	11300329	7475061	10780763	11217312	6177373. 5	12433183	7402643. 5	8100784. 5	11406286	8828020
9024505	9384452	8713789	6272560	9684162	12207081	6620030. 5	10458400	9273549	4266772. 5	6356626	6099821
8101239	8541777	8870437	7799133	9723101	11441908	8055155	9598375	8401437	5909877	7409380. 5	9225757
7307300	6990472	10265608	9614562	8061282. 5	9570529	8693682	9452351	9758399	5308655	8300318. 5	9013378
7983486	6310224. 5	9785396	8059304	8402664	11796171	7111318. 5	9274944	9569252	6097352. 5	6223704	7599608. 5
33983. 30469	112865. 6875	16378304	17612502	67618. 95313	54954. 67188	131972. 2813	10865786	9859669	3305156	32502. 625	14787222
7922059	6958583. 5	5277292. 5	4464576	4716482	7060579	7569556. 5	7089626	8155894. 5	5417431	8914553	7158866
6716496. 5	7096736	6414909	5346876. 5	7019847. 5	9303596	7023964	6637179	6461655	4447946	5230280	5312395
7985184. 5	33251. 59766	6473219	6327833	8145228	7462829. 5	5887103	9012056	6359145	6865775. 5	8765744	2711698. 75
5749787	5592866. 5	6823788. 5	4949463	4903243. 5	6321717. 5	5233648	16076978	6690035. 5	10467454	6709905	6161032
5915635. 5	7079795. 5	7060794. 5	6402723. 5	6334165. 5	7062256. 5	6392046. 5	7244046	7921931	7562972. 5	6022346	7282554. 5
6565507	6584843	7373387	4933162	6506784	7466293	6370385	6698607	6602735	3903795. 5	5557783	6880983. 5
5006792	6168644	5350346	4523238. 5	5441339	8344319	5799716. 5	6361262	6958039. 5	4910012	5001634	6203712. 5
5219981. 5	5315101	5968236	4870230. 5	4880415	6489351. 5	4733908	6710729	5894345	8500516	4990166. 5	5529067
6363683	5815355	5199315. 5	8023877	7219776	9532229	5376283. 5	6976242	6647737. 5	5501331	4190900	7551542. 5
4207845. 5	5886864. 5	3718885. 5	4690535. 5	3988297. 25	5148804. 5	4584304. 5	5526989	3980397. 5	2120601. 5	4190973	6358668
6240254	4235292	4460763	2696597. 75	4029181. 25	5936158. 5	5137076. 5	5082411. 5	7726267	4336789. 5	3536583. 25	5316930. 5
4821298	5325220	5050160	4089133	4101794	6936461. 5	3578420	6622976	6198009. 5	3584855. 75	4279519. 5	5332318. 5
4644731. 5	4742756	5745006	4578164. 5	5393083	6401919	4349953. 5	5092745. 5	4310087	4112143. 25	4186015. 5	4538285
4971148. 5	3588589. 5	5256579	3950009. 5	4213280. 5	3088948. 5	5075755. 5	5014750	5424069	3527246. 25	5582382	5362845
4159952. 25	5496677	2550632. 25	3446803. 25	6744616	4665522. 5	3439005. 25	3808628	3458300. 25	3561002. 5	3668911. 75	4845867. 5
2139641. 75	4561556	1475203. 75	2463673. 25	3023038. 5	4747432	9344869	1694498. 125	935439. 375	3485211. 5	3100029. 5	2552741. 5
3005105. 25	3108181. 75	2273086. 25	2457015. 25	3127243. 25	4307095	3393812. 75	2820751. 75	3278247. 25	2741451. 75	3311627. 5	4048150. 5
3851414	2838588	3358919. 5	3167351. 25	3150560. 75	3156123. 5	3860914. 25	4626842. 5	4923463. 5	4767951	3083217. 75	4444488
3591838. 75	3684020. 75	3620252. 5	2821609. 5	3147300. 25	4085893. 25	3300648	3480266. 5	3888417. 75	1912975. 375	3228108	3197731
14581599	Filtered	13711. 4668	3139. 302246	13034507	10670. 72949	2780. 614258	3590. 425293	Filtered	Filtered	1943. 193115	11573. 89648
3350256. 25	3761957	4558712	3751223	4819945	4017. 613525	2000432. 5	5040770. 5	5283316. 5	Filtered	1958667. 25	3078795. 5
3618412. 5	3746006. 75	2961275	2280492. 25	3312061	3199806. 25	4089484. 75	3292199. 25	4051392	2645803. 75	2509550. 75	4605013. 5
3422173. 75	2930323. 5	2778223. 25	3595918. 75	3116856. 5	3151535	2756885. 5	3590944. 25	3886086. 5	2492067	3073849. 75	2729488. 75
3600152. 25	3397779. 5	1048865. 25	4558577. 5	3785503. 75	4638214. 5	4094227. 75	4029600. 25	3674154. 5	3357466	3930215	907382. 9375
3291551. 75	3397115. 5	2764127. 5	1862219. 5	2531697	3821931. 5	2377503. 5	3152699. 25	3209607. 25	1392770	2116310. 5	2880393. 25
2926972. 75	4212268	2275661. 5	2996125. 5	3700723	4915197	2754670	3380748. 75	3466230. 25	1062524	1987472	3185397. 5
2771324. 25	3295488. 25	2301589	3082548. 5	3584654. 5	4519819. 5	2308073	3124031. 5	3438612. 75	1382809	1850244. 875	2567213. 5
1830724. 625	1852014. 5	1411863. 625	2578101	2121222	1536461. 25	3207426. 75	1674640. 875	1648517. 25	1607339. 625	1605953. 5	1725687. 25
3606325	2156548. 75	2954406. 5	2242542. 25	1989540. 75	1353665. 5	2366641. 25	2596994. 75	3568403. 5	2485494. 25	3885114. 75	3444454
2602219. 5	2984569. 25	3871552. 75	3393305	2237484. 5	3518718	2690446. 5	3268151. 25	2572656. 5	1602780. 875	2493943	2517605. 75
2923883	2690666. 75	2667221. 75	2213629	2650093	3221846. 25	2265607. 5	2611402. 75	3051578	1863346. 375	2115949. 5	2995979. 75
2291651. 5	2330728. 25	2732899. 75	1709509. 125	2415438. 75	2677206. 5	2856965. 5	2783221. 25	3028765. 75	1973405. 5	2285535. 5	3452669
2874140. 75	2494189	2430352. 5	1955968. 875	2216409. 75	2344897. 5	2267323. 5	1595351. 375	2466491	1687405. 125	1398147. 625	2203032. 5
2164141	2682072	1521448. 625	1967838	2783751	1794514. 125	6613079. 5	2312094	2816181	1985972. 75	2439107	2985360. 75
2535822. 5	2404138. 25	2086793. 75	2822324. 25	2500135. 75	2310305	2141657. 5	2229909. 5	2116633	2731942. 5	2287785. 75	1880705. 5
2141199. 5	1556317	2076308. 75	2098153. 75	2060269. 25	2456188. 5	1830211. 375	2396518. 5	2724784. 25	1556643. 5	2200382	1328209
2103654. 25	1746752. 375	2836691. 25	1831513. 625	1886523. 5	2569236. 25	1902327. 75	2717402	1651354. 875	1885800. 25	1813815. 375	2477223. 75
1975394. 875	1644517. 75	2335391. 25	1636504. 125	1891580. 875	2299245. 5	1856709. 75	2240631	2278078. 25	1801480. 625	1771050. 25	2059994. 125
2025250. 125	2739413. 25	1945715. 625	2287214. 5	2306292. 5	2638226	1768877. 375	1657822. 125	2326166. 75	1206607. 875	1783004. 375	2271499. 5
1803128. 5	1747910. 375	2740194. 5	1890836	1785864. 25	2349328. 75	1604265. 5	2787643. 5	2147210. 5	1820931. 625	1601339. 5	1748833. 125
2625752. 25	1251644. 75	1480407. 375	1149978. 875	1078420. 625	1847327. 75	2121529. 75	1890531. 875	2356188	1772287. 125	2734931. 25	2116698. 25
1556796	1935301. 625	1427462	2300743. 75	2741599. 5	1105440. 125	3884890. 25	1905528. 25	1231241. 75	1806364. 25	1550773	1905239. 25
1720704. 875	2028779. 75	2101777	2027417. 875	1852388	1777428. 875	1803355. 25	2121392. 5	2038632. 875	1800807. 375	1888310. 5	1818835. 375
1938425. 375	988153. 25	1296171. 875	2647873. 5	3341934	598582. 0625	7473695. 5	701136. 3125	699847. 0625	1353305. 25	1716880	1793541
Filtered	Filtered	Filtered	1513303. 375	Filtered	Filtered	2221514. 5	Filtered	Filtered	Filtered	Filtered	Filtered
1568139. 875	1853634. 5	3015322. 75	1527698. 75	1763664. 875	1953740. 25	1269913. 375	1835576. 25	1842209. 5	1755260. 5	2031713. 75	2125425. 5
1671327. 875	1773969. 875	2246220	1263187. 375	1628057. 625	1775028. 75	1918182. 25	1783018. 375	1945112. 125	1582365. 375	1416488. 625	1728732
1401446. 375	1543192. 375	1537767. 375	2468769. 25	1751863. 125	1897996. 5	1799378. 875	1039490. 188	1544883. 75	1661442. 5	1998583. 375	2522172. 75
2320632. 25	2310068. 25	840317. 5	1510657. 625	2127447. 5	1937857. 125	1614929. 625	1708266. 25	1725288	2353502. 25	1772480. 25	1177155. 625
1417933. 375	1571813. 125	2286227. 5	1583136	1652405	1692380. 75	1157797. 375	1684885. 625	1525526. 25	1434130	1450194. 25	1978610. 875
1746962	1821245. 875	1971067. 375	1554202. 875	1324459. 25	2219107	1336903	1081358. 25	1645592. 125	781329. 625	1457644. 75	1118346. 75
2278875	1862860	706197. 0625	1407332. 5	1806955. 125	3525241. 25	759840. 625	2849241. 5	1119697. 125	1717281. 125	1796322. 625	272315. 3125
1517227. 5	1195333. 125	1660012. 375	1398191. 875	1535016. 375	2113638	1466102	1780049. 25	1573821. 125	1231818. 625	1233231	1298186. 25

1941317.375	1170377.625	1704529.75	1358734.375	1522605	2157060.5	1655940.125	905259.75	2767583.75	1516394.25	1329481.875	1407995.25
1157571.625	1429444.75	820061.5	1071348	1285558.125	818283.4375	3020848.25	1336397	1517875.25	1465154.375	2346041	1239691.625
1369309.5	1658597.75	1531464	1267453.25	1402665.25	1567889.625	1119589.375	1805773.125	1511816.125	1344013.375	1548449	1534000.5
1817262.125	1802684.5	1185815.125	943303.4375	1011667.813	1105843.5	891815.3125	1897965	1283594.25	773099.9375	1020491.875	1852726.125
1485499.875	1717012.75	1963108.875	2301983.5	1546960.75	1594659.375	1334988.125	1331824.125	1634014.625	687984.875	1396812.25	1433791.375
Filtered	2485859.5	1519662	Filtered	Filtered	2122336.75	Filtered	Filtered	Filtered	2330175.75	1276761.75	981805.75
1131180.25	1371715.875	1567881.75	1488133.75	1375915.25	1413636.25	1354851.375	1521487.25	1518212	1246782.125	1312960.25	1351510.25
1103253.75	1484460.375	978793.1875	1395887.875	1246582.75	1304566.375	1380703.625	1524482.875	1018069.125	1108039.75	1108110	1743159.5
1609094.5	1844327.75	1483943.5	133887.625	1383420.75	2071889.375	1271155.25	2067306.25	2364131.25	1666833.625	1681413.75	674844.0625
1531151.75	1259108.125	1334733.5	1028468.438	1300705.75	1611613	1537609.75	1300149.5	1308678.375	1130470.375	1125223.875	1236981
1532933.125	1350743	377321	1651876.25	1173924.125	1692122.75	1553314.75	1217090	1724250.5	1052244.25	1048571	1464988.375
655902.6875	1941749.125	1337231.125	848560.5	2829579.25	671490.8125	7067679	261537.3438	766235	841691.75	981769.625	1151096.375
1231395.25	1357358.75	962846.875	920337.125	1168938.5	1357417.125	1272962.25	1228713.75	1324171.5	1093856	896415.625	1025826.25
933705.3125	1171075.125	1081133	1237633.5	1111692.75	1347810.125	1129260.625	1183315.875	1002202.063	687217.25	900581.875	926220.8125
1154649	1156042.375	804812.375	931406.4375	1099752.5	1046430.313	1144211.75	1025951.5	1342914.875	940456.5625	1250720.625	1090281
1105803.75	892907.9375	1450667.5	1022897.938	1001794	1192985.875	1099959.75	1661368.5	1314252.25	1224394.75	1463381.25	1348927
968670.875	797942.5	912104.125	1276571.625	805663.875	1193187.125	944703.875	1066997.75	1359357.875	1298408.5	1242000.5	1262207.125
1309455.25	1186632.5	1073063.25	1268066.5	886565.4375	1413283.5	1240007.125	1403437.625	1416465.25	1049302.375	1149323.5	987268.8125
1384967	380340.8125	211335.5	805010.625	938030.75	462431.4063	704228.375	2746980.75	744053.25	7153981	1935486.25	510584.7188
1148183.125	1020715.375	1214506.875	1212511.5	1204976.375	1127587	1057547.125	1099090.625	1236558.75	905247.1875	990801.625	1028308.313
90120.98438	74954.46094	106347.2266	1764248.375	138842.1406	151007.5781	115545.1328	1018686.688	9522.061523	2654385.75	114166.3672	1440536.375
1178628.375	877902.3125	1181092.5	978048.375	904566.5	1163713.5	954402.125	1177896.75	1238419.25	852383.0625	818109	890120.8125
271748.3438	190457.7344	181612.4688	490313.4375	348204.7813	200285.7188	2326088.5	182967.8125	353164.6563	282773.7813	192363.3281	256285.5469
1101314.5	1225023.25	1465437.5	697067.0625	1257218.125	899515.8125	959196.6875	1063461.25	885659.1875	626332.875	994731.875	950939.9375
986833.6875	1146424.25	1180429.25	1428689.5	1114124	1246303.75	789501.8125	1068105.5	1238809.25	746651.5625	964629.9375	1165131.25
849763.375	951643.5625	1142055	1217886	1020756.313	1300640.5	763672.375	1017770.813	934269.4375	633551.625	794385.4375	918402.4375
435294.75	1126446.875	453776.6563	713636.875	1113769.375	1283831.125	2274543	288964	327225.875	646264.6875	413819.5	429124.1563
575945.9375	766586.4375	2543979.5	588215.6875	847813.8125	1855675.5	1758956.375	847014.5	1081226.875	1635299.5	913752.375	724881.4375
1027138.938	598404.125	1050141.125	799210.9375	896632.8125	1007772.875	839054.9375	1012360.438	961012.8125	461107.8125	766593.8125	831513.6875
402693.7188	1430790.75	956962	547060.8125	2011235.875	383735.9063	3646489	233267.3281	448453.2813	761570.1875	752950	805975.625
658688.125	689096.8125	595353	626002.625	673283.5	1030208.063	608864.125	580489.25	779332.25	711873.625	671089.1875	802901.75
1007795.188	720500.125	743260.5	809472.5625	809229.875	994131.875	809522.625	537981.375	678549.5625	636315.125	660103.875	953030
720615.1875	Filtered	Filtered	634368.625	680846.1875	847724.25	Filtered	817160.75	Filtered	Filtered	1014452.063	Filtered
607228.6875	278089.1875	282791.1563	255059.7656	534802.25	232585.1563	2321834.75	472975.375	630001.0625	1105419.625	467610	382889.4375
560729.625	777471.75	861468.6875	821073	732937.9375	703494.9375	667066.9375	697241	677233.6875	572128.75	635521.4375	904505.9375
712935.75	622626.375	469285.5938	770717.625	809281.25	696224.0625	599650.9375	652075.4375	818306.3125	824911.5	638865.5625	435225.1563
679861.25	710161.5	595617.125	456061.5625	611930.1875	824258.0625	535196.25	632425	685673.75	416684.9688	519351.4375	578824
140971.5	102934.0703	114971.0859	247497.4844	204015.125	103945.3125	1582993.75	104136.6953	235360.3594	189183.125	101284.625	163828.4688
580455.5	627928.5625	737955.3125	734563.625	584194.3125	624354.0625	518430.625	630276.1875	640869.25	487327.3438	546450.5625	635309.8125
628996.3125	Filtered	Filtered	798913.875	Filtered	854158.5	Filtered	Filtered	617428.8125	Filtered	Filtered	584256.5625
526083.125	449601.875	683420.6875	552244.3125	492822.125	712930.875	471947.9688	568010	582410.625	606212.0625	440145.4688	528864.1875
592886.375	532402.625	578690	517983.9063	482498.3125	676187.1875	461777.6875	661197.5	654219.5625	540722.875	462176.6875	630092.4375
583841.5	514740.5625	469117.125	461783.2188	474739.5938	524296.3125	478712.5625	579833.25	642296.375	536425.375	480893.2813	551390.25
501545.8438	493883.625	394656.5625	528397.6875	1187357.625	198482.4063	2071131	377022	404114.6875	853085	558989.1875	483124.4063
585262.625	586202.8125	190383.5	870388.8125	635322	93929.02344	623413.75	169419.8281	698031.625	820457.4375	777846.5	797074.75
618463.125	446296.7188	600749.875	695460.375	468547.0313	611599	529618.0625	496696.5313	638967.25	490251.4375	499959	726116.875
263342.7813	268344.25	369432.9375	629769.25	1076259.375	281498.5313	1542265.75	243100.9844	343025.5313	454145.1563	651727.875	233727.8125
350257.875	524409.5	354674.9063	753001.875	942169.4375	345705.9375	1541922	155193.6719	333510.5	592542.875	728441.625	432248.6875
677007.9375	467149.375	496824.5	369591.8125	323071.75	167873.9844	553943.625	403245.6875	392809.5	431853.6563	642621.8125	430621.625
481564.5	371572.6563	265911.0625	457428.5938	940687.8125	126972.0938	1297978.875	410604.1563	591256.5625	650095.5	449629.875	536096
381086.8438	482276.25	432973.125	549538.25	413916.6563	689029.6875	474690.3125	512578.625	514046.8438	387557	518949.3438	482313.8125
376006.1563	436629.9688	418592.0625	393881.9688	465034.8438	515979.125	352650.875	447701.6875	428350.5938	594524.25	351088.6563	429500.2813
341895.7188	343175.75	463407.2188	537422.4375	374156.5	639744.375	408795.9688	219535.7969	432885.9375	312713.5938	209048.25	348389.5
519094.5	529119.4375	414832	394673.9063	442794.8125	503710.8125	342562.5625	688834.25	637889.8125	439409.0313	369021.1563	536333.25
54145.5	535794	800458.8125	55382.05859	708483.4375	51047.63281	502381.3125	1023060.75	27101.43359	2152772.75	814818.4375	733882.625
532442	612220.1875	127173.1172	456138	550418.8125	738197.75	300130.2813	673703.5625	362451.6563	380005.1875	580572.25	351175.625
346962.1875	270991.5938	276920.9375	305276.9063	331735.2188	301698.1875	251494.9063	368177.25	337654.7813	6491600.5	389469.5313	353646.7188
393507.4688	390161.25	472899.75	415706.3438	431728.5938	600950.875	436591.2188	382245.5938	468415.375	363362.0625	402230.5313	408526.75
457764.5625	444348.7188	480652.8438	467986.3438	482566.1563	507622.5313	377269.2188	449187.5313	411494.4375	392216.9063	404677.8125	543607.8125
562728.1875	353216.1563	1404429.375	537237.8125	314252.0313	595387.25	531361.3125	504955.3125	394981.7188	983293.375	12033.22852	649231.5625
311673.9688	328472.3438	502923.9375	262802.0625	330274.7188	305029.0313	279504.2813	461029.25	326495.4688	577185.3125	604302.9375	279669.8125
517606.5625	435759	277993.625	269296.1563	195305.9375	140694.7813	330138.9688	428688.2813	448929.6563	558136.625	450889.625	282218.5
235060.7031	153986.375	249161.3906	182283.9375	269178.4063	342513.5938	107762.8281	297177.3125	3920930.75	228365.9844	225078.625	269574.2188
421235.25	405194.5313	370286.125	296682.0938	313367	437961.9375	414232.5938	387914.5938	314539.0625	323752.1563	349811.3438	410723.25
Filtered	Filtered	118080.3672	Filtered	Filtered	Filtered	Filtered	Filtered	Filtered	Filtered	Filtered	1319653.625
350308.4063	334323.625	307982.0625	454316.4688	312593.4063	330352.0625	292895.0625	378805.8438	440391.5625	281791.9063	286617.2188	435016.2813
354744.875	441050.375	380237.0625	301535.3438	370715.3125	351906.4063	261224.5156	332860.0938	381710.9063	294026.375	324568.6875	341575.2188
298986.75	220774.1406	217879.4688	226935.0469	225271.875	138715.375	165330.2813	237328.7344	197243.9375	1623839.625	285531.1875	270379.2188
198001.7344	173709.2656	148272.3906	124917.9609	463769.2188	466055.625	181296.6563	179273.5313	183549.9531	185883.875	166300.2188	169328.4688
352113.75	364503.4375	302082.3125	373393.9688	313101.3125	436453.0313	311104.5938	426746.8125	391519.625	320545.4375	182653.8438	305138.6563
590469.125	451172.875	656926	349814.1875	351643.2813	366546.375	157265.4844	543441.375	351120.6563	384292.8125	470152.1563	441369.5938
328029.9688	444199.9375	286122.4375	387870.4688	380526.4375	448542.0625	309196.2188	235603.7813	363756.1563	Filtered	257957.5156	343863.4688
342817.9063	209989.5625	305133.9063	234547.3594	257587.9375	330472.25	205398.375	361783.2813	130092.4766	245214.5781	360720.875	375866.4375
Filtered	Filtered	Filtered	Filtered	Filtered	Filtered	Filtered	Filtered	Filtered	Filtered	Filtered	Filtered
288090.1875	316086.625	328666.5	238689.5313	326399.2188	382061.1563	357745.2188	306814.3125	324583.25	253735.1094	249634.1563	307295.1875

86563.33594	128182.0547	574682.3125	135947.75	310022.375	12977.88574	372166.5	494717.4375	143868.6875	212343.2031	302532.1563	315095.8438
Filtered	342465.7188	Filtered	164734.6406	229618.3438	294799.2188	Filtered	575385.4375	Filtered	Filtered	Filtered	527903.875
319454.7188	324000.125	257835.0469	336415.0625	329828.0313	308097.8125	258546.8906	268608.0625	261293.5	171311.2813	265237	412598.5938
322672.1875	217011.0156	172840.125	364206.2188	509100.9688	143793.0313	728449.375	151614.2031	158818.5	198816.3438	265961.7188	397878.6875
182863.0781	59972.63281	268087.0313	249166.6406	149230.3906	48225.46094	67199.35938	119383.8672	100300.7891	1553708.625	73550.21875	399645.625
Filtered	229244.2813	90376.13281	78319.84375	148501.5781	10520.70801	319515.6563	Filtered	17568.5	Filtered	9651.557617	Filtered
246910.2813	235620.2969	251086.0313	355640.4688	626904.9375	144586.1719	586066.3125	139105.7188	192620.7813	291377.25	327677.375	418905.0938
Filtered	Filtered	Filtered	Filtered	Filtered	Filtered	Filtered	Filtered	Filtered	Filtered	Filtered	2135474.75
Filtered	20050.87891	Filtered	Filtered	Filtered	Filtered	Filtered	Filtered	467811.375	79803.92188	Filtered	Filtered
310015.375	233716.4219	242722.25	174544.75	230070.2344	340091.9688	234073.0938	326513.875	321920.3125	211224.9531	231406.2656	267429.0313
307778.125	277350.5938	311410.9688	238924.9531	396080.5313	318743.9063	196239.375	244448.7344	270266.625	207600.8281	212094.1563	383724.4063
386194.5313	189624.9219	291670.125	161521.4375	220329.125	444141.625	361890.6563	189002.9375	451423.1875	179763.2188	226586.7031	226813.6094
234978.1719	179844.6094	255495.7344	343443.5938	317337.8125	423293.0313	216869.9375	313572.1875	384508.9375	229270.9531	311598	312661.875
Filtered	203315.3125	Filtered	Filtered	Filtered	239017.6094	Filtered	154709.8281	291618.4063	Filtered	280971.4375	Filtered
216040.7969	179009.7656	248534.7344	62475.25781	180304.6563	93245.13281	185658.0156	256087.875	270014.0938	3037701.25	253546.8125	250484.6719
246056.5469	235202.4531	250841.4063	187152.7344	615297.6875	143761.2656	569418.3125	138595.1406	192002.0938	291381.0625	323656.3438	416775.4063
215613.6094	249572.6094	313990.1563	235468.7344	196966.5	306557.9063	231056.8594	215280	333143.1875	191250.2969	248214.9844	245504.7813
194458.2813	41521.64063	169500.25	127427.1953	140259.8281	57164.80859	71158.39844	68729.42188	99720.39844	1070459.75	68729.30469	224097.4688
367080.375	381247.8125	152895.0938	126431.6484	253571.0625	305927.9688	227611.6406	281880.5938	217603.3281	258246.5781	212663.9688	255028.1563
228303.6563	232317.5313	302043.375	187363.7656	188492.8594	288435.3438	225279.5	291654	307258.5625	237785.4688	227529.5938	268126.5313
263289.25	327452.125	152428.8594	313666.1875	272294.0938	405439.625	161890.5625	391230.5938	203856.2188	331046.5938	342763.875	159355.5625
213757.0625	140085.1719	89359.63281	161883.3594	184232.8125	43159.15625	626696.875	256994.0781	380223.5	268353.4688	243948.0781	160974.7813
393250.8125	156887.3125	319528.25	172888.5	315453.5313	217803.5781	263035.9375	254826.6875	323442.625	309993.5938	303538.9375	265346.0938
199263.3125	219324.1719	307985.1563	281063.375	236665.3906	316094.4063	196433.625	250924.0313	272034.9063	224064.625	190657.6563	237106.4531
205588.4531	221957.9688	208913.2969	198421.0313	232599.0469	162245.9531	186758.4219	256171.25	195304.9219	144006.625	324353.125	207037.7969
Filtered	308895.9375	Filtered	214484.1875	Filtered	Filtered	344347.8125	176154.9844	296482.1875	Filtered	112819.2813	277704.6875
262600.8125	148184.3906	307375.7188	196490.6875	184588.3125	271821.625	66027.29688	283198	182328	215376.7969	187307.9219	260726
386320.4688	201603.9531	275463	220363.6563	97063.04688	190941.2344	102356.1094	559141.875	212859.5938	313374.25	194634.8438	228974.1719
238694.4531	167837.4063	224620.2813	205055.7031	198991.8906	241574.0625	214261.6406	251425.7813	249494.5469	204751.7344	213698.4844	234518.6719
311116.875	246570.6094	179863.8125	153637.875	154032.8125	244128.1406	21867.68359	255683.3594	310118.9063	691306.625	299693.75	231333.4063
127791.2188	150266.25	114640.7969	145851.8906	176161.2188	324936.8438	109823.2188	148893.7813	142262.7031	116814.2109	121702.4766	136018.625
165228.1719	126881.1875	165932.5	308266.4063	475520.7188	84549.66406	455242.6563	99017.36719	130660.4922	166401.1563	219505.375	279065.375
255386.2344	174489.5313	201908.75	188851.75	190237.0938	235934.2344	222179.0469	156438.9375	252338.4375	86171.57813	206547.5469	238467.8281
217612.125	228511.6875	258702.7813	230120.625	233760.3594	289921.4063	188738.1875	177215.3125	128762.4453	863822.0625	286828.625	205935.4375
164317.8906	144385.4375	206995.9531	309121.6563	460514.7188	77348.33594	854781.625	109724.2813	143123.2344	120649.1016	197466.2188	248372.875
173811.3438	215364.4688	176332.1719	236086.1875	204776.0938	238878.5156	156138.7813	185567.875	231431.8594	150353.8125	186374.4375	256981.7031
231954.1406	182744.625	190945.2344	76984.32813	175255.5625	220700.0313	194843.2344	236228.1563	202414.875	162974.9531	216615.3281	214509.2813
Filtered	Filtered	Filtered	Filtered	Filtered	Filtered	Filtered	Filtered	Filtered	Filtered	Filtered	Filtered
125180.1094	138216.0625	139189.2188	285263.75	387683.3125	65849.74219	715258.125	91310.14063	114363.6094	208418.2969	188706.9844	206439.2188
231218.3281	206253.5781	186283.7656	164116.8438	163178.1719	201789.7188	197043.2031	219431.2969	225649.5625	157743.5625	176454.6875	216210.0156
230397.125	179693.375	275010.625	97786.88281	97781.38281	196981.1719	154148.3125	246538.7656	146160.375	129036.4219	156594.875	180511.6094
Filtered	Filtered	Filtered	Filtered	Filtered	Filtered	Filtered	Filtered	Filtered	577859.875	4016.993408	Filtered
210684.8906	169138.9219	206254.75	171144.6406	155419.1875	204156.125	154124.4688	198892.6406	178115.6406	153410.2656	180833.8906	181255.125
Filtered	Filtered	253109.2344	Filtered	Filtered	Filtered	Filtered	Filtered	Filtered	Filtered	Filtered	Filtered
75803.86719	95867.42969	281863.9688	190081.4688	202156.4063	116305.875	66531.82031	183241.8125	153751.7656	160647.4063	156209	201709.7656
153097.5313	157882.6563	Filtered	Filtered	94655.34375	Filtered	Filtered	Filtered	108442.6406	Filtered	Filtered	161801.9219
190007.1719	171729.4219	295132	129026.2813	138759.8906	158691.6875	146322.1875	250884.75	263844	184301.25	179491.7813	241168.0625
143389.9844	146675.625	153498.6563	146544.8125	171169.4219	200161.0313	141920.3281	135503.8125	202814.0469	110700.1406	166251.5781	229714.9531
214372.4531	159377.1563	174828.0313	309727.0625	264768.5	175400.4531	187736.1406	163616.6875	166059.9688	199447.3125	190256.4531	251135.9531
91818.38281	29970.35742	133258.3594	126552.1953	76012.82813	25176.02539	34232.28516	59416.98828	49491.00781	772810	37206.95703	199614.1406
159653.5313	168095.6563	154430.6875	162706.2969	123784.8906	173236.5938	117092.6641	123572.8125	161739.5781	141256.6406	132708.7969	135276.875
114885.0547	114690.6953	120798.0078	159312.3906	374157.0313	90920.82813	380796.8125	50173.74219	80959.22656	230462.875	101954.0156	194672.75
91344.59375	27442.87891	130969.4219	123903.1719	65357.32031	32788.82813	32361.01953	63083.57813	44432.85547	624061.5	69360.14844	177773.2344
Filtered	Filtered	Filtered	Filtered	Filtered	Filtered	Filtered	Filtered	Filtered	Filtered	Filtered	Filtered
85507.03906	240102.6563	124730.4531	109873.2656	109340.2891	123035.5	75997.19531	190205.8281	133726.8906	175359.4688	182199.6719	230469
160018.3906	164607.9063	171638.0156	131533.4688	165430.2813	179183.9219	152661.3125	187813.7188	166430.625	154914.2969	142801.2031	156580.9531
15850.99512	Filtered	38007.14844	88060.28906	Filtered	Filtered	Filtered	Filtered	Filtered	Filtered	Filtered	89472.58594
43561.57422	45870.59766	122078.2734	165319.0938	377834.5313	17902.23828	683640.5	18912.22461	96077.51563	111180.5391	126786.8047	207831
41081.37891	43715.39844	40891.86719	57214.65625	21287.57031	34919.49219	46915.18359	1101698.5	52858.14844	Filtered	56077.50391	1342149.875
120792.5625	102652.8594	119879.8438	128568.4141	312970.0625	33180.80859	591085.375	57653.10547	86030.375	119294.8516	143769.8438	179841.6094
2.14E+05	7.34E+04	4.28E+05	5.01E+04	6.57E+04	6.29E+04	8.06E+04	2.26E+05	1.83E+05	1.12E+05	7.96E+04	1.44E+05
163865.4063	139236.75	118047.4922	202889.9219	170420.125	207459.0781	130074.0078	138362.0313	120364.2422	157088.0938	103876.6094	137735.0938
132267.2969	72029.61719	125202.7344	149630.3594	109846.4922	91565.54688	235270.8906	107653.0781	164558.2656	148555.0781	137382.6563	191157.9844
94732.75	5956.991211	141110.25	48688.21094	48964.09766	39987.28125	841015.5625	18951.32813	366527.6875	1021886.563	396270.2188	122960.5156
91252.125	81503.98438	418078.0938	127083.6797	120543.5313	233561.6094	98111.82813	141685.0625	140970.0938	157131.2344	159220.9375	107209.5313
127091.5781	204990.4531	65642.9375	102612.8984	112670.2422	109278.2734	156575.9063	82444.33594	84555.5625	89862.89063	64535.00781	90950.26563
83261.125	83887.95313	143703.2813	130218.1484	130332.8906	392587.3125	107074.1406	133495.3438	103309.3984	70619.92969	108628.5469	80469.03906
64131.26563	67409.21875	73437.71875	67303.55469	173984.3281	118837.1094	165219.6875	53175.64063	56525.09375	718903.25	43586.31641	47243.91406
231138.2813	267020.875	16451.74023	21077.39063	209868.0781	23426.8125	17328.30859	58506.72266	17141.35547	65708.28906	63580.47656	24055.33398
Filtered	Filtered	Filtered	Filtered	279048	Filtered	48099.33203	Filtered	Filtered	46220.52344	Filtered	Filtered
165696.0313	134632.5625	131328.1406	130470.6875	74564.75	106198.1719	152124.3594	131326.2031	138465.7031	171439.7188	170913.0938	186182.9219
139979.0313	72410.3125	86711.5625	165837.0938	246239.2656	43376.47656	492519.0938	42412.84766	54849.48828	64300.32813	118590.9531	136175.2969
120260.0781	100876.8516	105319.4141	99472.83594	137435.5	114004.5859	126927.75	141398.6875	122985.9766	118906.8906	87997.92969	176938.0938
98713.88281	114759.8438	99474.14844	104960.2891	115035.1797	161961.6719	110636.6953	149902.9063	266102.9688	31354.09375	57540.79688	99095.28125
2327.536133	9865.177734	12412.73145	17616.96289	Filtered	Filtered	6299.546875	6371.931641	Filtered	621374.9375	Filtered	Filtered

107148.7656	77789.1875	107126.8828	114891.4453	112286.5859	146534.8438	133005.8438	134102.2031	154154.1406	123228.7813	138987.3594	124582.8359
160684.0313	161774.2188	174659.9844	116682.5078	74334.85156	113808.4453	147166.9531	93983.14844	124440.3828	175385.8594	113370.5938	181704.0313
119823.8203	42769.40625	122160.8047	157742.3125	373825.7813	42453.76172	233346.2188	68572.24219	93426.16406	116700.1875	134292.9531	202932.2188
127932.4766	140059.75	110126.5547	219038.8594	103946.0547	110656.8281	100326	87881.4375	112876.2813	88882.70313	139156.1094	151271.7813
129440.2969	Filtered	186132.3125	104538.7969	Filtered	136746.1875	118772.3594	Filtered	111795.4531	Filtered	Filtered	154891.6094
Filtered	Filtered	Filtered	Filtered	Filtered	Filtered	5099.342773	Filtered	4565.03418	952060.875	Filtered	Filtered
113874.4219	159860.9063	65643.59375	155776	105551.3672	76064.28125	76975.17969	71259.17188	92352.03906	99223.60938	182495.1719	126089.3516
75444.57031	190004.8594	36480.80078	329313.1875	105388.5781	64672.58984	30095.4082	76421.10938	48165.01953	249923.0156	94913.76563	104932.1484
134982.5938	94691.90625	113372.375	127870.625	115363.5156	120396.8047	88234.8125	98006.64063	130173.2734	207297.1563	104607.4453	125981.4531
Filtered	Filtered	Filtered	Filtered	Filtered	Filtered	3606.018311	Filtered	Filtered	542961.75	Filtered	Filtered
65786.49219	158574.2969	59443.71484	83180.98438	123451.8984	132777.2813	247423.5	50269.64844	53498.45313	88808.75781	73590.83594	58727.82813
Filtered	Filtered	Filtered	Filtered	Filtered	Filtered	Filtered	Filtered	Filtered	105247.4063	Filtered	Filtered
Filtered	Filtered	Filtered	Filtered	Filtered	Filtered	Filtered	Filtered	Filtered	397062.125	Filtered	Filtered
132913.9063	84118.79688	132640.4531	152907.4531	88946.42188	154805.1406	237302.8281	111948.4219	302167.25	99512.25	51175.17578	155143.8906
205368.2188	Filtered	Filtered	114740.5469	Filtered	154359.7969	Filtered	Filtered	Filtered	Filtered	Filtered	45888.92969
Filtered	Filtered	Filtered	Filtered	Filtered	Filtered	Filtered	Filtered	Filtered	Filtered	Filtered	Filtered
117536.4609	92226.71094	84620.15625	55725.5	91815.6875	110440.7578	84511.78906	90560.95313	83573.73438	108507.9609	89827.71094	87424.53125
18618.37109	72187.21094	56217.61719	283713.9688	100551.4141	40397.05859	305744.0625	Filtered	34543.08984	Filtered	69615.69531	75566.34375
141312.9844	104373.7109	Filtered	120775.6406	139743.6563	52143.07422	558540.5	66023.97656	45770.90625	68775.11719	142025.0938	6386.256836
71767.17969	94329.50781	56875.61328	107034.2109	86005.41406	108923.4922	90462.99219	79559.95313	121066.4844	91142.34375	93660.875	107060.6016
93938.57813	64289.99219	64650.80078	138956.5625	68446.85938	94772.4375	76032.10938	54476.35938	129992.2109	67750.10938	92729.17188	121339.9453
86242.50781	113610.0938	45336.65625	91405.21094	106530.4063	33054.08594	332543.625	91569.23438	87291.58594	68977.78906	113935.8594	116391.4844
22198.9043	51302.5	23430.69531	21700.09961	27488.3418	25228.29102	25288.5	70978.50781	37341.64063	3400664.5	26060.60938	65922.73438
70922.14063	61886.30859	79104.1875	138093.5625	51476.62109	66850.42969	84463.79688	100337.25	139022.2188	92729.45313	96198.28125	144652.1875
71062.33594	104645.0625	143869.3438	104538.2188	70766.125	95520.33594	104749.2813	99536.10156	75240.07031	73069.26563	72446.57813	63223.88672
134125.9688	98043.80469	110571.7656	140999.3125	36296.84375	4340.522949	88949.69531	80304.90625	111350.3594	Filtered	66447.53906	107278.9766
93381.17188	77415.03906	92247.23438	65858.35156	43397.82813	27410.9082	110158.2813	59698.84375	51354.125	39370.16016	113638.0781	68734.85156
93697.9375	120221.1016	62132.26172	121552.0078	128433.8828	142895	78888.80469	116852.2109	33583.48438	104653.2266	99632.19531	47761.14844
102679.4766	20514.20313	40124.64063	111545.2969	41847.14063	25718.4375	26740.64063	56399.54297	48646.76563	304079.5938	17591.05859	148769.7031
110489.5	82008.21875	71644.84375	76856.71875	43931.37891	65606.24219	82525.03125	77018.64063	71444.6875	562731.6875	112292.9609	101979.6875
4762.992188	Filtered	Filtered	Filtered	Filtered	Filtered	8447.049805	Filtered	9668.962891	171658.9375	Filtered	Filtered
76819.82031	59979.69531	71526.82813	72402.59375	80575.90625	65502.38281	49819.14844	92490.99219	68882.70313	669374.625	96659.41406	97347.14063
Filtered	Filtered	Filtered	Filtered	Filtered	Filtered	Filtered	Filtered	Filtered	Filtered	Filtered	Filtered
48689.67578	17424.7793	38243.60938	65918.64844	27548.84766	14180.91699	14956.84863	65785.96094	40106.28125	250153.0156	22561.57422	136953.3438
34449.67969	60010.04688	80751.84375	24695.48242	31928.91406	47599.16406	25759.4043	109195.375	68024.14063	Filtered	186885.3906	147593.0781
79366.28125	62652.82422	137994.7344	51138.14844	42999.40625	31125.60742	80414.6875	77077.6875	95133.5	Filtered	53390.21875	106111.6797
72136.96875	97061.39063	59799.75391	51293.02344	71113.21875	84866.1875	77614.03906	80931.34375	77216.27344	36833.80859	52297.25781	96327.03125
Filtered	Filtered	Filtered	Filtered	Filtered	Filtered	Filtered	Filtered	Filtered	Filtered	Filtered	Filtered
28914.99219	30834.09961	39702.46875	47963.23047	72908.59375	62176.75	222981.8438	29891.03906	28413.13477	55793.30469	35861.22266	26165.82031
Filtered	Filtered	Filtered	Filtered	Filtered	Filtered	Filtered	Filtered	Filtered	71119.20313	Filtered	Filtered
61873.32031	66279.91406	54645.15625	81107.67969	66095.5	81061.55469	92026.66406	48091.01563	83243.25781	42407.58984	69779.125	82849.125
64968.46875	52140.9375	48188.10938	45238.63281	53961.26953	82532.76563	37303.35547	54949.9375	53141.46875	65217.04688	42516.40625	38876.20313
51824.95703	84691.02344	45499.49219	55433.05469	139767.9688	19856.71094	275885.5313	43736.54688	45439.78125	55485.11719	79330.07031	62589.54688
Filtered	59312.59766	Filtered	60006.98828	Filtered	Filtered	Filtered	Filtered	72173.125	Filtered	Filtered	52668.23828
61734.32813	54000.05859	297446.3438	75993.60938	87119.78125	69013.23438	14928.25391	155841.125	66675.38281	38997.03516	194195.0938	22207.9375
Filtered	Filtered	Filtered	Filtered	Filtered	Filtered	Filtered	Filtered	Filtered	Filtered	Filtered	Filtered
56646.75391	91708.26563	152831.5	61141.57422	55851.32031	57319.10547	99865.42188	134666.8906	115438.4609	188473.5156	62998.52734	104726.1406
61215.59766	85483.00781	73888.60938	82506.125	69214.72656	84430.03125	54183.80078	54948.95703	61501.0625	68767.67188	63569.59766	65282.15234
196665.5	101387.2578	21853.49414	74509.42188	3256.618164	94263.22656	3969.188477	117816.6016	80965.84375	Filtered	4560.006348	62742.37109
40027.73047	52365.21094	49170.63281	75597.33594	120761	25590.41602	239842.7813	31761.17969	36964.63672	78191.10156	81773.79688	70349.97656
40518.20313	18439.26758	40214.42969	44115.66797	26712.61133	12915.20117	24839.36328	10745.31055	24641.26563	73283.25781	26760.71484	48957.17578
77281.19531	87512.14844	85418.33594	55573.42188	66509.55469	72648.01563	35791.82813	74880.11719	61304.29688	65409.04297	88788.20313	72580.20313
43982.80859	64644.71875	51659.48438	60991.73828	62432.16016	78834.29688	51006.85156	44771.48828	82831.07813	Filtered	59240.32031	77158.1875
Filtered	Filtered	Filtered	Filtered	45692.57813	Filtered	Filtered	Filtered	77803.86719	Filtered	51517.33594	Filtered
38876.64453	41726.04688	29031.61914	61941.41016	46514.5	35208.99219	74003.75781	35731.24609	31736.26758	39624.44531	38930.83594	48309.20703
61648.92969	42229.27734	62377.77344	74322.19531	72982.14063	53058.57813	69078.375	53614.875	59886.38672	Filtered	50272.20313	59146.95703
Filtered	Filtered	Filtered	27318.16992	Filtered	Filtered	Filtered	Filtered	Filtered	56748.57031	Filtered	64025.85156
81714.27344	59180.08594	44149.38281	68581.75781	39838.37109	38950.87109	39991.63672	68957.38281	96145.35156	245553.875	78588.46094	31241.70703
Filtered	Filtered	Filtered	Filtered	Filtered	Filtered	Filtered	Filtered	Filtered	Filtered	Filtered	Filtered
42745.05469	54783.19922	40479.60938	56606.19141	79393.16406	42157.26563	153866.25	30135.10156	30666.89453	Filtered	58395.41016	40523.8125
38075.83203	42740.37109	38812.63672	64550.42578	68896.20313	52663.44141	34579.80469	74130.28125	62025.90625	55402.54688	78330.95313	37883.60156
28707.97852	55499.95313	43498.09375	34498.26953	33028.05078	45241.90234	53483.95313	52745.09766	46369.34766	78108.39844	44841.33203	46321.71484
47641.59766	50002.07031	31543.19336	31480.1543	80322.5625	39900.01953	154749.5469	27406.18945	27784.91406	36425.02344	45244.8125	40268.33984
Filtered	Filtered	Filtered	Filtered	Filtered	Filtered	Filtered	Filtered	Filtered	57935.87891	Filtered	Filtered
71753.25781	41746.52344	53521.18359	56623.28906	65251.67188	54075.50391	45297.97266	94174.38281	60033.08594	Filtered	83061.1875	61524.67969
48417.86719	63282.34375	49985.73438	44277.50391	63436.80078	66022.32031	45699.34766	55443.54297	54563.78125	Filtered	37024.57813	58689.48828
41616.60938	63717.74219	81827.125	86421.73438	53679.55859	71869.72656	59942.76172	43240.13672	62710.94531	49684.375	45515.03125	65739.09375
70796.6875	59282.05078	70126.67969	54922.25391	41707.25781	39221.96875	51206.71094	77352.71094	71575.46875	45902.36328	64646.3125	64625.54688
70049.46094	46110.13672	80650.85938	74492.04688	87526.02344	57130.76953	65450.25781	74425.97656	70213.625	Filtered	61591.35938	84960.73438
Filtered	Filtered	Filtered	26137.2793	69676.33594	Filtered	96032.15625	Filtered	Filtered	Filtered	31646.74219	24778.17578
72776.78125	61266.42188	51066.52344	30668.7168	53461.34375	52364.96484	44140.375	65173.57031	48546.13281	Filtered	48758.625	56389.35547
56352.58203	56858.48047	42432.65625	25636.11328	50072.6875	53077.24219	52941.78125	45789.28125	38461.5	165909.4063	43579.44531	33628.00781
60048.73438	55489.86328	53000.19141	38606.67969	56863.80078	70790.14063	42036.36328	48568.08594	61850.98047	Filtered	63252.38281	51946.07031
40034.76563	52102.88672	42340.33203	31360.04688	51664.88281	54596.41406	46225.02344	47424.09375	30872.46094	52737.52344	44087.94141	43043.21875
33259.37109	48560.29688	62290.24609	94250.96094	134397.1563	54081.22656	111514.0391	28948.7832	33050.45313	Filtered	40290.91797	36531.14453

54276.13672	83259.79688	Filtered	38873.625	63979.85156	71662.6875	44031.70313	71374.44531	52144.89063	Filtered	73195.33594	Filtered
48329.11328	57844.40625	58709.29688	40183.3125	113723.8203	13178.16113	245534.1406	44234.00391	38675.92578	39086.82422	54837.73828	68874.96875
31674.54688	9923.945313	22892.39844	17948.30664	17954.74414	10166.25293	21905.93945	34549.21484	23269.29688	166822.8594	18204.60547	87901.95313
Filtered	46323.16797	40974.83203	37232.42578	Filtered	Filtered	Filtered	Filtered	Filtered	Filtered	63186.67578	Filtered
34505.0625	35584.07422	46943.69141	39077.25781	46966.14453	57912.74609	27694.31641	32283.28906	36040.66406	Filtered	31500.42383	33166.51563
37457.89453	32694.04297	32746.45703	47330.82422	49318.15625	110722.3438	27801.74414	39502.62109	52673.92578	Filtered	44833.01172	44973.44531
42728.4375	46279.66797	50679.51953	57369.07422	43980.69531	62914.55078	41058.52734	58451.25391	51760.97656	36834.72656	30827.16602	43222.38672
43987.25781	90798.52344	59351.99609	25004.01172	32377.04688	34835.58984	9984.063477	95457.83594	67572.53906	181424	96030.57813	82063.77344
84242.25	Filtered	Filtered	Filtered	Filtered	Filtered	Filtered	Filtered	Filtered	Filtered	Filtered	Filtered
53741.00781	48920.85156	73611.15625	46030.82031	82239.75	19108.89844	48806.33984	Filtered	14083.01172	Filtered	28548.1543	70258.26563
40085.33594	42469.09766	37335.87109	30712.14453	124970.9219	15950.7793	216583.1563	27160.03125	24840.82227	39625.61328	49923.33984	40710.40625
47918.89063	44144.93359	34183.57813	36571.25781	37813.14844	58577.90625	37567.85938	34043.14063	52466.14844	66496.3125	39914.62891	41172.875
76160.28125	31113.65039	59133.86719	49224.11719	55412.62109	38530.17188	22887.60156	36361.17188	47930.05078	66309.21875	45880.92969	46351.70313
Filtered	14612.13672	Filtered	Filtered	Filtered	Filtered	Filtered	Filtered	Filtered	Filtered	Filtered	Filtered
45646.23047	65319.10547	35373.50391	28410.8125	45886.55469	39917.07031	43879.23828	55498.92188	52158.96484	28502.89844	24463.23633	58557.37109
78243.26563	44896.88281	57729.60938	30624.6543	20694.20703	12716.72949	33635.67969	17274.93555	25591.07813	Filtered	57275.74219	20444.50977
46096	35996.39844	43814.88672	60278.39844	40290.69531	50310.61328	63946.50781	68672.25	49374.5625	43792.14063	42379.07422	67444.98438
26159.64063	18466.86914	20553.90234	15632.9873	19799.61133	12928.96777	11680.62207	16591.98633	8550.657227	1791872.375	12767.58984	17000.875
42607.09766	33258.16406	48742.89453	40709.625	40861.88281	35245.92188	42433.73438	47900.13281	58009.88281	47056.49219	50363.0625	53315.76172
59445.60156	37057.06641	65953.58594	37084.30078	58468.15625	35750.25391	42431.01563	44432.63281	49275.72656	Filtered	50708.16016	41554.85938
17762.44922	12088.66895	125483.1875	30312.27734	15442.00488	44400.89063	51942.75781	72665.82031	14733.85938	Filtered	20865.56836	72901.38281
Filtered	Filtered	5671.741211	3022.12793	Filtered	82479.50781	2787.879395	2306.704346	10799.05762	Filtered	5713.404297	4779.128418
38693.91016	39300.16797	33934.76563	47673.48438	117415.0313	17211.32813	141820.125	30417.11133	39348.96484	83657.98438	75464.5	37583.04297
42889.17969	42705.94922	33834.48438	40275.04688	39631.42578	45654.00781	34378.19531	46111.72656	51039.32813	49371.50781	34519.14453	46969.13672
17890.54492	11332.69727	3896.808838	2255.574219	Filtered	26573.11914	2283.025879	28723.40039	Filtered	Filtered	Filtered	11332.29199
Filtered	Filtered	Filtered	Filtered	Filtered	Filtered	Filtered	Filtered	Filtered	44872.98047	Filtered	Filtered
Filtered	3941.789551	Filtered	Filtered	Filtered	3054.899658	Filtered	Filtered	Filtered	Filtered	Filtered	Filtered
42561.56641	54086.72266	40150.03125	56064.69922	55279.15625	41993.93359	56828.33203	30135.10742	30354.53125	Filtered	58653.51563	40525.42188
Filtered	87200.8125	Filtered	Filtered	Filtered	33553.36328	98621.47656	Filtered	Filtered	Filtered	Filtered	Filtered
Filtered	Filtered	Filtered	Filtered	Filtered	Filtered	Filtered	291839.5625	Filtered	Filtered	Filtered	Filtered
Filtered	Filtered	Filtered	Filtered	Filtered	Filtered	6180.44043	Filtered	Filtered	Filtered	Filtered	Filtered
36766.03125	39310.04297	50159.53516	55811.74219	22849.45117	39879.44531	43437.88281	74787.39063	93221.92188	114758.125	37927.42969	160501.125
26273.16602	54024.70313	25662.93945	24893.63672	42380.18359	25643.47461	208025.5156	32382.17773	27665.25977	98345.625	42094.80469	24153.67969
47593.875	40895.46875	38300.44141	24824.67383	36081.02734	43928.39844	30673.75977	40495.21875	44674.76563	Filtered	28762.81445	30416.83398
36035.67578	28672.94727	31953.95313	46254.71094	38475.49609	36414.875	67572.60938	32402.95313	31571.76367	Filtered	29450.4375	38673.96484
65506.71875	78663.74219	41656	Filtered	Filtered	2004.219727	2762.18335	84897.94531	45385.09375	Filtered	71181.4375	89350.22656
40871.83594	52946.40625	33740.07422	47553.02344	27542.62695	25127.16797	40180.28906	44169.76563	34859.17578	218056.9844	43936.9375	46714.46484
49731.84375	59872.11328	48513.53125	55661.44922	46227.39063	51580.90234	17615.60938	40734.91406	37686.42188	Filtered	41607.3125	46831.90234
35691.64063	40411.04688	31348.86523	45983.17188	19652.20898	40205.33984	41155.40625	70159.46875	84808.57813	155652.4688	40966.33203	117598.7813
41787.33984	42492.03125	41403.53516	24986.25	55364.79297	50381.63281	25119.06445	32813.5	28283.80469	Filtered	33580.96875	43287.60938
74157.82031	44553.22266	50167.02734	23386.27148	22739.36133	41463.32813	48518.3125	27228.43555	38317.57031	32032.4707	48689.30469	45036.66016
41719.19922	41266.35938	37211.46875	48996.02344	37848.82813	39034.67969	36212.76563	26878.32031	36538.69141	25212.33984	36354.18359	38134.38672
30697.87891	37366.39063	39874.66406	39477.64063	33247.57422	44234.41016	43346.3125	39005.6875	43404.03125	21130.15234	45426.14844	49534.21484
39772.53516	39114.62109	61054.09375	28871.58594	43667.08594	46825.82422	52072.69531	20433.40625	25934.25195	Filtered	34238.6875	37099.30469
Filtered	Filtered	Filtered	Filtered	Filtered	Filtered	Filtered	Filtered	Filtered	Filtered	Filtered	Filtered
36691.51172	Filtered	Filtered	Filtered	35638.85156	40569.72656	39333.44141	Filtered	8939.439453	Filtered	Filtered	Filtered
23848.78516	18846.10547	15775.5	16185.91504	13421.00195	18030.62891	13626.08496	101172.0547	66540.375	Filtered	17833.6543	37930.45313
Filtered	Filtered	Filtered	Filtered	Filtered	Filtered	8632.508789	Filtered	Filtered	70691.69531	Filtered	Filtered
Filtered	2557.874023	Filtered	Filtered	Filtered	Filtered	2886.887695	Filtered	Filtered	Filtered	Filtered	Filtered
33802.59766	33762.65234	56411.76172	51108.31641	61416.64453	25313.19141	44503.62891	Filtered	18568.05273	Filtered	18260.39453	29808.71484
49494.6875	39288.39063	45270.68359	35258.5625	26231.80078	35422.90625	26747.05469	34796.84375	38390.02734	60615.50391	48079.21875	39281.22266
20812.89844	26209.2207	27054.37305	42510.15234	35773.23438	50129.05859	35120.47266	32507.33203	27024.70703	25295.78125	18794.66016	27092.60547
28766.88672	23089.11133	35373.79688	22854.2207	27506.48828	30027.64063	37376.72656	30028.33008	33252.44141	Filtered	29406.42383	52492.67578
30084.74609	23353.76953	244497.2031	32298.02344	33275.82813	46843.72656	14526.13867	15571.75781	21258.76953	Filtered	18627.88281	28755.79688
48180.02344	12184.625	22025.69922	14157.88672	Filtered	32530.94336	18049.10938	28297.42383	56611.82813	17092.4043	11100.71289	59301.57813
Filtered	Filtered	Filtered	Filtered	63394.60156	Filtered	Filtered	Filtered	Filtered	Filtered	Filtered	Filtered
Filtered	Filtered	Filtered	Filtered	Filtered	Filtered	Filtered	Filtered	Filtered	Filtered	Filtered	Filtered
41835.21094	6708.05957	28794.91016	23927.23828	12513.36133	9249.97168	8622.563477	8765.31543	16789.52734	416518.7188	7463.827148	32529.5293
Filtered	Filtered	Filtered	20686.32227	25928.9082	Filtered	70046.79688	Filtered	Filtered	Filtered	16469.23828	23477.82813
41756.64844	38404.93359	43121.63281	28590.47852	37681.24219	27846.00586	28546.58594	31940.14844	31429.47656	Filtered	31478.46875	35888.32813
21020.49219	5869.500977	13057.32813	29334.14063	14335.6875	12754.86719	7426.729492	34990.64844	19513.16016	278693.3438	10227.2666	61650.69922
49615.76953	51898.47656	44695.51563	54037.62109	52822.45313	7088.894043	Filtered	22709.48242	25097.08398	Filtered	43614.86328	33590.6875
42923.01563	23391.66406	23030.5957	46720.48047	26903.86133	47813.31641	82876.02344	30596.89063	73762.72656	44753.75	20516.52734	27007.58789
Filtered	Filtered	Filtered	Filtered	Filtered	Filtered	Filtered	Filtered	Filtered	Filtered	Filtered	Filtered
6955.638672	5717.013184	6392.885742	14963.9209	11994.70801	Filtered	58381.63672	Filtered	11932.79102	Filtered	4372.57373	Filtered
3818.527832	Filtered	Filtered	5689.287109	Filtered	Filtered	1666.253418	Filtered	1252.324951	Filtered	Filtered	3492.755371
31637.75195	15310.98047	26798.96094	16970.33203	19377.75195	26504.64453	20832.77734	34918.35547	28473.49805	Filtered	18770.63477	38205.32031
25966.45508	25163.95313	28860.66797	27010.32227	33423.42969	44565.93359	23271.32813	30511.80078	23009.81641	29221.60156	23627.61914	26098.76563
Filtered	Filtered	Filtered	Filtered	Filtered	Filtered	Filtered	Filtered	Filtered	Filtered	Filtered	Filtered
9101.517578	39764.98438	51788.44922	30086.49414	7081.069824	9142.572266	5729.750488	7237.163086	9057.014648	Filtered	92176.15625	46464.44922
Filtered	68730.90625	Filtered	Filtered	Filtered	Filtered	Filtered	Filtered	Filtered	Filtered	Filtered	170207.0313
Filtered	81020.61719	Filtered	Filtered	Filtered	Filtered	Filtered	Filtered	Filtered	Filtered	Filtered	Filtered
Filtered	Filtered	Filtered	Filtered	Filtered	Filtered	47261.9375	Filtered	Filtered	Filtered	16509.40625	Filtered
29504.31641	24354.71484	28923.68359	27089.63672	28445.80273	35211.57813	23025.5625	27621.89844	35883.16406	14514.46094	23887.37891	41507.1875
Filtered	Filtered	Filtered	Filtered	Filtered	Filtered	Filtered	Filtered	Filtered	100438.6719	Filtered	Filtered

9027.342773	1972.463623	5519.81543	11659.88672	26261.00781	52368.27734	28437.37891	9006.695313	19141.19531	Filtered	Filtered	5623.740234
26008.49609	36556.82031	26118.83984	23813.61914	29245.41406	43194.50391	29397.08594	21073.67578	34207.05078	Filtered	34922.58984	39123.44531
25012.48633	25973.89648	Filtered	16655.47656	13166.6123	28642.40234	28385.73633	23524.49219	21095.36133	Filtered	29329.83398	33748.56641
36367.35547	27285.75	31757.5293	39388.03125	24338.91602	36807.82422	59539.22656	32185.17578	68831.4375	35957.35547	12150.43652	44750.97266
Filtered	11915.67188	Filtered	Filtered	21210.81836	Filtered	42152.54688	Filtered	Filtered	Filtered	Filtered	Filtered
31530.81055	31521.48828	25161.86133	21791.00977	27338.14648	29729.17383	20072.45313	32602.09766	25157.65234	36969.58594	16590.85938	22428.47852
Filtered	Filtered	32561.5332	14556.95313	Filtered	Filtered	Filtered	Filtered	Filtered	Filtered	Filtered	174799.5469
16398.51758	13431.10645	16304.0127	22872.57031	32410.08594	45192.92578	17640.83789	14633.81738	15050.31641	Filtered	15346.80078	14779.38477
Filtered	Filtered	7985.148438	10194.55566	4687.630371	12477.1748	12190.12891	2729.061523	11192.19531	Filtered	Filtered	8117.46875
45155.42969	4506.302734	3882.828857	35572.80859	14690.1543	4517.158203	11638.83398	Filtered	5940.130859	Filtered	Filtered	5177.897461
25936.64258	20509.9082	24371.77148	33912.11719	25085.10938	30465.52734	40980.01953	22506.81445	24649.69922	56333.87109	23927.6543	28828.32031
21317.45117	22367.28516	15213.60645	25332.67188	25193.06055	28549.26758	18034.70898	21941.12305	24541.74023	96038.13281	18572.04688	19272.35352
38859.10938	37994.03906	15197.89355	22611.20508	21842.36133	31410.70117	14595.9209	38752.00781	24021.86328	21693.9668	11478.10547	41581.49609
35605.23438	34345.39844	17566.02344	24663.28125	23729.37109	26575.75586	32116.94141	28798.96094	32590.80664	Filtered	28286.81445	38631.37891
33715.46875	27678.15625	24626.57227	24312.90039	27377.5625	29320.82422	31682.82617	17573.09766	19063.32813	28489.80078	24372.89453	24297.45703
22608.88281	38539.79688	18828.13281	14151.76172	13906.46484	46600.75	28181.75977	30352.19727	20346.44922	Filtered	25787.2207	29122.99805
23278.88281	28902.57813	25220.17383	Filtered	19333.64453	22967.68555	18659.94141	25737.97852	36464.42969	Filtered	30178.86133	Filtered
Filtered	Filtered	Filtered	4125.984863	6340.662598	9661.330078	5772.327148	Filtered	Filtered	Filtered	17049.11328	Filtered
12111.4209	5872.813965	8901.711914	10244.57813	9432.244141	4031.497314	23464.11914	45311.30859	7714.885254	111771.0391	8728.493164	10697.8457
Filtered	Filtered	Filtered	Filtered	Filtered	Filtered	Filtered	Filtered	Filtered	Filtered	Filtered	Filtered
11515.81738	20878.98242	23154.20508	5846.563477	17207.50781	29691.02539	Filtered	13960.38965	Filtered	Filtered	11626.50586	16806.38867
31769.96875	6855.115234	3101.602783	3287.983154	Filtered	7478.095215	13038.13672	Filtered	8532.420898	Filtered	6381.536133	7594.422852
27666.9668	20473.32813	19303.56445	16017.30664	17751.05078	15192.21973	25301.87109	24192.875	24579.71875	Filtered	26712.55469	31977.6875
17855.27148	24517.52344	16258.83594	51535.92578	23285.55469	26828.90039	19201.91406	14977.88281	27899.94336	24948.16602	26490.95117	40721.51172
24167.1875	20874.65039	17990.95313	27520.80859	21478.85352	27091.26953	17917.54102	43766.59766	22731.07227	Filtered	22972.98633	35893.10938
22060.73242	19546.94922	34842.65625	25026.24219	18171.17578	26813.38086	22570.64844	19131.85547	22949.76953	Filtered	24847.43164	29080.0625
Filtered	Filtered	Filtered	Filtered	Filtered	18142.80078	53261.46094	5200.20459	25260.17773	Filtered	Filtered	7869.353516
12234.66602	11709.92383	5814.915039	10417.39746	69267.125	Filtered	152471.3125	Filtered	8387.238281	Filtered	8165.088867	7736.955566
25287.89648	26480.91602	22608.65625	22151.92383	23846.81055	24232.30078	18306.71289	18681.74023	20856.24023	22980.68359	19113.66797	27044.99023
26368.03125	26060.38086	23819.55469	26073.75977	21859.43164	25059.91016	19594.04688	20886.78125	20726.60742	53463.86328	19154.99219	23323.875
18587.11523	15350.85352	19831.80859	16840.09375	28968.04102	45359.03125	15652.16895	16828.25	18475.53906	38202.17578	12763.07422	15910.41602
113152.2266	Filtered	Filtered	Filtered	Filtered	Filtered	Filtered	Filtered	Filtered	Filtered	Filtered	Filtered
20649.375	22021.70508	21425.63086	32671.88281	22681.69727	35217.38281	17116.16016	25367.28906	23809.47266	Filtered	21410.91016	20550.42969
Filtered	Filtered	Filtered	Filtered	Filtered	Filtered	Filtered	Filtered	Filtered	32221.21875	Filtered	Filtered
24955.65625	18484.87695	20743.69531	28620.91016	21574.87695	38962.70703	18437.31641	16160.24805	10617.83984	Filtered	17568.66211	22325.48438
18908.375	20062.75391	28442.17578	22244.62891	21595.72461	25687.83008	24042.80273	20450.97852	28701.10156	Filtered	21216.93555	30424.16602
25328.96875	31142.99219	22908.34766	24310.95703	13127.66699	23216.84961	15272.99902	18200.93945	23553.99219	Filtered	19377.34375	13899.55273
12138.66602	8644.525391	10903.68848	16283.23145	30295	48770.0625	10429.7207	11346.01563	4116.79248	Filtered	6914.325684	8345.568359
Filtered	Filtered	9200.546875	Filtered	Filtered	Filtered	Filtered	Filtered	Filtered	Filtered	Filtered	Filtered
30228.93945	18932.86133	20896.36328	52385.93359	27295.00977	18614.0918	24246.21875	13663.95117	22662.38281	Filtered	21075.67188	37645.4375
Filtered	13853.88086	9816.520508	10731.66797	Filtered	7785.361328	11790.43457	4342.722656	10237.96777	Filtered	Filtered	9425.268555
19203.42578	23772.06445	16166.86621	14616.35645	27889.375	24522.65234	13712.95703	16807.75586	16831.60547	104508.6641	36051.22656	34504.85547
21298.40234	22704.31836	17225.67773	24821.89453	20424.75586	30191.72461	23082.82617	19545.66602	25327.11914	Filtered	17535.22461	24779.06055
14891.94629	14708.31738	21749.83984	41658.28125	52111.90625	10862.46094	91242.72656	8917.286133	6975.410156	Filtered	20459.61133	26892.34375
22603.47266	9683.34375	10979.15137	8546.515625	Filtered	16075.24316	13075.26172	11808.38477	12031.65332	520662.0313	18683.71289	16795.66406
28601.04688	21270.61523	38945.42969	11731.49219	13387.92969	17624.35938	42393.83984	34754.65625	27798.33008	57081.28906	10362.15918	21839.31641
19835.62305	20766.57813	19080.48047	18886.50391	22094.65234	26885.95313	16548.25195	12315.8457	18820.96094	Filtered	14208.45801	24470.06055
23970.91016	25172.35156	20069.29102	25447.81445	21049.57617	25360.13672	14951.79395	15128.76953	20305.85156	Filtered	19233.93359	26705.75977
39893.6875	13355.77148	20800.14844	28929.8418	15437.40918	7369.274902	24266.26953	43759.89063	36024.35938	Filtered	25984.82813	28425.26172
15908.41211	20455.42969	25950.84766	20517.5332	16397.67773	32639.03125	18800.56836	21873.85156	31876.91211	Filtered	26740.12109	22768.55273
18077.71094	28922.56641	17092.06836	16459.00391	25347.66406	22370.93359	16160.96484	17160.63281	21408.90625	Filtered	21600.24805	17475.64063
14453.74902	Filtered	20275.96289	18228.63477	Filtered	14493.69531	12587.87109	32971.78906	38266.60547	Filtered	Filtered	60332.42188
24316.78906	31113.54492	18820.39063	19702.42969	22068.27344	23661.52148	20197.10742	20142.63672	22197.5332	29185.46875	17648.46289	20790.23828
Filtered	Filtered	Filtered	Filtered	Filtered	Filtered	Filtered	Filtered	Filtered	22422.49414	Filtered	Filtered
11859.50098	71047.24219	16862.08203	7006.445801	4116.357422	9542.06543	7765.544922	4513.914063	6583.770508	Filtered	17402.57227	144003.7188
16722.70898	13889.52344	15252.92188	16754.96289	14979.69238	13503.25684	24712.87891	16697.38477	22321.48633	169777.0469	21455.26953	17264.09766
Filtered	Filtered	Filtered	Filtered	Filtered	Filtered	Filtered	Filtered	Filtered	Filtered	Filtered	Filtered
16635.29297	10672.80957	9430.932617	18620.55469	15231.01172	19208.14453	10769.3623	10021.41309	13007.18066	129825.3047	15406.1582	104817.5547
25126.39258	28711.52734	24716.51172	13656.25586	16661.89844	27661.3457	31912.81055	28803.3125	33665.23438	Filtered	43952.30859	Filtered
21193.18164	22846.26953	24455.7168	9196.351563	21906.9668	26175.44141	15949.1582	21781.47852	11050.38867	Filtered	26000.0293	37156.49609
Filtered	Filtered	Filtered	4825.319336	Filtered	3487.164795	Filtered	Filtered	1552.851196	Filtered	Filtered	Filtered
30347.17773	38861.76172	18157.10547	28448.17188	27733.76953	20970.68164	21105.88281	16994.61328	23738.45313	Filtered	17726.66406	16455.45703
Filtered	17266.94531	6279.179199	10532.07617	31196.98242	5571.625977	32830.57813	Filtered	Filtered	Filtered	Filtered	Filtered
19643.63477	22446.22266	20387.17578	19353.10742	20049.49609	15188.84082	15010.63867	22679.51367	20117.62305	Filtered	23130.6543	23984.98047
20193.8418	18387.11328	17565.3418	18944.91992	21293.25391	25861.97656	20228.20898	13102.18848	17939.3125	Filtered	17151.25586	19918.3418
Filtered	Filtered	Filtered	Filtered	Filtered	Filtered	Filtered	Filtered	Filtered	Filtered	Filtered	Filtered
14448.59277	10133.45313	13281.55664	10141.62109	13160.58594	15781.80957	13094.90234	4296.470703	10662.87598	Filtered	10211.32227	9308.601563
15563.33105	43327.65625	14124.42773	16113.55859	18132.67773	21542.01953	13165.79785	20210.7832	24130.73047	Filtered	19494.83008	25544.4707
23116.97461	17923.49219	18690.75	17259.55469	19275.69141	20273.63086	17396.14844	26611.56445	13459.38477	Filtered	16089.10059	13924.08594
Filtered	Filtered	Filtered	Filtered	Filtered	Filtered	Filtered	Filtered	Filtered	Filtered	Filtered	Filtered
11653.76953	6971.524414	7395.176758	8256.943359	28445.67578	Filtered	109365.6406	Filtered	5850.5	Filtered	Filtered	11244.08496
20344.70313	20938.5332	16251.38672	22415.45508	16672.3457	21022.27539	15017.11816	10571.55469	15860.37793	Filtered	22314.06836	16738.60156
12225.72168	9149.482422	12697.31738	7444.768555	36276.85938	Filtered	41884.53906	Filtered	Filtered	Filtered	31206.47461	20671.79102
Filtered	Filtered	Filtered	Filtered	6944.541016	6418.632324	Filtered	Filtered	Filtered	Filtered	Filtered	7531.175781
Filtered	Filtered	Filtered	12388.22168	Filtered	16973.97266	Filtered	Filtered	Filtered	Filtered	Filtered	Filtered

17747.98438	24207.01563	19560.56055	21096.85156	21726.19727	13647.0625	12586.91992	14798.1377	18852.35547	Filtered	11478.07031	17998.35742
10694.49219	Filtered	11308.33008	17586.76172	17282.04102	17586.61328	16693.08594	15846.89063	12299.82813	Filtered	12973.28613	17519.60742
18190.125	15846.05566	12823.76563	19404.00391	14445.54199	19106.95703	20885.85547	15331.60254	22704.05078	159152.2188	29320.4043	24173.81836
16635.39258	15426.30957	15755.92773	18361.55859	19514.01758	28761.5332	25436.34961	12918	22755.58984	Filtered	12173.07422	20849.1875
21252.27148	23283.20508	21503.41797	28535.125	17977.39453	18312.24414	12495.53906	15218.95508	22768.37109	Filtered	19663.76172	19400.92773
Filtered	16325.4873	17245.88672	15403.6377	14706.52734	20413.93555	Filtered	Filtered	17665.02539	Filtered	18643.77734	13707.66797
16670.00977	16906.16211	19067.03906	13419.6543	12695.23438	16963.15039	10246.33203	15528.81738	18638.60938	Filtered	13132.54004	30266.59961
12175.90723	4557.287598	Filtered	12827.68945	8456.06543	12583.40234	6287.404785	14827.14844	12175.81055	Filtered	15461.41406	13135.23242
Filtered	Filtered	Filtered	Filtered	Filtered	Filtered	Filtered	Filtered	Filtered	Filtered	Filtered	Filtered
22749.17578	25305.55273	8530.626953	12641.50098	10089.93359	8776.676758	9930.717773	22834.2793	33274.26563	121295.3438	35240.42188	34062.72266
15840.93066	22639.57813	14905.87891	15310.59375	17989.31641	20370.9668	16255.30566	17808.02734	19502.66016	Filtered	15250.71387	19124.05078
Filtered	30500.04102	Filtered	Filtered	23083.46094	Filtered	27553.92969	1161.558838	25144.1875	Filtered	Filtered	Filtered
3667.201172	29924.0293	4735.822754	3788.668213	4071.47998	5091.770508	31005.45117	Filtered	Filtered	Filtered	2739.26709	3566.35498
7546.562988	8699.537109	4424.708984	6471.025391	7811.82666	6759.021973	4488.940918	6499.896973	4821.541016	Filtered	3426.150879	5948.591797
14270.95996	29508.01172	17300.50977	12216.25879	17428.07422	15288.48633	14866.9541	27736.42578	23581.61523	32439.49023	17580.95508	26051.16016
19401.00195	13465.44141	23658.37695	15679.09668	15632.88672	13929.17676	23626.30273	27264.29297	19038.09961	17376.55859	19484.3125	16178.11621
25442.32422	14658.28418	19169.99219	13506.29785	15638.09668	8919.032227	20730.58203	23709.82422	22407.37891	Filtered	21357.14648	18750.42578
31570.33398	12359.19531	18571.24609	19562.19141	17237.39844	17107.30078	20842.87695	15809.45313	14672.02539	Filtered	20689.25781	34409.76953
Filtered	Filtered	Filtered	18385.29297	31527.6875	5912.296875	68734.11719	Filtered	Filtered	Filtered	Filtered	Filtered
11367.00586	12793.61719	15530.33105	15296.41211	17082.62891	25400.68945	16954.88477	8290.266602	10829.22363	Filtered	15764.04395	17509.11523
21272.6875	19898.92578	11035.20508	13025.72852	18359.54688	17216.35156	16245.00977	21617.23438	23696.98633	Filtered	19179.37109	18603.34961
16280.08398	17480.45313	16430.78125	20129.67578	15707.89063	20282.85938	15640.87891	11837.50098	15006.7168	Filtered	13451.28516	17888.61719
18567.24219	17239.65625	15436.98047	14267.17383	16340.48926	21524.75	14729.47852	17044.57813	20366.88086	15024.08496	15843.35059	17128.1582
Filtered	Filtered	Filtered	Filtered	15246.68848	Filtered	25648.42578	Filtered	Filtered	Filtered	Filtered	Filtered
12460.8291	11035.54688	10770.58984	8985.716797	44597.38672	5464.256836	42938.72656	7248.474121	7227.209961	Filtered	11728.28906	Filtered
10063.44922	11821.82031	10159.98047	18961.38477	25417.93359	22858.89453	10986.15234	6807.375977	Filtered	Filtered	Filtered	10210.64746
16011.11523	17179.14063	16155.70996	21942.54883	10228.32031	16549.51758	Filtered	17028.28516	15285.13867	Filtered	27475.96094	18302.72266
Filtered	Filtered	Filtered	Filtered	Filtered	18024.56055	Filtered	Filtered	Filtered	Filtered	Filtered	Filtered
6360.719238	20128.73828	8839.905273	9529.635742	19008.00781	31960.5	7996.058594	16960.97266	7386.449219	Filtered	15088.81445	5545.438477
16126.78906	26882.9668	Filtered	16330.8457	Filtered	17512.62109	14731.97559	22209.98438	13701.94824	Filtered	Filtered	24842.25195
Filtered	Filtered	1436.639038	Filtered	Filtered	Filtered	Filtered	Filtered	Filtered	Filtered	Filtered	Filtered
14546.23828	17304.47852	17636.18359	13142.27246	16691.44531	7926.263672	20844.76563	17051.60547	13541.46289	Filtered	28419.82422	14933.79492
26660.14648	17052.28516	12067.56836	14142.69727	11493.70508	17059.10938	18028.28125	12014.15234	19577.01563	Filtered	25941.29688	16587.75391
3047.083496	3843.388672	6373.051758	6660.31543	11253.10352	2740.111572	34110.4375	2929.192871	9356.138672	Filtered	13996.63965	6899.566406
42003.74219	13491.36816	18044.32422	8580.030273	11540.96875	11677.08105	8589.624023	30901.91602	24970.52539	74298.94531	13803.18945	30456.20703
12922.82813	10479.55469	27126.23633	16158.00195	13020.05566	21375.81445	17327.58789	10382.05176	16542.33594	Filtered	17463.46289	22753.65039
Filtered	Filtered	Filtered	Filtered	Filtered	Filtered	10618.97266	Filtered	3227.386963	Filtered	Filtered	Filtered
16332.29297	14037.30371	14480.42871	21084.86133	12864.84668	15011.81836	15877.68555	12435.75781	16429.39063	Filtered	15746.67578	30443.69141
18098.71094	11298.11426	Filtered	Filtered	8981.116211	9676.601563	12057.22559	20603.26172	12328.69531	Filtered	Filtered	Filtered
15633.46875	14198.88574	16355.15039	10115.15625	12296.26855	22777.37305	14151.24707	17724.61523	26088.56836	Filtered	13391.3252	19563.84375
3151.371094	2966.448242	Filtered	1988.594604	Filtered	Filtered	2650.503906	Filtered	Filtered	142565.3438	2678.536133	3475.813965
13382.37695	12347.83301	10488.05566	12878.62207	11336.36133	17647.41602	12357.58984	9339.270508	12753.97363	Filtered	13358.39648	12871.87012
4147.453125	2572.090576	Filtered	3376.343994	80004.375	Filtered	Filtered	3692.630615	2863.774902	86072.69531	3204.409424	Filtered
20659.12305	15274.58105	13770.29492	13485.08105	9453.435547	15382.59277	17015.14844	15685.84863	11577.55273	Filtered	18989.56836	18756.36328
11723.09375	4082.125	4595.007324	Filtered	Filtered	5063.322754	Filtered	Filtered	Filtered	Filtered	6437.771484	Filtered
16930.77148	11947.96289	11860.57422	9164.821289	17878.5918	15290.54688	9148.831055	9911.895508	12600.36328	Filtered	12409.84766	13002.48047
Filtered	Filtered	Filtered	Filtered	Filtered	Filtered	Filtered	Filtered	Filtered	Filtered	Filtered	Filtered
Filtered	Filtered	Filtered	Filtered	Filtered	Filtered	Filtered	Filtered	Filtered	Filtered	Filtered	Filtered
19173.5332	18805.44336	8328.383789	26050.52539	19143.16016	15499.24121	12301.21973	11392.96094	13882.7627	Filtered	16020.17871	19708.3457
8988.896484	11377.88477	16608.82813	11615.84766	8657.024414	11954.73828	7648.850098	13353.06152	13916.85254	29675.0332	11476.97754	30942.13477
13603.84863	14359.00488	8850.553711	25910.49219	16377.87109	21235.57617	15717.13477	14824.5	17037.48438	Filtered	16551.68359	14403.43848
31599.42578	18066.52539	14909.72363	11821.65039	7291.412598	18032.98047	18567.61328	13601.19531	16165.0459	21092.55664	22827.78516	23850.7793
21777.76172	16082.54297	9709.9375	22976.77539	16411.38672	22670.25781	8056.955566	11020.37305	20482.46484	Filtered	15769.25781	11123.07031
Filtered	Filtered	Filtered	Filtered	Filtered	3148.51709	Filtered	Filtered	3509.047607	Filtered	Filtered	Filtered
16013.43164	12498.8252	14973.07715	22111.97656	9559.001953	20004.33594	26213.25	12919.11523	29469.8457	Filtered	16103.50977	18068.32422
15802.58789	11475.36816	12390.15332	12166.97852	8970.484375	12807.57617	8173.314941	11901.74414	10505.09961	111474.2578	18374.8418	12257.34766
15580.06836	20753.35547	17820.4707	18809.00195	16415.14648	14589.40234	12636.20996	Filtered	12439.59473	Filtered	18500.34766	20677.15625
Filtered	12778.68555	Filtered	Filtered	30930.89453	4724.499023	40145.63672	Filtered	Filtered	Filtered	12970.3125	7611.222656
19790.12891	14939.82422	13030.45801	16734.54297	15108.38281	15836.05078	10754.38867	13570.09668	13994.50195	Filtered	13391.18164	16728.28516
13151.27344	Filtered	3763.768066	Filtered	13189.47559	Filtered	34964.52344	Filtered	6608.519531	Filtered	5796.816895	Filtered
Filtered	6806.182617	8840.650391	7557.353027	23586.63867	8104.46875	28108.59766	7045.507813	Filtered	Filtered	9605.40918	8042.495605
15971.90527	7916.107422	12362.12012	13166.69141	8583.696289	12595.11035	23934.89648	9630.59375	23048.55469	172385.2813	12350.78613	18808.46094
14022.64648	18557.82422	12338.93652	16839.29688	14450.96973	21896.9707	Filtered	18654.86719	16342.02637	Filtered	8150.317383	19224
13113.67383	26053.62109	15867.3457	9243.503906	13918.27832	13295.8252	13106.19824	12457.48633	10039.70215	Filtered	8277.060547	12145.8291
15385.82617	14550.86328	11421.01855	21333.5332	18654.30469	18601.78125	16453.41797	7603.821289	14780.99512	Filtered	14405.46973	15598.70898
Filtered	Filtered	Filtered	5213.907227	9927.119141	Filtered	22625.54492	Filtered	Filtered	Filtered	Filtered	Filtered
11629.80176	11924.96191	Filtered	Filtered	Filtered	Filtered	12887.93164	Filtered	12442.44629	Filtered	7292.005371	14077.11719
Filtered	Filtered	Filtered	Filtered	Filtered	Filtered	15117.13672	Filtered	Filtered	Filtered	Filtered	Filtered
Filtered	Filtered	Filtered	Filtered	8249.219727	Filtered	3213.92627	Filtered	Filtered	Filtered	Filtered	Filtered
9817.922852	Filtered	25344.05469	Filtered	Filtered	Filtered	Filtered	23706.7832	16201.95508	Filtered	Filtered	Filtered
15028.71973	Filtered	Filtered	Filtered	Filtered	Filtered	Filtered	Filtered	14887.48438	Filtered	Filtered	Filtered
12180.74023	13712.19238	10540.69727	21803.70898	12184.93457	15326.18262	12550.09375	10782.28613	13434.32129	Filtered	11516.84668	17866.84961
26511.52734	9741.120117	8072.225586	Filtered	Filtered	Filtered	4457.647949	Filtered	Filtered	Filtered	Filtered	Filtered
11898.36426	16641.26172	11763.22461	16499.54492	12177.50684	19376.05469	14899.44629	10954.72754	14365.4873	Filtered	12434.6543	16224.00879
13342.48047	Filtered	Filtered	Filtered	21325.88867	3291.452637	Filtered	13600.6543	12932.9834	27102.67383	7908.254395	23563.37109

Filtered	Filtered	Filtered	3990.637451	Filtered	2659.711914	3910.159912	Filtered	4088.472412	Filtered	1671.812378	3758.464111
Filtered	Filtered	Filtered	Filtered	Filtered	Filtered	Filtered	Filtered	Filtered	Filtered	Filtered	Filtered
Filtered	15953.82227	12362.98828	16496.92969	12557.35156	6693.873535	Filtered	14241.25391	11186.06836	Filtered	12565.8457	14281.40137
21393.88477	11024.87988	12324.92188	14321.64453	12064.99023	10996.83301	12678.08789	12868.87109	17440.09375	Filtered	15529.54785	10239.26367
9683.512695	Filtered	Filtered	Filtered	5147.348633	5047.370117	4289.05127	Filtered	7539.757813	Filtered	Filtered	Filtered
14351.45508	10358.05664	7157.535645	13261.47363	36987.59375	5456.451172	30084.74805	11422.96875	7143.812988	Filtered	15405.80371	22520.70117
17530.85938	19814.5	8807.837891	9567.873047	16748.13477	15302.74707	5951.462402	14670.63965	16313.87598	Filtered	Filtered	14322.32715
14633.93359	14661.62305	14809.43262	12753.92969	13229.33789	15824.84961	11302.6123	13293.21484	14541.64063	Filtered	11731.51074	17994.80469
16249.98438	13754.74316	13079.16309	12970.85059	12862.28418	13309.17285	12315.08691	11169.17773	13937.9043	Filtered	14453.8125	12692.79297
14233.18164	16770.88281	9184.990234	13157.48242	9517.360352	16485.91211	13237.42285	13379.13184	15914.72266	Filtered	11865.96094	16371.26465
12513.44336	16251.10059	12048.39258	10063.29785	41112.25391	11451.61816	22797.12305	7958.529297	5071.356445	15635.2998	Filtered	5541.316895
Filtered	Filtered	1.50E+04	5.73E+03	Filtered	Filtered	Filtered	5.19E+03	3.52E+03	Filtered	Filtered	Filtered
9650.786133	14628.18457	13980.43457	16464.46484	13801.06445	15256.67969	12492.22168	10299.26758	12520.34277	Filtered	9456.238281	15422.06445
5967.992188	Filtered	Filtered	Filtered	Filtered	Filtered	Filtered	Filtered	Filtered	48085.60547	8286.694336	Filtered
12633.36133	13964.15234	14708.0166	6615.19043	10638.78906	18263	11048.32227	11332.51074	15714.71582	Filtered	13706.62988	16613.32422
14911.75	Filtered	16242.05859	12246.96875	Filtered	Filtered	11007.92285	18698.95898	Filtered	Filtered	12016.52051	10833.22754
Filtered	Filtered	Filtered	13422.41797	Filtered	Filtered	Filtered	Filtered	Filtered	Filtered	23727.52344	17841.62109
7806.021973	5024.479004	8969.230469	8157.984375	7845.543457	5646.549805	7086.020508	5382.283203	9599.822266	Filtered	8228.423828	6703.092773
15503.92188	14999.12012	16716.5332	11998.84375	11473.44922	13179.87207	7742.312988	15300.08789	10877.75586	Filtered	12143.60156	13044.93945
Filtered	3864.319824	1995.583008	6192.687012	12445.04199	Filtered	21675.97656	Filtered	4282.53125	Filtered	8889.712891	Filtered
11812.21875	Filtered	Filtered	Filtered	Filtered	5230.118652	45678.92188	Filtered	Filtered	64312.34375	Filtered	Filtered
Filtered	3896.051514	Filtered	1766.874878	2375.560303	4185.538574	6836.40332	Filtered	Filtered	53306.55078	Filtered	5096.133789
Filtered	11149.91211	Filtered	Filtered	Filtered	Filtered	Filtered	Filtered	Filtered	Filtered	Filtered	Filtered
9642.874023	17407.77734	12349.19531	6474.325195	16258.10156	10331.61523	12730.60742	12484.57227	6035.021484	Filtered	6438.21582	12836.43359
Filtered	Filtered	Filtered	Filtered	Filtered	Filtered	11732.34961	Filtered	Filtered	Filtered	Filtered	Filtered
Filtered	Filtered	Filtered	5897.458984	11571.58594	Filtered	22261.02148	Filtered	Filtered	Filtered	Filtered	Filtered
7210.783691	11023.72852	8923.282227	Filtered	Filtered	8713.067383	6273.693848	57034.56641	7164.219238	Filtered	70125.125	7825.392578
10672.22852	12998.06445	8812.421875	16619.50195	10344.20508	13894.33105	7040.035645	7816.220215	10396.00391	Filtered	11469.64453	11735.5166
13184.64746	16488.90625	15771.48438	16398.08008	12137.53906	13138.77148	11382.40527	8926.895508	11567.44141	Filtered	12369.9834	12358.68457
8768.24707	5780.833008	14002.87793	Filtered	Filtered	Filtered	Filtered	11322.38574	22334.67969	Filtered	3955.382813	13300.13867
13798.04102	11089.1416	9576.914063	10543.21289	13797.41211	12432.50293	12242.35352	9305.573242	12532.69043	83916.71094	10145.47266	12743.14551
Filtered	Filtered	Filtered	Filtered	Filtered	Filtered	Filtered	Filtered	Filtered	Filtered	Filtered	Filtered
16355.92188	19896.20703	4929.775879	14553.15234	10649.52539	11083.50781	4114.775391	7145.755859	5858.561035	Filtered	4870.263184	7760.577637
12257.0957	Filtered	Filtered	8066.65625	10607.16016	21355.60742	12925.72754	5810.682129	7536.365723	Filtered	6013.973633	Filtered
13496.81543	8983.068359	12299.57422	17780.5332	16283.36426	15196.12305	8061.651367	9445.943359	12153.70508	Filtered	9244.660156	10314.38281
Filtered	14781.04395	Filtered	9713.735352	12523.83691	19567.88086	Filtered	17125.54492	15675.7793	Filtered	Filtered	Filtered
Filtered	11454.06543	1558.470825	3258.184814	15394.16016	8113.555664	15153.46484	9288.186523	8817.026367	Filtered	25588.85547	5352.267578
Filtered	Filtered	2633.277832	3060.526367	2700.731201	2080.749023	3609.493652	Filtered	1703.521606	Filtered	1758.721924	Filtered
3115.629883	3606.647461	5621.172852	7026.286621	4531.70166	5986.876465	3197.649902	Filtered	4225.81543	Filtered	4482.669922	3826.033447
13589.6709	9667.017578	12049.16992	13714.23926	8565.029297	16520.40039	18327.29102	11905.15137	16174.7334	90932.10938	9411.986328	17159.26367
Filtered	Filtered	Filtered	Filtered	Filtered	Filtered	Filtered	Filtered	Filtered	Filtered	Filtered	Filtered
5775.259277	8006.191406	Filtered	2759.754639	3251.386475	6552.763672	10372.23633	7034.258789	Filtered	Filtered	Filtered	Filtered
Filtered	Filtered	Filtered	Filtered	Filtered	Filtered	Filtered	Filtered	Filtered	Filtered	Filtered	Filtered
17034.10938	14540.4082	12618.56055	9646.296875	11543.46094	10814.53906	Filtered	11586.76953	11646.05078	Filtered	8397.208984	12854.69531
Filtered	Filtered	Filtered	Filtered	Filtered	Filtered	Filtered	Filtered	Filtered	Filtered	Filtered	Filtered
Filtered	Filtered	4924.26709	4745.154785	Filtered	13793.94238	Filtered	Filtered	Filtered	Filtered	Filtered	24346.35156
Filtered	7101.239746	Filtered	11238.87598	9513.12207	Filtered	23929.49023	Filtered	Filtered	Filtered	Filtered	Filtered
12052.6582	11502.7207	19697.26172	11166.69824	3277.455566	7298.036621	13486.61914	18209.04883	63108.05078	Filtered	12501.16797	31759.38672
13355.41797	10448.67969	13239.25977	14317.95703	8954.807617	13933.78125	25269.58594	7658.078125	25037.39063	Filtered	7596.776855	22822.92383
11050.92285	9227.851563	10730.37598	14435.03809	10599.10742	20546.78906	9718.310547	10838.82324	10454.87402	Filtered	4411.821289	9885.544922
3190.38916	8739.702148	10099.54492	9939.643555	9591.698242	11381.14844	13887.26367	6230.519531	12353.95898	Filtered	10356.88379	9302.611328
16830.47656	10141.58203	8387.288086	13881.16211	10111.46484	15249.25586	10001.51465	10603.28516	10244.82324	Filtered	6720.291016	9321.170898
9718.541992	7530.866699	6383.445801	6343.211426	7952.20459	12425.11914	8647.59668	9259.179688	10578.4375	Filtered	11665.26953	6531.375488
11540.23828	12826.87305	8375.033203	4829.980469	10511.0332	11662.48633	12903.94434	17164.6875	12059.93555	Filtered	11084.91602	12497.55078
18225.65039	Filtered	1876.010742	12375.81738	Filtered	Filtered	Filtered	Filtered	6008.401367	Filtered	Filtered	3354.314453
10145.09375	14025.5293	8216.283203	11588.78125	9981.015625	12021.91406	9013.69043	9029.696289	11536.59375	Filtered	9174.003906	6960.940918
9751.3125	8829.386719	16522.38867	Filtered	Filtered	Filtered	Filtered	6418.313477	Filtered	Filtered	15612.50879	Filtered
12185.36523	9440.696289	8600.666992	5400.830566	6912.635254	11247.73242	5253.525879	12214.06738	13772.30566	Filtered	12484.77832	5318.447266
7538.831055	1906.547363	21882.23047	Filtered	Filtered	10266.69336	Filtered	3440.690674	3274.632324	Filtered	Filtered	2626.011963
13302.82715	8210.96875	13161.83594	11834.03027	8261.359375	15441.2168	21755.00195	10541.60938	15133.61133	Filtered	4351.385254	12833.7627
Filtered	Filtered	Filtered	Filtered	Filtered	5912.507324	7138.394531	Filtered	Filtered	84025.76563	11781.37305	Filtered
Filtered	Filtered	Filtered	5078.092773	5748.205566	Filtered	Filtered	Filtered	2913.179199	Filtered	Filtered	2236.692627
10783.07617	8906.099609	11288.3418	8119.055176	9982.685547	10769.41602	9215.894531	9198.660156	10773.79199	Filtered	9656.31543	10441.18164
Filtered	Filtered	2891.505615	3656.423584	46808.82813	3803.381348	4358.875	47312.62109	4051.171143	Filtered	Filtered	63717.31641
9091.72168	10591.08398	11711.70605	9741.724609	7975.522461	15200.10254	9486.490234	9217.914063	8967.294922	Filtered	11688.36719	8428.638672
9263.19043	Filtered	Filtered	2039.829224	Filtered	2136.594727	Filtered	Filtered	Filtered	Filtered	Filtered	7459.072754
22704.60352	8374.351563	10206.08789	4690.202637	1619.252441	6983.290039	4955.147461	Filtered	Filtered	Filtered	7682.724121	7820.950195
9303.963867	8445.230469	9740.849609	17354.4043	10227.98438	10715.00977	9719.174805	9019.811523	6702.382324	Filtered	6251.561523	14017.10156
Filtered	11485.33105	10468.04102	12230.19922	4781.054688	Filtered	Filtered	Filtered	Filtered	Filtered	Filtered	Filtered
12582.76563	5583.224121	6523.050293	13767.48145	8101.055664	15992.2168	19836.89063	6666.773926	19548.74805	Filtered	3562.793945	8192.398438
4841.58252	4941.671875	6052.770996	5688.071289	3510.635986	5105.588867	5229.005371	5550.297852	5185.34082	Filtered	4768.345703	4197.391113
11183.66016	7364.797363	7817.370117	20131.23828	24633.13867	10990.06836	6379.269043	10476.16504	14729.28809	Filtered	9440.242188	7816.59082
Filtered	Filtered	Filtered	Filtered	Filtered	Filtered	Filtered	Filtered	Filtered	Filtered	Filtered	Filtered
Filtered	Filtered	Filtered	Filtered	Filtered	Filtered	Filtered	Filtered	Filtered	Filtered	Filtered	Filtered
Filtered	Filtered	Filtered	Filtered	Filtered	Filtered	Filtered	Filtered	Filtered	Filtered	Filtered	Filtered

Filtered	10575.04688	Filtered	10141.94727	7144.325684	16010.14648	3424.217529	Filtered	Filtered	Filtered	Filtered	Filtered
Filtered	2713.720459	8093.240723	11134.54297	7896.77832	10490.26953	Filtered	Filtered	11622.75781	Filtered	Filtered	Filtered
9402.550781	6376.385742	6102.515625	10713.14355	6983.774902	Filtered	14114.29688	12587.67383	15887.78906	Filtered	6020.834961	Filtered
Filtered	Filtered	Filtered	3918.203125	Filtered	Filtered	Filtered	Filtered	Filtered	Filtered	Filtered	7314.288086
Filtered	Filtered	Filtered	Filtered	Filtered	Filtered	Filtered	Filtered	14177.58496	Filtered	Filtered	Filtered
8982.764648	7888.272461	Filtered	9913.088867	15087.5332	Filtered	Filtered	13150.32324	13695.70508	Filtered	6857.773438	Filtered
Filtered	Filtered	Filtered	Filtered	3402.94873	Filtered	Filtered	Filtered	Filtered	Filtered	Filtered	6761.753418
Filtered	Filtered	11076.96484	Filtered	Filtered	Filtered	Filtered	Filtered	Filtered	Filtered	18359.15625	Filtered
8942.017578	7151.45752	6944.381348	10749.24805	76804.39844	9669.574219	13981.13184	7796.602539	10205.84766	35131.32422	5578.864258	10079.85742
6693.724121	13296.66797	6166.537109	8944.400391	8704.790039	8939.198242	7245.428711	4576.101074	7154.481445	Filtered	6159.469238	5406.14209
6969.01123	8794.504883	6973.401367	12416.72168	8512.890625	Filtered	Filtered	Filtered	Filtered	Filtered	5986.664063	Filtered
8557.918945	Filtered	Filtered	6246.083008	Filtered	5676.285156	Filtered	2931.735107	9868.595703	Filtered	9848.273438	11223.20801
Filtered	Filtered	Filtered	Filtered	10622.29395	Filtered	Filtered	Filtered	Filtered	Filtered	Filtered	Filtered
10401.96387	12142.55566	10352.52148	12438.20996	6367.616211	8905.228516	4336.583496	2605.926514	Filtered	Filtered	11265.56055	7940.763672
8646.091797	8563.087891	5380.04541	7716.876465	7108.588379	8499.261719	8282.783203	7965.117676	6110.90332	87429.53906	14336.68457	9638.342773
6569.646973	8594.09375	7157.714355	10534.12207	11625.18066	7243.684082	12162.41211	Filtered	5842.530762	Filtered	Filtered	5638.265137
5032.214844	10389.44629	Filtered	Filtered	8977.652344	6602.544434	Filtered	9612.849609	8720.472656	Filtered	Filtered	4884.491211
9417.811523	10245.15039	7221.874023	14198.80469	7003.095215	4141.124512	11226.25293	10063.43359	6564.424805	Filtered	7093.120605	12026.16602
3221.179199	Filtered	1126.730103	2195.524658	2136.136963	4563.957031	4564.293945	Filtered	Filtered	Filtered	Filtered	Filtered
9294.791992	8457.536133	9237.860352	7305.066895	4849.999023	7972.097168	7979.754395	7857.597168	9794.838867	Filtered	Filtered	Filtered
12210.96484	Filtered	Filtered	Filtered	Filtered	Filtered	2056.300049	Filtered	Filtered	Filtered	8771.62207	9141.339844
Filtered	Filtered	Filtered	Filtered	Filtered	Filtered	Filtered	Filtered	Filtered	Filtered	Filtered	Filtered
3532.749756	5369.333496	Filtered	4599.557617	7083.102051	5782.915039	Filtered	Filtered	Filtered	48575.73047	Filtered	7165.126465
Filtered	8596.695313	7309.695313	Filtered	6950.238281	Filtered	Filtered	Filtered	Filtered	Filtered	Filtered	7944.915527
7021.977051	4728.07666	Filtered	14784.28125	Filtered	13082.30273	12171.09961	Filtered	6083.081543	Filtered	Filtered	Filtered
9452.182617	7407.26416	6917.889648	5105.856934	7857.335938	10792.99219	7139.736816	8016.192871	10253.04492	Filtered	5970.555664	7742.530762
Filtered	Filtered	Filtered	Filtered	Filtered	Filtered	Filtered	Filtered	Filtered	Filtered	Filtered	Filtered
10173.47852	Filtered	7335.67334	8459.576172	Filtered	7542.046387	9942.386719	Filtered	14332.89453	43395.51953	Filtered	4770.647461
10454.99609	Filtered	6277.660645	Filtered	Filtered	Filtered	6609.5	Filtered	Filtered	Filtered	12669.56836	Filtered
8999.027344	Filtered	9311.139648	7058.73877	Filtered	Filtered	Filtered	Filtered	Filtered	Filtered	Filtered	Filtered
Filtered	Filtered	9522.710938	Filtered	7324.933594	9192.661133	Filtered	Filtered	Filtered	Filtered	Filtered	Filtered
Filtered	Filtered	Filtered	Filtered	6572.598145	Filtered	10487.55469	6429.331055	10216.56445	Filtered	Filtered	9478.709961
Filtered	Filtered	Filtered	Filtered	Filtered	Filtered	7416.311035	Filtered	Filtered	Filtered	Filtered	Filtered
8750.130859	9433.464844	6642.041992	8056.689941	Filtered	9695.015625	7707.674805	6749.575195	3383.520508	Filtered	6782.745117	9482.618164
Filtered	4554.169434	5341.390137	5729.641113	5960.226563	8937.271484	3657.553955	Filtered	8310.310547	Filtered	15012.97559	14351.44141
9869.498047	10948.41211	7523.435059	4622.700195	6233.66748	9136.175781	4988.461914	6974.741211	12477.56738	Filtered	8848.594727	8367.677734
5779.541016	5879.801758	9313.091797	7340.149414	6773.699219	10158.81738	8827.499023	6197.722168	9217.700195	Filtered	7668.692383	10061.37305
Filtered	1901.717407	8879.470703	10193.80859	8613.257813	11810.20605	3353.427246	Filtered	2803.832031	Filtered	5798.026367	1831.78064
6192.90918	Filtered	Filtered	Filtered	Filtered	Filtered	Filtered	Filtered	Filtered	Filtered	6946.25293	Filtered
5844.400391	7498.287109	7833.257324	6916.329102	7246.209961	9849.597656	6298.666992	6787.993164	8469.125977	Filtered	7824.496582	9797.344727
Filtered	Filtered	Filtered	Filtered	Filtered	Filtered	12415.80859	Filtered	8311.404297	Filtered	Filtered	Filtered
Filtered	5027.522461	Filtered	11008.29883	7376.830566	9097.071289	10334.9043	7608.78418	8662.337891	Filtered	4963.321777	7759.571289
8463.608398	4271.469727	6110.745605	4824.42627	Filtered	3745.518799	8334.839844	6705.909668	5443.399902	45398.36328	11067.50977	5243.181641
Filtered	Filtered	5940.501953	7369.76123	7880.854004	7341.986816	Filtered	Filtered	7798.774414	Filtered	Filtered	9263.819336
6866.287109	13683.15137	Filtered	3270.322754	9787.756836	4303.62793	Filtered	Filtered	Filtered	Filtered	Filtered	Filtered
Filtered	Filtered	Filtered	Filtered	Filtered	Filtered	Filtered	Filtered	Filtered	Filtered	Filtered	Filtered
7374.680176	Filtered	10056.42188	10806.41797	5394.649902	4332.450684	7670.525879	7637.669922	9822.21582	Filtered	7993.666016	8244.736328
Filtered	Filtered	Filtered	Filtered	Filtered	Filtered	9429.030273	Filtered	Filtered	Filtered	Filtered	Filtered
Filtered	6558.369629	Filtered	Filtered	6320.227539	7651.383789	Filtered	Filtered	7642.530273	Filtered	Filtered	7672.286621
1681.186157	2582.932617	2463.263428	Filtered	Filtered	178832.2969	Filtered	Filtered	Filtered	Filtered	Filtered	4624.954102
Filtered	Filtered	9159.307617	7321.608398	3303.702148	8573.173828	7496.091309	Filtered	5888.671387	46995.51563	8403.441406	6404.228027
10764.75	7534.237305	9179.166992	6551.618652	5936.917969	8368.498047	6848.576172	5764.239258	Filtered	Filtered	9649.740234	7543.355469
10652.57422	9506.524414	Filtered	12001.01465	Filtered	Filtered	7663.976563	4858.494141	6217.462891	Filtered	Filtered	Filtered
7664.790039	8964.488281	4519.960449	5624.399414	4678.424805	8188.840332	7368.8125	10979.73242	10236.59375	Filtered	Filtered	8570.756836
4523.427734	4267.164063	Filtered	3477.979736	6401.648438	11153.55664	4989.551758	4299.341797	Filtered	Filtered	2259.325684	Filtered
8369.708008	5965.388672	8774.922852	7406.816406	8297.757813	3087.396729	6817.675293	7581.351563	8169.805664	Filtered	9074.897461	7007.791016
11363.82129	5223.308594	6036.841309	9568.977539	4772.129883	11409.09766	6357.355469	13477.33984	8747.250977	Filtered	8180.477051	4890.152344
Filtered	Filtered	Filtered	Filtered	4772.836914	Filtered	Filtered	Filtered	Filtered	Filtered	1279.622314	Filtered
Filtered	Filtered	4251.452148	13466.60254	1954.422485	5980.519531	Filtered	8351.802734	6770.550781	Filtered	5549.950195	8500.90332
Filtered	Filtered	Filtered	Filtered	Filtered	Filtered	8277.547852	Filtered	Filtered	Filtered	Filtered	Filtered
4087.531738	4724.367676	9185.079102	5443.968262	4120.772949	5557.855469	11131.85449	3218.557861	9610.500977	Filtered	12870.28516	15506.10547
Filtered	Filtered	Filtered	Filtered	Filtered	Filtered	Filtered	Filtered	Filtered	Filtered	Filtered	Filtered
3623.656494	Filtered	5448.543945	Filtered	Filtered	Filtered	5713.968262	Filtered	1575.164185	70537.76563	3575.575439	4298.57373
7569.669922	6174.835938	8793.964844	7567.597168	8135.031738	9789.509766	6447.855469	6282.621094	7189.599609	Filtered	4837.390137	6684.12207
Filtered	Filtered	5899.830078	Filtered	Filtered	Filtered	11785.04199	6033.792969	9409.037109	Filtered	Filtered	Filtered
8316.136719	5613.803223	6510.210938	5865.602539	Filtered	Filtered	Filtered	Filtered	Filtered	Filtered	6438.620117	7840.512695
Filtered	Filtered	Filtered	Filtered	3979.539063	Filtered	6960.78418	Filtered	Filtered	Filtered	Filtered	Filtered
9502.280273	Filtered	6675.169922	5068.528809	6975.944824	5487.004395	Filtered	Filtered	6912.611328	Filtered	10142.27539	Filtered
Filtered	Filtered	Filtered	Filtered	4332.243652	5135.963867	6540.161133	Filtered	6202.938477	36113.96484	Filtered	Filtered
Filtered	Filtered	1665.899536	3754.230469	Filtered	Filtered	Filtered	Filtered	Filtered	Filtered	Filtered	5437.197266
11359.06641	2279.951904	Filtered	Filtered	9967.958008	3826.41333	6715.782227	5705.461426	6692.278809	Filtered	Filtered	14967.86426
8702.378906	6349.496094	6987.764648	7482.575195	5661.508301	5941.17041	7148.904785	Filtered	7594.331543	Filtered	6494.009277	4355.574707
7622.940918	4774.498047	4431.17041	8461.688477	7646.447754	6846.753418	7452.677246	5932.799316	8260.651367	Filtered	6491.688965	Filtered
Filtered	Filtered	Filtered	Filtered	Filtered	Filtered	Filtered	Filtered	Filtered	Filtered	Filtered	Filtered
Filtered	Filtered	Filtered	Filtered	Filtered	Filtered	Filtered	Filtered	Filtered	Filtered	Filtered	Filtered

4450.557617	6594.87207	Filtered	Filtered	7754.268066	5347.498047	7631.979492	5980.341309	Filtered	Filtered	Filtered	Filtered
6002.725586	Filtered	8771.775391	6577.606445	5920.22998	6415.948242	10981.11621	8215.165039	8642.427734	Filtered	Filtered	Filtered
7004.29541	7955.212891	9962.462891	7865.509766	9803.210938	7778.407227	7959.029785	5715.34082	6710.723633	Filtered	5830.794922	9134.956055
8330.479492	6099.646973	6874.46582	9607.84668	6621.168945	6779.026855	4954.726563	Filtered	6320.647461	Filtered	6825.878906	7609.543945
Filtered	Filtered	Filtered	Filtered	Filtered	Filtered	Filtered	Filtered	Filtered	Filtered	Filtered	Filtered
Filtered	5982.706055	4900.187012	5827.228516	4935.349609	7560.661133	6604.849609	Filtered	Filtered	Filtered	Filtered	Filtered
Filtered	Filtered	Filtered	4956.979004	5246.298828	7024.804688	Filtered	Filtered	Filtered	Filtered	Filtered	Filtered
Filtered	Filtered	Filtered	1471.921143	Filtered	3339.324219	13896.52344	Filtered	12054.75586	Filtered	Filtered	Filtered
Filtered	2204.275391	3009.067139	Filtered	Filtered	Filtered	Filtered	Filtered	Filtered	Filtered	9137.220703	Filtered
Filtered	Filtered	Filtered	Filtered	Filtered	Filtered	3147.209717	Filtered	Filtered	Filtered	Filtered	Filtered
Filtered	1638.516357	4486.996094	Filtered	Filtered	Filtered	5899.4375	Filtered	10033.49609	Filtered	Filtered	5331.199219
Filtered	Filtered	Filtered	Filtered	Filtered	Filtered	Filtered	Filtered	Filtered	Filtered	Filtered	Filtered
2738.615967	3681.102783	2114.112061	1791.268799	8346.067383	Filtered	14687.42285	Filtered	4130.324219	Filtered	Filtered	7638.475586
Filtered	3698.049316	Filtered	Filtered	Filtered	Filtered	Filtered	Filtered	Filtered	Filtered	Filtered	Filtered
Filtered	Filtered	6477.316895	Filtered	Filtered	4765.241211	Filtered	Filtered	8358.196289	Filtered	Filtered	5565.035645
Filtered	5396.705078	7667.906738	8074.013184	Filtered	5190.222656	1471.735718	Filtered	7262.597656	Filtered	8261.75293	5633.328125
5721.287109	6964.714355	7870.444824	8065.807617	3550.516113	6895.527344	9473.810547	5396.45459	6389.533203	Filtered	8364.243164	7877.410645
Filtered	Filtered	Filtered	5102.802734	Filtered	5526.711426	Filtered	5435.598633	Filtered	Filtered	Filtered	Filtered
5883.750488	Filtered	4760.165527	Filtered	1079.273682	3925.518555	Filtered	6844.317383	14085.99121	Filtered	7476.510254	6668.036133
Filtered	Filtered	Filtered	Filtered	Filtered	Filtered	5730.762207	Filtered	5077.858887	Filtered	Filtered	Filtered
Filtered	Filtered	Filtered	2571.030273	Filtered	Filtered	6874.888672	Filtered	Filtered	Filtered	Filtered	Filtered
6447.144531	Filtered	Filtered	4145.595215	Filtered	6029.925781	Filtered	Filtered	3820.407471	Filtered	Filtered	Filtered
3874.869141	Filtered	5596.077637	1957.164673	2908.016357	Filtered	Filtered	Filtered	Filtered	Filtered	Filtered	Filtered
Filtered	Filtered	5892.557617	5977.565918	Filtered	Filtered	Filtered	Filtered	Filtered	Filtered	Filtered	3754.941406
Filtered	Filtered	Filtered	Filtered	Filtered	Filtered	3229.295898	Filtered	Filtered	Filtered	Filtered	Filtered
6934.101563	Filtered	Filtered	6092.460449	4654.126953	5397.41748	3091.634766	5879.128906	3795.057617	Filtered	2214.39502	2951.464355
Filtered	Filtered	Filtered	Filtered	5073.994141	Filtered	Filtered	Filtered	Filtered	Filtered	Filtered	Filtered
Filtered	Filtered	Filtered	Filtered	Filtered	Filtered	Filtered	Filtered	Filtered	Filtered	Filtered	Filtered
4689.957031	5660.46875	Filtered	Filtered	Filtered	7100.818359	Filtered	3782.908447	4182.104004	Filtered	Filtered	Filtered
Filtered	Filtered	4861.520996	Filtered	Filtered	Filtered	Filtered	Filtered	3129.857666	Filtered	Filtered	Filtered
5619.647461	Filtered	Filtered	Filtered	Filtered	4421.081055	4032.415283	3221.656738	Filtered	Filtered	Filtered	Filtered
10484.34375	3696.574219	4850.458984	3704.943848	5080.475098	6265.571289	6163.856934	6026.589844	12004.54688	Filtered	10078.66309	4518.338379
4805.473633	7534.452148	3919.002441	Filtered	3821.818359	3661.072266	Filtered	Filtered	9493.444336	Filtered	Filtered	Filtered
3841.359863	Filtered	Filtered	Filtered	Filtered	Filtered	5052.880859	Filtered	Filtered	Filtered	8006.916016	Filtered
Filtered	Filtered	Filtered	Filtered	Filtered	3138.279785	8257.948242	Filtered	Filtered	Filtered	Filtered	Filtered
6532.609863	5722.8125	8382.129883	6415.170898	3064.179688	6930.431152	8638.353516	5085.825684	6417.857422	Filtered	4030.783447	8902.375977
Filtered	Filtered	Filtered	Filtered	Filtered	6399.725098	Filtered	Filtered	Filtered	Filtered	Filtered	4186.729004
Filtered	5842.850586	8133.697266	5741.445313	Filtered	5504.875	Filtered	Filtered	Filtered	Filtered	Filtered	Filtered
5553.883789	5548.392578	4776.352051	4919.36084	3217.01416	6594.876953	3737.171387	5508.496094	7040.469727	Filtered	6788.505859	3085.359619
Filtered	Filtered	Filtered	Filtered	Filtered	5829.083984	Filtered	Filtered	12459.95703	Filtered	Filtered	Filtered
4383.40625	Filtered	8462.904297	4069.026123	Filtered	3298.046143	8634.325195	Filtered	4611.344727	Filtered	Filtered	Filtered
6858.776855	4066.367188	Filtered	Filtered	Filtered	Filtered	Filtered	Filtered	Filtered	Filtered	Filtered	Filtered
Filtered	5648.825195	Filtered	Filtered	Filtered	Filtered	Filtered	Filtered	Filtered	Filtered	Filtered	Filtered
1851.788574	Filtered	Filtered	Filtered	30850.31641	4115.246094	Filtered	Filtered	5892.318359	Filtered	Filtered	Filtered
Filtered	Filtered	Filtered	Filtered	Filtered	Filtered	Filtered	Filtered	Filtered	Filtered	Filtered	Filtered
Filtered	Filtered	Filtered	Filtered	Filtered	Filtered	Filtered	Filtered	Filtered	Filtered	Filtered	Filtered
4668.336914	Filtered	Filtered	5373.81543	Filtered	2131.241943	Filtered	Filtered	Filtered	Filtered	Filtered	Filtered
2803.453125	Filtered	Filtered	3299.020752	3566.201172	3964.840576	5092.162109	Filtered	5687.911621	Filtered	Filtered	Filtered
Filtered	Filtered	Filtered	Filtered	Filtered	Filtered	10022.6709	Filtered	Filtered	Filtered	Filtered	Filtered
6134.427734	1314.782715	Filtered	Filtered	Filtered	Filtered	Filtered	3485.968262	Filtered	30414.11719	6673.913086	Filtered
4617.449707	2637.150635	4217.919922	7740.287598	3432.416748	6724.379883	5028.231934	7664.325195	6330.54541	Filtered	Filtered	4213.264648
Filtered	Filtered	Filtered	Filtered	7282.599121	9422.162109	Filtered	Filtered	Filtered	Filtered	Filtered	Filtered
6296.25	Filtered	5298.220703	5337.253906	Filtered	Filtered	6913.404297	4461.60498	7222.023438	Filtered	5817.397949	5863.97168
8425.998047	Filtered	1998.795288	3105.995361	2500.539551	7376.06543	4668.540527	3790.290527	Filtered	Filtered	6250.801758	Filtered
5228.568359	3006.80835	5890.102051	6686.640137	5866.548828	5143.992188	2652.035156	2453.664307	Filtered	Filtered	5267.830078	7857.491699
Filtered	Filtered	Filtered	Filtered	Filtered	Filtered	Filtered	Filtered	Filtered	Filtered	Filtered	Filtered
Filtered	Filtered	Filtered	Filtered	Filtered	Filtered	6012.34668	Filtered	Filtered	Filtered	Filtered	Filtered
3568.032715	5616.605957	3596.5271	5106.179688	5495.003906	4173.337402	7721.84668	3084.465576	4835.212891	Filtered	3478.005127	6457.246094
Filtered	6379.012695	5321.844727	4735.638672	Filtered	Filtered	2259.345947	Filtered	Filtered	Filtered	Filtered	Filtered
7947.279785	4007.354492	4395.040527	6518.036621	Filtered	6007.016113	4487.567383	4854.985352	5382.354004	Filtered	Filtered	6104.926758
3793.250244	Filtered	4337.055176	Filtered	Filtered	Filtered	Filtered	3590.700195	Filtered	Filtered	Filtered	Filtered
Filtered	2613.206055	4160.269043	1759.921753	2336.911133	4375.414551	7417.233398	Filtered	6899.0625	Filtered	Filtered	Filtered
Filtered	Filtered	Filtered	2222.978027	4426.768066	12804.50879	1208.426514	Filtered	Filtered	Filtered	Filtered	Filtered
Filtered	Filtered	Filtered	Filtered	Filtered	Filtered	Filtered	1600.974487	Filtered	Filtered	Filtered	Filtered
Filtered	Filtered	Filtered	Filtered	Filtered	Filtered	Filtered	Filtered	Filtered	Filtered	Filtered	Filtered
7279.803223	3882.03418	4491.762207	2882.7229	2652.180664	3776.646484	5233.994629	6285.983398	6688.970703	Filtered	2018.088745	5250.188477
5127.928223	3342.444336	5164.162598	3512.290527	5251.882813	6046.101074	9979.501953	4505.616211	9806.654297	Filtered	Filtered	5667.943359
Filtered	Filtered	Filtered	Filtered	Filtered	Filtered	Filtered	Filtered	Filtered	Filtered	Filtered	Filtered
6820.623047	Filtered	4957.435547	3881.873047	5018.820801	3315.183594	Filtered	Filtered	5304.578125	Filtered	6181.674805	Filtered
4549.691895	4280.445313	Filtered	3397.608398	2552.709717	Filtered	Filtered	4904.16748	7322.757324	Filtered	Filtered	5988.849121
Filtered	4882.179688	Filtered	10819.46875	3806.227539	Filtered	3434.713135	Filtered	Filtered	Filtered	4447.890137	7473.20459
3991.680176	3877.992188	3897.88208	7187.624512	2984.622559	2758.602539	4494.590332	3703.09082	3676.158691	Filtered	3812.487305	3095.79126
Filtered	Filtered	Filtered	Filtered	Filtered	Filtered	2277.695068	Filtered	Filtered	Filtered	Filtered	Filtered
2686.435059	2589.106934	Filtered	Filtered	4398.684082	4268.021973	3845.473633	3802.71875	2973.029541	Filtered	2448.407959	4953.071289
Filtered	4535.504883	Filtered	Filtered	Filtered	4969.294434	3582.807861	Filtered	Filtered	Filtered	Filtered	Filtered

Filtered	Filtered	Filtered	Filtered	Filtered	Filtered	Filtered	Filtered	Filtered	Filtered	Filtered	Filtered
Filtered	Filtered	Filtered	Filtered	Filtered	Filtered	6221.896973	Filtered	Filtered	Filtered	Filtered	Filtered
Filtered	5579.624512	Filtered	Filtered	Filtered	Filtered	Filtered	Filtered	Filtered	Filtered	Filtered	Filtered
Filtered	6575.996582	Filtered	Filtered	Filtered	Filtered	Filtered	Filtered	Filtered	Filtered	Filtered	Filtered
Filtered	2123.718262	Filtered	Filtered	Filtered	Filtered	5367.668945	Filtered	3586.098389	Filtered	Filtered	Filtered
Filtered	Filtered	Filtered	Filtered	Filtered	Filtered	Filtered	Filtered	Filtered	Filtered	Filtered	Filtered
Filtered	Filtered	Filtered	Filtered	Filtered	Filtered	Filtered	Filtered	Filtered	Filtered	Filtered	Filtered
Filtered	Filtered	Filtered	Filtered	Filtered	Filtered	Filtered	Filtered	Filtered	Filtered	Filtered	Filtered
6703.851563	4073.921875	Filtered	1183.339478	3757.269043	2759.054443	Filtered	2818.172852	Filtered	Filtered	6844.630371	Filtered
Filtered	Filtered	2750.842285	Filtered	2395.52417	Filtered	Filtered	Filtered	3413.898193	Filtered	3813.507324	Filtered
Filtered	2713.796875	2407.911621	Filtered	Filtered	Filtered	3156.04248	Filtered	Filtered	Filtered	2284.198486	2782.695068
Filtered	Filtered	Filtered	Filtered	Filtered	Filtered	Filtered	Filtered	Filtered	Filtered	Filtered	Filtered
Filtered	4084.942871	3110.199951	2700.467285	3658.563965	Filtered	Filtered	3633.952637	6574.566895	Filtered	4818.791016	3373.661621
Filtered	Filtered	Filtered	Filtered	3187.064209	3894.891602	Filtered	Filtered	Filtered	Filtered	Filtered	2216.435303
Filtered	Filtered	Filtered	Filtered	Filtered	Filtered	Filtered	Filtered	Filtered	Filtered	Filtered	Filtered
Filtered	Filtered	Filtered	Filtered	Filtered	Filtered	Filtered	Filtered	Filtered	Filtered	Filtered	Filtered
Filtered	3295.586182	4627.822266	2642.20874	4030.299561	14878.34766	5877.135742	2784.690186	Filtered	Filtered	Filtered	Filtered
Filtered	Filtered	Filtered	Filtered	Filtered	Filtered	Filtered	Filtered	Filtered	Filtered	Filtered	Filtered
3234.47168	Filtered	Filtered	Filtered	Filtered	Filtered	Filtered	Filtered	Filtered	Filtered	Filtered	Filtered
Filtered	Filtered	Filtered	2569.887207	Filtered	Filtered	Filtered	Filtered	5603.28125	Filtered	8510.415039	Filtered
Filtered	2199.3396	2531.037109	Filtered	Filtered	Filtered	5516.604004	2505.422363	Filtered	Filtered	Filtered	4683.861816
Filtered	Filtered	Filtered	2184.591309	3244.84668	3313.574219	2773.731689	Filtered	Filtered	Filtered	Filtered	Filtered
4968.719238	Filtered	3442.346436	4030.837646	3025.064453	3897.353516	3025.593506	2872.41748	3390.013184	Filtered	2706.530273	2995.577637
Filtered	Filtered	Filtered	Filtered	Filtered	Filtered	Filtered	Filtered	Filtered	Filtered	Filtered	Filtered
Filtered	Filtered	Filtered	Filtered	Filtered	Filtered	6566.924805	Filtered	Filtered	Filtered	Filtered	Filtered
Filtered	3856.716553	3297.445557	5078.611816	2719.780762	3255.824219	3078.174805	Filtered	4631.228516	Filtered	2525.265869	3040.976563
Filtered	Filtered	Filtered	Filtered	Filtered	Filtered	Filtered	Filtered	Filtered	Filtered	Filtered	Filtered
Filtered	Filtered	Filtered	Filtered	Filtered	Filtered	Filtered	Filtered	Filtered	Filtered	Filtered	Filtered
Filtered	Filtered	Filtered	Filtered	Filtered	Filtered	Filtered	Filtered	Filtered	Filtered	Filtered	Filtered
6338.597656	6574.187988	3069.151611	5750.490723	4289.431641	5231.975586	3721.052002	Filtered	3438.292725	Filtered	Filtered	3115.896973
Filtered	Filtered	Filtered	3002.287109	Filtered	3752.5625	Filtered	Filtered	Filtered	Filtered	Filtered	Filtered
Filtered	3933.133545	Filtered	Filtered	2572.650146	3999.997559	Filtered	Filtered	2911.992432	Filtered	Filtered	2476.53833
2253.305664	4054.820068	2770.896973	2621.930664	2728.62207	3213.828857	3148.17749	2608.070801	3917.748291	Filtered	Filtered	5066.985352
3735.296631	Filtered	Filtered	Filtered	Filtered	4358.669922	Filtered	Filtered	Filtered	Filtered	Filtered	Filtered
Filtered	Filtered	1600.06958	1700.135742	2594.471436	3156.790527	Filtered	Filtered	Filtered	Filtered	Filtered	Filtered
Filtered	Filtered	Filtered	Filtered	Filtered	775.4846191	Filtered	Filtered	Filtered	Filtered	Filtered	Filtered
Filtered	Filtered	Filtered	Filtered	Filtered	Filtered	Filtered	Filtered	Filtered	Filtered	Filtered	Filtered
Filtered	Filtered	3840.585693	Filtered	Filtered	Filtered	2718.63208	Filtered	Filtered	Filtered	Filtered	Filtered
Filtered	Filtered	Filtered	Filtered	Filtered	1247.510864	Filtered	Filtered	Filtered	Filtered	Filtered	Filtered
Filtered	Filtered	Filtered	Filtered	Filtered	Filtered	Filtered	Filtered	Filtered	Filtered	Filtered	Filtered
Filtered	Filtered	Filtered	Filtered	Filtered	Filtered	Filtered	Filtered	Filtered	Filtered	Filtered	Filtered
Filtered	Filtered	Filtered	Filtered	Filtered	Filtered	3889.193115	Filtered	1561.108887	Filtered	3056.946289	2486.861328
Filtered	Filtered	Filtered	Filtered	Filtered	Filtered	Filtered	Filtered	Filtered	Filtered	Filtered	Filtered
Filtered	Filtered	Filtered	Filtered	Filtered	Filtered	Filtered	Filtered	Filtered	Filtered	Filtered	Filtered
Filtered	Filtered	Filtered	Filtered	Filtered	Filtered	Filtered	Filtered	Filtered	Filtered	Filtered	Filtered
Filtered	Filtered	Filtered	Filtered	Filtered	Filtered	Filtered	Filtered	Filtered	Filtered	Filtered	Filtered
Filtered	Filtered	Filtered	Filtered	1676.920898	Filtered	Filtered	Filtered	Filtered	Filtered	Filtered	Filtered
Filtered	Filtered	Filtered	3130.772705	Filtered	Filtered	1797.876221	Filtered	2904.568115	Filtered	Filtered	Filtered
Filtered	Filtered	Filtered	Filtered	Filtered	Filtered	Filtered	Filtered	Filtered	Filtered	Filtered	Filtered
Filtered	Filtered	3525.914307	Filtered	Filtered	1457.46814	Filtered	Filtered	4556.568359	Filtered	Filtered	Filtered
Filtered	Filtered	Filtered	Filtered	Filtered	Filtered	Filtered	Filtered	Filtered	Filtered	Filtered	Filtered
Filtered	Filtered	Filtered	Filtered	Filtered	2150.642822	Filtered	Filtered	Filtered	Filtered	Filtered	Filtered
Filtered	Filtered	Filtered	Filtered	Filtered	Filtered	Filtered	Filtered	Filtered	Filtered	Filtered	1779.609497
1937.187744	1374.493896	1760.126709	1369.44043	Filtered	1244.6604	4840.884766	2026.445557	3107.922852	Filtered	2459.848145	3714.449707
Filtered	Filtered	Filtered	Filtered	Filtered	Filtered	Filtered	Filtered	Filtered	Filtered	Filtered	Filtered
Filtered	Filtered	Filtered	Filtered	Filtered	Filtered	Filtered	Filtered	Filtered	Filtered	Filtered	Filtered
Filtered	Filtered	Filtered	Filtered	Filtered	Filtered	1622.054321	Filtered	Filtered	Filtered	Filtered	Filtered
2020.744507	Filtered	Filtered	Filtered	Filtered	Filtered	Filtered	Filtered	Filtered	Filtered	Filtered	Filtered
Filtered	Filtered	Filtered	Filtered	Filtered	Filtered	Filtered	Filtered	Filtered	Filtered	Filtered	Filtered
Filtered	Filtered	Filtered	Filtered	Filtered	Filtered	Filtered	Filtered	Filtered	Filtered	Filtered	Filtered
Filtered	Filtered	Filtered	Filtered	Filtered	Filtered	Filtered	Filtered	Filtered	Filtered	Filtered	Filtered

PCOS_13	PCOS_14	PCOS_15	PCOS_16	PCOS_17	PCOS_18	PCOS_19	PCOS_20	PCOS_21	PCOS_22	PCOS_23	PCOS_24
77629352	61659488	186983152	73637344	56204128	224035984	85717520	100599064	73244896	174001824	56920552	65786720
124329720	92413472	104104936	80946944	134013816	60477272	116668736	113955480	162414400	97633152	82893560	81951800
29096290	30445646	24698080	30602796	35499908	33344480	28220336	37675796	34382080	29815406	26852148	25824584
22858774	24124106	20321988	22847250	31612276	25806842	25216364	21645260	24230218	28997434	27056778	24085168
19947282	18301552	17254918	20843284	25370172	14890034	19894544	16827238	24209558	18852108	21900684	17992062
18606300	21225236	15764669	13174512	30634536	19922310	19709660	20344908	21968930	24223392	23664008	17078660
14015144	17065916	14420529	18646346	20637800	18765832	17253718	18452080	18009584	21024864	20902764	16735517
10933288	13987261	16511918	11634974	19674316	10111518	16222479	19430662	25517968	14541319	12290209	12788020
17474490	14950091	10369719	15044528	12855248	14516709	12154957	13611482	15334289	14120630	17532834	12640836
11537163	13127994	12482751	9988782	16454085	11797112	13084322	15271277	15776155	14826252	14537861	11798953
13200162	12634604	6526621.5	12391039	11494810	13437559	9009941	10781220	12500435	11647723	13417930	10344768
14426471	11196135	5363430.5	8285869.5	11261237	3235875.5	13905899	9203280	13908133	7373928	12185262	22029148
11312134	10357252	7182316.5	8983850	14213792	7753586.5	9028171	12133477	10722272	10928874	9927014	9324365
9075353	10104181	8214804.5	8933860	7710433.5	9312090	7533716	11055784	10316441	10150920	10197356	7663092
10862164	8102581	3940378	9695517	9797909	10260756	11179127	8656966	8893004	9603603	11573196	11191179
8441473	6720416	8260343.5	7941525.5	9299389	6572620.5	7464519.5	10252472	12027522	9860618	10278264	8726915
9204758	9046386	7715366.5	10264357	9610795	6400821.5	9414775	8600065	11623493	8746178	9986428	7861170
6635679	9806907	6957277	9927884	9393595	8726526	8335475.5	10558322	7979572.5	10085356	9098541	8468458
6856507	7914304.5	7360739.5	7687713.5	6831675	9461452	7121117.5	8873132	8924750	8662156	8934238	6444835
41138.64063	13901334	32573628	35322.52734	11859334	92518.00781	17378598	53292.86719	37396.48047	111021.0547	11539942	8436915
7020703	6936847	7143014	9075879	8723288	7362176	7616721	10967611	7189485	7391764.5	9243996	8208214.5
7021535	5751388.5	6190065	6791042	9585236	7847981	6276220	7159120.5	6055964	6821848.5	6520132.5	5527607
8412356	7305324.5	4894098.5	9358134	7789661	8643262	9657954	5896375.5	8459858	10686727	9879236	9799213
7804144.5	5297203.5	5269094.5	5309036.5	7018410	6352496.5	5197662	5552865	7091528.5	7038835.5	7705406.5	14324962
8541442	5510659.5	5614865	6195246.5	7804736.5	6242557	6495558	7854116	8411866	6406960.5	6865810	5941659.5
6046112	6538658	6280255.5	7293634	6909750	6085395.5	6591457	7032728.5	7454545.5	7090074.5	4210754.5	5797394
5654484	6323346.5	5382668.5	5125257.5	6556308	4805427	6257967	7855692.5	7154526	5006898	6209109.5	6094524
6136084	6222742.5	4233068.5	5915657.5	7215158.5	5915182.5	5678160.5	6331874	5492618	5921261.5	4831574.5	4734747
4272663	6977965.5	4812439.5	6750584	4655602	6534993	6466134	8627517	5357280	8619775	4942511	6088853.5
8028873	4409945	4307439	3815731	4518080	3835942	4281288	5035195	6683833	4307090	4829868	5215195.5
3346100	3488279	6305225.5	2705088	10081682	2754193.75	5218947	9921551	6689211	5836161	7504812	5208334
4426626	4446808.5	4740022.5	5659304	6814107	5050637.5	4560611.5	6162240	4826890	5148788.5	6379577	5031415.5
4553207.5	5528607	4775470	5764132.5	4973458.5	4303880	4589842	5163399.5	6369279.5	4930419.5	4902482	4066543.25
2982546.25	4814947.5	4480319	4901306	3483802.25	3795893	4433327	5051746.5	5048857	5071117	5610193	5083463.5
5048723.5	4657666.5	4366212.5	4639475	5028668.5	3262592	4718266	4472780.5	3730327.75	5154247	5074455.5	5047187
2625639	1861626.25	5096034	2169280.5	1215299.875	8477207	2431420	3141138.5	1979253.875	7639665	1546162.875	1614879.5
3355952.5	3561072	3398208.25	3720304.25	2978092	3095466	3484036.5	3906130.25	4954023.5	3803678.25	3407766	3850938.25
3072989.75	3397169.25	4514075	2603455.5	5407709.5	2840945.5	3747891.25	3598283.5	5418441	4306208	4594495.5	3900296
3315537.25	2816999	3376029	3236279.5	3575236.25	2794131.25	3587021.5	3922323.25	4458365	4034490.75	3584709.25	2957716.75
Filtered	2901.003906	Filtered	Filtered	5583.675781	12459.59375	2671.159424	3520.615479	13001269	3517.972168	10837928	Filtered
2481270.75	4033450	3785632	5247686.5	5457855	1785304	1578344.75	4897021.5	5492236.5	2853050.25	5141083	3902295.25
2349967.25	2367287.25	4308783.5	2111924.75	3498471	1892858.875	3906774.5	4588252	5829842.5	3913455	3326495.25	2804053.5
3472036.5	3754195.5	2399788	3468601	5277378	3091635.75	3040668	2658020.5	5592858	3337177.5	4075585.5	2452264.25
3946255.75	5387741	3714935	1258065.875	4129298.25	4213592	3819287.75	868679.6875	1309026.75	4518307.5	1381013.75	4492275.5
3690757	2567855.75	2768597.5	2836126.75	3244896.25	2642597.75	2991301.5	4068151.5	3314111	3484448.75	3546330.5	2613905.25
3561878.25	3045980.75	2866719	3160214.5	3876580.75	3159512.5	2917887.75	3521774.5	2932353	2805411	2408133.25	2474850
3423844	2664829.25	2887004.25	3056671	3511846.5	2383537	2373662	3560785.75	2987943	2332629.25	2385067.75	2022293.125
1617968.5	1719335.25	3048664.75	1423015.875	1610151.75	4446397	1122259.75	1168569.125	1763041.375	2139737	1496279.75	1457467.25
1916684.75	3008802	2453992.75	2861779.5	1675315.875	1836061	2252403.5	2468657.75	2231461.25	2581474	3727846.5	2608319.5
2071115.5	3111248.75	2499863.5	3511280.5	2990398.5	4340486.5	2761191.5	2791929.25	1900865.5	3483820	2358482.5	1894129
3086420.25	2622543.25	2323627	2819804.5	3073980.25	2571650.75	2312894	3427132	2990081.75	2676459.5	2608642.5	2596782.5
2069936.375	3161137.25	2062472.625	2881569.5	2622081.75	3383939.25	2553464.5	2976681.75	2655905.25	3284071.5	3060622.5	2045139.375
2484209.5	2540447.5	3190433.5	2369629.75	2644942.25	1408725.25	2299694.5	3698839.75	3837823.75	2549730	2408261.5	1934808
2752771.5	2254135	9460598	1811655	2624395.25	6876255.5	1237409.625	1147327.375	1412763.25	1573936.375	1940841.25	1809793.75
2634622	2275634.5	2074721.875	1861599.75	2421633	2138632.75	2567858.25	2275933.75	2353431.25	2733729.5	1839800.75	2406888
1870675.125	2077039.625	1504873.75	1973641.875	2572926.25	2075784.625	2720745.25	2675877.5	2975863	2033354.5	2079195.625	2556586.5
2331585.5	2154603.5	1779785.875	2554702	2323441.25	2012458	2248709.5	2162891	2371724	2468091.5	2458090.25	2259270.75
2038226	2138742.25	1747968.75	2102525.75	1582665.25	1944373.125	1985997.875	2302325	2063482.25	1995767.125	1875665.375	2327106.5
1906242	1771056.125	1729733.375	2029822.875	1325203.625	1710037.75	2239981.75	2399487.25	1704117.125	1849499.375	1686261.875	1824735.25
2053058.375	2330162	1569362	2322044	1947055.625	3018885	2065206.375	1689727.125	1982052.5	2349014	2351551	1557650.75
1989045.875	1815378.375	1840202.75	2514837.5	2508519	1767483.75	2285547.75	2388409.75	2260729.75	2270086.25	2405165	2295480.5
1689271	1769918.875	4979224	1046608.813	1782256.25	5205889.5	952479.1875	1012407.938	1412359.375	908847.125	1367145.125	1691239.125
2184516	1928922.25	1658074.75	1782343.875	2136377	1792477.25	1948877.625	2285719.5	2367669	2395515.75	2092242.625	2085981.375
940644.25	1564589	10572522	758842.5	752234.8125	9604229	577776.8125	512811.125	279093.1875	667978.875	1413133	895140.0625
Filtered	1558440.75	1448024.625	1363238.875	Filtered	Filtered	Filtered	1938781.625	2401670.75	Filtered	2019105.625	3652308.5
1845010.875	1815815.625	736296.0625	2048390.125	1637040.625	1173861.75	2360884.5	2224364.5	2692655.25	1439263.5	3228262.25	2575400.25
2101112	1956901.625	247790.3281	2178232.5	1461084.25	2250990.25	1644129.625	2020463.75	2368673	2000080	2155756.75	1985262.5
1975430.875	1669216.125	1443549.5	1425483.75	1539914.75	1472306.875	1510589	1676869.25	1847798.625	2209849.5	1865497.875	1837219.125
3016761.75	1934935.5	1589095.75	1939904.625	2643889.25	960031.9375	2101408.5	2222314.5	3558625.25	1058986.5	2061353.625	1765372.25
2400886.75	2150120.75	1281090.875	1598953.125	1506433.625	2674708.75	1460133.125	1916347.125	2776168	1514161.25	2533376.5	1331728
1252386.5	1833687.375	1711895.625	1549939.125	2825169	1195509.5	1289887.75	2035354.625	2285086.25	1293137.5	1495732.625	706862.0625
2330671.5	1359085.25	1862496.5	691669.875	3129246.25	661904	2311394	846233.75	1117837.125	850464.5	1001609.188	1671039.75
1567124.5	1472230	1331614	1656417	1479644.625	2277440	1539291.125	1520964.625	1581693.75	1626949	1635890.875	1245484.625

841544.5	1625128.875	1754054.625	1230453.125	1775114.875	1026426	1206910.25	2928933	2065416.75	2473555	1958132.875	1998461.25
828430.5	1088245.75	7201553	850300.1875	782891.125	13744159	305396.0313	625593.75	297832.125	775411.875	1200344	1395555.625
1797627.25	1640066	1104869	1599461.25	1769972	1431064.5	1456609.375	1678282.5	1710727.75	1512654	1165569.625	1691066.25
1823562	978926.5625	1016658.063	1028684.625	2260759	923265.8125	918108.8125	2166270.5	2553050.5	1588291.875	1166901.625	1157554.25
1653209.25	1507475.25	1441419	1751528	1302402	1365493.25	1666211	1756584.25	1868236.75	1882610.625	1434480	1451653.75
Filtered	Filtered	3002622.75	Filtered	928247.5	Filtered	1302879.875	1194722.125	889397.0625	1821639.375	589337.875	Filtered
1520840.5	1380435.125	1213340.5	1284133.75	1481613.75	1340549.875	1549562.5	1578139.5	1681787.75	1792892.125	1423051	1403363.375
2142350.25	1206658.375	1156673.375	1021986.5	1285714.375	981319.875	1178869.625	1360123.25	1880681.5	1034799.063	1242095.875	1439335.875
1427052.875	1595648.125	1389664.5	1967041.25	2274135.5	1576677.5	1317873	1898157.75	1575042.5	1480602.875	2127693.25	2121858
1393508.25	1323743.5	1408227.875	1011937.063	1763870.5	1202676.875	1307476.875	1231769.625	1695445	1275012.625	1393325.625	1260410.375
1771119.5	1566526.875	1364851	1498504.375	954661.8125	1516926.375	772820.8125	1304973.875	1419504	1761307.25	1596296.125	1583657.5
428005.75	1314710.875	2898543.5	861431.75	540376.6875	2644660.25	487282.0313	509230	517541.5938	504701.625	298131.4688	423562.875
1418997.375	1052429.25	1134267.5	1113622.375	1512952.5	760322.125	1295275.25	1304542	1398451	1185159.375	1078666	1437834.375
1789699.25	1024145	1241068.625	1122661	1074993.75	659138.6875	1141392.75	656317.1875	1505518.375	1068960.875	1169197.5	962294.75
932882.5	1111266.5	1168427.25	1038648.75	1168597.25	962867.5	1221497.75	1382309.375	1835215.875	966780	1209312.625	1176309
975419.5625	1186016.25	844936.5625	1175092.875	1704687.75	1038846.5	1146105	1103384.625	1366848.75	1438622.875	1636883.625	1589584.25
850769.375	996818.0625	950895.25	975939.125	1135969	966385.25	1050092.625	1423008.875	1194529	1297654.25	1140823.125	1434342.5
1094087.5	1262079.625	1112882.125	1249505	1153836.375	1338266.25	1316477.75	941627.375	627197.9375	1204642.625	1435855.875	1298075.875
228057.5781	1065914.375	492909.375	690394	703757.8125	510112.3438	965608.9375	459558.5313	2126941.5	908922.4375	1231813	1707298
1191076.25	949249.125	960390.125	1071731.75	1290425.625	910855.8125	1247538.5	1027587.563	1179193.25	1203203	981400.6875	857970.625
7748.804688	1204161.5	209979.9844	1526792.625	1166378.625	76768.25	1968805.125	108319.9375	1316726.5	4000712.25	1085167	1332472.75
1006848.188	922897.5	949271.125	916602.75	1241222	826139.25	1024879.813	1356163.875	1488173.25	1029918	1205553.25	1069699.625
270082.125	432863.25	877811.0625	126613.8828	156510.4063	345749.625	107384.4766	340390.6563	104339.1484	178534.875	111539.0156	182901.6563
1230209.375	1172040.5	1185249	1126388.75	1187852	1327068.75	1135925.375	1097759.5	1032393.25	771543.375	1179468.5	539804.1875
944091.125	1057378.125	837964.25	938011.75	1054247.25	1198536.25	871180.8125	1096033.25	1075644.25	1173274.125	1033439.438	1052190.875
831085.125	980756.5	811788.5	1053670.375	986562	1310034.75	827520.75	848782.4375	1006240.375	1082237.25	890967.875	857504.5625
547001.6875	442920.125	1848753.25	573424.5	493194	1867176.75	467270.7188	660090.4375	455002.9375	1765620.25	419754.0938	397813.75
465423	960919.75	730648.4375	905564.4375	737005.9375	819388.8125	693542.875	550440.1875	630125.0625	1168702.75	655422.875	891707
935035	957264.75	754822.4375	964698.6875	1111293.875	792486.375	847270	975583	989484.875	988761.1875	642593.625	729466.5
317290.5625	854625.0625	2709816.25	417743.3438	377523.875	2183739.75	284339.8125	314391.3438	445946.125	407576.0938	203531.6406	410038.5
897988.5625	1143617.125	603793.3125	652865.5	692325.0625	558046.4375	799381.6875	798956.75	729793.5	727066.5625	662468.6875	893537.6875
972818.5625	731981.75	802367.1875	821416.5	663320.5	519972.75	645277	767243.625	827021	864247.3125	1015618.688	790708.1875
Filtered	916977.5	894806.1875	390379.0938	982579.125	1036352.813	878987.4375	902977.1875	Filtered	1208640.375	Filtered	Filtered
336702.875	324563.625	790037.3125	382543.8438	388465.0625	281452.9688	277506.375	249383.6563	264657.9688	215377.9375	290520.3125	612842.875
780259.1875	782180.25	618224.4375	573157.0625	1469728.875	548106.5625	685490.875	789995.9375	787121.3125	990349.75	617139.4375	663408.3125
751331.75	559213.125	542628.9375	634067.25	782895.0625	551078.0625	855778.9375	753100	824986.5	785599.875	723172.625	611294.375
707675.8125	602845.3125	510437.25	647798.75	813245.3125	517746.5313	528680.8125	762876.375	746113.8125	604641.375	627783.3125	592900.9375
147837.4063	233267.6563	480525.2813	79988.34375	83811.89063	186680.7188	57142.15625	220514.2031	67779.90625	106930.6328	77366.92188	139151.1094
571407	699804.625	518062.2188	623557.4375	673577.5	725875.0625	547486.75	593741.5	677614.4375	674328.125	605229.125	487634.0938
471550.5938	480656.9063	Filtered	Filtered	Filtered	Filtered	591153.5625	Filtered	621475.25	707444	Filtered	Filtered
456552.375	739159.375	499715.5313	533403.0625	399556.0625	507217.25	458155	618195.8125	625250.9375	495114.0313	525610.25	630249.875
620678.1875	565045.4375	477109.125	565175.875	860097.3125	458692.1563	589640.5	589052.75	557447.5	701729.875	694847.875	596863.8125
694704.5	472703.7813	518325.7813	505658.3438	612377.4375	423355.125	537194.1875	726596.4375	756299.3125	473929.5	667472.375	724311.4375
394215.0625	522672.6563	1809895.75	212445.6563	196184.5	2221142.75	153056.1094	138812.1875	105056.8125	260916.5313	218632.9688	779134.625
553869.1875	220170.2031	568614.0625	158915.6719	113484.0938	221425.4688	665326.75	86927.21875	52086.39844	720911.25	607519.5625	916903.8125
480959.1563	721664.0625	482293.4375	531749.75	472489.1875	423687.9375	611983.5	577102.875	665600.0625	631935.3125	651097	745646.9375
464803.8438	611152.5625	895897.5	266050.0938	279031.125	2117310.75	212403.9688	176046.0156	125597.4063	209481.5938	208070.6406	510622.75
211976.7813	678865.5625	1745279.5	276170.2813	238639.0313	2590503.75	160326.7188	145724.7344	207529.1563	232251.8906	103250.1953	476492.4063
388520.125	448776.6875	464066.6875	505063.7813	236331.6563	463793.6875	678615.5	439613.4688	729689.75	592725.875	647581.125	403419.0938
505453.0938	396427.8438	1392767	491304.6875	235500.1406	1426366.25	171392.625	123649.9922	250508.5781	499187.125	519749.4063	767929.125
474911.125	358160.7813	404240.9375	482730.25	562597.0625	427945.9688	558151.1875	547206.875	563340.375	540904	555422.75	507635.125
618940.25	423798.3125	345359.75	632546.1875	483162.5938	407738.3125	420110.1875	497793.125	742964.125	476696.5938	683445.0625	789744.0625
372377.4688	819982.1875	577596.3125	531252.75	402630.875	382910.5938	400100.0938	393159.5625	532407.4375	409726.2188	485059.25	391361.2188
418403.1875	391291.875	404214.875	397876.5625	685794.9375	344843.5938	582223.4375	632028.875	622853.75	469975.4375	334698.6563	508369.1563
501817.5625	627491.25	33606.64844	33030.65625	1003052.75	42667.61328	238728.0938	43765.01563	1103489.375	1083189.75	45048.65625	1726546.75
592768.75	378775.4688	293960.4063	444536.4375	572033	720385.0625	597589.9375	340487.0625	364976.6875	244308.4219	378791.9375	560155.875
380217.7813	325299.5	264359.5625	305722.4688	388140.3125	208378.125	290598.0625	229563.8906	291828	170023.7969	280440.25	1558402.375
405943.375	477190	396195.9375	405717.9688	557146.0625	455900.6875	475357.1875	420852.4063	323685.8125	235906.125	264913.6875	445512.9375
436819.4375	496124.0625	402436.0625	308852.5	465964.5625	358979.0313	457566.0938	479030.7188	450122.2188	502361.5313	477722.75	401889.1563
17546.71484	674889.3125	279105.5938	1402163.375	476821.9063	534105	376784.2813	14417.74219	313507.0313	9789.884766	704459.6875	11340.19336
702026.875	266807.9688	296143.7813	271894.9688	674994.4375	290308.9375	270774.125	375526.0313	411551.5625	299095	315775.4063	863196.125
359191.7813	370514.5	259149.0781	301965.7188	260595.3281	336304.9688	371476.625	254723.1875	459150.875	456628.3125	675349.125	510475.5625
291695.375	268287.6563	135764.0469	160821.3281	318241.875	124801.1406	160082.4375	170843.5	373770.4063	126944.8984	251612.1563	258206.6875
565486.9375	349620.875	351830.3438	406443.75	444158.8125	236598.0781	327833	376807.5	461336.5625	399223.2188	447182.375	449616.5
Filtered	Filtered	Filtered	Filtered	Filtered	Filtered	Filtered	Filtered	Filtered	Filtered	Filtered	Filtered
343918.9063	323316.9375	321837.375	403328.3438	811988.5	193074.4063	410231.7813	396646.4375	394465.5313	382649.5625	365210.6563	338107.2813
398214.5	411521.5	279120.7813	350670.6875	365087.3125	317158.0625	320156.9375	266388.0938	414522.5313	344574.0625	355090.6875	342693.4063
287906.9063	200767.7813	189793.2031	165113.9844	189707.7656	47102.35938	233818.1406	183282.6875	232251.1406	121612.1328	249823.4844	536137.4375
176373.7969	104733.9453	169295.0469	150548.3281	436109.25	298549.9375	294385.5938	224357.6563	301805.6875	148697.9219	149482.375	220701.3438
432638.5	314742.7813	286505.0313	285005.375	473568.8438	370584.8438	302715.375	419174.625	461120.75	432711.7188	293954.625	392008.2813
403547.4688	365077.625	183109.1719	448049.625	372717.0938	263856.9688	494553.5	274756.3125	383230.3438	405874.3438	391230.0938	582949.125
280143.4375	228562.2969	268713.9375	294884.25	197279.4063	352968.75	328861.0938	400976.9688	302091.25	306566.8125	276916.75	271054.4688
492967.4375	293169.7813	300712.4375	256122.0469	481460.25	236743.6094	337178.1563	387904.25	494307.5938	281775.875	391950.3125	321828.625
Filtered	Filtered	Filtered	3519.116455	Filtered	Filtered	Filtered	Filtered	400978.7813	Filtered	415890.3438	Filtered
317673.6875	305334.5625	316205.8125	319870.875	407840.9063	293878.8125	305985.6875	300598.5625	487085.2188	306788.8125	346543.4688	229393.6406

60048.33203	881010.6875	269203.5313	383925.5625	4235821.5	31118.49219	240268.5469	29118.18164	407668	467611.5	304852.25	387302.3125
353590.5	350381.0938	462889.75	Filtered	199589.7031	Filtered	259350.2969	Filtered	Filtered	Filtered	Filtered	716428.25
324761.625	234863.4844	313350.3125	277982.125	266047.3125	309406	249894.0625	336685.2813	308267.5938	308274.4063	323197.25	200103.1563
223888.75	297367.6875	1039874.875	188825.0313	170875.7813	1383054.375	171000.7656	106980.6719	138628.5781	189413.0625	244042.8594	167435.6719
164328.3594	71512.72656	154784.5	524171.25	824602.875	355015.9375	291705.6875	16714.79102	612844.4375	31575.94922	216302.4531	1213540.625
Filtered	Filtered	384988.9063	50827.46484	26592.75	412390.1563	Filtered	140102.3438	Filtered	178054.2656	4392.339844	Filtered
233499.6875	277117.0938	713636.875	178363.7344	162448.0313	588482.6875	83329.60938	90077.96875	105834.8203	144132.6875	145539.8906	310466.0625
43943.41797	Filtered	30610.08789	13248.76074	7625.628418	Filtered	Filtered	12186.05664	3248412.5	15671.72852	Filtered	Filtered
31059.44922	Filtered	Filtered	Filtered	Filtered	Filtered	Filtered	Filtered	Filtered	570475.1875	163316.7031	Filtered
344290.6875	256974.0469	230120.6563	277793.3125	305015.4375	202992.2813	267610.5	278315.6563	289997.5938	272178	269747.4375	312730.6563
270957.4375	260717.6875	252681.4688	373438.5938	299459.625	246095.3125	239416.2188	469069.7188	260620.2656	225197.7188	271347.6875	156498.9844
146498.7656	354260.4688	285089.6875	217140.9063	248035.2344	129727.3984	201808.7813	460578.125	288609.8438	278270.375	445923.7188	325958.4375
175857.5625	393200.9063	282515.9063	232067.3594	362852.7188	387136.6563	261946.7344	306312.3438	153484.5	338361.3438	212603.2969	343640.6563
249130.0938	Filtered	358790.4688	360162.9063	242251.4844	224026.7188	Filtered	195540.3594	124954.3672	45039.8125	315179.6875	Filtered
289774.5	251906.9219	202746.8281	207659	231893.3594	200488.5781	179041.6875	190443.6406	203178.5313	210302.1719	310599.4688	802588.5
233910.9531	275610.6563	363196.5313	177604.3281	162444.3125	606682.1875	83193.65625	89918.5625	103859.4766	143229.1563	145538.7969	305286.2188
308990.5313	281344.5	177972.9219	286039.0625	232654.4375	305437.875	268169.625	304342.375	225864.375	245967.0469	197255.8281	286254.75
125799.8828	66412.78125	233017.5156	653755.3125	542436.6875	229718.2813	144462.0469	9201.992188	332966.875	18851.26172	141699.4219	602623
162475.1563	102399.4375	134130.0781	259059	342434.6875	211894.9531	212999.9531	349449.6563	304679.0625	262878.6563	230564.9688	190896.5938
256368.3906	293314	234782.875	204202.9844	307403.9375	241841.6563	280081.2188	205395.4063	304304.7188	251755.5313	275535.8438	324169.9063
333250.375	332454.75	279494.625	152638.3594	239161.4531	173865.8125	272781.75	180189.4688	148591.6875	181178.2031	202723.5938	207336.8438
475249.8438	332416.625	516926.5625	70395.74219	76090.09375	676097.4375	49683.78516	101061.8281	51588.59375	184225.1406	163740.1094	389544.125
150696.6875	274394.9063	274126.75	194236.6094	173190.2656	170335.5469	207923.0313	218767.9688	431828.125	183293.4063	329872.5938	400596.8125
223737.6563	212743.9063	260153.25	276131.4375	259167.3906	206147.5	243980.3438	207680.2813	205353.4688	235725.6719	167798.1719	174677.0781
248790.2344	213729.5469	560126.9375	226083.0469	194663.5313	908814.1875	106168.5938	157918	135379.875	178212.4688	237699.7813	224665.1406
219968.625	138742.4375	368085.8438	164540.9531	103836.2734	Filtered	214078.4219	295906.75	603481.75	337971.5625	212502.5625	Filtered
366800.7188	314890.7188	152680.9063	179572.4844	231995.2813	88247.60156	139190.8594	155079.25	384570.9063	154948.9375	386008.5625	464112.625
144977.3594	119608.3672	189797.5938	56538.31641	510380.5938	404099.4063	349237.4375	304667.3125	292156.25	358469.5938	309515.6563	168837.7031
229626.5625	285479.375	176746.3594	238768.875	244984.2969	227423.1563	248457.7969	229695.0781	269622.4063	242091.3594	272156.9375	229591.375
246802.625	195576.3906	250114.7188	299761.5625	261654.7031	233344.3906	315977.0625	389643.0313	289135.5313	274488.4375	349394.25	324435.3125
172138.6406	110680.2031	113804.1484	157077.5469	262016.5625	181655.5938	141081.8594	189539.1563	187876.4844	118032.8281	167576.75	85467.72656
146507.4219	183968.6094	685205.75	143844.0313	113373.8438	493368.8438	74886.28906	48845.35938	46620.42969	103905.2188	99836.70313	180153.7031
169809.2813	232565.8281	224017.0313	203024.8594	160114.0781	281790.625	249232.4844	265989.3125	253870.8281	258598.2813	236549.8125	228270.6875
226601.5	191476.0156	163405.6563	157644.25	225063.2656	184069.0938	191868.0469	158097.25	185724.8906	111888.2109	150389.6875	189758.6094
156763.5313	172620.3125	835293.5625	98436.76563	56209.14063	498913.625	144111.5	59189.81641	73056.03906	194318.3906	91409.71094	131163.9531
261103.7656	202427.7188	188397.1875	159723.1406	177789.7813	169766.1875	159527.25	212598.3438	237145.4844	217750.9688	686688.375	176546.0781
304149	208214.2344	248750.0938	215710.5625	169597.0156	121220.2031	222576.75	208023.0156	190335.6719	141843.7031	189393.6563	228095.3125
Filtered	Filtered	Filtered	Filtered	Filtered	Filtered	Filtered	Filtered	Filtered	Filtered	Filtered	Filtered
124068.8984	187894.8281	504198.125	99778.17969	83776.28125	829410.75	72056.76563	74082.125	44352.68359	103612.4375	72907.84375	144724.0938
215431.5625	182472.0938	178725.2969	185772.9688	255674.5	161483.9531	222615.2344	227515.4219	244063.5781	186714.625	222097.3594	197021.5
262090.5313	180631.3281	210769.5781	91517.6875	281170.0625	130713.9609	260377.2188	109905.0078	383154.4688	147918.2344	269210.6875	223636.3281
Filtered	Filtered	Filtered	Filtered	Filtered	Filtered	Filtered	Filtered	Filtered	Filtered	Filtered	7842.022949
230414.7031	200475.5781	158518.5938	174135.4531	197944.2813	144036.6875	223167.1406	189346.3125	233256.7813	184554.9844	169101.2031	194842.4375
Filtered	Filtered	Filtered	Filtered	Filtered	Filtered	Filtered	Filtered	Filtered	Filtered	Filtered	Filtered
250979.7344	220979.1875	65441.80859	90544.23438	155084.25	165460.1563	180839.5469	209621.2188	285960.4375	77534.17188	260399.7188	138377
177304.7188	85059.73438	79437.16406	96635.35156	179473.1875	87293.27344	86843.96094	222984.6094	219602.0156	146174.9375	Filtered	Filtered
141691.5156	360754.2813	133361.2969	145056.75	199231.5313	263169.6563	152239.5781	89584.10156	154944.8906	268061.8438	220233.9688	209076.8125
185346.5469	168907.5	150638.9375	154472.9531	211469.5	127549.7031	158290.7188	207285.25	217383	210605.4844	172254.7188	137047.1875
213962.6563	265270.5	146796.7031	220273.6563	173722.1094	134793.0625	215213.0625	208086.875	276425.0625	301442.7813	146060.2188	114575.5625
82435.22656	Filtered	78024.53125	525810.0625	211054.1094	178904.0156	74454.125	Filtered	306983.5938	15478.94629	105511.1406	606736.125
144272.3281	153753.2031	109340.7813	166247.4531	135503.2344	118422.3594	139728.6875	201904.3125	198300.2969	150352.1094	212172.0469	152433.1406
105293.9375	99463.51563	417707.3438	93843.6875	79305.8125	749994.3125	97097.24219	52590.42188	48024.72266	69680.85938	47674.75	146453.6563
60889.16016	43674.98438	46933.15234	498056.5313	330361.4688	138769.375	255362.0469	13942.92773	534319.9375	27485.56445	203540.8438	452944.4688
629240.8125	Filtered	637.2938232	Filtered	Filtered	Filtered	Filtered	Filtered	Filtered	Filtered	Filtered	Filtered
127921.0078	145958.0625	63955.28516	219805.5781	138564.9219	61983.93359	199265.5781	252852.875	244202.7969	109531.7031	111422.6094	251555.25
222402.9531	167241.7031	127150.2656	171758.1875	156984	134954.3125	127694.7266	138112.3125	163949.8438	148525.25	203457.5313	152745.5938
246012.0625	Filtered	Filtered	Filtered	Filtered	Filtered	Filtered	Filtered	173912.1563	Filtered	Filtered	Filtered
31210.19141	100909.0703	619871.625	73000.80469	Filtered	394737.875	44552.51563	Filtered	Filtered	Filtered	66641.46875	88427.75781
1145111.125	53988.98828	37529.51172	21344.86914	17986.16992	16943.87891	39854.04297	43858.91016	34506.10156	50218.43359	52353.19922	63452.25781
95320.125	121172.0469	548706.4375	88436.23438	71497.32813	495707.4375	52313.30859	44471.27734	42933.71094	45397.375	69280.78906	79834.3125
6.24E+04	1.55E+05	1.18E+05	8.64E+04	1.61E+05	2.70E+05	8.03E+04	5.85E+04	9.85E+04	2.18E+05	1.87E+05	1.17E+05
142129.75	110062.2813	106230.3828	135133.4063	183580.25	161092.625	126476.0625	158945.1875	157757.0313	151742.0469	143324.3438	156678.9219
156414.2188	252848.6719	338263.25	85400.4375	141815.1406	181888.4063	98207.89844	91726.63281	79559.57813	87153.67188	148997.6875	208163.1094
113895.9219	67864.38281	247321.4844	115018.25	93087.53125	79908.25781	153063.5	131005.8828	95079.8125	336796.2188	117534.2891	11507.89844
70551.07813	125860.8906	53009.1875	193007.4688	129326.1797	87616.23438	92952.80469	218576.75	237065.0781	62844.39063	172315.8125	223998.375
141389.0781	88030.92969	165687.4531	93554.78906	95175.21094	158931.7344	184003.3125	128193.6328	33481.52344	80739.46094	69278.4375	80389.27344
62388.11719	102251.0078	128416.9219	82080.05469	184711.8906	212041.6563	100135.0625	106091.1641	96067.73438	141324.9063	102949.0938	73926.03125
40677.19531	35128.49609	119182.5625	71305.6875	87105.97656	442511.7813	48763.8125	50264.48047	188731.5938	73628.39063	39766.125	39320.64063
398717.625	51275.13672	19241.61719	14071.27344	195289.7344	13557.62988	23077.52734	35078.72656	35794.875	18218.28906	26686.41406	139244.3281
Filtered	Filtered	24968.42969	Filtered	Filtered	Filtered	Filtered	114169.6094	62633.0625	44950.69922	Filtered	Filtered
106735.25	122639.3359	94115.35156	101543.8906	78095.76563	70635.90625	175141.3594	162952.8906	174518.125	176484.5938	154317.6563	172946.9375
69268.41406	114281.1875	658042.8125	62131.24219	50453.68359	573990.1875	37809.375	31354.36523	16252.52344	43543.42969	82117.57813	60290.58203
103981.9141	103444.3906	182888.7656	57088.375	119555.1328	75591.30469	118246.6172	121451.5234	211180.6563	197816.2344	202424.5469	145390.4688
126234.9063	208560.3438	76625.02344	148940.3438	192003.6563	93324.97656	97424.875	89743.30469	149465.4531	114174.5391	195903.0469	150904.5469
12159.27832	17488.50586	Filtered	Filtered	6975.040039	4082.653809	10741.16406	Filtered	Filtered	Filtered	Filtered	1346426.375

86670.76563	119600.6563	91563.75781	111462.2578	78215.78906	123437.2344	110618.6016	127515.3906	119693.1406	134809.8281	126755.6484	182407.4375
121979.6719	103783.6094	143425.1719	128575.6328	97842.66406	111522.1094	139216.2344	78050.07031	133297.8594	112125.7813	168056.4063	243573.4531
99935.125	101533.3438	181281.0938	73086.53125	73469.82813	406926.0313	44199.83203	45000.27734	42627.51953	72668.77344	79427.75781	95959.96094
155071.8281	103557.8594	99843.55469	98187.72656	90224.125	119699.4688	141769.2031	91895.02344	172153.7813	152635.5625	103058.8047	92334.42188
Filtered	139903.1719	93534.58594	169924.1875	125950.0859	90555.35938	Filtered	121425.2891	123853.75	171081.6719	135562.6406	Filtered
Filtered	4152.432617	Filtered	Filtered	Filtered	Filtered	Filtered	5738.592773	Filtered	1350.304443	Filtered	Filtered
127733.6875	124384.3984	157670.5938	72156.48438	78310.14063	93201.10938	99054.14844	58069.92188	98536.59375	145003.9844	168309.1875	130408.4688
248537.2188	31967.80664	82825.60156	57107.0625	500404.5313	18117.3125	163713.9688	32844.78125	356694.25	63548.69531	41973.45313	48960.54297
111773.2188	138952.3906	101182.3828	89396.09375	89295.85938	76430.78125	127684.5547	127835.9219	123018.625	111272.8516	104102.5703	198029.9219
87426.09375	Filtered	Filtered	30253.42578	Filtered	Filtered	Filtered	Filtered	Filtered	Filtered	Filtered	Filtered
73822.375	52563.35156	208356.4063	67743.3125	50778.55078	268814.3438	80881.74219	96467.57813	63841.15234	184429.0781	45120.69531	53004.30859
Filtered	Filtered	Filtered	Filtered	Filtered	Filtered	Filtered	Filtered	Filtered	Filtered	Filtered	Filtered
Filtered	Filtered	Filtered	Filtered	8675.188477	Filtered	Filtered	Filtered	Filtered	Filtered	Filtered	Filtered
83375.64063	271367.4688	100119.9531	92497	105883.3594	39307.72656	111351.1016	295274	116056.2578	112056.6484	96986.03125	98262.85156
180755	Filtered	Filtered	Filtered	101357.3906	Filtered	Filtered	Filtered	Filtered	Filtered	Filtered	Filtered
Filtered	Filtered	Filtered	Filtered	Filtered	Filtered	Filtered	Filtered	Filtered	Filtered	Filtered	Filtered
79475.94531	81882.16406	78785.03906	65029.23828	79993.05469	68375.01563	84386.52344	42449.25781	106615.9531	78208.53125	106095.4141	92210.21875
55964.72656	88766.10156	492689.9375	59453.28906	49908.25781	278777.9688	13190.38281	30797.26563	41941.51563	15405.22266	Filtered	323262.9688
Filtered	93161.21875	517508.0938	Filtered	43439.32031	583651	13138.11719	Filtered	7505.959961	36362.17578	20115.42188	89245.71875
101985.7578	90846.29688	83678.51563	93984.54688	78889.44531	70598.25	89762.39063	113220.8672	109110.6172	106468.8594	111383.2969	90356.64063
64787.51563	73574.01563	96797.35938	82726.375	80129.39063	77321.96094	94116.70313	90177.4375	78881.48438	131302.5625	111238.4063	67275.99219
117854.4453	77649.13281	497684.6875	47644.70703	40450.67578	328142.4688	33371.60547	25166.29102	29371.53125	57593.4375	71428.39063	61561.87109
25132.9375	45725.87109	28833.53906	51470.97656	39259.87109	24092.31445	32613.95117	46249.15234	43259.55078	25446.11328	32589.58398	80830.98438
59221.78516	127109.8672	64153.96484	87733.00781	116092.9297	112690	100534.2266	129498.2891	83089.25781	257037.7656	131147.7188	126450.5156
83643.63281	95132.33594	100342.0859	111969.8438	73971.01563	128397.1797	60916.25391	113833.9375	57132.58984	107473.7188	114376.0156	90418.49219
141817.0938	94392.75781	91109	98779.24219	62130.05078	9699.880859	102473.9219	97460.03906	201815.6406	163043.2813	130747.6016	Filtered
57736.31641	56992.67188	89694.89844	63030.54688	47365.39453	56158.125	89892.0625	62035.87891	116324.3438	87595.96875	106618.0156	94670.32813
161132.8281	138505.375	81000.48438	75145.96875	95107.61719	105857.1797	106916.9297	89641.52344	181406.6719	97693.92969	45906.88281	85571.78906
48410.64453	19649.25977	95845.78125	310556.4375	372830.5625	76066.67969	89098.875	10063.79395	223263.8906	13901.12305	30566.31055	271373.25
68271.78906	69367.57031	60475.54297	52968.125	49981.57031	41719.22656	92043.70313	80314.61719	92024.19531	81404.55469	88527.39063	96467.65625
Filtered	Filtered	Filtered	Filtered	Filtered	Filtered	Filtered	10633.06445	Filtered	Filtered	Filtered	Filtered
85782.83594	89139.5625	74062.89844	71416.99219	80401.57813	39727.21875	76624.14844	53672.80859	92560.26563	44878.30469	70710.9375	187367.4844
Filtered	Filtered	77886.75	Filtered	Filtered	Filtered	Filtered	Filtered	Filtered	Filtered	Filtered	Filtered
32194.3457	15887.04883	18507.87305	329785.3438	170731.5781	44772.12891	113946.5	9312.879883	171955.8281	11751.39063	47109.11719	243262.4063
31793.92969	100482.8516	28291.52539	99834.94531	50891.85547	79133.04688	50176.02734	64382.50391	63129.41797	24235.36328	126168.3047	305503.875
44855.33594	52857.27734	57900.17188	83437.11719	44372.95703	80729.83594	59908.64453	69735.60156	102933.4219	99107.60938	36057.62109	74845.57031
82534.66406	66487.89063	80228.125	43369.19922	79798.79688	45540.30469	87101.9375	60960.89063	81429.79688	73816.5	84928.78125	66975.82031
Filtered	Filtered	Filtered	53820.91016	Filtered	Filtered	Filtered	Filtered	Filtered	Filtered	Filtered	Filtered
32710.96289	35517.71484	83899.52344	27197.96094	32412.34375	397608.1563	23761.77148	41579.23438	24105.02148	23450.53125	36830.71875	33711.17969
Filtered	Filtered	Filtered	Filtered	Filtered	Filtered	Filtered	Filtered	Filtered	Filtered	Filtered	Filtered
63642.39844	58593.10547	60121.8125	79160.89063	61208.25	41655.87891	76121.71875	73171.0625	71184.82813	115716.9141	63092.10938	72589.85938
60520.25391	47116.66016	39032.60547	50752.97656	93733.05469	54589.26172	61639.89063	36161.36328	80778.05469	70712.96094	47648.98438	38263.31641
35311.62109	59777.80469	167815.6563	31209.63281	27808.32227	246348.2188	24898.08984	9513.125977	12524.8125	30372.61523	20286.60156	63812.77344
Filtered	64508.87891	30347.74219	Filtered	43849.60547	Filtered	Filtered	89384.67969	Filtered	59090.15234	Filtered	Filtered
111182.6719	110714.0469	50885.57813	26134.03906	118379.5547	23500.20313	88324.76563	28239.63086	57563.85938	35592.81641	41825.15625	119265.4766
Filtered	Filtered	Filtered	Filtered	Filtered	Filtered	Filtered	Filtered	Filtered	Filtered	Filtered	Filtered
104016.25	109344.3984	42987.37109	56326.60938	69281.78125	23499.78516	112217.3984	106413.1719	141763.0469	60461.6875	106688.8672	142173.8906
80298.96875	77581.76563	60231.55859	60520.13672	69671.71094	59054.63281	103597.0234	72069.9375	75168.22656	73184.88281	71201.59375	83695.07813
3661.185547	3715.133301	1159.97229	4318.560547	4149.479492	5405.023438	79997.9375	105167.4141	4558.202637	69928.85938	160278.625	Filtered
52559.75	67413.28125	162693.75	34314.65234	30070.88672	157080.875	30405.33398	28729.88477	15533.57617	32974.20313	22288.74219	51038.03516
27772.07422	17620.20117	21059.0293	131764.6719	123120.1172	117153.8438	42071.03906	Filtered	114024.8203	15120.88086	33953.08594	96859.49219
84757.82813	61737.59375	35911.66797	74042.63281	67169.78906	59479.41016	96721.5625	104412.1953	110422.9766	93009.92188	132671.3594	185611.9375
60521.39063	57082.41406	63070.50781	54067.39063	53363.90625	48861.05859	74287.28906	75501.75	67964.44531	55153.4375	53573.32031	96416.64063
Filtered	42946.17969	Filtered	65448.89063	72183.89063	29885.00391	Filtered	65031	Filtered	Filtered	Filtered	Filtered
39902.33203	42880.30078	67380.39063	29873.83398	32948.08594	111198.6797	25698.61133	23606.78516	37446.34766	49346.22656	34482.76172	33772.93359
45272.05859	43186.89453	53802.09375	44858.70703	22638.89648	75682.15625	34337.79688	65496.83984	51972.41406	72167	68821.29688	59966.16016
26443.38477	Filtered	Filtered	Filtered	Filtered	Filtered	Filtered	164750.8281	Filtered	Filtered	Filtered	Filtered
87695.03125	84185	62737.17578	63846.37891	55413.76172	21374.58203	69258.23438	34916.71875	73379.8125	10873.54199	117511.5625	169863.2813
Filtered	Filtered	Filtered	Filtered	Filtered	Filtered	Filtered	Filtered	Filtered	Filtered	Filtered	Filtered
40991.72266	59703.37891	7230.083496	40364.16016	32190.13672	168895.875	25083.70508	20687.17773	17863.82227	25075.56836	24932.74805	23627.20508
69024.71875	65520.44141	48473.57422	36619.48047	82055.46875	50083.48047	69905.48438	41525.15234	48904.74219	59479.57813	41113.33984	93387.95313
32589.85547	77248.99219	12867.66699	98148.29688	31294.45508	123384.9375	69316.375	126989.625	46159.33594	35968.1875	63010.19922	53769.21484
38774.46094	50397.92969	161324.2031	24066.42578	21409.0918	173238.2813	21890.2793	12926.02246	Filtered	24443.85938	Filtered	Filtered
Filtered	Filtered	Filtered	Filtered	Filtered	Filtered	Filtered	Filtered	Filtered	Filtered	Filtered	Filtered
51785.25781	55393.05469	40169.5	60479.0625	68436.77344	17740.71875	33897.1875	Filtered	Filtered	Filtered	Filtered	Filtered
95413.34375	49756.85156	49870.58984	42680.92188	39885.61328	38575.25391	51911.37109	47939.94141	79567.71875	66068.54688	29653.4043	87537.47656
40921.89844	54938.34375	54301.21484	54704.66406	46273.44922	71851.74219	62739.02734	48269.73047	69842.99219	96509.61719	50070.82422	23129.96484
47256.23438	65578.98438	48956.02734	120076.875	55721.86719	35993.70313	48672.03125	58254.40625	72623.44531	83252.96094	82331.38281	58797.6875
52178.38672	76292.5625	43929.95703	39074.95703	67793.10938	46222.21875	80848.57031	72546.94531	49805.48047	40855.72266	61433.03516	85742.21094
33471.61719	19745.42188	91987.625	Filtered	Filtered	133883.6719	Filtered	Filtered	Filtered	10006.55273	Filtered	Filtered
43344.38281	46178.51563	55840.14844	47842.84766	93798.47656	22782.66797	45295.33594	68109.67188	68206.89844	44851.58594	55226.59375	63387.76172
49469.15234	36113.67188	41455.77734	41593.39453	41934.53125	29544.48242	61532.37891	45570.9375	63741.41797	51816.26563	42820.76953	57359.70703
45010.71094	46114.20313	47832.48438	47594.83594	37103.23047	49267.66016	60214.03516	62658.19922	56291.13672	51300.35156	45248.17188	49741.875
57821.83594	51496.55078	62131.42578	51164.40234	72092.3125	25774.57031	57970.89063	37530.60156	108090.3281	54527.99219	47559.32813	49786.08984
37974.81641	27988.98633	89170.99219	81515.6875	49612.75	114351.1875	28896.99414	24296.17773	15554.89063	15169.59766	16875.62891	Filtered

59810.03516	36998.92188	25386.28906	54124.32031	54689.90625	50627.61328	53776.86328	Filtered	Filtered	54891.04688	66960.78125	75609.23438
37880.99219	43540.42969	190428.6563	22749.53516	16053.82813	130644.3281	9532.762695	13208.57617	10330.71191	24457.06641	26635.63086	57250.09375
14651.89648	11879.2793	40437.60156	179701.5469	108060.3125	38164.79688	29779.90625	5447.706543	142688.5	7615.641602	18138.6875	173033.2031
Filtered	Filtered	59639.60547	26764.07422	Filtered	32558.37109	48858.19922	27181.41211	29963.80078	Filtered	31382.34375	86275.14844
35409.60156	37135.17969	43320.04297	46324.50781	86611.15625	38947.42578	48876.35938	37256.28125	58372.62891	42785.75391	36172.14844	43366.92969
71474.34375	47914.80469	31195.22656	63908.42188	45624.75781	43002.21094	36237.68359	36467.04688	54722.60547	39791.68359	53270.14063	37134.48828
28946.75781	48846.93359	39040.56641	56721.76563	64479.95703	39885.98438	43068.84766	58706.20703	40943.69531	47452.26172	42170.94922	45227.52734
100001.1484	43042.42188	39618.74609	Filtered	25812.31836	17168.88086	5603.45752	25152.29102	18790.00391	46491.32422	69336.42188	169850.3906
Filtered	Filtered	Filtered	Filtered	Filtered	Filtered	Filtered	Filtered	Filtered	Filtered	Filtered	Filtered
37586.49219	47655.59766	140607.5	68355.75781	28307.55273	97734.57031	11206.86719	25697.38672	Filtered	30828.83789	Filtered	Filtered
24222.04297	47746.49609	199477.5781	21028.0625	20579.87891	118965.7109	10193.88867	14522.98047	10835.3125	20427.18945	15222.70117	29801.48047
50577.47656	43050.27734	41244.24609	45492.00781	69293.30469	42021.74609	38668.80469	48598.70313	48496.67578	52627.90234	47197.46484	56074.20313
32230.71875	20046.52734	46153.56641	53022.90625	31020.80859	22592.20313	71986.28906	41984.86328	72742.39844	53425.42578	36189.51563	24305.8125
Filtered	Filtered	Filtered	Filtered	Filtered	4682.586914	11728.66895	Filtered	15974.22266	127766.7578	Filtered	Filtered
64850.47266	50692.08594	43064.75781	34709.63281	88267.48438	42711.50781	57383.15625	65075.40625	71284.23438	54646.19922	45308.41406	37826.48047
25139.11914	22880.23633	30986.6582	23447.81641	17561.33594	20470.4375	42316.60938	17373.62109	38801.57813	35065.59375	77450.94531	34155.38281
40382.09375	43488.42188	67493.82813	33481.39453	40648.22266	36528.54688	55858.76172	47905.05078	44783.98047	50143.67188	54295.28125	49391.94922
24747.10742	15221.02344	21761.4043	15267.28516	36733.51953	16231.26465	20065.34961	17604.42969	17210.43164	17060.27148	14989.84277	19648.57227
33899.00781	50270.93359	42109.51563	42271.66406	32708.16992	27018.45117	51651.9375	58992.125	45945.41797	44838.66797	55691.58984	53072.46094
81113.51563	42010.92188	40823.6875	45555.54688	29094.60742	44661.67969	63522.9375	31397.07422	42246.76563	57268.00391	39742.48828	45366.08203
11611.05176	17265.43555	84881.33594	11872.9873	Filtered	25955.05078	5345.537109	32869.78125	19515.62891	33567.41406	28670.53906	70624.55469
Filtered	5933.336426	Filtered	Filtered	31651.45117	119119.3906	97083.24219	19622.95703	77110.23438	64406.17969	Filtered	Filtered
49954.18359	48610.72656	152417.375	28008.50781	21619.0293	187508.5	23187.46094	12760.79785	10454.99023	30543.17969	27826.61914	85479.26563
45892.98438	51026.87891	35830.16406	34089.32422	56226.77734	28620.00781	43601.11719	33952.63672	51604.97656	47964.46094	40914.52344	54261.82813
2355.340576	Filtered	Filtered	959964.6875	Filtered	5677.868652	9207.571289	6471.566895	7516.563965	6336.79248	9239.095703	Filtered
Filtered	Filtered	Filtered	Filtered	Filtered	Filtered	Filtered	Filtered	Filtered	Filtered	Filtered	Filtered
2596.949219	Filtered	Filtered	Filtered	Filtered	Filtered	Filtered	3922.008301	Filtered	3354.71582	Filtered	Filtered
40489.78516	58710.95313	63519.82031	40082.22266	31746.37695	63466.69531	25026.73438	20571.01758	18045.59766	25046.55273	25404.70703	23302.70313
Filtered	Filtered	Filtered	Filtered	Filtered	Filtered	Filtered	Filtered	Filtered	137777.875	Filtered	Filtered
Filtered	Filtered	Filtered	Filtered	Filtered	Filtered	24912.25586	26853.08203	Filtered	Filtered	Filtered	Filtered
Filtered	Filtered	Filtered	Filtered	Filtered	Filtered	Filtered	Filtered	Filtered	Filtered	Filtered	Filtered
38528.79297	29763.88867	30285.33594	38824.30078	41472.01953	25663.1582	57715.17578	325943.5938	40245.23438	61412.9375	23975.61133	79436.89063
26172.82031	22750.40625	144223.3438	28527.32227	28668.41406	144640.1563	15502.91406	20757.17188	19015.56641	28807.57617	21457.12695	18661.89844
69431.26563	35310.45313	35375.57031	34383.49219	28872.77539	23618.95703	39147.55469	36321.53516	37797.67188	33341.58984	33000.69531	24837.22266
33636.69922	43725.85938	44133.32813	35472.69922	32016.17383	31437.41016	26024.79297	33467.33203	34762.60156	46509.16797	36538.95313	Filtered
72830.25	Filtered	Filtered	1394.521606	643.5240479	81433.32813	62879.70703	75594.76563	38524.62109	86508.03125	Filtered	Filtered
33735.96875	41008.06641	37165.125	32219.19336	26288.86523	34739.59375	39577.73047	41168.97266	45424.64453	47180.98828	40130.96484	26772.625
59507.58203	40982.27734	41255.30469	21226.16797	27011.91406	25898.75781	45562.69531	43318.12891	50104.13281	24124.29492	37075.78906	34917.13281
56033.33594	24539.00195	22019.08398	34552.53516	37175.34375	19367.85352	45334.22656	319587.25	39187.04297	45720.01563	28902.57422	56838.74219
46620.55859	27039.14844	43520.77344	30285.99805	47827.08594	21160.18164	44497.09766	26401.27148	34958.15234	31649.81445	25233.83984	48173.84766
26372.25781	30586.20313	28642.1582	22262.26953	18119.87109	32214.16406	38377.42188	32430.10938	28941.9375	33101.55859	72910.39844	37544.71094
50158.14844	35670.57422	30002.17578	34051.84375	32300.1875	26574.48242	31966.83594	37931.4375	39778.50391	44437.54297	37925.40625	40107.29688
33514.5625	43432.375	30247.16211	44015.05469	31049.95508	33826.78516	45606.72656	45564.73828	47623.71875	48794.54688	46208.72656	45083.91406
28968.625	25465.12891	25135.12305	23407.68555	36496.76172	29303.24219	50444.82031	21258.81055	73425.74219	81959.59375	29807.06445	36096.16016
Filtered	Filtered	Filtered	Filtered	Filtered	Filtered	Filtered	Filtered	Filtered	Filtered	Filtered	Filtered
42864.82422	Filtered	31326.84766	Filtered	64476.95313	46203.4375	Filtered	10519.51367	Filtered	Filtered	Filtered	Filtered
13643.89063	11570.01758	28810.1543	15691.23438	44349.85938	25825.01953	15019.35938	57853.54688	69872.54688	12826.14355	85201.30469	50010.5625
9179.492188	Filtered	Filtered	7287.299316	Filtered	2144.309082	Filtered	Filtered	9610.936523	Filtered	Filtered	10504.97949
Filtered	Filtered	Filtered	Filtered	Filtered	Filtered	Filtered	Filtered	Filtered	Filtered	Filtered	Filtered
38860.98047	31895.52344	87075.46094	37791.30078	17402.71094	88562	29650.12109	15448.70605	11704.97559	29184.73242	15277.6377	28816.02148
30687.55469	37761.57031	31120.21289	27391.6543	25531.07422	29974.63281	45330.51953	35173.59375	34037.59766	34297.47266	43994.60156	50327.47266
24020.14258	23041.75	21887.34961	28463.94531	56486.07813	37588.35156	48595.69922	40378.31641	29580.07813	25473.57813	31773.47852	48018.67969
50254.70313	55537.48438	19725.02344	53047.70313	49704.51172	19313.79492	29487.6875	43018.07422	37685.27344	26018.80078	41556.71094	36820.70703
33362.42969	17069.83398	28107.63867	32343.51758	13523.19531	18658.99219	15478.50586	22785.56445	31067.98047	19017.00977	16871.15234	18021.22656
19183.72266	Filtered	16843.52734	102827.8203	45247.91797	25818.85156	12470.11328	34505.89063	54717.75781	37997.75391	49791.94531	Filtered
Filtered	Filtered	Filtered	Filtered	Filtered	Filtered	Filtered	Filtered	Filtered	Filtered	Filtered	Filtered
Filtered	Filtered	Filtered	Filtered	Filtered	Filtered	Filtered	Filtered	Filtered	Filtered	Filtered	Filtered
47176.67188	20641.48438	36568.91797	57503.35938	55745.30859	15217.49316	8631.952148	Filtered	21944.6543	Filtered	19387.31445	55097.89453
Filtered	Filtered	34267.55078	Filtered	Filtered	54746.65625	Filtered	Filtered	Filtered	Filtered	Filtered	Filtered
42392.71094	23586.78906	20968.35156	25601.70313	39253.97656	28566.5293	23495.67578	28011.28125	41110.75391	32278.9375	42587.19141	17083.2793
14976.24707	6255.871582	7839.811035	117564.2266	70140.8125	22022.05859	47661.05859	6546.054688	62083.37109	10543.28516	8348.296875	115256.3203
53298.35156	40594.45703	54869.13281	26034.23242	46269.32422	5088.80957	30020.87109	47970.66406	33661.84766	25366.95313	29418.05469	Filtered
43538.75	72596.36719	24663.93555	35876.51563	33064.80469	9553.587891	42517.5	129119.3438	31287.46289	47223.37109	30903.95898	40447.39063
Filtered	Filtered	Filtered	Filtered	Filtered	23030.14258	Filtered	Filtered	Filtered	Filtered	Filtered	Filtered
7313.959473	13332.23047	20018.24414	Filtered	4327.436035	9854.482422	Filtered	10524.30078	Filtered	4070.301514	Filtered	Filtered
Filtered	31465.2168	1519.703735	Filtered	914.3677368	Filtered	Filtered	Filtered	2892.670898	2684.392822	2608.36499	891783.875
26962.88672	19758.73438	19520.61719	8538.155273	38346.03906	13959.02344	26122.4043	27577.54883	23153.67969	29065.65625	34288.68359	31809.62109
20755.76953	31136.51367	21056.00195	27622.71289	38635.46484	40684.51563	34643.04297	24781.10938	26598.99805	21694.6875	29896.26563	39818.10156
Filtered	Filtered	Filtered	Filtered	Filtered	Filtered	Filtered	Filtered	Filtered	Filtered	Filtered	Filtered
40544.75391	7439.518066	5936.774414	5101.471191	6107.156738	55272.66797	7285.419434	45382.01172	52804.39063	7990.53418	6985.418945	Filtered
10322.80859	9731.556641	48518.48047	Filtered	Filtered	37160.55859	Filtered	Filtered	Filtered	Filtered	Filtered	Filtered
Filtered	Filtered	Filtered	Filtered	Filtered	57359.28906	Filtered	Filtered	Filtered	Filtered	Filtered	Filtered
Filtered	Filtered	Filtered	16221.20117	Filtered	34018.33594	Filtered	Filtered	Filtered	Filtered	Filtered	Filtered
28064.73047	29139.22266	27249.45508	26947.18164	29047.79688	28525.82813	29379.17969	35352.03516	35144.41406	36917.5	37920.14844	34507.20313
Filtered	Filtered	Filtered	Filtered	Filtered	Filtered	Filtered	Filtered	Filtered	Filtered	2676.194336	Filtered

5429.615234	14878.30371	7484.155762	8013.302734	44660.34375	14101.81641	13760.49023	22206.90039	8480.728516	7599.083008	3209.018799	12161.06152
38708.83594	20720.58008	39483.88281	36156.14453	35927.24219	18441.49805	37675.3125	43019.41406	38829.05078	29895.83984	31923.74219	35410.67188
25094.94922	20983.90234	28064.85547	24992.4668	29040.4043	15676.21094	26372.66211	Filtered	47013.01172	29814.46289	12500.21973	Filtered
30395.52539	59029.03906	29802.51367	23651.08984	31474.69922	10846.84473	31317.05273	73317.125	37711.04297	31110.14258	28876.04297	35861.89844
Filtered	6984.144043	Filtered	Filtered	Filtered	53967.27344	Filtered	Filtered	Filtered	Filtered	Filtered	Filtered
39150.85938	18632.23242	24005.88086	30708.00195	26359.57813	21409.06836	27414.91992	28146.52539	35522.78125	32114.41211	23592.55664	28405.125
Filtered	18502.73633	Filtered	Filtered	117194.375	3578.689697	Filtered	Filtered	Filtered	2182.672363	Filtered	Filtered
20346.75	14087.53027	13890.68945	16785.36328	39635.44922	32576.4082	16933.29102	18743.54883	24327.97656	11246.10547	18850.60547	Filtered
57936.8125	12174.27441	47524.44141	22876.26953	22892.00781	79303.63281	2977.306885	17894.11328	6197.268066	24881.14453	5776.222168	473161
Filtered	6118.830078	45796.46875	Filtered	20571.83008	2712.192139	4249.544922	5202.691895	Filtered	Filtered	19565.17578	Filtered
30497.31445	36480.29688	30665.16016	29974.40039	24074.20508	19976.62891	24286.79883	25397.87695	30764.52734	30976.10742	30644.7207	35035.3125
28231.14844	19419.73438	18171.65625	19761.44531	24530.71094	20160.94727	26156.24414	19340.16992	30930.30664	22565.23438	22365.22852	70489.92969
30248.41406	23687.35742	23412.01367	14413.29102	22626.30273	32901.55469	36154.74219	23551.63086	21195.55273	25127.30469	22421.37109	29005.90234
30537.33203	28952.1582	25799.08203	26144.05664	31099.78125	17595.60352	30863.07813	29905.78125	27890.38672	28933.14063	31108.12109	24930.08008
32650.65039	25138.25781	30914.53906	20460.9375	21973.43164	19664.49219	32060.27344	19154.55664	33305.97656	29139.65625	31690.18945	26181.25586
26990.83789	24300.79883	27667.08203	38819.72266	41388.14453	21711.08984	30744.08984	25752.82422	21918.47266	20682.9375	35402.91016	29222.0293
22844.71289	21485.03711	25155.22266	Filtered	Filtered	18538.40625	Filtered	Filtered	29223.30078	32178.50977	33069.75781	Filtered
Filtered	Filtered	Filtered	Filtered	12072.7207	14887.82813	Filtered	Filtered	11193.88477	4152.26123	Filtered	Filtered
20851.76758	Filtered	9906.831055	23954.31641	25112.75	10862.16602	86186.41406	Filtered	20436.9043	Filtered	5512.041992	33884.10547
Filtered	Filtered	Filtered	23401.55469	Filtered	Filtered	Filtered	Filtered	Filtered	Filtered	Filtered	Filtered
9706.103516	Filtered	6125.760742	Filtered	Filtered	55317.99609	22670.18555	26371.80469	16754.07227	39134.26563	152917	147468.1875
Filtered	Filtered	4255.623047	Filtered	Filtered	Filtered	8208.03418	11466.87793	8999.808594	7755.791992	9610.317383	Filtered
15578.55957	19455.19922	22420.77734	21556.55469	16824.86133	22884.14648	24836.63672	16955.14844	27428.96875	27048.21484	29429.23438	Filtered
23574.55078	17165.41406	17343.16211	20541.95703	16783.38477	18332.64063	21439.8457	25453.31836	30204.875	32210.89453	30479.14648	31252.77734
24422.30078	21077.4082	28194.25586	16014.18945	21378.62891	19924.32617	18756.38086	26120.53516	22549.13477	24167.64844	49342.67969	56105.73438
23195.36523	20041.25781	20486.75977	19742.96289	29272.57813	24553.76172	25028.18164	27458.76563	33700.10938	25619.36719	23633.35938	42096.87109
2180.732422	Filtered	33050.60938	21108.25195	11724.98535	37798.16797	6933.964355	3307.819336	4075.961914	15042.77051	7910.067871	Filtered
Filtered	11730.87402	58054.30859	7926.856934	24465.06641	33787.10547	Filtered	Filtered	Filtered	11175.09766	Filtered	Filtered
32935.72656	20057.51953	21168.0625	20879.63281	27391.89844	15386.99512	22233.89844	19666.51758	26355.93555	20501.19531	21105.47461	25954.02734
31327.33203	18596.5918	21722.85156	20176.80273	20021.52734	14953.15137	21339.40039	26770.59961	26218.55078	22964.06641	22990.24219	25678.83789
19412.6582	15012.49707	17805.68555	17669.80469	23701.93555	27814.1543	24508.13477	16047.66113	25051.0957	17737.90625	19197.75195	15355.07715
Filtered	Filtered	Filtered	Filtered	Filtered	Filtered	Filtered	Filtered	Filtered	1262.759033	Filtered	Filtered
15876.02637	26714.92578	14378.67676	21691.05664	24134.25	20355.37109	26650.68164	25466.74414	22962.41406	26937.92773	20091.49414	21778.58984
Filtered	Filtered	Filtered	Filtered	Filtered	Filtered	Filtered	Filtered	Filtered	Filtered	Filtered	Filtered
27785.27734	17285.9375	20210.62305	23799.26953	21928.74609	17375.49219	22935.23828	24900.39453	23214.30078	21398.45898	21537.17578	25653.56055
25644.92383	22254.97461	20314.01953	21362.43359	21551.22266	18310.84766	22966.99219	21480.06836	23592.03125	24823.05664	32954.6875	20873.79492
21742.68359	15528.06641	11196.7998	26221.89453	12316.55859	17820.88672	24791.33984	27004.77539	40582.14063	26605.875	31041.02539	Filtered
10004.2666	13046.11523	8880.4375	18178.5332	35613.72656	31049.17578	13128.80957	15246.62207	10862.12305	7620.584473	9078.379883	5499.351563
Filtered	Filtered	Filtered	8729.901367	31843.76367	38940.24609	Filtered	Filtered	37243.34375	Filtered	Filtered	22266.91016
26287.875	19786.51953	16361.93457	22764.55469	15163.71191	12206.09473	21854.53711	30742.42773	18356.11133	42365.69922	24773.43359	53545.74609
Filtered	5943.978516	7289.525391	8358.837891	Filtered	6982.347168	8224.447266	8914.918945	17451.68164	10781.3418	14736.96875	Filtered
21308.24609	15963.30469	14147.09375	23261.4707	23833.98047	7893.770508	11827.69238	9070.319336	20071.40039	18100.68555	24186.02344	49522.14063
28640.52148	15775.93066	20983.67383	21196.1543	20729.98047	14889.24805	22741.61523	24799.54492	26993.21094	25416.58008	22231.16406	23005.57031
21468.16992	18915.96875	100453.8516	20659.64648	12922.50391	74628.95313	8045.40332	6073.727051	Filtered	7702.543945	15980.23242	16758.875
Filtered	8102.665527	Filtered	5639.017578	Filtered	Filtered	16084.49023	12082.69336	13045.33887	13702.8584	20587.93945	Filtered
13362.15918	17838.68555	19872.63281	17539.27148	14761.50293	36450.02344	34942.22656	20920.88281	13458.55664	19348.73438	45005.91797	29526.60547
30137.16406	15150.85547	20799.36914	21457.5918	22868.35938	14080.73926	16919.8125	14548.06445	25205.73828	23827.86914	23327.96875	20178.46875
28686.99609	14220.85547	15644.01563	18720.40492	19250.79297	9328.216797	20312	21854.27734	39594.18359	23109.56641	27486.44336	27553.92773
35420.24219	25763.76172	33249.03125	14975.42188	19603.90625	22173.87695	36300.55859	15912.26758	32387.32031	8094.211914	77673.40625	16662.31836
6112.300293	24327.29102	17604.71875	18367.48047	25913.83984	26575.41016	20529.03125	31508.25781	27896.09961	30331.93945	23166.13867	Filtered
33847.09375	15056.92578	24079	15863.1123	19995.41211	15849.10742	18948.35742	20288.24219	32745.11523	18879.02539	18886.88867	20095.52344
Filtered	14112.24023	Filtered	17180.41602	18778.47656	8903.947266	23421.17773	134643.0156	19419.73828	22479.50586	Filtered	31833.8457
22710.60742	19481.49219	22950.7207	19933.6875	22267.08008	20202.07813	20382.87109	22124.97266	14514.8916	22150.49414	19283.51172	29941.19531
Filtered	Filtered	Filtered	Filtered	Filtered	Filtered	Filtered	Filtered	Filtered	Filtered	Filtered	Filtered
6938.408203	150080.8281	6200.375977	13883.35254	3846.118896	7039.817871	12687.17969	10486.20801	9035.59082	9092.189453	8476.115234	224413.5
20817.55664	20209.83008	14671.58691	18645.3457	18154.52734	15913.73047	22847.26172	24046.67969	29688.81836	20839.79102	14252.15527	41324.30078
Filtered	Filtered	Filtered	Filtered	Filtered	Filtered	Filtered	Filtered	Filtered	Filtered	Filtered	Filtered
18343.7793	12370.29688	16457.96484	15691.8125	16869.68555	12016.0918	15170.46387	33048.80469	30064.87305	12637.23242	17373.89844	22689.93164
23697.56445	16421.94727	23522.73438	4478.055664	22633.08203	17944.27148	20292.28516	5170.735352	6234.03125	22950.32813	8542.443359	30110.04492
22962.72656	19508.35547	15510.04199	22268.89453	15519.50781	13893.86621	13499.17188	22045.4082	24147.12305	26997.55469	15254.3457	18901.30273
Filtered	4584.415039	Filtered	Filtered	Filtered	Filtered	Filtered	Filtered	91498.34375	1029.085449	3354.03418	Filtered
24833.14648	26177.32813	14400.66406	20194.84375	14876.28223	20334.5332	21481.46289	22176.4043	14412.63379	27944.57422	18377.86719	Filtered
Filtered	9809.292969	33639.17969	Filtered	Filtered	59960.41406	Filtered	Filtered	Filtered	Filtered	Filtered	Filtered
14474.56934	22027.30859	16469.21094	18771.24414	15383.47266	17727.14844	23587.95313	20794.96094	24343.30273	23652.80273	25781.62305	27296.31641
24623.22461	16182.66699	19440.91602	20608.19531	17659.05078	19227.66406	19887.11523	21987.66016	27173.05078	23951.32227	19366.78906	16701.33594
37135.65234	21276.38086	Filtered	Filtered	Filtered	Filtered	Filtered	Filtered	38228.57422	Filtered	Filtered	Filtered
53102.11719	9500.858398	9551.224609	25911.62305	28777.34961	14800.33398	11113.1748	43845.40625	68524.71875	17381.66211	10718.48438	15233.3291
23464.51367	16838.58789	15921.60059	17149.24219	23109.26563	16300.57715	18612.89258	29006.66016	25068.28125	21218.14258	23224.88477	16216.1416
19205.02148	17848.57813	16663.31641	16312.00879	26241.49609	15181.89551	28136.08203	20496.85547	23819.37891	19096.75977	18729.72461	13819.36719
Filtered	Filtered	Filtered	Filtered	Filtered	22121.88477	Filtered	Filtered	Filtered	Filtered	Filtered	Filtered
10457.68945	9005.891602	54823.03125	Filtered	4292.312012	53726.53125	Filtered	Filtered	Filtered	Filtered	Filtered	Filtered
24560.86328	14918.99707	20454.1875	20961.33789	9484.264648	17422.87109	20410.70703	16757.07422	24922.7832	19394.71484	16391.30273	10590.48535
18754.41406	15218.29199	38141.875	7828.597168	5005.724121	59257.21484	Filtered	Filtered	Filtered	6815.54541	Filtered	Filtered
9900.948242	6082.155273	Filtered	Filtered	Filtered	45099.84375	Filtered	Filtered	1371.819336	Filtered	Filtered	Filtered
13868.31543	17094.27148	Filtered	Filtered	Filtered	8405.791016	9784.339844	Filtered	Filtered	Filtered	Filtered	Filtered

23464.38672	17367.16016	21813.87109	22673.19922	16401.83203	16245.95703	21479.10938	18038.99609	20447.4375	21330.84961	21802.45117	11556.99023
20082.31055	14713.94629	18465.08398	Filtered	19771.41406	Filtered	16295.43945	Filtered	29778.03516	15848.93066	18599.24023	20244.32813
19158.76953	19678.69922	15273.36719	14572.76367	16199.61816	10734.20117	17910.73828	20297.75195	20565.05078	19045.38281	14963.32324	46376.21094
18663.68555	18595.35156	13179.41895	16640.99609	18465.44531	16272.89648	19651.06641	25189.74609	21659.71875	20378.19922	15675.56055	Filtered
21177.65234	19577.05469	15226.08691	14029.71289	14309.7168	24798.9082	13996.5127	14087.40723	21516.5918	24716.0625	21043.6582	28105.28516
12698.53418	12746.86035	15990.65137	16697.62695	21025.09961	10067.12988	Filtered	42793.44922	Filtered	Filtered	59862.52734	Filtered
16411.08203	51424.37109	9784.172852	22609.82813	18250.45703	18393.23438	12995.08398	34511.72656	17549.60742	16766.55273	18771.07813	13054.66113
20548.90039	10142.03418	13813.75879	17098.45703	10591.13672	11876.30078	20465.26563	14263.55176	12251.875	14222.3623	14307.39258	23764.67188
Filtered	Filtered	Filtered	24165.36719	18300.78711	Filtered	Filtered	Filtered	Filtered	Filtered	Filtered	86975.15625
31336.57422	23974.64063	14294.62012	7383.108398	8846.84668	6169.146484	9602.512695	10919.15625	6168.578613	17236.10938	21574.51172	54551.21875
24346.75586	22088.79297	14669.32813	18386.62109	17056.76563	15142.31055	16897.54688	20380.46094	18835.8125	19630.98828	15385.90527	16226.25391
Filtered	25043.41406	24404.74805	21942.62695	34257.58594	11681.3291	Filtered	24590.49805	Filtered	Filtered	Filtered	Filtered
4285.295898	19619.13672	3874.678467	4283.878906	8833.657227	43488.35156	3470.235596	42975.38672	34423.30078	47217.08984	43477.51953	3528.771484
7417.389648	3374.144531	5561.372559	4596.041016	6518.122559	3970.059082	4080.539063	5111.625977	5037.842285	4264.607422	6274.81543	2600.231445
27437.75977	19682.49805	15263.45703	19508.68555	19261.64648	15342.52441	16219.02832	19100.08594	19457.05469	22757.32422	22383.63281	37595.76563
21120.05078	16809.97266	17459.92188	23127.66797	24914.79688	31601.84766	12336.11816	28511.06641	16370.0957	12402.77344	24739.14648	31847.81836
12559.60156	18450.04688	17510.10938	19037.4375	14285.05566	15644.0498	19513.40625	21222.56641	21821.8125	18406.48438	21340.60352	10399.10938
14724.35449	17708.80469	14386.21484	20414.35352	12829.25	11542.37695	21324.50391	14959.17383	28515.49023	17602.04883	35106.48828	19538.27148
Filtered	17280.49609	42925.76563	9065.698242	Filtered	12465.27344	Filtered	Filtered	Filtered	6360.737793	Filtered	Filtered
19490.39063	11241.70996	14626.85449	12335.46191	15725.00488	11685.03906	16304.69043	11161.24414	22863.59375	22483.50781	30248.875	17309.79688
18523.85156	21595.54102	16080.11426	11885.57813	11557.99902	17924.38867	14253.57324	21603.76367	15529.41211	19381.55469	15686.75781	26115.94141
23944.08984	13653.26563	15269.61621	8972.806641	14793.41406	6550.740723	18041.04102	15688.33398	24714.71484	21709.35547	19902.14258	Filtered
22523.25781	27876.19531	14323.23145	20773.41016	16208.78906	15546.94434	15218.72168	21920.75781	17737.01563	17953.00977	10681.1377	12798.39453
Filtered	Filtered	21302.77734	Filtered	Filtered	24405.88867	Filtered	Filtered	Filtered	Filtered	Filtered	Filtered
9077.546875	12832.10352	54918.50391	8717.939453	7458.820801	65476.59766	7097.677734	Filtered	Filtered	6501.866211	Filtered	Filtered
14176.98242	7271.885742	11701.79492	12457.77148	14759.0752	17731.91016	12974.31055	11777.3252	9483.362305	11742.36816	7712.177734	Filtered
19007.30078	15502.9082	14310.8916	15215.56934	17749.9043	20645.57031	15861.1416	15909.89258	19008.99805	18960.38281	29681.33398	21683.47656
21398.1543	Filtered	Filtered	17810.81055	11861.64844	11294.10742	Filtered	15664.27344	27187.8125	Filtered	Filtered	Filtered
15805.2168	7050.409668	7035.758301	14576.65137	15442.19141	18764.3125	8597.118164	16323.92285	16124.44336	14348.17773	13076.50879	13691.4209
27842.92188	15164.09961	14906.36133	11611.9668	15454.80469	13185.62109	12325.10059	17896.96094	22364.57813	12720.0293	15371.91406	Filtered
Filtered	Filtered	Filtered	Filtered	Filtered	Filtered	Filtered	Filtered	Filtered	28417.41992	Filtered	Filtered
9586.643555	13416.00391	81118.94531	11108.78125	11058.94336	127722.6797	3763.094482	5827.574219	3099.313721	8001.607422	16542.54883	16181.82422
19502.14063	14927.36523	14278.24219	13368.42773	16633.39844	10172.01953	12649.95703	15627.91699	18583.22266	30185.41406	18126.15039	Filtered
Filtered	4556.501953	23369.40625	5362.622559	2335.357178	18807.13867	32554.32422	12678.66309	4758.428223	2408.296631	40166.13672	17011.93555
36416.85547	9206.384766	18330.73828	16617.45508	13178.26367	8491.973633	21302.06836	14583.31836	20376.00195	18747.73047	25306.61133	7161.723145
16968.18555	16482.94922	11891.38281	12936.52637	12947.91992	9993.171875	18453.39258	14662.22949	15492.54297	15788.84277	17455.62109	32594.17383
Filtered	Filtered	Filtered	Filtered	2459.70166	4937.875977	Filtered	Filtered	Filtered	Filtered	36156.47266	Filtered
15169.125	14589.97852	10600.62891	18616.91016	14660.00098	16645.88672	17854.53711	15764.95215	18252.78906	12675.53516	10854.16016	Filtered
Filtered	15602.80664	16435.07422	Filtered	22692.44141	11284.07031	10088.64063	17826.43359	21777.28125	14772.43066	26294.85352	Filtered
19173.70703	11746.03418	13039.13672	18612.77344	15626.95996	10837.22754	17792.62695	16876.42773	14126.68457	16584.69922	17980.34766	18919.02539
Filtered	13668.2373	Filtered	Filtered	Filtered	Filtered	Filtered	4809.494629	4067.751709	89437.80469	4435.94043	Filtered
9178.710938	11592.62695	12968.51855	10714.48047	11787.06445	8591.725586	12504.09375	6795.256836	11986.07813	15942.58008	10263.24219	8703.125
3248.99585	4825.177246	2206.344727	Filtered	28524.13281	Filtered	2401.77002	5727.999512	13526.74902	Filtered	Filtered	3978.428467
12694.35352	16415.55859	19970.75391	8593.936523	9255.414063	8228.301758	18828.7832	17557.26367	25277.17773	12163.96484	17205.37695	18355.89063
Filtered	Filtered	Filtered	Filtered	Filtered	Filtered	2492.277344	4321.242188	5842.713867	4226.155273	7395.034668	Filtered
13989.44629	11509.22656	61654.95703	9129.601563	10644.91602	68236.39063	10679.15137	10818.80957	16452.13477	18008.73828	9441.321289	Filtered
Filtered	Filtered	Filtered	Filtered	Filtered	23018.22461	Filtered	Filtered	Filtered	Filtered	Filtered	Filtered
Filtered	Filtered	Filtered	34018.67969	Filtered	Filtered	Filtered	Filtered	Filtered	Filtered	Filtered	Filtered
23481.39648	15506.07422	14961.02148	10115.60059	15653.39844	13184.54688	12246.77539	16659.9082	21544.65039	17243.45508	14424.93457	13421.93066
15367.5957	36899.03906	8181.416016	20712.10352	16713.06641	21137.50977	11072.78223	28847.37305	17339.49023	17565.73438	18203.88867	15499.27344
Filtered	13981.10645	14239.56934	6948.616211	16527.52734	7938.449707	Filtered	12835.68945	16635.16797	16770.90625	13873.71191	Filtered
14755.93457	14825.75977	13074.29492	10454.51855	7871.196289	7960.712891	16942.23438	22262.06641	22975.4043	15509.22656	23250.66016	73161.88281
38494.98438	15886.94531	16217.5127	12456.03223	13068.7666	11733.34082	15422.49219	17715.55273	12077.54199	9865.425781	15703.18457	19789.43555
Filtered	Filtered	1611.545898	2481.674072	556.8078613	3003.485107	Filtered	2582.946289	Filtered	35261.375	85288.95313	Filtered
15349.41602	22067.47461	13456.33691	13093.75977	14404.48438	9201.855469	15722.31055	28380.3125	17273.94727	22088.07813	15560.0293	26390.62305
9959.135742	10386.35547	8316.041992	10169.42969	14535.52246	6356.329102	12241.91016	12346.87598	10697.30859	13977.59766	11532.2168	38649.16016
18838.61914	14622.19336	15921.95605	15742.42285	16542.51367	8080.968262	18236.15234	14781.34277	14407.35352	20418.68359	14430.21484	15403.76465
Filtered	8710.332031	18524.41016	Filtered	Filtered	20840.91602	Filtered	Filtered	Filtered	6392.997559	Filtered	Filtered
16188.05078	13122.75195	12874.71387	12351.21289	15022.99805	12711.69043	11481.23438	20528.05859	16760.88281	16599.21484	16797.43555	24729.86133
Filtered	19632.42383	48654.36719	Filtered	Filtered	38501.44922	Filtered	Filtered	Filtered	Filtered	Filtered	Filtered
9902.740234	10591.05273	31044.48633	Filtered	7309.192383	5231.226074	Filtered	Filtered	Filtered	Filtered	Filtered	Filtered
7910.748047	19691.9043	8583.666992	6711.795898	15637.13574	9293.62793	13000.99707	24317.01953	14349.94824	14253.60547	12637.37598	16491.46484
17958.90234	16298.53711	12518.05371	15142.73828	23081.08203	Filtered	16857.44336	19099.71094	18393.94922	11528.86328	12487.62012	14935.93848
19390.01758	14559.60938	16355.74805	12535.47754	10284.08789	5905.533203	15306.58008	14155.7832	24636.46094	12134.3623	11583.73535	Filtered
13385.39648	9433.270508	6304.001465	10925.36426	11933.52734	22583.20313	18119.58789	16648.60156	14297.51563	18336.51172	13454.07813	15901.00781
Filtered	Filtered	Filtered	Filtered	Filtered	15864.61133	Filtered	Filtered	Filtered	Filtered	Filtered	Filtered
18179.83594	Filtered	56691.44141	Filtered	Filtered	15073.08496	Filtered	Filtered	Filtered	6740.336426	Filtered	Filtered
Filtered	Filtered	Filtered	Filtered	Filtered	Filtered	Filtered	Filtered	Filtered	Filtered	Filtered	Filtered
Filtered	Filtered	Filtered	Filtered	Filtered	12432.61133	36346.30078	Filtered	Filtered	Filtered	Filtered	Filtered
Filtered	14436.03418	3757.880371	Filtered	5664.884766	17049.92578	Filtered	Filtered	Filtered	24497.07422	7267.554688	Filtered
18479	Filtered	Filtered	8791.28418	Filtered	Filtered	Filtered	Filtered	Filtered	Filtered	Filtered	Filtered
17180.12891	13104.77832	13082.9082	10016.37012	13866.70801	10350.24609	13420.57422	14915.88477	18428.62109	16402.07422	15250.57227	17176.47461
Filtered	Filtered	Filtered	Filtered	Filtered	Filtered	Filtered	Filtered	Filtered	3982.461182	22721.16211	Filtered
12148.80371	11026.5498	11225.23242	10096.46875	13226.50195	15054.89355	9632.791016	14693.98633	12236.72266	16746.50586	10485.0293	20917.53125
19790.17969	Filtered	Filtered	Filtered	15772.50391	Filtered	22030.64258	2651.923584	17690.20313	14711.65234	Filtered	Filtered

1511.563843	3521.586426	1531.863647	5037.933594	3699.333008	3541.56543	Filtered	2581.646484	Filtered	4879.088867	Filtered	Filtered
Filtered	Filtered	Filtered	Filtered	Filtered	2503.505127	5308.898926	Filtered	3004.624268	Filtered	Filtered	Filtered
26570.53516	16045.41406	19489.38867	19026.19727	10233.4873	12444.44434	10949.19043	14029.32422	7748.661621	15719.83887	17230.89453	Filtered
9920.152344	10477.8291	11584.16113	14921.64746	10347.27051	11151.67676	12600.93164	15335.63086	14776.75586	10512.01953	14792.99414	14771.00488
10209.49219	6363.489258	4553.31543	4586.768555	Filtered	4069.842773	5515.212891	3320.389648	64323.21875	6073.838379	7811.73291	Filtered
9496.702148	11446.30859	33676.65234	9765.185547	5656.404785	37998.34375	9050.047852	3698.558594	4559.813477	6374.709473	6260.152832	Filtered
22001.94141	12392.2959	14052.62695	10134.29785	13101.52051	10040.48242	12078.16309	9723.695313	21846.64844	10856.85742	14686.4082	Filtered
15573.68848	11925.9082	12133.36523	13647.77832	12293.23633	8864.047852	8535.873047	11463.66992	19676.93359	13194.12988	14618.33887	Filtered
21187.25	9467.396484	12825.1377	9333.296875	12178.40234	8721.004883	14804.04004	12043.76465	17537.13281	14187.94141	13014.75195	12844.80762
8889.063477	17064.57617	11149.05469	10772.87109	13088.22266	8694.810547	7937.618652	15566.97168	9114.498047	6339.452148	12415.67969	12240.91211
4443.666016	7615.28418	13737.33691	8201.985352	29653.35938	23746.18359	7881.788086	21314.74219	15708.56445	12373.5459	6618.776367	Filtered
Filtered	2.06E+04	Filtered	4.94E+03	Filtered	1.42E+04	Filtered	Filtered	Filtered	5.65E+03	Filtered	Filtered
16628.44141	12422.1709	11851.45996	10922.3291	12921.52246	9357.479492	11591.48926	11170.72168	13483.08203	16745.83008	11635.00586	6281.620117
Filtered	Filtered	Filtered	3050.772949	Filtered	Filtered	5610.812988	Filtered	Filtered	Filtered	Filtered	15662.58496
13172.41016	14674.92676	12101.60352	10810.63965	10559.29102	13653.07813	11727.08398	15363.96387	10904.44238	12341.35449	13269.3457	Filtered
Filtered	11283.75488	Filtered	11188.61328	18916.70703	14355.16406	Filtered	Filtered	10473.50488	Filtered	13999.93457	Filtered
Filtered	13988.90527	Filtered	Filtered	Filtered	Filtered	Filtered	5242.985352	15932.47852	Filtered	Filtered	Filtered
7210.38916	7948.10498	7099.930664	6478.255859	7618.068359	4990.088379	6995.878906	7005.006836	5953.245605	6065.617676	6534.334961	354970.6875
13722.74512	10778.66797	8676.669922	14638.6123	9338.739258	9486.12207	11482.28418	12484.78809	12974.54199	13662.3623	12985.07227	11270.03809
Filtered	5106.916504	13419.02246	Filtered	Filtered	Filtered	Filtered	Filtered	Filtered	Filtered	Filtered	Filtered
Filtered	Filtered	Filtered	Filtered	Filtered	Filtered	Filtered	Filtered	12395.50684	10365.3623	11579.35938	Filtered
5078.618652	Filtered	3500.596436	3130.487061	Filtered	2914.751953	Filtered	3154.000977	4840.693848	3686.539551	3821.086914	164624.0625
Filtered	Filtered	Filtered	Filtered	Filtered	Filtered	Filtered	Filtered	Filtered	Filtered	Filtered	Filtered
24429.83984	11703.26953	9856.03125	16701.53906	12249.77539	2067.042969	9690.359375	9187.332031	15648.8457	10561.19727	8533.222656	10595.54102
Filtered	Filtered	11278.34473	Filtered	Filtered	8735.149414	Filtered	13094.69141	9898.65625	16613.41406	Filtered	Filtered
Filtered	Filtered	12949.24414	Filtered	Filtered	16576.59375	Filtered	Filtered	Filtered	Filtered	Filtered	Filtered
6450.149902	24054.83008	135204.8594	4856.791504	5649.257813	Filtered	8143.889648	14153.52832	9364.15625	4511.408203	11608.85547	Filtered
9512.06543	9813.967773	14944.89746	8299.959961	10123.71875	10022.96289	8791.491211	11455.36621	10777.84863	13185.36328	11459.85938	Filtered
13395.31543	10053.89941	11371.94629	8074.280273	9645.214844	12355.65527	14190.27344	7573.679199	13502.87598	15169.03418	9020.527344	10170.47363
7648.703613	14040.10547	5446.479004	Filtered	Filtered	Filtered	Filtered	42642.07422	Filtered	9258.478516	Filtered	15191.32715
10381.61426	12565.92578	8725.776367	12659.7002	8385.838867	7134.600586	10877.92773	14050.02246	10228.46387	7803.260254	13271.66602	26929.05469
Filtered	Filtered	Filtered	Filtered	4713.422363	Filtered	4570.914063	Filtered	Filtered	Filtered	Filtered	Filtered
19096.86914	6175.674805	12826.11035	5701.870117	6420.645508	4906.333984	15481.88281	3979.791016	10366.90625	6445.708496	5155.705078	Filtered
12373.16699	7816.944824	6844.642578	6538.335938	8204.942383	12047.14648	9935.430664	4047.507324	13068.82813	6438.387695	8790.066406	Filtered
8882.112305	13815.69727	8656.666992	8148.233398	13438.89941	13588.49414	11422.69727	11495.73926	7181.756836	7909.381348	Filtered	Filtered
14707.52344	10672.29688	9573.598633	Filtered	10305.04492	Filtered	11453.21387	Filtered	Filtered	6181.640625	6103.589355	Filtered
21118.53906	11881.17969	9257.922852	7748.399414	1521.61853	Filtered	1768.896362	12655.64746	6282.115723	9933.40332	5578.220215	Filtered
4798.702637	4670.40625	3869.435547	1836.108643	2202.614746	2549.662354	Filtered	2124.926758	6373.463867	4000.26416	Filtered	Filtered
3903.35498	2669.602783	5142.650879	2323.138184	37871.01563	7666.146484	2804.287354	6799.02832	47370.4375	7356.544922	3756.715332	Filtered
10442.36719	16371.01563	8152.087402	9555.759766	7068.358398	6451.675293	13002.4082	17081.23047	14007.43848	10984.08203	13131.97656	16107.21582
Filtered	Filtered	Filtered	Filtered	Filtered	Filtered	Filtered	Filtered	7642.18457	Filtered	Filtered	Filtered
5991.331543	Filtered	8899.841797	Filtered	6188.287109	14364.15527	Filtered	2884.354248	4756.237793	9543.375977	3636.060059	Filtered
Filtered	Filtered	Filtered	Filtered	Filtered	10088.41797	Filtered	Filtered	5054.340332	Filtered	Filtered	Filtered
13581.58398	11456.13965	7776.394531	9936.419922	12653.64844	8211.945313	13069.79883	17113.45703	11370.35352	12536.13477	15225.77246	15197.54004
Filtered	Filtered	Filtered	Filtered	Filtered	Filtered	Filtered	Filtered	Filtered	Filtered	Filtered	Filtered
17656.36328	Filtered	Filtered	Filtered	5828.74707	Filtered	Filtered	Filtered	7416.206543	Filtered	7490.995605	Filtered
Filtered	8697.625977	13626.91504	Filtered	Filtered	15810.87402	Filtered	Filtered	7407.040039	Filtered	Filtered	Filtered
14030.81348	31697.00195	6541.534668	7005.509766	8028.582031	2272.108887	5697.292969	77680.49219	5611.578613	24492.35742	5662.466309	18183.88477
10056.72656	24638.8125	9652.867188	8681.760742	9952.848633	3660.572021	7917.645508	27003.4707	14918.41602	10395.58789	8290.759766	20396.07813
10977.57422	8332.876953	11689.91211	8577.977539	14642.9668	6801.818848	11966.02734	10929.11328	11733.56641	10777.17188	9666.730469	10227.98145
18052.43945	6875.352051	8846.839844	7984.123535	8632.668945	7193.670898	16663.79492	11185.57617	12932.68164	13580.90625	8809.897461	Filtered
6708.754883	7168.811523	11689.18359	7395.226563	13466.26855	11871.77832	8179.860352	9472.245117	16860.26953	11823.2998	12713.23242	Filtered
12844.94727	8256.678711	9851.293945	8591.085938	9201.783203	8223.295898	8567.613281	9672.192383	15230.02344	10576.81445	10233.73828	19066.74023
10534.87891	12243.45898	11099.11328	11752.31445	11756.29883	11105.98145	14936.58984	10428.1416	9501.056641	11537.87305	19996.04297	10337.60449
21679.97461	3247.007324	Filtered	Filtered	Filtered	Filtered	Filtered	14346.64063	12603.65039	3026.196289	Filtered	Filtered
8370.03125	9071.732422	10867.91016	8387.831055	11481.81348	10710.16211	8474.594727	11116.62988	23144.27344	9101.011719	6839.041504	Filtered
5243.30957	Filtered	Filtered	6785.78418	Filtered	Filtered	10083.42285	12574.96875	16138.87402	7975.333008	14559.0625	Filtered
14333.80859	9163.685547	14672.69922	8814.25293	6951.720703	6414.600586	14229.88672	16253.53906	19267.33984	11712.86523	14141.69043	Filtered
Filtered	1412.683716	1461.013062	Filtered	1825.248535	3270.82373	11754.79102	7256.517578	12990.60156	8341.679688	19592.94336	Filtered
7407.553223	6577.593262	10100.88477	8301.291992	8779.060547	3362.02417	12563.51172	43625.13672	13529.14063	11242.60156	11211.52734	Filtered
Filtered	Filtered	Filtered	Filtered	Filtered	Filtered	Filtered	Filtered	7249.613281	5781.045898	Filtered	19577.07227
5734.916992	8485.203125	Filtered	6068.118652	5270.977539	3863.889648	Filtered	1524.446167	Filtered	12746.29297	Filtered	Filtered
13936.1416	12744.00586	9072.501953	7861.613281	11136.54883	9376.399414	10442.54102	9436.616211	12558.13574	9999.819336	7271.257324	12672.35352
1634.060791	4433.597168	2490.419678	3024.365723	3422.802734	1895.736206	3523.337402	3177.222412	3702.595947	5474.817871	2919.376221	Filtered
10886.91016	13387.41699	6472.285156	10845.23438	7155.795898	13552.22852	13304.7041	6557.51123	9372.039063	12821.73828	10638.34277	12536.7207
1504.083008	2581.395264	17927.23438	24573.5918	3777.83667	1483.226318	1200.998779	3413.543945	37869.65625	31266.75	13426.70508	Filtered
Filtered	Filtered	Filtered	Filtered	1565.317017	Filtered	4332.033203	7638.069336	3867.270508	3502.9646	14575.70703	Filtered
8138.039063	7011.910156	9993.545898	9593.387695	6354.640137	8091.419434	6934.770508	7181.552246	9639.358398	10756.18555	13902.70313	Filtered
12104.24023	Filtered	Filtered	Filtered	Filtered	8936.720703	9411.508789	Filtered	Filtered	9664.021484	Filtered	Filtered
4628.963867	16770.86523	13242.36426	4394.67041	11187.7793	9481.888672	10515.39844	16438.17773	12384.22266	6236.203613	5375.990234	22444.00586
5583.45459	4926.341309	5060.429688	3916.560303	5526.012695	5533.387695	5821.055176	5316.4375	5004.791016	6776.678711	4102.570313	120412.75
18499.70508	6757.007813	8106.544922	6333.720703	5328.024414	7034.545898	7647.300293	5538.342773	8709.588867	22501.02734	6452.086914	22023.45117
Filtered	Filtered	Filtered	Filtered	Filtered	Filtered	Filtered	Filtered	Filtered	Filtered	Filtered	Filtered
Filtered	Filtered	Filtered	4200.600586	Filtered	Filtered	Filtered	Filtered	Filtered	Filtered	Filtered	Filtered
Filtered	Filtered	Filtered	Filtered	Filtered	Filtered	Filtered	Filtered	Filtered	Filtered	Filtered	Filtered

9342.474609	9349.297852	Filtered	7551.618164	Filtered	Filtered	6773.475586	16453.49023	9027.390625	11965.51465	11561.91797	Filtered
9979.036133	Filtered	Filtered	Filtered	2434.22168	Filtered	Filtered	6801.144043	3858.351563	9056.575195	Filtered	Filtered
Filtered	14138.37695	10343.32324	5083.054199	13102.32129	20838.17578	5027.585449	15137.7002	Filtered	9842.275391	Filtered	Filtered
Filtered	Filtered	Filtered	15811.74414	8641.121094	Filtered	Filtered	Filtered	6842.432129	Filtered	Filtered	21497.39648
Filtered	Filtered	Filtered	Filtered	Filtered	Filtered	Filtered	12822.97461	Filtered	2952.514893	Filtered	Filtered
13503.02344	2694.449707	Filtered	10528.33105	Filtered	6728.354492	Filtered	3293.273193	10198.43652	9345.749023	Filtered	Filtered
Filtered	Filtered	Filtered	Filtered	Filtered	Filtered	Filtered	Filtered	Filtered	Filtered	Filtered	Filtered
Filtered	Filtered	Filtered	Filtered	Filtered	7138.121582	5454.685547	Filtered	Filtered	7592.323242	6226.821289	Filtered
8551.920898	12152.58105	5529.258789	5787.31543	6735.495605	4016.032959	7993.668945	14964.22852	6869.792969	6797.895508	9146.666016	9039.786133
15468.37793	5371.185547	6472.265625	7231.647949	6661.488281	6143.237793	10325.55176	9373.188477	7816.303223	13630.4082	11836.24609	Filtered
9689.669922	10753.7627	8221.942383	Filtered	9428.94043	9902.959961	9974.125	13735.49707	12581.34961	10896.7832	10968.76367	Filtered
7082.851074	Filtered	Filtered	6371.128418	5811.131836	Filtered	Filtered	4598.383789	14484.51367	9568.238281	6582.8125	Filtered
Filtered	Filtered	Filtered	Filtered	Filtered	4736.102539	Filtered	Filtered	Filtered	Filtered	Filtered	Filtered
15407.72559	6853.714355	4840.434082	5613.071289	6024.437988	11040.47949	10451.26758	5743.361328	17093.60938	8975.609375	6770.425293	Filtered
8008.605469	6724.899902	6758.193848	5778.925293	9923.082031	6319.750488	6744.461426	8562.886719	8073.97998	12570.16992	7598.416992	18856.91211
6185.25	9879.021484	8832.960938	6308.515137	11287.68457	22514.33789	Filtered	7065.493164	11152.49512	19619.91406	9064.586914	Filtered
7550.13916	Filtered	9071.936523	4262.855957	15140.88672	10953.38867	Filtered	8002.072266	4734.533691	Filtered	5163.837891	Filtered
13634.92578	10741.68945	16356.43848	8503.584961	30149.27148	6442.727051	2205.728271	9665.341797	6932.716797	7119.84082	12596.58398	Filtered
Filtered	2222.369141	3829.481201	Filtered	Filtered	Filtered	2939.525635	2867.27417	22022.20703	18412.47266	Filtered	Filtered
14971.50879	4920.97168	8054.627441	Filtered	3659.696045	5645.210449	Filtered	6065.414063	12722.56348	7829.032227	Filtered	Filtered
Filtered	Filtered	Filtered	Filtered	Filtered	Filtered	8001.225586	Filtered	11674.40039	5746.439453	6134.166016	Filtered
Filtered	Filtered	Filtered	Filtered	2197.842529	Filtered	Filtered	Filtered	Filtered	Filtered	Filtered	Filtered
Filtered	7131.651367	Filtered	5645.020996	Filtered	4295.223633	3742.967529	Filtered	70086.63281	Filtered	Filtered	Filtered
15541.84082	Filtered	Filtered	Filtered	Filtered	4292.553223	Filtered	6821.451172	6968.355957	7295.824219	7900.854492	Filtered
4666.999512	18417.92188	7004.229492	Filtered	Filtered	1834.662598	5728.247559	8578.023438	Filtered	Filtered	9600.023438	Filtered
14320.49609	7313.916992	8325.24707	6090.88916	8301.261719	5699.028809	6848.246094	6219.189453	7830.276367	8435.061523	8031.791992	8148.381836
Filtered	Filtered	Filtered	Filtered	Filtered	Filtered	Filtered	Filtered	Filtered	Filtered	Filtered	Filtered
4656.262695	10289.42188	4492.163574	Filtered	Filtered	Filtered	Filtered	15096.9248	4143.808105	5433.016113	4191.343262	Filtered
Filtered	Filtered	Filtered	Filtered	Filtered	Filtered	Filtered	Filtered	5054.801758	6882.216797	9005.876953	Filtered
7729.160645	Filtered	Filtered	Filtered	Filtered	Filtered	Filtered	6846.165039	9451.431641	Filtered	Filtered	Filtered
3532.330322	Filtered	Filtered	Filtered	8026.467773	4812.525391	Filtered	2344.903564	3865.243896	7627.782227	Filtered	Filtered
Filtered	Filtered	Filtered	7680.309082	4633.04248	Filtered	Filtered	7658.174805	Filtered	5492.316895	Filtered	Filtered
Filtered	Filtered	Filtered	Filtered	Filtered	13516.1709	Filtered	Filtered	6237.959473	Filtered	Filtered	Filtered
10723.09375	5753.866211	10439.25684	5998.913574	7114.755859	6564.837891	5908.384766	5772.522949	7821.84668	7248.942383	8821.386719	6421.444336
12538.4707	7756.091309	Filtered	4594.59668	5331.088867	7443.83252	10524.57617	6300.584473	13514.8418	3296.246094	Filtered	Filtered
10701.00586	7883.123047	5500.09082	4927.152344	9363.832031	6474.691406	8398.3125	10372.73633	7085.680664	7471.820801	7007.509766	6372.342773
8307.866211	8132.488281	Filtered	5840.932617	4331.888184	9379.522461	6167.115234	5897.454102	14072.24609	7561.555664	11384.10547	Filtered
7943.22168	Filtered	Filtered	6673.959961	3885.208984	3647.621582	6990.015625	Filtered	5726.415039	6484.414551	4859.789063	Filtered
Filtered	Filtered	Filtered	Filtered	Filtered	Filtered	Filtered	Filtered	4521.996582	Filtered	7059.970215	Filtered
7700.609863	9982.358398	6463.808105	7690.118164	6367.865723	7259.319336	6261	13307.30078	8728.875977	7232.932617	6471.512207	7421.554688
Filtered	Filtered	6983.482422	Filtered	5885.767578	6337.655273	Filtered	8953.613281	Filtered	4707.992676	Filtered	Filtered
5811.144531	4947.270508	7309.933105	7507.739258	7118.611328	7625.976563	5736.492188	Filtered	8302.379883	8590.553711	8312.68457	Filtered
6550.226563	5594.75	4467.674316	2949.981201	3646.829346	1641.680542	Filtered	2728.09375	5338.487305	Filtered	6958.512207	Filtered
9144.984375	6129.362793	Filtered	Filtered	7008.33252	5481.11377	Filtered	8890.648438	7393.067871	9528.59082	Filtered	Filtered
16228.56543	Filtered	6276.89209	Filtered	7880.922363	Filtered	Filtered	Filtered	16494.3125	5348.898438	4244.154297	Filtered
Filtered	Filtered	Filtered	Filtered	Filtered	Filtered	Filtered	Filtered	Filtered	Filtered	Filtered	Filtered
Filtered	10864.72656	5038.489746	7320.491699	4435.554688	9804.154297	6118.941406	8151.931152	9680.177734	10957.07324	7577.911133	11834.45605
Filtered	Filtered	Filtered	Filtered	Filtered	10951.31738	Filtered	Filtered	Filtered	Filtered	Filtered	Filtered
10376.61133	6495.155273	7350.280762	4915.422363	Filtered	5331.680664	Filtered	Filtered	9008.567383	7700.153809	Filtered	Filtered
Filtered	3042.605225	2687.085449	2583.234131	2326.58667	Filtered	1883.936157	Filtered	Filtered	3629.166992	4400.010742	Filtered
Filtered	9376.21582	Filtered	5171.636719	4065.070557	Filtered	5907.536621	11435.71582	906.1893311	2584.31543	9019.304688	Filtered
6265.651855	7721.818848	8120.197266	6000.695313	8539.404297	6406.102539	6874.673828	9336.302734	9116.950195	7537.80957	7758.621582	10266.44141
27248.35938	7059.043945	5819.181152	7057.525391	2529.613037	Filtered	6020.008301	2176.905518	15949.17773	8402.28125	10415.30371	6512.261719
8581.744141	3164.579834	5641.007324	Filtered	5142.613281	11205.77734	4859.857422	Filtered	6303.147949	5981.189941	13109.78613	Filtered
13196.60938	Filtered	4379.714844	2692.049805	7859.900391	6388.034668	4550.860352	4506.356934	8700.933594	1518.263184	3379.953369	Filtered
Filtered	9240.951172	7025.420898	5934.861816	3613.372803	7279.775879	7021.952148	6805.464844	8589.605469	7834.263672	6022.589844	Filtered
3914.379883	9427.943359	2563.199219	8042.695313	Filtered	4208.732422	4348.492676	6111.870117	7619.952148	9915.950195	11570.7041	Filtered
Filtered	Filtered	7879.106934	Filtered	Filtered	13258.04199	Filtered	Filtered	Filtered	6281.696289	Filtered	Filtered
Filtered	Filtered	8494.489258	Filtered	6517.097656	5063.353516	Filtered	7830.089844	6749.161133	7839.461426	Filtered	9720.780273
Filtered	Filtered	7088.395996	Filtered	Filtered	Filtered	Filtered	Filtered	Filtered	Filtered	Filtered	Filtered
6080.066406	9879.34375	10325.4502	4005.296631	4741.762695	3213.608398	15460.94824	9987.303711	6587.887207	6065.395508	8597.133789	Filtered
Filtered	Filtered	Filtered	Filtered	Filtered	Filtered	Filtered	Filtered	Filtered	Filtered	Filtered	Filtered
Filtered	Filtered	Filtered	Filtered	Filtered	Filtered	1858.99707	5301.353516	6448.658691	5688.021484	Filtered	Filtered
6805.410645	6090.505371	5966.196777	5337.022461	7092.257324	5810.097656	6711.620605	4896.568359	7350.085938	7310.320313	5719.412109	Filtered
Filtered	Filtered	Filtered	Filtered	Filtered	2604.5354	Filtered	2445.011719	Filtered	Filtered	14251.66992	Filtered
Filtered	5512.716797	Filtered	Filtered	Filtered	Filtered	Filtered	10906.25586	Filtered	Filtered	Filtered	Filtered
Filtered	Filtered	Filtered	Filtered	Filtered	Filtered	Filtered	Filtered	Filtered	Filtered	Filtered	Filtered
Filtered	Filtered	2864.050293	6448.355469	7342.923828	7808.818359	Filtered	Filtered	6200.661621	Filtered	Filtered	Filtered
Filtered	4004.146484	Filtered	3401.008545	4582.768555	Filtered	4306.927246	6736.541016	Filtered	Filtered	Filtered	12682.72949
Filtered	Filtered	Filtered	17085.23438	9738.791016	4394.188965	4115.05127	Filtered	11765.11133	Filtered	Filtered	12928.17383
8471.989258	Filtered	6075.18457	5147.669434	3501.998779	4128.691895	7900.404297	4606.40332	12405.7207	4549.583008	6105.849121	Filtered
7023.705566	4903.983398	8618.791992	Filtered	3485.066895	5277.020508	5670.884277	6608.504395	6836.067383	5288.380859	10529.22461	Filtered
6272.887207	9072.804688	6075.172852	5419.902832	7428.894043	4421.299316	Filtered	5438.52002	Filtered	9000.337891	6426.930176	Filtered
Filtered	Filtered	Filtered	Filtered	Filtered	Filtered	Filtered	Filtered	Filtered	4798.356934	Filtered	Filtered
Filtered	Filtered	Filtered	Filtered	Filtered	Filtered	Filtered	Filtered	Filtered	Filtered	Filtered	Filtered

Filtered	5598.324219	7610.992188	Filtered	4419.501465	5238.265625	3983.147461	7188.078125	Filtered	7059.396484	Filtered	Filtered
994.2077637	16680.80664	Filtered	5247.379395	5143.21582	Filtered	885.7855835	10830.7334	Filtered	Filtered	Filtered	Filtered
2720.700439	7618.91748	5671.007813	7651.831543	6425.213867	8414.597656	2642.788574	5947.044434	3596.057617	7729.750488	7166.769043	Filtered
7005.754883	6474.576172	5625.010742	6373.727051	3198.580322	5227.179688	7639.777832	5090.925293	10494.89551	6504.338867	5944.241211	Filtered
Filtered	Filtered	Filtered	Filtered	Filtered	Filtered	Filtered	Filtered	Filtered	Filtered	Filtered	Filtered
Filtered	4982.407227	5482.32959	5777.355469	Filtered	3862.684326	5275.819336	6134.266113	6531.254395	6367.686523	Filtered	Filtered
Filtered	Filtered	Filtered	3900.119873	3371.206543	5730.52832	2491.239502	Filtered	Filtered	3248.583008	Filtered	Filtered
Filtered	10699.9375	6580.172852	Filtered	3130.224121	Filtered	Filtered	7046.636719	Filtered	3823.458008	Filtered	Filtered
Filtered	Filtered	Filtered	Filtered	Filtered	Filtered	Filtered	Filtered	9014.871094	Filtered	Filtered	Filtered
Filtered	Filtered	3163.572021	Filtered	Filtered	4265.790039	Filtered	Filtered	Filtered	Filtered	Filtered	Filtered
Filtered	Filtered	Filtered	8478.746094	2311.82251	6001.528809	Filtered	5820.633301	6444.476074	10852.30371	Filtered	Filtered
9764.628906	Filtered	5466.90918	Filtered	Filtered	Filtered	Filtered	Filtered	Filtered	3442.64502	Filtered	Filtered
Filtered	2992.330566	16062.49414	Filtered	Filtered	13277.14941	Filtered	Filtered	Filtered	2113.132813	Filtered	Filtered
Filtered	Filtered	Filtered	Filtered	Filtered	3835.092285	Filtered	Filtered	Filtered	Filtered	2484.797607	Filtered
Filtered	7730.768066	Filtered	3849.704102	7663.937012	6259.862305	Filtered	7818	Filtered	8243.834961	Filtered	Filtered
3199.977783	8533.297852	6365.330078	3071.035889	3773.105225	Filtered	Filtered	6499.543457	8200.255859	5177.895996	Filtered	Filtered
5067.172363	8374.139648	4916.476563	Filtered	Filtered	5356.441895	4449.174805	8576.864258	7424.521973	6140.583496	5966.13623	Filtered
8863.229492	6235.58252	6530.772461	6915.700684	Filtered	7616.527832	Filtered	Filtered	Filtered	6630.976563	Filtered	Filtered
8773.649414	11951.67285	5358.203613	3287.478027	3144.915771	9327.069336	Filtered	6845.533691	Filtered	7345.127441	Filtered	Filtered
Filtered	6165.482422	Filtered	Filtered	5882.348633	7751.517578	6425.866211	7771.021484	Filtered	6107.648438	3524.733887	Filtered
Filtered	Filtered	3701.195068	Filtered	Filtered	7795.745605	Filtered	Filtered	Filtered	Filtered	Filtered	Filtered
8367.439453	Filtered	6289.818848	Filtered	4965.074219	Filtered	Filtered	Filtered	7891.756836	4430.152832	5622.39502	Filtered
Filtered	Filtered	Filtered	Filtered	Filtered	Filtered	Filtered	Filtered	Filtered	20723.51563	Filtered	Filtered
Filtered	3854.772461	Filtered	Filtered	Filtered	Filtered	Filtered	Filtered	Filtered	2160.29248	Filtered	Filtered
Filtered	Filtered	Filtered	Filtered	Filtered	5695.955078	Filtered	Filtered	Filtered	Filtered	Filtered	Filtered
7089.848145	5055.321289	3801.98999	4598.769043	Filtered	Filtered	Filtered	4146.648438	2779.454834	5956.257324	3809.739502	Filtered
Filtered	6002.298828	Filtered	Filtered	3849.07666	Filtered	Filtered	Filtered	Filtered	7938.772949	Filtered	Filtered
Filtered	Filtered	Filtered	Filtered	Filtered	Filtered	Filtered	Filtered	Filtered	Filtered	Filtered	Filtered
3289.114502	2623.422852	Filtered	3430.742676	Filtered	997.1396484	Filtered	3920.414551	5250.61084	Filtered	Filtered	Filtered
Filtered	Filtered	8623.672852	Filtered	Filtered	Filtered	Filtered	Filtered	Filtered	Filtered	Filtered	Filtered
6480.342285	Filtered	4795.976074	Filtered	Filtered	Filtered	5052.064941	4352.367676	Filtered	4714.010742	Filtered	Filtered
3505.753662	4371.468262	4147.280273	5121.630859	4384.632813	3408.674316	4805.244141	5454.35498	4807.927734	5183.850586	2888.759277	7010.871094
Filtered	Filtered	Filtered	4776.003418	Filtered	4218.271973	3655.981689	4514.588379	5213.698242	4226.14502	Filtered	Filtered
Filtered	Filtered	Filtered	Filtered	Filtered	Filtered	Filtered	4173.382324	4096.90332	4560.867188	Filtered	Filtered
Filtered	7361.258301	Filtered	Filtered	Filtered	Filtered	Filtered	Filtered	Filtered	Filtered	Filtered	Filtered
6181.931152	8431.935547	4681.697266	3215.888184	5904.06543	1206.786133	6097.54834	9423.453125	3382.658203	4277.239258	4057.661133	Filtered
Filtered	Filtered	Filtered	Filtered	11307.65234	Filtered	Filtered	Filtered	6715.67334	Filtered	Filtered	Filtered
Filtered	Filtered	Filtered	4182.660156	Filtered	3111.51416	Filtered	Filtered	Filtered	5665.687988	Filtered	Filtered
3825.259033	5614.764648	5793.00293	5131.56543	5138.062012	4436.958496	6867.302246	7287.907227	6097.779785	6012.672363	6862.170898	7511.333008
Filtered	Filtered	Filtered	6408.750977	3244.429932	5438.376465	6314.080078	4559.925781	Filtered	4100.97998	Filtered	Filtered
Filtered	3394.149658	5561.204102	6418.406738	Filtered	1885.932007	Filtered	3307.686279	Filtered	Filtered	Filtered	Filtered
Filtered	Filtered	Filtered	Filtered	Filtered	Filtered	Filtered	Filtered	Filtered	Filtered	6560.106445	Filtered
Filtered	Filtered	Filtered	Filtered	Filtered	5380.273926	Filtered	Filtered	Filtered	5347.162109	Filtered	Filtered
Filtered	2583.301758	3109.956055	4521.197266	Filtered	Filtered	Filtered	Filtered	4912.835449	4664.830566	Filtered	Filtered
Filtered	7171.314453	Filtered	Filtered	Filtered	Filtered	Filtered	Filtered	Filtered	Filtered	Filtered	Filtered
Filtered	Filtered	Filtered	Filtered	Filtered	Filtered	Filtered	Filtered	Filtered	Filtered	Filtered	Filtered
4813.250977	Filtered	Filtered	Filtered	Filtered	2770.538574	Filtered	Filtered	Filtered	Filtered	4044.693115	Filtered
2569.467773	5372.695801	6215.315918	Filtered	4270.751953	Filtered	Filtered	9528.59668	Filtered	Filtered	Filtered	Filtered
Filtered	Filtered	Filtered	Filtered	Filtered	Filtered	Filtered	8274.771484	Filtered	Filtered	2406.660645	Filtered
Filtered	Filtered	Filtered	Filtered	Filtered	Filtered	Filtered	4396.445313	2434.963379	Filtered	6063.039063	Filtered
2961.758789	6444.058594	3080.998047	5230.68457	4117.79541	3220.188477	5307.80127	6954.00293	6799.62793	4911.733887	3959.179199	Filtered
Filtered	2574.970947	2959.850586	Filtered	Filtered	2275.049805	7365.369141	3089.632324	Filtered	Filtered	Filtered	Filtered
Filtered	6127.598633	Filtered	Filtered	3246.714844	2410.791016	5540.71875	7232.644531	3558.195313	3300.828125	5069.120117	Filtered
3527.842773	Filtered	6551.722656	4151.752441	Filtered	3845.099365	Filtered	3024.164795	4132.690918	3245.058105	5835.964844	Filtered
4445.486328	5346.100586	3199.838867	5486.199707	1534.168701	3583.72583	4101.652832	6071.720703	6743.928711	4538.866211	5136.844238	Filtered
6168.929688	Filtered	1687.318237	Filtered	Filtered	Filtered	Filtered	Filtered	Filtered	Filtered	Filtered	Filtered
Filtered	Filtered	5429.958496	Filtered	Filtered	3810.072754	Filtered	Filtered	Filtered	Filtered	Filtered	Filtered
Filtered	Filtered	6320.359375	Filtered	4625.421875	4803.683105	4695.786621	6410.021973	8311.598633	8798.206055	4054.062988	Filtered
Filtered	Filtered	Filtered	Filtered	Filtered	5268.877441	1822.416626	Filtered	Filtered	2610.236572	Filtered	Filtered
Filtered	5633.554199	Filtered	Filtered	4401.873535	3663.697998	Filtered	7109.180664	Filtered	5526.476563	3343.332764	Filtered
Filtered	4909.012207	2852.157227	5890.568359	Filtered	Filtered	Filtered	5697.554688	6219.116211	6006.286133	Filtered	Filtered
Filtered	6335.313965	2825.776123	Filtered	Filtered	Filtered	3333.056152	8017.268066	Filtered	3367.55957	Filtered	Filtered
Filtered	Filtered	Filtered	Filtered	1142.474731	7131.460449	Filtered	Filtered	Filtered	2874.908936	Filtered	Filtered
Filtered	Filtered	Filtered	Filtered	2389.005615	Filtered	Filtered	Filtered	Filtered	Filtered	Filtered	Filtered
Filtered	Filtered	Filtered	Filtered	Filtered	Filtered	Filtered	Filtered	Filtered	Filtered	Filtered	Filtered
1899.535156	6156.026855	3888.503662	3229.750732	3134.041016	5761.447266	4323.970703	4540.448242	6578.765137	4854.208496	7716.734863	Filtered
4537.585449	8576.750977	3122.205078	3359.977051	3702.578369	1709.381714	Filtered	9446.84082	4958.663574	3771.890625	3530.482422	Filtered
Filtered	Filtered	Filtered	Filtered	3727.85376	Filtered	Filtered	Filtered	Filtered	Filtered	Filtered	Filtered
Filtered	Filtered	Filtered	Filtered	Filtered	3774.60791	Filtered	Filtered	Filtered	1417.493896	Filtered	Filtered
4620.04541	4152.085938	3635.083984	2991.299805	5097.04248	4894.46582	Filtered	4747.765137	Filtered	4596.151367	4596.682129	Filtered
4020.794189	Filtered	2811.867432	2591.847656	2747.787109	4470.321289	Filtered	4070.785156	Filtered	3504.562988	4554.916992	Filtered
Filtered	4178.592285	5480.693359	3007.658936	5292.633789	2585.437988	Filtered	2820.501221	4985.761719	3531.01416	Filtered	Filtered
Filtered	Filtered	Filtered	Filtered	Filtered	Filtered	Filtered	Filtered	Filtered	Filtered	Filtered	Filtered
3134.919678	Filtered	3484.449707	4279.568359	6560.400879	3934.237061	Filtered	5693.025391	8524.896484	4199.21875	2718.28418	Filtered
4459.972168	Filtered	Filtered	Filtered	4954.727539	2585.506348	Filtered	Filtered	5598.34082	Filtered	Filtered	Filtered

Filtered	Filtered	Filtered	Filtered	Filtered	Filtered	Filtered	Filtered	Filtered	Filtered	Filtered	Filtered
Filtered	6330.156738	Filtered	Filtered	Filtered	Filtered	Filtered	3269.487793	Filtered	Filtered	Filtered	Filtered
Filtered	Filtered	3982.82373	Filtered	1952.178467	2790.448486	Filtered	3686.155029	4695.682617	Filtered	Filtered	Filtered
Filtered	Filtered	Filtered	Filtered	Filtered	2117.629639	Filtered	Filtered	Filtered	1764.243774	3210.529541	Filtered
Filtered	Filtered	2979.093994	Filtered	Filtered	9925.265625	Filtered	Filtered	Filtered	Filtered	Filtered	Filtered
Filtered	Filtered	Filtered	1961.173828	Filtered	Filtered	Filtered	Filtered	Filtered	3475.455322	Filtered	Filtered
Filtered	Filtered	Filtered	2834.006104	Filtered	Filtered	Filtered	Filtered	Filtered	Filtered	Filtered	Filtered
Filtered	Filtered	Filtered	Filtered	Filtered	Filtered	1715.165771	Filtered	1078.575073	Filtered	Filtered	Filtered
4743.650391	3280.698486	4049.435059	Filtered	4392.486328	Filtered	5188.92334	Filtered	Filtered	Filtered	Filtered	Filtered
Filtered	Filtered	Filtered	Filtered	Filtered	Filtered	2639.406006	3443.842041	Filtered	Filtered	Filtered	Filtered
Filtered	Filtered	2628.052246	1751.764038	Filtered	2131.338379	Filtered	1808.776733	3594.992676	2974.944336	4402.462891	Filtered
Filtered	Filtered	Filtered	Filtered	Filtered	Filtered	Filtered	Filtered	Filtered	Filtered	Filtered	Filtered
Filtered	Filtered	Filtered	2423.266357	Filtered	Filtered	Filtered	Filtered	Filtered	Filtered	Filtered	Filtered
2578.466309	Filtered	Filtered	2459.648438	Filtered	3447.898438	Filtered	5345.538086	Filtered	2489.335205	Filtered	Filtered
Filtered	Filtered	Filtered	Filtered	Filtered	Filtered	Filtered	Filtered	Filtered	Filtered	Filtered	Filtered
Filtered	Filtered	Filtered	Filtered	Filtered	Filtered	Filtered	Filtered	Filtered	Filtered	Filtered	Filtered
4045.185303	4782.259766	2575.273193	3528.449219	Filtered	2073.244629	6107.817871	2691.372559	Filtered	Filtered	Filtered	Filtered
Filtered	Filtered	Filtered	Filtered	Filtered	Filtered	Filtered	Filtered	Filtered	Filtered	Filtered	Filtered
Filtered	Filtered	3766.350586	Filtered	Filtered	Filtered	Filtered	Filtered	Filtered	Filtered	4479.569824	Filtered
Filtered	1304.008179	Filtered	Filtered	Filtered	3702.001709	3556.232666	1964.549561	Filtered	5120.639648	Filtered	Filtered
Filtered	4324.006348	Filtered	Filtered	Filtered	Filtered	3743.659912	5712.953125	4444.773438	5543.866211	Filtered	Filtered
4645.994141	2492.56543	5221.895996	2594.1521	2632.659912	2759.893311	Filtered	3848.078613	3421.565186	2977.236816	Filtered	Filtered
4226.029785	Filtered	3235.618408	3115.763184	2197.536133	2680.630859	2891.237793	3010.931641	4642.452637	3956.999268	2869.916016	Filtered
Filtered	Filtered	Filtered	Filtered	Filtered	Filtered	Filtered	Filtered	Filtered	Filtered	Filtered	Filtered
Filtered	Filtered	Filtered	Filtered	Filtered	Filtered	Filtered	Filtered	Filtered	Filtered	Filtered	Filtered
Filtered	2714.936279	1943.496216	3410.513184	3128.826172	3038.629639	2715.591797	3453.567139	3806.91748	4625.78125	2746.149414	Filtered
Filtered	Filtered	Filtered	Filtered	Filtered	Filtered	Filtered	Filtered	Filtered	5156.982422	Filtered	Filtered
Filtered	Filtered	Filtered	Filtered	Filtered	Filtered	Filtered	Filtered	Filtered	Filtered	Filtered	Filtered
Filtered	Filtered	Filtered	Filtered	Filtered	Filtered	Filtered	Filtered	Filtered	Filtered	Filtered	Filtered
1929.437866	4490.029785	2726.910889	3438.79126	Filtered	4963.659668	Filtered	Filtered	Filtered	3853.561523	Filtered	Filtered
Filtered	Filtered	Filtered	Filtered	3791.465332	1999.627441	Filtered	Filtered	Filtered	1880.141235	Filtered	Filtered
Filtered	Filtered	2522.29126	1704.978027	Filtered	3073.136719	Filtered	2334.162109	Filtered	Filtered	Filtered	Filtered
3718.343018	2883.820068	2778.058594	3316.668213	3906.213867	2415.937988	1865.918457	Filtered	5421.279297	4372.812988	4360.539551	Filtered
Filtered	Filtered	Filtered	Filtered	Filtered	Filtered	2714.083252	Filtered	Filtered	2817.839355	Filtered	Filtered
Filtered	3132.955811	Filtered	1640.805298	2824.02002	1923.621094	Filtered	Filtered	1854.594727	Filtered	Filtered	Filtered
Filtered	Filtered	Filtered	Filtered	Filtered	Filtered	Filtered	Filtered	Filtered	Filtered	Filtered	Filtered
Filtered	Filtered	Filtered	Filtered	Filtered	Filtered	Filtered	Filtered	Filtered	1499.390259	Filtered	Filtered
Filtered	Filtered	Filtered	Filtered	Filtered	Filtered	Filtered	Filtered	Filtered	3114.796143	Filtered	Filtered
Filtered	1899.926514	Filtered	Filtered	Filtered	Filtered	Filtered	Filtered	Filtered	Filtered	Filtered	Filtered
Filtered	Filtered	Filtered	Filtered	Filtered	Filtered	Filtered	Filtered	Filtered	Filtered	Filtered	Filtered
Filtered	Filtered	Filtered	Filtered	Filtered	Filtered	Filtered	Filtered	Filtered	Filtered	Filtered	Filtered
Filtered	Filtered	Filtered	Filtered	Filtered	2363.711914	Filtered	Filtered	3665.347412	3383.072266	Filtered	Filtered
Filtered	Filtered	Filtered	Filtered	Filtered	Filtered	Filtered	Filtered	Filtered	Filtered	Filtered	Filtered
Filtered	Filtered	Filtered	Filtered	Filtered	Filtered	Filtered	Filtered	Filtered	Filtered	Filtered	Filtered
Filtered	Filtered	Filtered	Filtered	Filtered	Filtered	Filtered	Filtered	Filtered	Filtered	Filtered	Filtered
Filtered	Filtered	1641.736816	Filtered	4409.11084	1669.766602	Filtered	Filtered	Filtered	2420.679932	Filtered	Filtered
Filtered	Filtered	1589.072144	Filtered	Filtered	Filtered	Filtered	Filtered	1313.547485	Filtered	1692.526855	Filtered
Filtered	Filtered	Filtered	Filtered	Filtered	3158.391357	Filtered	Filtered	Filtered	3682.812012	Filtered	Filtered
Filtered	Filtered	Filtered	Filtered	Filtered	2719.526855	Filtered	Filtered	Filtered	3891.939941	Filtered	Filtered
Filtered	3536.661865	Filtered	963.5360107	Filtered	2394.559082	Filtered	1429.698853	2342.131592	Filtered	Filtered	Filtered
Filtered	Filtered	Filtered	Filtered	Filtered	Filtered	Filtered	Filtered	Filtered	Filtered	Filtered	Filtered
2490.574463	Filtered	1928.532593	Filtered	2368.110596	Filtered	2120.568604	Filtered	Filtered	3069.206299	Filtered	Filtered
Filtered	Filtered	Filtered	Filtered	Filtered	1212.231201	Filtered	Filtered	Filtered	Filtered	Filtered	Filtered
1011.852844	1598.833984	1459.053711	1168.277466	Filtered	2302.107666	1092.973877	4551.196289	4097.311523	3034.660889	1917.005737	Filtered
Filtered	Filtered	Filtered	Filtered	Filtered	Filtered	Filtered	Filtered	Filtered	Filtered	Filtered	Filtered
Filtered	Filtered	Filtered	Filtered	Filtered	Filtered	Filtered	Filtered	Filtered	Filtered	Filtered	Filtered
Filtered	Filtered	Filtered	Filtered	Filtered	3900.651123	Filtered	Filtered	Filtered	Filtered	Filtered	Filtered
Filtered	Filtered	Filtered	Filtered	Filtered	Filtered	Filtered	Filtered	Filtered	Filtered	Filtered	Filtered
Filtered	Filtered	Filtered	Filtered	Filtered	Filtered	Filtered	Filtered	Filtered	Filtered	Filtered	Filtered
Filtered	Filtered	Filtered	Filtered	Filtered	982.3848877	Filtered	Filtered	Filtered	968.8303223	Filtered	Filtered
Filtered	Filtered	Filtered	Filtered	Filtered	Filtered	Filtered	Filtered	Filtered	1080.555786	Filtered	Filtered

PCOS_25	PCOS_26	PCOS_27	PCOS_28	PCOS_29	PCOS_30	PCOS_31	QC_1	QC_2	QC_3	QC_4	QC_5
149524144	69536904	44625320	137363984	167805872	604668544	625673088	141673312	143199680	140887728	143688544	151764400
116142776	90956240	100478160	120882256	101595656	71456360	72213968	99618880	110826768	107576072	109154816	109077584
23163880	36520696	33929080	35553184	26261330	41592604	42987848	33132448	32297484	31790158	33924936	32624992
22304070	25694194	30922434	30353888	26303346	28498350	23930962	24233574	25002092	24653986	24373022	24086088
19847106	21834908	20317026	20110762	18597972	16168880	16129290	18430964	18658192	17836514	18478638	18621704
20056954	22035614	24124630	18823600	15783002	20091560	20323668	18884232	19976236	20371928	19654712	19511572
13185784	18868332	19579556	19096976	14368749	15994223	16868552	16305015	15041153	16663391	16734246	16021649
17524330	16625767	18758374	16343339	11206572	14416187	14396765	15941228	15194847	14562280	16457430	14816670
9993106	16392268	13153182	14414325	11696703	17125598	16812144	14184894	15407808	14484075	14981629	14571749
11047396	12126214	13532409	12882092	11215063	15248631	14845520	11806758	13330967	13160353	13440972	12882254
8154158	11825528	9114613	10438915	8559543	14099314	14428773	11199375	12370381	12117238	10514533	11566588
23853270	11319431	16552691	9345817	7474098	1709837.625	2062540.125	10782494	9528816	12479872	8856276	8046245
10500780	9957865	11220314	10945904	7729731.5	11244164	11737030	9598838	9288938	9780173	9819449	9489096
5857682.5	11249456	8879835	8647659	5870689.5	11860286	12149897	9709372	9751119	9668527	9724906	9452546
7427277	13059224	10093655	6183650	8410110	10408331	9015850	8636411	8755965	8560150	8308894.5	9047496
9362700	11553023	11857901	9793601	9420467	15035467	12746703	7095880	8031893.5	8573486	7952804	7801081.5
7828610.5	8983780	9478389	9549813	7309610	6950143.5	4971862.5	7745856.5	8932266	8062132	8893248	8414668
5219016	8514006	9717597	9503138	7957111.5	10777650	11249449	8397114	8233142	8557806	9066631	8551340
4926237.5	9809635	7993573	7834723	5196179	11378025	12549443	7473649.5	8987458	8805255	8540087	8357833
90490.33594	13397870	7531763.5	70714.375	85730.07813	54533324	245844.875	23102060	20745822	58329.63281	21534270	66603.88281
6966820.5	9861131	8103126	6019064.5	6305680	8578277	8629585	7568667	7671452.5	7184727	7665145.5	7115318.5
5329049.5	6711881.5	7453729.5	7649502	6172423	12043726	12212268	7376476.5	7381910	7510836.5	7381156	7369109.5
6234567.5	10871124	8790168	6006268.5	6456983.5	9043176	5687054	7012496.5	8195004.5	7443174	7926088	7172152
10439632	6902344	8435513	5333461.5	4873960.5	6130447	12362385	5072671	5926625	5441892.5	6451198	5654054.5
3897888.5	8330949.5	6671702	6922569	6103386.5	5369473	5280671.5	6159319	6604347	6240685	6514573.5	6535470.5
5345716	7005642	5748416	6954360.5	4911461	8176768.5	8245907.5	6758098	6336040	6416009.5	6576210	6471516.5
4541166	5994095	6818175.5	6016038	4638970	8367652	8338263	6357959.5	6033520.5	6089278	6162310	5965803.5
4456281.5	6280005.5	6384684.5	7010219	4996360	4204560.5	4208336.5	5833046	6135063	5977801.5	5205331.5	6375309.5
5032971.5	6089444	11434688	5559010	4770121	7350703.5	7215054.5	5597218.5	5294990	5439540	5279549	5535891.5
3488951.75	3150972.5	4700486	8021629	5973123.5	4370109	4353611	4972521.5	4893519	4984832.5	5285002.5	4929482
7113710.5	6740902	4974303	4649543	2836594.5	3348082.25	4164544.75	5108517	4549995.5	4996057	5208525.5	4667832
4428370.5	5536422.5	6720041	5807847	4169704.5	5408411	5705968	5258122.5	4731483.5	5246283.5	5340200	5374428.5
4001875.25	4941137	4939451.5	4690679.5	3269947	5471442.5	5712423	4218032	4686285	4469414.5	4426469	4644013
3807701.75	5606003.5	3981834.25	4011647.75	3224309	6030652	6064950	4418103.5	4581533	4369283	4563183	4621193.5
1786841.125	2236526.75	3632469	6649839.5	5691880.5	4714118	4377094	4937427.5	5521986	5043740.5	4602504	4941635.5
5901367.5	2210220	1292413.625	4685038.5	7150440.5	27396822	26396946	3696613	4674422	4605249.5	2872797.5	5271435
2816562.5	2943242.75	3429755.5	4188625	4391097	4138731.5	4558064	3419884.5	2997470	3316249.5	3612323.75	3522814.75
3573165	5027175.5	3810738.25	4768231.5	3162672.5	4118866.5	4177208	3512980.25	4634268	3581414.5	3758485	3865333
2577836.75	3517960.5	2855169	3857168.5	2346379.25	4190946.75	3457199	3584505.5	3688319.75	3531898	3794625.75	3761690
Filtered	1655.765381	3551775.5	12655.99121	9991.525391	Filtered	Filtered	3100.067871	10424.71777	10136874	220710.6719	14808145
4435468	5232401.5	5935292.5	5812012.5	Filtered	6593950.5	6683129	2308.171143	3865136.25	3977099.25	1771624.25	2104816.5
3333124	3977844.5	3062600.5	3222909	1954527.625	3523175.25	2421344	3351468.75	3588188.5	3579969	3523392.5	3372933.75
3676079.25	3332889.5	4503960	3974581.5	3105234	2792877.25	3202619.25	3260942	3477681.25	3483387.75	3515829.5	3327464.75
950684.625	4562774.5	1342339.875	935811.3125	2911547.25	1057266.75	1056770	4398478.5	4336615	3929386.5	3247063.5	4256584
2059827.5	3622991	2581527	3381738.5	2528993.5	3227180.5	3304011.5	3015359	3120331.75	3159911.5	2916385	3092016.25
1980640.375	2972390	2980239	3508103	2915072.5	3645646.5	3776963.75	2660919.25	2347659.75	2530012	2559763.25	2280333.75
1825752.875	2833863.75	2917014.5	3650986.75	3286103	3995757.5	4186968.5	2668066.75	2764175.25	2674492.25	2585641.75	2625064
3331455.5	1150782.25	2039464.75	1277088	2589603.5	2553728	2687033.5	3519291.75	3853569.5	3737858	3939796	3177330.25
1844870.75	3130071.25	2274865.75	2284866.5	1598867.375	1946964.375	1654239.375	2769067.75	3003359	2642753.5	2533572.75	2574638.5
1192093.375	3285212.25	2146324	2828760.25	1625941.375	3861665.5	4084254.75	2471453.75	2424623	2364450.75	2409465.5	2418818.25
2221339.5	3252753.75	3110880.25	2560352.25	2223387.75	3227975.75	2113303	2620354.25	2788488.75	2570895.5	2759964.5	2680937.5
1786728.125	3163198.75	2945852.75	2823617.5	2276513.5	3638373.75	1269259.5	2958374.25	2795398.75	2856024.5	2476584.5	2937455.5
1982260.625	2828734.5	2424686.75	3122793.5	2219776.75	2883766.25	2820989.25	2232928.75	2345531.5	2256445	2400201.5	2192995.75
4293792	1585756.625	1406340.875	1363068	5614787.5	2342263	2421350.5	2526436.25	2438055.25	2431532.5	2396421	2225169.5
1594030.25	2361527.75	1768175.125	2358551.75	2414153.25	2531014	2548375.25	2376365.5	2495113.5	2421506	2026286.625	2476479.5
2228409	2106342.5	3217130.25	2005626.875	1985288.375	2503106	2479106	2250701.25	1954400	2082054.125	2295752.5	2261936.5
1579661.875	2164804.25	2335240.25	2274497.25	1667649.875	2306379.5	1532847.5	2079473.25	2163850.5	2216067	2273636	2146669
1400789.125	2339639.5	1959084.875	1935458.625	1449255.75	2565254.5	2804585	2058207.375	2036523	2080883.625	2117628.75	2003726
878051.25	1970558.75	1592107.125	2042051	2161376	2468699.25	2552959.75	1875010.125	2041806.125	2067993	1945876.875	2089630.75
1091974.75	1822248.125	1472118.75	2286178.25	1709638.75	2340049.5	2399113.25	2033931	2063640	2096020.25	2112518	2028111.375
2604994.25	3076114.25	2430456	1860905.25	1796396.375	1038837.438	Filtered	1768775.375	2269995.25	2210479.25	2378801.75	2148106.25
3489334.5	1007204.75	1111635.25	857044	2856789	2868872.5	2944885	2090217.5	2205246.5	2208456.25	2104928	2198924
1586452	1806768.125	1766685	2180554.5	1786825.75	2099264.75	2015092.25	1908491.5	1948494.5	1932221.125	1949902.25	1943625.875
3992307.75	498511.625	538448.5625	1002695.625	1267944.125	3220025.5	3255350.25	2187495	2047263.375	2189218	2173202.75	2064392.75
Filtered	Filtered	1876706.625	Filtered	2759150.5	Filtered	1661575.375	Filtered	1706077.375	Filtered	Filtered	2218862.25
1363683.875	2191896.75	2731891.75	1531701.75	1657837.375	199320.4219	214539.3125	2268744	1150847.875	1395832.75	1929658.875	1863350.125
1019862.25	2586419.75	1724208.5	1832805	1413198	2359258.5	2421916.25	1859863.25	1787388.875	1373164.75	1899450.125	1882296.625
1186114.25	1440118	1555922.5	2349993.25	1657843.5	1387653	1458816.875	1785427.5	1806673.375	1766028.625	1782351.375	1829048.25
4210727	2079662.375	2988420.5	2525633	1934211	591478.75	577640.75	704224.9375	2131739	2163830.25	2262189.25	2166283.75
1325226.5	1503077.125	1706854.875	1551183.75	1374331	1165823.625	1301893.5	1626029.375	1920564.75	1929778.625	2018206.25	1919485.375
2608341.75	1859362.875	2328024.75	1792363.75	1481015.625	1666683	1857754.5	1670620.375	1543477.75	1477374.375	1454654	1479895.5
1396014.125	1819913.125	1321531.25	918895.875	969513.5	852623.1875	782936.875	2022538.25	2447659	2543006.5	1129500.75	2550225.75
1053306.125	1364298.875	1483477.375	1614154.125	1524608.875	1949181.25	1711539.75	1632939.25	1710806.75	1694881.875	1598493.375	1652477.5

1579635.25	2253546.5	1493104	1167114.375	559336.375	1784454.375	1895825	1525892.875	1494175.75	1512357.125	1465549.375	1476520
2070369	869115.8125	571427.875	597783.4375	4275944.5	3882965.5	4044417.25	2089579.75	2286838.5	2232686	2343951.25	2247460
1300515	1597181.125	1819587.375	1623098.75	1165472.5	2081697.75	2168406.5	1461963.625	1449329	1555632.625	1531674.125	1530734.625
1915723	1191313.875	2408915	1718265.875	1907733.5	2050934	2123249.5	1373346	1400249.625	1404129.75	1551805.75	1446464.25
1114905	1370438.125	1282024.875	1822809.5	1328859.875	1211238.25	1256392.375	1556217.25	1371171.25	1448335.75	1469907.875	1395555.25
Filtered	871699.875	659140.5625	1708569.75	Filtered	Filtered	Filtered	1587073.875	1729156.5	1776697.625	1499268.625	1914098.125
1187059	1335699.375	1312747.25	1620133.75	1318073.625	1499693.625	1461553.25	1406888.125	1502029.5	1423721.5	1476897.125	1458804.375
910692.375	868088	1415480.25	2107254.5	1575156.125	1287722.25	1174410.75	1271489.25	1390522.125	1484294.375	1396544.875	1417741.5
1829427.125	2137845.5	2494077.75	2081125.375	504427.375	2173072.75	1896402.625	1629861.75	131936.8125	1712560.125	1782281.75	1664065.75
1191935	1593521.375	1274202.75	1077124.875	984261.75	1206188.875	1267090.25	1225742.5	1298893.875	1321174.75	1302751.5	1237869.75
1425913.875	1811021.625	1473937	963667.3125	844381.5	1784662.5	1877349.75	1358652.25	1418076.125	1331773.375	1296170.875	1345032.625
1249158.375	829783.75	514496.5313	678084.3125	5166993.5	3244138	3414657.25	1272470.625	1176861.75	1313978.875	1214767.5	1188266.625
1360058.125	1434079.25	1504354.75	1229578.625	973308.5	1684866.875	1710144.625	1182501.625	1244475.25	1235919.25	1275012.75	1218041.75
860419.375	853652.625	942909.8125	1411548.375	1420637.125	1065971	1073310.875	1182858.75	1193825.5	1149226.75	1155562.375	1129910.25
1013739.688	1431843.25	1543605.125	1232388.625	802812.3125	1540537.375	1520377.5	1120353.75	1237021.5	1245478	1165862.75	1259678.875
1257780.375	1479781.25	1689716.875	1302812.25	1102447	1050735.125	1118012	1122303.875	1295100.375	1232292.875	1211251.875	1232685
1122517.125	1343056.25	1645602.875	1096978.625	967646.5625	1071407.75	1107253.125	1137235.75	1098017.75	1104314.25	1117967.75	1102494.375
935173.375	1451750.125	1356249	1231182.875	931727.5	877150.4375	845500.125	916629.625	1212628.375	1261521.75	1239768.125	1226069.25
9808154	177671.5781	1173792	942875.875	690279.125	264894.3125	232860.8594	1609879.5	580407.875	536731.4375	205477.3438	366425.3438
977996.25	731413.9375	958037.3125	1397644	1197893.75	120993.2422	Filtered	1042660.438	1278383.25	1204846.25	1323460.625	1252249.375
460480.4375	1354484.5	874298.3125	1532582	273487.2813	63614.53125	64311.75391	Filtered	1600200.875	144545.4375	2994195.25	3232951.75
1117998.75	1030830	1345653.875	1219828.25	906528.375	1342518.125	1360347.75	984984	1022913.313	960024.4375	992278.125	987805.5
687760	182229.0625	93559.07031	123354.8828	670771.75	255504.2031	266229.125	244886.3594	254532.25	245298.0625	200097.9844	242051.3906
485822.125	740395.875	678149.5	778246.25	977819.0625	850157.5625	915965.5	1088372.625	1048309.125	1026224.063	1031198.625	1065282.25
819376	1260841.375	1066370.875	1188522.5	711536.25	926008.25	928855.1875	1008393.188	1055602.75	990361.1875	1037745.688	960655.25
633032.25	1056306	828102.25	1117235.875	781215.375	1226517.375	1272581	928417.6875	942193.0625	915302.0625	937551.4375	909758.375
1105785.125	582831.375	229732.7344	871717.25	1044575.813	5290902.5	6277373	1023276.625	1276731.125	1259397	936609.4375	862541.0625
1522567.5	532498.3125	682774.75	591211.5625	408008.2188	1245260.125	1392887	858901.6875	833304	951213.4375	503782.7813	811576.625
861004.25	812407.1875	869694.5625	957716.8125	786337.5625	926759.125	976220.75	882308.75	867274.5625	880989.6875	850354.9375	865547.6875
1204283	430681.5313	415966.0313	472153.4688	3366374.25	1332148.375	1380272.375	780226.1875	827970.3125	790922.4375	806659.375	832296.0625
595587	718149.1875	753571.25	900189.5	814390.8125	624790.625	610336.9375	692733.375	725714.3125	666030.875	681422.8125	719582.5
606879.0625	505288.8125	603396.625	833568.9375	967911.9375	537093.0625	562657	814970.5	794765.5625	804082.0625	764260.875	798762.25
Filtered	Filtered	Filtered	Filtered	Filtered	Filtered	Filtered	Filtered	858216.75	864733.8125	Filtered	1090942
580326.4375	301803.2188	260770.8125	315092.0313	477609.9688	223739.6406	118862.4922	426643.0313	213954.9375	343033.2188	349134.2813	325027.7813
804803.25	642935.5625	705730.6875	802178.5	532913.125	1017760.313	921027.875	866929.9375	624833.4375	591991	786351.4375	662830.4375
652419.625	506351.2813	698783.3125	933798.375	884132.125	229390.9531	442099.0938	675499	747484.6875	768256.125	780524.875	768714.625
533123.0625	746046.3125	632466.5	594874.125	509993.9688	831158.5	875968	631663	598248	645905.125	641866.375	652427.5
453633.125	112330.2031	55345.44531	80535.91406	433213.875	129007.4922	132945.4219	122643.6328	119290.3828	119981.8516	117668.6875	121811.5391
400910.5	669836.6875	560363.1875	640257.3125	402530.8125	701413.875	723090.0625	684129.8125	680729.625	656187.6875	630233.625	623255.75
Filtered	547234.625	696222.6875	636538.8125	Filtered	654667.25	Filtered	478940.0938	561268.375	626227.625	523405.1875	504103.7188
379850.5313	588566.375	560745.3125	482840.7188	368586.2188	703413.375	692553.3125	676683.375	607134.5	528465.75	587355.75	559451
641392.75	604243.1875	616902.5	620937.875	460726.5938	498352.9063	697847	615525.1875	584201	568578.8125	564382.3125	594360.5
573622.375	570312.9375	728161.875	693583.5	433710.9063	555889	597160.8125	578093.1875	591709.1875	591248.125	594765.0625	555713.6875
756940.5625	198455.2813	253256.9063	197015.125	1646742.125	1487477.625	1621240.125	499396.4688	491535.5	476876	465243.8125	457306.25
1271798	71265.16406	1025377	680680.6875	533109.75	1044827	1114464.5	855023.5625	772003.5	786461.75	773215.5	714465.0625
514882.25	674181.125	674764	613099.8125	378934.3438	543525.625	536218.8125	537561.625	544092.75	522001.8438	543445.5	533389.9375
1303108.25	146838.7813	166837.6563	204839.4688	2458926.25	2066741.125	2249963.75	545398.125	528346.0625	433460.7188	539978.75	457566.6875
1151553.875	299458.6563	163856.0469	211195.6406	1957541.375	215118.9375	201526.3438	526058.9375	630965.5625	558629.25	538322.25	594240.5
470776.4063	573872.8125	223642.6406	607289.0625	395138.0625	540539.125	537225.625	495573.4063	567317.625	541383.625	563616.25	541454.4375
1479825.375	172342.0938	219031.5938	139860.3281	1374382.125	1415405.5	1369932.75	550616.4375	500989.625	549828.375	514776.9688	489951.2188
428779.1563	402554.5313	442396.7188	662686.25	484418.125	217433.5938	211622.5313	486723.0938	480284.2813	498523.9688	506314	502543.125
658211.375	721606.8125	781682.3125	727014.3125	358566.0313	557427.25	607423.3125	440334.9688	509275.3125	405532.0625	415844.8438	415346.1563
362819.3125	485951.6875	472567.6875	375071.8438	35223.40625	624702.5	719530.125	708362.0625	685833.75	361213.7188	456964.6875	393673.6875
523840.1875	470076.5938	432320.3438	535174.75	204963.6563	572111.625	597915.375	480636.7813	479992.6563	464778.7813	454495.5313	468598.6875
3189930.5	24150.05664	1489673.125	1335317.25	32695.20508	20249.25391	19686	43459.60547	50545.29297	591061.8125	627546.8125	663981.375
Filtered	742506.375	Filtered	301552.6875	480709.9688	692520.4375	439016.6563	411455.3438	574387.3125	588540.75	413042.3438	555048.75
1796197.25	228281.3438	423116.8438	343923.4063	318075.0625	182341.7031	78602.17969	306230.9688	334321.0313	367365.875	334143.7813	339339.4688
308415.1563	382470.2813	406082.0313	478243.4375	391337.9063	567647.8125	504852.1563	441896.3438	445718.8438	433496.9688	473637.6563	452762.2813
424273.125	451309.9375	488751.7813	413911.75	384115.4063	456777.3438	490516.375	402155.3125	420433.1875	430037.75	346833.375	464898.25
851412.375	488224.9375	472926.2188	645131.25	296718.5938	2663.046875	5121.708496	9206.021484	12309.40234	407991.1563	406410.7188	403922.5938
602626.1875	343321.6563	446748.75	384130.875	476078.625	402481.4375	402362.875	325165.4375	320967	329750.625	332090.0625	317168.1563
450237.5938	271282.625	264242.6563	422466.5313	239775.5156	301847.7813	325115.8125	352132.8438	469719.1875	436608.625	437471.7813	404549.25
226524.6875	170786.4063	305197.7813	214973.3125	260363.3594	4442859.5	206443.2969	275507.5313	280384.0938	250081.2813	264761.2188	159150.8438
423811.2813	378864.5313	329688.875	479648.7813	329790.9375	405145.4063	425770.6875	412335.3438	407405.6875	401775	404859.4375	406519.9063
Filtered	Filtered	16326.51855	179748.4219	Filtered	Filtered	Filtered	3411.956787	Filtered	Filtered	Filtered	Filtered
675730.25	280518.125	521520.6875	364392.3125	378580.4375	254928.4531	259893.625	348539.0313	345478.9688	354661.375	331890.25	343084.6563
292156.25	372513.0625	405910.25	405849.0938	296657.2188	405800.5938	440801.625	378532	394436.2188	381471.5625	361432.0313	390525.875
540495.4375	204792.4844	359496.2813	203825.8594	213149.4844	56753.46875	23981.82422	495545.375	452572.5	488990.4688	496083.375	327806.9375
224241.1875	121221.9688	165339.875	279925.6563	542608.1875	620071.875	624309.8125	372596.4063	411921.9375	388371.1563	407105.375	399199
436069.3125	446481.6563	427764.9063	292142.1563	262417.25	347697.375	334324.625	355515.2188	351080.3125	348017.9688	367870.2188	364354.8125
255880.8125	184819.5781	364054.4375	164823.4688	248676.9219	386532.8125	241374.3594	232563.4063	338103.4375	321444.7188	258940.0625	265834.2188
121938.0078	382057.0938	254678.3906	336685.6563	393366.5625	527944.875	535345.1875	292921.0625	367559.9375	368114.2188	289651.9375	371461.0313
476518.375	298660.875	474107.75	381210.8125	294356.75	218901.2031	224516.4219	325726	322695.2188	304624.2813	307306.7813	314706.0938
Filtered	465043.8125	2487.5896	351884.25	Filtered	Filtered	Filtered	Filtered	Filtered	Filtered	Filtered	Filtered
324791.3438	375039	333686.9375	405534.75	263613.7813	284735.5	290383.3125	313887.2813	315976.75	329814.5938	310948.25	313893.3125

476417.6563	236928.1406	497969.0938	131659.8281	132322.9375	148221.2344	151852.3281	207912.625	214471.8281	216395.4688	218627.1563	224588.2344
Filtered	594744.3125	498377.4688	Filtered	236359.0781	Filtered	Filtered	97544.75	352805.5	306300.9063	381773.3438	354586.9375
174413.7969	336201.75	237146.875	311734.4375	320493.3125	321540.6875	315087.5938	318405.0938	310172.625	305563	316979.8438	303877.3125
429429.5313	126192.5	133486.4531	154881.0781	253236.3125	373702.2188	393139.9375	364707.1875	378582.6875	373222.375	326012.3438	401260.4688
255153.3281	68578.72656	137931.4219	580493.375	730285.4375	24105.9668	27102.14453	242456.0313	223037.7031	250765.375	255544.7344	253554.1094
89765.96875	30957.52344	5975.699219	132881.1563	178335.0313	3960972.25	4343973	264271.625	124341.7813	178207.0781	123299.875	225823.6094
802334.9375	143442.4219	95127.65625	124161.0781	620196	107056.9375	672822.5	365247	323613.7813	369621.7813	391651.9063	375975.7188
Filtered	Filtered	Filtered	20992.50391	Filtered	Filtered	Filtered	24936.08594	Filtered	Filtered	Filtered	5713.92334
Filtered	Filtered	147053.1719	Filtered	102873.5547	Filtered	Filtered	641494.5625	Filtered	621308.875	62359.35156	Filtered
269761.5313	438192.625	272926	298594.9375	189139.9375	270455.7188	266648	266654.3125	290071.6875	278770.8125	279223.8438	279337.9063
212169.8438	245384.7656	446987.875	360236.8125	302316.9688	239765	246038.2031	249428.2656	250094.5156	255704.2969	263754.2813	256176.2031
344890.3125	238017.7188	254924.6563	97552.67188	109880.8438	174754.1406	162485.6719	264634.375	291185.1563	292368.4063	264517.9063	283745.4688
215325.5	348022.9688	399104.9688	204106.375	190141.7969	228549.6563	236249.2344	272215.4375	292257.5625	266015.6563	281772.0625	250766.5156
Filtered	266781.8125	236398.6563	344840.7813	Filtered	Filtered	Filtered	129167.1641	276850	183310.7344	Filtered	131054.4609
302495.5313	180633.2969	269230.5938	193069.4844	217498.0469	151455.9531	156799.7656	159019.6563	147919.0313	149225.9844	154806.7188	154231.1875
414614.625	143194.7188	93054.27344	124213.5938	629785.875	72193.03125	354845.9688	366450.0313	324678.6875	370473.625	391134.3125	372632.8125
125722.5391	254940.9063	270731.25	219829.75	199960.3125	284647.9375	278644	244145.3438	252333.2656	256174.2813	252553.5	243937.6875
131361.5	67410.25	155650.9375	331914.2813	449104.4375	17888.94727	18224.49023	202545.9219	220878.0313	204564.5938	202955.375	215040.5156
104655.375	213368.9688	173386.7813	289951.9063	106152.7969	353438.125	383181.9688	278540.375	247468.2813	251050.5156	262953.0313	218008.4063
259564.4063	312248.3438	351500.8125	239897.2188	178575.4063	204475.25	218382.7344	225277.0938	224472.9531	220765.6094	227208.4063	241188.7656
149068.6875	222745.2813	197036.9063	133202.0156	188311.4219	183933.0625	199251.1563	311894.125	302283.3125	319826.5625	162794.9375	308041.0313
354565.125	83676.82813	45218.07031	81191.78125	934802.0625	1041718.625	999208.4375	231711.375	220215.7188	232339.0313	248975.9063	253647.5
354408.1875	331732.5625	358770.3438	217079.4844	200788.5625	165708.1406	106827.8984	156828.3438	254728.3594	150526.9688	299207.5	277068.5313
187385.875	257734.0313	236930.7188	167010.6719	148241.8438	329199.0313	328006.2188	204311	202677.125	205943.4531	194358.6563	192530.0938
245919.3594	198427.7969	215138.0625	169262.1094	356271.625	427605.875	491221.25	278443.3438	299407.3125	318448.7188	276148.6563	281777.75
Filtered	237442.8594	5976.833008	182124.2188	Filtered	Filtered	Filtered	142562.8594	159379.6406	Filtered	Filtered	136576.8594
334966.875	177377.25	588763.875	321645.1563	225968.3125	86178.59375	72623.96875	207086.1563	259133.0313	185142.4844	193322.4219	213665.4531
70375.46094	77579.10938	145995.5313	451488.5	298399.375	195036.4531	200754.9063	235282.5313	271266.1563	251874.4219	251803.6094	237779.2969
237577.0938	204801.75	239923.5469	257271.2813	191878	227284.3906	235972.7344	242321.3281	236454.7813	237619.7969	238955.9688	234309.4063
6033.125488	396296.625	303805.75	252832.0781	263448.4063	229748.1875	241820.7656	246058	248788.2031	282600.1875	258140.25	298900.4688
151268.8438	131579.1875	178669.0938	195036.1719	234150.0781	229183.2969	218445.2813	260102.4531	262173.4375	257807.6875	262196.5625	249526.0625
558051.5	67441.0625	55859.66406	76527.8125	400592.2188	653967.75	647478	223809.1875	241908.3906	237693.4063	252057.7813	260277.7656
170514.4375	216664.4531	175633.3906	257157	246539.7656	190866.3281	194589.7656	177794.1563	195347.875	199348.0938	194282.0469	205377.375
185605.4063	171026.0938	154176.0156	165382.2813	179009.7813	140907.5156	154814.875	273500.6563	205484.7969	224188.5781	202262.125	218082.2813
351550.0625	158880.6406	99458.39844	124840.8516	667834.3125	227237.5625	258402.9219	523004.7813	470983.0313	222692.0781	193753.375	226226.6094
140239.8438	186454.6563	156503.0625	242467.2969	190735.1563	193898.9063	184924.6875	185170.6563	182345.1563	188210.625	181956.3438	197443.875
115802.5938	215710.875	158376.1406	236499.8594	167780.4219	134938.0625	141160.8125	204270.8125	213969.9688	196172.2188	200831.875	210048.4063
Filtered	Filtered	Filtered	Filtered	Filtered	Filtered	Filtered	11943.7998	15621.61816	14508.11523	18571.22656	13513.0918
503529.5313	87273.57813	43209.26953	51943.32813	709358.625	478257.75	510796.5938	188901.4688	195841.3281	207879.7344	204843.3438	201609.9375
146409	204658.8281	164112.1094	186606.125	133481.0156	237210.4375	245392.625	191801.7031	194583.7656	197208.2344	199220.7188	191731.4063
247876.6406	236476.9063	186163.4063	225390.0781	138145.375	177160.8594	179733.25	175120.2344	172911.4375	176453.9531	184760.7656	180560.4531
Filtered	Filtered	Filtered	Filtered	Filtered	Filtered	Filtered	Filtered	Filtered	3788.877441	6195.739258	5164.061035
167643.4844	187583.7031	235830.8906	202917.2813	161040.4375	265921.1875	174176.0156	183230.875	185234.1406	179587.9844	170947.875	178920.0313
Filtered	Filtered	Filtered	Filtered	Filtered	Filtered	Filtered	Filtered	Filtered	Filtered	Filtered	Filtered
117609.625	152299.4688	185284.5156	189762.5781	147788.9063	88138.89063	92782.32813	111336.0938	111050.8125	115666.75	115888.0703	101194.4141
197430.5	1805455.25	194914.0625	160626.3906	170412.9688	209608.8125	219457.5313	118932.3359	Filtered	109524.4453	117879.0938	114493.8672
181507.5313	202074.375	164875.4688	161073.6406	101342.2188	132706.2656	121223.1172	143005.9375	160285.75	165591	169988.4219	167283.7813
123535.1641	172662.2188	174133.8281	223493.5781	196051.5625	144634.8125	143738.75	162453.1719	157808.3594	172843.3594	169535.3438	166494.5156
236862.3125	108410.4453	158599.3438	208441.1719	171952.4688	101025.3125	104793.8281	158971.625	157010.3125	153197.3281	156073.6719	162402.1563
125982.3828	34649.4375	181174.25	288145.6875	364122.5938	Filtered	Filtered	122535.7422	110751.9063	124467.7109	128113.2656	126245.5156
146446.3594	146628.6406	165546.1094	211654.5938	162361.9375	162660.1875	165388.8906	164304.8125	170429.6094	170721.0625	166738.5313	176034.9688
302736.6875	66623.66406	40860.99609	59334.30859	520374.4375	219606.7813	237408.2188	172016.4219	183888.875	184855.7969	188037.4063	187865.7656
105271.1406	26307.65039	46708.05469	210739	305067.125	34458.4375	35569.85547	69898.07813	115917.7891	119491.6641	117517.4297	114973.4922
Filtered	Filtered	Filtered	Filtered	Filtered	Filtered	Filtered	Filtered	Filtered	Filtered	Filtered	Filtered
159637.8438	151988.4844	133361.3281	242864.5781	165967	95936.6875	115876.6953	169629.375	169547.9375	177076.0156	160603.0313	177706.0938
127566.3906	147366.9219	126884.6875	132660.2813	113254.0703	145535.6875	184426.9219	138007.6094	170978.3906	169083.8594	151457.3125	159778.6719
Filtered	Filtered	Filtered	Filtered	Filtered	Filtered	Filtered	Filtered	Filtered	Filtered	58835.33594	Filtered
425799.2813	15009.33789	Filtered	22630.07227	596109.0625	378182.4688	442428.8125	134568.4063	51981.12109	136659.875	153283.9688	53895.75781
38256.64063	40407.91406	32812.74219	39605.125	23248.85547	13328.95215	14666.22559	25716.04883	18190.91602	19713.50781	22162.12891	20653.33789
431431.125	35970.01953	39824.94531	42310.15625	521825.8125	359051.375	378161.5625	135696.2031	128420.1875	142938.9219	141059.375	58725.39453
1.02E+05	9.67E+04	1.08E+05	1.06E+05	7.40E+04	2.74E+04	1.93E+04	2.20E+05	1.73E+05	2.40E+05	2.42E+05	2.31E+05
140334.2813	119401.3359	142789.0313	164322.9531	149398.1719	177015.7969	180313.1563	134317.1563	131726.4063	138764.6719	138169.9375	136536.0781
121699.9375	83933.5	66548.21094	94572.03906	180184.25	99824.74219	293831.1563	156987.6406	158932.0469	146006.6563	295978.5	99246.39063
92190.04688	112504.5859	200688.5313	78666.375	10736.99609	44371.07422	9537.949219	52763.45313	56291.66406	55120.75391	47710.51953	71047.14844
119252.6875	140113.2813	265902.4688	107198.5313	82726.89063	17184.25195	160266.5313	175498.1563	84830.10156	97054.91406	179141.9531	192561.9375
99755.20313	54748.71094	77772.53125	123300.1953	107280.5156	149776.0625	155082.1406	132712.375	140310.4531	132194.3438	135732.5625	136044.9219
133377.8438	128535.0234	158376.4375	95877.51563	73700.5	140529.125	148947.7813	144228.7031	141409.6094	136041.9219	133517.4688	138272.6719
88787.70313	44523.5	68057.69531	204464.75	209203.7188	506346.8125	489700.8438	156433.5	148220.4375	147141.1875	149701.5625	145279.8281
53989.34766	21576.63672	59663.60547	29095	23890.0332	16222.06836	11987.83887	258935.7188	19347.20508	246959.4531	22877.40234	262707.5313
259719.0156	Filtered	Filtered	Filtered	Filtered	952753.5625	106192.125	2593.658936	Filtered	Filtered	Filtered	Filtered
101626.0625	153543.0156	93516.65625	156572.4375	90036.45313	121572.4063	130044.3281	132077.75	122514.75	124631.2813	122037.7734	126413.2656
273349.25	35207.52734	34509.14063	65362.47656	177965.4375	219911.2813	222093.0625	134363.9219	135112.7969	143323.7344	152762.9688	144531.875
74682.70313	136387.4063	112668.4453	138932.8906	93614.6875	135961.2031	194837.0625	158621.4219	105278.2266	107622.1953	105748.6563	124596.8125
150511.8281	165328.625	235058.8594	98698.28125	82873.97656	Filtered	Filtered	14906.10547	105607.2578	107000.6328	243092.2813	21960.06055
730666.1875	Filtered	12111.00391	Filtered	Filtered	Filtered	Filtered	Filtered	Filtered	3299.974854	Filtered	16892.4082

141116.4063	131470.1094	109108.25	115763.6172	113782.9688	155966.2031	157303.7813	138255.5	134915.3125	127735.1563	129648.9453	118379.2891
127177.3125	115750.9297	125226.5234	72967.45313	159545.6563	94650.46094	71412.10938	116812.1641	119474.5781	134636.3906	114598.3594	113665.3359
414939.375	50758.27734	42173.43359	53218.98438	194444.125	425357.0625	418598.6875	133433.7969	133186.8906	155939.5781	148627.5938	156023.6094
61140.20703	101510.0391	85308.41406	141498.0938	105838.2031	126917.5703	127895.0469	122935.0156	119486.8203	122225.6016	125876.6328	124435.875
Filtered	99097.92969	131682.1094	Filtered	Filtered	45973.15234	Filtered	134581.0938	144642.0625	127363.0391	100334.3828	148278.5313
Filtered	Filtered	Filtered	Filtered	Filtered	Filtered	Filtered	Filtered	Filtered	Filtered	Filtered	Filtered
35067.34375	88093.3125	69063.71094	137878.25	114870.9609	7684.770508	10820.30469	115398.2813	118060.5547	113427.0703	113189.5078	113674.3906
351028.0313	42748.19922	179095.8438	107529.3984	67024.32813	32138.02734	39779.44531	68650.79688	121078.0156	98236.61719	60595.625	72796.85938
109191.1563	114482.5625	93785.75	117140.3672	102971.125	114435.6953	106927.4688	97027.1875	92203.02344	117629.2578	122288.1797	100087.8203
Filtered	Filtered	110692	Filtered	Filtered	Filtered	Filtered	Filtered	5308.581055	Filtered	Filtered	Filtered
139595.2344	62373.04297	37722.77344	136232.9219	163020.7344	498580.0313	557634.9375	119822.2109	144699.2031	137649.1406	141641.25	129061.9297
Filtered	Filtered	Filtered	Filtered	Filtered	Filtered	Filtered	Filtered	Filtered	Filtered	Filtered	Filtered
Filtered	Filtered	Filtered	Filtered	Filtered	Filtered	Filtered	Filtered	Filtered	Filtered	Filtered	Filtered
89581.24219	166781.2344	81892.59375	96665.51563	97482.65625	54197.41016	52860.35938	92961.48438	79852.625	96567.89844	92197.9375	95518.88281
Filtered	Filtered	Filtered	Filtered	Filtered	Filtered	Filtered	Filtered	49714.11719	Filtered	Filtered	Filtered
Filtered	Filtered	Filtered	Filtered	Filtered	60819.13281	Filtered	6056.908691	4558.552246	Filtered	Filtered	Filtered
125745.2266	84379.5625	76247.71875	84173.10938	72637.35938	98518.16406	129025.1719	91069.82031	83372.58594	88889.83594	96980.98438	99673.6875
147026.6094	27571.02734	18680.71484	30412.55859	258621.1875	183265.4219	204405.0781	69916.20313	129891.0078	88582.23438	120649.25	118385.1016
Filtered	26549.76953	Filtered	Filtered	162729.1875	Filtered	55457.38672	141909.6406	114748.9063	Filtered	70312.74219	161402.4844
67921.53906	101314.5781	85543.96094	129451.3125	99019.11719	90346.08594	94342.14063	91303.53125	93374.83594	83371.20313	100052.7891	98154.13281
47094.96875	50617.38672	66779.11719	154449	88259.51563	73973.29688	76872.73438	95898.75	95134.24219	92926.77344	88890.82813	93489.34375
155388.1094	31476.86523	29273.12695	34900.69141	230085.7969	90278.73438	84146.33594	85094.75781	80406.60156	81524.85938	78679.24219	73601.3125
37638.61719	30467.58594	38270.25391	38479.03906	26409.83984	51474.98047	Filtered	29128.94531	28353.83984	31827.30273	27895.60156	57320.51563
87193.41406	127514.7656	260718.6406	61831.75391	53291.20703	53711.16406	52382.10938	86955.84375	84821.35938	89556.02344	88701.375	88219.47656
87894.79688	124815.1484	85738.13281	44167.52344	66007.17188	68473.20313	76795.67969	83345.82813	73530.33594	84547.5625	87980.78125	86783.16406
100099.7578	109137.25	135194.625	138961.9531	32223.85547	6581.470215	Filtered	63610.41016	69276.29688	64300.9375	70649.0625	69876.5625
69225.92188	76286.28125	43314.10938	76689	58046.23828	116065.1719	118528.7031	91533.8125	85133.83594	85573.71875	87240.94531	86576.90625
75199.58594	94879.36719	44285.30859	118267.2734	94744.3125	96842.71094	102652.5703	51406.72266	47994.49609	45874.28125	146415.1406	87425.67188
65718	64280.79297	53950.57813	130114.2031	157911.7188	62989.21094	28063.64453	64825.53906	61844.93359	67084.35938	62030.45703	64884.51172
60797.69141	57628.22266	52929.20313	85806.15625	77282.75	88467.15625	102291.2578	75187.95313	82964.23438	82885.5	74491.49219	79213.89844
Filtered	Filtered	Filtered	Filtered	Filtered	Filtered	Filtered	Filtered	Filtered	7149.356934	Filtered	Filtered
168596.4063	55863.20703	113576.0234	75432.95313	88170.79688	45563.15625	42635.57813	57802.73047	63838.13281	65505.42578	82172.77344	84082.76563
Filtered	Filtered	Filtered	Filtered	Filtered	Filtered	Filtered	Filtered	Filtered	Filtered	Filtered	Filtered
59557.8125	19694.19531	61159.76563	118951.5078	162177.3594	61546.69531	56101.98828	53289.87891	54526.55469	56180.27734	53715.12109	55361.44141
314689.2188	311543.375	396553	177478.6094	40236.88672	66225.78125	69629.67188	Filtered	32328.69922	59346.85938	40255.74609	38333.19531
75725.17969	73783.36719	58509.10547	44423.09766	67932.16406	131336.6406	119122.6953	86176.26563	86755.25	81190.34375	131468.0938	77594.1875
62663.67969	51179.32422	49920.51563	93486.625	70894.1875	41258.63281	41605.21094	81783.64063	80465.30469	83604.45313	87214.6875	79391.23438
Filtered	Filtered	Filtered	Filtered	Filtered	Filtered	Filtered	Filtered	Filtered	Filtered	Filtered	Filtered
71107.05469	24978.93164	23112.96484	25069.89648	173547.1875	263220.2188	267844.25	74829.67969	76130.52344	75599.75781	73869.94531	95738.625
Filtered	Filtered	Filtered	Filtered	Filtered	Filtered	Filtered	Filtered	Filtered	Filtered	Filtered	Filtered
42460.1875	58053.34375	59760.80469	65840.9375	84563.57813	55174.73828	55088.43359	69102.44531	65337.57031	72930.39063	71238.10938	70241.96875
41334.42578	42006.47656	48848.26953	59060.97656	60111.81641	45587	47891.75	65812.4375	72039.57031	67590.29688	65544.27344	66006.41406
156631.1563	24608.01563	14638.00391	23421.64844	308681.25	195355.4531	278075.0938	66685.75781	73019.30469	69638.60156	67453.42188	74568.3125
Filtered	80793.96875	Filtered	Filtered	Filtered	Filtered	236418.4219	Filtered	72413.71094	Filtered	46298.80469	Filtered
30225.26953	19634.93945	125866.3672	25532.05273	27147.56641	27609.3418	26961.57031	61380.13672	70303.64844	58315.19531	72004.40625	68667.94531
Filtered	Filtered	Filtered	Filtered	Filtered	Filtered	Filtered	2554.208008	1239.121216	2572.228027	Filtered	Filtered
123230.1875	68588.78906	100570.4688	40509.58203	64380.36719	63540.37891	66021.79688	40298.80078	37395.4375	36375.53906	40308.30469	55623.58984
41131.56641	47057.39063	67830.45313	95968.78906	70847.0625	51941.89844	53341.21875	61960.01172	57297.0625	64093.52734	62272.59375	66652.34375
Filtered	70544.57031	260496.8438	91173.03906	103979.875	6988.066406	6843.003906	87464.60156	5304.242188	91322.03906	100323.1094	102460.7031
162044.6406	23603.65039	24151.64648	30576.50391	140303.9531	160333.7188	172402.125	38242.85938	72498.60156	69995.85156	72340.49219	63046.12109
29479.60742	16327.71289	94583.86719	97650.48438	135058.0313	23381.87305	23501.56055	52125.50781	51278.82422	57760.39063	48509.31641	49784.24609
105931.3516	129490.5156	150140.7656	87123.28906	100906.9844	Filtered	Filtered	17118.37305	54742.93359	58982.83984	68572.23438	71685.33594
68522.57031	63094.31641	52607.91406	66739.92188	50635.24219	52625.46875	54916.62109	64651.28125	61200.44531	62107.82422	66039.60938	62161.53906
Filtered	Filtered	76078.10938	Filtered	62938.32813	Filtered	35833.32031	Filtered	Filtered	Filtered	48423.36719	Filtered
74281.72656	28118.80273	44539.13672	32102.19141	58912.84375	62727.43359	69066.98438	81667.57813	84066.94531	75968.25781	61915.80469	82629.89844
47669.05078	33791.92188	38355.11328	70347.75781	60600.36328	70412.60156	79460.34375	60425.35547	52747.52344	53790.57031	74785.0625	53603.43359
Filtered	48413.96484	Filtered	Filtered	Filtered	Filtered	Filtered	Filtered	Filtered	Filtered	Filtered	Filtered
139996.3438	73892.59375	117781.3594	78379.89063	103903.0234	24508.78906	27168.23047	22162.99609	70039.10938	69907.85938	80740.42188	64322.78125
Filtered	Filtered	Filtered	Filtered	Filtered	Filtered	Filtered	Filtered	Filtered	Filtered	Filtered	Filtered
81807.73438	18110.15234	17126.66211	29413.33789	115456.4609	Filtered	Filtered	69214.45313	64475.35156	73923.92188	71294.92969	68504.47656
60440.46094	67574.89844	76760.41406	67631.54688	54111.66406	85547.98438	74570.8125	47357.35156	48724.68359	48033.58594	74531.72656	52752.35547
52517.07031	40163.62109	20913.27734	62653.52344	39465.19141	67043.35938	72762.21875	65242.93359	64548.69531	59682.97656	63464.64844	63453.48828
97874.38281	30769.25586	Filtered	16584.54492	219463.7344	215181.8125	228770.1875	47143.48047	56127.45313	54310.45313	54069.58594	49035.72266
Filtered	Filtered	Filtered	Filtered	Filtered	Filtered	Filtered	Filtered	Filtered	Filtered	Filtered	Filtered
Filtered	Filtered	Filtered	Filtered	Filtered	Filtered	Filtered	52969.8125	62036.91016	55086.37109	46699.69922	53924.76953
64386.13672	48477.85547	57983.39063	67003.125	54065.61719	59631.43359	47253.70703	51440.86328	49225.9375	49936.20313	63209.46484	55885.41797
17405.95313	46072.99219	47533.26172	90862.85938	44682.33984	87922.42188	91469.94531	59824.99609	63223.08203	57927.09766	58497.04297	57182.28906
40483.38281	79905.26563	77628.77344	64845.48047	35059.58984	46105.03125	43902.61328	55057.46094	55823.16406	48654.27344	50867.16797	52051.01563
56355.62109	47489.76563	82537.77344	38553.29297	74732.21875	34307.44531	31361.84375	38377.45313	38024.04688	75140.48438	83884.35156	84926.875
36047.06641	Filtered	Filtered	Filtered	120749.0625	84110.42188	86341.23438	35175.98047	Filtered	28372.29883	27418.81055	32896.6875
81603.72656	45605.82813	65825.49219	81313.82813	48932.8125	34521.35938	35597.34375	52740.36719	51271.67969	52587.1875	51635.42188	52394.08984
44041.125	37255.48828	57647.28906	51244.42969	44869.69141	54230.45313	56823.29297	48578.58594	53315.45313	51929.09766	49555.82031	53022.06641
30281.81641	45710.74609	39998.26953	58863.27734	49136.43359	46330.01563	55934.41016	50114.9375	51779.46875	50945.46875	50517.24609	54459.92188
70260.23438	57621.96094	55169.22266	104361.8281	39642.97656	37641.02734	39980.55469	43712.82422	45150.87109	45343.72656	46777.67188	49050.52734
68332.71094	31310.89258	21446.17383	37301.27344	107263.0938	126027.9844	119742.8828	36390.76563	48053.69531	39603.5	42638.74609	43012.25781

45507.47266	79793.13281	53192.55469	40077.32813	53513.40625	34500.64844	83561.89844	50508.23047	42200.64844	55511.64453	58670.39063	53056.48828
105588.1875	11782.43945	11570.83496	15421.51563	220374.6563	118782.9063	115346.5	46263.99609	45349.08984	42019.65234	49788.82422	47072.39844
23809.23438	17139.08789	39849.67969	66399.91406	86650.26563	Filtered	Filtered	34875.17188	34858.54297	36537.82813	37004.99609	37201.42578
Filtered	Filtered	Filtered	Filtered	Filtered	Filtered	Filtered	Filtered	Filtered	Filtered	83814.125	Filtered
36491.57031	48525.87109	41163.48047	45540.16406	36032.03516	53697.63672	52050.64844	50474.86328	51137.34766	50552.96094	48985.86328	45644.53125
26231.07422	128223.4375	47159.69141	44304.89063	35496.00781	28998.42773	31548.0293	40451.03906	42321.96094	42443.23047	42049.17969	39327.40234
32991.27734	43382.52734	42905.73047	61515.81641	55176.91016	48260.35547	43697.125	46688.01563	49555.77344	47989.16797	52145.21484	49261.40625
89118.54688	3428.49292	42749.37109	36600.66406	28486.83203	Filtered	Filtered	47204.14063	37428.25	48243.46094	49647.64844	49014.82813
Filtered	Filtered	Filtered	Filtered	Filtered	Filtered	Filtered	9764.133789	12115.62988	6425.373047	Filtered	Filtered
111418.8438	24490.74219	Filtered	23034.89063	28443.55469	108401.6875	110826.0859	98749.78906	83131.39063	84310.27344	78494.5625	78094.29688
113241.7734	8753.150391	12351.91992	18288.19531	172580.75	170829.3281	159798.8594	44289.15234	46762.64844	46224.91406	45515.66797	44867.3125
62169.59766	60411.76953	65324.67969	60801.52734	54165.51953	36768.78125	36871.87891	42544.16016	46619.76953	45832.85156	50299.76172	50868.92969
40605.30469	6717.350098	51905.49609	79949.04688	26112.6543	38824.32813	39938.09766	44744.41016	42967.29688	42799.47656	41665.27344	40289.72266
Filtered	Filtered	Filtered	45032.4375	Filtered	Filtered	55884.57031	Filtered	Filtered	Filtered	94144.76563	Filtered
26831	67715.6875	44895.82031	50614.71484	37105.60938	45671.98047	46211.1875	48326.64844	50545.48438	49713.51172	48477.39453	46996.74219
Filtered	25111.10352	15049.18262	37507.39844	17961.26367	33261.36719	36212.18359	48093.32813	52023.13281	42483.33984	49381.03516	51947.52734
44521.07422	49096.44922	46660.53125	43247.17188	39135.86719	34077.51953	39370.70313	41994.96094	44141.9375	63051.05469	37466.75781	40100.29297
19866.71094	12768.95996	18169.08203	17251.78516	26191.59766	14440.95605	14142.89844	17920.29297	17030.20508	16505.49219	15421.07422	17147.30469
36182.25781	42523.01953	38415.23438	44323.80469	36896.28125	47673.68359	50679.69922	52649.60156	49789.72656	51298.44922	53989.42969	47849.96875
36401.40625	41259.82813	33522.67578	45713.48047	37751.8125	30969.09375	Filtered	55679.78906	38510.10156	39846.49609	55051.82813	38435.29297
32146.27148	49140.27344	Filtered	36418.14453	84124.21875	71359.38281	201199.0313	90774.65625	83967.46875	22110.01953	13148.85156	22379.41797
Filtered	1334.822998	Filtered	120940.2031	Filtered	Filtered	Filtered	Filtered	Filtered	Filtered	Filtered	34044.84375
95789.03125	22056.45703	14292.55273	29335.83984	142998.8281	67384.3125	48645.4375	30220.42773	35436.71875	56788.06641	61612.22266	58795.92578
56282.57813	48485.22656	55121.26563	33499.09375	31795.89453	38163.21484	37486.11328	40662.13672	43753.47266	42579.84375	41302.07031	41104.35938
Filtered	2903.471191	Filtered	3367.720215	5532.312012	2924.850586	3988.499512	6016.709961	6158.18457	4723.362793	Filtered	Filtered
Filtered	Filtered	Filtered	Filtered	Filtered	Filtered	Filtered	Filtered	Filtered	Filtered	Filtered	Filtered
Filtered	133337.6094	74479.50781	Filtered	Filtered	98318.00781	120324.7969	Filtered	Filtered	Filtered	Filtered	Filtered
81395.14844	16479.23242	17382.39063	29120.16797	44031.22266	44328.41406	47337.19922	68980.32813	64207.57813	73737.70313	70470.46875	68362.08594
Filtered	13525.22852	Filtered	Filtered	Filtered	Filtered	Filtered	41520.69531	Filtered	23310.85938	Filtered	Filtered
Filtered	19668.67578	Filtered	Filtered	Filtered	Filtered	Filtered	Filtered	Filtered	Filtered	30912.91992	Filtered
Filtered	Filtered	Filtered	Filtered	Filtered	Filtered	Filtered	Filtered	Filtered	Filtered	Filtered	Filtered
35562.88281	83594.79688	57948.82813	62433.31641	26480.53711	59096.88281	40747.96484	37595.28516	36916.92578	30021.5625	44259.42188	38935.12891
46521.10547	24187.23633	22220.3418	22384.69141	70297.89844	85151.46875	88061.10938	40875.55078	37701.125	38181.15625	42942.4375	38185.84766
30738.04883	29859.50391	28781.74414	37804.26563	32707.01758	47339.6875	46874.98828	42529.49609	41475.98047	39407.4375	40470.71484	38922.82031
Filtered	35664.5	22906.11133	49469.32813	37574.35938	36118.70703	37209.03516	43781.68359	39877.98438	43473.57422	47633.36328	39153.25
Filtered	93100.39063	Filtered	2203.029785	Filtered	Filtered	Filtered	Filtered	26161.73633	55216.0625	33959.44531	1254.854492
40597.00391	30907.05859	25203.12891	37103.86719	20399.39258	44355.67578	45022.5	30950.77148	38910.08984	40381.42969	43072.15625	45906.14844
27299.1543	16841.12695	38930.42578	26446.14844	24888.01172	34510.33203	37693.18359	30078.14844	42921.41016	42618.34766	39028.40625	39278.75781
48560.50781	86173.4375	55787.21094	78749.02344	24679.64453	31559.84375	32280.95508	42397.00781	39261.94531	46304.1875	44248.30469	43307.54688
55763.48438	39148.58203	20516.67188	46949.49609	34217.80078	88750.17969	95690.39844	38839.19531	31797.60938	34733.16406	35651.62109	38105.12891
22569.72266	38285.30859	19960.66992	28152.32031	21282.02734	28317.68945	30413.10156	41186.89063	33967.68359	41240.25	44436.35938	43502.23047
22072.82813	33750.18359	33266.30859	46340.03906	34374.39844	26563.67383	36585.02734	37980.48047	35272.44531	35574.6875	35710.42578	38081.62109
23650.79297	34592.66797	44630.53906	35177.27344	41967.48047	22654.40234	22895.08008	36669.03906	36497.66406	38687.21484	36036.75391	37990.27344
27883.25977	32603.95898	36986.79297	24944.16211	31267.98633	61396.28516	74599.10156	34002.66406	36471.03516	34942.60547	28644.70508	34320.11719
Filtered	Filtered	Filtered	Filtered	Filtered	Filtered	Filtered	Filtered	Filtered	Filtered	Filtered	Filtered
Filtered	Filtered	Filtered	21096.12109	51337.63672	42349.23828	38377.69141	Filtered	46318.29688	48224.27344	40637.33984	Filtered
24918.68359	11201.38477	19620.49805	22256.49023	17052.18555	44707.09375	44870.59766	13540.5293	18392.15039	17120.56641	19005.65039	18268.99805
Filtered	Filtered	31834.45313	Filtered	4280.541992	Filtered	Filtered	Filtered	2880.258789	2250.57373	1497.503174	Filtered
Filtered	Filtered	Filtered	Filtered	Filtered	Filtered	Filtered	Filtered	Filtered	Filtered	Filtered	Filtered
89692.46094	20819.17578	Filtered	13530.64453	44879.04688	118638.0625	75369.46094	57394.05859	50865.77344	51145.60156	53902.34766	27394.65039
35894.53906	40666.09766	33581.21875	43908.62109	23338.95508	57345.91406	54655.67188	31841.54883	31953.33203	32416.05859	29700.28711	41147.24219
26863.65625	22282.4375	39289.07422	42940.28906	39939.63281	27144.59961	29179.22656	39913.78516	35009.98828	37622.58594	35292.53125	38879.02734
42682.71094	59503.35547	44823.92969	59121.32422	39266.69922	49620.05078	32218.93359	35552.14844	37279.1875	20961.54688	36942.45703	88629.72656
10579.76758	11941.16895	13152.30859	35038.90234	28832.30078	13292.02539	15234.40137	37134.45313	32336.06445	30674.74609	36431.77344	31879.36133
59869.94531	19141.55078	67947.78906	19933.63281	22065.98633	Filtered	107514.1328	7647.204102	42067.99219	81387.03125	9044.80957	91422.61719
Filtered	Filtered	Filtered	Filtered	Filtered	Filtered	Filtered	Filtered	Filtered	44447.69531	Filtered	Filtered
Filtered	Filtered	Filtered	Filtered	Filtered	Filtered	Filtered	Filtered	Filtered	Filtered	23477.86523	Filtered
23526.22656	13919.74902	45270.49219	32686.45117	38954.9375	Filtered	Filtered	17348.01367	20407.66406	20736.41406	22177.31641	22864.70898
43910.16406	Filtered	Filtered	Filtered	58976.76563	28479.89844	34131.40234	Filtered	Filtered	Filtered	27698.16211	7569.083984
23665.45508	37183.41797	26556.45117	39996.56641	29379.93359	51106.35938	54514.27734	32451.42383	32050.60938	26896.66602	33802.23828	38050.30469
Filtered	9033.87207	10438.06934	44663.74609	62749.27344	9962.368164	9874.541992	23998.57813	24723.1543	23585.31445	19784.74023	23200.54883
23440.67188	29960.01953	31227.29297	23988.66602	52705.51953	31094.73047	29163.3125	4402.599121	2636.568359	23352.42773	30605.84766	24728.96484
34961.52344	68835.84375	34155.96094	37594.05469	43474.12891	17766.8125	20128.02734	28827.84375	28245.49609	24396.74219	24935.84961	34841.16797
Filtered	Filtered	Filtered	Filtered	Filtered	Filtered	Filtered	Filtered	Filtered	Filtered	Filtered	Filtered
16603.01563	6096.769531	Filtered	Filtered	16592.66406	3693.769043	3120.942871	4902.636719	4737.132813	5160.094727	Filtered	7425.803711
Filtered	1944.299438	1300.128418	Filtered	1435.901978	8633.332031	1840.193359	3298.038574	2342.817139	1369.250854	1506.086914	7157.575195
16453.97852	21717.49805	227973.4375	17043.43945	19126.00977	21784.08984	38717.60156	31603.99219	25696.43945	26141.50977	25662.32227	19552.06641
44571.75	26457.58008	28797.35742	27465.77148	32482.36133	26876.79688	31560.05859	32257.14453	33217.98047	33784.20703	34546.96875	33534.28125
Filtered	Filtered	Filtered	Filtered	Filtered	Filtered	Filtered	Filtered	Filtered	Filtered	Filtered	Filtered
Filtered	5588.489258	Filtered	7627.972656	6681.482422	51786.00391	54570.07031	6980.392578	6938.457031	44304.67578	50315.02344	52051.17969
Filtered	Filtered	Filtered	3197.742188	29406.55469	12494.98047	7283.599121	8631.893555	11255.14453	7840.249512	7856.408691	8251.211914
Filtered	Filtered	Filtered	62430.44141	Filtered	Filtered	Filtered	Filtered	Filtered	Filtered	Filtered	Filtered
Filtered	Filtered	Filtered	22420.22852	45842.03906	43169.31641	48823.72656	Filtered	Filtered	Filtered	Filtered	Filtered
33213.73047	42233.82031	29251.42773	35723.98047	29372.4043	29854.88672	35373.16406	26983.34766	23128.92969	27040.51758	27739.84961	30206.08594
Filtered	Filtered	Filtered	Filtered	Filtered	Filtered	Filtered	Filtered	Filtered	Filtered	Filtered	Filtered

Filtered	8069.227539	7025.150391	15578.76367	35041.67578	18285.5625	20942.25195	23906.45703	24979.68555	24921.07617	26124.44922	24760.02734
34145.13281	24462.9375	47941.68359	64851.28125	44117.44531	13903.04883	15891.45508	24342.02344	25157.24219	23871.2207	20533.53516	19354.53125
Filtered	25138.35742	27057.36133	32990.48828	Filtered	Filtered	20777.82227	25647.68359	32688.25977	32771.39063	27662.20117	33024.55469
25432.90039	50300.82031	26161.98047	34724.60547	28359.28516	16019.91504	16381.46973	29530.07422	26233.81641	26914.4375	26753.19922	27897.17969
27342.20508	Filtered	Filtered	Filtered	46806.27344	Filtered	Filtered	12978.35742	8947.045898	12433.5166	13589.37012	8835.811523
25345.67773	26527.80859	25780.51953	33083.11719	28866.83008	30586.71875	36151.95313	29945.35742	26950.09766	26727.53711	26784.55859	22273.0957
78661.98438	12548.95117	Filtered	Filtered	Filtered	16772.05664	Filtered	2403.634277	Filtered	Filtered	47175.98438	2037.058838
28600.61328	15018.85352	23672.04297	23939.12891	35894.40625	42472.10547	43815.54297	31557.71875	29758.96094	20330.35547	32135.22266	29710.0293
Filtered	7947.831055	30127.97266	6769.313477	37818.68359	Filtered	Filtered	3278.565186	3407.375732	Filtered	35367.08203	34395.92578
38432.05078	Filtered	44546.42188	19875.47461	Filtered	Filtered	Filtered	4869.773926	Filtered	105213.5156	34834.57031	48250.41406
17222.8125	26514.41992	21413.77539	41045.94141	28603.90039	22467.63477	24043.36133	26294.36914	25361.39453	24960.72852	26756.11914	28394.5293
56125.02344	22164.95703	25151.60938	28862.94336	32109.14258	23528.70117	23928.16797	25099.51172	24039.07422	26357.9668	27414.76172	26530.9707
Filtered	24494.52734	29422.25977	20124.45703	25598.25781	24395.95313	24943.32813	42438.53125	21769.50195	21625.45703	22719.24609	24227.51758
Filtered	30391.48828	34458.55078	36395.65625	24343.79883	10962.16309	13520.31738	26641.32617	29677.7793	27284.2168	28449.8457	26815.04102
16409.68555	22300.58398	25069.92969	31184.26758	32051.6875	32234.62305	31180.20898	28219.26172	26447.52344	25695.58398	24540.64063	25034.41211
23607.58008	26907.68555	20569.27539	21327.66992	33840.65625	13244.74805	35454.53125	40455.95703	23546.33008	20783.50781	23010.46289	35950.76953
Filtered	30472.60938	22735.69336	25814.58398	24488.60742	Filtered	Filtered	28317.25781	29268.80859	28903.37695	26809.43945	22949.10742
Filtered	16974.77344	Filtered	Filtered	Filtered	Filtered	Filtered	5472.024902	6692.408691	Filtered	Filtered	7986.194336
65777.54688	6115.145996	15946.99023	18569.73047	20843.29297	Filtered	Filtered	12508.31445	12319.97461	10628.05371	14086.12793	12264.63672
Filtered	Filtered	Filtered	9357.536133	29113.74219	Filtered	Filtered	Filtered	Filtered	Filtered	Filtered	Filtered
Filtered	14951.67383	132153.625	37388.54688	Filtered	69470.3125	Filtered	14346.66797	18279.84375	17945.97266	15501.83496	17433.9082
Filtered	28124.38672	Filtered	4618.449707	Filtered	4563.305176	4726.911621	37240.24609	66899.14063	32744.54492	Filtered	Filtered
18641.99609	23218.95703	17007.60742	27324.9043	20893.63281	23113.10352	46051.16406	31388.32813	32630.62109	29449.76758	26718.23828	23009.97852
15133.18164	33969.46094	22964.76563	29903.50586	29238.48438	19773.87695	20268.94141	22753.36523	20929.50977	22982.49414	24903.33203	24442.59766
30455.64844	21163.06641	25525.49609	24423.37305	20056.79102	47731.13281	18790.39258	22334.54102	21584.89063	19360.44141	20310.55664	19523.58398
22147.11328	20170.74414	32762.07617	30232.86328	19880.20508	25655.21094	29717.85742	24218.64258	23644.24609	24969.35938	27474.36914	24761.35547
Filtered	110384.375	8029.373047	9136.362305	5013.672363	65888.30469	48007.99609	Filtered	Filtered	Filtered	29265.64258	18462.23828
59750.54688	Filtered	Filtered	Filtered	66577.42188	31932.35938	29425.53516	17611.38672	11827.78613	14730.66602	13714.72852	39882.79688
22419.92383	18558.07422	26064.19922	25872.61719	26578.73828	20478.20508	19999.31836	22539.60352	22901.08984	24066.38867	22631.12891	24537.64648
20647.12891	20026.92188	22464.5957	25977.54688	21149.45117	19087.11914	19563.16016	24528.52734	23538.06445	23468.98633	24496.55273	22440.25391
19823.54492	13817.74609	21370.14648	31758.5625	34593.625	31551.51367	31048.18945	23550.50195	23573.18555	24158.60938	24809.25	25188.23828
Filtered	Filtered	Filtered	Filtered	Filtered	Filtered	Filtered	Filtered	Filtered	Filtered	Filtered	Filtered
11234.08203	20361.875	15768.82129	24500.4375	17254.15234	17479.3125	18837.00391	26304.12695	25273.29297	26072.65039	16133.42383	21849.81836
Filtered	Filtered	Filtered	Filtered	Filtered	Filtered	Filtered	Filtered	Filtered	Filtered	Filtered	Filtered
34350.84766	13149.16309	15291.7373	24985.65039	22262.04297	16036.2041	16969.37695	21030.83789	21757.24023	21127.83984	21348.28516	20496.00195
15664.69238	18908.81055	14350.68066	27765.9707	30311.73438	19025.48633	15474.88867	20589.78906	21137.61328	21616.01953	23118.39844	22077.51563
15602.16895	24376.14844	20950.49609	30196.12891	21083.25977	31648.31836	28183.93945	13771.50586	21049.52734	22703.32031	24312.55469	15991.88477
8095.077148	16529.68945	9973.130859	31149.23438	46736.75	31829.75391	53590.875	24689.73828	28304.70703	25067.58594	24862.32031	26562.22266
Filtered	Filtered	Filtered	29516.95898	41313.01953	Filtered	Filtered	Filtered	Filtered	16922.6582	13210.66797	Filtered
29246.00391	17062.80273	26464.875	19699.05078	27386.72461	15653.5625	16141.91699	16508.47656	15260.57813	19268.31445	23112.625	18240.94922
Filtered	13496.49219	Filtered	11978.97363	10384.10449	8639.710938	18578.67969	6519.166016	13175.53418	9131.953125	8200.369141	7310.621094
45335.28516	14959.02832	24098.89453	16824.73438	16607.98633	10811.99902	10984.28613	30277.30664	17073.9043	15715.93164	29461.25586	24760.8418
18317.43555	19599.28516	19149.64453	24794.5293	22801.47461	24843.85938	24837.10352	22689.85156	22186.83789	21073.08398	21990.28906	20502.7793
65892.75781	9947.480469	5526.916016	6437.398926	87586.3125	25622.56641	8069.836914	23841.99414	27327.56055	25498.42383	26797.66211	39675.92578
Filtered	13319.81738	5645.658203	12486.59375	Filtered	Filtered	Filtered	10383.70801	11821.125	11021.17969	11477.49219	13988.34766
13449.33105	11129.20117	6717.175293	22833.54297	13452.1084	26453.49414	27113.60547	25141.98633	22736.24219	23774.08789	24101.29688	22130.76758
9354.65918	20594.72461	15138.57227	28869.29492	19948.80859	20976.84375	21005.00195	21045.96289	23591.29297	22617.81055	23059.13672	21742.64258
26633.16211	19732.24609	25900.10156	25970.84766	20546.56641	16867.52344	28577.50781	19534.96094	20793.10352	19561.78516	27188.12695	19707.53125
14962.82031	26027.70117	Filtered	17706.49805	26184.16406	25433.90039	20592.82422	30638.03516	27641.75586	27447.04297	21959.11328	27868.57617
Filtered	28880.9707	28082.05273	16397.25781	21234.13477	40551.35156	40532.66016	20239.57422	15122.24219	20750.36719	23129.36133	22900.25586
15402.3584	16415.04297	19257.55859	24937.75586	23619.56445	22254.19336	21702.48828	25068.10938	20013.72852	21379.49609	20343.33203	23674.51172
Filtered	36216.10547	28570.24805	28551.59766	14472.81445	20042.50977	20042.62695	18063.68359	Filtered	15354.39648	15944.71094	17828.76367
18018.91406	19886.14258	21416.38086	23685.43555	18366.22266	24580.44531	26573.28711	20414.87891	22654.51953	22109.2207	18960.23047	19552.90625
Filtered	Filtered	Filtered	Filtered	Filtered	Filtered	Filtered	Filtered	Filtered	Filtered	Filtered	Filtered
Filtered	6522.482422	2871.754395	12240.08301	221297.3125	11596.64746	10992.52539	7783.068359	8584.245117	7967.630859	6065.906738	6609.077637
28600.27539	17888.28125	19937.5	20851.07422	21915.9082	17987.22461	20767.62891	20064.04297	15611.8252	19830.61523	18664.375	8620.859375
Filtered	Filtered	Filtered	Filtered	Filtered	Filtered	Filtered	Filtered	Filtered	Filtered	Filtered	Filtered
Filtered	16793.19531	26129.64844	16246.83789	13571.71387	15870.37695	17032.26172	15705.76367	17073.49414	12804.42188	18332.81836	17549.07813
Filtered	39111.85547	5861.50293	4546.774414	28640.57227	3658.227051	48344.13672	24449.16211	17292.53125	24342.65625	23166.82813	38035.69922
23858.10547	22276.87891	24656.17188	30131.08008	21892.42383	10604.64355	10685.19922	14343.30957	17005.02734	15671.42871	14955.96484	16931.69141
Filtered	3870.60376	Filtered	Filtered	Filtered	Filtered	Filtered	Filtered	Filtered	Filtered	Filtered	Filtered
Filtered	22324.26953	24493.89063	21555.77148	19170.94922	14033.01855	20984.31055	20405.47461	19532.84961	19928.6875	19083.97852	19661.61719
26829.67383	Filtered	Filtered	Filtered	30326.91016	48518.39453	52341.77734	14513.27832	14872.81738	14498.89258	15985.38281	13287.26172
11994.59668	21968.17969	19286.30273	20110.44922	17733.2832	27033.99219	22913.57617	19982.0332	21863.67578	20804.51367	20058.5918	18507.17188
12215.56836	13103.83984	16277.84863	22034.64648	25359.58789	23265.4082	24228.87305	21908.86133	20352.15234	21913.46484	19841.40039	21048.19336
Filtered	Filtered	Filtered	Filtered	Filtered	Filtered	Filtered	Filtered	Filtered	Filtered	Filtered	Filtered
10054.47168	16510.52734	62627.22266	68994.85156	23206.75586	15782.56641	8572.691406	13634.31543	11195.83105	8586.172852	9376.541992	16495.07422
23549.75391	20735.26758	22801.27734	24166.47852	19415.58594	14905.42871	17867.94141	19488.21875	18722.97266	20482.25977	20520.16016	20892.67578
17954.33594	19961.5957	23877.88281	20744.15039	30449.94727	19722.8125	21532.08789	17672.15625	19068.12891	20443.89453	19352.0918	19990.74805
Filtered	Filtered	Filtered	Filtered	Filtered	23688.16211	28447.70703	14003.24805	Filtered	Filtered	Filtered	Filtered
26107.24219	Filtered	Filtered	Filtered	57010.57813	28373.24219	29707.73438	15291.95508	13932.76563	15452.62305	14118.73145	12771.44824
Filtered	19698.83398	10629.38281	33191.71484	19220.87891	22675.62891	24230.69141	17590.80664	18305.00977	18191.86914	18993.25781	18486.32227
58367.71094	Filtered	Filtered	Filtered	39196.38281	25761.35938	26967.83008	27597.60547	24129.27344	21390.37109	21789.26367	15650.32031
Filtered	5532.698242	Filtered	Filtered	Filtered	52511.35938	Filtered	Filtered	Filtered	Filtered	Filtered	Filtered
Filtered	Filtered	Filtered	Filtered	Filtered	Filtered	Filtered	19166.54297	15641.00977	20864.03711	Filtered	18039.63867

Filtered	16959.02344	13473.62109	25267.21875	13457.65625	13118.48535	14332.4707	22436.82031	21781.1582	20393.67969	19599.46484	20476.75586
10893.94336	13207.39941	16689.58984	28384.85938	23519.86133	13338.06445	17433.00195	18530.13672	21594.7793	16413.26758	16615.56055	15325.40039
20635.62891	16376.79395	14173.65527	17313.91406	15927.03906	14674.64746	14950.63184	15011.66016	18621.52539	16348.48438	15831.12402	19007.18359
Filtered	18397.73047	17833.30469	18157.32813	18813.87305	18009.16211	18056.75195	18623.90234	19350.24609	19117.98438	18661.16602	19421.14844
16791.75	20835.10352	22611.63672	25955.28906	18882.30859	15387.86914	17259.75	17265.94336	17806.95313	18034.36133	17182.64258	14506.50781
33459.8125	Filtered	Filtered	48907.78906	Filtered	Filtered	Filtered	27396.51172	15161.86523	22771.69922	17218.57813	15369.10645
21072.55859	11914.45996	25704.55273	17603.6875	14236.59375	9850.064453	11150.13574	16507.60547	17477.32617	17208.75781	18101.86133	16329.06445
10418.66895	11479.52539	18550.04883	14769.79004	19260.15039	7137.019531	Filtered	8880.082031	3558.35083	Filtered	10187.46484	11368.37598
Filtered	Filtered	Filtered	18994.70117	15593.52734	Filtered	Filtered	Filtered	Filtered	Filtered	Filtered	Filtered
39259.76563	9798.602539	20897.05078	16086.90039	18011.16211	7331.343262	7516.726074	17592.45313	15571.46289	16383.69238	22505.91016	18946.32813
24708.95313	22375.02344	18509.95703	17400.49023	19599.74219	14711.69922	15185.91895	20836.4707	22552.91602	20071.6875	21314.55469	17992.35742
Filtered	45729.22266	26060.80078	35493.70703	23020.88867	20001.90039	21800.80469	Filtered	Filtered	Filtered	Filtered	24586.9707
Filtered	97198.03906	39560.66406	2906.63623	Filtered	94180.85938	95716.75781	4901.797363	4129.058594	6124.125488	2787.590088	15305.00293
Filtered	4364.884766	2214.899658	7348.387695	7200.361328	7619.375977	7136.414551	26992.66602	26616.23633	31894.62891	26592.71875	27183.66797
32728.54688	10897.65039	11170.72363	14293.09473	18432.66992	13646.42773	18881.4043	25881.36914	16758.48633	16424.125	16818.92773	24107.30469
13574.51367	20820.71289	24001.7168	19633.01953	38884.85547	15078.96289	14914.375	14643.30957	26746.57422	16058.33789	15937.61523	15498.11914
22070.54883	25567.07813	13687.27832	20206.09375	14553.01563	25100.25	22038.37891	18346.32813	19065.65039	18630.88086	20123.35156	19413.5625
Filtered	25021.24609	27492.95898	12485.76953	13448.92676	17141.23242	18330.68359	12365.73145	26821.22852	15960.4043	14968.14648	19561.77539
Filtered	Filtered	Filtered	Filtered	30565.21289	Filtered	Filtered	14632.49219	15889.64355	16277.88477	14986.41016	13517.18652
13274.44824	8661.135742	14791.20313	19961.23828	24901.80664	17868.01953	18925.64648	18399.24219	16605.65625	16605.44336	17521.73633	16617.29883
18872.88672	17376.13477	20574.52734	20221.80664	20140.57031	16827.08203	19803.39844	17512.49609	15130.81055	15989.04004	20367.17773	19635.54102
Filtered	11259.8418	54404.72656	22936.60352	16691.12109	11459.99512	12025.8457	14768.9082	15064.99023	15227.30859	16178.65527	14341.78613
6424.583008	15758.52637	16297.48047	19474.93164	17386.51172	18536.76758	18528.86719	18062.0957	17186.76758	15690.51367	15452.6748	17746.20703
Filtered	Filtered	Filtered	Filtered	27772.61719	8891.889648	10542.2041	Filtered	7431.961914	Filtered	Filtered	Filtered
34000.72266	Filtered	Filtered	Filtered	49865.63672	36486.97266	36785.64453	16912.25	16219.35938	16013.30371	15546.49414	16893.55664
Filtered	10616.15332	Filtered	20373.25781	25330.11328	22158.85156	24141.15625	17963.34766	17676.79688	18299.57422	14645.40332	16081.83984
22201.49414	14466.86719	12384.56836	20086.57227	32025.87695	9657.866211	6697.0625	13668.23535	12528.25977	13460.91211	12818.83887	15070.2207
Filtered	Filtered	62613.84375	15512.46777	Filtered	Filtered	Filtered	Filtered	19651.80859	20096.8457	Filtered	Filtered
Filtered	15590.00879	15335.61035	16349.05078	17149.63867	22561.08984	21769.82227	18181.69141	17126.76953	16676.94336	16407.51758	17307.56836
Filtered	9294.949219	13705.2627	23649.79102	17770.34961	Filtered	Filtered	Filtered	14236.51758	17207.82617	15404.06348	15708.3584
Filtered	Filtered	Filtered	Filtered	Filtered	Filtered	Filtered	Filtered	Filtered	Filtered	Filtered	Filtered
29361.08984	9445.521484	5207.496094	7465.943359	57507.80469	47545.44922	45963.64063	23659.75977	18421.04883	17211.9082	21443.57617	22255.53516
Filtered	14852.46973	29642.46094	16098.69336	11874.78906	Filtered	Filtered	15219.76172	14779.18457	15254.49414	15246.94336	15981.79199
20781.58398	3829.20459	Filtered	15732.79297	40242.72656	29846.44336	30669.1875	5731.347168	6002.624023	5741.606934	5958.918945	6074.555664
51773.96875	24247.04102	9674.547852	16096.66016	7492.637207	9478.211914	11698.78516	12522.21582	17657.85938	16482.29883	12629.4375	12448.08105
19874.50977	12579.3125	27711.67969	14183.62598	14510.18164	13597.06543	14378.97461	14703.2793	11590.77734	16119.19238	15393.21973	16024.37305
Filtered	Filtered	Filtered	Filtered	2739.147949	Filtered	Filtered	Filtered	Filtered	Filtered	Filtered	Filtered
20763.91602	11859.1875	18693.43164	24343.36523	25024.49219	26106.82617	18284.12109	13908.08496	10075.52637	14468.77148	12236.15332	11828.5918
Filtered	16666.70703	9804.835938	Filtered	Filtered	14540.00781	20698.83984	14726.85156	17911.46289	14698.38672	13318.30273	15127.78809
13788.07813	16172.39063	14499.39453	13166.5791	13631.56445	13740.65332	19029.76172	15113.38184	16533.4082	16804.57227	17617.18945	16378.10059
Filtered	3130.52002	Filtered	Filtered	Filtered	Filtered	Filtered	3179.55835	1951.804932	3013.635742	Filtered	1490.997314
Filtered	9189.177734	219413.0469	16736.85156	12404.17578	7790.35791	6754.953125	13158.3457	13190.55371	12220.38867	9936.515625	12211.37988
36922.02734	5029.416992	Filtered	12560.41992	2836.277588	Filtered	Filtered	Filtered	1990.054932	2645.333008	3584.178711	1896.188965
Filtered	16816.6543	18684.92773	11756.09766	22437.74414	24785.76758	14543.22656	19608.42383	25988.28516	10614.06445	10749.75098	23200.42188
Filtered	Filtered	407930.1875	9657.823242	Filtered	3757.43042	4421.886719	5891.953125	4562.138672	3705.685547	3765.203857	3224.831055
Filtered	9559.102539	9460.609375	13067.97852	25694.19336	20324.99805	16455.71094	9467.152344	10415.69824	9850.154297	29006.82227	28057.26953
Filtered	Filtered	Filtered	Filtered	Filtered	Filtered	Filtered	Filtered	Filtered	Filtered	Filtered	Filtered
Filtered	3325.605957	Filtered	Filtered	Filtered	Filtered	Filtered	Filtered	Filtered	Filtered	Filtered	Filtered
12359.86523	13605.73828	16014.50488	21510.58203	18631.50781	13874.78711	12275.60547	13233.00781	13844.74121	14615.32031	14057.0498	13846.98633
13755.26953	9210.206055	22231.01758	15436.1543	9505.137695	10650.2793	10908.25293	15495.02637	11556.77832	12060.70117	13809.15527	10152.82422
Filtered	16398.12109	14212.16797	21778.25195	20304.47266	7467.793945	17122.22656	17057.39453	15475.53711	17396.11914	13352.93555	13703.14648
11786.19531	15830.62305	8656.59082	15204.87891	9161.6875	16066.36523	18247.98047	12296.42969	12997.88184	12152.21387	10911.73828	16519.29297
11538.7666	14779.4668	14058.89941	15355.92676	9851.701172	15071.00781	13753.26074	11175.49316	17588.4668	15158.38574	17616.25586	17423.31055
Filtered	24634.86133	Filtered	2714.264648	Filtered	1171.840332	3240.375732	Filtered	Filtered	Filtered	3644.666748	Filtered
15931.9668	21519.38672	21223.64844	15778.87598	14410.81836	13269.31152	12291.40332	15250.09961	13446.85059	13748.01367	12666.1748	12683.61328
43593.38672	12093.21289	19998.4043	20157.64648	11937.13672	9059.914063	9236.332031	13651.5498	12529.29297	12452.80469	12054.38965	11704.49023
Filtered	12553.01758	14764.97656	15963.66504	14521.02637	25359.45117	17497.99219	16976.19727	15918.32813	11294.55859	17537.4668	13579.84668
17233.26758	Filtered	Filtered	Filtered	30576.07031	21745.94922	14736.42676	11837.92871	11675.74805	11103.78027	10514.19922	12316.10156
15195.52344	12180.29688	16041.66992	16414.89258	16765.19727	13493.32813	13496.00586	15499.19434	13748.76855	15481.4502	15562.56445	14818.03516
9723.27832	Filtered	Filtered	Filtered	40014.89063	13112.93359	15541.60547	13300.14844	6744.098633	5617.79834	7142.189941	6810.602539
23090.02148	Filtered	Filtered	Filtered	10034.85645	11292.66406	38512.04297	11665.04199	15846.52734	14300.01855	12229.98828	14968.62598
8423.292969	19694.41211	14270.18555	14965.8252	12563.81836	8622.893555	8590.476563	12740.24023	12948.70801	11733.6709	12673.23535	12540.52344
12971.59082	10013.25098	15509.63281	22667	17083.25781	15909.22266	18023.57031	14646.30469	11246.93164	14641.92285	12985.32031	15212.62793
15533.53809	11842.57031	10338.31543	21707.36523	16281.1748	11505.94922	13092.42773	13570.84668	21436.15039	14952.06836	15336.76855	14160.85059
Filtered	13139.33398	14632.31348	13670.17188	14142.14355	17354.00977	15262.32715	19743.44922	12138.04688	13624.99121	17645.52734	17394.49414
Filtered	Filtered	Filtered	Filtered	29690.52148	21080.94336	25954.40625	Filtered	Filtered	Filtered	Filtered	Filtered
Filtered	Filtered	4098.791504	Filtered	12383.18457	Filtered	Filtered	Filtered	4415.34082	Filtered	Filtered	Filtered
Filtered	Filtered	Filtered	Filtered	Filtered	Filtered	Filtered	Filtered	Filtered	Filtered	Filtered	Filtered
Filtered	Filtered	Filtered	Filtered	16864.39258	2923.206787	Filtered	2177.524414	Filtered	Filtered	Filtered	Filtered
Filtered	4248.64209	3441.194336	Filtered	4394.248535	Filtered	Filtered	16079.99805	17751.9707	17208.60156	17773.99023	14921.81055
Filtered	Filtered	Filtered	Filtered	Filtered	Filtered	Filtered	Filtered	Filtered	Filtered	Filtered	Filtered
13420.06152	11630.47656	12280.05762	16931.04297	17961.25	14923.07422	14435.58594	13971.82715	13555.61426	13503.70508	14614.9043	14059.98828
Filtered	Filtered	Filtered	Filtered	Filtered	Filtered	Filtered	9781.441406	10122.73535	8810.557617	9393.225586	8928.262695
Filtered	8445.681641	11628.75098	14257.22754	14140.93945	12269.08105	13522.25	12271.42578	12112.34863	13292.75977	13485.86133	13539.26074
14592.72168	13942.76855	Filtered	Filtered	Filtered	Filtered	2551.979492	Filtered	14596.59668	18472.53516	Filtered	Filtered

Filtered	Filtered	Filtered	2479.200195	Filtered	4087.872314	8036.32373	3550.858398	Filtered	1742.47522	Filtered	2981.923828
Filtered	Filtered	Filtered	Filtered	Filtered	Filtered	Filtered	Filtered	Filtered	Filtered	Filtered	Filtered
Filtered	12544.99805	14908.15039	14986.77539	12944.21973	Filtered	20376.98242	14924.90039	Filtered	Filtered	Filtered	10435.55078
12445.29688	15353.83008	8875.661133	16106.87012	11688.2373	15884.47852	18531.01563	13750.49805	12499.16211	13803.13477	14251.36621	13697.16211
Filtered	93085.03906	Filtered	7185.027344	4653.333008	5430.016113	5559.301758	Filtered	Filtered	7163.518555	Filtered	8585.720703
Filtered	5938.041016	Filtered	4344.278809	33484.50391	30076.00781	29211.08398	15126.20117	13042.26172	13465.15625	12517.05957	13595.23242
11636.27148	10116.60352	11404.49219	14072.02344	15626.41699	16211.02051	18671.16406	14426.46777	9790.685547	10094.53613	12890.98047	18140.75195
Filtered	13212.4541	14154.89355	15494.08887	11370.83984	8762.115234	9815.587891	16848.96875	13992.35156	13403.19629	12308.60156	13634.61426
11388.99219	20874.05664	18120.60938	18265.00195	16688.33594	17885.8125	8437.454102	14869.72266	14784.34277	11792.97754	15163.5293	11995.55078
12134.6709	11386.56836	23456.50391	20133.37891	20081.67969	12630.30469	13674.5752	11215.00391	13436.69434	10087.66797	11083.7959	11451.73926
Filtered	13695.67773	13659.79688	12739.22461	17175.16016	19531.31055	21907.53711	9550.625977	4988.764648	3887.552734	2781.359375	5351.03418
Filtered	Filtered	Filtered	Filtered	Filtered	Filtered	Filtered	1.23E+04	1.05E+04	9.94E+03	9.14E+03	8.62E+03
Filtered	11057.69824	11358.19043	11982.43359	11931.61914	11189.51465	10918.8457	12851.68066	13001.41309	12752.71875	12420.66113	12410.86426
Filtered	Filtered	Filtered	Filtered	Filtered	Filtered	Filtered	Filtered	Filtered	Filtered	Filtered	Filtered
Filtered	14746.8457	13456.01563	13458.40723	11271.33789	11173.53613	13608.89941	12943.87207	13201.59766	13629.15332	11956.28906	13368.13086
7185.285645	14018.77246	14425.17969	Filtered	Filtered	7219.379883	Filtered	Filtered	14019.7998	14354.73242	Filtered	12602.95215
Filtered	Filtered	Filtered	Filtered	Filtered	Filtered	Filtered	Filtered	Filtered	19626.9668	15577.2334	Filtered
9653.085938	6893.236328	8238.777344	7409.887695	7228.430176	8475.640625	7154.055664	9454.488281	6557.19873	4712.135742	5026.850586	5410.875488
12679.26855	15520.5625	13246.34766	13458.53906	13258.40137	15703.66113	12187.65723	11854.9248	11004.10938	10563.59277	9961.158203	11765.48926
11839.83496	Filtered	Filtered	Filtered	25733.2793	Filtered	Filtered	Filtered	7002.500488	7176.583984	6717.418457	Filtered
Filtered	Filtered	Filtered	Filtered	Filtered	2007.684082	Filtered	7202.664063	Filtered	8240.00293	9200.575195	7966.079102
134082.2969	Filtered	Filtered	5139.210449	2808.670654	Filtered	3281.262695	5567.483887	2702.605957	Filtered	3996.166748	4318.44043
Filtered	Filtered	Filtered	Filtered	Filtered	Filtered	Filtered	Filtered	Filtered	Filtered	Filtered	Filtered
Filtered	4389.694824	11139.34863	11250.2207	7911.136719	12425.54102	12863.82324	8333.198242	9092.275391	13073.14941	12825.32324	13562.77246
Filtered	10867.65918	Filtered	Filtered	Filtered	Filtered	Filtered	11994.22559	Filtered	Filtered	Filtered	Filtered
Filtered	Filtered	Filtered	Filtered	12662.3457	8646.186523	7986.452148	Filtered	Filtered	Filtered	4605.865723	Filtered
Filtered	9457.053711	Filtered	Filtered	5658.714844	7445.924805	8400.956055	7344.062012	4808.65332	16437.37109	4532.465332	Filtered
Filtered	7161.403809	Filtered	14514.01953	14106.2041	12097.94531	14070.75098	11704.4668	11396.55859	12982.16797	13512.36035	12542.03613
Filtered	9551.917969	9987.72168	14270.68359	8412.634766	10896.37305	12200.32227	13302.35449	12073.96973	12177.16699	13866.61914	12279.72949
Filtered	Filtered	Filtered	9694.336914	Filtered	Filtered	Filtered	Filtered	Filtered	7478.948242	7661.317383	6194.881836
20824.23047	11070.45313	9761.444336	11301.52637	10188.53809	9922.259766	7163.939941	10064.49902	10967.67383	10246.42285	10233.23828	7385.697266
Filtered	Filtered	Filtered	Filtered	Filtered	Filtered	Filtered	3936.05127	4704.690918	4713.583496	Filtered	5107.920898
Filtered	4924.494141	Filtered	10057.45313	13324.82422	10658.27246	8191.855469	13121.58594	12907.61133	13471.77148	11122.99414	6889.780273
Filtered	6308.879883	5750.435059	9705.762695	22007.25977	21294.62891	22909.66797	15506.05176	16086.36426	14492.64453	17676.69727	15245.88281
Filtered	9115.327148	8802.386719	9827.448242	6298.935059	6772.495117	6676.515137	12243.98438	10306.96973	11846.71777	12194.18164	11797.76367
Filtered	13206.2002	Filtered	3733.712646	Filtered	Filtered	5876.02002	11525.18555	12070.06348	Filtered	14638.27637	12925.91309
Filtered	9173.041016	24328.11719	Filtered	19084.2832	Filtered	Filtered	Filtered	3461.465576	Filtered	Filtered	Filtered
Filtered	5422.5625	Filtered	2945.071777	Filtered	Filtered	Filtered	Filtered	Filtered	Filtered	Filtered	Filtered
Filtered	1895.826904	94681.96094	4308.587891	2571.575928	3954.363037	3714.753418	5143.253906	7625.022949	5865.36377	4415.325684	4403.128906
Filtered	16005.05078	8393.337891	10230.61035	9575.251953	6060.5625	4494.242188	8284.557617	10713.53906	10316.2959	9868.376953	11252.1543
Filtered	Filtered	Filtered	Filtered	Filtered	Filtered	21236.08984	Filtered	Filtered	Filtered	Filtered	Filtered
Filtered	2170.018799	Filtered	4500.495117	9765.765625	68762.74219	78998.5	Filtered	Filtered	5359.83252	3823.567383	4413.96875
Filtered	Filtered	Filtered	Filtered	17116.97852	8746.882813	10001.67285	Filtered	Filtered	Filtered	Filtered	Filtered
Filtered	9387.292969	Filtered	Filtered	15513.22168	5226.051758	7713.259277	10734.78027	11122.3877	7557.390625	11996.77344	12638.75977
Filtered	Filtered	Filtered	Filtered	Filtered	Filtered	Filtered	Filtered	Filtered	Filtered	Filtered	Filtered
Filtered	Filtered	65718.98438	Filtered	7589.456543	Filtered	Filtered	4527.773926	Filtered	8313.400391	5847.115234	4856.508301
17874.76758	Filtered	Filtered	Filtered	Filtered	Filtered	3427.20874	Filtered	9016.801758	7302.266113	Filtered	Filtered
12505.82324	13704.98926	5256.952637	16464.0293	5429.966309	4645.341797	3204.967041	9988.914063	11330.50879	10343.54102	8238.764648	9755.052734
11609.60742	17621.01758	8199.623047	12652.39551	9834.007813	4277.42334	5523.489258	10905.04492	11154.50586	11719.65332	9610.693359	9092.541016
Filtered	12870.4668	12598.21387	11218.69727	8495.692383	11461.80273	12071.89844	11152.9375	10568.4502	10208.64063	10560.40527	10992.14941
Filtered	9970.422852	9324.505859	9721.973633	13913.59766	13374.24805	13026.74414	13079.50293	9356.496094	8771.489258	9150.749023	9435.428711
21262.22461	11218.37988	11347.89258	10850.18945	9907.210938	16535.15625	16924.09961	10288.16797	9905.006836	10103.83984	9806.771484	10821.02051
Filtered	7959.292969	6252.09375	10816.26855	9845.703125	12812.10254	12041.89746	9382.523438	9288.917969	9793.964844	9165.59082	8478.166016
11597.56934	18662.96484	12613.76367	7596.119629	12393.12988	7917.485352	7839.793457	9325.366211	13263.24902	11691.01953	11314.48633	13169.44434
Filtered	Filtered	Filtered	Filtered	Filtered	Filtered	Filtered	1503.044922	Filtered	Filtered	Filtered	Filtered
Filtered	10606.5166	9117.299805	11690.33203	11038.35547	9580.887695	12262.89453	10996.78711	10355.64844	10763.45801	10419.06934	10704.99023
Filtered	10078.75391	Filtered	12750.43652	Filtered	10523.4043	9306.506836	5075.90918	8293.326172	6484.025879	6777.527344	6545.75
10686.91797	9842.707031	Filtered	8400.21875	7369.472168	9900.988281	11232.37305	11522.02246	12066.4873	12633.99219	10297.85547	10680.5791
Filtered	6240.018066	Filtered	10275.13379	2233.908203	Filtered	Filtered	19530.53711	10141.2207	18446.24805	8990.25	1942.212524
Filtered	8807.087891	12299.31445	12590.48633	9420.885742	1149.762817	5175.441895	11329.24414	13361.16699	8210.129883	8786.669922	12790.07227
Filtered	Filtered	Filtered	Filtered	Filtered	Filtered	Filtered	5571.40625	Filtered	5350.221191	Filtered	Filtered
Filtered	5054.887695	Filtered	2524.554443	Filtered	2232.27417	Filtered	7682.141602	Filtered	96466.20313	Filtered	Filtered
Filtered	10442.26563	12484.16504	6359.706543	8246.160156	9390.045898	8210.56543	11239.69824	10274.97754	10544.09375	8428.001953	10244.41113
Filtered	6061.905273	4599.441895	56144.29297	Filtered	2296.358887	2257.312988	3571.580322	Filtered	2877.648193	3343.444336	34347.10547
Filtered	9184.813477	9362.154297	13627.04395	11219.56836	11008.9707	13207.58398	8430.352539	8757.765625	9569.254883	10709.70996	9629.871094
Filtered	2295.044434	10719.34766	4153.931641	4085.179688	1675.216064	2206.85083	3391.749512	3380.143555	Filtered	9511.583008	3649.132813
Filtered	Filtered	6662.608887	5529.543945	Filtered	3525.306152	3495.717529	12384.97852	9810.46875	7932.061523	5868.239746	4651.01416
Filtered	12209.67773	8387.333984	11292.78125	9856.46582	9907.046875	10156.65137	8944.78418	8400.851563	8925.882813	9342.955078	9629.539063
Filtered	Filtered	Filtered	7643.867188	4237.058594	8760.092773	9729.192383	Filtered	8764.916016	7825.922363	Filtered	Filtered
Filtered	13932.06641	10476.39844	7765.679688	7502.341309	6108.878906	Filtered	10430.73438	9347.421875	9552.480469	9592.193359	9127.914063
Filtered	4657.679688	3168.466309	5112.697754	4553.67334	4843	7467.415527	4877.836914	8002.723633	6078.714355	4889.634277	4630.413086
3947.217529	23289.08984	35674.61328	9219.575195	7591.974121	6626.04834	5653.920898	8937.268555	10169.95313	7421.692871	8074.239258	7559.487305
Filtered	Filtered	Filtered	Filtered	Filtered	Filtered	Filtered	Filtered	Filtered	Filtered	Filtered	Filtered
Filtered	Filtered	Filtered	Filtered	Filtered	Filtered	Filtered	Filtered	Filtered	Filtered	Filtered	Filtered
Filtered	Filtered	Filtered	Filtered	Filtered	Filtered	Filtered	Filtered	Filtered	Filtered	Filtered	Filtered

Filtered	3588.868408	11274.07422	10405.69531	9495.383789	9760.144531	10037.99121	9454.109375	6439.954102	10302.83008	6958.629883	12723.08301
Filtered	Filtered	Filtered	8960.164063	11677.82129	Filtered	Filtered	6321.651367	4524.503418	10970.13574	9624.776367	Filtered
Filtered	4597.087402	7692.009277	18293.31641	8615.035156	Filtered	Filtered	9096.408203	8171.739746	7184.79248	7402.491211	9559.730469
Filtered	Filtered	5442.46875	12378.1748	Filtered	Filtered	Filtered	Filtered	Filtered	Filtered	Filtered	Filtered
Filtered	Filtered	Filtered	Filtered	Filtered	Filtered	Filtered	Filtered	Filtered	Filtered	Filtered	Filtered
Filtered	11456.79785	Filtered	10172.86133	Filtered	5040.563477	7497.419922	10450.5293	9323.979492	3046.504395	11477.64453	Filtered
Filtered	Filtered	Filtered	Filtered	Filtered	15925.9375	14478.69629	Filtered	Filtered	Filtered	Filtered	Filtered
Filtered	Filtered	Filtered	Filtered	7321.489746	Filtered	12359.12891	Filtered	Filtered	Filtered	Filtered	Filtered
15698.6748	7474.436035	8839.701172	8473.275391	5734.437012	6403.76123	7023.87793	6838.674316	6124.366699	8110.953613	7877.478027	7321.839355
Filtered	8225.098633	Filtered	8132.177246	10211.07422	4845.173828	3821.032227	11532.01758	8586.105469	9375.254883	6952.696289	4916.781738
Filtered	Filtered	11835.15723	13445.5918	10169.95801	4559.165527	3921.990479	6032.674805	10069.33887	11485.80176	10257.54785	10310.07422
Filtered	4862.80957	Filtered	8404.387695	10174.36719	5767.118652	7695.944336	4351.263184	5500.917969	Filtered	Filtered	5723.426758
Filtered	Filtered	Filtered	Filtered	6577.968262	11245.90527	9131.256836	Filtered	Filtered	Filtered	Filtered	Filtered
Filtered	Filtered	5972.37793	9837.03418	5178.891602	9922.689453	7761.67041	12221.51855	10827.52734	10336.21094	8692.357422	8964.358398
Filtered	8667.955078	9846.869141	8579.583008	5858.885254	5449.815918	5524.228027	8014.53125	7607.191406	8902.012695	8180.343262	7406.425781
Filtered	Filtered	Filtered	12633.46777	10955.70703	16282.0957	17827.62305	Filtered	9140.225586	8594.871094	14631.85449	11347.81348
Filtered	Filtered	Filtered	10780.9873	Filtered	Filtered	Filtered	Filtered	10531.10547	7834.20752	Filtered	4799.340332
Filtered	6701.694824	11708.02734	7017.225098	11539.24219	6320.594238	6629.415527	5728.365234	5560.956543	5556.62793	5317.915527	5836.837891
Filtered	Filtered	Filtered	Filtered	Filtered	2713.106445	3251.090088	2238.48584	Filtered	Filtered	Filtered	Filtered
Filtered	4454.436523	Filtered	6103.199219	8572.99707	9325.274414	7938.987305	8491.270508	9201.561523	8724.383789	Filtered	8123.941895
Filtered	Filtered	Filtered	12997.5498	Filtered	6239.77002	7227.180664	Filtered	Filtered	10954.44043	10874.31836	Filtered
Filtered	Filtered	Filtered	Filtered	Filtered	6601.318848	5202.839355	Filtered	Filtered	Filtered	Filtered	4660.301758
Filtered	Filtered	Filtered	7444.203613	7020.088867	7352.73877	8190.919434	5634.497559	6511.65332	4675.78125	Filtered	5426.977539
Filtered	5451.23877	Filtered	7550.02832	Filtered	5016.739258	6661.65332	9523.855469	8758.415039	7519.052734	7736.063965	Filtered
Filtered	7741.033203	Filtered	Filtered	Filtered	3523.21582	3995.797607	6173.261719	10115.06445	4614.083496	Filtered	Filtered
Filtered	8109.300781	7916.285156	11955.35742	8536.601563	12633.4082	12917.92578	8203.655273	10842.13379	8894	7294.68457	8662.255859
Filtered	6119.170898	Filtered	Filtered	Filtered	Filtered	Filtered	Filtered	Filtered	Filtered	Filtered	Filtered
Filtered	7959.245605	Filtered	4272.273926	Filtered	Filtered	Filtered	5090.64209	6212.103516	5336.320313	5827.442871	5516.299316
Filtered	Filtered	Filtered	7599.620117	Filtered	8635.040039	8112.523438	5240.59668	4913.958984	4997.648438	6258.449219	Filtered
13458.70703	9135.708984	Filtered	Filtered	Filtered	Filtered	Filtered	10092.58984	8343.09668	6862.188477	7962.554688	Filtered
Filtered	Filtered	Filtered	4127.836426	10258.4668	28027.04883	7538.972656	8462.167969	2662.725342	3837.300293	4668.229004	5349.356934
Filtered	7058.121094	Filtered	8863.911133	5207.970215	9300.387695	9988.709961	Filtered	8405.735352	7081.347656	6724.447266	8835.3125
Filtered	Filtered	Filtered	Filtered	Filtered	Filtered	10656.34277	Filtered	Filtered	Filtered	Filtered	Filtered
Filtered	9895.963867	8204.697266	10320.56641	7735.535156	5792.720215	6238.538086	8663.839844	8406.063477	7008.283203	11628.03125	7198.44873
Filtered	Filtered	11083.9668	5142.644531	5921.250977	6016.681152	7763.803711	7305.054688	6978.414063	7188.663086	7537.269531	6969.299316
Filtered	10467.16992	7726.680664	10181.57031	Filtered	5009.890625	4604.656738	8698.646484	7857.401367	10599.07813	6997.299805	9766.546875
3369.269043	7722.398926	6043.188477	6088.577148	Filtered	10321.99512	6389.196777	9345.30957	7117.696289	9114.808594	8397.482422	9147.001953
Filtered	3927.261963	Filtered	9335.237305	7879.730469	5507.824219	5542.365723	6952.55127	9120.96875	4939.560059	4596.888672	6972.083496
Filtered	Filtered	Filtered	6477.927246	Filtered	7008.533691	7913.652344	5973.201172	Filtered	3992.12085	Filtered	2803.973633
6901.114258	8011.561523	7851.461914	7929.918945	8688.222656	10106.10645	10392.5	7328.337402	7384.980469	8352.994141	7323.908691	6783.164063
Filtered	Filtered	Filtered	12715.0957	Filtered	Filtered	Filtered	Filtered	Filtered	Filtered	Filtered	Filtered
Filtered	5600.688477	5787.75293	9421.00293	9431.894531	9705.923828	12477.43164	6088.212891	7132.472656	4063.101318	7093.23877	6844.483398
10456.30469	5280.047852	8675.379883	13911.14453	6210.381836	6921.79834	6984.971191	7718.991699	7918.821289	8252.439453	7463.037598	6465.524902
Filtered	7878.149902	4116.283203	9278.461914	7729.76709	Filtered	Filtered	Filtered	4849.259277	6806.03418	5119.156738	Filtered
18752.98438	Filtered	Filtered	8630.155273	5313.47168	Filtered	Filtered	Filtered	1929.204468	Filtered	4972.849121	6830.200684
Filtered	Filtered	Filtered	Filtered	Filtered	Filtered	Filtered	Filtered	Filtered	Filtered	Filtered	Filtered
Filtered	7066.570313	9325.415039	4167.652832	Filtered	Filtered	Filtered	8871.614258	8474.648438	8708.835938	7787.885742	5721.666504
Filtered	Filtered	Filtered	Filtered	Filtered	4497.994141	4873.041504	Filtered	Filtered	Filtered	Filtered	Filtered
Filtered	8816.689453	Filtered	8373.588867	7427.275391	6908.487305	Filtered	7986.481445	Filtered	8036.475586	7545.057617	Filtered
Filtered	2015.759155	Filtered	4695.174805	Filtered	1667.895264	2115.319092	2230.261963	2965.775146	Filtered	Filtered	Filtered
Filtered	8290.760742	Filtered	5420.851563	3237.510986	3702.037354	3794.539551	5273.456543	7016.59082	2943.364502	3652.199707	Filtered
Filtered	7432.835449	6120.892578	8111.008789	8148.050293	11708.72949	8306.913086	6728.318848	9982.537109	7540.008789	7660.635742	8463.618164
Filtered	4165.075195	9390.480469	11791.10938	1658.590454	Filtered	Filtered	5211.908203	5870.586426	4204.711914	5664.668945	5555.707031
Filtered	4776.463867	Filtered	5835.077637	Filtered	3188.800781	6162.296875	7796.280273	8374.556641	8378.248047	6337.035645	6677.325195
Filtered	Filtered	Filtered	8461.032227	10881.28711	8223.448242	6228.554688	7339.751465	8025.913086	7749.805176	7057.115234	7820.085938
Filtered	7242.551758	Filtered	5606.053711	3467.63916	9980.93457	10827.24707	8596.37793	5317.806152	7606.643555	8227.160156	9342.966797
Filtered	10821.38477	5668.811523	12209.96094	7427.80957	11811.05762	11001.90527	6628.904785	6700.029297	6894.508789	6062.161133	7484.117676
Filtered	Filtered	Filtered	2740.999268	3271.406738	20983.68945	21041.80078	6720.416992	Filtered	1688.155151	Filtered	Filtered
Filtered	3853.508545	10306.46191	7433.828125	Filtered	5138.416016	5754.990723	7848.932129	6093.53418	7106.084961	5403.306641	2031.543091
Filtered	Filtered	Filtered	Filtered	Filtered	Filtered	Filtered	Filtered	Filtered	Filtered	Filtered	Filtered
Filtered	3364.66748	9422.772461	5969.79248	4431.922363	5297.084961	4398.411133	4308.309082	11256.35156	10141.74219	11322.44629	11601.48828
Filtered	Filtered	Filtered	Filtered	Filtered	Filtered	Filtered	Filtered	Filtered	Filtered	2931.869141	Filtered
Filtered	Filtered	Filtered	6090.696777	Filtered	4947.04834	4028.239746	Filtered	2365.612061	3856.56958	Filtered	5380.992188
Filtered	4948.117188	8525.896484	8144.728516	7276.458984	7387.588867	9558.84375	6201.056641	6794.785156	5572.197266	4804.992676	7005.90332
Filtered	Filtered	Filtered	Filtered	Filtered	Filtered	Filtered	Filtered	Filtered	5573.404785	9108.673828	4607.852051
Filtered	Filtered	Filtered	7342.433105	Filtered	Filtered	Filtered	6379.47168	Filtered	Filtered	Filtered	6367.440918
Filtered	Filtered	Filtered	Filtered	10789.80762	Filtered	Filtered	Filtered	Filtered	Filtered	Filtered	Filtered
2735.440918	6124.620117	2750.854736	7752.847656	1343.329956	9106.751953	12217.87695	10282.90234	6197.882813	6208.71582	2331.37915	9294.977539
10128.07129	7440.649902	8525.230469	5539.787109	Filtered	Filtered	Filtered	3224.126953	Filtered	Filtered	Filtered	4059.788574
Filtered	Filtered	Filtered	3895.161377	7932.939453	Filtered	Filtered	2287.911377	2994.068115	1406.082397	2557.188232	Filtered
Filtered	5711.985352	8341.429688	8773.347656	8353.197266	4049.202881	6027.509766	6412.476074	7044.931152	4236.799805	6357.078613	5195.052246
Filtered	6972.222656	Filtered	7508.523926	Filtered	7833.44043	7125.256348	7400.228027	6960.309082	Filtered	5498.780273	6751.966309
Filtered	5372.696777	Filtered	6550.106445	8605.786133	7879.138184	7959.916016	7888.303223	7242.730957	3990.341064	7014.52832	7754.421387
Filtered	Filtered	Filtered	Filtered	Filtered	4392.495605	5502.740723	Filtered	Filtered	Filtered	Filtered	Filtered
Filtered	Filtered	Filtered	Filtered	Filtered	8353.538086	7788.70166	Filtered	Filtered	Filtered	2768.927979	Filtered

Filtered	7282.735352	Filtered	7939.449219	Filtered	6727.043457	6284.599609	8247.46875	8769.538086	8565.707031	4528.375488	6755.006836
Filtered	5539.776367	Filtered	Filtered	Filtered	5356.536133	3975.454834	Filtered	Filtered	Filtered	Filtered	Filtered
Filtered	4875.998047	6052.911133	4775.660156	Filtered	8021.359375	6023.557617	5662.696289	7417.569336	6954.219727	5299.24707	5621.850586
Filtered	5363.824707	10532.99805	6617.940918	8642.446289	5587.3125	6452.207031	5360.71875	4278.821777	4744.041992	6724.928711	5651.834473
Filtered	Filtered	Filtered	Filtered	Filtered	6760.971191	6339.921875	Filtered	Filtered	Filtered	Filtered	4297.254395
Filtered	31966.26953	3237.918457	5184.12207	3610.230225	7389.014648	8053.678711	7009.145996	4965.628418	5810.068359	4901.643555	5448.867188
Filtered	3400.99292	Filtered	Filtered	Filtered	20401.33594	15447.5625	3897.80249	Filtered	5339.964844	3469.290527	3253.190918
Filtered	5926.793945	Filtered	2105.488037	2471.313477	Filtered	Filtered	Filtered	Filtered	Filtered	Filtered	Filtered
Filtered	Filtered	Filtered	5221.223145	Filtered	2702.858398	4748.128418	Filtered	3634.612305	4166.162598	Filtered	Filtered
Filtered	Filtered	Filtered	Filtered	Filtered	2344.274414	2336.158203	Filtered	Filtered	Filtered	Filtered	Filtered
Filtered	5583.618164	5916.782227	7901.493652	Filtered	Filtered	Filtered	1788.676147	4660.140137	Filtered	5951.723633	1778.452515
Filtered	Filtered	Filtered	9395.538086	Filtered	Filtered	Filtered	Filtered	Filtered	Filtered	Filtered	Filtered
4499.478027	Filtered	Filtered	Filtered	16028.18066	15304.99805	Filtered	3726.263916	Filtered	2609.040527	3127.966797	3508.020508
Filtered	Filtered	Filtered	6986.800781	Filtered	5105.162109	5472.004883	Filtered	Filtered	Filtered	Filtered	Filtered
Filtered	5913.059082	15504.60254	Filtered	Filtered	4116.445801	5067.407227	4593.223145	Filtered	4080.645264	Filtered	4824.640625
Filtered	4615.26123	5689.950195	9786.898438	Filtered	5511.563477	5778.736328	9698.283203	Filtered	Filtered	Filtered	Filtered
Filtered	6399.304199	Filtered	5513.428223	4938.807617	Filtered	Filtered	5988.02002	5662.279297	4474.959961	6101.782715	5658.471191
Filtered	5925.460449	5761.468262	7713.169922	Filtered	Filtered	7041.40918	4285.645508	3650.552002	1517.268311	Filtered	Filtered
Filtered	8304.445313	Filtered	Filtered	Filtered	Filtered	3541.997803	6897.601074	6790.63916	5307.225586	Filtered	5440.814941
Filtered	7899.071777	Filtered	2585.837646	Filtered	Filtered	Filtered	Filtered	Filtered	Filtered	Filtered	Filtered
Filtered	Filtered	Filtered	Filtered	Filtered	Filtered	Filtered	3343.154541	Filtered	Filtered	Filtered	Filtered
Filtered	3770.262207	Filtered	5938.008789	Filtered	2465.100342	Filtered	4219.802246	Filtered	4160.401855	4311.344238	Filtered
Filtered	Filtered	Filtered	Filtered	Filtered	Filtered	Filtered	Filtered	4811.187988	Filtered	3216.446289	Filtered
Filtered	Filtered	Filtered	8586.668945	Filtered	Filtered	4390.150879	Filtered	Filtered	Filtered	4843.884766	Filtered
Filtered	4461.391113	Filtered	Filtered	Filtered	3929.260254	4579.271973	Filtered	1075.921631	Filtered	Filtered	2744.012695
Filtered	5438.182129	6256.237305	4910.971191	Filtered	4016.753418	4095.761719	5459.851563	5536.622559	4556.742188	3992.560791	4938.938477
Filtered	Filtered	Filtered	Filtered	Filtered	Filtered	Filtered	4327.62793	3899.968018	Filtered	Filtered	3638.93457
Filtered	Filtered	Filtered	Filtered	Filtered	Filtered	Filtered	Filtered	Filtered	Filtered	Filtered	Filtered
Filtered	Filtered	Filtered	Filtered	Filtered	Filtered	Filtered	5504.092285	4911.436035	4956.00293	Filtered	Filtered
Filtered	Filtered	Filtered	5138.556641	Filtered	2872.781982	3926.494141	Filtered	Filtered	Filtered	Filtered	Filtered
Filtered	Filtered	Filtered	7481.994629	Filtered	Filtered	Filtered	4119.209961	Filtered	4020.065186	4238.172852	4253.949707
Filtered	3448.421875	3946.929688	5485.254395	4014.068848	6093.395508	4515.575195	2528.767334	Filtered	3586.407715	1396.986328	5187.896973
Filtered	Filtered	5379.513672	Filtered	6840.424316	4302.039551	4448.850586	3669.364746	4507.087402	Filtered	3238.973389	3235.094482
Filtered	Filtered	Filtered	6748.991211	Filtered	Filtered	Filtered	4607.374023	Filtered	Filtered	Filtered	Filtered
Filtered	Filtered	Filtered	Filtered	Filtered	Filtered	Filtered	Filtered	Filtered	Filtered	Filtered	Filtered
Filtered	5418.16748	4951.345703	3988.97168	5421.338867	2434.866943	2474.477295	6677.85791	5240.745117	6753.742676	7143.354004	6071.779785
Filtered	7354.915527	3509.554443	Filtered	Filtered	4144.220703	3764.331055	4161.773926	Filtered	Filtered	Filtered	Filtered
Filtered	Filtered	Filtered	Filtered	Filtered	Filtered	Filtered	Filtered	Filtered	3239.685547	Filtered	Filtered
Filtered	6966.463867	4202.93457	8173.45752	5116.755859	5857.983398	4717.371094	4517.727539	5285.851563	4514.451172	6047.160156	5895.64502
Filtered	10041.02539	Filtered	6830.635742	Filtered	Filtered	3672.58374	5757.513672	Filtered	5482.895996	3622.486084	Filtered
Filtered	5546.499512	6718.228516	3009.647461	Filtered	12369.94531	4568.049805	2190.604004	5619.518066	Filtered	1577.729004	Filtered
Filtered	Filtered	Filtered	Filtered	Filtered	Filtered	Filtered	Filtered	Filtered	Filtered	Filtered	Filtered
Filtered	Filtered	Filtered	Filtered	2349.731445	4754.067871	4052.715332	Filtered	Filtered	Filtered	Filtered	Filtered
Filtered	Filtered	Filtered	Filtered	Filtered	Filtered	4157.897949	5091.516113	3006.665283	3479.011963	Filtered	Filtered
Filtered	Filtered	Filtered	Filtered	Filtered	Filtered	Filtered	Filtered	Filtered	Filtered	Filtered	Filtered
Filtered	Filtered	Filtered	Filtered	Filtered	Filtered	Filtered	Filtered	Filtered	Filtered	Filtered	Filtered
Filtered	2438.741455	Filtered	Filtered	Filtered	9756.188477	9529.900391	Filtered	Filtered	4867.586426	Filtered	Filtered
Filtered	3373.310791	Filtered	Filtered	Filtered	Filtered	Filtered	3020.364746	3059.76416	Filtered	Filtered	Filtered
Filtered	Filtered	Filtered	Filtered	Filtered	Filtered	Filtered	4118.872559	Filtered	Filtered	Filtered	Filtered
Filtered	Filtered	Filtered	1207.099731	Filtered	Filtered	6327.808105	Filtered	2071.316406	Filtered	Filtered	3935.087402
Filtered	3009.313477	Filtered	7634.258789	5659.750977	2987.956055	3309.673096	4812.208496	4736.300781	5352.916504	4291.520996	5008.768555
Filtered	1870.497314	Filtered	8885.279297	Filtered	2765.743896	3671.378906	Filtered	Filtered	Filtered	7340.860352	10865.72559
Filtered	4701.851074	Filtered	5033.814941	Filtered	Filtered	Filtered	3423.735596	Filtered	4004.520752	2601.150146	4952.622559
Filtered	2623.595947	Filtered	4245.675781	Filtered	Filtered	Filtered	4520.140137	3584.439209	4320.67334	4412.225098	3685.960205
Filtered	Filtered	Filtered	5486.5625	2675.617432	7769.583496	7497.914551	8445.838867	4496.342773	3564.941406	5611.811523	4302.064453
Filtered	Filtered	6909.442871	Filtered	6518.621094	Filtered	Filtered	Filtered	Filtered	Filtered	Filtered	Filtered
Filtered	Filtered	Filtered	Filtered	4437.353027	Filtered	Filtered	Filtered	Filtered	Filtered	Filtered	Filtered
Filtered	4336.880859	Filtered	4298.599121	5686.958984	5942.966309	5705.39209	Filtered	Filtered	4966.093262	5797.655273	6223.76123
Filtered	2996.898926	Filtered	3980.000244	Filtered	5395.791016	4850.116211	Filtered	5344.428223	Filtered	Filtered	Filtered
Filtered	Filtered	Filtered	2735.204346	Filtered	5344.982422	4020.390869	4322.133301	4397.146484	3584.636475	Filtered	Filtered
Filtered	Filtered	Filtered	Filtered	Filtered	5513.692383	6505.314453	4157.580566	5771.018066	4447.110352	4716.660156	3509.626465
Filtered	5525.375	Filtered	Filtered	Filtered	1786.196533	Filtered	Filtered	3336.455811	Filtered	2535.167725	Filtered
Filtered	Filtered	Filtered	Filtered	4631.037109	13281.53809	15118.85938	3065.545898	1843.156372	Filtered	1004.244995	Filtered
Filtered	Filtered	Filtered	Filtered	Filtered	3176.629395	2833.432617	Filtered	Filtered	Filtered	2202.845459	Filtered
Filtered	Filtered	Filtered	1509.010986	Filtered	Filtered	Filtered	Filtered	Filtered	Filtered	Filtered	Filtered
Filtered	4782.085449	1455.115723	4759.853516	3875.034668	3747.751465	4491.677246	6322.805664	5805.709473	6037.585449	2780.608154	5137.57373
Filtered	6757.271484	3073.527832	4314.79248	3960.050537	3464.532715	4428.977539	4545.495605	Filtered	4468.529297	2601.398926	4522.583008
Filtered	Filtered	Filtered	Filtered	Filtered	Filtered	Filtered	Filtered	Filtered	Filtered	Filtered	Filtered
Filtered	Filtered	Filtered	Filtered	2968.390869	4276.564941	6391.024414	Filtered	2447.364746	Filtered	Filtered	Filtered
Filtered	Filtered	Filtered	Filtered	3860.161133	Filtered	Filtered	4608.420898	3820.035889	Filtered	4598.294922	Filtered
Filtered	11668.63281	Filtered	3021.629395	Filtered	2528.153809	3108.563721	4403.563965	3305.531494	3364.496582	3594.865234	3060.687988
Filtered	2653.308594	Filtered	Filtered	Filtered	Filtered	2635.507568	3859.440918	4287.883789	3557.410889	4533.285156	4251.837891
Filtered	Filtered	Filtered	Filtered	6440.626953	Filtered	Filtered	Filtered	Filtered	Filtered	Filtered	Filtered
Filtered	3607.905762	12140.18164	5979.12207	6574.002441	5351.512695	5317.222168	Filtered	3096.63623	2546.331787	6443.458496	Filtered
Filtered	Filtered	Filtered	5184.431152	Filtered	Filtered	Filtered	3248.089844	Filtered	Filtered	3658.912354	2555.040039

Filtered	Filtered	Filtered	Filtered	Filtered	Filtered	Filtered	Filtered	Filtered	Filtered	Filtered	Filtered
Filtered	Filtered	Filtered	Filtered	Filtered	Filtered	Filtered	Filtered	Filtered	Filtered	Filtered	Filtered
Filtered	Filtered	Filtered	Filtered	Filtered	2659.607666	Filtered	3316.758057	3027.893066	2945.486816	Filtered	Filtered
Filtered	Filtered	Filtered	Filtered	Filtered	2743.585205	3134.240967	Filtered	3490.62915	Filtered	Filtered	Filtered
Filtered	Filtered	Filtered	Filtered	Filtered	Filtered	Filtered	Filtered	Filtered	Filtered	Filtered	Filtered
Filtered	Filtered	Filtered	Filtered	Filtered	Filtered	Filtered	Filtered	Filtered	Filtered	Filtered	Filtered
Filtered	Filtered	Filtered	Filtered	Filtered	Filtered	Filtered	Filtered	Filtered	Filtered	Filtered	Filtered
Filtered	Filtered	Filtered	Filtered	Filtered	Filtered	Filtered	Filtered	Filtered	Filtered	Filtered	Filtered
Filtered	Filtered	Filtered	Filtered	Filtered	Filtered	Filtered	3969.560303	3222.148438	4720.479004	Filtered	4546.915527
Filtered	Filtered	Filtered	Filtered	Filtered	5429.873535	5943.081543	Filtered	Filtered	Filtered	Filtered	Filtered
Filtered	3731.249756	Filtered	2570.245605	Filtered	5993.020508	3976.883057	Filtered	Filtered	Filtered	Filtered	Filtered
Filtered	Filtered	Filtered	Filtered	Filtered	Filtered	Filtered	Filtered	Filtered	Filtered	Filtered	Filtered
Filtered	Filtered	Filtered	Filtered	Filtered	Filtered	Filtered	Filtered	4374.791992	3244.165283	3220.786133	2586.132813
Filtered	Filtered	Filtered	Filtered	Filtered	Filtered	3980.510498	Filtered	1909.901489	Filtered	Filtered	Filtered
Filtered	Filtered	Filtered	Filtered	Filtered	5502.847168	Filtered	Filtered	Filtered	Filtered	Filtered	Filtered
Filtered	Filtered	Filtered	Filtered	Filtered	Filtered	Filtered	Filtered	Filtered	Filtered	Filtered	Filtered
Filtered	Filtered	Filtered	3728.897461	Filtered	Filtered	1231.950195	3324.167236	2386.131836	2871.853516	2744.535645	2230.008545
Filtered	Filtered	Filtered	Filtered	Filtered	Filtered	Filtered	Filtered	Filtered	Filtered	Filtered	Filtered
Filtered	Filtered	Filtered	Filtered	Filtered	Filtered	Filtered	Filtered	Filtered	Filtered	Filtered	Filtered
Filtered	3689.245361	8768.175781	Filtered	Filtered	Filtered	Filtered	Filtered	Filtered	Filtered	Filtered	Filtered
Filtered	4682.09375	Filtered	Filtered	Filtered	4934.510254	Filtered	Filtered	1858.289551	Filtered	Filtered	2409.051514
Filtered	1710.174805	Filtered	Filtered	1764.127808	5044.711426	4161.084961	4279.717285	Filtered	2607.878662	2880.675781	3663.912842
Filtered	4120.397461	Filtered	3370.264404	1625.422974	6014.228516	5993.587891	3289.351074	2897.029297	3213.736084	3834.663574	3857.049316
Filtered	Filtered	Filtered	Filtered	Filtered	4477.723633	5213.462891	Filtered	Filtered	Filtered	Filtered	Filtered
Filtered	Filtered	Filtered	Filtered	Filtered	Filtered	Filtered	Filtered	Filtered	Filtered	Filtered	Filtered
Filtered	2563.271729	3534.093262	Filtered	Filtered	Filtered	Filtered	3499.715088	2890.622559	2882.630859	3558.759033	2944.021729
Filtered	2744.862793	Filtered	Filtered	Filtered	4140.434082	Filtered	2611.149658	Filtered	Filtered	Filtered	Filtered
Filtered	Filtered	Filtered	Filtered	Filtered	Filtered	Filtered	Filtered	Filtered	Filtered	Filtered	Filtered
Filtered	Filtered	Filtered	Filtered	Filtered	Filtered	Filtered	Filtered	Filtered	Filtered	Filtered	Filtered
Filtered	3426.837891	Filtered	3741.945313	2480.291016	Filtered	Filtered	2189.100586	1742.414429	1888.636108	2272.184326	2249.138672
Filtered	Filtered	Filtered	Filtered	Filtered	Filtered	Filtered	2802.071045	1334.509521	2103.262451	Filtered	Filtered
Filtered	3765.920166	Filtered	Filtered	Filtered	5664.724121	5884.57666	2205.927002	Filtered	Filtered	Filtered	Filtered
Filtered	2591.661621	2463.184814	4498.125977	4628.349121	2919.498047	2041.937988	Filtered	3147.436279	Filtered	2981.381592	3562.287354
Filtered	Filtered	Filtered	Filtered	Filtered	Filtered	Filtered	2289.833008	Filtered	Filtered	Filtered	Filtered
Filtered	Filtered	Filtered	1191.266968	1613.430908	3834.562988	3337.966553	1562.406738	1751.869995	2548.102295	1747.378052	1438.665771
Filtered	Filtered	Filtered	Filtered	Filtered	Filtered	Filtered	Filtered	1914.930176	Filtered	Filtered	Filtered
Filtered	Filtered	Filtered	Filtered	Filtered	Filtered	Filtered	Filtered	Filtered	Filtered	Filtered	Filtered
Filtered	Filtered	Filtered	Filtered	Filtered	Filtered	Filtered	Filtered	Filtered	Filtered	Filtered	Filtered
Filtered	Filtered	Filtered	Filtered	Filtered	Filtered	Filtered	Filtered	Filtered	Filtered	Filtered	Filtered
4640.01123	Filtered	Filtered	Filtered	Filtered	Filtered	Filtered	Filtered	Filtered	Filtered	Filtered	Filtered
Filtered	Filtered	Filtered	Filtered	Filtered	1843.044678	Filtered	Filtered	Filtered	Filtered	Filtered	1135.296997
Filtered	1476.938721	Filtered	2161.792969	Filtered	3165.270508	Filtered	2168.584229	Filtered	Filtered	1876.712036	Filtered
Filtered	Filtered	Filtered	Filtered	Filtered	Filtered	Filtered	Filtered	Filtered	Filtered	Filtered	Filtered
Filtered	Filtered	Filtered	Filtered	Filtered	Filtered	Filtered	Filtered	Filtered	Filtered	Filtered	Filtered
Filtered	Filtered	Filtered	Filtered	Filtered	Filtered	Filtered	Filtered	Filtered	Filtered	Filtered	Filtered
Filtered	3003.241455	Filtered	Filtered	Filtered	Filtered	Filtered	Filtered	Filtered	Filtered	Filtered	Filtered
Filtered	Filtered	Filtered	Filtered	Filtered	Filtered	Filtered	Filtered	Filtered	Filtered	Filtered	Filtered
Filtered	Filtered	Filtered	1946.07251	Filtered	Filtered	Filtered	Filtered	Filtered	Filtered	Filtered	Filtered
Filtered	Filtered	Filtered	Filtered	Filtered	Filtered	Filtered	Filtered	Filtered	Filtered	2379.141602	Filtered
Filtered	Filtered	Filtered	Filtered	Filtered	Filtered	Filtered	Filtered	Filtered	Filtered	Filtered	Filtered
Filtered	Filtered	Filtered	Filtered	Filtered	Filtered	954.2178955	Filtered	Filtered	Filtered	Filtered	Filtered
Filtered	Filtered	Filtered	Filtered	Filtered	Filtered	Filtered	Filtered	1886.616577	Filtered	Filtered	2824.430908
Filtered	Filtered	Filtered	Filtered	Filtered	Filtered	Filtered	Filtered	982.5679321	Filtered	Filtered	Filtered
Filtered	2781.739746	Filtered	875.8726807	1027.336792	1333.919189	2096.878906	Filtered	Filtered	1607.287354	1411.608521	Filtered
Filtered	Filtered	Filtered	Filtered	Filtered	Filtered	Filtered	Filtered	Filtered	Filtered	Filtered	Filtered
Filtered	Filtered	Filtered	Filtered	Filtered	Filtered	Filtered	Filtered	Filtered	Filtered	Filtered	Filtered
Filtered	Filtered	Filtered	Filtered	Filtered	Filtered	1632.646851	Filtered	Filtered	Filtered	Filtered	Filtered
Filtered	Filtered	Filtered	Filtered	Filtered	1447.696167	Filtered	Filtered	Filtered	Filtered	1227.255737	Filtered
Filtered	Filtered	Filtered	Filtered	Filtered	1293.609131	Filtered	Filtered	Filtered	Filtered	Filtered	Filtered
Filtered	Filtered	Filtered	Filtered	Filtered	Filtered	Filtered	Filtered	Filtered	Filtered	Filtered	Filtered
Filtered	Filtered	Filtered	Filtered	Filtered	Filtered	Filtered	Filtered	Filtered	Filtered	Filtered	Filtered

QC_6	QC_7
147504896	132661584
79815952	99980624
32000642	28978814
20985076	22480420
18309014	17983316
19927600	17360222
17185430	14105416
17782830	14574949
15325967	14244230
13949474	12142551
12870516	11406972
9729488	8867305
10174920	8675030
10000382	8990038
9213455	8394867
6444208.5	8483332
5734856	7916285
8876702	8044168
9398417	7968933.5
65264.22656	62860.60938
7696261	6873494.5
6979787.5	6863623
5540971.5	6398730
5549312.5	5284380.5
6822902	5960035.5
6817421	6133968.5
6294146.5	5453669
6613511	5089571
5779523.5	5013832.5
5693802	4935342
5247130.5	4517167.5
5295579	4939339.5
4705920	3718822.5
4955555.5	4403638
5242527.5	4818232.5
5630848	4738129.5
2951837.5	3855058.25
4971874	3677011.25
3912545.5	3411204.25
13298.47461	Filtered
3593135.25	2219408.25
3837311.75	3178405.25
2310210.5	3380203.5
1076956.5	3648049
3113916.5	2932827.5
2762703.5	2412122
2682913.5	2503940
4085476.75	3247016
2722230	2612284.75
2645641.75	2231470.25
2683416.5	2515840
3524486.5	2654038.25
2478339.75	2180505
2470330	2119519.25
2173765.5	2229937
2244641	1973182.5
2406093.5	2068293.125
2199356.25	1985794.75
2170706	1922935.75
1988755	1962450
823345.75	2056994
2216149	2066448.375
2096320.125	1833977.25
2317804.5	1937501.5
3019338.5	2613752.75
1783636.5	1450618
1992931.125	1520883.75
2065697.875	1671275.25
800999.25	1885554.5
2099529.75	1851846.875
1659089.25	1467547
680238.125	1033624.125
1766465.375	1557580.625

1628685.75	1368874.875
1700678.625	2095738.125
1752128.5	1331687
1491996.625	1335962.375
1542313.5	1375219.5
2014034.625	1529273.5
1565630.375	1325860.5
1562934.125	1369234.375
1954735	728341.5625
1223399.5	1153148
1506737.25	1287241.125
1245065.125	1086944.875
1294778.875	1179701
1270674.125	1169448.75
1420094.125	1148177
1280406.5	1063773
1167902.75	1004188.125
907477.3125	1168804.75
334616.4063	619275.6875
878808.375	1206649.875
3475442	1541918
1086810.25	1005603.813
212246.1094	226518.6563
1056103	1031131.188
1021312.063	882762.5625
799460.125	856698.625
824947.625	1029956.5
539170.5	749962.375
959831.75	819666.75
875770.1875	760790.5625
679545.75	698579.6875
845557.3125	690910.625
925847.875	824069.875
666163.8125	153861.6406
791552.875	339288.1563
506380.9688	720765.875
691888.4375	602274.375
131657.5156	115539.8984
720591.25	559966.625
Filtered	536819.875
600971.8125	540000.1875
655280.75	542701.125
610897.125	539163.125
469687.9375	455846.75
763968.75	663805
547877	531148.875
445909.3125	514782.4688
587010.75	561710.5
506306.375	494726.625
502165.0625	459669.5313
538723.6875	476426.0313
471139.2188	413579.1563
631028.5625	432809.7188
507283.5313	436165.9688
652691	22804.19922
309870.0625	524014.875
315801.5	308940.4375
486007.6875	407678.0938
539308.25	426467.5625
431761.7188	524289.0625
350902.2188	315369.9375
205037	407054.125
289148.125	138691.1406
421261	357697.6563
Filtered	Filtered
372545.4688	337941.1875
400652.25	342571.6563
343631.5313	313234.9063
449060.625	361781.6875
384902	327397.8438
314430.875	288149.375
374749.75	318966.8438
346226.4688	283111.875
Filtered	340933.4375
343141.3125	316968.2188

230208.9219	206827.4375
Filtered	303709.9063
326275.9688	297724.1563
355056.7188	349374.0625
272135.2188	240738.4219
Filtered	168449.9219
369351.5313	339474.3438
35628.05859	Filtered
Filtered	Filtered
297821.8438	263864.0938
281228.5313	252108.375
304270.9688	256732.2656
291233.3125	258875.2031
Filtered	261116.4688
159072.0156	149755.8594
368855.3125	173675.6563
257223.9375	233032.4688
211979.9219	197925.4375
296886.4063	238217.6563
241823.75	216642.25
160785.5313	187308.4531
255376.8438	252413.2188
162123.1094	149751.6406
199412.5	188887.9063
253085.7813	293353.9063
146912.3281	125847.6484
261514.9688	214669.9219
215098.3281	222308.9688
174002.9844	210989.5781
269872.0625	275678.7188
274765.2188	230250.5938
267275.875	228463.3438
214955.6406	199484.2188
176220.8125	154016.2344
236971.6719	210390.5938
202960.5938	167102.3125
216070.7656	183046.5
10370.90039	24305.56641
214027.9688	176570.125
200341.7031	177158.8594
190896.4531	157618.7031
Filtered	3616.287842
183575.7344	157623.75
Filtered	Filtered
231997.0313	97434.625
118773.2266	113844.7422
160707.2188	161699.1875
181083.2031	147608.5781
160721.2031	149668.0313
137442.8438	120153.9531
189404.5156	158360.5469
197355.5313	162640.4219
123036.3281	109106.1719
Filtered	Filtered
173761.6719	154219.4531
178635.1094	170643.5781
80618.34375	50143.07422
56207.52734	46534.41016
29606.54492	19086.86523
97480.17969	124062.2188
2.20E+05	1.97E+05
153517	131132.8438
144938.9063	83680.46875
58826.80078	53756.12891
153382.5625	155861.5781
174054.125	133947.5156
144096.4531	124879.6484
146894.875	139777.7813
22724.19141	17098.54492
Filtered	119358.6016
129752.0469	114692.25
138968.9219	138006.2969
136652.5469	154518.0938
93941.96875	182632.7188
16924.3418	2607.222656

139772.3594	126295.8125
94753.35938	107248.3438
169689.2031	126872.9531
129735.9453	112554.0313
Filtered	114045.5
Filtered	Filtered
120526.4375	109800.7266
85314.96875	72290.15625
99766.75781	102541.3594
Filtered	Filtered
154986.5625	142444.2188
Filtered	Filtered
Filtered	Filtered
97770.53125	91416.76563
Filtered	Filtered
Filtered	5280.969727
91159.64844	87658.27344
105781.3594	102314.4063
Filtered	130119.1875
75414.59375	93881.91406
100170.5547	84340.53125
74524.65625	80055.32031
33537.70313	33156.65625
91309.26563	84506.54688
90555.1875	81508.64844
67472.0625	59735.65625
93558.54688	86777.66406
145200.5625	118469.75
69574	58537.92188
75556.59375	73163.625
Filtered	Filtered
97673.28125	88240.57813
Filtered	Filtered
53287.17188	49818.91797
115519.6797	106155.0938
144121.0625	66245.42188
76101.82031	74278.07031
Filtered	Filtered
89595.19531	69900.25781
Filtered	Filtered
64686.80078	52386.29688
72560.20313	66838.96875
68666.03125	65283.24219
74284.53125	61961.32422
36167.41406	38206.72656
3394.481689	3681.367432
107768.5469	47650.46484
67159.17188	59268.63281
13294.79004	57909.625
83685.64063	61026.11328
55237.73438	48740.57422
80950.65625	79417.46094
65640.14844	65872.14063
Filtered	53394.52734
86030.44531	74744.89063
73977.13281	50427.41016
Filtered	Filtered
40530.375	67273.35938
Filtered	Filtered
71560.88281	66698.1875
47546.44531	64793.23828
66138.64063	54623.63281
53857.85547	44681.56641
Filtered	Filtered
61661.56641	59689.79688
44179.60156	61827.49219
58944.33203	53778.1875
63617.13281	51977.36328
34227.96094	41287.40234
28480.87695	28077.21484
56256.44531	50082.86719
56342.80469	49779.71094
53650.59375	50490.17578
50598.62891	47109.59766
51238.05859	41933.28125

Filtered	46870.76953
49228.33203	39830.59375
23618.57227	34108.19922
Filtered	Filtered
44724.55859	44761.18359
41084.99219	41932.79688
54308.40234	48434.53516
48945.99219	Filtered
7309.04248	6324.993164
80481.3125	69218.59375
50286.86719	42095.60938
48676.86328	45680.74609
45280.53906	37125.94141
Filtered	Filtered
50893.22656	46178.88281
54997.64844	49306.13672
40491.70703	41893.55078
16316.89746	15837.8291
54345.61328	47186.44141
60022.35938	33638.99219
22819.32031	20218.88672
3614.461426	Filtered
53884.63281	37125.66016
46273.44141	42847.22266
1859.888428	484837.625
Filtered	Filtered
3729.221191	Filtered
71546.77344	Filtered
Filtered	Filtered
Filtered	Filtered
Filtered	Filtered
40217.46484	38177.60547
38252.19922	42914.71875
42730.83984	37086.10547
44815.55078	35914.90625
31805.26953	32832.21094
48573.47656	41970.91797
42158.5625	29489.47656
39154.94531	32467.52344
37922.91016	40089.48047
31283.23242	30707.95508
39438.03516	34532.13672
39507.125	35924.73828
37015.80469	21462.125
Filtered	Filtered
29632.66406	40096.24219
20856.63281	16850.59961
Filtered	Filtered
Filtered	Filtered
57239.63672	43270.83984
45474.16797	29507.2168
41310.38281	33819.53516
25073.21875	28478.41797
34166.4375	27882.2168
33997.05859	15752.13867
Filtered	Filtered
Filtered	Filtered
23144.61523	21518.45898
6346.745117	6418.387207
30212.35547	26862.6875
17831.19531	21325.71875
23663.02148	25538.7832
25882.17578	31377.21289
Filtered	Filtered
5644.100586	5069.474609
1870.036255	8677.542969
26706.19141	23515.23242
34766.94141	30623.48242
Filtered	Filtered
8645.554688	8312.766602
5559.475586	6544.94043
Filtered	Filtered
28650.00977	24674.57031
33399.12891	28323.32422
Filtered	Filtered

29886.94531	23385.80859
24793.26953	22907.48438
Filtered	29435.17773
28658.36914	26141.39453
16286.04297	Filtered
29148.07617	22304.71484
36737.86328	Filtered
33947.79688	25008.60742
3064.583008	44824.97266
110645.0547	27828.49219
30913.38672	30056.50781
25719.79688	23775.17773
48077.26953	21162.39844
18859.09375	26643
25678.85742	30001.64258
40374.24219	34358.95703
24368.72461	23623.39648
Filtered	8151.501953
13010.82129	10057.03418
Filtered	Filtered
27192.49219	25014.63672
Filtered	Filtered
24655.9375	26093.88086
25511.10547	22561.80859
52305.53516	15543.81152
22726.48047	20587.9707
Filtered	6076.507324
14660.70313	51977.23047
23670.375	20744.46875
25552.78906	19959.68555
24278.4375	22834.58594
Filtered	Filtered
19632.78906	22363.56055
Filtered	Filtered
22700.83594	19239.22656
22927.53906	19580.68164
20757.02734	18599.80078
27303.01953	23791.14453
Filtered	Filtered
19655.17188	17624.32422
8574.608398	6487.762207
17796.57422	14386.41309
22838.43359	19954.02539
27882.92383	26522.56836
10677.97266	11867.2041
26086.10938	21763.92188
21257.43555	18632.10938
21583.76172	19348
19947.23438	18769.85156
20949.89258	21136.10547
22609.21875	18998.22656
19983.57422	16329.32617
21634.64258	19582.58008
Filtered	Filtered
8403.651367	6753.515137
19792.88281	18051.10938
Filtered	Filtered
16038.12012	14326.52344
Filtered	28630.26758
17256.08594	26991.5293
Filtered	Filtered
17845.0293	18984.66992
14414.91309	12006.6084
21731.88867	19296.34375
18532.65039	20037.30664
Filtered	Filtered
13988.65625	9068.71582
23391.8418	18181.80664
20917.26563	18507.41602
Filtered	Filtered
15900.80273	12592.64258
19027.02344	18161.25
25850.62305	14314.45898
Filtered	Filtered
Filtered	Filtered

20504.2793	15154.4375
17560.35547	17286.92773
18612.5918	18186.68945
18076.31641	17643.29297
15275.35449	16045.43457
17689.47461	Filtered
23137.91602	16509.09961
16645.08203	14317.51172
Filtered	5962.515625
17796.98242	20482.15039
25242.47266	19663.74609
24367.40625	26105.36328
Filtered	28132.69531
30289.40234	29235.35742
18221.43555	19877.49609
18253.81641	14089.30859
19549.77148	17597.34766
20220.20508	14517.24121
9522.073242	12384.38477
15398.32324	15232.04785
13168.04297	17593.74414
14741.72656	14204.70117
17886.42578	15379.68945
Filtered	Filtered
Filtered	14139.8623
16564.08594	15154.99512
3367.765625	14893.83594
3665.487061	Filtered
12800.53223	13189.59863
16323.99023	15417.64844
Filtered	Filtered
22450.42383	24947.70508
17550.43555	14364.39844
47090.41406	6012.132813
8270.695313	11868.55469
16220.10449	10601.01563
Filtered	Filtered
19376.78711	18483.92773
13523.24414	14310.07422
17871.42578	14654.34277
27135.32227	24492.08594
12655.73047	10229.41895
Filtered	2091.126221
26103.63867	19695.91211
3706.475098	16635.16992
9768.5	25912.27734
17021.7832	Filtered
Filtered	Filtered
15563.73633	14577.25
16026.10742	12078.55859
16843.02539	15930.2832
21330.44727	15467.62891
11129.73926	16614.17383
Filtered	Filtered
15128.49414	12517.02734
12394.44434	11939.99023
19470.02344	18446.6582
Filtered	8902.134766
16296.68652	14210.91309
10711.59277	6194.651367
13920.77148	14501.63477
11644.38672	11805.66016
16323.47266	12502.78711
14740.4502	12515.31641
16332.93066	13005.125
Filtered	Filtered
Filtered	Filtered
Filtered	Filtered
Filtered	Filtered
19185.5918	14044.0918
Filtered	Filtered
14801.14844	12947.72168
10092.99512	9196.389648
11833.33203	15448.92969
Filtered	Filtered

Filtered	Filtered
Filtered	Filtered
12692.10547	17050.60352
15726.18359	13168.5332
7564.410156	5964.128906
15172.59668	14178.9541
12969.02734	16683.375
13202.48535	12011.87012
11778.51758	12168.43652
19683.39063	11971.58203
3326.471191	8457.576172
1.26E+04	1.08E+04
11222.07324	11712.95898
31801.97266	Filtered
13727.8418	11818.31543
Filtered	Filtered
Filtered	Filtered
4435.987793	6251.582031
10860.1543	13009.45313
Filtered	5693.285645
8533.18457	8007.780762
3869.520752	2562.781738
Filtered	Filtered
15168.63086	13899.33301
12879.82227	12510.35645
6313.783691	4017.771729
9672.682617	14891.70508
13612.74121	13439.16406
13113.29102	11075.34375
6324.233887	7367.152832
9360.933594	9404.621094
Filtered	4079.802002
15901.13086	11439.29688
Filtered	15224.75488
10316.90234	8276.655273
Filtered	Filtered
12477.63379	Filtered
Filtered	Filtered
5732.5	5435.213867
9370.889648	9310.441406
Filtered	Filtered
4062.682861	7483.322754
Filtered	Filtered
11505.20313	10241.87598
Filtered	Filtered
Filtered	Filtered
Filtered	Filtered
11996.52832	10634.44922
9737.591797	11477.15527
12544.32422	10557.56543
17639.95313	7299.273438
11014.09473	10478.68164
9701.987305	8470.067383
12753	11163.38379
Filtered	Filtered
11287.67969	10403.92578
15470.15234	7009.610352
12298.41211	9287.484375
3651.868896	11295.19727
10407.04102	9210.896484
Filtered	Filtered
2148.49292	Filtered
8894.263672	8301.806641
36457.16406	2716.897217
10415.1416	7947.186523
2784.283936	12710.38965
4437.433594	4501.692871
8593.516602	9179.019531
Filtered	5832.0625
5905.90918	8110.605957
4969.33252	4494.80127
8823.78418	6758.751465
Filtered	Filtered
Filtered	Filtered
Filtered	27073.04492

5440.614258	3365.551758
2099.950439	9883.503906
9603.119141	8070.388672
Filtered	Filtered
Filtered	Filtered
Filtered	7505.022949
Filtered	Filtered
Filtered	Filtered
7183.942871	6352.802734
10323.76074	8593.193359
Filtered	Filtered
Filtered	5613.407227
Filtered	Filtered
5480.565918	6799.422852
8369.607422	6674.076172
10009.52051	12355.86621
4297.12207	Filtered
7579.44043	9334.885742
Filtered	Filtered
Filtered	7468.082031
Filtered	8058.828125
Filtered	Filtered
5577.614746	3477.184326
Filtered	8848.790039
Filtered	Filtered
7927.109863	7967.512695
Filtered	Filtered
Filtered	4253.699707
Filtered	4905.343262
Filtered	Filtered
5429.808594	7436.646484
7253.132813	6393.900391
Filtered	Filtered
9172.458008	9218.839844
5712.816406	5467.890625
6867.856934	7209.028809
6379.675781	8110.575195
7865.99707	27456.55078
Filtered	3949.401123
9154.495117	7562.850586
Filtered	Filtered
5227.202637	6278.958496
7011.395508	6576.977539
Filtered	5420.050293
6226.679199	3995.95752
Filtered	Filtered
7477.892578	7779.882813
Filtered	Filtered
8466.961914	7515.707031
Filtered	Filtered
3420.861328	6478.071289
7431.487793	7655.04248
Filtered	3655.841797
6791.099609	5315.626465
4538.269043	6076.585938
5337.333984	5188.388672
5679.088379	6558.395996
Filtered	4675.04541
6143.143066	2495.463623
Filtered	Filtered
8936.833008	11607.48047
Filtered	2017.105591
Filtered	Filtered
5534.776367	5427.219727
4269.623535	Filtered
Filtered	Filtered
Filtered	Filtered
6341.834473	10675.19336
Filtered	Filtered
2102.173584	Filtered
6943.419922	4630.50293
7461.762207	7969.100098
Filtered	5104.119629
Filtered	Filtered
Filtered	Filtered

4741.085449	6153.947754
Filtered	Filtered
7451.96582	6455.373047
6849.296387	5972.813477
Filtered	Filtered
Filtered	Filtered
Filtered	4969.353516
Filtered	Filtered
Filtered	Filtered
Filtered	Filtered
Filtered	4462.960938
Filtered	Filtered
3915.88208	Filtered
Filtered	Filtered
4125.980957	Filtered
Filtered	4191.415039
5542.658691	5528.107422
8551.56543	6042.092285
5756.035645	4488.018555
Filtered	3373.210449
Filtered	Filtered
5244.776367	3514.679932
Filtered	Filtered
Filtered	Filtered
Filtered	Filtered
3479.150391	4519.635742
Filtered	Filtered
Filtered	Filtered
Filtered	Filtered
Filtered	Filtered
Filtered	3526.38501
3329.81958	3638.080078
3640.202881	3330.610352
Filtered	Filtered
Filtered	Filtered
7059.467285	3663.774902
Filtered	3901.621094
Filtered	Filtered
5827.928223	5152.799316
Filtered	Filtered
Filtered	Filtered
Filtered	Filtered
Filtered	Filtered
Filtered	Filtered
Filtered	Filtered
Filtered	Filtered
Filtered	Filtered
Filtered	Filtered
Filtered	Filtered
3805.053223	Filtered
5093.539063	3578.370361
Filtered	6459.969238
Filtered	Filtered
3308.888916	4876.945313
Filtered	3821.035889
Filtered	Filtered
Filtered	Filtered
6150.862793	5721.04541
4618.045898	Filtered
Filtered	Filtered
Filtered	Filtered
Filtered	Filtered
Filtered	Filtered
Filtered	Filtered
Filtered	Filtered
4500.318359	4625.212891
4763.415039	2967.173828
Filtered	Filtered
Filtered	Filtered
Filtered	4180.300781
3146.103027	3321.069824
4427.817383	3445.236816
Filtered	Filtered
Filtered	Filtered
Filtered	Filtered

Filtered	Filtered
Filtered	Filtered
Filtered	2565.14917
Filtered	3463.000488
Filtered	Filtered
Filtered	Filtered
Filtered	Filtered
Filtered	Filtered
Filtered	3931.04541
Filtered	Filtered
Filtered	Filtered
Filtered	Filtered
Filtered	Filtered
Filtered	4219.032227
Filtered	Filtered
Filtered	Filtered
Filtered	2965.18335
Filtered	Filtered
Filtered	Filtered
Filtered	3390.775146
3783.411133	Filtered
Filtered	3632.67627
3797.664551	3189.760742
Filtered	Filtered
Filtered	Filtered
Filtered	2372.310303
Filtered	Filtered
Filtered	Filtered
Filtered	Filtered
1485.027588	1250.166626
Filtered	Filtered
1670.429077	Filtered
2999.275879	3100.552979
Filtered	Filtered
1537.495605	Filtered
Filtered	Filtered
Filtered	Filtered
Filtered	Filtered
Filtered	Filtered
Filtered	Filtered
Filtered	Filtered
2567.608154	Filtered
Filtered	Filtered
Filtered	Filtered
Filtered	Filtered
Filtered	Filtered
Filtered	Filtered
Filtered	Filtered
Filtered	Filtered
Filtered	Filtered
Filtered	Filtered
Filtered	Filtered
Filtered	Filtered
508.361145	1584.267822
Filtered	Filtered
Filtered	Filtered
Filtered	Filtered
Filtered	Filtered
Filtered	Filtered
Filtered	Filtered
Filtered	Filtered
